# The Development of Stereoselective Substrate and Reagent-Controlled Lithiation–Borylation Chemistry

**DOI:** 10.1021/acs.joc.5c01854

**Published:** 2025-10-17

**Authors:** Yannick Linne, Maike Birkner, Daniel Lücke, Jan Flormann, Kjeld Gerdes, Giada Tedesco, Gaia Stojanovic, Tom Jentsch, Birk Jäger, Kevin Bajerke, Jörg August Becker, Markus Kalesse

**Affiliations:** a Institute of Organic Chemistry, Gottfried Wilhelm Leibniz Universität Hannover, Schneiderberg 1B, Hannover 30167, Germany; b Centre of Biomolecular Drug Research (BMWZ), Gottfried Wilhelm Leibniz Universität Hannover, Schneiderberg 38, Hannover 30167, Germany; c Institute of Physical Chemistry, Gottfried Wilhelm Leibniz Universität Hannover, Callinstraße 3A, Hannover 30167, Germany

## Abstract

Allylic alcohols are a privileged motif in polyketide-based natural product synthesis, and new methods that access them in a stereoselective fashion are highly sought after. Toward this goal, we found that the use of chiral polyketide fragments allows for performing the Hoppe–Matteson–Aggarwal rearrangement in the absence of sparteine with high yields and diastereoselectivities, rendering this protocol a highly valuable alternative to existing methods. Various stereodyads and -triads bearing different protecting and directing groups were investigated to determine their substrate induction. The mostly strong inherent induction was attributed to either steric or a combination of steric and electronic effects. The stereochemical outcome could be explained by (DFT-based) conformational analysis and a Felkin-like model, allowing guidance flowcharts to be created for the substrate- and reagent-controlled lithiation–borylation chemistry.

## Graphical abstract

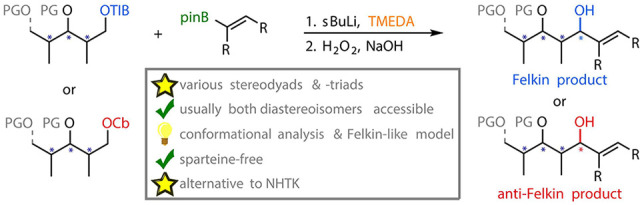

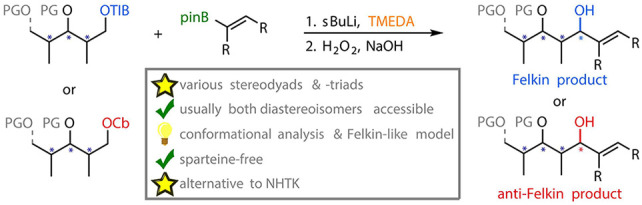

## Introduction

Chiral allylic alcohols are prominent structural motifs in natural products (Figure 1)^1^ as well as versatile synthetic building blocks.

**Figure 1:**
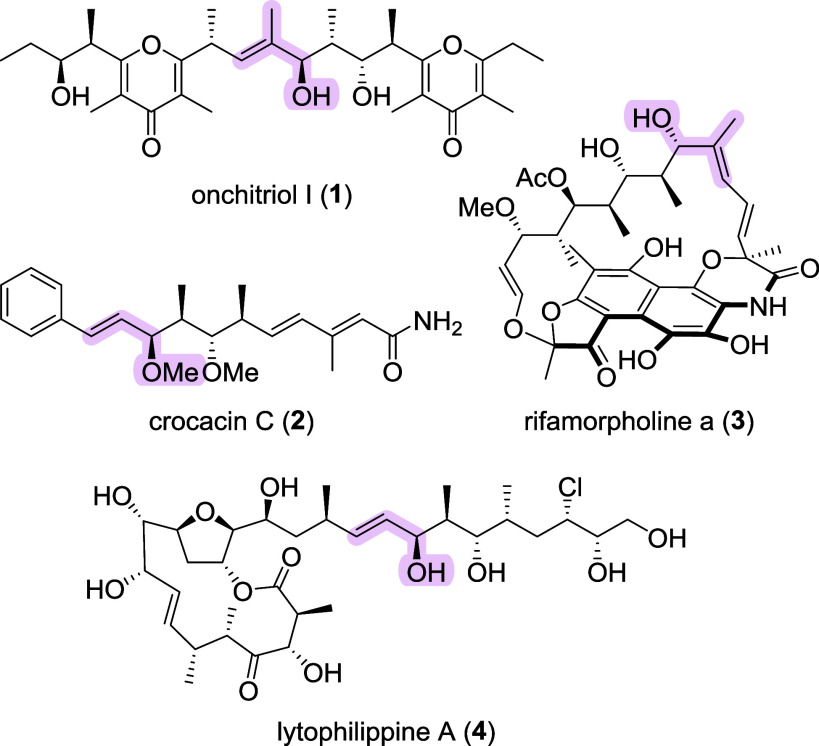
Natural products bearing chiral allylic alcohols or their derivative motifs.

A general way of accessing these motifs is the addition of a vinyl organometallic species to an aldehyde. Among other protocols, the Nozaki–Hiyama–Takai–Kishi reaction (NHTK reaction, Scheme 1a)^2^ is the method of choice for the asymmetric synthesis of chiral allylic alcohols. The high chemoselectivity toward aldehydes tolerates various other functional groups like ketones, acetals, nitriles, or esters. However, the obtained yields and selectivities are often moderate, especially for more complex substrates.^1b,2e,2f,3^ We recognized that the chemistry of lithiation and borylation developed by Hoppe, Matteson, and Aggarwal^4^ could serve as a platform for the stereoselective synthesis of chiral allylic alcohols. In contrast to the NHTK reaction, the polarities are interchanged as a vinyl boronic ester acts as the electrophile and is attacked by the anion of a masked alcohol (Hoppe anion). Even though the Hoppe–Matteson–Aggarwal rearrangement has been applied to the synthesis of numerous natural products,^4b^ no example for the synthesis of an allylic alcohol was given in the literature before we enrolled our program. During our research, we developed a protocol for the synthesis of chiral allylic alcohols by lithiation–borylation chemistry (Scheme 1b)^5^ and applied it in our total synthesis of the polyketide-peptide hydrid chondrochloren A (**8**) (Scheme 1c).^6^ Interestingly, the linkage of vinyl boronic ester (VBE) **6** and 2,4,6-triisopropylbenzoyl (TIB) ester **5a** provided alcohol **7** as a single diastereoisomer in the presence of achiral *N,N,N′,N′-*tetramethylethylenediamine (TMEDA), indicating a strong substrate induction for this transformation. Surprisingly, the formation of the opposite diastereoisomer *epi*-**7** was favored, when the TIB group was replaced by a *N*,*N*-diisopropyl carbamoyl (Cb) group. Even though the stereochemistry of the obtained secondary alcohol was inconsequential for our synthesis as it was oxidized later on, this finding served as a starting point for further investigations on directing effects in 1,2-metalate rearrangements (Scheme 1d).^7^ Apart from our striving for a deeper understanding of the origin of asymmetric induction, we also noticed the potential for even more synthetic applications of lithiation–borylation chemistry, if high levels of diastereoselectivity could be obtained in the absence of the expensive chiral diamine sparteine.^8^

**Scheme 1:**
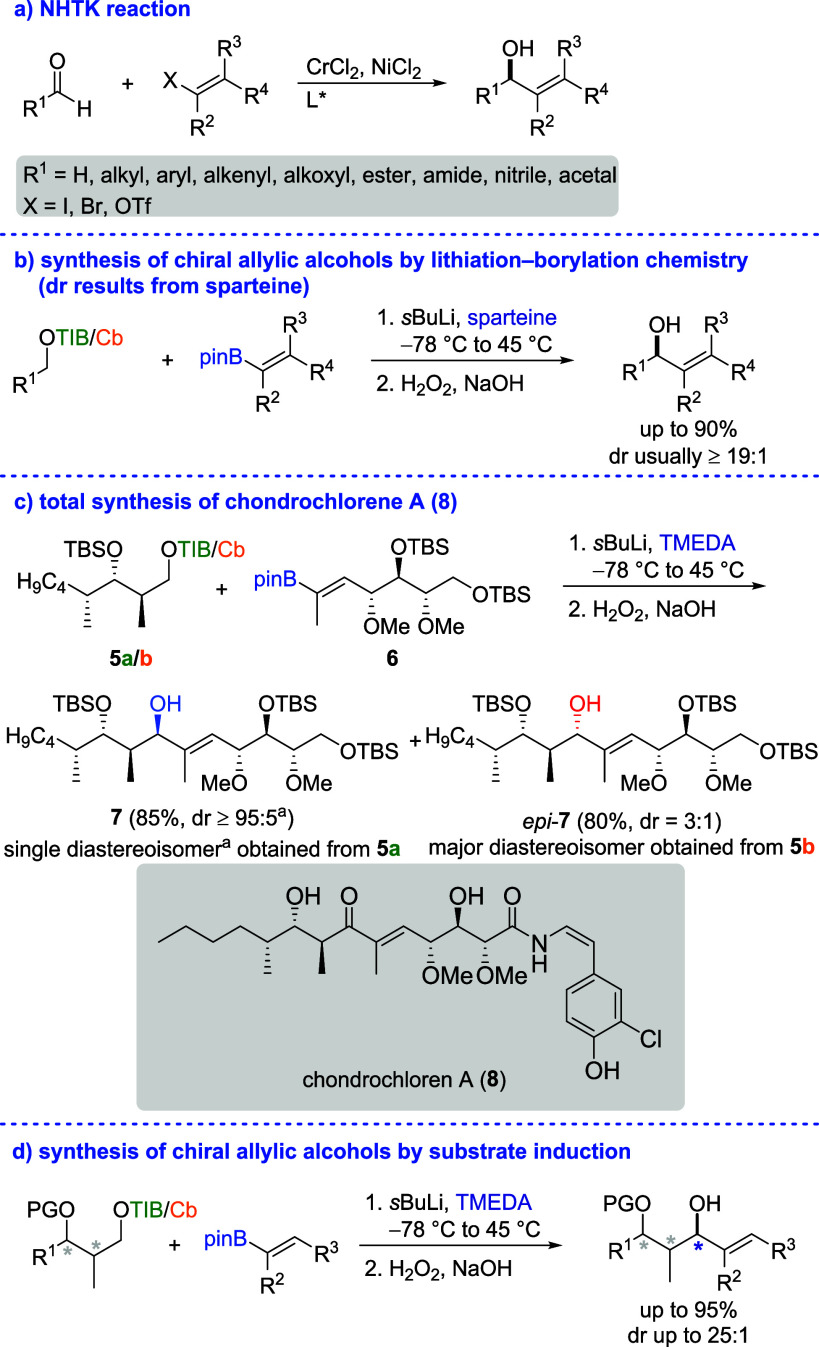
Synthesis of Chiral Allylic Alcohols by the NHTK Reaction and Lithiation–Borylation Chemistry

a Attributed to NMR accuracy.

Herein, we report a detailed overview of our investigations on substrate induction in lithiation–borylation chemistry for the synthesis of allylic alcohols. Parts of this work were previously published^7^ and are included to provide an overall overview. Furthermore, flowcharts for synthetic applications are given.

## Results and Discussion

We started our investigations with isobutyraldehyde derived diketides **9a**/**b** and **10a**/**b** as their isobutyl residue resembles a polyketidal framework (Figure 2). In our synthesis of chondrochloren A (**8**), we obtained a switch in selectivity when altering the directing group from TIB to Cb.^6^ To examine whether this is a general trend, both directing groups were utilized throughout the entire study. In addition, the influence of different conformations originating from a *syn* as well as an *anti* relation between the protected alcohol and the methyl branch was investigated. Vinyl boronic esters **11**–**14** were used as electrophiles (Figure 2). The more complex VBEs **11**–**13** representing the situation of branched/complex double bonds present in many natural products, whereas vinylboronic acid pinacol ester (**14**) was chosen to determine whether the size of the electrophile has an impact on the diastereoselectivity of the reaction.

**Figure 2:**
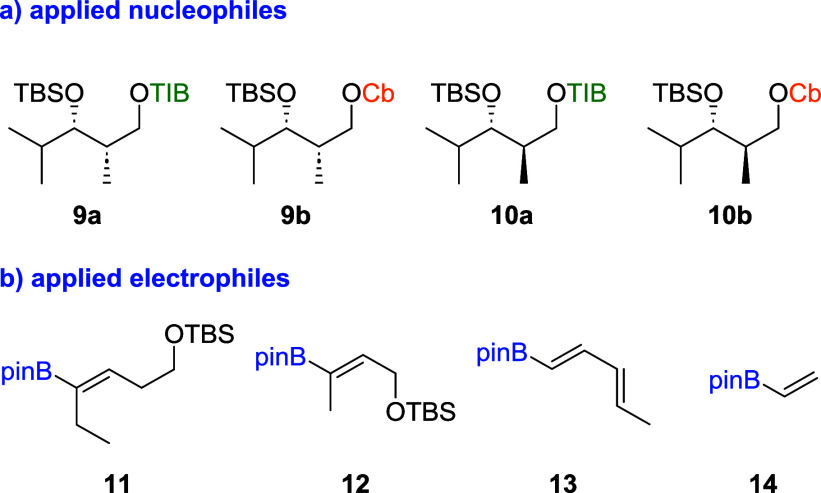
Nucleophiles and electrophiles applied to the first study.

For all reactions with the anion derived from TIB diketide **9a**, the formal Felkin products were obtained as the major diastereoisomers (Table 1, entries 1, 3, 5, and 7). Very good yields were obtained for branched vinyl boronic esters **11** and **12** (86 and 79%), whereas **13** and **14** provided the corresponding allylic alcohols in moderate yields (45 and 50%). In terms of selectivity, better results were obtained with the more complex VBEs **11**–**13** (dr starting from 10:1) compared to vinylboronic acid pinacol ester (**14**) (entry 7, dr 2:1). The low selectivity and moderate yield of the reaction of **9a** and **14** could be increased by the use of (+)-sparteine (entry 8), indicating a matched situation for this transformation. This was further supported when (+)-sparteine was replaced by its enantiomer (−)-sparteine (entry 9). In theory, the exchange of enantiomers should lead to the formal *anti*-Felkin product; however, formal Felkin product **18a** was obtained as the major diastereoisomer. The decrease in selectivity (dr 3:1) and yield (16%) indicates a mismatched situation. As both enantiomers of sparteine favor the formation of diastereomeric anions,^9^ this observation serves as a first indicator for an inversion during the borylation step. In comparison to TIB ester **9a**, lithiation–borylation of Cb carbamate **9b** proceeded in lower yields (entries 2, 4, 6, and 10). Branched VBEs **11** and **12** led to the formal *anti*-Felkin products in moderate yields (41 and 38%) and selectivities (4:1). Surprisingly, formal Felkin product **17a** was obtained in a good selectivity (dr 8:1) but very low yield (3%) from the reaction of dienyl boronic ester **13**. Transformation with less complex VBE **14** (entry 10) provided the formal *anti*-Felkin product in a good selectivity (dr 8:1) and moderate yield (31%). The *anti*-Felkin selectivity could be increased by the use of (−)-sparteine (entry 12). Interestingly, the use of (+)-sparteine resulted in the expected formal Felkin product **18a** as the sole diastereoisomer (entry 11), indicating a preference for retention during the borylation of Cb carbamates.^7a^

**Table 1:**
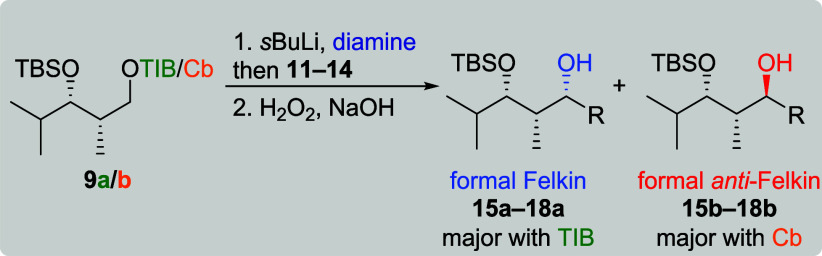

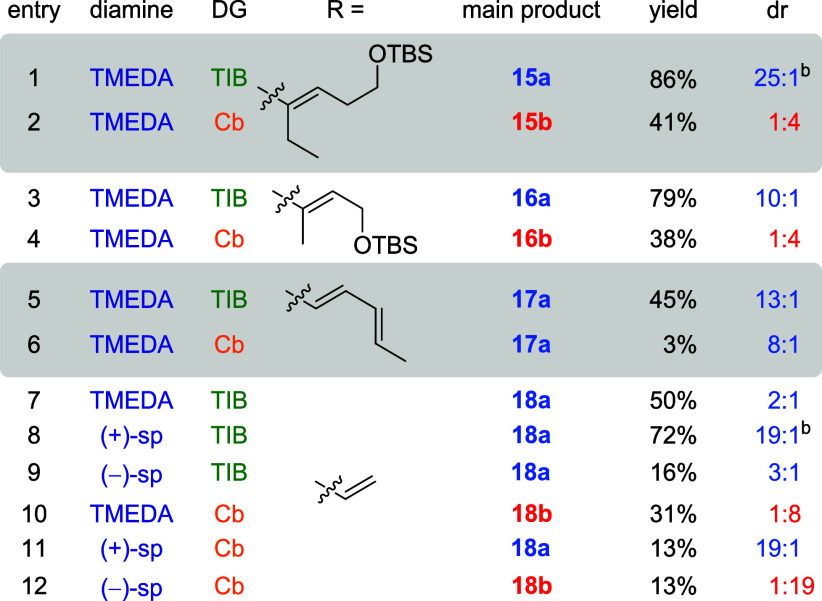
Substrate and Reagent Induction in Lithiation–Borylation Chemistry of *syn*-Diketides **9a**/**b** ^a^

a The results shown in this table are already published.^7a^ [a] General conditions: 1. TIB ester (1.5 equiv), diamine (1.5 equiv), *s*BuLi (1.4 equiv), Et_2_O, −78 °C, 5 h, and then vinyl boronic ester (1.0 equiv), Et_2_O, −78 °C, 3 h, and then 45 °C, o/n or carbamate (1.5 equiv), diamine (1.5 equiv), *s*BuLi (1.4 equiv), Et_2_O, −78 °C, 5 h, and then vinyl boronic ester (1.0 equiv), Et_2_O, −78 °C, 3 h, and then MgBr_2_·OEt_2_ (2.0 equiv), −78 °C, 30 min, and then 45 °C, o/n. 2. H_2_O_2_, NaOH, THF, −20 °C to rt.

b Attributed to NMR accuracy. sp: sparteine.

Comparable results were obtained when *anti*-diketides **10a**/**b** were applied to lithiation–borylation conditions (Table 2). For TIB ester **10a**, the formal Felkin products were obtained in all cases (entries 1, 3, 5, and 7). Again, branched vinyl boronic esters **11** and **12** performed well providing excellent selectivities (≥19:1) along with good to very good yields (79 and 69%). The reactions of linear VBEs **13** and **14** provided good to moderate yields (69 and 50%) and lower selectivities (dr 5:1 and 2:1). With Cb carbamate **10b**, the formal *anti*-Felkin product was obtained in all cases. Compared to the formal Felkin products, lower yields (11–60%) and much lower selectivities (approximately 2:1 for all VBEs) were obtained. It was again possible to fully override the *anti*-Felkin selectivity of the Cb carbamate for the reaction with VBE **14** when (−)-sparteine was used instead of TMEDA (entry 12, dr 19:1). Interestingly, all other reactions of *anti*-diketides **10a**/**b** with **14** in the presence of either enantiomer of sparteine (entries 8, 9, and 11) did not lead to any product formation, indicating a strong dependency between conformation of the (Hoppe) anion and the size of the diamine ligand.^7a^

**Table 2:**
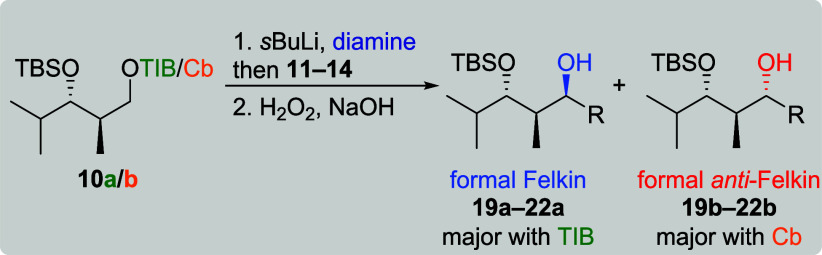

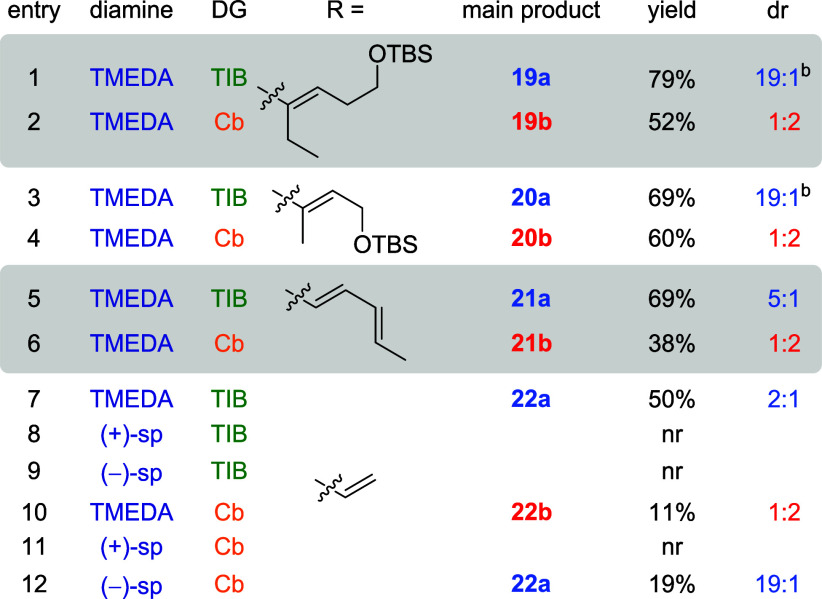
Substrate and Reagent Induction in Lithiation–Borylation Chemistry of *anti*-Diketides **10a**/**b** ^a^

a The results shown in this table are already published.^7a^ General conditions: Table 1.

b Attributed to NMR-accuracy. sp: sparteine. nr: no reaction.

For a better understanding whether the stereoselectivity originates from the lithiation or borylation step, the carbanions of all diketides were trapped as their corresponding trimethylstannanes.^7a^ This transmetalation process is known to proceed under full retention,^10^ which is a general trend for the reaction of nonmesomerically stabilized Hoppe anions with electrophilic reagents (e.g., TMSCl, CO_2_, Me_3_SnCl, MeI, or boronic acids).^11–14^ Thus, the diastereomeric ratios of the obtained stannanes should reflect the diastereomeric ratios of the carbanions. TIB ester **9a**-derived stannane **23** (Scheme 2a) was obtained in a low diastereoselectivity of 1.4:1 favoring the fomal Felkin product (configurations of all stannanes were determined by deprotonation with (+)-sparteine and (−)-sparteine, followed by addition of Me_3_SnCl; for more details, see the Supporting Information). The obtained low selectivity does not parallel the excellent results obtained for the reaction of the same carbanion with VBEs **11**–**13** (Table 1, entries 1, 3, and 5). This indicates that the overall stereoselectivity of this transformation is not controlled during lithiation, but the borylation step. For the selective formation of the formal Felkin product, the ate-complex formation most likely takes place under inversion and retention depending on the configuration of the carbanion. To further support this hypothesis, stannane **23** was subjected to Sn–Li exchange followed by addition of VBEs **11**–**14**. In all cases, the diastereomeric ratio of the obtained allylic alcohols increased compared to the applied stannanes, supporting the hypothesized inversion processes during the ate-complex formation step. The obtained selectivities of allylic alcohols **15a**, **16a**, and **18a** paralleled the results of the one-pot reaction. However, the formation of **17a** proceeded in a lower diastereoselectivity. These observations are in line with previous reports by Hoppe and Schleyer describing inversion and retention processes during the reaction of benzylic carbanions.^15^ They mentioned that electronic as well as steric effects determine which mechanistic pathway is chosen. The strong influence of sterics is highlighted in our work by the excellent selectivities obtained with sterically demanding electrophiles **11** and **12**. Stannane **24** derived from Cb carbamate **9b** was obtained in a diastereomeric ratio of 5:1 favoring the formal *anti*-Felkin product (Scheme 2b), which parallels the previously observed selectivities. Reliberation of the carbanion followed by borylation with VBEs **11**–**14** gave access to allylic alcohols **15b**–**18b**. The obtained diastereoselectivities of **15b**–**17b** are in accordance with **24**, indicating that the ate-complex formation takes place under retention of configuration. The higher formal *anti*-Felkin selectivity of **18b** can either be explained by partial inversion during the ate-complex formation or by the preferred reaction of the formal *anti*-Felkin carbanion. A preferred reactivity of this carbanion and partial decomposition of nonreacting formal Felkin carbanion could also serve as an explanation for the lower yield obtained for this transformation. Similar results were obtained for the carbanions derived from diketides **10a**/**b** (see the Supporting Information for more details), providing additional support for inversion and retention processes during the ate-complex formation of TIB ester-derived anions and mainly retention for the reaction of their Cb counterparts. A stronger tendency toward retention during the ate-complex formation of carbamate-derived anions could also be explained by a hypothesis made by Beak, who reported that ″highly reactive or nonlithium coordinating electrophiles proceed with inversion, while less reactive and lithium coordinating electrophiles give retention″.^16^ In our case, electrophile is exchanged by nucleophile, so both of Beak’s criteria could serve as an explanation for the observed reactivities. The lower reactivity of the Cb carbamates compared to the TIB esters is reflected by the need of MgBr_2_·OEt_2_ as an additional Lewis acid and elevated temperatures to initiate the 1,2-metalate rearrangement. The stronger coordination of lithium can be rationalized with the amide resonance.

**Scheme 2:**
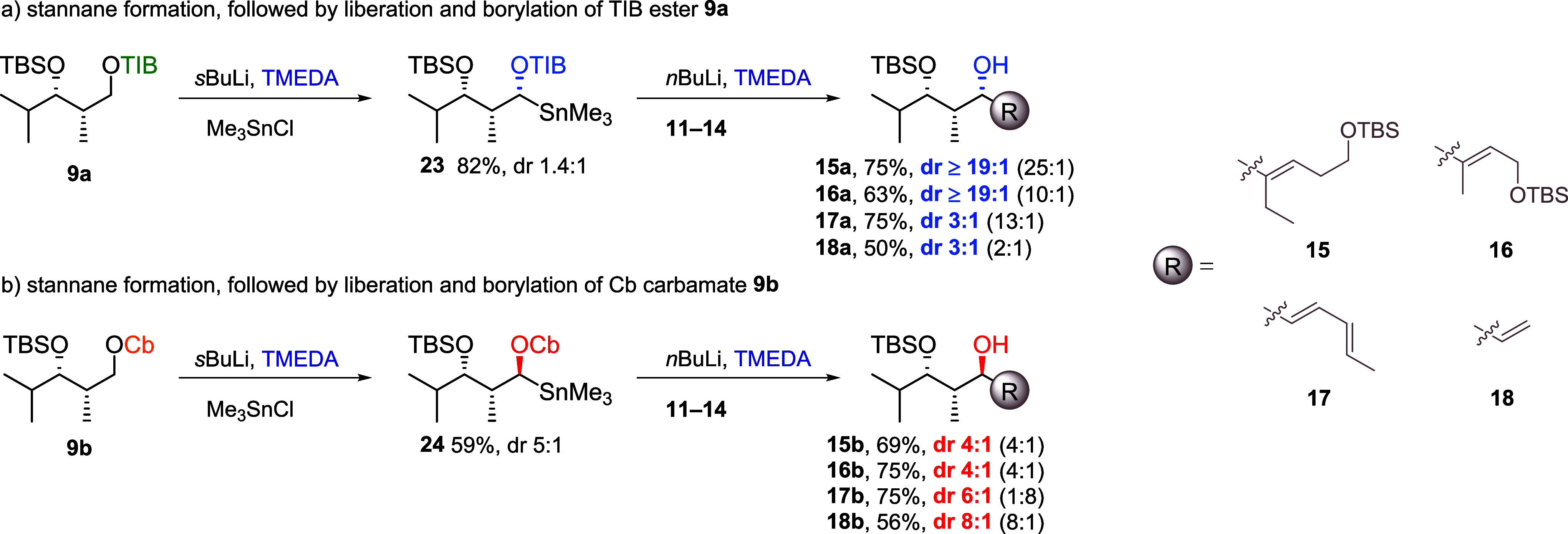
Stannane Formation Followed by Liberation and Borylation of **9a**/**b** ^a^

a Yields in Parentheses Refer to the One-pot Protocol. The results shown in this scheme are already published.^7a^

To be able to discuss the differences between the diastereomeric ratio of the formed carbanions and the obtained allylic alcohols in more detail, we decided to initiate a quantum chemical search for all four diketides (**9a**/**b** and **10a**/**b**). The results of this computational analysis were discussed in our initial study^7a^ and highlighted **9a**–**c1** and **9a**–**c2** as the most stable conformers of TIB ester **9a** at −78 °C. For both conformers, the carbonyl oxygen of the TIB ester points toward the formal *anti*-Felkin proton. This is in line with the assumption that the base is coordinated and directed by the carbonyl group prior to lithiation. In **9a**–**c1**, the proton in the *anti*-Felkin hemisphere is more easily accessible, which would lead to a preferred formation of anion **Li-9a-c1a**. However, in **Li-9a-c1a**, both hemispheres are difficult to access by an electrophile as the *anti*-Felkin hemisphere is shielded by the 2,4,6-triisopropylphenyl (TIP) group and the Felkin hemisphere is blocked by the isobutanol residue. A slight conformational change to **Li-9a-c1b** could lead to a free Felkin hemisphere supporting an inversion during the ate-complex formation. In conformer **9a-c2**, which is higher in energy than **9a-c1** but also populated at −78 °C, it is most likely that the conformation changes before deprotonation of the formal Felkin proton as the Felkin hemisphere is otherwise too hindered. Formed anion **Li-9a-c2** could then perform the borylation under retention of its configuration. The preferred deprotonation of the formal Felkin proton in **9a-c2** might be explained by its close proximity to the oxygen of the TBS-ether. An increased basicity/reactivity of protons close to silyl ethers was previously described by Knochel et al.^17^

In the case of Cb carbamate **9b**, the most stable conformer at −78 °C is **9b-c1**. Here, the *anti-*Felkin hemisphere is easier to access leading to **Li-9b-c1** as the preferentially formed anion, which could then react under retention of its configuration.

As the obtained yields and selectivities for the reactions of vinylboronic acid pinacol ester (**14**) were below all other VBEs, we decided to further optimize this transformation. During their synthesis of stemaphylline, Aggarwal and co-workers showed that a solvent swap drastically increased the yield of a 1,2-metalate rearrangement,^18^ so we decided to perform a solvent screening on the aforementioned transformation. *Anti*-diketides **10a**/**b** were chosen for the optimization studies as they performed weaker than their *syn*-analogs (Table 1, entries 7–12 vs Table 2, entries 7–12). When TIB ester **10a** was applied, a solvent switch from diethyl ether (previous conditions, Table 3, entry 1) to tetrahydrofuran (THF, entry 2) for the addition of **14** provided a comparable yield of 47% along with a much better diastereoselectivity of 6:1 favoring the formal Felkin product. Performing the entire reaction in THF, however, led to no product formation (entry 3). To keep the reaction operationally simple, we continued searching for a solvent performing well in the deprotonation as well as the metalate rearrangement step. Cyclopentyl methyl ether (CPME), which provided very good results in the study of Aggarwal et al.,^18^ led in our case only to traces of the product (entry 4). An increase in yield to 60% along with a diastereoselectivity of 5:1 favoring the formal Felkin product was obtained when the reaction was performed in methyl *tert*-butyl ether (MTBE, entry 5). Using a nonpolar solvent for the deprotonation (toluene) followed by addition of **14** in THF did not provide any product (entry 6). For Cb carbamate **10b**, only MTBE turned out to be suitable for the formation of formal *anti*-Felkin product **22b** (entry 11). However, the obtained yield and selectivity was slightly lower than our previous result (entry 7). With all other solvent combinations, no product formation or only traces of **22b** were observed (entries 8–10 and 12).

**Table 3:**
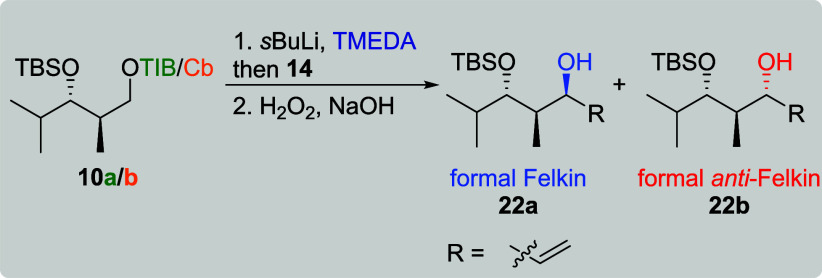

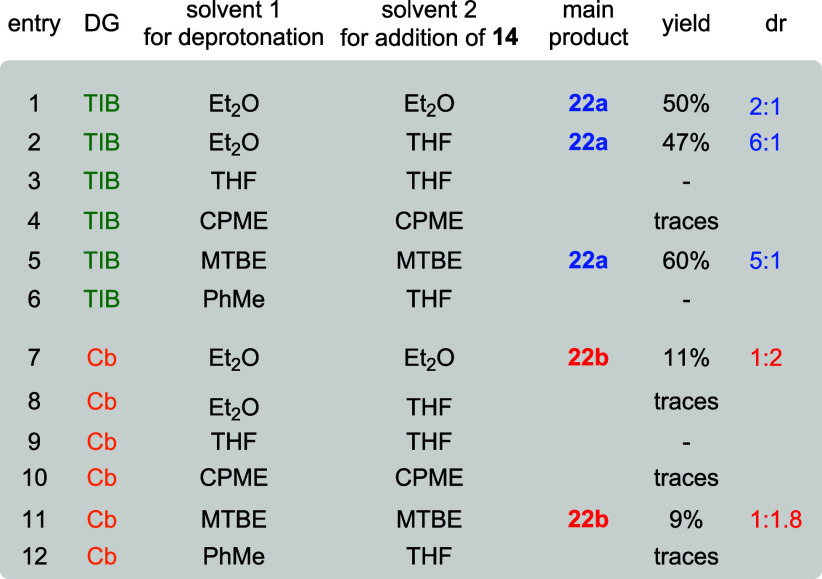
Solvent Screening for the Synthesis of Allylic Alcohols **22a**/**b** ^a^

a General conditions: (1) **10a** (1.5 equiv), TMEDA (1.5 equiv), *s*BuLi (1.4 equiv), solvent 1, −78 °C, 5 h, and then **14** (1.0 equiv), solvent 2, −78 °C, 3 h, and then 45 °C, o/n or **10b** (1.5 equiv), TMEDA (1.5 equiv), *s*BuLi (1.4 equiv), solvent 1, −78 °C, 5 h, and then **14** (1.0 equiv), solvent 2, −78 °C, 3 h, and then MgBr_2_·OEt_2_ (2.0 equiv), −78 °C, 30 min, and then 45 °C, o/n. (2) H_2_O_2_, NaOH, THF, −20 °C to rt.

We were also interested if the protecting group of the neighboring alcohol would have a strong impact on the substrate induction. Previous work by Hoppe showed that excellent diastereoselectivies could be obtained for the alkylation of carbanions if a chelating five-membered acetonide was present.^19^ Again, the reaction of VBE **14** with different protected *anti*-configured diketides was investigated first (Table 4). In comparison to our previous investigations (Table 2, entries 7–12), the *tert*-butyldimethylsilyl (TBS)-protecting group was replaced by a theoretically stronger chelating benzyl (Bn) or *para*-methoxybenzyl (PMB) group. For TIB diketides**25a** and **26a**, the formal Felkin products **27a** and **28a** were obtained in excellent selectivities (entries 1 and 3, dr 19:1) along with moderate yields of 45 and 54%, respectively. When the corresponding Cb carbamates **25b** and **26b** were used, the obtained major diastereomers were the formal Felkin products, respectively (entries 2 and 4), supporting an intramolecular coordination of the carbanion. However, the excellent selectivity of 19:1 was only obtained in the case of the PMB ether. In line with our previous results for the reaction of Cb carbamates with VBE **14** (Table 1 and 2, entries 10–12), low yields of 10% (Bn ether) respectively 4% (PMB ether) were obtained.

**Table 4:**
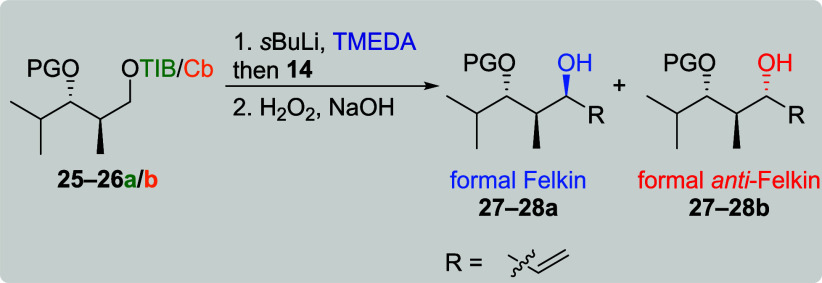

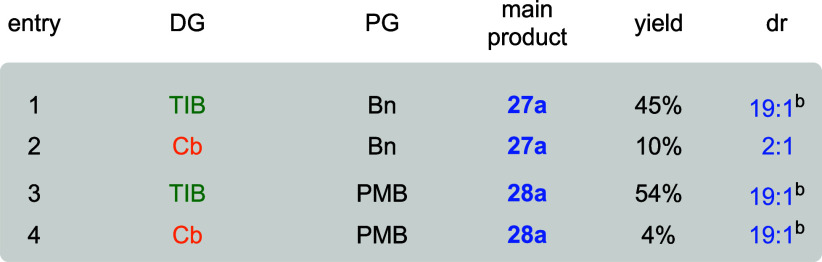
Screening of Different Protecting Groups for the Reaction of *anti*-Configured Diketides with VBE 14^a^

a General conditions: Table 1.

b Attributed to NMR accuracy.

Due to the improved selectivities obtained for the *anti*-configured diketides, their *syn* counterparts were investigated next (Table 5). TIB diketides **29a** and **30a** provided the formal Felkin products **31a** and **32a** in moderate yields along with moderate to low selectivities (entries 1 and 3). For Cb carbamates **29b** and **30b**, the formal *anti*-Felkin products **31b** and **32b** were obtained in low yields and moderate to good selectivities. These selectivities are comparable to the previously obtained results of TBS protected diketides **9a**/**b** (Table 1, entries 7 and 10) indicating less influence through coordination.

**Table 5:**
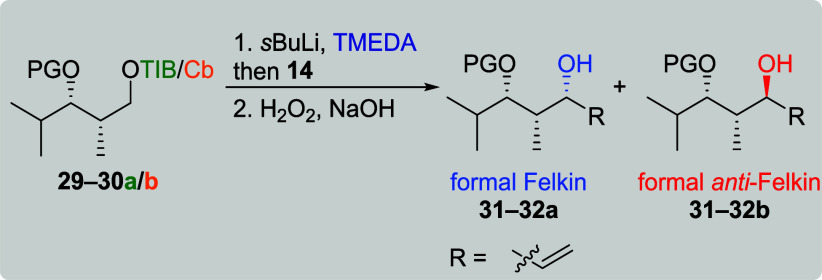

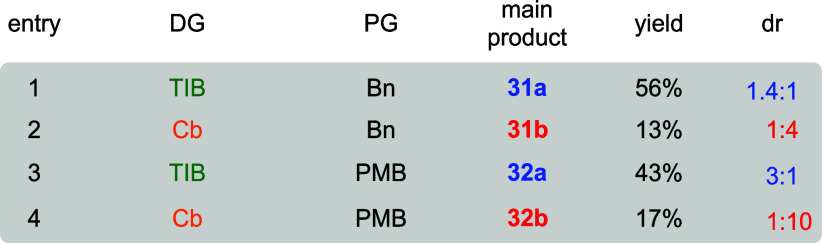
Screening of Different Protecting Groups for the Reaction of *syn*-Configured Diketides with VBE 14^a^

a General conditions: Table 1.

The difference in selectivity of the applied *anti*- and *syn*-diketides might be explained by intermediate carbanions (Scheme 5). Similar to the work of Hoppe,^19^ we propose intramolecular lithium coordination by the carbonyl group and the ether oxygen for *anti*-diketides **25a/b** and **26a/b** leading to bicyclic **Li-25**–**26-I** as favored carbanion. In line with experimental results, this carbanion would lead to the formal Felkin products. Diasteromeric carbanion **Li-25–26-II** might be formed as well but should be disfavored since for abstraction of the *anti*-Felkin proton, the chelate would have to adopt an energetically less favorable half-chairlike conformation, which is in line with previous observations of cyclic five-membered carbanions described by Knochel et al.^17c^ For *syn*-diketides **29a/b** and **30a/b** double coordination would lead to carbanion **Li-29**–**30**. However, 1,2-repulsion between the isopropyl and methyl group^20^ might favor carbanions similar to **9a/b** (Schemes 3 and 4), which would also explain the similarity of selectivities (Table 1, entries 7 and 10, and Scheme 5).

**Scheme 3:**
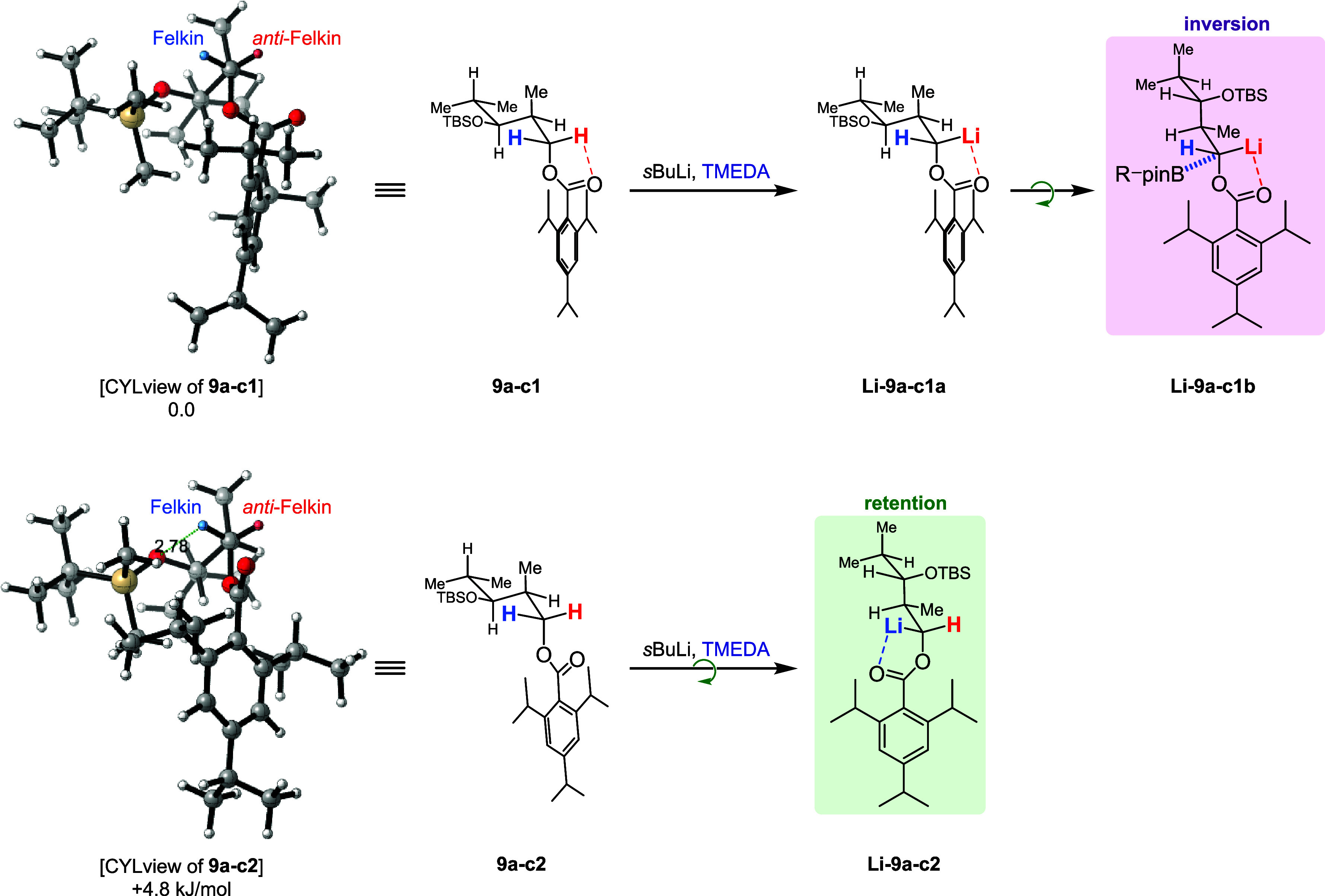
Rationalization of Inversion and Retention during the Borylation of TIB Ester **9a** ^a^

a The results shown in this scheme are already published.^7a^

**Scheme 4:**
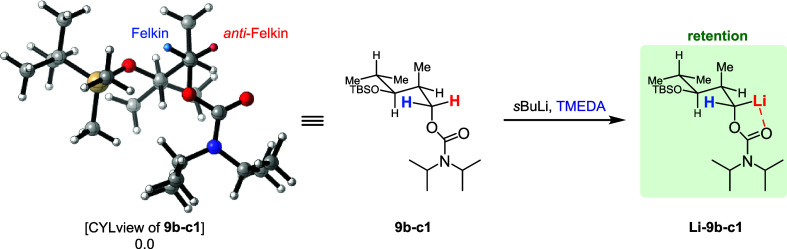
Rationalization of Retention during the Borylation of Cb Carbamate **9b** ^a^

a The results shown in this table are already published.^7a^

**Scheme 5:**
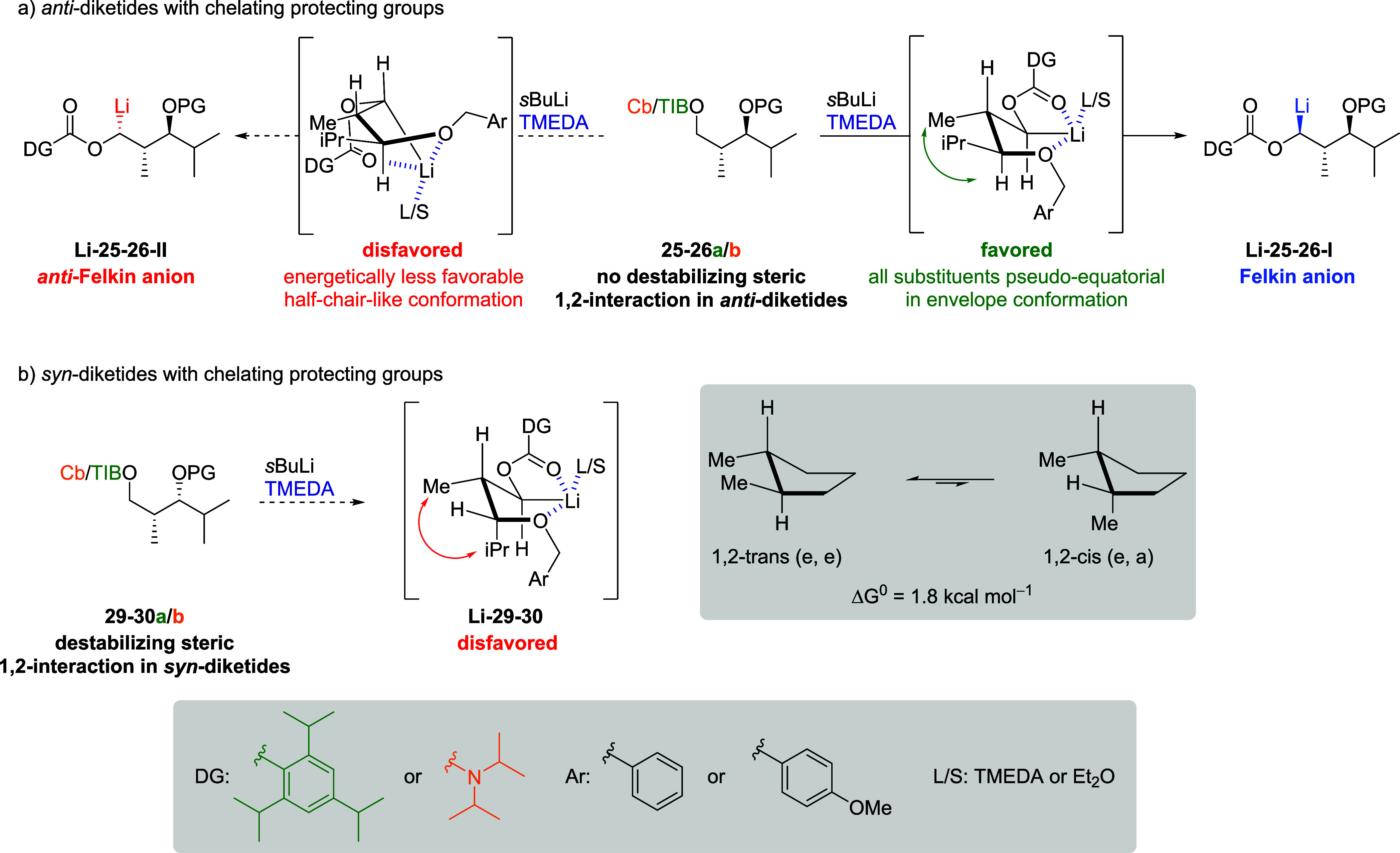
Proposed Carbanions Rationalizing the Observed Selectivities of Diketides **25**, **26**, **29**, and **30**

Interested in the effect of a less flexible conformation, we decided to investigate the substrate induction of acetonide-protected diketides **33a**/**b** and **34a**/**b** with VBEs **11**–**14** next (Figure 3).^7b^

**Figure 3:**
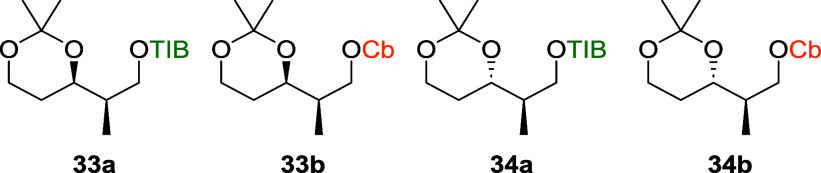
Investigated acetonide-protected diketides.

In contrast to the abovementioned five-membered acetonide utilized by Hoppe,^19^ we chose six-membered acetonides as their functional group distance parallels the situation in polyketides. Additionally, the acetonides in **33a**/**b** and **34a**/**b** do most likely adopt the chair conformation, so their level of substrate induction could be compared to previous work done by the Aggarwal group. In their synthesis of bastimolide B, the substrate induction of a six-membered *anti*-acetonide (twist-boat conformation) was investigated, which unfortunately showed nearly no asymmetric induction.^21^ For *syn*-configured TIB ester **33a**, the formal Felkin products **35a**–**38a** (Table 6, entries 1, 3, 5, and 7) were obtained in good yields (starting from 72%) and very good selectivities (dr starting from 10:1). This time, the reaction with vinylboronic acid pinacol ester (**14**) provided similar results as with all of the other VBEs. Surprisingly, Cb carbamate **33b** did as well provide the formal Felkin products although in lower selectivities (entries 2, 4, 6, and 8). In the presence of (+)-sparteine, the reaction of **33b** with **14** (entry 9) provided the formal *anti*-Felkin product **38b** in a moderate selectivity of 4:1 along with a low yield of 17%. In contrast to our previous results (Tables 1 and 2), this was the first time that the selectivity for the reaction of a carbamate in the presence of sparteine did not provide a dr of 19:1, indicating a mismatched situation between substrate and reagent control. However, for the reaction with (−)-sparteine (entry 10), the previously obtained excellent selectivities of 19:1 could be restored.^7b^

**Table 6:**
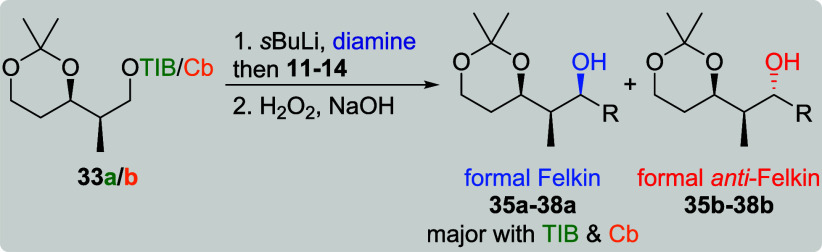

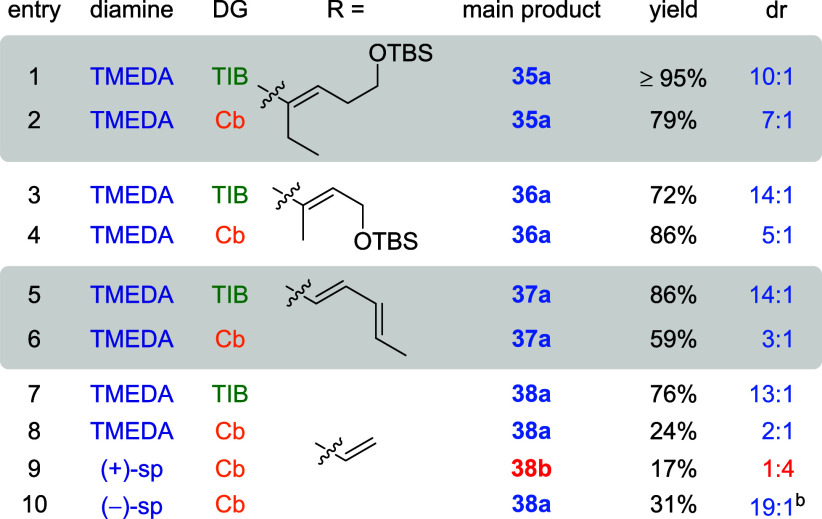
Substrate and Reagent Induction in Lithiation–Borylation Chemistry of *syn*-Diketides **33a**/**b** ^a^

a The results shown in this table are already published.^7b^ General conditions: Table 1.

b Attributed to NMR accuracy. sp: sparteine.

Compared to its *syn*-configured counterpart, *anti*-configured TIB ester **34a** provided the formal Felkin products **39a**–**42a** (Table 7, entries 1, 3, 5, and 7) in even higher selectivities (dr starting from 14:1) but lower yields (starting from 62%). For the reactions of Cb carbamate **34b** with more complex vinyl boronic esters **11**–**13** (entries 2, 4, and 6), the formal *anti*-Felkin products were obtained in moderate to good yields (52–66%) and low to moderate selectivities (dr 1:1.3–1:4). The reaction with **14** (entry 8), however, led to formal Felkin product **42a** in a low yield of 10% along with a low selectivity of 2:1. The presence of (+)-sparteine (entry 9) could fully overrule the substrate induction as formal *anti*-Felkin product **42b** was obtained in an excellent selectivity of 1:19 but a low yield of 7%. Interestingly, the presence of (−)-sparteine (entry 10) did not favor the formation of any diastereoisomer (dr 1:1).^7b^

**Table 7:**
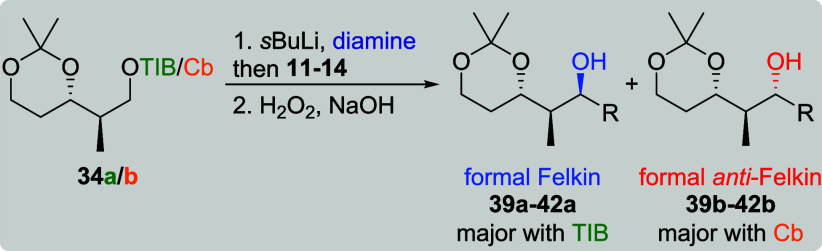

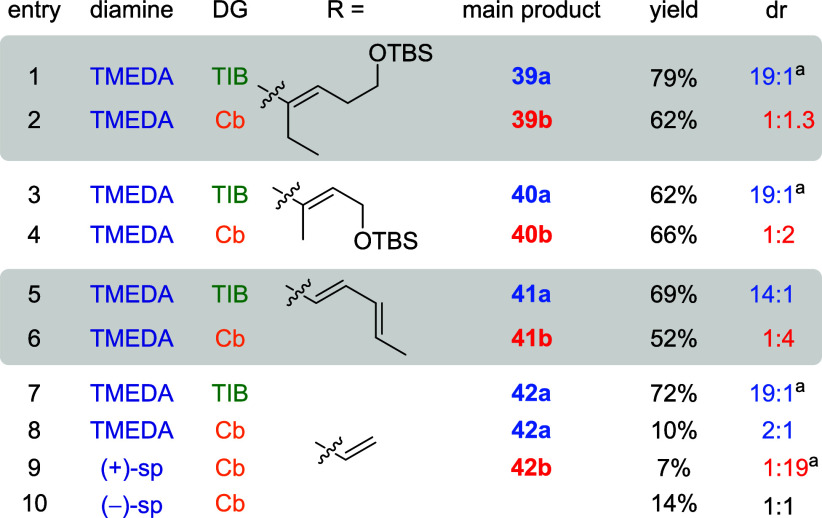
Substrate and Reagent Induction in Lithiation–Borylation Chemistry of *anti*-Diketides **34a**/**b** ^b^

a The results shown in this table are already published.^7b^ General conditions: Table 1.

b Attributed to NMR accuracy. sp: sparteine.

This time, the origin of the diastereoselectivity was investigated by deuteration of the carbanions of **33a**/**b** and **34a**/**b** (see the Supporting Information).^7b^ For **33a**, a dr of 6:1 was determined indicating that the borylation occurs again under retention and inversion. The diastereomeric ratio obtained for deuterated **33b** was 4:1, which is in good accordance with the results obtained for the reactions with **12** and **13** (Table 6, entries 4 and 6), indicating mainly retention during the borylation. However, the differences in selectivity obtained for the reactions with **11** and **14** could be explained by either partial inversion during the borylation or the preferred reaction of one carbanion and partial nonproductive decomposition of the other. Deuteration of **34a** provided a single diastereoisomer (dr ≥19:1). This result matches the selectivities obtained for **39a**, **40a**, and **42a** (Table 7, entries 1, 3, and 7), providing evidence for full retention during the borylation of these substrates. The slightly lower selectivity obtained for **41a** (entry 5) could be explained by partial inversion during this step. For Cb carbamate **34b**, no selectivity was observed during deuteration (dr 1.1:1), which is in line with the result of the reaction with VBE **11** (entry 2). The differing selectivities obtained for all other substrates (entries 4, 6, and 8) could again be explained by partial inversion during the borylation or the preferred reaction of one carbanion and partial nonproductive decomposition of the other. To explain the observed very good to excellent selectivities obtained for the transformations of TIB ester **33a** and **34a**, we proposed a model based on minimization of steric repulsion and stabilizing electronic effects^22^ (Scheme 6). For both TIB esters, we propose the deprotonation of the sterically less hindered proton. In the case of **33a**, this would lead to **Li-33a** as the intermediate carbanion. As the deprotonation of **33a** is not fully selective (dr 6:1), the diastereoisomer of **Li-33a** (not shown) is formed as well. In this, the carbanion would point toward the acetonide, which could explain a preference for inversion during the borylation step due to steric hindrance. In addition to this steric argument, orbital overlap could also stabilize conformer **Li-33a**. The C–Li σ-orbital overlaps with the σ*-orbital of the neighboring C–C bond, which in turn overlaps with the σ*-orbital of the neighboring O–C bond of the acetonide. Based on the same assumptions, the selectivities obtained for **34a** could be explained with **Li-34a** as the carbanion. The close proximity of the methylene group that forms the carbanion and the geminal methyl groups of the acetonide might also explain the high level of diastereoselectivity during the borylation.

**Scheme 6:**
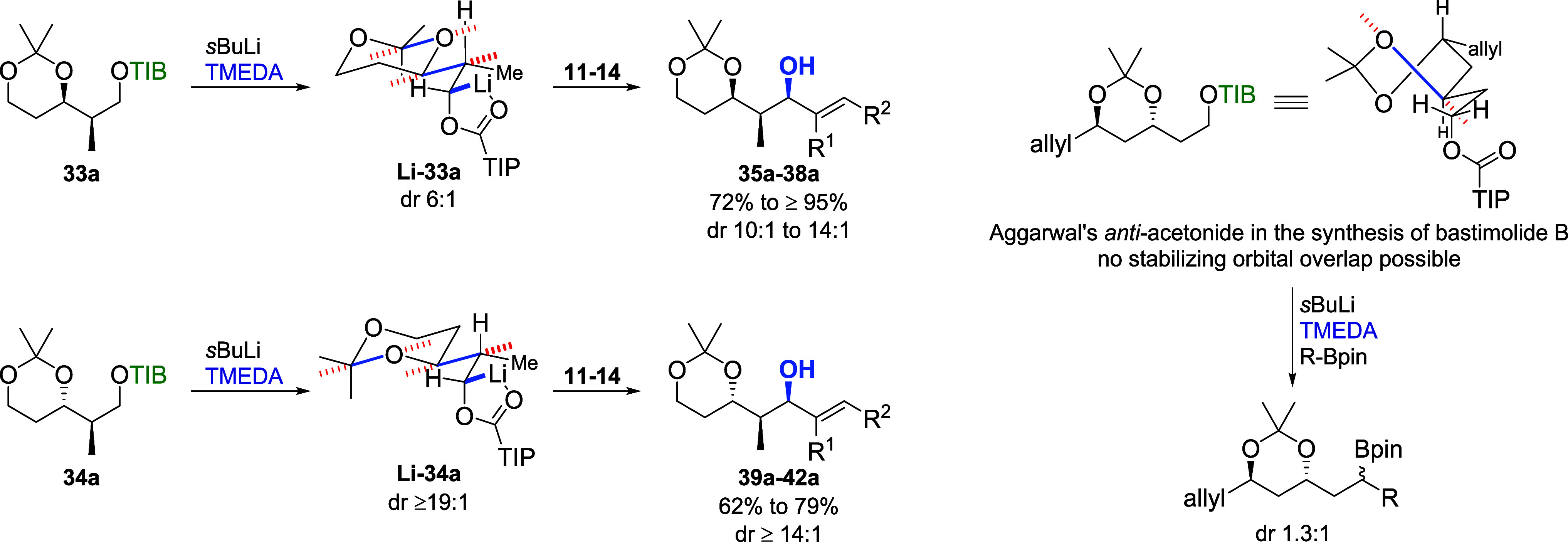
Proposed Transition States Rationalizing Our Observed Selectivities (σ*-Orbitals Are Indicated by Red Dotted Lines)^a^

a TIP: 2,4,6-triisopropylphenyl. The results shown in this scheme are already published.^7b^

When we compare our results to the abovementioned work of Aggarwal et al.,^21^ the huge differences in diastereoselectivity could also be explained with our model. In the twist-boat conformation of an *anti*-acetonide, steric interactions between the substituents might be less pronounced leading to a less defined transition state. Additionally, the stabilization by electronic effects is not given in a twist-boat conformation.

To summarize our studies on carbanions with two neighboring stereocenters (Figure 4), we differentiated our substrates by their conformational flexibility given by the used protecting groups first. For flexible substrates bearing a silyl ether as the protecting group, we obtained the formal Felkin products, when TIB esters were used as directing groups. In combination with large VBEs, excellent selectivities were obtained, which dropped to moderate, when small VBEs were applied. The selectivity for the reaction with small VBEs could be improved for *anti*-diketides, when coordinating protecting groups were used. However, this was only applicable to *anti*-diketides, since *syn*-diketides still provided moderate selectivities. For Cb carbamates, the formal *anti*-Felkin products were obtained in moderate selectivities regardless of the size of the VBEs. More rigid substrates bearing a six-membered acetonide as the protecting group provided the formal Felkin products in excellent selectivities for all applied TIB esters. When Cb carbamates were used, the selectivity dropped to moderate again, but the obtained major diastereoisomer did not always change to the formal *anti*-Felkin product.

**Figure 4:**
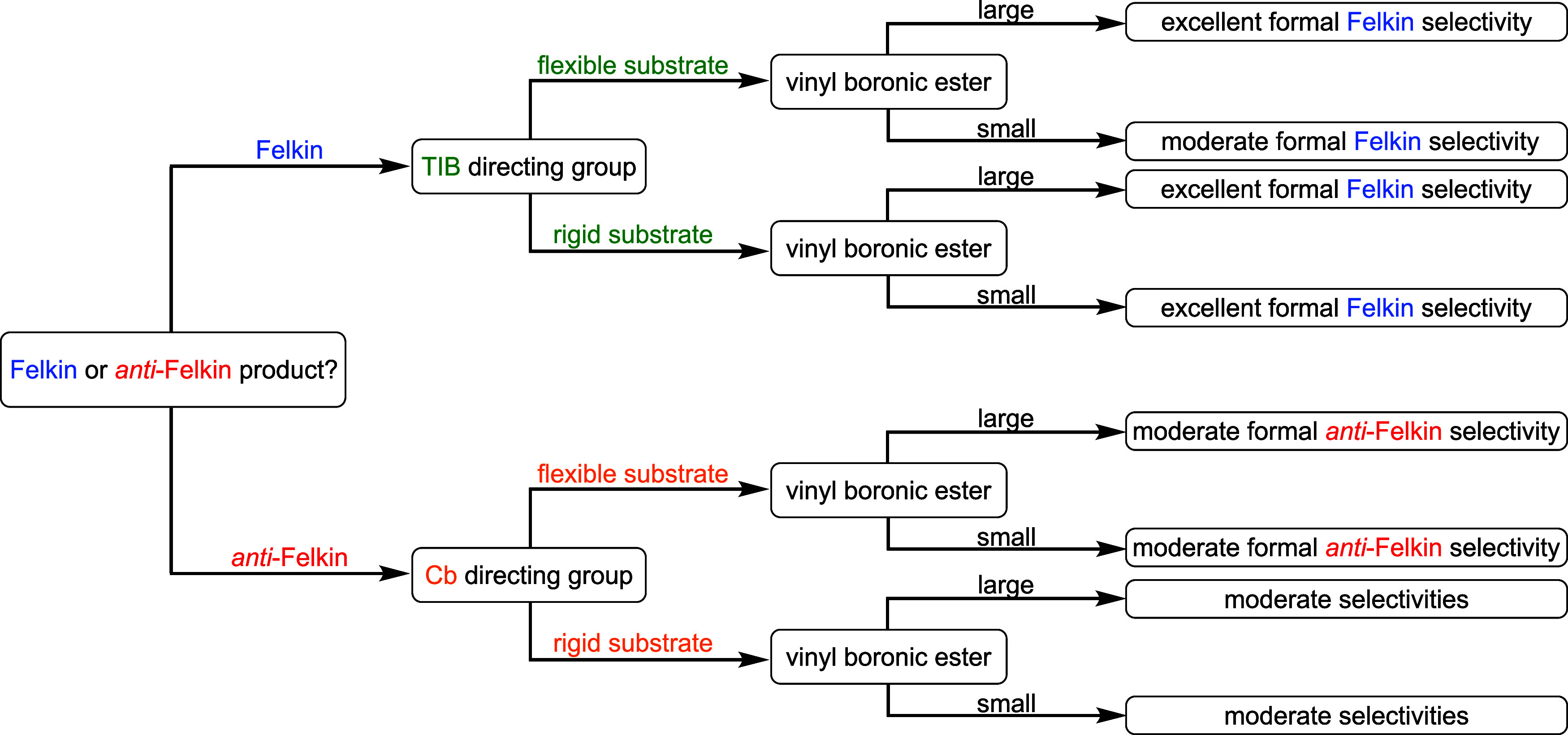
Guidance flowchart for the substrate- and reagent-controlled lithiation–borylation chemistry of diketides and VBEs.

Following our detailed investigations on diketides bearing two consecutive stereocenters next to the directing group, we were interested in the effect of an additional third stereocenter. The idea that remote stereocenters might affect the stereochemical outcome of the reaction was based on previous studies on aldol reactions, showing the impact of the overall conformation of substrates on their outcome.^23^ We started our investigations with acetonide-protected diketides,^7b^ as we estimated that the effect of distal stereocenters would be more pronounced in rigid systems due to potential *syn*-pentane interactions. The potential transition states of the acetonide-protected substrates with three contiguous stereocenters are shown below the corresponding table (Tables 8, 9, 10, and 11) and are based on minimization of *syn*-pentane interactions and removal of the less hindered proton as well as maximization of stabilizing electronic effects. A more detailed examination of these transition states is provided in our previous study.^7b^ We also decided to no longer apply four different vinyl boronic esters, but only VBEs **12** and **14**, as the results for both branched VBEs **11** and **12** were usually comparable. Additionally, vinylboronic acid pinacol ester (**14**) was chosen as it showed the lowest selectivities so far. All-*syn*-diketides **43a**/**b** (Table 8) provided the formal Felkin products as the major diastereoisomers. With TIB esters as directing groups (entries 1 and 2), excellent selectivities (dr 19:1) along with moderate to very good yields were obtained. In the case of Cb carbamate **43b** and VBE **14** (entry 3), the formal Felkin product was obtained in a moderate selectivity of 3:1 along with a low yield.^7b^

**Table 8:**
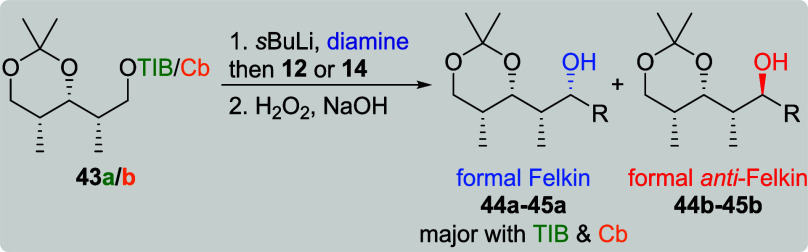

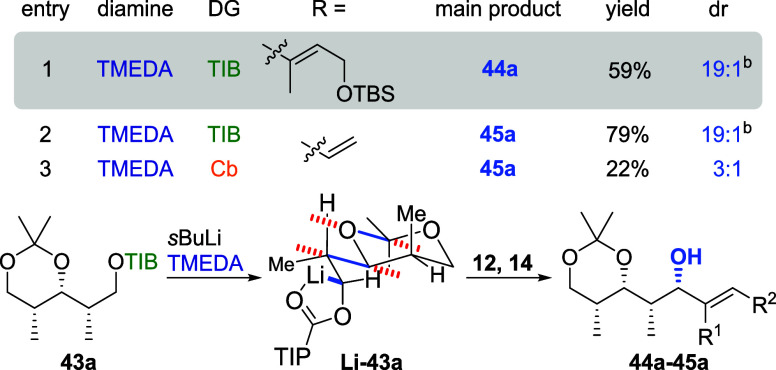
Substrate and Reagent Induction in Lithiation–Borylation Chemistry of All-*syn*-Diketides **43a**/**b** ^a^

a The results shown in this table are already published.^7b^ General conditions: Table 1.

b Attributed to NMR accuracy.

**Table 9:**
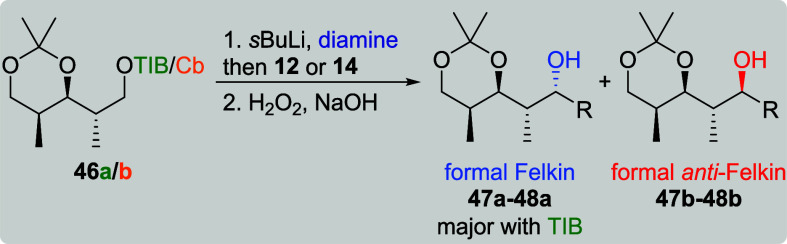

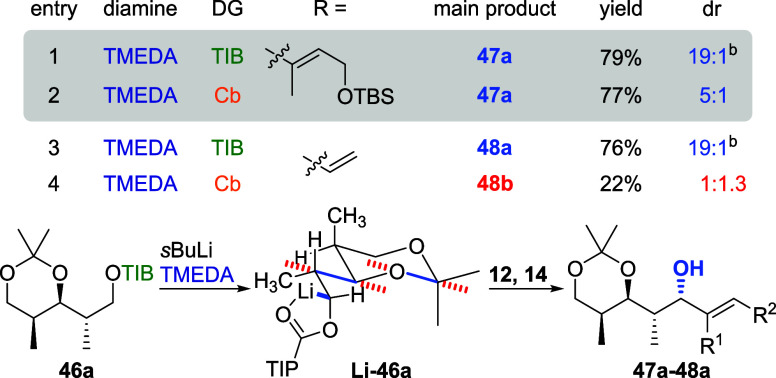
Substrate and Reagent Induction in Lithiation–Borylation Chemistry of *anti*, *syn*-Diketides **46a**/**b** ^a^

a The results shown in this table are already published.^7b^ General conditions: Table 1.

b Attributed to NMR accuracy.

**Table 10:**
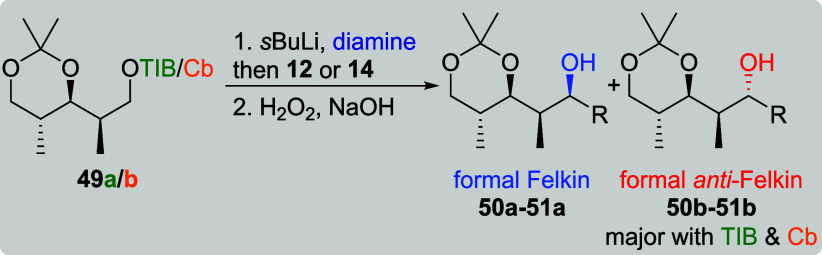

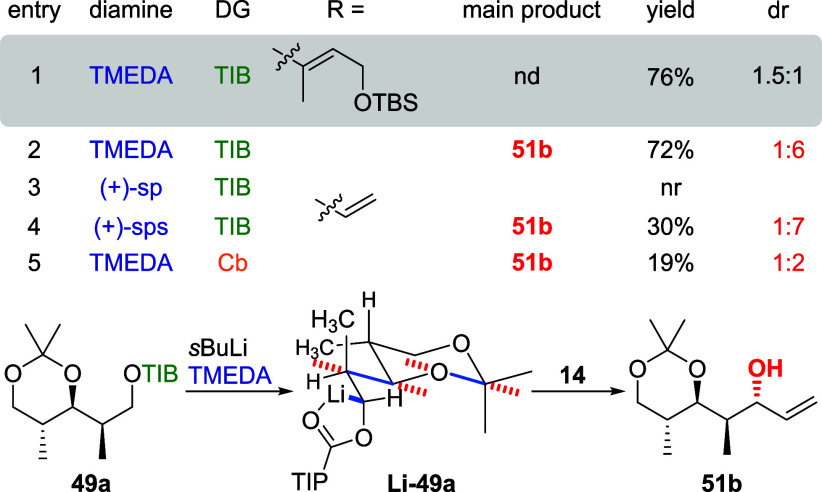
Substrate and Reagent Induction in Lithiation–Borylation Chemistry of *syn*, *anti*-diketides **49a**/**b** ^a^

a The results shown in this table are already published.^7b^ General conditions: Table 1. sp: sparteine. sps: sparteine surrogate. nr: no reaction.

**Table 11:**
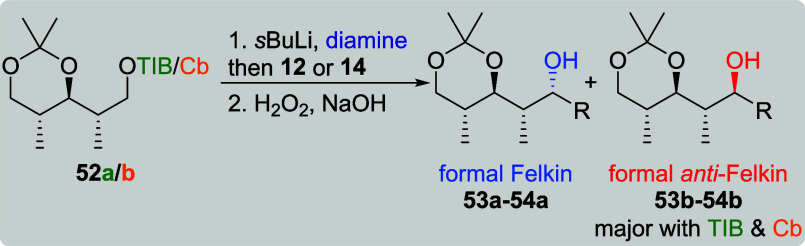

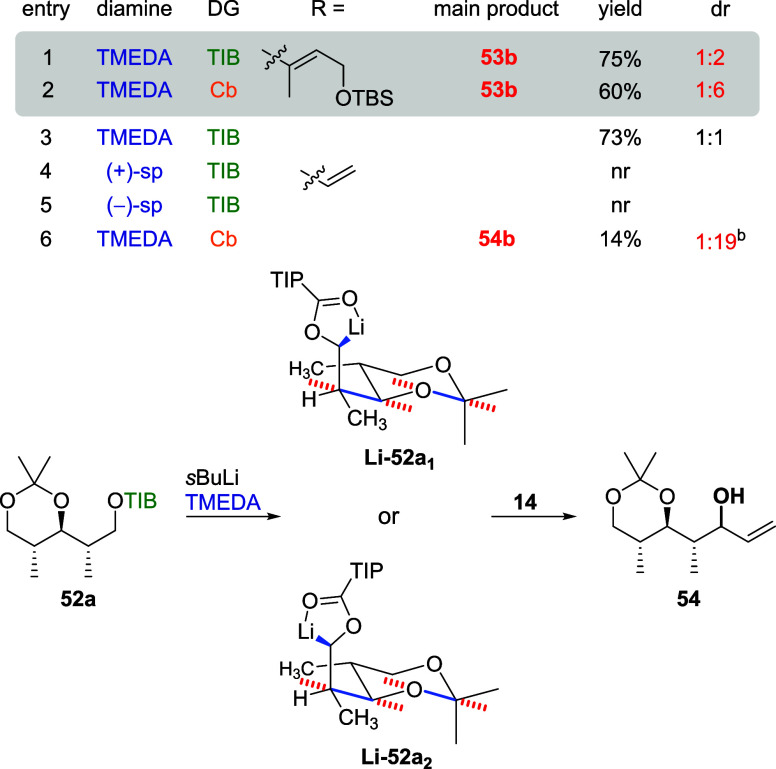
Substrate and Reagent Induction in Lithiation–Borylation Chemistry of *anti*, *anti*-diketides **52a**/**b** ^a^

a The results shown in this table are already published.^7b^ General conditions: Table 1.

b Attributed to NMR accuracy. sp: sparteine. nr: no reaction.

The reactions of TIB ester **46a** (*anti*, *syn*-diketide, Table 9, entries 1 and 3) with both VBEs led again to the formal Felkin products in excellent selectivities and very good yields. For its Cb counterpart **46b**, the formal Felkin product **47a** was obtained in a moderate selectivity and very good yield in the transformation with VBE **12** (entry 2). With unsubstituted vinylboronic acid pinacol ester (**14**) (entry 4), a slight preference toward the formal *anti*-Felkin product **48b** was observed. This low level of selectivity was accompanied by a low yield.^7b^

Surprisingly, nearly no selectivity was obtained for the reaction of *syn*, *anti*-TIB ester **49a** and methyl-branched VBE **12** (Table 10, entry 1).^7b^ As the TIB esters of acetonide-protected diketides usually provided the higher level of diastereoselectivity with sterically demanding VBEs, we did not perform this transformation with its Cb analogue. In the reaction with **14**, we obtained formal *anti*-Felkin product **51b** as the major diastereoisomer regardless of the directing group (entries 2 and 5). Interestingly, TIB ester **49a** provided a higher level of selectivity (dr 1:6) along with a much higher yield (72%) compared to Cb carbamate **49b** (1:2, 14%). The selectivity for the reaction of **49a** and **14** could be slightly increased to 1:7, when TMEDA was replaced by the (+)-sparteine surrogate (entry 4). It is noteworthy that no product formation was observed, when the bulkier (+)-sparteine was used as diamine ligand (entry 3), which further supports that the size of the ligand plays a crucial role for this transformation.^24^

Formal *anti*-Felkin product **53b** was obtained as the major diastereoisomer in the reaction of methyl-branched VBE **12** and both *anti*, *anti*-diketides **52a**/**b** (Table 11, entries 1 and 2). The *anti*, *anti*-configuration is a rare example where the Cb carbamate did provide a higher level of diastereoselectivity than the TIB ester (1:6 vs 1:2). However, the yield was higher for the reaction of TIB ester **52a** (75% vs 60%). For the reaction of **52a** with VBE **14** (entry 3), no diastereoisomer was favored (dr 1:1). Attempts to increase the diastereoselectivity by the use of sparteine (entries 4 and 5) failed due to no product formation. In contrast to these results, Cb carbamate **52b** (entry 6) led to the formation of formal *anti*-Felkin product **54b** in an excellent selectivity (1:19) but low yield.^7b^

Taking these results as evidence that distal stereocenters affect the stereochemical outcome of this reaction, we next turned our attention to more flexible stereotriads bearing two silyl ethers. For all-*syn*-TIB ester **55a**, a high level of diastereoselectivity was obtained in the reaction with branched VBE **12** (13:1, Table 12, entry 1) leading to formal Felkin product **56a**. The same VBE provided no level of selectivity (dr 1:1), when Cb carbamate **55b** was used (entry 2). With **14**, both diketides led to formal *anti*-Felkin product **57b** as the major diastereoisomer (entries 3 and 4). The yields of both reactions were comparable; however, Cb carbamate **57b** provided the higher level of diastereoselectivity (1:3 vs 1:1.2). In accordance with previous results for more flexible Cb carbamates (Table 1, entries 11 and 12, and Table 2, entry 12), a full level of stereocontrol was obtained, when the reaction was performed in the presence of sparteine (entries 5 and 6).

**Table 12:**
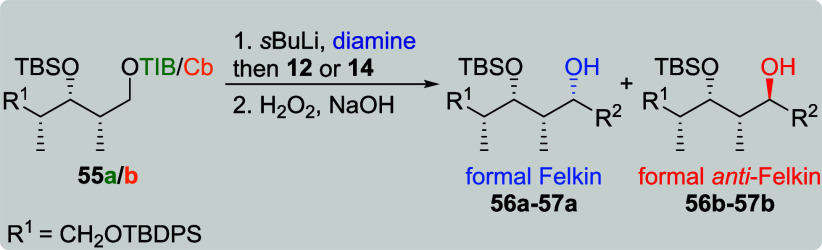

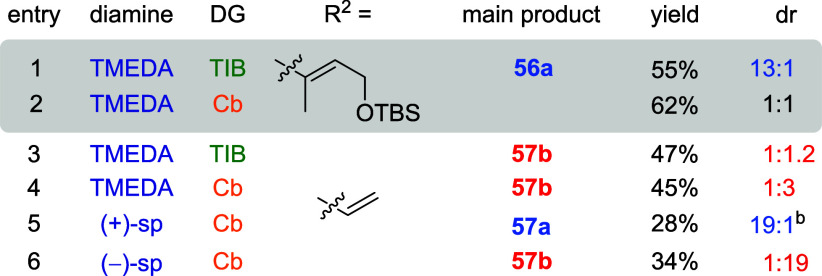
Substrate and Reagent Induction in Lithiation–Borylation Chemistry of All-*syn*-diketides **55a**/**b** ^a^

a General conditions: Table 1.

b Attributed to NMR accuracy. sp: sparteine.

In the reaction of *anti*, *syn*-TIB ester **58a** and VBE **14**, formal Felkin product **59a** was obtained as the sole diastereoisomer (Table 13, entry 1). This is the second example for an excellent selectivity of an *anti*, *syn*-configured TIB ester, as this structural motif was also present in our synthesis of chondrochloren A (**8**) (Scheme 1c).^6^ Based on this observation and the excellent result for the reaction of **58a** and **14**, we did not investigate the combination of **58a** and **12**. The reaction of Cb carbamate **58b** and **14** (entry 2) provided the formal *anti*-Felkin product in a slight excess (dr 1:1.2). Attempts to increase the selectivity of this reaction by the addition of sparteine (entries 3 and 4) failed due to no product formation.

**Table 13:**
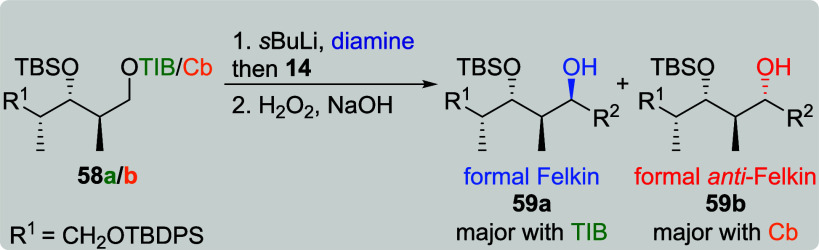

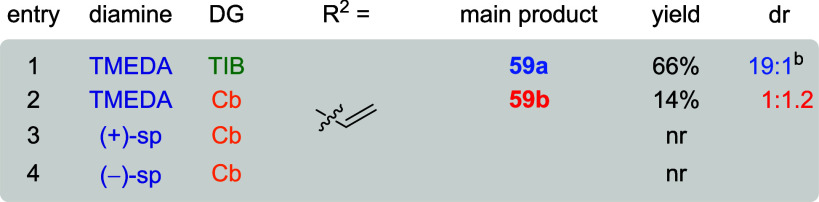
Substrate and Reagent Induction in Lithiation–Borylation Chemistry of *anti*, *syn*-Diketides **58a**/**b** ^a^

a General conditions: Table 1.

b Attributed to NMR accuracy. sp: sparteine. nr: no reaction.

Both *syn*, *anti*-diketides **60a**/**b** provided moderate yields in the reaction with branched VBE **12** (Table 14, entries 1 and 2). For TIB ester **60a**, formal Felkin product **61a** was obtained in a good selectivity (dr 11:1). Its Cb counterpart **60b** led to formal *anti*-Felkin product **61b** in a good selectivity (dr 1:11) as well. Interestingly, TIB ester **60a** and VBE **14** gave formal *anti*-Felkin product **62b** in a low selectivity (dr 1:2, entry 3). The formal Felkin selectivity of the TIB ester was restored by the use of (−)-sparteine (entry 5), while in contrast to this result, the use of (+)-sparteine (entry 4) did not lead to any product formation. Formal *anti*-Felkin product **62b** was obtained as a single diastereoisomer in the reaction of Cb carbamate **60b** and VBE **14** (entry 6). The same level of diastereoselectivity along with a lower yield was obtained, when (+)-sparteine was used (entry 7), providing more evidence for the impact of the size of the diamine ligand on the yield of the reaction.

**Table 14:**
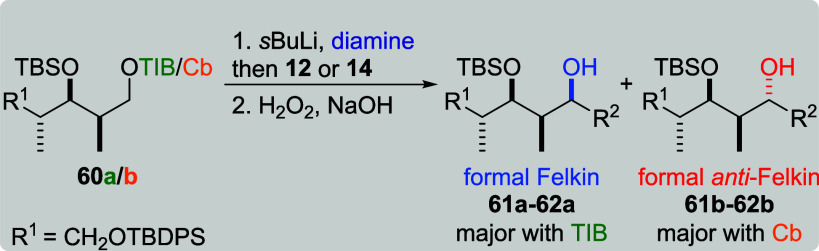

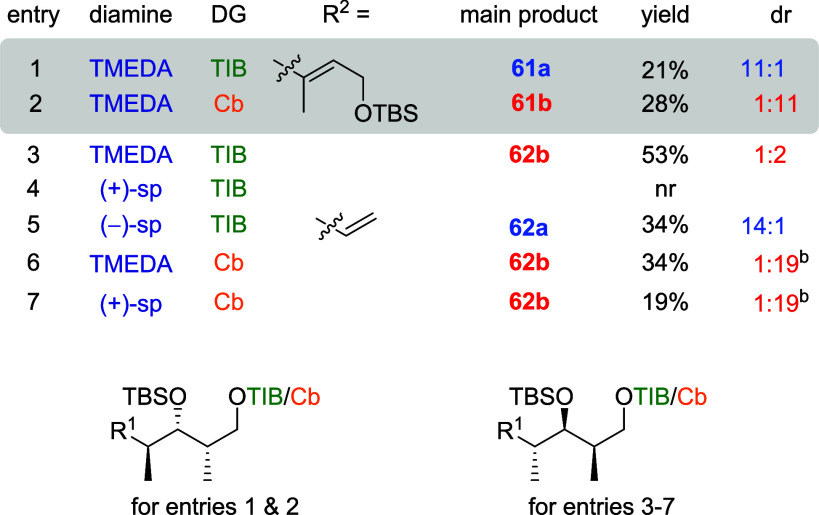
Substrate and Reagent Induction in Lithiation–Borylation Chemistry of *syn*, *anti*-Diketides **60a**/**b** ^a^

a General conditions: Table 1.

b Attributed to NMR accuracy. sp: sparteine. nr: no reaction.

For the *anti*, *anti*-configuration (Table 15), formal Felkin product **64a** was obtained in a good selectivity (dr 10:1), when TIB ester **63a** was combined with **12** (entry 1). In combination with Cb carbamate **63b** (entry 2), formal *anti*-Felkin product **64b** was obtained in a low level of diastereoselectivity (dr 1:2). Simple vinylboronic acid pinacol ester (**14**) and TIB ester **63a** (entry 3) provided formal Felkin product **65a** in a slight excess (dr 2:1). The selectivity of this reaction could not be changed by the use of sparteine (entries 4 and 5) due to no product formation. In the reaction with Cb carbamate **63b** (entry 6), the formal *anti*-Felkin product was obtained as a single diastereoisomer.

**Table 15:**
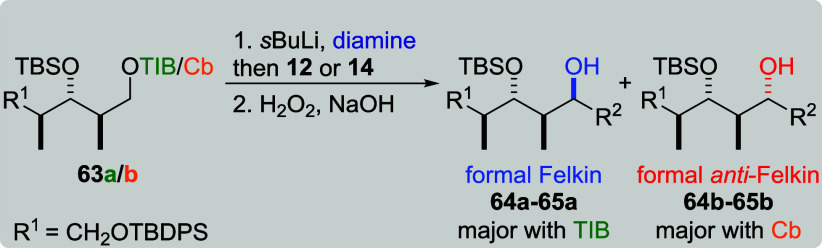

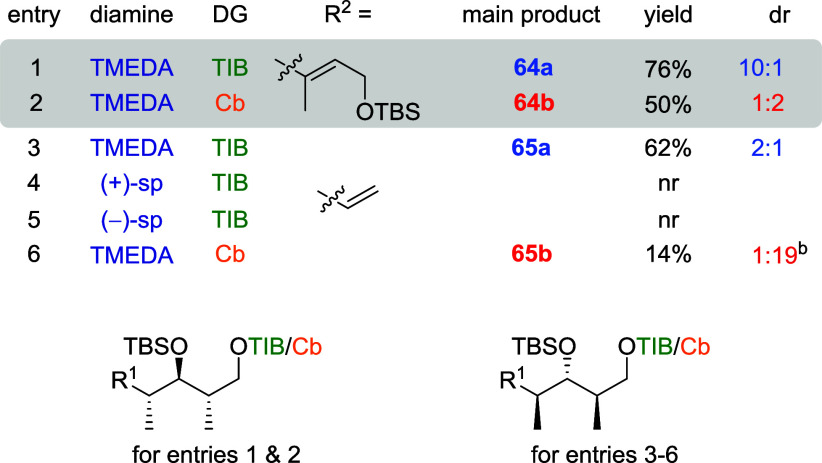
Substrate and Reagent Induction in Lithiation–Borylation Chemistry of *anti*, *anti*-Diketides **63a**/**b** ^a^

a General conditions: Table 1.

b Attributed to NMR accuracy. sp: sparteine. nr: no reaction.

Summarizing our work on TIB diketides bearing three contiguous stereocenters (Figure 5), we observed excellent formal Felkin selectivities for rigid substrates, when the stereocenters at C3 and C4 were *syn*-configured. These selectivities were obtained regardless of the size of the used vinyl boronic esters. However, with an *anti*-relation between C3 and C4, only moderate selectivities were obtained with both VBEs. Additionally, there was no general trend favoring either the formal Felkin or formal *anti*-Felkin product for these substrates. More flexible substrates provided excellent formal Felkin selectivities with large vinyl boronic esters regardless of the stereochemical relation between C3 and C4. For small VBEs, a moderate formal Felkin selectivity was obtained, when an *anti*-configuration between C3 and C4 was present. For a *syn*-relation between C3 and C4, no preference regarding the formation of either the formal Felkin or formal *anti*-Felkin product could be observed.

**Figure 5:**
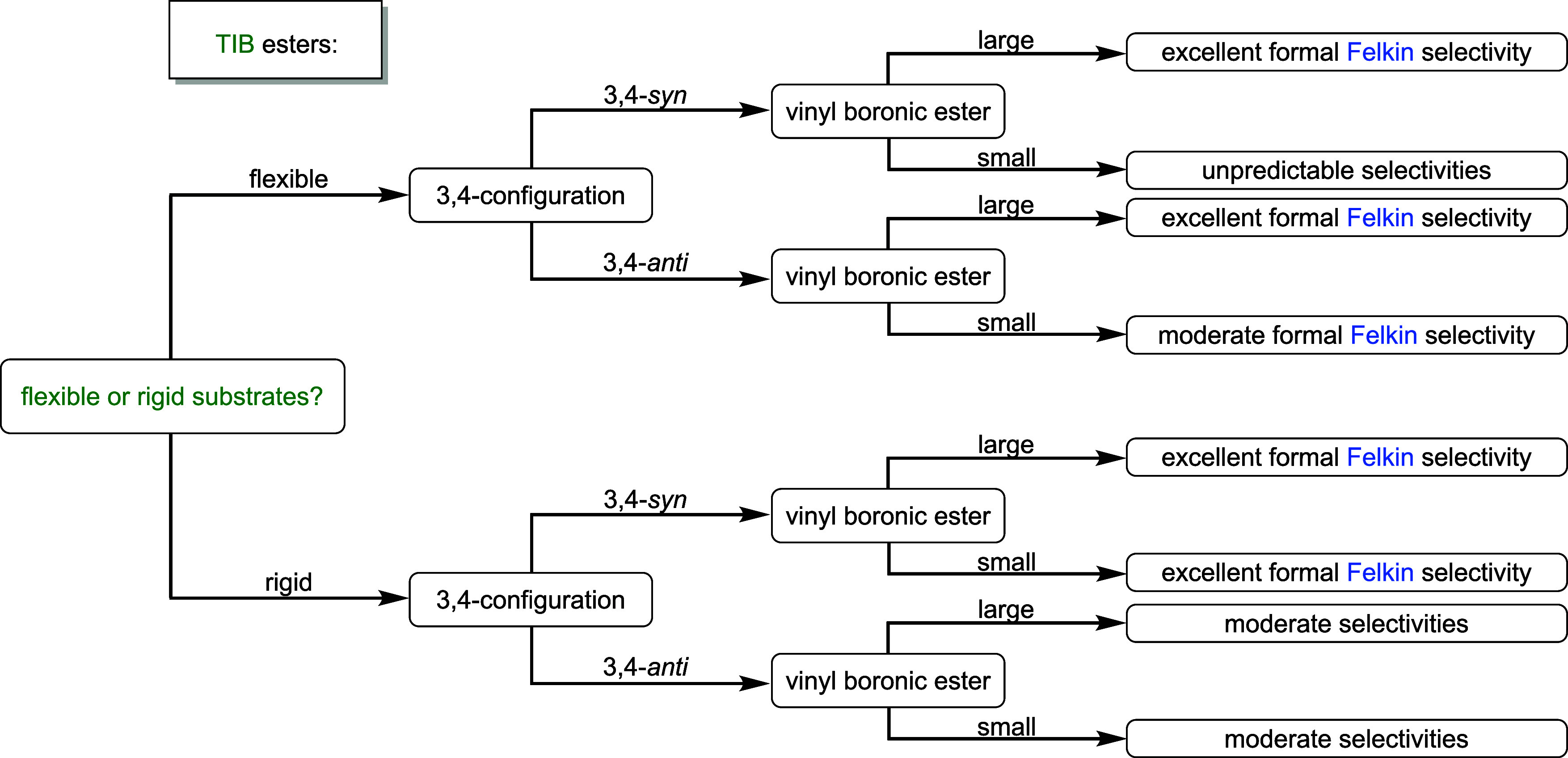
Guidance flowchart for the substrate- and reagent-controlled lithiation–borylation chemistry of TIB diketides bearing three contiguous stereocenters and VBEs.

In terms of Cb carbamates (Figure 6), a 3,4-*syn*-configuration in rigid substrates provided moderate selectivities for both large and small vinyl boronic esters and no general trend regarding the formation of the formal Felkin or formal *anti*-Felkin products. When an *anti*-relation was present, the formal *anti*-Felkin products were obtained in all cases. With large VBEs, moderate selectivities were obtained, whereas small reaction partners provided moderate to excellent selectivities. For more flexible substrates, a *syn*-configuration led to the formal *anti*-Felkin product in moderate selectivities with small VBEs. With larger reaction partners, no general preference toward one diastereoisomer was observed. In terms of an 3,4-*anti*-relation, the formal *anti*-Felkin products were formed in all reactions. For large VBEs, varying selectivities were obtained; however, with their small analogues, excellent diastereomeric ratios were obtained.

**Figure 6:**
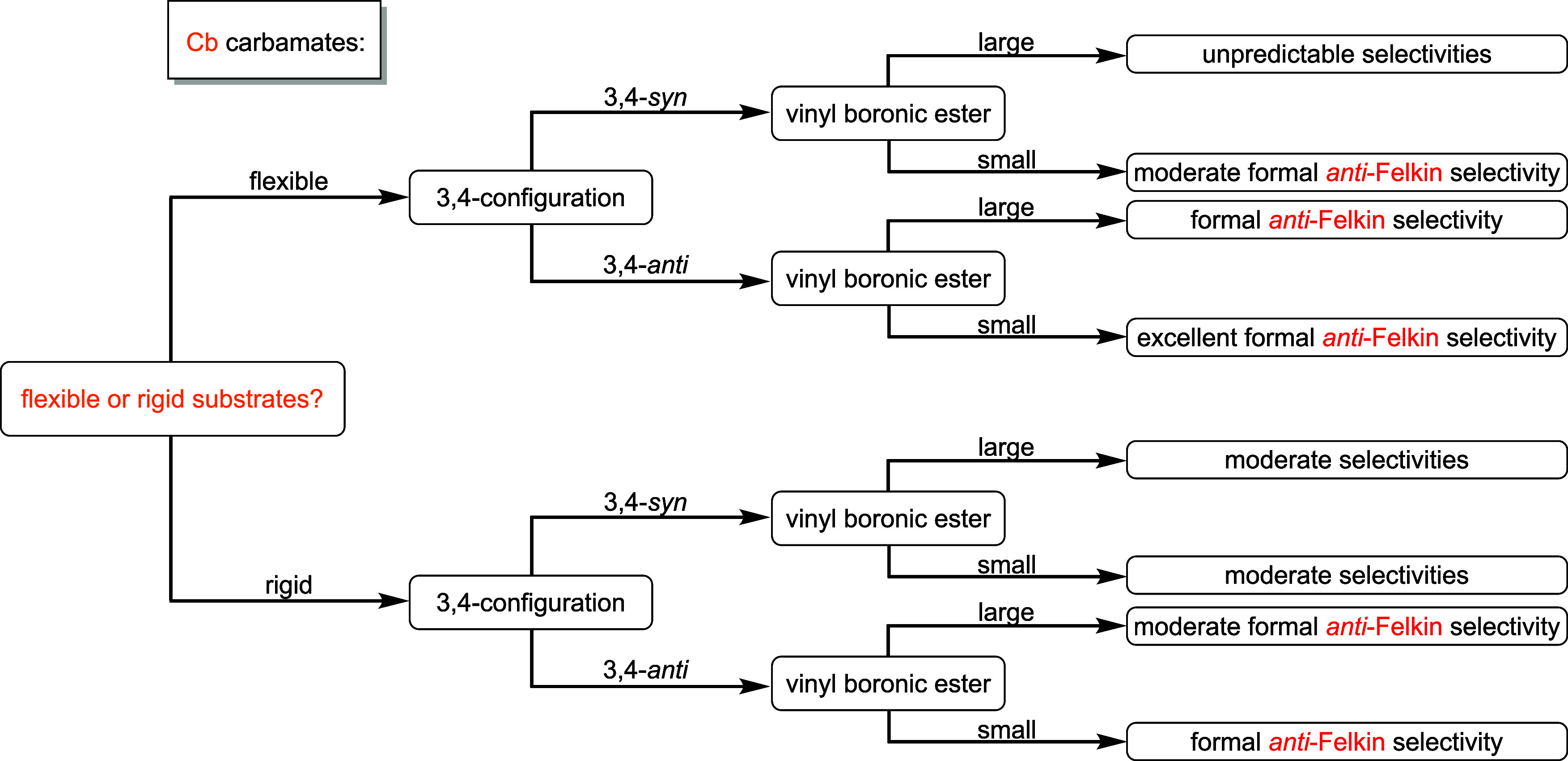
Guidance flowchart for the substrate- and reagent-controlled lithiation–borylation chemistry of Cb diketides bearing three contiguous stereocenters and VBEs.

## Conclusions

In conclusion, we have demonstrated the potential of substrate induction in the stereoselective synthesis of allylic alcohols through lithiation–borylation chemistry. We showed that most of the applied TIB esters led to the formal Felkin products in excellent selectivities. Access to the formal *anti*-Felkin products could be obtained for most of the conformational flexible substrates, when the TIB group was replaced by the Cb group. This shows the potential for stereodivergent applications of this methodology even though the formal *anti*-Felkin selectivities were usually lower. Due to the good yields and selectivities obtained for more complex substrates—which is also in line with our observations during the total synthesis of chondrochloren A (**8**)^6^—we believe that this methodology will be applied extensively in the syntheses of natural products or other complex molecules in the future, especially as a valuable alternative to the NHTK reaction.

## Experimental Section

### Substrate-Controlled 1,2-Metallate Rearrangement of Vinyl Boronates

#### General Procedure for the Conversion of TIB Esters

To a stirred solution of TIB ester (1.5 equiv) and diamine (1.5 equiv) in Et_2_O (0.2 M) at −78 °C was added *s*BuLi (1.3 M in hexanes, 1.4 equiv). The reaction mixture was stirred for 5 h at that temperature before a solution of vinyl boronic ester (1.0 equiv) in Et_2_O (0.5 M) was added. After stirring for further 3 h at −78 °C, the reaction mixture was warmed to 45 °C and stirred overnight. The reaction mixture was cooled to rt, sat. aq. NH_4_Cl was added, and the biphasic mixture was stirred for 15 min. The phases were separated, the organic layer was washed with sat. aq. NH_4_Cl (3×) and the combined aqueous phases were extracted with MTBE (3×). The combined organic phases were dried over Na_2_SO_4_ and concentrated in vacuo and the crude material was purified by a short flash column chromatography (to remove TIBOH).^7^ The residue was dissolved in THF (0.2 M) and cooled to −20 °C. A premixed, ice-cooled solution of NaOH (2.0 M)/H_2_O_2_ (35%, 2/1 v/v, 0.12 M) was added dropwise. The reaction mixture was stirred at rt before being diluted with MTBE and quenched by the slow addition of sat. aq. Na_2_S_2_O_3_ at 0 °C after TLC showed full conversion. The phases were separated, and the aqueous phase was extracted with MTBE (3×). The combined organic layers were dried over Na_2_SO_4_ and concentrated in vacuo. The crude product was purified by flash column chromatography to afford allylic alcohol.^7^

#### General Procedure for the Conversion of Carbamates

To a stirred solution of carbamate (1.5 equiv) and diamine (1.5 equiv) in Et_2_O (0.2 M) at −78 °C was added *s*BuLi (1.3 M in hexanes, 1.4 equiv). The reaction mixture was stirred for 5 h at that temperature before a solution of vinyl boronic ester (1.0 equiv) in Et_2_O (0.5 M) was added. The reaction mixture was stirred for 3 h at −78 °C.^7^ In parallel, magnesium turnings were activated (2× 1.0 M HCl, 2× H_2_O, 2× acetone, drying under high vacuum). The required amount (2.0 equiv) was dissolved in Et_2_O (0.8 M), and 1,2-dibromoethane (2.0 equiv) was added under water bath cooling. The reaction mixture was stirred for 2 h at this temperature.^7^ The biphasic MgBr_2_·OEt_2_ solution was added dropwise to the main reaction mixture, which was then stirred for another 30 min at −78 °C before being warmed to 45 °C and stirred overnight. The reaction mixture was cooled to rt, sat. aq. NH_4_Cl was added, and the biphasic mixture was stirred for 15 min. The phases were separated, the organic layer was washed with sat. aq. NH_4_Cl (3×), and the combined aqueous phases were extracted with MTBE (3×). The combined organic phases were dried over Na_2_SO_4_ and concentrated in vacuo, and the crude material was purified by a short flash column chromatography (to remove excess of the carbamate).^7^ The residue was dissolved in THF (0.2 M) and cooled to −20 °C. A premixed, ice-cooled solution of NaOH (2.0 M)/H_2_O_2_ (35%, 2/1 v/v, 0.12 M) was added dropwise. The reaction mixture was stirred at rt before being diluted with MTBE and quenched by the slow addition of sat. aq. Na_2_S_2_O_3_ at 0 °C after TLC showed full conversion. The phases were separated, and the aqueous phase was extracted with MTBE (3×). The combined organic layers were dried over Na_2_SO_4_ and concentrated in vacuo. The crude product was purified by flash column chromatography to afford allylic alcohol.^7^

## Supplementary Material



## Data Availability

The data underlying this study are available in the published article and its Supporting Information.

## Notes

Experimental procedures, NMR spectra, and compound characterizations (PDF)

## †

Bruno Bock GmbH, 21436 Marschacht, Germany

## Abbreviations

Ar: aryl
Bn: benzyl
Bu: butyl
Cb: *N*,*N*-diisopropyl carbamoyl
DG: directing group
Me: methyl
PG: protecting group
pin: pinacolato
PMB: *p*-methoxybenzyl
Pr: propyl
sp: sparteine
sps: sparteine surrogate
TBS: *tert*-butyldimethylsilyl
TMEDA: *N,N,N′,N′*-tetramethylethylenediamine
TIB: 2,4,6-triisopropylbenzoyl
TIP: 2,4,6-triisopropylphenyl
VBE: vinyl boronic ester
