# Supplementary material for: The Development of Stereoselective Substrate and Reagent-Controlled Lithiation–Borylation Chemistry
Source: J Org Chem. 2025 Oct 17;90(43):15256–70. doi: 10.1021/acs.joc.5c01854 (PMC12584137; doi:10.1021/acs.joc.5c01854)

Supporting Information

The Development of Stereoselective Substrate and Reagent Controlled Lithiation–Borylation Chemistry

Yannick Linne, Maike Birkner, Daniel Lücke, Jan Flormann, Kjeld Gerdes, Giada Tedesco, Gaia Stojanovic, Tom Jentsch, Birk Jäger, Kevin Bajerke, Jörg August Becker and Markus Kalesse*

Institute of Organic Chemistry, Gottfried Wilhelm Leibniz Universität Hannover, Schneiderberg 1B, 30167 Hannover (Germany)

Corresponding Author

*markus.kalesse@oci.uni-hannover.de

Table of Contents

1. General Considerations..S3

2. Experimental Procedures and Characterization Data............................S5

2-1. Synthesis of the 1,2-syn-motif..S5

2-2. Synthesis of the 1,2-anti-motif..S10

2-3. Synthesis of the acetonide syn-motifs..S15

2-4. Synthesis of the acetonide anti-motifs..S19

2-5. Synthesis of open-chained stereotriad syn-motifs..S23

2-6. Synthesis of open-chained stereotriad anti-motifs...S24

2-7. Synthesis of TIB esters...S26

2-8. Synthesis of carbamates...S35

2-9. Substrate-controlled 1,2-metallate rearrangement–solvent screening..............S44

2-10. Substrate-controlled 1,2-metallate rearrangement of vinyl boronates.............S46

2-11. Analysis of stereochemistry..*S81*

2-12. Rationalization of substrate control..*S106*

3. References...S125

4. Spectra...S127

1. General Considerations

Unless otherwise noted all reactions were carried out under an argon atmosphere using a Drierite™ gas-drying unit. The used glassware was flame dried under high vacuum. Air- and moisture-sensitive liquids and solutions were transferred via syringe flushed with argon prior to use. All reagents were purchased from commercial suppliers and used without further purification unless otherwise noted. Vinylboronic acid pinacol ester (**14**) was bought from Sigma Aldrich and Alfa Aesar and was distilled prior to use. Vinyl boronic esters **11–13** were synthesized according to literature procedures.[1] **(+)-Sparteine** was purchased from Chem-Impex and **(−)-sparteine** was bought from TCI. Both were distilled under high vacuum and stored under argon at −25 °C. Stated temperatures, except room temperature, refer to bath temperatures (heating was conducted using oil baths).

Dry solvents Dichloromethane and all amine bases were distilled under an inert atmosphere over calcium hydride. Tetrahydrofuran, diethyl ether and methanol were purchased from Acros Organics over molecular sieves and under inert atmosphere.

Thin layer chromatography All reactions were stirred magnetically and monitored using pre-coated TLC sheets ALUGRAM® Xtra SIL G/UV_{254} (0.2 mm, silica gel, F_{254}, aluminum-backed, MACHEREY-NAGEL) with detection by UV light (λ = 254 nm) and/or by staining with either basic potassium permanganate, acidic ceric ammonium molybdate, acidic anisaldehyde or acidic vanillin stain.

Flash column chromatography was performed using silica gel (0.04-0.063 mm, 240-400 mesh) obtained from MACHEREY-NAGEL. The applied petroleum ether fraction had a bp of 40-60 °C. The eluent is given in volume ratios (v/v).

^{1}H-NMR experiments were recorded in $CDCl_3$ or C_6D_6 using either a DPX 400 (Bruker), an AMX 400 (Bruker) or an Ascend 400 Avance III HD (Bruker). The spectra were calibrated using the residual solvent peak: $\delta(CDCl_3)$ = 7.26 ppm, $\delta(C_6D_6)$ = 7.16 ppm. Chemical shift δ is given in parts per million (ppm), coupling constant J in hertz (Hz) and multiplicity as follows: s, singlet; d, doublet; t, triplet; q, quadruplet; p, pentet; sex, sextet; sep, septet; m, multiplet; m_c, centered multiplet; brs, broad signal; or combination of these acronyms. NMR spectra were processed using TopSpin (Bruker).

^{13}C-NMR experiments were recorded in $CDCl_3$ or C_6D_6 using either a DPX 400 (Bruker), an AMX 400 (Bruker) or an Ascend 400 Avance III HD (Bruker). The spectra were calibrated using the residual solvent peak: $\delta(CDCl_3)$ = 77.16 ppm, $\delta(C_6D_6)$ = 128.06 ppm. Chemical shift δ is given in parts per million (ppm). NMR spectra were processed using TopSpin (Bruker).

High Resolution Mass Spectra (HRMS) were obtained either using a Q-Tof Premier (Waters), a LCT Premier (Waters) or a GC-system Agilent 6890 coupled with an Agilent 5973. Both the masses found and the masses calculated are given.

Optical rotation $[\alpha]_D^{20}$ were measured either on a P3000 polarimeter (A. Krüss Optronic, λ = 589 nm) or a Perkin-Elmer 341 (λ = 589 nm). The sample concentration (in g/100 mL) is given with every single experiment.

2. Experimental Procedures and Characterization Data

2-1. Synthesis of the 1,2-*syn*-motif

General Procedure 1a (GP1a): Evans-Aldol Reaction, TBS-Protection, Reduction

Note: Since the scale of the performed reactions differs due to need and availability, this general procedure reports the relative molar quantities, equivalents and relative solvent volumes of a representative experiment, whereby the exact volumes/weights of the structure-building (or key) chemicals are listed in the corresponding individual procedure.

A solution of the required propionated Evans auxiliary (12.8 mmol, 1.0 equiv) in CH_2Cl_2 (130 mL, 0.1 M) was cooled to −78 °C and treated with Et_3N (16.7 mmol, 1.3 equiv). Subsequently, *n*Bu_2BOTf (1.0 M in CH_2Cl_2, 14.7 mmol, 1.15 equiv) was added. The reaction mixture was stirred at −78 °C for 1 h, was then allowed to warm to 0 °C and stirred at this temperature for 1 h until it was re-cooled to −78 °C and the corresponding aldehyde (16.7 mmol, 1.3 equiv) was added. The reaction mixture was stirred at −78 °C for 2 h, until it was warmed to 0 °C and stirred for further 2 h. Following the successive addition of pH 7 buffer/MeOH (3/1) and MeOH/35% H_2O_2 (2/1) stirring was continued for 30 min at 0 °C. Afterwards, the organic layer was separated. The aqueous layer was extracted with CH_2Cl_2 (3x), the combined organic layers were washed with sat. aq. NaCl, dried over Na_2SO_4 and concentrated *in vacuo*.[2]
To a solution of the obtained *syn*-aldol product (12.8 mmol) in CH_2Cl_2 (13.0 mL, 1.0 M) at −78 °C were added 2,6-lutidine (66.8 mmol, 4.0 equiv) and TBSOTf (25.6 mmol, 2.0 equiv) successively. The reaction mixture was stirred for 20 min at −78 °C and at 0 °C until TLC showed full conversion. After the addition of sat. aq. NH_4Cl the organic layer was separated and the aqueous layer was extracted with CH_2Cl_2 (3x). The combined organic layers were washed with aq. $KHSO_4$ (1.0 M) and sat. aq. NaCl, dried over Na_2SO_4 and concentrated *in vacuo*. The crude material was purified by flash column chromatography to afford the TBS-protected *syn*-aldol product.
To a solution of the TBS-protected *syn*-aldol product (12.8 mmol) in THF (130 mL, 0.1 M) and MeOH (102 mmol, 8.0 equiv) at 0 °C was added $LiBH_4$ (4.0 M in THF, 102 mmol, 8.0 equiv) dropwise. The reaction mixture was stirred for 30 min at 0 °C and overnight at rt. The reaction mixture was then cooled to 0 °C and sat. aq. NaCl and MTBE were added. The organic layer was separated and the aqueous layer was extracted with MTBE (3x). The combined organic layers were dried over Na_2SO_4 and concentrated *in vacuo*. The crude product was purified by flash column chromatography to afford the corresponding primary alcohol.[3]

General Procedure 1b (GP1b): Evans-Aldol Reaction, PMB-Protection, Reduction

Note: Since the scale of the performed reactions differs due to need and availability, this general procedure reports the relative molar quantities, equivalents and relative solvent volumes of a representative experiment, whereby the exact volumes/weights of the structure-building (or key) chemicals are listed in the corresponding individual procedure.

A solution of the required propionated Evans auxiliary (6.43 mmol, 1.0 equiv) in CH_2Cl_2 (65 mL, 0.1 M) was cooled to −78 °C and treated with Et_3N (8.36 mmol, 1.3 equiv). Subsequently, *n*Bu_2BOTf (1.0 M in CH_2Cl_2, 7.39 mmol, 1.15 equiv) was added. The reaction mixture was stirred at −78 °C for 1 h, was then allowed to warm to 0 °C and stirred at this temperature for 1 h until it was re-cooled to −78 °C and isobutyraldehyde (8.36 mmol, 1.3 equiv) was added. The reaction mixture was stirred at −78 °C for 2 h, until it was warmed to 0 °C and stirred for further 2 h. Following the successive addition of pH 7 buffer/MeOH (3/1) and MeOH/35% H_2O_2 (2/1) stirring was continued for 30 min at 0 °C. Afterwards, the organic layer was separated. The aqueous layer was extracted with CH_2Cl_2 (3x), the combined organic layers were washed with sat. aq. NaCl, dried over Na_2SO_4 and concentrated *in vacuo*.[2]
To a solution of the obtained *syn*-aldol product (6.43 mmol) in CH_2Cl_2 (18.5 mL, 0.35 M) at rt were added PMB trichloroacetimidate (9.65 mmol, 1.5 equiv) and CSA (0.64 mmol, 0.1 equiv) successively. The reaction mixture was stirred overnight at rt. After the addition of sat. aq. $NaHCO_3$ the organic layer was separated, and the aqueous layer was extracted with MTBE (3x). The combined organic layers were washed with sat. aq. NaCl, dried over Na_2SO_4 and concentrated *in vacuo*. The crude material was purified by flash column chromatography to afford the PMB-protected *syn*-aldol product.[4]
To a solution of the PMB-protected *syn*-aldol product (6.43 mmol) in THF (65 mL, 0.1 M) and MeOH (51.4 mmol, 8.0 equiv) at 0 °C was added $LiBH_4$ (4.0 M in THF, 51.4 mmol, 8.0 equiv) dropwise. The reaction mixture was stirred for 30 min at 0 °C and overnight at rt. The reaction mixture was then cooled to 0 °C and sat. aq. NaCl and MTBE were added. The organic layer was separated and the aqueous layer was extracted with MTBE (3x). The combined organic layers were dried over Na_2SO_4 and concentrated *in vacuo*. The crude product was purified by flash column chromatography to afford the corresponding primary alcohol.[3]

General Procedure 1c (GP1c): Evans-Aldol Reaction, Bn-Protection, Reduction

Note: Since the scale of the performed reactions differs due to need and availability, this general procedure reports the relative molar quantities, equivalents and relative solvent volumes of a representative experiment, whereby the exact volumes/weights of the structure-building (or key) chemicals are listed in the corresponding individual procedure.

A solution of the required propionated Evans auxiliary (6.43 mmol, 1.0 equiv) in CH_2Cl_2 (65 mL, 0.1 M) was cooled to −78 °C and treated with Et_3N (8.36 mmol, 1.3 equiv). Subsequently, *n*Bu_2BOTf (1.0 M in CH_2Cl_2, 7.39 mmol, 1.15 equiv) was added. The reaction mixture was stirred at −78 °C for 1 h, was then allowed to warm to 0 °C and stirred at this temperature for 1 h until it was re-cooled to −78 °C and isobutyraldehyde (8.36 mmol, 1.3 equiv) was added. The reaction mixture was stirred at −78 °C for 2 h, until it was warmed to 0 °C and stirred for further 2 h. Following the successive addition of pH 7 buffer/MeOH (3/1) and MeOH/35% H_2O_2 (2/1) stirring was continued for 30 min at 0 °C. Afterwards, the organic layer was separated. The aqueous layer was extracted with CH_2Cl_2 (3x), the combined organic layers were washed with sat. aq. NaCl, dried over Na_2SO_4 and concentrated *in vacuo*.[3]

To a solution of the obtained *syn*-aldol product (8.36 mmol) in a mixture of cyclohexane and CH_2Cl_2 (2/1, v/v, 17.0 mL, 0.50 M) at rt were added benzyl trichloroacetimidate (12.5 mmol, 1.5 equiv) and TfOH (1.25 mmol, 0.15 equiv) successively. The reaction mixture was stirred overnight at rt. After the addition of sat. aq. $NaHCO_3$ the organic layer was separated, and the aqueous layer was extracted with CH_2Cl_2 (4x). The combined organic layers were dried over Na_2SO_4 and concentrated *in vacuo*. The crude material was purified by flash column chromatography to afford the Bn-protected *syn*-aldol product.[5]

To a solution of the Bn-protected *syn*-aldol product (8.36 mmol) in THF (8.5 mL, 0.1 M) and MeOH (66.9 mmol, 8.0 equiv) at 0 °C was added $LiBH_4$ (4.0 M in THF, 66.9 mmol, 8.0 equiv) dropwise. The reaction mixture was stirred for 30 min at 0 °C and overnight at rt. The reaction mixture was then cooled to 0 °C and sat. aq. NaCl and MTBE were added. The organic layer was separated, and the aqueous layer was extracted with MTBE (3x). The combined organic layers were dried over Na_2SO_4 and concentrated *in vacuo*. The crude product was purified by flash column chromatography to afford the corresponding primary alcohol.[3]

TBS-protected 1,2-*syn* alcohol S1

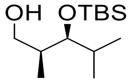

S1

$C_{13}H_{30}O_2Si$

246.47

Using GP1a, (−)-4-benzyl-3-propionyl-2-oxazolidinone (3.00 g, 12.8 mmol, 1.0 equiv) and freshly distilled isobutyraldehyde (1.5 mL, 16.7 mmol, 1.3 equiv) gave primary alcohol **S1** (1.30 g, 5.27 mmol, 41% o3s, dr ≥ 19:1) after purification by flash column chromatography (PE:MTBE 7:1) as a colorless oil.

^{1}H NMR (400 MHz, $CDCl_3$): δ = 3.62 (dd, J = 10.4, 8.1 Hz, 1H), 3.52 (dd, J = 5.7, 2.7 Hz, 1H), 3.47 (dd, J = 10.1, 5.9 Hz, 1H), 1.92 (m_c, 1H), 1.81 (m_c, 2H), 0.94-0.88 (m, 15H), 0.86 (d, J = 7.0 Hz, 3H), 0.08 (s, 3H), 0.06 (s, 3H) ppm;

^{13}C{^{1}H} NMR (101 MHz, $CDCl_3$): δ = 78.5, 66.6, 39.4, 31.6, 26.2, 20.5, 19.3, 18.5, 12.2, −3.8, −4.0 ppm;

HRMS (ESI): $C_{13}H_{30}O_2SiNa$ $[M+Na]^+$ calculated: 269.1913, found: 269.1920;

R*f* = 0.3 (PE:MTBE 4:1, vanillin);

$[\alpha]_D^{20}$ = +5.7 (*c* 0.9, $CHCl_3$).

Analytical data are in accordance with the literature.[6]

PMB-protected 1,2-*syn* alcohol S2

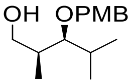

S2

$C_{15}H_{24}O_3$

252.35

Using GP1b, (−)-4-benzyl-3-propionyl-2-oxazolidinone (1.50 g, 6.43 mmol, 1.0 equiv) and freshly distilled isobutyraldehyde (0.76 mL, 8.36 mmol, 1.3 equiv) gave PMB-protected primary alcohol **S2** (0.53 g, 2.10 mmol, 33% o3s, dr ≥ 19:1) after purification by flash column chromatography (PE:MTBE 4:1) as a colorless oil.

^{1}H NMR (400 MHz, C_6D_6): δ = 7.29-7.27 (m, 2H), 6.81-6.79 (m, 2H), 4.49 (s, 2H), 3.52-3.47 (m, 1H), 3.36 (dd, J = 10.2, 5.5 Hz, 1H), 3.31 (s, 3H), 3.17 (dd, J = 7.7, 3.2 Hz, 1H), 1.90-1.83 (m, 1H), 1.82-1.74 (m, 1H), 1.30 (brs, 1H), 1.04 (d, J = 6.7 Hz, 3H), 0.92 (d, J = 7.0 Hz, 3H), 0.83 (d, J = 6.8 Hz, 3H) ppm;

^{13}C{^{1}H} NMR (101 MHz, C_6D_6): δ = 159.6, 131.9, 129.4, 114.1, 85.2, 74.7, 66.2, 54.8, 38.2, 31.3, 20.0, 19.7, 11.2 ppm;

HRMS (ESI): $C_{15}H_{24}O_3Na$ $[M+Na]^+$ calculated: 275.1623, found: 275.1621;

R*f* = 0.2 (PE:MTBE 4:1, uv, CAN);

$[\alpha]_D^{20}$ = +11.1 (c 1.1, $CHCl_3$).

Analytical data are in accordance with the literature.[4]

Bn-protected 1,2-*syn* alcohol S3

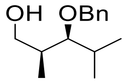

S3

$C_{14}H_{22}O_2$

222.33

Using GP1c, (−)-4-benzyl-3-propionyl-2-oxazolidinone (1.50 g, 6.43 mmol, 1.0 equiv) and freshly distilled isobutyraldehyde (0.76 mL, 8.36 mmol, 1.3 equiv) gave Bn-protected primary alcohol **S3** (0.36 g, 1.62 mmol, 25% o3s, dr ≥ 19:1) after purification by flash column chromatography (PE:MTBE 4:1) as a colorless oil.

^{1}H NMR (400 MHz, $CDCl_3$): δ = 7.38-7.32 (m, 4H), 7.30-7.27 (m, 1H), 4.62 (dd, J = 26.7, 11.3 Hz, 2H), 3.66-3.56 (m, 2H), 3.26 (dd, J = 7.7, 3.2 Hz, 1H), 2.02-1.90 (m, 2H), 1.69 (brs, 1H), 1.05 (d, J = 6.7 Hz, 3H), 0.95-0.92 (m, 6H) ppm;

^{13}C{^{1}H} NMR (101 MHz, $CDCl_3$): δ = 139.1, 128.5, 127.8, 127.7, 85.9, 74.8, 66.6, 37.8, 31.0, 20.2, 19.6, 11.1 ppm;

HRMS (ESI): $C_{14}H_{22}O_2Na$ $[M+Na]^+$ calculated: 245.1517, found: 245.1513;

R$_f$ = 0.3 (PE:MTBE 4:1, uv, CAN);

$[\alpha]_D^{20}$ = +10.7 (c 1.0, $CHCl_3$).

Analytical data are in accordance with the literature.[7]

2-2. Synthesis of the 1,2-*anti*-motif

General Procedure 2a (GP2a): Oppolzer-Aldol Reaction, TBS-Protection, Reduction

Note: Since the scale of the performed reactions differs due to need and availability, this general procedure reports the relative molar quantities, equivalents and relative solvent volumes of a representative experiment, whereby the exact volumes/weights of the structure-building (or key) chemicals are listed in the corresponding individual procedure.

The required propionated Oppolzer auxiliary (5.53 mmol, 1.0 equiv) was dissolved in CH_2Cl_2 (5.5 mL, 1.0 M) and Et_3N (8.29 mmol, 1.5 equiv) and TMSOTf (9.40 mmol, 1.7 equiv) were added successively over a period of 15 min. The reaction mixture was then stirred overnight at rt. A solution of the corresponding aldehyde (8.29 mmol, 1.5 equiv) in CH_2Cl_2 (10.5 mL, 0.8 M refers to aldehyde) at −78 °C was treated with $TiCl_4$ (1.0 M in CH_2Cl_2, 8.29 mmol, 1.5 equiv) over a period of 15 min. The solution containing the (*Z*)-ketene acetal was cooled to −78 °C and then transferred to the cooled aldehyde containing solution via cannula. After complete addition, the reaction mixture was stirred at −78 °C until TLC showed full conversion before being quenched by the addition of sat. aq. NH_4Cl. After warming to 0 °C and stirring for further 30 min at this temperature the organic layer was separated. The aqueous layer was extracted with CH_2Cl_2 (3x), the combined organic layers were dried over Na_2SO_4 and the solvent was removed *in vacuo*.
To a solution of the obtained *anti*-aldol product (5.53 mmol) in CH_2Cl_2 (5.5 mL, 1.0 M) at −78 °C were added 2,6-lutidine (22.1 mmol, 4.0 equiv) and TBSOTf (11.1 mmol, 2.0 equiv) successively. The reaction mixture was stirred for 20 min at −78 °C and at 0 °C until TLC showed full conversion. After the addition of sat. aq. NH_4Cl the organic layer was separated and the aqueous layer was extracted with CH_2Cl_2 (3x). The combined organic layers were washed with aq. $KHSO_4$ (1.0 M) and sat. aq. NaCl, dried over Na_2SO_4 and concentrated *in vacuo*. The crude material was purified by flash column chromatography to afford the TBS-protected *anti*-aldol product.
To a solution of the TBS-protected *anti*-aldol product (5.53 mmol) in THF (55 mL, 0.1 M) and MeOH (44.2 mmol, 8.0 equiv) at 0 °C was added $LiBH_4$ (4.0 M in THF, 44.2 mmol, 8.0 equiv) dropwise. The reaction mixture was stirred for 30 min at 0 °C and overnight at rt. The reaction mixture was then cooled to 0 °C and sat. aq. NaCl and MTBE were added. The organic layer was separated and the aqueous layer was extracted with MTBE (3x). The combined organic layers were dried over Na_2SO_4 and concentrated *in vacuo*. The crude product was purified by flash column chromatography to afford the corresponding primary alcohol.[3]

General Procedure 2b (GP2b): Oppolzer-Aldol Reaction, PMB-Protection, Reduction

Note: Since the scale of the performed reactions differs due to need and availability, this general procedure reports the relative molar quantities, equivalents and relative solvent volumes of a representative experiment, whereby the exact volumes/weights of the structure-building (or key) chemicals are listed in the corresponding individual procedure.

(–)-*N*-Propionyl sultam (11.0 mmol, 1.0 equiv) was dissolved in CH_2Cl_2 (11.0 mL, 1.0 M) and Et_3N (16.5 mmol, 1.5 equiv) and TMSOTf (19.8 mmol, 1.8 equiv) were added successively over a period of 15 min. The yellow mixture was stirred overnight at rt. A solution of isobutyraldehyde (16.5 mmol, 1.5 equiv) in CH_2Cl_2 (20.5 mL, 0.8 M refers to aldehyde) was prepared at −78 °C and $TiCl_4$ (1.0 M in CH_2Cl_2, 16.5 mmol, 1.5 equiv) was added over a period of 15 min. The solution containing the (*Z*)-ketene acetal was cooled to the same temperature and transferred into the cooled aldehyde solution via cannula. The dark red reaction mixture was stirred at −78 °C for 5 h. Upon completion, the reaction mixture was quenched by the addition of sat. aq. NH_4Cl and was stirred at 0 °C for 2 h. The layers were separated and the aqueous phase extracted with CH_2Cl_2 (3x). The combined organic layers were dried over Na_2SO_4, filtered and concentrated *in vacuo*. The crude material was purified by flash column chromatography to afford the corresponding *anti*-alcohol.

To a stirred solution of the obtained *anti*-alcohol (11.0 mmol) in CH_2Cl_2 (31 mL, 0.35 M) at rt were added PMB trichloroacetimidate (16.5 mmol, 1.5 equiv) and CSA (1.10 mmol, 0.1 equiv) successively. The reaction mixture was stirred overnight at the same temperature, before sat. aq. $NaHCO_3$ was added. The aqueous phase was extracted with MTBE (3x) and the combined organic layers were washed with sat. aq. NaCl, then dried over Na_2SO_4, filtered and concentrated *in vacuo*. The crude product was purified by flash column chromatography affording the corresponding *anti*-PMB-ether.

To a solution of the *anti*-PMB-ether (11.0 mmol) in THF (110 mL, 0.1 M) and MeOH (88.0 mmol, 8.0 equiv) at 0 °C was added $LiBH_4$ (4.0 M in THF, 88.0 mmol, 8.0 equiv) dropwise. The reaction mixture was stirred for 1 h at 0 °C and overnight at rt. The reaction mixture was then cooled to 0 °C and sat. aq. NaCl and MTBE were added. The organic layer was separated and the aqueous layer was extracted with MTBE (3x). The combined organic layers were dried over Na_2SO_4 and concentrated *in vacuo*. The crude product was purified by flash column chromatography to afford the corresponding primary alcohol.

General Procedure 2c (GP2c): Oppolzer-Aldol Reaction, Bn-Protection, Reduction

Note: Since the scale of the performed reactions differs due to need and availability, this general procedure reports the relative molar quantities, equivalents and relative solvent volumes of a representative experiment, whereby the exact volumes/weights of the structure-building (or key) chemicals are listed in the corresponding individual procedure.

(–)-*N*-Propionyl sultam (11.0 mmol, 1.0 equiv) was dissolved in CH_2Cl_2 (11.0 mL, 1.0 M) and Et_3N (16.5 mmol, 1.5 equiv) and TMSOTf (19.8 mmol, 1.8 equiv) were added successively over a period of 15 min. The yellow mixture was stirred overnight at rt. A solution of isobutyraldehyde (16.5 mmol, 1.5 equiv) in CH_2Cl_2 (20.5 mL, 0.8 M refers to aldehyde) was prepared at −78 °C and $TiCl_4$ (1.0 M in CH_2Cl_2, 16.5 mmol, 1.5 equiv) was added over a period of 15 min. The solution containing the (*Z*)-ketene acetal was cooled to the same temperature and transferred into the cooled aldehyde solution via cannula. The dark red reaction mixture was stirred at −78 °C for 5 h. Upon completion, the reaction mixture was quenched by the addition of sat. aq. NH_4Cl and was stirred at 0 °C for 2 h. The layers were separated and the aqueous phase extracted with CH_2Cl_2 (3x). The combined organic layers were dried over Na_2SO_4, filtered and concentrated *in vacuo*. The crude material was purified by flash column chromatography to afford the corresponding *anti*-alcohol.
To a solution of the obtained *anti*-aldol product (11.0 mmol) in a mixture of cyclohexane and CH_2Cl_2 (2/1, v/v, 22.0 mL, 0.50 M) at rt were added benzyl trichloroacetimidate (13.2 mmol, 1.2 equiv) and TfOH (1.65 mmol, 0.15 equiv) successively. The reaction mixture was stirred until TLC showed full conversion. After the addition of sat. aq. $NaHCO_3$ the organic layer was separated, and the aqueous layer was extracted with CH_2Cl_2 (4x). The combined organic layers were dried over Na_2SO_4 and concentrated *in vacuo*. The crude material was purified by flash column chromatography to afford the Bn-protected *anti*-aldol product.
To a solution of the Bn-protected *anti*-aldol product (11.0 mmol) in THF (110 mL, 0.1 M) and MeOH (88.0 mmol, 8.0 equiv) at 0 °C was added $LiBH_4$ (4.0 M in THF, 88.0 mmol, 8.0 equiv) dropwise. The reaction mixture was stirred for 1 h at 0 °C and overnight at rt. The reaction mixture was then cooled to 0 °C and sat. aq. NaCl and MTBE were added. The organic layer was separated, and the aqueous layer was extracted with MTBE (3x). The combined organic layers were dried over Na_2SO_4 and concentrated *in vacuo*. The crude product was purified by flash column chromatography to afford the corresponding primary alcohol.

TBS-protected 1,2-*anti* alcohol S4

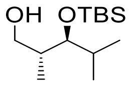

S4
$C_{13}H_{30}O_2Si$
246.47

Using GP2a, (−)-*N*-propionyl sultam (1.50 g, 5.53 mmol, 1.0 equiv) and freshly distilled isobutyraldehyde (0.76 mL, 8.29 mmol, 1.5 equiv) gave primary alcohol **S4** (0.91 g, 3.69 mmol, 67% o3s, dr ≥ 19:1) after purification by flash column chromatography (PE:MTBE 4:1) as a colorless oil.

^{1}H NMR (400 MHz, $CDCl_3$): δ = 3.67 (dd, J = 11.0, 4.2 Hz, 1H), 3.58 (dd, J = 11.0, 6.0 Hz, 1H), 3.43 (t, J = 4.9 Hz, 1H), 1.91-1.76 (m, 2H), 0.98 (d, J = 6.8 Hz, 3H), 0.95-0.88 (m, 15H), 0.11 (s, 3H), 0.08 (s, 3H) ppm;

^{13}C{^{1}H} NMR (101 MHz, $CDCl_3$): δ = 82.6, 66.2, 37.0, 33.3, 26.2, 19.1, 18.6, 18.4, 16.7, −3.8, −3.9 ppm;

HRMS (ESI): $C_{13}H_{30}O_2SiNa$ $[M+Na]^+$ calculated: 269.1913, found: 269.1907;

R*f* = 0.3 (PE:MTBE 4:1, $KMnO_4$);

$[\alpha]_D^{20}$ = +3.5 (*c* 0.6, $CHCl_3$).

Analytical data are in accordance with the literature.[8]

PMB-protected 1,2-*anti* alcohol S5

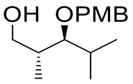

S5
$C_{15}H_{24}O_3$
252.35

Using GP2b, (–)-*N*-propionyl sultam (2.98 g, 11.0 mmol, 1.0 equiv) and freshly distilled isobutyraldehyde (1.5 mL, 16.5 mmol, 1.5 equiv) gave primary alcohol **S5** (0.26 g, 1.05 mmol, 55% o3s).[1]

^{1}H NMR (400 MHz, $CDCl_3$): δ = 7.27 (d, J = 8.7 Hz, 2H), 6.87 (d, J = 8.7 Hz, 2H), 4.59 (d, J = 10.7 Hz, 1H), 4.51 (d, J = 10.7 Hz, 1H), 3.80 (s, 3H), 3.71 (dd, J = 10.9, 3.5 Hz, 1H), 3.58 (dd, J = 10.9, 5.7 Hz, 1H), 3.17-3.09 (m, 2H), 1.97-1.87 (m, 2H), 1.01-0.98 (m, 9H) ppm;

^{13}C{^{1}H} NMR (101 MHz, $CDCl_3$): δ = 159.4, 130.7, 129.6, 114.0, 90.2, 75.3, 66.3, 55.4, 37.2, 31.3, 20.4, 17.8, 15.8 ppm;

HRMS (ESI): $C_{15}H_{24}O_3Na$ $[M+Na]^+$ calculated: 275.1623, found: 275.1619;

R*f* = 0.2 (PE:MTBE 6:1, vanillin);

[1] One fifth was taken forward after the aldol reaction.

$[\alpha]_D^{20}$= +16.1 (*c* 0.7, $CHCl_3$).

Analytical data are in accordance with the literature.[9]

Bn-protected 1,2-*anti* alcohol S6

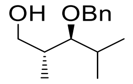

S6

$C_{14}H_{22}O_2$

222.33

Using GP2c, (–)-*N*-propionyl sultam (2.98 g, 11.0 mmol, 1.0 equiv) and freshly distilled isobutyraldehyde (1.5 mL, 16.5 mmol, 1.5 equiv) gave primary alcohol **S6** (0.38 g, 1.72 mmol, 79% o3s).[2]

^{1}H NMR (400 MHz, $CDCl_3$): δ = 7.35-7.29 (m, 5H), 4.66 (d, J = 11.0 Hz, 1H), 4.59 (d, J = 11.0 Hz, 1H), 3.73 (dd, J = 10.8, 3.5 Hz, 1H), 3.61 (dd, J = 10.8, 5.7 Hz, 1H), 3.17 (dd, J = 6.2, 6.1 Hz, 1H), 2.01-1.87 (m, 2H), 1.04-0.97 (m, 9H) ppm;

^{13}C{^{1}H} NMR (101 MHz, $CDCl_3$): δ = 138.5, 128.6, 127.91, 127.86, 90.4, 75.7, 66.2, 37.3, 31.3, 20.3, 17.8, 15.8 ppm;

HRMS (ESI): $C_{14}H_{22}O_2Na$ $[M+Na]^+$ calculated: 245.1517, found: 245.1515;

R*f* = 0.2 (PE:MTBE 6:1, vanillin);

$[\alpha]_D^{20}$ = +14.0 (*c* 0.5, $CHCl_3$).

[2] Again, one fifth was taken forward after the aldol reaction.

2-3. Synthesis of the acetonide *syn*-motifs

General Procedure 3 (GP3): Evans-Aldol Reaction, Hydrogenation, Acetonide Formation, Reduction

Note: Since the scale of the performed reactions differs due to need and availability, this general procedure reports the relative molar quantities, equivalents and relative solvent volumes of a representative experiment, whereby the exact volumes/weights of the structure-building (or key) chemicals are listed in the corresponding individual procedure.

A solution of the required propionated Evans auxiliary (16.5 mmol, 1.0 equiv) in CH_2Cl_2 (83 mL, 0.2 M) was cooled to 0 °C and treated with Et_3N (19.8 mmol, 1.2 equiv) and *n*Bu_2BOTf (1.0 M in CH_2Cl_2, 18.2 mmol, 1.1 equiv). The reaction mixture was stirred at 0 °C for 1 h until it was cooled to –78 °C and the corresponding aldehyde (16.5 mmol, 1.0 equiv) was added. The reaction mixture was stirred at –78 °C for 1.5 h, until it was warmed to 0 °C and stirred for further 2 h. Following the successive addition of pH 7 buffer/MeOH (3/1) and MeOH/35% H_2O_2 (2/1) stirring was continued for 45 min at 0 °C. Afterwards, the organic layer was separated. The aqueous layer was extracted with CH_2Cl_2 (3x), the combined organic layers were washed with sat. aq. $NaHCO_3$ and sat. aq. NaCl, dried over Na_2SO_4 and concentrated *in vacuo*. The crude material was purified by flash column chromatography to afford the corresponding *syn*-aldol product.[10]

Through a suspension of the obtained *syn*-aldol product (16.5 mmol) and Pd/C (10 wt%, 1.65 mmol, 0.1 equiv) in EtOAc (165 mL, 0.1 M) were bubbled three balloons of H_2. The reaction mixture was stirred at rt under an atmosphere of H_2 until TLC showed full conversion. The reaction mixture was filtered through a short plug of Celite® (EtOAc) and concentrated *in vacuo*.

To a solution of the obtained diol (16.5 mmol) in 2,2-DMP/acetone (2:1, 40 mL, 0.4 M) was added PPTS (1.65 mmol, 0.1 equiv). The reaction mixture was stirred at rt until TLC showed full conversion. After the addition of sat. aq. $NaHCO_3$ and MTBE the organic layer was separated, and the aqueous layer was extracted with MTBE (3x). The combined organic layers were washed with sat. aq. NaCl, dried over Na_2SO_4 and concentrated *in vacuo*. The crude material was purified by flash column chromatography to afford the corresponding acetonide.

To a solution of the obtained acetonide (16.5 mmol) in THF (165 mL, 0.1 M) and MeOH (132 mmol, 8.0 equiv) at 0 °C was added $LiBH_4$ (4.0 M in THF, 132 mmol, 8.0 equiv) dropwise. The reaction mixture was stirred for 30 min at 0 °C and overnight at rt. The reaction mixture was then cooled to 0 °C and sat. aq. NaCl and MTBE were added. The organic layer was separated, and the aqueous layer was extracted with MTBE (3x). The combined organic layers were dried over Na_2SO_4 and concentrated *in vacuo*. The crude product was purified by flash column chromatography to afford the corresponding primary alcohol.[3]

General Procedure 4 (GP4): Evans-Aldol Reaction, Reduction, Acetonide Formation, cleavage of TBDPS-ether

Note: Since the scale of the performed reactions differs due to need and availability, this general procedure reports the relative molar quantities, equivalents and relative solvent volumes of a representative experiment, whereby the exact volumes/weights of the structure-building (or key) chemicals are listed in the corresponding individual procedure.

A solution of the required propionated Evans auxiliary (6.70 mmol, 1.1 equiv) in CH_2Cl_2 (34 mL, 0.2 M refers to propionated Evans auxiliary) was cooled to –78 °C and treated with Et_3N (8.22 mmol, 1.35 equiv). Subsequently, $n$$Bu_2BOTf$ (1.0 M in CH_2Cl_2, 6.70 mmol, 1.1 equiv) was added. The reaction mixture was allowed to warm to 0 °C and stirred at this temperature for 1 h until it was re-cooled to –78 °C and the corresponding aldehyde (6.09 mmol, 1.0 equiv) was added. The reaction mixture was stirred at –78 °C for 1 h, until it was warmed to 0 °C and stirred for further 2 h. Following the successive addition of pH 7 buffer/MeOH (3/1) and MeOH/35% H_2O_2 (2/1) stirring was continued for 1 h at 0 °C. Afterwards, the organic layer was separated. The aqueous layer was extracted with CH_2Cl_2 (3x), the combined organic layers were washed with sat. aq. NaCl, dried over $MgSO_4$ and concentrated *in vacuo*. The crude material was purified by flash column chromatography to afford the *syn*-aldol product.[2]

To a solution of the obtained *syn*-aldol product (6.09 mmol) in THF (30 mL, 0.2 M) and MeOH (18.3 mmol, 3.0 equiv) at 0 °C was added $LiBH_4$ (4.0 M in THF, 18.3 mmol, 3.0 equiv) dropwise. The reaction mixture was stirred for 2 h at 0 °C, before a solution of NaOH (1.0 M) and EtOAc were added. The organic layer was separated, and the aqueous layer was extracted with EtOAc (3x). The combined organic layers were dried over $MgSO_4$ and concentrated *in vacuo*. The crude product was purified by flash column chromatography to afford the corresponding diol.[11]

To a solution of the obtained diol (6.09 mmol) and 2,2-DMP (122 mmol, 20.0 equiv) in acetone (15.0 mL, 0.4 M) was added PPTS (0.30 mmol, 0.05 equiv). The reaction mixture was stirred at rt until TLC showed full conversion. After the addition of sat. aq. $NaHCO_3$ and MTBE the organic layer was separated, and the aqueous layer was extracted with MTBE (3x). The combined organic layers were washed with sat. aq. NaCl, dried over $MgSO_4$ and concentrated *in vacuo*. The crude material was purified by flash column chromatography to afford the corresponding acetonide.

To a solution of the obtained acetonide (6.09 mmol) in THF (30 mL, 0.2 M) was added TBAF (1.0 M in THF, 18.3 mmol, 3.0 equiv). The reaction mixture was stirred overnight at rt. After the addition of H_2O and MTBE the organic layer was separated, and the aqueous layer was extracted with MTBE (3x). The combined organic layers were washed with sat. aq. NaCl, dried over $MgSO_4$ and concentrated *in vacuo*. The crude material was purified by flash column chromatography to afford the corresponding primary alcohol.

Acetonide-protected 1,2-*syn* alcohol S7

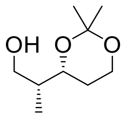

S7
$C_9H_{18}O_3$
174.24

Using GP3, (+)-4-benzyl-3-propionyl-2-oxazolidinone (3.85 g, 16.5 mmol, 1.0 equiv) and literature known 3-(benzyloxy)propanal (2.71 g, 16.5 mmol, 1.0 equiv)[12] gave primary alcohol **S7** (1.08 g, 6.20 mmol, 38% o4s, dr 19:1) after purification by flash column chromatography (PE:MTBE 1:1) as a colorless oil.

^{1}H NMR (400 MHz, C_6D_6): δ = 3.74 (m_c, 1H), 3.65-3.54 (m, 3H), 3.43 (m_c, 1H), 2.01 (brs, 1H), 1.65-1.52 (m, 2H), 1.41 (d, J = 0.6 Hz, 3H), 1.24 (d, J = 0.5 Hz, 3H), 0.84 (d, J = 7.1 Hz, 3H), 0.80 (m_c, 1H) ppm;

^{13}C{^{1}H} NMR (101 MHz, C_6D_6): δ = 98.4, 71.7, 65.3, 59.9, 40.0, 30.3, 27.6, 19.2, 11.8 ppm;

HRMS (EI): $C_8H_{15}O_3$ $[M–CH_3]^+$ calculated: 159.1021, found: 159.1022;

R*f* = 0.3 (PE:MTBE 1:1, vanillin);

$[\alpha]_D^{20}$ = –9.73 (*c* 1.6, $CHCl_3$).

Analytical data are in accordance with the literature.[13]

Acetonide-protected all-*syn* alcohol S8

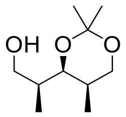

S8
$C_{10}H_{20}O_3$
188.27

Using GP4, (+)-4-benzyl-3-propionyl-2-oxazolidinone (1.56 g, 6.70 mmol, 1.1 equiv) and literature known (*S*)-3-((*tert*-butyldiphenylsilyl)oxy)-2-methylpropanal (1.99 g, 6.09 mmol, 1.0 equiv)[14] gave primary alcohol **S8** (0.79 g, 4.20 mmol, 69% o4s, dr ≥ 19:1) after purification by flash column chromatography (PE:MTBE 4:1 → 2:1 → 1:1) as a colorless oil.

^{1}H NMR (400 MHz, C_6D_6): δ = 3.80 (dd, J = 11.5, 2.7 Hz, 1H), 3.61 (dd, J = 9.6, 2.4 Hz, 1H), 3.43 (dd, J = 11.5, 1.7 Hz, 1H), 3.22-3.14 (m, 2H), 1.68-1.59 (m, 1H), 1.49 (s, 3H), 1.28 (s, 3H), 1.28-1.21 (m, 1H), 1.08 (d, J = 7.0 Hz, 3H), 1.06 (d, J = 6.6 Hz, 3H), 0.57 (brs, 1H) ppm;

^{13}C{^{1}H} NMR (101 MHz, C_6D_6): δ = 98.8, 73.9, 67.0, 64.0, 37.4, 30.7, 30.3, 19.1, 14.0, 11.3 ppm;

HRMS (ESI): $C_9H_{17}O_3$ $[M–Me]^+$ calculated: 173.1178, found: 173.1178;

R*f* = 0.4 (PE:MTBE 1:1, vanillin);

$[\alpha]_D^{25}$ = +8.0 (c 1.0, $CHCl_3$).

Acetonide-protected *syn, anti* alcohol S9

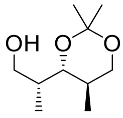

S9
$C_{10}H_{20}O_3$
188.27

Using a modified version of GP3, (+)-4-benzyl-3-propionyl-2-oxazolidinone (2.51 g, 10.8 mmol, 1.1 equiv) and literature known (*R*)-3-(4-methoxybenzyloxy)-2-methylpropanal (2.04 g, 9.80 mmol, 1.0 equiv)[15] gave primary alcohol **S3** (0.68 g, 3.61 mmol, 75% o4s,[3] dr ≥ 19:1) after purification by flash column chromatography (PE:MTBE 2:1) as a colorless oil.

^{1}H NMR (400 MHz, C_6D_6): δ = 3.62-3.49 (m, 4H), 3.26 (t, J = 11.2 Hz, 1H), 1.79-1.67 (m, 1H), 1.61-1.52 (m, 2H), 1.41 (s, 3H), 1.25 (s, 3H), 0.95 (d, J = 7.1 Hz, 3H), 0.32 (d, J = 6.6 Hz, 3H) ppm;

^{13}C{^{1}H} NMR (101 MHz, C_6D_6): δ = 98.2, 76.6, 66.7, 66.3, 35.9, 30.8, 30.0, 19.2, 12.0, 9.6 ppm;

HRMS (ESI): $C_9H_{17}O_3$ $[M–Me]^+$ calculated: 173.1178, found: 173.1176;

R$_f$ = 0.2 (PE:MTBE 3:1, vanillin);

$[\alpha]_D^{22}$ = –46.0 (c 1.0, $CHCl_3$).

[3] The material was halved after the aldol reaction.

2-4. Synthesis of the acetonide *anti*-motifs

General Procedure 5 (GP5): Oppolzer-Aldol Reaction, Hydrogenation, Acetonide Formation, Reduction

Note: Since the scale of the performed reactions differs due to need and availability, this general procedure reports the relative molar quantities, equivalents and relative solvent volumes of a representative experiment, whereby the exact volumes/weights of the structure-building (or key) chemicals are listed in the corresponding individual procedure.

The required propionated Oppolzer auxiliary (21.9 mmol, 1.0 equiv) was dissolved in CH_2Cl_2 (22.0 mL, 1.0 M) and Et_3N (32.8 mmol, 1.5 equiv) and TMSOTf (37.2 mmol, 1.7 equiv) were added successively over a period of 15 min. The reaction mixture was then stirred overnight at rt. A solution of the required aldehyde (32.8 mmol, 1.5 equiv) in CH_2Cl_2 (41 mL, 0.8 M refers to aldehyde) at –78 °C was treated with $TiCl_4$ (1.0 M in CH_2Cl_2, 32.8 mmol, 1.5 equiv) over a period of 15 min. The solution containing the (*Z*)-ketene acetal was cooled to –78 °C and then transferred to the cooled aldehyde containing solution via cannula. After complete addition, the reaction mixture was stirred at –78 °C until TLC showed full conversion before being quenched by the addition of sat. aq. NH_4Cl. After warming to 0 °C and stirring for further 30 min at this temperature the organic layer was separated. The aqueous layer was extracted with CH_2Cl_2 (3x), the combined organic layers were dried over Na_2SO_4, and the solvent was removed *in vacuo*. The crude material was purified by flash column chromatography.[3]

Through a suspension of the obtained *anti*-aldol product (21.9 mmol) and Pd/C (10 wt%, 2.19 mmol, 0.1 equiv) in EtOAc (220 mL, 0.1 M) were bubbled three balloons of H_2. The reaction mixture was stirred at rt under an atmosphere of H_2 until TLC showed full conversion. The reaction mixture was filtered through a short plug of Celite® (EtOAc) and concentrated *in vacuo*.

To a solution of the obtained diol (21.9 mmol) in 2,2-DMP/acetone (2:1, 55 mL, 0.4 M) was added PPTS (2.19 mmol, 0.1 equiv). The reaction mixture was stirred at rt until TLC showed full conversion. After the addition of sat. aq. $NaHCO_3$ and MTBE the organic layer was separated, and the aqueous layer was extracted with MTBE (3x). The combined organic layers were washed with sat. aq. NaCl, dried over Na_2SO_4 and concentrated *in vacuo*. The crude material was purified by flash column chromatography to afford the corresponding acetonide.

To a solution of the obtained acetonide (21.9 mmol) in THF (220 mL, 0.1 M) and MeOH (175 mmol, 8.0 equiv) at 0 °C was added $LiBH_4$ (4.0 M in THF, 175 mmol, 8.0 equiv) dropwise. The reaction mixture was stirred for 30 min at 0 °C and overnight at rt. The reaction mixture was then cooled to 0 °C and sat. aq. NaCl and MTBE were added. The organic layer was separated, and the aqueous layer was extracted with MTBE (3x). The combined organic layers were dried over Na_2SO_4 and concentrated *in vacuo*. The crude product was purified by flash column chromatography to afford the corresponding primary alcohol.[3]

General Procedure 6 (GP6): Oppolzer-Aldol Reaction, Reduction, Acetonide Formation, cleavage of TBDPS-ether

Note: Since the scale of the performed reactions differs due to need and availability, this general procedure reports the relative molar quantities, equivalents and relative solvent volumes of a representative experiment, whereby the exact volumes/weights of the structure-building (or key) chemicals are listed in the corresponding individual procedure.

The required propionated Oppolzer auxiliary (16.8 mmol, 1.0 equiv) was dissolved in CH_2Cl_2 (170 mL, 1.0 M) and Et_3N (25.3 mmol, 1.5 equiv) and TMSOTf (28.6 mmol, 1.7 equiv) were added successively over a period of 15 min. The reaction mixture was then stirred overnight at rt. A solution of the required aldehyde (25.3 mmol, 1.5 equiv) in CH_2Cl_2 (32 mL, 0.8 M refers to aldehyde) at –78 °C was treated with $TiCl_4$ (1.0 M in CH_2Cl_2, 25.3 mmol, 1.5 equiv) over a period of 15 min. The solution containing the (*Z*)-ketene acetal was cooled to –78 °C and then transferred to the cooled aldehyde containing solution via cannula. After complete addition, the reaction mixture was stirred at –78 °C until TLC showed full conversion before being quenched by the addition of sat. aq. NH_4Cl. After warming to 0 °C and stirring for further 30 min at this temperature the organic layer was separated. The aqueous layer was extracted with CH_2Cl_2 (3x), the combined organic layers were dried over $MgSO_4$, and the solvent was removed *in vacuo*. The crude product was purified by flash column chromatography to afford the corresponding *anti*-aldol product.[3]
To a solution of the obtained *anti*-aldol product (16.8 mmol) in THF (84 mL, 0.2 M) and MeOH (50.4 mmol, 3.0 equiv) at 0 °C was added $LiBH_4$ (4.0 M in THF, 50.4 mmol, 3.0 equiv) dropwise. The reaction mixture was warmed to rt and stirred at this temperature for 2 h, before a solution of NaOH (1.0 M) and EtOAc were added. The organic layer was separated, and the aqueous layer was extracted with EtOAc (3x). The combined organic layers were dried over $MgSO_4$ and concentrated *in vacuo*. The crude product was purified by flash column chromatography to afford the corresponding diol.
To a solution of the obtained diol (16.8 mmol) and 2,2-DMP (336 mmol, 20.0 equiv) in acetone (42 mL, 0.4 M) was added PPTS (0.84 mmol, 0.05 equiv). The reaction mixture was stirred at rt until TLC showed full conversion. After the addition of sat. aq. $NaHCO_3$ and MTBE the organic layer was separated, and the aqueous layer was extracted with MTBE (3x). The combined organic layers were washed with sat. aq. NaCl, dried over $MgSO_4$ and concentrated *in vacuo*. The crude material was purified by flash column chromatography to afford the corresponding acetonide.
To a solution of the obtained acetonide (16.8 mmol) in THF (84 mL, 0.2 M) was added TBAF (1.0 M in THF, 50.4 mmol, 3.0 equiv). The reaction mixture was stirred overnight at rt. After the addition of H_2O and MTBE the organic layer was separated, and the aqueous layer was extracted with MTBE (3x). The combined organic layers were washed with sat. aq. NaCl, dried over $MgSO_4$ and concentrated *in vacuo*. The crude material was purified by flash column chromatography to afford the corresponding primary alcohol.

Acetonide-protected 1,2-*anti* alcohol S10

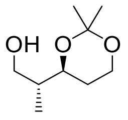

S10
$C_9H_{18}O_3$
174.24

Using GP5, (–)-*N*-propionyl sultam (5.94 g, 21.9 mmol, 1.0 equiv) and literature known 3-(benzyloxy)propanal (5.39 g, 32.8 mmol, 1.5 equiv)[12] gave primary alcohol **S10** (2.01 g, 11.5 mmol, 53% o4s, dr 19:1) after purification by flash column chromatography (PE:MTBE 1:1) as a colorless oil.

^{1}H NMR (400 MHz, C_6D_6): δ = 3.64-3.53 (m, 4H), 3.51-3.44 (m, 1H), 2.39 (dd, J = 7.2, 4.1 Hz, 1H), 1.62 (m$_c$, 1H), 1.46-1.38 (m, 1H), 1.37 (d, J = 0.5 Hz, 3H), 1.21 (d, J = 0.5 Hz, 3H), 0.88 (m$_c$, 1H), 0.65 (d, J = 7.0 Hz, 3H) ppm;

^{13}C{^{1}H} NMR (101 MHz, C_6D_6): δ = 98.3, 74.0, 66.8, 59.8, 41.0, 30.2, 29.5, 19.2, 12.8 ppm;

HRMS (EI): $C_8H_{15}O_3$ [M–CH$_3$]$^+$ calculated: 159.1021, found: 159.1021;

R$_f$ = 0.3 (PE:MTBE 1:1, vanillin);

$[\alpha]_D^{20}$ = +12.8 (*c* 0.6, $CHCl_3$).

Acetonide-protected *anti, syn* alcohol S11

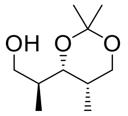

S11
$C_{10}H_{20}O_3$
188.27

Using GP4, (–)-4-benzyl-3-propionyl-2-oxazolidinone (6.48 g, 27.8 mmol, 1.1 equiv) and literature known (*S*)-3-((*tert*-butyldiphenylsilyl)oxy)-2-methylpropanal (8.25 g, 25.3 mmol, 1.0 equiv)[14] gave primary alcohol **S11** (2.38 g, 12.6 mmol, 73% o4s,[4] dr ≥ 19:1) after purification by flash column chromatography (PE:MTBE 1:1) as a colorless oil.

^{1}H NMR (400 MHz, C_6D_6): δ = 3.71-3.67 (m, 1H), 3.63-3.56 (m, 2H), 3.48 (d, J = 9.9 Hz, 1H), 3.37-3.34 (m, 1H), 2.51 (dd, J = 6.4, 4.8 Hz, 1H), 1.82-1.72 (m, 1H), 1.35 (d, J = 0.6 Hz, 3H), 1.21 (d, J = 0.4 Hz, 3H), 0.99 (d, J = 0.7 Hz, 4H), 0.50 (d, J = 6.9 Hz, 3H) ppm;

^{13}C{^{1}H} NMR (101 MHz, C_6D_6): δ = 98.6, 77.0, 68.0, 66.8, 37.0, 30.1, 30.0, 19.1, 11.9, 10.6 ppm;

HRMS (ESI): $C_9H_{17}O_3$ [M–Me]$^+$ calculated: 173.1178, found: 173.1178;

R$_f$ = 0.5 (PE:MTBE 1:1, vanillin);

[4] 9.00 g (16.1 mmol) of the aldol product were further modified.

$[\alpha]_D^{26}$ = +6.9 (*c* 0.6, $CHCl_3$).

Acetonide-protected *anti, anti* alcohol S12

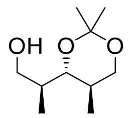

S12
$C_{10}H_{20}O_3$
188.27

Using GP6, (–)-*N*-propionyl sultam (4.57 g, 16.8 mmol, 1.0 equiv) and literature known (*S*)-3-((*tert*-butyldiphenylsilyl)oxy)-2-methylpropanal (8.25 g, 25.3 mmol, 1.5 equiv)[14] gave primary alcohol **S12** (0.60 g, 3.16 mmol, 68% o4s[5], dr 19:1) after purification by flash column chromatography (PE:MTBE 1:1) as a colorless oil.

^{1}H NMR (400 MHz, C_6D_6): δ = 3.83 (dq, *J* = 11.0, 2.0 Hz, 1H), 3.60-3.55 (m, 1H), 3.52 (dd, *J* = 11.4, 5.0 Hz, 1H), 3.25 (dd, *J* = 10.3, 2.4 Hz, 1H), 3.18 (t, *J* = 11.0 Hz, 1H), 2.39 (dd, *J* = 8.0, 2.0 Hz, 1H), 1.87-1.76 (m, 1H), 1.61-1.53 (m, 1H), 1.35 (s, 3H), 1.17 (s, 3H), 1.11 (d, *J* = 7.1 Hz, 3H), 0.33 (d, *J* = 6.7 Hz, 3H) ppm;

^{13}C{^{1}H} NMR (101 MHz, C_6D_6): δ = 98.6, 80.4, 66.1, 63.7, 35.6, 32.0, 29.9, 18.8, 15.4, 12.5 ppm;

HRMS (ESI): $C_9H_{17}O_3$ [M–Me]$^+$ calculated: 173.1178, found: 173.1178;

R*f* = 0.5 (PE:MTBE 1:1, vanillin);

$[\alpha]_D^{24}$ = –22.0 (*c* 1.0, $CHCl_3$).

[5] 2.50 g (4.18 mmol) of the aldol product were further modified.

2-5. Synthesis of open-chained stereotriad *syn*-motifs

syn, anti **alcohol S13**

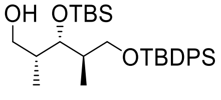

S13
$C_{29}H_{48}O_3Si_2$
500.87

Using GP1a, (+)-4-benzyl-3-propionyl-2-oxazolidinone (1.10 g, 4.71 mmol, 1.0 equiv) and literature known (*R*)-3-((*tert*-butyldiphenylsilyl)oxy)-2-methylpropanal (1.99 g, 6.09 mmol, 1.3 equiv)[14] gave primary alcohol **S13** (0.97 g, 1.94 mmol, 41% o3s, dr ≥ 19:1) after purification by flash column chromatography (PE:MTBE 4:1) as a colorless oil.

^{1}H NMR (400 MHz, $CDCl_3$): δ = 7.67-7.64 (m, 4H), 7.44-7.35 (m, 6H), 3.79-3.75 (m, 2H), 3.51 (dd, J = 10.2, 7.8 Hz, 1H), 3.44-3.40 (m, 2H), 2.00-1.93 (m, 1H), 1.88-1.82 (m, 1H), 1.68 (brs, 1H), 1.06 (s, 9H), 0.93 (d, J = 6.9 Hz, 3H), 0.84 (d, J = 6.9 Hz, 3H), 0.84 (s, 9H), 0.02 (s, 3H), −0.10 (s, 3H) ppm;

^{13}C{^{1}H} NMR (101 MHz, $CDCl_3$): δ = 135.796, 135.790, 133.99, 133.97, 129.71, 129.70, 127.76, 127.75, 74.4, 66.73, 66.68, 40.5, 38.8, 27.1, 26.1, 19.4, 18.3, 14.2, 11.9, −4.0, −4.2 ppm;

HRMS (ESI): $C_{29}H_{48}O_3Si_2Na$ $[M+Na]^+$ calculated: 523.3040, found: 523.3019;

R*f* = 0.4 (PE:MTBE 4:1, vanillin);

$[\alpha]_D^{20}$ = −3.1 (*c* 1.0, $CHCl_3$).

The enantiomer was prepared using (−)-4-benzyl-3-propionyl-2-oxazolidinone and (*S*)-3-((*tert*-butyldiphenylsilyl)oxy)-2-methylpropanal.

Analytical data are in accordance with the literature.[2]

all-*syn* alcohol S14

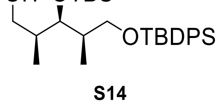

S14
$C_{29}H_{48}O_3Si_2$
500.87

Using GP1a, (−)-4-benzyl-3-propionyl-2-oxazolidinone (1.10 g, 4.71 mmol, 1.0 equiv) and literature known (*R*)-3-((*tert*-butyldiphenylsilyl)oxy)-2-methylpropanal (1.99 g, 6.09 mmol, 1.3 equiv)[14] gave primary alcohol **S14** (1.21 g, 2.42 mmol, 51% o3s, dr ≥ 19:1) after purification by flash column chromatography (PE:MTBE 4:1) as a colorless oil.

^{1}H NMR (400 MHz, $CDCl_3$): δ = 7.67-7.63 (m, 4H), 7.43-7.36 (m, 6H), 3.98 (t, J = 3.3 Hz, 1H), 3.67 (dd, J = 10.6, 8.4 Hz, 1H), 3.53-3.41 (m, 3H), 2.33 (brs, 1H), 2.01-1.95 (m, 1H), 1.90-

1.84 (m, 1H), 1.06 (s, 9H), 0.89 (d, J = 6.8 Hz, 3H), 0.88 (s, 9H), 0.81 (d, J = 7.0 Hz, 3H), 0.09 (s, 3H), 0.01 (s, 3H) ppm;

$^{13}C\{^1H\}$ NMR (101 MHz, $CDCl_3$): δ = 135.8, 135.7, 134.0, 133.9, 129.76, 129.75, 127.78, 127.76, 74.3, 67.0, 66.5, 40.6, 37.8, 27.0, 26.1, 19.4, 18.4, 13.2, 12.4, −4.0, −4.3 ppm;

HRMS (ESI): $C_{29}H_{48}O_3Si_2Na$ $[M+Na]^+$ calculated: 523.3040, found: 523.3024;

R$_f$ = 0.4 (PE:MTBE 4:1, vanillin);

$[\alpha]_D^{20}$ = −5.1 (c 1.0, $CHCl_3$).

2-6. Synthesis of open-chained stereotriad *anti*-motifs

anti, syn **alcohol S15**

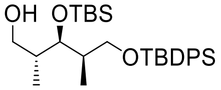

S15
$C_{29}H_{48}O_3Si_2$
500.87

Using GP2a, (−)-N-propionyl sultam (1.65 g, 6.09 mmol, 1.0 equiv) and literature known (R)-3-((*tert*-butyldiphenylsilyl)oxy)-2-methylpropanal (2.98 g, 9.13 mmol, 1.5 equiv)[14] gave primary alcohol **S15** (2.33 g, 4.65 mmol, 76% o3s, dr ≥ 19:1) after purification by flash column chromatography (PE:MTBE 6:1) as a colorless oil.

^1H NMR (400 MHz, C_6D_6): δ = 7.80-7.76 (m, 4H), 7.27-7.22 (m, 6H), 3.93 (dd, J = 6.4, 2.7 Hz, 1H), 3.63 (m_c, 2H), 3.51 (dd, J = 5.1, 4.5 Hz, 2H), 2.90 (m_c, 1H), 1.76 (m_c, 1H), 1.51 (t, J = 4.9 Hz, 1H), 1.20 (s, 9H), 0.95 (s, 9H), 0.92 (d, J = 6.9 Hz, 3H), 0.88 (d, J = 7.0 Hz, 3H), 0.12 (s, 3H), 0.05 (s, 3H) ppm;

$^{13}C\{^1H\}$ NMR (101 MHz, C_6D_6): δ = 136.1, 134.3, 134.2, 130.08, 130.06, 128.1, 74.8, 67.4, 65.4, 40.3, 39.5, 27.2, 26.4, 19.5, 18.6, 14.8, 11.5, −3.85, −3.93 ppm;

HRMS (ESI): $C_{29}H_{48}O_3Si_2Na$ $[M+Na]^+$ calculated: 523.3040, found: 523.3055;

R$_f$ = 0.3 (PE:MTBE 6:1, vanillin);

$[\alpha]_D^{20}$ = +1.31 (c 1.2, $CHCl_3$).

***anti, anti* alcohol S16**

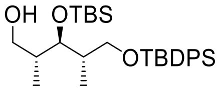

S16
$C_{29}H_{48}O_3Si_2$
500.87

Using GP2a, (−)-*N*-propionyl sultam (1.38 g, 5.07 mmol, 1.0 equiv) and literature known (*S*)-3-((*tert*-butyldiphenylsilyl)oxy)-2-methylpropanal (2.48 g, 7.61 mmol, 1.5 equiv)[13] gave primary alcohol **S16** (1.56 g, 3.11 mmol, 61% o3s, dr 19:1) after purification by flash column chromatography (PE:MTBE 6:1) as a colorless oil.

^{1}H NMR (400 MHz, C_6D_6): δ = 7.80-7.77 (m, 4H), 7.27-7.22 (m, 6H), 3.85 (dd, J = 10.1, 6.4 Hz, 1H), 3.70 (t, J = 4.9 Hz, 1H), 3.60-3.50 (m, 3H), 2.12 (m_c, 1H), 1.83 (m_c, 1H), 1.80-1.74 (m, 1H), 1.19 (s, 9H), 0.99 (d, J = 6.9 Hz, 3H), 0.93 (d, J = 7.0 Hz, 3H), 0.89 (s, 9H), 0.06 (s, 3H), −0.03 (s, 3H) ppm;

^{13}C{^{1}H} NMR (101 MHz, C_6D_6): δ = 136.075, 136.066, 134.23, 134.22, 130.06, 130.03, 128.12, 128.11, 78.3, 66.6, 65.5, 41.3, 37.8, 27.2, 26.3, 19.5, 18.4, 16.1, 13.9, −4.02, −4.04 ppm;

HRMS (ESI): $C_{29}H_{48}O_3Si_2Na$ $[M+Na]^+$ calculated: 523.3040, found: 523.3027;

R$_f$ = 0.3 (PE:MTBE 6:1, vanillin);

$[\alpha]_D^{20}$ = +3.68 (c 3.0, $CHCl_3$).

The enantiomer was prepared using (+)-*N*-propionyl sultam and (*R*)-3-((*tert*-butyldiphenylsilyl)oxy)-2-methylpropanal.

2-7. Synthesis of TIB esters

General Procedure 7 (GP7): Mitsunobu Conditions

Note: Since the scale of the performed reactions differs due to need and availability, this general procedure reports the relative molar quantities, equivalents and relative solvent volumes of a representative experiment, whereby the exact volumes/weights of the structure-building (or key) chemicals are listed in the corresponding individual procedure.

The required primary alcohol (4.06 mmol, 1.1 equiv) was dissolved in anhydrous THF (12.5 mL, 0.3 M), PPh_3 (3.69 mmol, 1.0 equiv) and TIBOH (3.69 mmol, 1.0 equiv) were added successively. After cooling to 0 °C, DIAD (4.06 mmol, 1.1 equiv, 0.12 mL/min) was added, the reaction mixture was slowly warmed to rt and stirred overnight at that temperature. MTBE and sat. aq. $NaHCO_3$ were added and the phases separated. The aqueous phase was extracted with MTBE (3x), the organic layers combined and dried over Na_2SO_4. The crude material was loaded on silica and purified by flash column chromatography.

TIB ester 9a

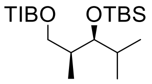

9a
$C_{29}H_{52}O_3Si$
476.82

Using GP7 and primary alcohol **S1** (1.00 g, 4.06 mmol, 1.1 equiv) gave TIB ester **9a** (1.49 g, 3.12 mmol, 85%) after purification by flash column chromatography (PE:MTBE 98:2) as a colorless oil.

^{1}H NMR (400 MHz, $CDCl_3$): δ = 7.01 (s, 2H), 4.21 (m_c, 2H), 3.49 (dd, J = 5.6, 3.1 Hz, 1H), 2.95- 2.77 (m, 3H), 2.08 (m_c, 1H), 1.78 (m_c, 1H), 1.24 (m_c, 18H), 0.86 (d, J = 7.2 Hz, 3H), 0.93-0.85 (m, 15H), 0.06 (s, 3H), 0.04 (s, 3H) ppm;

^{13}C{^{1}H} NMR (101 MHz, $CDCl_3$): δ = 171.2, 150.1, 144.9, 130.8, 121.0, 77.2, 68.4, 36.5, 34.5, 32.1, 31.7, 26.2, 24.5, 24.3, 24.1, 20.1, 19.0, 18.6, 12.3, −3.7, −3.8 ppm;

HRMS (ESI): $C_{29}H_{52}O_3SiNa$ $[M+Na]^+$ calculated: 499.3583, found: 499.3589;

R$_f$ = 0.3 (PE:MTBE 98:2, uv, vanillin);

$[\alpha]_D^{20}$ = +9.4 (c 0.7, $CHCl_3$).

TIB ester 10a

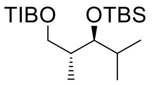

10a
$C_{29}H_{52}O_3Si$
476.82

Using GP7 and primary alcohol **S4** (500 mg, 2.03 mmol, 1.1 equiv) gave TIB ester **10a** (815 mg, 1.71 mmol, 92%) after purification by flash column chromatography (PE:MTBE 100:1) as a colorless oil.

^{1}H NMR (400 MHz, $CDCl_3$): δ = 7.00 (s, 2H), 4.51 (dd, J = 10.8, 4.0 Hz, 1H), 4.85 (dd, J = 10.7, 8.3 Hz, 1H), 3.39 (t, J = 4.7 Hz, 1H), 2.96-2.78 (m_c, 3H), 2.11-2.00 (m, 1H), 1.88-1.77 (m, 1H), 1.28-1.20 (m, 18H), 1.02 (d, J = 7.0 Hz, 3H), 0.95-0.86 (m, 15H), 0.09 (s, 3H), 0.06 (s, 3H) ppm;

^{13}C{^{1}H} NMR (101 MHz, $CDCl_3$): δ = 171.3, 150.1, 144.9, 131.0, 121.0, 79.4, 68.2, 36.9, 36.8, 34.5, 31.8, 31.7, 26.3, 24.4, 24.3, 24.1, 20.3, 20.3, 18.6, 17.9, 17.9, 15.8, −3.6, −3.8 ppm;

HRMS (ESI): $C_{29}H_{52}O_3SiNa$ $[M+Na]^+$ calculated: 499.3583, found: 499.3582;

R$_f$ = 0.3 (PE:MTBE 98:2, uv, vanillin);

$[\alpha]_D^{20}$ = +7.4 (c 0.5, $CHCl_3$).

TIB ester 25a

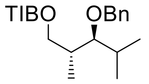

25a
$C_{30}H_{44}O_3$
452.68

Using GP7 and Bn-protected primary alcohol **S6** (247 mg, 1.11 mmol, 1.1 equiv) gave TIB ester **25a** (310 mg, 0.68 mmol, 67%) after purification by flash column chromatography (cyclohexane:EtOAc 97:3) as a colorless oil.

^{1}H NMR (400 MHz, $CDCl_3$): δ = 7.37-7.27 (m, 5H), 7.01 (s, 2H), 4.59 (s, 2H), 4.50 (dd, J = 10.9, 3.6 Hz, 1H), 4.32 (dd, J = 10.7, 6.6 Hz, 1H), 3.16 (dd, J = 7.3, 4.3 Hz, 1H), 2.92-2.82 (m, 3H), 2.17-2.10 (m, 1H), 1.97-1.90 (m, 1H), 1.26-1.22 (m, 18H), 1.04 (d, J = 5.3 Hz, 3H), 1.03 (d, J = 5.3 Hz, 3H), 0.96 (d, J = 6.7 Hz, 3H) ppm;

^{13}C{^{1}H} NMR (101 MHz, $CDCl_3$): δ = 171.4, 150.1, 144.9, 139.0, 130.9, 128.5, 127.6, 127.5, 121.0, 86.2, 75.4, 67.7, 36.0, 34.5, 31.7, 30.6, 24.5, 24.4, 24.1, 20.6, 16.8, 15.3 ppm;

HRMS (ESI): $C_{30}H_{44}O_3Na$ $[M+Na]^+$ calculated: 475.3188, found: 475.3179;

R$_f$ = 0.4 (cyclohexane:EtOAc 97:3, uv, vanillin);

$[\alpha]_D^{20}$ = +3.8 (c 1.2, $CHCl_3$).

TIB ester 29a

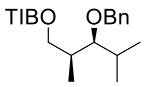

30a
$C_{30}H_{44}O_3$
452.68

Using GP7 and Bn-protected primary alcohol **S3** (180 mg, 0.81 mmol, 1.1 equiv) gave TIB ester **30a** (290 mg, 0.64 mmol, 86%) after purification by flash column chromatography (PE:MTBE 99:1) as a colorless oil.

^{1}H NMR (400 MHz, $CDCl_3$): δ = 7.35-7.27 (m, 5H), 7.02 (s, 2H), 4.59 (m_c, 2H), 4.34 (dd, J = 10.8, 6.6 Hz, 1H), 4.24 (dd, J = 10.8, 7.6 Hz, 1H), 3.21 (dd, J = 7.7, 3.5 Hz, 1H), 2.94-2.83 (m, 3H), 2.21 (m_c, 1H), 1.92 (m_c, 1H), 1.27-1.24 (m, 18H), 1.04-1.02 (m, 6H), 0.91 (d, J = 6.8 Hz, 3H), ppm;

^{13}C{^{1}H} NMR (101 MHz, $CDCl_3$): δ = 171.2, 150.2, 144.9, 139.0, 130.8, 128.5, 127.6, 127.5, 121.0, 85.1, 75.3, 68.0, 35.4, 34.5, 31.7, 31.2, 24.43, 24.36, 24.1, 19.8, 19.4, 11.4 ppm;

HRMS (ESI): $C_{30}H_{44}O_3Na$ $[M+Na]^+$ calculated: 475.3188, found: 475.3187;

R$_f$ = 0.3 (PE:MTBE 95:5, uv, vanillin);

$[\alpha]_D^{20}$ = +33.0 (c 1.0, $CHCl_3$).

TIB ester 26a

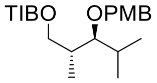

26a
$C_{31}H_{46}O_4$
482.71

Using GP7 and PMB-protected primary alcohol **S5** (254 mg, 1.01 mmol, 1.1 equiv) gave TIB ester **26a** (317 mg, 0.66 mmol, 72%) after purification by flash column chromatography (PE:MTBE 100:1) as a pale yellow oil.

^{1}H NMR (400 MHz, $CDCl_3$): δ = 7.28 (d, J = 8.6 Hz, 2H), 7.01 (s, 2H), 6.87-6.88 (m, 2H), 4.53-4.47 (m, 3H), 4.31 (dd, J = 10.9, 6.6 Hz, 1H), 3.81 (s, 3H), 3.14 (dd, J = 7.4, 4.3 Hz, 1H), 2.92-2.83 (m, 3H), 2.16-2.11 (m, 1H), 1.95-1.87 (m, 1H), 1.27-1.23 (m, 18H), 1.05-1.01 (m, 6H), 0.95 (d, J = 7.0 Hz, 3H) ppm;

^{13}C{^{1}H} NMR (101 MHz, $CDCl_3$): δ = 171.4, 159.3, 150.1, 144.9, 131.2, 130.9, 129.2, 121.0, 113.9, 85.9, 75.0, 67.8, 55.4, 36.0, 34.5, 31.7, 30.5, 24.5, 24.4 24.1, 20.7, 16.8, 15.3 ppm;

HRMS (ESI): $C_{31}H_{46}O_4Na$ $[M+Na]^+$ calculated: 505.3294, found: 505.3285;

R$_f$ = 0.4 (PE:MTBE 100:1, uv, vanillin);

$[\alpha]_D^{20}$ = +40.0 (c 0.5, $CHCl_3$).

TIB ester 30a

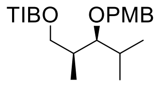

31a
$C_{31}H_{46}O_4$
482.71

Using GP7 and PMB-protected primary alcohol **S2** (205 mg, 0.81 mmol, 1.1 equiv) gave TIB ester **31a** (259 mg, 0.54 mmol, 73%) after purification by flash column chromatography (PE:MTBE 97:3) as a colorless oil.

^{1}H NMR (400 MHz, C_6D_6): δ = 7.29-7.25 (m, 2H), 7.11 (s, 2H), 6.82-6.78 (m, 2H), 4.51-4.36 (m, 4H), 3.31 (s, 3H), 3.25-3.13 (m, 3H), 2.77 (sept, J = 6.9 Hz, 1H), 2.21 (m_c, 1H), 1.84 (m_c, 1H), 1.33-1.30 (m, 12H), 1.20 (d, J = 6.9 Hz, 6H), 1.01-0.99 (m, 6H), 0.80 (d, J = 6.8 Hz, 3H) ppm;

^{13}C{^{1}H} NMR (101 MHz, C_6D_6): δ = 170.7, 159.7, 150.4, 145.5, 131.9, 131.6, 129.3, 121.2, 114.1, 84.9, 75.3, 68.1, 54.8, 35.7, 34.9, 32.2, 31.4, 24.5, 24.4, 24.2, 19.8, 19.3, 11.5 ppm;

HRMS (ESI): $C_{31}H_{46}O_4Na$ $[M+Na]^+$ calculated: 505.3294, found: 505.3298;

R_f = 0.4 (PE:MTBE 95:5, uv, CAN);

$[\alpha]_D^{20}$ = +39.3 (*c* 3.2, MeOH).

TIB ester 33a

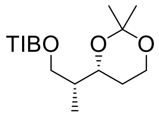

33a
$C_{25}H_{40}O_4$
404.59

Using GP7 and primary alcohol **S7** (0.50 g, 2.87 mmol, 1.1 equiv) gave TIB ester **33a** (1.02 g, 2.52 mmol, ≥ 95%) after purification by flash column chromatography (PE:MTBE 95:5 → 9:1) as a colorless oil.

^{1}H NMR (400 MHz, C_6D_6): δ = 7.10 (s, 2H), 4.42 (dd, J = 10.9, 5.7 Hz, 1H), 4.27 (dd, J = 11.0, 6.3 Hz, 1H), 3.72 (m_c, 1H), 3.63-3.60 (m, 2H), 3.14 (sep, J = 6.8 Hz, 2H), 2.76 (sep, J = 6.9 Hz, 1H), 1.84 (m_c, 1H), 1.62-1.49 (m, 1H), 1.46 (s, 3H), 1.29 (d, J = 6.8 Hz, 12H), 1.24 (s, 3H), 1.20 (d, J = 6.9 Hz, 6H), 1.04 (d, J = 6.9 Hz, 3H), 0.87 (m_c, 1H) ppm;

^{13}C{^{1}H} NMR (101 MHz, C_6D_6): δ = 170.7, 150.4, 145.4, 131.9, 121.2, 98.4, 69.6, 67.0, 59.8, 38.2, 34.9, 32.1, 30.2, 28.6, 24.44, 24.37, 24.2, 19.3, 12.2 ppm;

HRMS (ESI): $C_{25}H_{40}O_4Na$ $[M+Na]^+$ calculated: 427.2824, found: 427.2803;

R_f = 0.3 (PE:MTBE 9:1, uv, vanillin);

$[\alpha]_D^{20}$ = –16.1 (*c* 1.5, $CHCl_3$).

TIB ester 34a

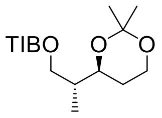

34a
$C_{25}H_{40}O_4$
404.59

Using GP7 and primary alcohol **S10** (0.70 g, 4.02 mmol, 1.1 equiv) gave TIB ester **34a** (1.16 g, 2.87 mmol, 79%) after purification by flash column chromatography (PE:MTBE 95:5 → 9:1) as a colorless oil.

^{1}H NMR (400 MHz, C_6D_6): δ = 7.10 (s, 2H), 4.53 (dd, J = 10.8, 4.4 Hz, 1H), 4.41 (dd, J = 10.8, 6.3 Hz, 1H), 3.65-3.54 (m, 3H), 3.14 (sep, J = 6.8 Hz, 2H), 2.77 (sep, J = 6.9 Hz, 1H), 1.88 (m_c, 1H), 1.47 (s, 3H), 1.45-1.35 (m, 1H), 1.29 (d, J = 6.8 Hz, 12H), 1.25 (s, 3H), 1.20 (d, J = 6.9 Hz, 6H), 0.94 (m_c, 1H), 0.84 (d, J = 6.9 Hz, 3H) ppm;

^{13}C{^{1}H} NMR (101 MHz, C_6D_6): δ = 170.8, 150.3, 145.5, 132.0, 121.2, 98.4, 70.0, 66.5, 59.8, 38.5, 34.9, 32.1, 30.3, 28.8, 24.45, 24.42, 24.2, 19.3, 12.8 ppm;

HRMS (ESI): $C_{25}H_{40}O_4Na$ $[M+Na]^+$ calculated: 427.2824, found: 427.2837;

R$_f$ = 0.3 (PE:MTBE 9:1, uv, vanillin);

$[\alpha]_D^{20}$ = +13.9 (c 2.2, $CHCl_3$).

TIB ester 43a

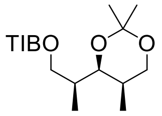

43a
$C_{26}H_{42}O_4$
418.62

Using GP7 and primary alcohol **S8** (240 mg, 1.27 mmol, 1.1 equiv) gave TIB ester **43a** (480 mg, 1.15 mmol, ≥ 95%) after purification by flash column chromatography (PE:EtOAc 20:1) as a colorless oil.

^{1}H NMR (400 MHz, C_6D_6): δ = 7.09 (s, 2H), 4.24 (dd, J = 11.2, 3.5 Hz, 1H), 4.12 (dd, J = 11.2, 5.7 Hz, 1H), 3.83 (dd, J = 11.5, 2.7 Hz, 1H), 3.63 (dd, J = 9.6, 2.4 Hz, 1H), 3.44 (dd, J = 11.5, 1.6 Hz, 1H), 3.12 (sep, J = 6.8 Hz, 2H), 2.75 (sep, J = 7.0 Hz, 1H), 1.99-1.90 (m, 1H), 1.46 (s, 3H), 1.35-1.30 (m, 1H), 1.29 (d, J = 6.8 Hz, 6H), 1.29 (d, J = 6.9 Hz, 6H), 1.21 (s, 3H), 1.19 (d, J = 7.0 Hz, 6H), 1.17 (d, J = 6.8 Hz, 3H), 1.10 (d, J = 6.9 Hz, 3H) ppm;

^{13}C{^{1}H} NMR (101 MHz, C_6D_6): δ = 170.9, 150.5, 145.4, 131.7, 121.3, 98.9, 73.4, 66.8, 66.1, 34.9, 34.9, 32.2, 30.7, 30.1, 24.4, 24.4, 24.2, 19.0, 14.5, 11.0 ppm;

HRMS (ESI): $C_{26}H_{42}O_4Na$ $[M+Na]^+$ calculated: 441.2981, found: 441.2965;

R$_f$ = 0.2 (PE:EtOAc 19:1, uv, vanillin);

$[\alpha]_D^{26}$ = +9.0 (*c* 1.0, $CHCl_3$).

TIB ester 46a

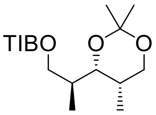

46a
$C_{26}H_{42}O_4$
418.62

Using GP7 and primary alcohol **S11** (700 mg, 3.72 mmol, 1.1 equiv) gave TIB ester **46a** (1.33 g, 3.18 mmol, 94%) after purification by flash column chromatography (PE:EtOAc 20:1) as a colorless amorphous solid.

^{1}H NMR (400 MHz, C_6D_6): δ = 7.11 (s, 2H), 4.57 (dd, *J* = 10.7, 3.3 Hz, 1H), 4.50 (dd, *J* = 10.7, 5.6 Hz, 1H), 3.73 (dd, *J* = 11.4, 2.1 Hz, 1H), 3.56 (dd, *J* = 10.1, 2.0 Hz, 1H), 3.40 (dd, *J* = 11.4, 1.3 Hz, 1H), 3.15 (sep, *J* = 6.8 Hz, 2H), 2.77 (sep, *J* = 6.9 Hz, 1H), 1.92-1.82 (m, 1H), 1.46 (s, 3H), 1.29 (d, *J* = 6.8 Hz, 12H), 1.26 (s, 3H), 1.20 (d, *J* = 6.9 Hz, 6H), 1.05-0.99 (m, 4H), 0.77 (d, *J* = 6.9 Hz, 3H) ppm;

^{13}C{^{1}H} NMR (101 MHz, C_6D_6): δ = 170.9, 150.3, 145.5, 132.1, 121.2, 98.8, 72.2, 66.9, 66.8, 34.9, 34.9, 32.1, 30.1, 29.8, 24.5, 24.4, 24.2, 19.2, 12.2, 10.3 ppm;

HRMS (ESI): $C_{26}H_{42}O_4Na$ $[M+Na]^+$ calculated: 441.2981, found: 441.2976;

R*f* = 0.2 (PE:EtOAc 19:1, uv, vanillin);

$[\alpha]_D^{26}$ = –12.5 (*c* 0.3, $CHCl_3$).

TIB ester 49a

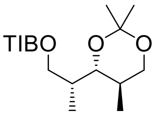

49a
$C_{26}H_{42}O_4$
418.62

Using GP7 and primary alcohol **S9** (240 mg, 1.27 mmol, 1.1 equiv) gave TIB ester **49a** (476 mg, 1.14 mmol, ≥ 95%) after purification by flash column chromatography (PE:EtOAc 20:1) as a colorless amorphous solid.

^{1}H NMR (400 MHz, C_6D_6): δ = 7.11 (s, 2H), 4.52 (dd, *J* = 10.7, 7.9 Hz, 1H), 4.39 (dd, *J* = 10.7, 6.4 Hz, 1H), 3.59 (dd, *J* = 10.3, 2.0 Hz, 1H), 3.55 (dd, *J* = 11.4, 5.0 Hz, 1H), 3.26 (t, *J* = 11.4 Hz, 1H), 3.18 (sep, *J* = 6.8 Hz, 2H), 2.77 (sep, *J* = 6.9 Hz, 1H), 2.18-2.09 (m, 1H), 1.79-1.67 (m, 1H), 1.48 (s, 3H), 1.31 (d, *J* = 6.8 Hz, 6H), 1.30 (d, *J* = 6.8 Hz, 6H), 1.27 (s, 3H), 1.20 (d, *J* = 6.9 Hz, 6H), 0.98 (d, *J* = 6.9 Hz, 3H), 0.33 (d, *J* = 6.7 Hz, 3H) ppm;

^{13}C{^{1}H} NMR (101 MHz, C_6D_6): δ = 170.7, 150.4, 145.4, 132.0, 121.2, 98.4, 74.2, 67.6, 66.2, 34.9, 33.9, 32.1, 30.7, 30.0, 24.5, 24.4, 24.2, 19.2, 11.9, 9.9 ppm;

HRMS (ESI): $C_{26}H_{42}O_4Na$ $[M+Na]^+$ calculated: 441.2981, found: 441.2976;

$\mathbf{R}_f$ = 0.3 (PE:EtOAc 19:1, uv, vanillin);

$[\alpha]_D^{27}$ = –32.0 (*c* 1.0, $CHCl_3$).

TIB ester 52a

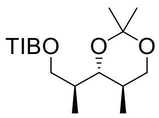

52a
$C_{26}H_{42}O_4$
418.62

Using GP7 and primary alcohol **S12** (240 mg, 1.27 mmol, 1.1 equiv) gave TIB ester **52a** (463 mg, 1.11 mmol, ≥ 95%) after purification by flash column chromatography (PE:EtOAc 20:1) as a colorless amorphous solid.

^{1}H NMR (400 MHz, C_6D_6): δ = 7.10 (s, 2H), 4.85 (dd, J = 11.0, 4.7 Hz, 1H), 4.33 (dd, J = 11.0, 8.7 Hz, 1H), 3.53 (dd, J = 11.4, 5.0 Hz, 1H), 3.27 (dd, J = 10.4, 2.4 Hz, 1H), 3.22-3.12 (m, 3H), 2.76 (sep, J = 6.9 Hz, 1H), 2.26-2.17 (m, 1H), 1.94-1.83 (m, 1H), 1.47 (s, 3H), 1.30 (d, J = 6.8 Hz, 6H), 1.30 (d, J = 6.8 Hz, 6H), 1.23 (s, 3H), 1.20 (d, J = 6.9 Hz, 6H), 1.14 (d, J = 6.9 Hz, 3H), 0.47 (d, J = 6.7 Hz, 3H) ppm;

^{13}C{^{1}H} NMR (101 MHz, C_6D_6): δ = 170.9, 150.3, 145.5, 132.0, 121.2, 98.5, 78.3, 66.6, 66.2, 34.9, 34.3, 32.1, 31.7, 29.8, 24.4, 24.4, 24.2, 19.1, 15.8, 12.7 ppm;

HRMS (ESI): $C_{26}H_{42}O_4Na$ $[M+Na]^+$ calculated: 441.2981, found: 441.2971;

$\mathbf{R}_f$ = 0.2 (PE:EtOAc 19:1, uv, vanillin);

$[\alpha]_D^{22}$ = –25.1 (*c* 1.0, $CHCl_3$).

TIB ester 55a

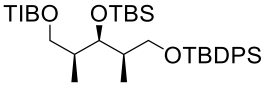

55a
$C_{45}H_{70}O_4Si_2$
731.22

Using a modified version of GP7 and primary alcohol **S14** (380 mg, 0.76 mmol, 1.0 equiv) gave TIB ester **55a** (550 mg, 0.75 mmol, ≥ 95%) after purification by flash column chromatography (PE:MTBE 100:1) as a colorless oil.

^{1}H NMR (400 MHz, $CDCl_3$): δ = 7.66-7.62 (m, 4H), 7.43-7.34 (m, 6H), 7.00 (s, 2H), 4.36 (dd, J = 10.6, 4.8 Hz, 1H), 4.10 (dd, J = 10.6, 8.8 Hz, 1H), 3.89 (t, J = 3.6 Hz, 1H), 3.54 (dd, J = 9.9, 7.2 Hz, 1H), 3.40 (dd, J = 9.9, 6.7 Hz, 1H), 2.92-2.80 (m, 3H), 2.11-2.04 (m, 1H), 1.89-1.83 (m, 1H), 1.25 (d, J = 6.9 Hz, 6H), 1.23 (d, J = 6.9 Hz, 12H), 1.03 (s, 9H), 0.95 (d, J = 6.8 Hz, 3H), 0.87 (s, 9H), 0.85 (d, J = 6.8 Hz, 3H), 0.05 (s, 3H), −0.02 (s, 3H) ppm;

^{13}C{^{1}H} NMR (101 MHz, $CDCl_3$): δ = 171.3, 150.1, 145.5, 144.9, 135.7, 134.0, 133.9, 130.9, 129.7, 129.7, 127.8, 127.7, 121.2, 121.0, 72.9, 68.0, 66.9, 38.7, 38.5, 34.6, 31.7, 27.0, 26.1, 24.43, 24.41, 24.3, 24.1, 19.3, 18.4, 13.8, 12.3, −4.0, −4.2 ppm;

HRMS (ESI): $C_{45}H_{70}O_4Si_2Na$ $[M+Na]^+$ calculated: 753.4710, found: 753.4691;

R$_f$ = 0.4 (PE:MTBE 98:2, uv, vanillin);

$[\alpha]_D^{20}$ = −5.7 (*c* 0.5, $CHCl_3$).

TIB ester 58a

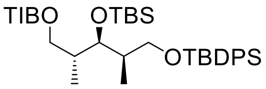

58a
$C_{45}H_{70}O_4Si_2$
731.22

Using GP7 and primary alcohol **S15** (1.30 g, 2.60 mmol, 1.1 equiv) gave TIB ester **58a** (1.71 g, 2.34 mmol, ≥ 95%) after purification by flash column chromatography (PE:MTBE 100:1) as a colorless oil.

^{1}H NMR (400 MHz, C_6D_6): δ = 7.80-7.77 (m, 4H), 7.26-7.22 (m, 6H), 7.10 (s, 2H), 4.79 (dd, *J* = 10.8, 3.7 Hz, 1H), 4.19 (m_c, 1H), 3.93 (dd, *J* = 6.8, 2.6 Hz, 1H), 3.66 (m_c, 2H), 3.17 (sep, *J* = 6.8 Hz, 2H), 2.77 (sep, *J* = 6.9 Hz, 1H), 2.21-2.12 (m, 1H), 1.92 (m_c, 1H), 1.30 (d, *J* = 6.9 Hz, 12H), 1.21 (d, *J* = 6.9 Hz, 6H), 1.19 (s, 9H), 1.03 (d, *J* = 6.9 Hz, 3H), 0.98 (s, 9H), 0.90 (d, *J* = 6.9 Hz, 3H), 0.18 (s, 3H), 0.05 (s, 3H) ppm;

^{13}C{^{1}H} NMR (101 MHz, C_6D_6): δ = 170.9, 150.3, 145.5, 136.08, 136.07, 134.3, 134.2, 132.0, 130.09, 130.07, 128.13, 128.12, 121.2, 73.7, 68.4, 67.3, 39.1, 38.2, 34.9, 32.1, 27.2, 26.4, 24.5, 24.4, 24.2, 19.5, 18.7, 15.1, 10.9, −3.7, −3.8 ppm;

HRMS (ESI): $C_{45}H_{70}O_4Si_2Na$ $[M+Na]^+$ calculated: 753.4710, found: 753.4696;

R$_f$ = 0.4 (PE:MTBE 98:2, uv, vanillin);

$[\alpha]_D^{20}$ = −0.90 (*c* 2.3, $CHCl_3$).

TIB ester 60a

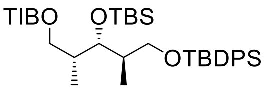

60a
$C_{45}H_{70}O_4Si_2$
731.22

Using GP7 and primary alcohol **S13** (550 mg, 1.10 mmol, 1.1 equiv) gave TIB ester **60a** (612 mg, 0.84 mmol, 84%) after purification by flash column chromatography (PE:MTBE 100:1) as a colorless oil.

^{1}H NMR (400 MHz, $CDCl_3$): δ = 7.65-7.63 (m, 4H), 7.42-7.32 (m, 6H), 7.00 (s, 2H), 4.20-4.11 (m, 2H), 3.79 (dd, J = 9.9, 5.1 Hz, 1H), 3.64 (dd, J = 6.6, 2.5 Hz, 1H), 3.35 (dd, J = 9.9, 8.2 Hz, 1H), 2.93-2.78 (m, 3H), 2.09 (m_c, 1H), 1.95-1.88 (m, 1H), 1.25 (d, J = 6.9 Hz, 6H), 1.22 (d, J = 6.8 Hz, 12H), 1.04 (s, 9H), 0.93 (dd, J = 6.9, 1.4 Hz, 6H), 0.80 (s, 9H), 0.00 (s, 3H), −0.17 (s, 3H) ppm;

^{13}C{^{1}H} NMR (101 MHz, $CDCl_3$): δ = 171.1, 150.1, 144.9, 135.8, 134.0, 130.8, 129.73, 129.71, 127.74, 127.73, 121.0, 74.0, 68.4, 66.6, 40.5, 36.1, 34.5, 31.7, 27.0, 26.1, 24.5, 24.3, 24.1, 19.4, 18.4, 14.4, 11.7, −3.9, −4.0 ppm;

HRMS (ESI): $C_{45}H_{70}O_4Si_2Na$ $[M+Na]^+$ calculated: 753.4710, found: 753.4717;

R$_f$ = 0.4 (PE:MTBE 98:2, uv, vanillin);

$[\alpha]_D^{20}$ = −5.4 (*c* 1.0, $CHCl_3$).

The enantiomer was prepared using ***ent*-S13**.

TIB ester 63a

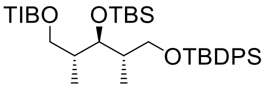

63a
$C_{45}H_{70}O_4Si_2$
731.22

Using GP7 and primary alcohol **S16** (507 mg, 1.01 mmol, 1.1 equiv) gave TIB ester **63a** (541 mg, 0.74 mmol, 80%) after purification by flash column chromatography (PE:MTBE 100:1) as a colorless oil.

^{1}H NMR (400 MHz, C_6D_6): δ = 7.80-7.78 (m, 4H), 7.26-7.22 (m, 6H), 7.10 (s, 2H), 4.78 (dd, J = 11.0, 4.1 Hz, 1H), 4.23 (m_c, 1H), 3.95 (m_c, 1H), 3.69 (t, J = 4.7 Hz, 1H), 3.58 (m_c, 1H), 3.17 (sep, J = 6.8 Hz, 2H), 2.77 (sep, J = 6.9 Hz, 1H), 2.30-2.23 (m, 1H), 2.14 (m_c, 1H), 1.29 (d, J = 6.9 Hz, 12H), 1.21-1.19 (m, 15H), 1.10-1.06 (m, 6H), 0.92 (s, 9H), 0.08 (s, 3H), −0.03 (s, 3H) ppm;

^{13}C{^{1}H} NMR (101 MHz, C_6D_6): δ = 170.9, 150.3, 145.5, 136.1, 134.2, 132.0, 130.1, 130.0, 128.14, 128.12, 121.2, 77.0, 68.0, 66.5, 40.6, 36.9, 34.9, 32.1, 27.2, 26.3, 24.5, 24.4, 24.2, 19.5, 18.5, 16.0, 14.5, −3.87, −3.94 ppm;

HRMS (ESI): $C_{45}H_{70}O_4Si_2Na$ $[M+Na]^+$ calculated: 753.4710, found: 753.4716;

R$_f$ = 0.4 (PE:MTBE 98:2, uv, vanillin);

$[\alpha]_D^{20}$ = +0.82 (*c* 2.0, $CHCl_3$).

The enantiomer was prepared using ***ent*-S16**.

2-8. Synthesis of carbamates

General Procedure 8 (GP8): CbCl

Note: Since the scale of the performed reactions differs due to need and availability, this general procedure reports the relative molar quantities, equivalents and relative solvent volumes of a representative experiment, whereby the exact volumes/weights of the structure-building (or key) chemicals are listed in the corresponding individual procedure.

The required primary alcohol (4.30 mmol, 1.0 equiv) was dissolved in 1,2-dichloroethane (14.5 mL, 0.3 M) and Et_3N (12.9 mmol, 3.0 equiv) and *N,N*-di*iso*propylcarbamoyl chloride (12.9 mmol, 3.0 equiv) were added successively. After heating to 70 °C overnight H_2O was added. The phases were separated and the aqueous phase was extracted with CH_2Cl_2 (3x), the organic layers were combined and dried over Na_2SO_4. The solvent was removed *in vacuo* and the crude material was purified by flash column chromatography to afford the corresponding carbamate.

Carbamate 9b

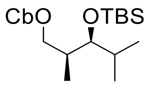

9b

$C_{20}H_{43}NO_3Si$

373.65

Using GP8 and primary alcohol **S1** (1.06 g, 4.30 mmol, 1.0 equiv) gave carbamate **9b** (1.55 g, 4.15 mmol, ≥ 95%) after purification by flash column chromatography (PE:MTBE 95:5) as a colorless oil.

^{1}H NMR (400 MHz, $CDCl_3$): δ = 4.20-3.75 (m, 4H), 3.48 (dd, J = 5.9, 2.7 Hz, 1H), 2.01 (m_c, 1H), 1.77 (m_c, 1H), 1.24-1.18 (m, 12H), 0.89 (m, 18H), 0.05 (s, 3H), 0.04 (s, 3H) ppm;

^{13}C{^{1}H} NMR (101 MHz, $CDCl_3$): δ = 155.8, 77.1, 68.2, 45.9 (brs), 36.0, 32.5, 26.3, 21.4 (brs), 19.8, 19.3, 18.6, 11.9, −3.6, −3.8 ppm;

HRMS (ESI): $C_{20}H_{43}NO_3SiNa$ $[M+Na]^+$ calculated: 396.2910, found: 396.2899;

R_f = 0.2 (PE:MTBE 95:5, vanillin);

$[\alpha]_D^{20}$ = +10.3 (c 0.5, $CHCl_3$).

Carbamate 10b

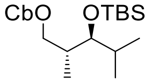

10b
$C_{20}H_{43}NO_3Si$
373.65

Using GP8 and primary alcohol **S4** (400 mg, 1.62 mmol, 1.0 equiv) gave carbamate **10b** (600 mg, 1.61 mmol, ≥ 95%) after purification by flash column chromatography (PE:MTBE 95:5) as a colorless oil.

^{1}H NMR (400 MHz, $CDCl_3$): δ = 4.20 (dd, J = 10.7, 4.9 Hz, 1H), 4.15-3.61 (m, 3H), 3.41 (dd, J = 5.2, 4.3 Hz, 1H), 2.06-1.95 (m, 1H), 1.87-1.76 (m, 1H), 1.24-1.18 (m, 12H), 0.98 (t, J = 7.0 Hz, 3H), 0.93 (t, J = 6.9 Hz, 3H), 0.91 (s, 9H), 0.88 (t, J = 6.8 Hz, 3H), 0.05 (s, 3H), 0.04 (s, 3H) ppm;

^{13}C{^{1}H} NMR (101 MHz, $CDCl_3$): δ = 156.0, 79.0, 67.5, 45.8 (brs), 37.3, 31.4, 26.3, 21.2 (brs), 20.7, 18.6, 17.7, 15.5, −3.8, −3.8 ppm;

HRMS (ESI): $C_{20}H_{43}NO_3SiNa$ $[M+Na]^+$ calculated: 396.2910, found: 396.2907;

R$_f$ = 0.3 (PE:MTBE 95:5, $KMnO_4$);

$[\alpha]_D^{20}$ = +10.7 (*c* 0.5, $CHCl_3$).

Carbamate 25b

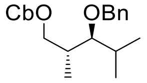

25b
$C_{21}H_{35}NO_3$
349.52

Using GP8 and Bn-protected primary alcohol **S6** (382 mg, 1.72 mmol, 1.0 equiv) gave carbamate **25b** (291 mg, 0.83 mmol, 48%) after purification by flash column chromatography (PE:EtOAc 19:1) as a pale yellow oil.

^{1}H NMR (400 MHz, $CDCl_3$): δ = 7.37-7.26 (m, 5H), 4.57 (s, 2H), 4.27 (dd, J = 10.6, 4.0 Hz, 1H), 4.13 (dd, J = 10.6, 6.9 Hz, 1H), 3.92 (brs, 2H), 3.12-3.09 (m, 1H), 2.16-2.00 (m, 1H), 1.98-1.89 (m, 1H), 1.23-1.21 (m, 12H), 1.04-1.02 (m, 6H), 0.97 (d, J = 6.7 Hz, 3H) ppm;

^{13}C{^{1}H} NMR (101 MHz, $CDCl_3$): δ = 156.0, 139.1, 128.4, 127.7, 127.5, 86.8, 75.4, 67.1, 46.0 (brs), 36.3, 30.6, 21.2 (brs), 20.7, 17.2, 15.5 ppm;

HRMS (ESI): $C_{21}H_{35}NO_3Na$ $[M+Na]^+$ calculated: 372.2515, found: 372.2509;

R$_f$ = 0.2 (PE:MTBE 95:5, uv, vanillin);

$[\alpha]_D^{20}$ = +26.0 (*c* 0.5, $CHCl_3$).

Carbamate 26b

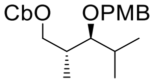

26b

$C_{22}H_{37}NO_4$

379.54

Using GP8 and PMB-protected primary alcohol **S5** (191 mg, 0.75 mmol, 1.0 equiv) gave carbamate **26b** (113 mg, 0.30 mmol, 40%) after purification by flash column chromatography (PE:EtOAc 19:1) as a pale yellow oil.

^{1}H NMR (400 MHz, $CDCl_3$): δ = 7.28 (d, J = 8.7 Hz, 2H), 6.87 (d, J = 8.6 Hz, 2H), 4.49 (s, 2H), 4.25 (dd, J = 10.5, 3.9 Hz, 1H), 4.12 (dd, J = 10.3, 6.9 Hz, 1H), 4.95 (bs, 2H) 3.79 (s, 3H), 3.08 (dd, J = 7.2, 4.6 Hz, 1H), 2.13-2.03 (m, 1H), 1.98-1.88 (m, 1H), 1.23-1.20 (m, 12H), 1.05-0.98 (m, 6H), 0.94 (d, J = 6.7 Hz, 3H) ppm;

^{13}C{^{1}H} NMR (101 MHz, $CDCl_3$): δ = 159.2, 156.0, 131.3, 129.4, 113.9, 86.4, 75.1, 67.2, 55.4, 46.0 (brs), 36.3, 30.6, 21.2 (brs), 20.8, 17.1, 15.5 ppm;

HRMS (ESI): $C_{22}H_{37}NO_4Na$ $[M+Na]^+$ calculated: 402.2620, found: 402.2617;

R$_f$ = 0.3 (PE:MTBE 95:5, uv, vanillin);

$[\alpha]_D^{20}$ = +3.8 (*c* 1.2, $CHCl_3$).

Carbamate 29b

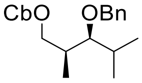

29b

$C_{21}H_{35}NO_3$

349.52

Using GP8 and Bn-protected primary alcohol **S3** (170 mg, 0.76 mmol, 1.0 equiv) gave carbamate **29b** (178 mg, 0.51 mmol, 67%) after purification by flash column chromatography (PE:MTBE 95:5) as a colorless oil.

^{1}H NMR (400 MHz, $CDCl_3$): δ = 7.37-7.25 (m, 5H), 4.58 (m_c, 2H), 4.11-3.70 (m, 4H), 3.17 (dd, J = 7.4, 3.6 Hz, 1H), 2.13 (m_c, 1H), 1.92 (m_c, 1H), 1.24-1.21 (m, 12H), 1.02 (d, J = 6.7 Hz, 3H), 0.99 (d, J = 6.9 Hz, 3H), 0.92 (d, J = 6.8 Hz, 3H) ppm;

^{13}C{^{1}H} NMR (101 MHz, $CDCl_3$): δ = 155.8, 139.1, 128.4, 127.7, 127.6, 85.5, 75.2, 68.0, 46.0 (brs), 35.7, 31.2, 21.3 (brs), 20.0, 19.3, 11.7 ppm;

HRMS (ESI): $C_{21}H_{35}NO_3Na$ $[M+Na]^+$ calculated: 372.2515, found: 372.2507;

R$_f$ = 0.4 (PE:MTBE 9:1, uv, vanillin);

$[\alpha]_D^{20}$ = +21.5 (*c* 0.9, $CHCl_3$).

Carbamate 30b

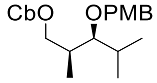

30b
$C_{22}H_{37}NO_4$
379.54

Using GP8 and PMB-protected primary alcohol **S2** (175 mg, 0.69 mmol, 1.0 equiv) gave carbamate **30b** (182 mg, 0.48 mmol, 70%) after purification by flash column chromatography (PE:MTBE 9:1) as a colorless oil.

^{1}H NMR (400 MHz, $CDCl_3$): δ = 7.30-7.27 (m, 2H), 6.88-6.85 (m, 2H), 4.50 (m_c, 2H), 4.11-3.80 (m, 7H), 3.15 (dd, J = 7.4, 3.5 Hz, 1H), 2.12 (m_c, 1H), 1.89 (m_c, 1H), 1.24-1.21 (m, 12H), 1.02 (d, J = 6.6 Hz, 3H), 0.97 (d, J = 6.9 Hz, 3H), 0.91 (d, J = 6.8 Hz, 3H) ppm;

^{13}C{^{1}H} NMR (101 MHz, $CDCl_3$): δ = 159.2, 155.8, 131.3, 129.4, 113.9, 85.2, 74.9, 68.0, 55.4, 45.9 (brs), 35.7, 31.2, 21.4 (brs), 20.0, 19.3, 11.6 ppm;

HRMS (ESI): $C_{22}H_{37}NO_4Na$ $[M+Na]^+$ calculated: 402.2620, found: 402.2618;

R$_f$ = 0.5 (PE:MTBE 4:1, uv, CAN);

$[\alpha]_D^{20}$ = +23.9 (*c* 1.1, $CHCl_3$).

Carbamate 33b

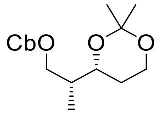

33b
$C_{16}H_{31}NO_4$
301.43

Using GP8 and primary alcohol **S7** (600 mg, 3.44 mmol, 1.0 equiv) gave carbamate **33b** (738 mg, 2.45 mmol, 71%) after purification by flash column chromatography (PE:MTBE 3:1) as a colorless oil.

^{1}H NMR (400 MHz, C_6D_6): δ = 4.28 (dd, J = 10.8, 5.6 Hz, 1H), 4.13 (dd, J = 10.8, 6.8 Hz, 1H), 4.04-3.60 (m, 5H), 1.82 (m_c, 1H), 1.62-1.48 (m, 1H), 1.46 (s, 3H), 1.30 (s, 3H), 1.11 (d, J = 6.6 Hz, 12H), 1.04 (d, J = 6.9 Hz, 3H), 0.88 (m_c, 1H) ppm;

^{13}C{^{1}H} NMR (101 MHz, C_6D_6): δ = 155.4, 98.3, 70.0, 66.8, 59.9, 45.9 (brs), 38.6, 30.3, 28.9, 21.2 (brs), 19.3, 12.5 ppm;

HRMS (ESI): $C_{16}H_{31}NO_4Na$ $[M+Na]^+$ calculated: 324.2151, found: 324.2144;

R$_f$ = 0.3 (PE:MTBE 3:1, vanillin);

$[\alpha]_D^{20}$ = –17.4 (*c* 1.3, $CHCl_3$).

Carbamate 34b

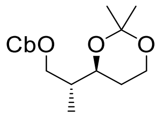

34b
$C_{16}H_{31}NO_4$
301.43

Using GP8 and primary alcohol **S10** (1.00 g, 5.74 mmol, 1.0 equiv) gave carbamate **34b** (0.85 g, 2.82 mmol, 49%) after purification by flash column chromatography (PE:MTBE 3:1) as a colorless oil.

^{1}H NMR (400 MHz, C_6D_6): δ = 4.43 (dd, J = 10.7, 4.6 Hz, 1H), 4.25 (dd, J = 10.7, 6.7 Hz, 1H), 4.09-3.54 (m, 5H), 1.87 (m$_c$, 1H), 1.46 (d, J = 0.5 Hz, 3H), 1.45-1.37 (m, 1H), 1.29 (d, J = 0.4 Hz, 3H), 1.11 (d, J = 6.7 Hz, 12H), 0.95 (m$_c$, 1H), 0.87 (d, J = 7.0 Hz, 3H) ppm;

^{13}C{^{1}H} NMR (101 MHz, C_6D_6): δ = 155.5, 98.4, 70.3, 66.3, 59.9, 46.0 (brs), 39.0, 30.2, 28.7, 21.2 (brs), 19.2, 13.0 ppm;

HRMS (ESI): $C_{16}H_{31}NO_4Na$ $[M+Na]^+$ calculated: 324.2151, found: 324.2150;

R$_f$ = 0.3 (PE:MTBE 3:1, vanillin);

$[\alpha]_D^{20}$ = +19.5 (c 0.7, $CHCl_3$).

Carbamate 43b

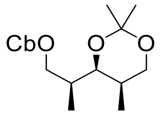

43b
$C_{17}H_{33}NO_4$
315.45

Using GP8 and primary alcohol **S8** (200 mg, 1.06 mmol, 1.0 equiv) gave carbamate **43b** (323 mg, 1.02 mmol, ≥ 95%) after purification by flash column chromatography (PE:EtOAc 10:1) as a colorless oil.

^{1}H NMR (400 MHz, C_6D_6): δ = 4.15-3.55 (brm, 2H), 4.13 (dd, J = 11.0, 3.7 Hz, 1H), 3.97 (dd, J = 11.0, 6.4 Hz, 1H), 3.79 (dd, J = 11.4, 2.7 Hz, 1H), 3.57 (dd, J = 9.7, 2.3 Hz, 1H), 3.40 (dd, J = 11.4, 1.7 Hz, 1H), 1.99-1.89 (m, 1H), 1.48 (s, 3H), 1.30 (s, 3H), 1.28-1.20 (m, 1H), 1.17 (d, J = 6.6 Hz, 3H), 1.11-1.08 (m, 15H) ppm;

^{13}C{^{1}H} NMR (101 MHz, C_6D_6): δ = 155.4, 98.8, 73.9, 66.9, 66.1, 45.9 (brs), 35.2, 30.7, 30.2, 21.1 (brs), 19.0, 14.9, 11.0 ppm;

HRMS (ESI): $C_{17}H_{33}NO_4Na$ $[M+Na]^+$ calculated: 338.2307, found: 338.2314;

R$_f$ = 0.2 (PE:EtOAc 9:1, vanillin);

$[\alpha]_D^{26}$ = +6.0 (c 1.0, $CHCl_3$).

Carbamate 46b

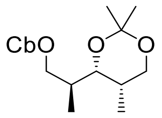

46b
$C_{17}H_{33}NO_4$
315.45

Using GP8 and primary alcohol **S11** (700 mg, 3.72 mmol, 1.0 equiv) gave carbamate **46b** (808 mg, 2.56 mmol, 69%) after purification by flash column chromatography (PE:EtOAc 10:1) as a colorless amorphous solid.

^{1}H NMR (400 MHz, C_6D_6): δ = 4.47 (dd, J = 10.5, 3.2 Hz, 1H), 4.37 (dd, J = 10.5, 6.4 Hz, 1H), 3.83 (brs, 2H), 3.77 (dd, J = 11.4, 2.6 Hz, 1H), 3.54 (dd, J = 10.1, 2.2 Hz, 1H), 3.42 (dd, J = 11.4, 1.6 Hz, 1H), 1.90-1.80 (m, 1H), 1.44 (s, 3H), 1.28 (s, 3H), 1.13-1.04 (m, 13H), 1.00 (d, J = 6.3 Hz, 3H), 0.80 (d, J = 6.8 Hz, 3H) ppm;

^{13}C{^{1}H} NMR (101 MHz, C_6D_6): δ = 155.6, 98.8, 72.7, 67.0, 66.7, 45.9 (brs), 35.4, 30.1, 29.9, 21.1 (brs), 19.0, 12.7, 10.5 ppm;

HRMS (ESI): $C_{17}H_{33}NO_4Na$ $[M+Na]^+$ calculated: 338.2307, found: 338.2310;

R*f* = 0.2 (PE:EtOAc 9:1, vanillin);

$[\alpha]_D^{27}$ = –21.6 (*c* 0.4, $CHCl_3$).

Carbamate 49b

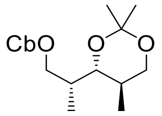

49b
$C_{17}H_{33}NO_4$
315.45

Using GP8 and primary alcohol **S9** (200 mg, 1.06 mmol, 1.0 equiv) gave carbamate **49b** (279 mg, 0.88 mmol, 83%) after purification by flash column chromatography (PE:EtOAc 10:1) as a colorless oil.

^{1}H NMR (400 MHz, C_6D_6): δ = 4.39 (dd, J = 10.6, 7.7 Hz, 1H), 4.22 (dd, J = 10.6, 6.7 Hz, 1H), 3.84 (brs, 2H), 3.59-3.55 (m, 2H), 3.30 (t, J = 11.1 Hz, 1H), 2.11-2.02 (m, 1H), 1.79-1.67 (m, 1H), 1.47 (s, 3H), 1.32 (s, 3H), 1.13 (d, J = 6.8 Hz, 12H), 0.96 (d, J = 7.0 Hz, 3H), 0.37 (d, J = 6.7 Hz, 3H) ppm;

^{13}C{^{1}H} NMR (101 MHz, C_6D_6): δ = 155.4, 98.3, 74.5, 67.3, 66.3, 45.8 (brs), 34.1, 30.7, 29.9, 21.1 (brs), 19.2, 12.1, 9.9 ppm;

HRMS (ESI): $C_{17}H_{33}NO_4Na$ $[M+Na]^+$ calculated: 338.2307, found: 338.2299;

R*f* = 0.3 (PE:EtOAc 9:1, vanillin);

$[\alpha]_D^{23}$ = –41.0 (*c* 1.0, $CHCl_3$).

Carbamate 52b

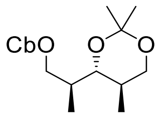

52b
$C_{17}H_{33}NO_4$
315.45

Using GP8 and primary alcohol **S12** (590 mg, 3.11 mmol, 1.0 equiv) gave carbamate **52b** (928 mg, 2.94 mmol, ≥ 95%) after purification by flash column chromatography (PE:EtOAc 10:1) as a colorless oil.

^{1}H NMR (400 MHz, C_6D_6): δ = 4.64 (dd, J = 11.0, 5.2 Hz, 1H), 4.16 (dd, J = 11.0, 8.0 Hz, 1H), 3.81-3.78 (m, 2H), 3.55 (dd, J = 11.4, 5.0 Hz, 1H), 3.26 (dd, J = 10.4, 2.4 Hz, 1H), 3.22 (t, J = 11.4 Hz, 1H), 2.27-2.18 (m, 1H), 1.99-1.88 (m, 1H), 1.45 (s, 3H), 1.24 (s, 3H), 1.20-0.95 (brm, 12H), 1.12 (d, J = 7.0 Hz, 3H), 0.52 (d, J = 6.7 Hz, 3H) ppm;

^{13}C{^{1}H} NMR (101 MHz, C_6D_6): δ = 155.3, 98.4, 78.3, 66.4, 65.9, 45.9 (brs), 34.4, 31.8, 29.9, 21.1 (brs), 19.2, 15.7, 12.7 ppm;

HRMS (ESI): $C_{17}H_{33}NO_4Na$ $[M+Na]^+$ calculated: 338.2307, found: 338.2308;

R*f* = 0.3 (PE:EtOAc 9:1, vanillin);

$[\alpha]_D^{25}$ = –28.0 (*c* 1.0, $CHCl_3$).

Carbamate 55b

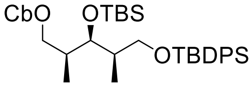

55b
$C_{36}H_{61}NO_4Si_2$
628.06

Using a modified version of GP8, primary alcohol **S14** (320 mg, 0.64 mmol, 1.0 equiv), Et_3N (1.5 equiv) and CbCl (1.5 equiv) gave carbamate **55b** (390 mg, 0.62 mmol, ≥ 95%) after purification by flash column chromatography (PE:MTBE 95:5) as a colorless oil.

^{1}H NMR (400 MHz, $CDCl_3$): δ = 7.67-7.63 (m, 4H), 7.44-7.34 (m, 6H), 4.08-3.72 (m, 5H), 3.57 (dd, J = 9.9, 6.5 Hz, 1H), 3.42 (dd, J = 9.9, 6.9 Hz, 1H), 2.04-1.98 (m, 1H), 1.90-1.84 (m, 1H), 1.19 (d, J = 6.8 Hz, 12H), 1.04 (s, 9H), 0.90 (d, J = 6.9 Hz, 3H), 0.88 (d, J = 6.8 Hz, 3H), 0.86 (s, 9H), 0.04 (s, 3H), −0.02 (s, 3H) ppm;

^{13}C{^{1}H} NMR (101 MHz, $CDCl_3$): δ = 155.9, 135.7, 134.0, 134.0, 129.69, 129.68, 127.8, 127.7, 73.2, 67.8, 66.9, 49.6, 45.8 (brs), 39.6, 37.7, 27.1, 27.0, 26.2, 21.0 (brs), 19.4, 18.5, 13.3, 12.7, −3.9, −4.0 ppm;

HRMS (ESI): $C_{36}H_{61}NO_4Si_2Na$ $[M+Na]^+$ calculated: 650.4037, found: 650.4007;

R*f* = 0.4 (PE:MTBE 9:1, vanillin);

$[\alpha]_D^{20}$ = −5.01 (*c* 1.0, $CHCl_3$).

Carbamate 58b

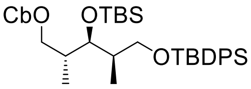

58b
$C_{36}H_{61}NO_4Si_2$
628.06

Using GP8 and primary alcohol **S15** (1.20 g, 2.40 mmol, 1.0 equiv) gave carbamate **58b** (1.46 g, 2.32 mmol, ≥ 95%) after purification by flash column chromatography (PE:MTBE 95:5) as a colorless oil.

^{1}H NMR (400 MHz, C_6D_6): δ = 7.82-7.78 (m, 4H), 7.29-7.22 (m, 6H), 4.41 (dd, *J* = 10.8, 4.4 Hz, 1H), 4.19 (dd, *J* = 10.8, 6.5 Hz, 1H), 3.98 (dd, *J* = 7.1, 2.2 Hz, 1H), 3.82 (m_c, 2H), 3.73 (dd, *J* = 10.0, 7.7 Hz, 1H), 3.62 (dd, *J* = 9.9, 6.6 Hz, 1H), 2.07-1.91 (m, 2H), 1.21 (s, 9H), 1.14 (brs, 12H), 0.99 (d, *J* = 7.0 Hz, 3H), 0.96 (s, 9H), 0.87 (d, *J* = 6.8 Hz, 3H), 0.17 (s, 3H), 0.08 (s, 3H) ppm;

^{13}C{^{1}H} NMR (101 MHz, C_6D_6): δ = 155.2, 136.11, 136.08, 134.34, 134.30, 130.1, 130.0, 128.1, 73.2, 67.4, 67.3, 45.9 (brs), 38.9, 38.3, 27.2, 26.4, 21.2 (brs), 19.5, 18.7, 14.9, 10.7, −3.8, −3.9 ppm;

HRMS (ESI): $C_{36}H_{61}NO_4Si_2Na$ $[M+Na]^+$ calculated: 650.4037, found: 650.4027;

R*f* = 0.2 (PE:MTBE 95:5, vanillin);

$[\alpha]_D^{20}$ = +1.17 (*c* 1.2, $CHCl_3$).

Carbamate 60b

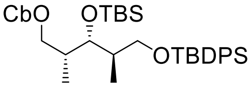

60b
$C_{36}H_{61}NO_4Si_2$
628.06

Using a modified version of GP8, primary alcohol **S13** (380 mg, 0.76 mmol, 1.0 equiv), Et_3N (1.5 equiv) and CbCl (1.5 equiv) gave carbamate **60b** (460 mg, 0.73 mmol, ≥ 95%) after purification by flash column chromatography (PE:MTBE 95:5) as a colorless oil.

^{1}H NMR (400 MHz, $CDCl_3$): δ = 7.68-7.26 (m, 4H), 7.44-7.32 (m, 6H), 4.15-3.67 (m, 5H), 3.64 (dd, *J* = 6.8, 2.3 Hz, 1H), 3.39 (dd, *J* = 9.8, 8.1 Hz, 1H), 2.00 (m_c, 1H), 1.96-1.87 (m, 1H), 1.19 (d, *J* = 6.8 Hz, 12H), 1.06 (s, 9H), 0.95 (d, *J* = 7.0 Hz, 3H), 0.88 (d, *J* = 6.8 Hz, 3H), 0.79 (s, 9H), −0.01 (s, 3H), −0.16 (s, 3H) ppm;

^{13}C{^{1}H} NMR (101 MHz, $CDCl_3$): δ = 155.7, 135.83, 135.82, 134.10, 134.08, 129.7, 129.6, 127.74, 127.69, 73.7, 68.1, 66.7, 46.0 (brs), 40.7, 36.0, 27.1, 26.2, 21.2 (brs), 19.4, 18.5, 14.4, 11.3, −3.9, −4.0 ppm;

HRMS (ESI): $C_{36}H_{61}NO_4Si_2Na$ [M+Na]$^+$ calculated: 650.4037, found: 650.4007;

R*f* = 0.4 (PE:MTBE 9:1, $KMnO_4$);

$[\alpha]_D^{20}$ = −0.71 (*c* 0.5, $CHCl_3$).

The enantiomer was prepared using ***ent*-S13**.

Carbamate 63b

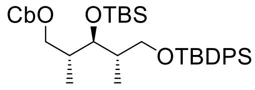

63b
$C_{36}H_{61}NO_4Si_2$
628.06

Using GP8 and primary alcohol **S16** (450 mg, 0.90 mmol, 1.0 equiv) gave carbamate **63b** (550 mg, 0.88 mmol, ≥ 95%) after purification by flash column chromatography (PE:MTBE 95:5) as a colorless oil.

^{1}H NMR (400 MHz, C_6D_6): δ = 7.81-7.78 (m, 4H), 7.26-7.23 (m, 6H), 4.47 (dd, *J* = 10.9, 5.0 Hz, 1H), 4.11 (dd, *J* = 10.9, 7.6 Hz, 1H), 3.98 (dd, *J* = 10.0, 5.8 Hz, 1H), 3.82 (brs, 2H), 3.67 (t, *J* = 4.9 Hz, 1H), 3.57 (dd, *J* = 10.0, 7.9 Hz, 1H), 2.25-2.10 (m, 2H), 1.20 (s, 9H), 1.17-1.08 (m, 15H), 1.02 (d, *J* = 7.0 Hz, 3H), 0.90 (s, 9H), 0.08 (s, 3H), −0.03 (s, 3H) ppm;

^{13}C{^{1}H} NMR (101 MHz, C_6D_6): δ = 155.3, 136.11, 136.10, 134.3, 129.99, 129.97, 128.1, 77.1, 67.3, 66.6, 45.9 (brs), 40.4, 37.3, 27.2, 26.3, 21.2 (brs), 19.5, 18.5, 15.7, 14.9, −3.89, −3.92 ppm;

HRMS (ESI): $C_{36}H_{61}NO_4Si_2Na$ [M+Na]$^+$ calculated: 650.4037, found: 650.4038;

R*f* = 0.2 (PE:MTBE 95:5, vanillin);

$[\alpha]_D^{20}$ = +3.10 (*c* 0.7, $CHCl_3$).

The enantiomer was prepared using ***ent*-S16**.

2-9. Substrate-controlled 1,2-metallate rearrangement–solvent screening

General Procedure 9 (GP9): TIB esters

Note: Since the scale of the performed reactions differs due to need and availability, this general procedure reports the relative molar quantities, equivalents and relative solvent volumes of a representative experiment, whereby the exact volumes/weights of the structure-building (or key) chemicals are listed in the corresponding individual procedure.

To a stirred solution of TIB ester (0.48 mmol, 1.5 equiv) and diamine (0.48 mmol, 1.5 equiv) in solvent 1 (2.4 mL, 0.2 M refers to TIB ester) at −78 °C was added *s*BuLi (1.3 M in hexanes, 0.45 mmol, 1.4 equiv). The reaction mixture was stirred for 5 h at that temperature before a solution of vinyl boronic ester (0.32 mmol, 1.0 equiv) in solvent 2 (0.64 mL, 0.5 M) was added. After stirring for further 3 h at −78 °C, the reaction mixture was warmed to 45 °C and stirred overnight. The reaction mixture was cooled to rt, sat. aq. NH_4Cl was added and the biphasic mixture was stirred for 15 min. The phases were separated, the organic layer was washed with sat. aq. NH_4Cl (3x) and the combined aqueous phases were extracted with MTBE (3x). The combined organic phases were dried over Na_2SO_4, concentrated *in vacuo* and the crude material was purified by a short flash column chromatography (to remove TIBOH).
The residue was dissolved in THF (1.6 mL, 0.2 M) and cooled to −20 °C. A premixed, ice-cooled solution of NaOH (2.0 M)/H_2O_2 (35%, 2/1 v/v, 2.7 mL, 0.12 M) was added dropwise. The reaction mixture was stirred at rt before being diluted with MTBE and quenched by the slow addition of sat. aq. $Na_2S_2O_3$ at 0 °C after TLC showed full conversion. The phases were separated and the aqueous phase was extracted with MTBE (3x). The combined organic layers were dried over Na_2SO_4 and concentrated *in vacuo*. The crude product was purified by flash column chromatography to afford allylic alcohol.

General Procedure 10 (GP10): Carbamates

Note: Since the scale of the performed reactions differs due to need and availability, this general procedure reports the relative molar quantities, equivalents and relative solvent volumes of a representative experiment, whereby the exact volumes/weights of the structure-building (or key) chemicals are listed in the corresponding individual procedure.

To a stirred solution of carbamate (0.48 mmol, 1.5 equiv) and diamine (0.48 mmol, 1.5 equiv) in solvent 1 (2.4 mL, 0.2 M refers to carbamate) at −78 °C was added *s*BuLi (1.3 M in hexanes, 0.45 mmol, 1.4 equiv). The reaction mixture was stirred for 5 h at that temperature before a solution of vinyl boronic ester (0.32 mmol, 1.0 equiv) in solvent 2 (0.64 mL, 0.5 M) was added. The reaction mixture was stirred for 3 h at −78 °C.
In parallel, magnesium turnings were activated (2x 1.0 M HCl, 2x H_2O, 2x acetone, drying under high vacuum). The required amount (0.64 mmol, 2.0 equiv) was dissolved in Et_2O (0.80 mL, 0.8 M refers to Mg turnings) and 1,2-dibromoethane (0.64 mmol, 2.0 equiv) was added under water bath cooling. The reaction mixture was stirred for 2 h at this temperature.
The biphasic $MgBr_2 \cdot OEt_2$ solution was added dropwise to the main reaction mixture, which was then stirred for another 30 min at −78 °C before being warmed to 45 °C and stirred

overnight. The reaction mixture was cooled to rt, sat. aq. NH_4Cl was added and the biphasic mixture was stirred for 15 min. The phases were separated, the organic layer was washed with sat. aq. NH_4Cl (3x) and the combined aqueous phases were extracted with MTBE (3x). The combined organic phases were dried over Na_2SO_4 and concentrated *in vacuo* and the crude material was purified by a short flash column chromatography (to remove excess of the carbamate).
The residue was dissolved in THF (1.6 mL, 0.2 M) and cooled to −20 °C. A premixed, ice-cooled solution of NaOH (2.0 M)/H_2O_2 (35%, 2/1 v/v, 2.7 mL, 0.12 M) was added dropwise. The reaction mixture was stirred at rt before being diluted with MTBE and quenched by the slow addition of sat. aq. $Na_2S_2O_3$ at 0 °C after TLC showed full conversion. The phases were separated and the aqueous phase was extracted with MTBE (3x). The combined organic layers were dried over Na_2SO_4 and concentrated *in vacuo*. The crude product was purified by flash column chromatography to afford allylic alcohol.

Table 1. Solvent screening for the synthesis of allylic alcohols **22a/b**.

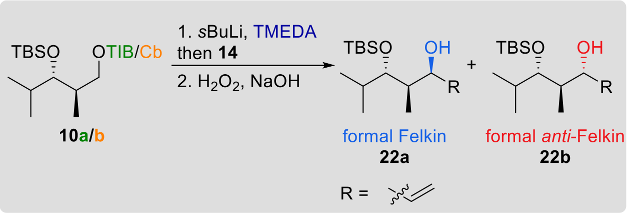

entry	DG	solvent 1 for deprotonation	solvent 2 for addition of **14**	main product	yield	dr
1	TIB	Et_2O	Et_2O	**22a**	50%	2:1
2	TIB	Et_2O	THF	**22a**	47%	6:1
3	TIB	THF	THF		-	
4	TIB	CPME	CPME		traces	
5	TIB	MTBE	MTBE	**22a**	60%	5:1
6	TIB	PhMe	THF		-	
7	Cb	Et_2O	Et_2O	**22b**	11%	1:2
8	Cb	Et_2O	THF		traces	
9	Cb	THF	THF		-	
10	Cb	CPME	CPME		traces	
11	Cb	MTBE	MTBE	**22b**	9%	1:2
12	Cb	PhMe	THF		traces	

[a] General conditions: 1. TIB ester (1.5 equiv), diamine (1.5 equiv), *s*BuLi (1.4 equiv), solvent 1, −78 °C, 5 h then vinyl boronic ester (1.0 equiv), solvent 2, −78 °C, 3 h then 45 °C, o/n or carbamate (1.5 equiv), diamine (1.5 equiv), *s*BuLi (1.4 equiv), solvent 1, −78 °C, 5 h then vinyl boronic ester (1.0 equiv), solvent 2, −78 °C, 3 h then $MgBr_2·OEt_2$ (2.0 equiv), −78 °C, 30 min then 45 °C, o/n. 2. H_2O_2, NaOH, THF, −20 °C to rt.

In the following experiments Et_2O was always used as solvent 1 and solvent 2.

2-10. Substrate-controlled 1,2-metallate rearrangement of vinyl boronates

(*R*)-Allylic alcohol 7

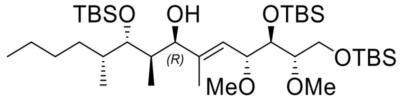

(*R*)-7
$C_{37}H_{80}O_6Si_3$
705.30

According to GP9, TIB ester **5a** (1.43 g, 2.75 mmol, 1.5 equiv),[3] vinyl boronic ester **6** (1.00 g, 1.84 mmol, 1.0 equiv)[3] and TMEDA gave allylic alcohol **(*R*)-7** (1.10 g, 1.56 mmol, 85% o2s, dr ≥ 19:1) after purification by flash column chromatography (PE:MTBE 95:5) as a colorless oil.

^{1}H NMR (400 MHz, C_6D_6): δ = 5.96 (m_c, 1H), 4.58 (brs, 1H), 4.43 (dd, J = 9.8, 2.5 Hz, 1H), 4.26 (dd, J = 7.7, 2.5 Hz, 1H), 4.03 (dd, J = 11.2, 2.3 Hz, 1H), 3.90 (dd, J = 11.2, 5.0 Hz, 1H), 3.68 (m_c, 1H), 3.34 (s, 3H), 3.24 (m_c, 1H), 3.19 (s, 3H), 2.58 (d, J = 2.3 Hz, 1H), 1.92 (m_c, 1H), 1.78-1.70 (m, 4H), 1.58-1.49 (m, 1H), 1.36-1.17 (m, 5H), 1.10 (s, 9H), 1.05-0.97 (m, 24H), 0.93 (t, J = 7.0 Hz, 3H), 0.37 (s, 3H), 0.28 (s, 3H), 0.16 (s, 3H), 0.15 (s, 3H), 0.12 (s, 3H), 0.10 (s, 3H) ppm;

^{13}C{^{1}H} NMR (101 MHz, C_6D_6): δ = 142.0, 121.9, 83.4, 80.6, 78.3, 74.7, 74.6, 62.7, 57.9, 55.6, 39.0, 37.6, 34.4, 30.4, 26.52, 26.45, 26.2, 23.5, 18.8, 18.7, 18.6, 15.3, 14.8, 14.4, 11.6, −3.4, −3.6, −3.8, −4.6, −4.97, −5.04 ppm;

HRMS (ESI): $C_{37}H_{80}O_6Si_3Na$ $[M+Na]^+$ calculated: 727.5160, found: 727.5166;

R*f* = 0.3 (PE:MTBE 95:5, vanillin);

$[\alpha]_D^{20}$ = −10.9 (*c* 0.7, $CHCl_3$).

Analytical data are in accordance with the literature.[3]

(*S*)-Allylic alcohol 15a

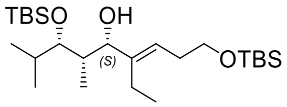

(*S*)-15a
$C_{25}H_{54}O_3Si_2$
458.87

According to GP9, TIB ester **9a** (210 mg, 0.44 mmol, 1.5 equiv), vinyl boronic ester **11** (100 mg, 0.29 mmol, 1.0 equiv) and TMEDA gave allylic alcohol **(*S*)-15a** (119 mg, contaminated with 3% ketone, 0.25 mmol, 86% o2s, dr 25:1) after purification by flash column chromatography (PE:MTBE 95:5) as a colorless oil.

^{1}H NMR (400 MHz, C_6D_6): δ = 5.58 (m_c, 1H), 4.09 (brs, 1H), 3.68-3.62 (m, 3H), 2.40-2.30 (m, 2H), 2.15-2.02 (m, 1H), 1.94-1.81 (m, 3H), 1.26 (d, J = 3.2 Hz, 1H), 1.07 (d, J = 7.0 Hz, 3H),

1.03 (s, 9H), 1.00-0.96 (m, 15H), 0.93 (d, J = 6.8 Hz, 3H), 0.16 (s, 3H), 0.10 (s, 3H), 0.09 (s, 6H) ppm;

^{13}C{^{1}H} NMR (101 MHz, C_6D_6): δ = 144.5, 121.7, 79.6, 76.9, 63.5, 38.8, 33.1, 31.8, 26.5, 26.2, 21.8, 19.9, 18.8, 18.6, 17.8, 14.4, 9.8, −3.3, −3.6, −5.097, −5.105 ppm;

HRMS (ESI): $C_{25}H_{54}O_3Si_2Na$ $[M+Na]^+$ calculated: 481.3509, found: 481.3514;

R$_f$ = 0.3 (PE:MTBE 95:5, vanillin);

$[\alpha]_D^{20}$ = +9.66 (c 1.1, $CHCl_3$).

(*R*)-Allylic alcohol 15b

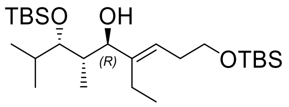

(*R*)-15b
$C_{25}H_{54}O_3Si_2$
458.87

According to GP10, carbamate **9b** (165 mg, 0.44 mmol, 1.5 equiv), vinyl boronic ester **11** (100 mg, 0.29 mmol, 1.0 equiv) and TMEDA gave allylic alcohol **(*R*)-15b** (57 mg, contaminated with 5% ketone, 0.12 mmol, 41% o2s, dr 4:1) after purification by flash column chromatography (PE:MTBE 95:5) as a colorless oil.

^{1}H NMR (400 MHz, C_6D_6): δ = 5.45 (m_c, 1H), 3.99-3.95 (m, 2H), 3.59 (t, J = 6.7 Hz, 2H), 2.31 (q, J = 6.8 Hz, 2H), 2.16-2.05 (m, 2H), 1.94-1.82 (m, 2H), 1.66 (brs, 1H), 1.10 (t, J = 7.6 Hz, 3H), 1.05-1.03 (m, 12H), 1.00 (s, 9H), 0.92 (d, J = 6.9 Hz, 3H), 0.85 (d, J = 7.0 Hz, 3H), 0.21 (s, 3H), 0.14 (s, 3H), 0.08 (s, 6H) ppm;

^{13}C{^{1}H} NMR (101 MHz, C_6D_6): δ = 144.8, 125.3, 80.1, 77.5, 63.2, 39.9, 32.7, 31.7, 26.5, 26.2, 20.34, 20.32, 20.29, 18.8, 18.5, 15.3, 12.0, −3.71, −3.74, −5.13, −5.14 ppm;

HRMS (ESI): $C_{25}H_{54}O_3Si_2Na$ $[M+Na]^+$ calculated: 481.3509, found: 481.3506;

R$_f$ = 0.3 (PE:MTBE 95:5, vanillin);

$[\alpha]_D^{20}$ = −3.80 (c 1.0, $CHCl_3$).

(*S*)-Allylic alcohol 16a

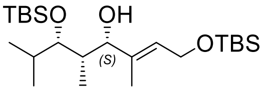

(*S*)-16a
$C_{23}H_{50}O_3Si_2$
430.82

According to GP9, TIB ester **9a** (802 mg, 1.68 mmol, 1.5 equiv), vinyl boronic ester **12** (350 mg, 1.12 mmol, 1.0 equiv) and TMEDA gave allylic alcohol **(*S*)-16a** (379 mg, 0.88 mmol, 79% o2s, dr 10:1) after purification by flash column chromatography (PE:MTBE 9:1) as a colorless oil.

^{1}H NMR (400 MHz, C_6D_6): δ = 5.80 (m_c, 1H), 4.26 (m_c, 2H), 3.87 (brs, 1H), 3.59 (t, J = 4.0 Hz, 1H), 1.85-1.77 (m, 2H), 1.46 (s, 3H), 1.18-1.16 (m, 1H), 1.08 (d, J = 6.9 Hz, 3H), 1.02 (s, 9H), 1.01 (s, 9H), 0.95 (d, J = 6.9 Hz, 3H), 0.89 (d, J = 6.9 Hz, 3H), 0.15 (s, 3H), 0.12 (s, 6H), 0.10 (s, 3H) ppm;

^{13}C{^{1}H} NMR (101 MHz, C_6D_6): δ = 138.0, 126.5, 78.6, 78.5, 60.1, 38.3, 33.4, 26.5, 26.2, 19.7, 18.8, 18.5, 18.1, 13.0, 10.1, −3.4, −3.5, −4.9, −5.0 ppm;

HRMS (ESI): $C_{23}H_{50}O_3Si_2Na$ $[M+Na]^+$ calculated: 453.3196, found: 453.3200;

R*f* = 0.3 (PE:MTBE 9:1, vanillin);

$[\alpha]_D^{20}$ = +4.77 (*c* 1.0, $CHCl_3$).

(*R*)-Allylic alcohol 16b

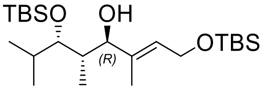

(*R*)-16b
$C_{23}H_{50}O_3Si_2$
430.82

According to GP10, carbamate **9b** (165 mg, 0.44 mmol, 1.5 equiv), vinyl boronic ester **12** (91 mg, 0.29 mmol, 1.0 equiv) and TMEDA gave allylic alcohol **(*R*)-16b** (48 mg, 0.11 mmol, 38% o2s, dr 4:1) after purification by flash column chromatography (PE:MTBE 95:5) as a colorless oil.

^{1}H NMR (400 MHz, C_6D_6): δ = 5.68 (m_c, 1H), 4.28-4.21 (m, 2H), 3.94-3.87 (m, 2H), 1.89-1.77 (m, 2H), 1.70 (brs, 1H), 1.57 (d, J = 1.2 Hz, 3H), 1.02-1.01 (m, 12H), 1.00 (s, 9H), 0.89 (d, J = 6.8 Hz, 3H), 0.79 (d, J = 7.1 Hz, 3H), 0.15 (s, 3H), 0.12-0.10 (m, 9H) ppm;

^{13}C{^{1}H} NMR (101 MHz, C_6D_6): δ = 138.0, 128.6, 79.8, 77.5, 60.1, 39.2, 32.6, 26.4, 26.2, 20.3, 20.2, 18.7, 18.5, 11.7, 10.8, −3.7, −3.8, −4.941, −4.945 ppm;

HRMS (ESI): $C_{23}H_{50}O_3Si_2Na$ $[M+Na]^+$ calculated: 453.3196, found: 453.3199;

R*f* = 0.3 (PE:MTBE 9:1, vanillin);

$[\alpha]_D^{20}$ = −8.63 (*c* 1.0, $CHCl_3$).

(*R*)-Allylic alcohol 17a

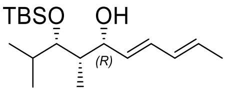

(*R*)-17a
$C_{18}H_{36}O_2Si$
312.57

According to GP9, TIB ester **9a** (210 mg, 0.44 mmol, 1.5 equiv), vinyl boronic ester **13** (57 mg, 0.29 mmol, 1.0 equiv) and TMEDA gave allylic alcohol **(*R*)-17a** (41 mg, 0.13 mmol, 45% o2s, dr 13:1) after purification by flash column chromatography (PE:MTBE 95:5) as a colorless oil.
According to GP10, carbamate **9b** (165 mg, 0.44 mmol, 1.5 equiv), vinyl boronic ester **13** (57 mg, 0.29 mmol, 1.0 equiv) and TMEDA gave allylic alcohol **(*R*)-17a** (4 mg, 0.01 mmol, 3% o2s, dr 8:1) after purification by flash column chromatography (PE:MTBE 95:5) as a colorless oil.

Analytical data are given for allylic alcohol **(*R*)-17a** obtained by the reaction of TIB ester **9a** and TMEDA (dr 13:1).

^{1}H NMR (400 MHz, C_6D_6): δ = 6.30-6.23 (m, 1H), 6.08-6.01 (m, 1H), 5.59-5.50 (m, 2H), 4.08 (brs, 1H), 3.63 (t, *J* = 4.1 Hz, 1H), 1.86-1.78 (m, 1H), 1.74-1.68 (m, 1H), 1.59 (dd, *J* = 6.6, 1.3 Hz, 3H), 1.22-1.20 (m, 1H), 1.10 (d, *J* = 6.9 Hz, 3H), 1.02 (s, 9H), 0.92 (d, *J* = 6.9 Hz, 3H), 0.88 (d, *J* = 6.8 Hz, 3H), 0.13 (s, 3H), −0.08 (s, 3H) ppm;

^{13}C{^{1}H} NMR (101 MHz, C_6D_6): δ = 133.7, 131.7, 131.3, 129.0, 78.4, 74.9, 41.8, 33.5, 26.4, 19.3, 18.7, 18.3, 18.2, 10.5, −3.4, −3.7 ppm;

HRMS (ESI): $C_{18}H_{36}O_2SiNa$ $[M+Na]^+$ calculated: 335.2382, found: 335.2375;

R*f* = 0.3 (PE:MTBE 95:5, uv, vanillin);

$[\alpha]_D^{20}$ = +21.8 (*c* 1.0, $CHCl_3$).

(*R*)-Allylic alcohol 18a

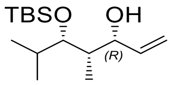

(*R*)-18a
$C_{15}H_{32}O_2Si$
272.50

According to GP9, TIB ester **9a** (232 mg, 0.48 mmol, 1.5 equiv), vinyl boronic ester **14** (50 mg, 0.32 mmol, 1.0 equiv) and TMEDA gave allylic alcohol **(*R*)-18a** (43 mg, 0.16 mmol, 50% o2s, dr 2:1) after purification by flash column chromatography (PE:MTBE 95:5) as a colorless oil.
According to GP9, TIB ester **9a** (232 mg, 0.48 mmol, 1.5 equiv), vinyl boronic ester **14** (50 mg, 0.32 mmol, 1.0 equiv) and (+)-sparteine gave allylic alcohol **(*R*)-18a** (63 mg, 0.23 mmol, 72% o2s, dr ≥ 19:1) after purification by flash column chromatography (PE:MTBE 95:5) as a colorless oil.
According to GP9, TIB ester **9a** (232 mg, 0.48 mmol, 1.5 equiv), vinyl boronic ester **14** (50 mg, 0.32 mmol, 1.0 equiv) and (−)-sparteine gave allylic alcohol **(*R*)-18a** (13 mg, 0.05 mmol, 16% o2s, dr 3:1) after purification by flash column chromatography (PE:MTBE 95:5) as a colorless oil.
According to GP10, carbamate **9b** (182 mg, 0.48 mmol, 1.5 equiv), vinyl boronic ester **14** (50 mg, 0.32 mmol, 1.0 equiv) and (+)-sparteine gave allylic alcohol **(*R*)-18a** (11 mg, 0.04 mmol, 13% o2s, dr 19:1) after purification by flash column chromatography (PE:MTBE 95:5) as a colorless oil.

Analytical data are given for allylic alcohol **(*R*)-18a** obtained by the reaction of TIB ester **9a** and (+)-sparteine (dr ≥ 19:1).[6]

^{1}H NMR (400 MHz, C_6D_6): δ = 5.74 (ddd, *J* = 17.4, 10.5, 5.7 Hz, 1H), 5.22 (dt, *J* = 17.2, 1.6 Hz, 1H), 5.03 (dt, *J* = 10.6, 1.6 Hz, 1H), 4.01 (brs, 1H), 3.59 (t, *J* = 4.1 Hz, 1H), 1.85-1.75 (m, 2H), 1.70-1.60 (m, 1H), 1.05 (d, *J* = 7.0 Hz, 3H), 1.01 (s, 9H), 0.91 (d, *J* = 6.9 Hz, 3H), 0.87 (d, *J* = 7.1 Hz, 3H), 0.10 (s, 3H), 0.07 (s, 3H) ppm;

^{13}C{^{1}H} NMR (101 MHz, C_6D_6): δ = 141.2, 114.6, 78.5, 75.0, 41.3, 33.3, 26.4, 19.4, 18.7, 18.2, 10.2, −3.4, −3.7 ppm;

HRMS (ESI): $C_{15}H_{32}O_2SiNa$ $[M+Na]^+$ calculated: 295.2069, found: 295.2077;

R*f* = 0.2 (PE:MTBE 9:1, vanillin);

$[\alpha]_D^{20}$ = +17.4 (*c* 0.8, $CHCl_3$).

[6] Stereochemistry was assigned by usual induction of (+)-sparteine (matched-case TIB ester).[16, 17]

(*S*)-Allylic alcohol 18b

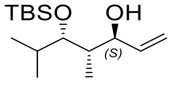

(*S*)-18b
$C_{15}H_{32}O_2Si$
272.50

According to GP10, carbamate **9b** (182 mg, 0.48 mmol, 1.5 equiv), vinyl boronic ester **14** (50 mg, 0.32 mmol, 1.0 equiv) and TMEDA gave allylic alcohol **(*S*)-18b** (26 mg, 0.10 mmol, 31% o2s, dr 8:1) after purification by flash column chromatography (PE:MTBE 95:5) as a colorless oil.
According to GP10, carbamate **9b** (182 mg, 0.48 mmol, 1.5 equiv), vinyl boronic ester **14** (50 mg, 0.32 mmol, 1.0 equiv) and (−)-sparteine gave allylic alcohol **(*S*)-18b** (10 mg, 0.04 mmol, 13% o2s, dr 19:1) after purification by flash column chromatography (PE:MTBE 95:5) as a colorless oil.

Analytical data are given for allylic alcohol **(*S*)-18b** obtained by the reaction of carbamate **10** and (−)-sparteine (dr 19:1).[7]

^{1}H NMR (400 MHz, C_6D_6): δ = 5.71 (m$_c$, 1H), 5.15 (m$_c$, 1H), 4.99 (m$_c$, 1H), 3.91-3.85 (m, 2H), 1.78 (m$_c$, 1H), 1.69-1.61 (m, 2H), 1.02 (s, 9H), 0.99 (d, J = 6.7 Hz, 3H), 0.87-0.84 (m, 6H), 0.15 (s, 3H), 0.11 (s, 3H) ppm;

^{13}C{^{1}H} NMR (101 MHz, C_6D_6): δ = 141.4, 115.5, 77.2, 75.5, 42.1, 32.6, 26.5, 20.1, 19.9, 18.7, 11.6, −3.75, −3.79 ppm;

HRMS (ESI): $C_{15}H_{32}O_2SiNa$ $[M+Na]^+$ calculated: 295.2066, found: 295.2069;

R$_f$ = 0.2 (PE:MTBE 95:5, vanillin);

$[\alpha]_D^{20}$ = −10.2 (c 0.9, $CHCl_3$).

(*R*)-Allylic alcohol 19a

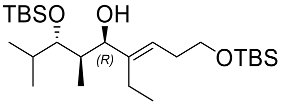

(*R*)-19a
$C_{25}H_{54}O_3Si_2$
458.87

According to GP9, TIB ester **10a** (210 mg, 0.44 mmol, 1.5 equiv), vinyl boronic ester **11** (100 mg, 0.29 mmol, 1.0 equiv) and TMEDA gave allylic alcohol **(*R*)-19a** (107 mg, 0.23 mmol, 79% o2s, dr ≥ 19:1) after purification by flash column chromatography (PE:MTBE 98:2 → 9:1) as a colorless oil.

^{1}H NMR (400 MHz, C_6D_6): δ = 5.76 (m$_c$, 1H), 4.62 (brs, 1H), 3.66 (t, J = 7.0 Hz, 2H), 3.51 (t, J = 5.1 Hz, 1H), 2.54 (d, J = 2.0 Hz, 1H), 2.48-2.35 (m, 2H), 2.16 (m$_c$, 1H), 1.91-1.77 (m, 3H),

[7] Stereochemistry was assigned by usual induction of (−)-sparteine (matched-case carbamate).[16, 17]

1.06-0.99 (m, 21H), 0.96 (d, J = 7.1 Hz, 3H), 0.93 (d, J = 6.8 Hz, 3H), 0.90 (d, J = 7.0 Hz, 3H), 0.12 (s, 3H), 0.09 (s, 6H), 0.07 (s, 3H) ppm;

^{13}C{^{1}H} NMR (101 MHz, C_6D_6): δ = 143.8, 120.6, 81.9, 72.2, 63.6, 38.1, 32.1, 31.8, 26.5, 26.2, 22.1, 20.4, 18.7, 18.6, 18.3, 14.2, 11.3, −3.46, −3.55, −5.1 ppm;

HRMS (ESI): $C_{25}H_{54}O_3Si_2Na$ $[M+Na]^+$ calculated: 481.3509, found: 481.3511;

R$_f$ = 0.3 (PE:MTBE 95:5, vanillin);

$[\alpha]_D^{20}$ = +3.19 (c 1.2, CHCl$_3$).

(*S*)-Allylic alcohol 19b

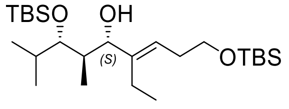

(*S*)-19b
$C_{25}H_{54}O_3Si_2$
458.87

According to GP10, carbamate **10b** (165 mg, 0.44 mmol, 1.5 equiv), vinyl boronic ester **11** (100 mg, 0.29 mmol, 1.0 equiv) and TMEDA gave allylic alcohol **(*S*)-19b** (71 mg, 0.15 mmol, 52% o2s, dr 2:1) after purification by flash column chromatography (PE:MTBE 20:1) as a colorless oil.

^{1}H NMR (400 MHz, C_6D_6): δ = 5.43 (t, J = 7.2 Hz, 1H), 3.98 (d, J = 9.8 Hz, 1H), 3.71 (t, J = 4.4 Hz, 1H), 3.61 (t, J = 6.7 Hz, 2H), 2.49 (s, 1H), 2.34 (q, J = 6.7 Hz, 2H), 2.29-2.11 (m, 2H), 2.07-1.98 (m, 1H), 1.94-1.84 (m, 1H), 1.19 (t, J = 7.6 Hz, 3H), 1.01-0.98 (m, 6H), 1.00 (s, 9H), 0.99 (s, 9H), 0.85 (d, J = 7.1 Hz, 3H), 0.16 (s, 3H), 0.09 (s, 6H), 0.07 (s, 3H) ppm;

^{13}C{^{1}H} NMR (101 MHz, C_6D_6): δ = 144.3, 125.1, 81.7, 80.8, 63.3, 41.3, 32.8, 31.7, 26.3, 26.2, 20.6, 20.4, 18.7, 18.6, 18.5, 15.8, 15.3, −3.9, −4.2, −5.1 ppm;

HRMS (ESI): $C_{25}H_{54}O_3Si_2Na$ $[M+Na]^+$ calculated: 481.3509, found: 481.3511;

R$_f$ = 0.3 (PE:MTBE 95:5, vanillin);

$[\alpha]_D^{31}$ = ±0 (c 1.0, CHCl$_3$).

(*R*)-Allylic alcohol 20a

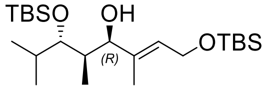

(*R*)-20a
$C_{23}H_{50}O_3Si_2$
430.82

According to GP9, TIB ester **10a** (802 mg, 1.68 mmol, 1.5 equiv), vinyl boronic ester **12** (350 mg, 1.12 mmol, 1.0 equiv) and TMEDA gave allylic alcohol **(*R*)-20a** (333 mg,

0.77 mmol, 69% o2s, dr ≥ 19:1) after purification by flash column chromatography (PE:MTBE 98:2 → 9:1) as a colorless oil.

^{1}H NMR (400 MHz, C_6D_6): δ = 6.08 (m_c, 1H), 4.43 (brs, 1H), 4.34 (m_c, 2H), 3.43 (m_c, 1H), 2.75 (d, J = 2.0 Hz, 1H), 1.87-1.75 (m, 2H), 1.51 (s, 3H), 1.01 (s, 9H), 0.98 (s, 9H), 0.94 (d, J = 7.1 Hz, 3H), 0.91 (d, J = 6.8 Hz, 3H), 0.86 (d, J = 6.9 Hz, 3H), 0.13 (s, 6H), 0.07 (s, 3H), 0.04 (s, 3H) ppm;

^{13}C{^{1}H} NMR (101 MHz, C_6D_6): δ = 137.3, 124.8, 82.1, 74.1, 60.4, 37.9, 32.1, 26.4, 26.2, 20.3, 18.69, 18.65, 18.6, 14.1, 11.4, −3.5, −3.6, −4.85, −4.88 ppm;

HRMS (ESI): $C_{23}H_{50}O_3Si_2Na$ $[M+Na]^+$ calculated: 453.3196, found: 453.3198;

R$_f$ = 0.3 (PE:MTBE 9:1, vanillin);

$[\alpha]_D^{20}$ = +10.1 (c 1.3, $CHCl_3$).

(*S*)-Allylic alcohol 20b

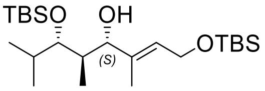

(*S*)-20b
$C_{23}H_{50}O_3Si_2$
430.82

According to GP10, carbamate **10b** (165 mg, 0.44 mmol, 1.5 equiv), vinyl boronic ester **12** (91 mg, 0.29 mmol, 1.0 equiv) and TMEDA gave allylic alcohol **(*S*)-20b** (76 mg, 0.18 mmol, 60% o2s, dr 2:1) after purification by flash column chromatography (PE:MTBE 20:1) as a colorless oil.

^{1}H NMR (400 MHz, C_6D_6): δ = 5.68-5.66 (m, 1H), 4.26-4.24 (m, 2H), 3.90 (d, J = 9.6 Hz, 1H), 3.69 (t, J = 4.4 Hz, 1H), 2.36 (d, J = 1.5 Hz, 1H), 2.01-1.92 (m, 1H), 1.90-1.82 (m 1H), 1.65 (d, J = 1.1 Hz, 3H), 1.01 (s, 9H), 0.99-0.96 (m, 6H), 0.98 (s, 9H), 0.80 (d, J = 7.2 Hz, 3H), 0.13 (s, 3H), 0.11 (s, 3H), 0.10 (s, 3H), 0.06 (s, 3H) ppm;

^{13}C{^{1}H} NMR (101 MHz, C_6D_6): δ = 137.6, 128.5, 81.0, 80.3, 60.1, 40.7, 32.4, 26.2, 26.2, 20.7, 18.6, 18.5, 18.5, 15.0, 11.0, −3.9, −4.2, −4.9, −5.0 ppm;

HRMS (ESI): $C_{23}H_{50}O_3Si_2Na$ $[M+Na]^+$ calculated: 453.3196, found: 453.3198;

R$_f$ = 0.3 (PE:MTBE 9:1, vanillin);

$[\alpha]_D^{31}$ = +3.0 (c 1.0, $CHCl_3$).

(*S*)-Allylic alcohol 21a

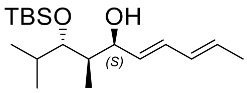

(*S*)-21a
$C_{18}H_{36}O_2Si$
312.57

According to GP9, TIB ester **10a** (210 mg, 0.44 mmol, 1.5 equiv), vinyl boronic ester **13** (57 mg, 0.29 mmol, 1.0 equiv) and TMEDA gave allylic alcohol **(*S*)-21a** (62 mg, 0.20 mmol, 69% o2s, dr 5:1) after purification by flash column chromatography (PE:MTBE 98:2 → 10:1) as a colorless oil.

^{1}H NMR (400 MHz, C_6D_6): δ = 6.48 (m_c, 1H), 6.14-6.07 (m, 1H), 5.59-5.54 (m, 2H), 4.73 (brs, 1H), 3.43 (t, J = 4.9 Hz, 1H), 2.56 (d, J = 2.3 Hz, 1H), 1.86-1.77 (m, 1H), 1.69-1.58 (m, 4H), 0.99-0.97 (m, 12H), 0.89 (d, J = 6.8 Hz, 3H), 0.85 (d, J = 6.9 Hz, 3H), 0.11 (s, 3H), 0.05 (s, 3H) ppm;

^{13}C{^{1}H} NMR (101 MHz, C_6D_6): δ = 134.0, 132.0, 130.1, 128.3, 81.9, 71.1, 41.1, 32.1, 26.4, 20.2, 18.7, 18.5, 18.2, 11.8, −3.55, −3.62 ppm;

HRMS (ESI): $C_{18}H_{36}O_2SiNa$ $[M+Na]^+$ calculated: 335.2382, found: 335.2387;

R*f* = 0.3 (PE:MTBE 95:5, uv, vanillin);

$[\alpha]_D^{20}$ = −0.92 (*c* 0.8, $CHCl_3$).

(*R*)-Allylic alcohol 21b

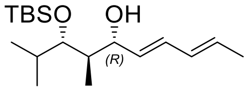

(*R*)-21b
$C_{18}H_{36}O_2Si$
312.57

According to GP10, carbamate **10b** (165 mg, 0.44 mmol, 1.5 equiv), vinyl boronic ester **13** (57 mg, 0.29 mmol, 1.0 equiv) and TMEDA gave allylic alcohol **(*R*)-21b** (35 mg, 0.11 mmol, 38% o2s, dr 2:1) after purification by flash column chromatography (PE:MTBE 95:5) as a colorless oil.

^{1}H NMR (400 MHz, C_6D_6): δ = 6.30 (dd, J = 15.1, 10.4 Hz, 1H), 6.10-6.03 (m, 1H), 5.63-5.52 (m, 2H), 4.09 (t, J = 7.6 Hz, 1H), 3.64 (dd, J = 5.0, 4.3 Hz, 1H), 2.21 (s, 1H), 1.92-1.82 (m, 2H) 1.59 (dd, J = 6.6, 1.4 Hz, 3H), 1.00 (s, 9H), 0.99-0.97 (m, 3H), 0.95 (d, J = 6.8 Hz, 3H), 0.86 (d, J = 7.1 Hz, 3H), 0.13 (s, 3H), 0.07 (s, 3H) ppm;

^{13}C{^{1}H} NMR (101 MHz, C_6D_6): δ = 133.1, 131.9, 131.6, 129.0, 80.2, 75.0, 44.1, 32.3, 26.3, 20.6, 18.6, 18.3, 18.2, 14.4, −3.8, −4.1 ppm;

HRMS (ESI): $C_{18}H_{36}O_2SiNa$ $[M+Na]^+$ calculated: 335.2382, found: 335.2387;

R$_f$ = 0.3 (PE:MTBE 95:5, uv, vanillin);

$[\alpha]_D^{31}$ = +8.7 (*c* 1.0, CHCl$_3$).

(*S*)-Allylic alcohol 22a

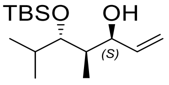

(*S*)-22a
$C_{15}H_{32}O_2Si$
272.50

According to GP9, TIB ester **10a** (143 mg, 0.30 mmol, 1.5 equiv), vinyl boronic ester **14** (31 mg, 0.20 mmol, 1.0 equiv) and TMEDA gave allylic alcohol **(*S*)-22a** (27 mg, 0.10 mmol, 50% o2s, dr 2:1) after purification by flash column chromatography (PE:MTBE 95:5) as a colorless oil.
According to GP10, carbamate **10b** (182 mg, 0.48 mmol, 1.5 equiv), vinyl boronic ester **14** (50 mg, 0.32 mmol, 1.0 equiv) and (−)-sparteine gave allylic alcohol **(*S*)-22a** (15 mg, 0.06 mmol, 19% o2s, dr 19:1) after purification by flash column chromatography (PE:MTBE 95:5) as a colorless oil.

Analytical data are given for allylic alcohol **(*S*)-22a** obtained by the reaction of carbamate **10b** and (−)-sparteine (dr 19:1).[8]

^{1}H NMR (400 MHz, C$_6$D$_6$): δ = 5.77 (m$_c$, 1H), 5.44 (dt, *J* = 17.3, 1.9 Hz, 1H), 5.12 (dt, *J* = 10.6, 1.9 Hz, 1H), 4.68-4.64 (m, 1H), 3.40 (t, *J* = 4.9 Hz, 1H), 1.84-1.73 (m, 1H), 1.65-1.57 (m, 1H), 0.97 (s, 9H), 0.94 (d, *J* = 7.1 Hz, 3H), 0.87 (d, *J* = 7.1 Hz, 3H), 0.82 (d, *J* = 6.9 Hz, 3H), 0.09 (s, 3H), 0.04 (s, 3H) ppm;

^{13}C{^{1}H} NMR (101 MHz, C$_6$D$_6$): δ = 141.2, 113.7, 81.9, 71.4, 40.4, 32.1, 26.4, 20.2, 18.7, 18.5, 11.5, −3.56, −3.64 ppm;

HRMS (ESI): $C_{15}H_{32}O_2SiNa$ [M+Na]$^+$ calculated: 295.2069, found: 295.2077;

R$_f$ = 0.2 (PE:MTBE 95:5, vanillin);

$[\alpha]_D^{20}$ = −3.0 (*c* 0.8, CHCl$_3$).

(*R*)-Allylic alcohol 22b

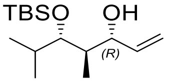

(*R*)-22b
$C_{15}H_{32}O_2Si$
272.50

According to GP10, carbamate **10b** (109 mg, 0.29 mmol, 1.5 equiv), vinyl boronic ester **14** (30 mg, 0.19 mmol, 1.0 equiv) and TMEDA gave allylic alcohol **(*S*)-22a** (1.5 mg) and

[8] Stereochemistry was assigned by usual induction of (−)-sparteine (matched-case carbamate).[16, 17]

allylic alcohol **(*R*)-22b** (3.5 mg, 5.0 mg in total, 0.02 mmol, 11% o2s, dr 2:1) after purification by flash column chromatography (PE:MTBE 95:5) as colorless oils.

Analytical data are given for the pure allylic alcohol **(*R*)-16d**.

^{1}H NMR (400 MHz, C_6D_6): δ = 5.84 (m$_c$, 1H), 5.27 (ddd, J = 17.1, 10.5, 4.1 Hz, 1H), 5.06 (m$_c$, 1H), 4.04 (t, J = 7.2 Hz, 1H), 3.57 (dd, J = 5.3, 4.2 Hz, 1H), 2.22 (brs, 1H), 1.88-1.78 (m, 2H), 0.98 (s, 9H), 0.95 (d, J = 6.7 Hz, 3H), 0.93 (d, J = 6.5 Hz, 3H), 0.83 (d, J = 5.9 Hz, 3H), 0.09 (s, 3H), 0.05 (s, 3H) ppm;

^{13}C{^{1}H} NMR (101 MHz, C_6D_6): δ = 140.5, 115.1, 80.4, 75.3, 43.5, 32.4, 26.3, 20.5, 18.5, 18.2, 14.4, −3.8, −4.1 ppm;

HRMS (ESI): $C_{15}H_{32}O_2SiNa$ $[M+Na]^+$ calculated: 295.2069, found: 295.2066;

R*f* = 0.2 (PE:MTBE 95:5, vanillin);

$[\alpha]_D^{20}$ = +8.3 (*c* 0.8, $CHCl_3$).

(*S*)-Allylic alcohol 27a

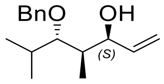

(*S*)-27a
$C_{16}H_{24}O_2$
248.37

According to GP9, TIB ester **25a** (208 mg, 0.45 mmol, 1.5 equiv), vinyl boronic ester **14** (52 mg, 0.29 mmol, 1.0 equiv) and TMEDA gave allylic alcohol **(*S*)-27a** (33 mg, 0.13 mmol, 44% o2s, dr ≥ 19:1) after purification by flash column chromatography (cyclohexane:MTBE 95:5) as a colorless oil.
According to GP10, carbamate **25b** (158 mg, 0.45 mmol, 1.5 equiv), vinyl boronic ester **14** (52 mg, 0.29 mmol, 1.0 equiv) and TMEDA gave allylic alcohol **(*S*)-27a** (8 mg, 0.03 mmol, 10% o2s, dr 2:1) after purification by flash column chromatography (PE:MTBE 94:6) as a colorless oil.

Analytical data are given for allylic alcohol **(*S*)-27a** obtained by the reaction of TIB ester **25a** and TMEDA (dr ≥ 19:1).

^{1}H NMR (400 MHz, C_6D_6): δ = 7.30-7.28 (m, 2H), 7.15-7.07 (m, 3H), 5.76 (ddd, J = 17.2, 10.6, 4.1 Hz, 1H), 5.41 (dt, J = 17.2, 2.0 Hz, 1H), 5.11 (dt, J = 10.6, 2.0 Hz, 1H), 4.60 (s, 1H), 4.45 (dd, J = 15.2, 11.1 Hz, 2H), 3.05 (t, J = 5.8 Hz, 1H), 2.57 (d, J = 2.7 Hz, 1H), 1.81 (m$_c$, 1H), 1.72 (m$_c$, 1H), 0.92 (d, J = 6.7 Hz, 3H), 0.90 (d, J = 7.1 Hz, 3H) 0.84 (d, J = 6.9 Hz, 3H) ppm;

^{13}C{^{1}H} NMR (101 MHz, C_6D_6): δ = 141.1, 139.1, 128.7, 127.8 (covered by C_6D_6), 127.7, 113.8, 88.7, 75.9, 71.6, 40.1, 31.1, 20.4, 17.6, 11.3 ppm;

^{13}C{^{1}H} NMR (101 MHz, $CDCl_3$): δ = 140.1, 138.4, 128.6, 127.9, 127.8, 114.3, 89.7, 75.9, 72.2, 39.5, 31.1, 20.3, 18.3, 11.8 ppm;

HRMS (ESI): $C_{16}H_{24}O_2Na$ $[M+Na]^+$ calculated: 271.1674, found: 271.1666;

$\mathbf{R}_f$ = 0.3 (PE:MTBE 95:5, uv, vanillin).

$[\alpha]_D^{20}$ = −3.00 (*c* 0.5, $CHCl_3$).

(*S*)-Allylic alcohol 28a

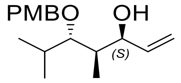

(*S*)-28a
$C_{17}H_{26}O_3$
278.39

According to GP9, TIB ester **26a** (217 mg, 0.45 mmol, 1.5 equiv), vinyl boronic ester **14** (52 mg, 0.29 mmol, 1.0 equiv) and TMEDA gave allylic alcohol **(*S*)-28a** (45 mg, 0.16 mmol, 54% o2s, dr ≥ 19:1) after purification by flash column chromatography (cyclohexane:MTBE 95:5) as a colorless oil.
According to GP10, carbamate **26b** (171 mg, 0.45 mmol, 1.5 equiv), vinyl boronic ester **14** (52 mg, 0.29 mmol, 1.0 equiv) and TMEDA gave allylic alcohol **(*S*)-28a** (3 mg, 0.01 mmol, 4% o2s, dr 19:1) after purification by flash column chromatography (PE:MTBE 94:6) as a colorless oil.

Analytical data are given for allylic alcohol **(*S*)-28a** obtained by the reaction of TIB ester **26a** and TMEDA (dr ≥ 19:1).

^{1}H NMR (400 MHz, C_6D_6): δ = 7.21 (d, J = 8.5 Hz, 2H), 6.75 (d, J = 8.5 Hz, 2H), 5.88 (ddd, J = 17.1, 10.6, 4.1 Hz, 1H), 5.46 (d, J = 17.2 Hz, 1H), 5.13 (d, J = 10.6 Hz, 1H), 4.62 (s, 1H), 4.41 (q, J = 10.6 Hz, 2H), 3.27 (s, 3H), 3.05 (t, J = 5.7 Hz, 1H), 2.92 (brs, 1H), 1.82 (dq, J = 13.3, 6.6 Hz, 1H), 1.73 (m_c, 1H), 0.93 (dd, J = 6.8, 4.5 Hz, 6H), 0.83 (d, J = 6.8 Hz, 3H) ppm;

^{13}C{^{1}H} NMR (101 MHz, C_6D_6): δ = 159.8, 141.0, 131.1, 129.4, 114.2, 113.8, 88.9, 75.8, 71.7, 54.8, 40.0, 31.2, 20.4, 18.0, 11.6 ppm;

HRMS (ESI): $C_{17}H_{26}O_3Na$ $[M+Na]^+$ calculated: 301.1780, found: 301.1780;

$\mathbf{R}_f$ = 0.4 (PE:MTBE 94:6, uv, vanillin);

$[\alpha]_D^{20}$ = +4.00 (*c* 0.8, $CHCl_3$).

(*R*)-Allylic alcohol 31a

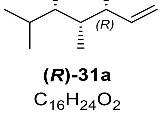

(*R*)-31a
$C_{16}H_{24}O_2$
248.37

According to GP9, TIB ester **29a** (141 mg, 312 µmol, 1.5 equiv), vinyl boronic ester **14** (32 mg, 208 µmol, 1.0 equiv) and TMEDA gave allylic alcohol **(*R*)-31a** (29 mg,

117 µmol, 56% o2s, dr 1.4:1) after purification by flash column chromatography (PE:MTBE 7:1) as a colorless oil.

^{1}H NMR (400 MHz, C_6D_6): δ = 7.34-7.32 (m, 2H), 7.21-7.15 (m, 2H), 7.12-7.08 (m, 1H), 5.76 (m_c, 1H), 5.32 (m_c, 1H), 5.07 (m_c, 1H), 4.43 (m_c, 2H), 4.14 (m_c, 1H), 3.12 (dd, J = 6.7, 3.8 Hz, 1H), 2.16 (brs, 1H), 1.87 (m_c, 1H), 1.73-1.67 (m, 1H), 1.06 (d, J = 7.0 Hz, 3H), 0.94 (d, J = 6.6 Hz, 3H), 0.82 (d, J = 7.0 Hz, 3H) ppm;

^{13}C{^{1}H} NMR (101 MHz, C_6D_6): δ = 140.9, 139.3, 128.6, 127.8, 127.7, 114.6, 87.6, 75.6, 74.4, 40.7, 31.4, 19.7, 18.9, 8.4 ppm;

HRMS (ESI): $C_{16}H_{24}O_2Na$ $[M+Na]^+$ calculated: 271.1674, found: 271.1672;

R*f* = 0.2 (PE:MTBE 9:1, uv, vanillin).

(*S*)-Allylic alcohol 31b

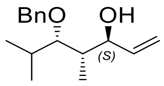

(*S*)-31b
$C_{16}H_{24}O_2$
248.37

According to GP10, carbamate **29b** (82 mg, 234 µmol, 1.5 equiv), vinyl boronic ester **14** (24 mg, 156 µmol, 1.0 equiv) and TMEDA gave allylic alcohol **(*S*)-31b** (5 mg, 20.1 µmol, 13% o2s, dr 4:1) after purification by flash column chromatography (PE:MTBE 7:1) as a colorless oil.

^{1}H NMR (400 MHz, C_6D_6): δ = 7.39-7.38 (m, 2H), 7.21-7.18 (m, 2H), 7.12-7.08 (m, 1H), 5.71 (m_c, 1H), 5.14 (m_c, 1H), 5.00 (m_c, 1H), 4.62 (m_c, 2H), 3.92 (brt, J = 7.3 Hz, 1H), 3.51 (dd, J = 8.3, 2.3 Hz, 1H), 1.88 (m_c, 1H), 1.72-1.66 (m, 1H), 1.53 (brs, 1H), 1.03 (d, J = 6.7 Hz, 3H), 0.95 (d, J = 7.0 Hz, 3H), 0.80 (d, J = 6.9 Hz, 3H) ppm;

^{13}C{^{1}H} NMR (101 MHz, C_6D_6): δ = 141.5, 140.0, 128.6, 127.6, 127.5, 115.6, 84.3, 75.7, 74.4, 40.8, 31.5, 20.0, 19.7, 10.8 ppm;

HRMS (ESI): $C_{16}H_{24}O_2Na$ $[M+Na]^+$ calculated: 271.1674, found: 271.1670;

R*f* = 0.2 (PE:MTBE 9:1, uv, vanillin);

$[\alpha]_D^{20}$ = +4.35 (*c* 0.2, $CHCl_3$).

(*R*)-Allylic alcohol 32a

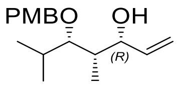

(*R*)-32a
$C_{17}H_{26}O_3$
278.39

According to GP9, TIB ester **30a** (125 mg, 259 µmol, 1.5 equiv), vinyl boronic ester **14** (27 mg, 175 µmol, 1.0 equiv) and TMEDA gave allylic alcohol **(*R*)-32a** (21 mg,

75.4 µmol, 43% o2s, dr 3:1) after purification by flash column chromatography (PE:MTBE 4:1) as a colorless oil.

^{1}H NMR (400 MHz, C_6D_6): δ = 7.29-7.25 (m, 2H), 6.80-6.77 (m, 2H), 5.78 (m_c, 1H), 5.39 (m_c, 1H), 5.10 (m_c, 1H), 4.41 (m_c, 2H), 4.21 (m_c, 1H), 3.29 (s, 3H), 3.14 (dd, J = 6.9, 3.6 Hz, 1H), 2.44 (brs, 1H), 1.88 (m_c, 1H), 1.73-1.66 (m, 1H), 1.06 (d, J = 7.0 Hz, 3H), 0.96 (d, J = 6.7 Hz, 3H), 0.82 (d, J = 6.9 Hz, 3H) ppm;

^{13}C{^{1}H} NMR (101 MHz, C_6D_6): δ = 159.8, 140.9, 131.2, 129.5, 114.5, 114.2, 87.8, 75.7, 74.2, 54.8, 40.5, 31.4, 19.7, 19.1, 8.0 ppm;

HRMS (ESI): $C_{17}H_{26}O_3Na$ $[M+Na]^+$ calculated: 301.1780, found: 301.1778;

R$_f$ = 0.2 (PE:MTBE 9:1, uv, vanillin);

$[\alpha]_D^{20}$ = –3.64 (c 1.1, $CHCl_3$).

(*S*)-Allylic alcohol 32b

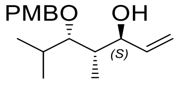

(*S*)-32b
$C_{17}H_{26}O_3$
278.39

According to GP10, carbamate **30b** (85 mg, 224 µmol, 1.5 equiv), vinyl boronic ester **14** (23 mg, 149 µmol, 1.0 equiv) and TMEDA gave allylic alcohol **(*S*)-32b** (7 mg, 25.1 µmol, 17% o2s, dr 10:1) after purification by flash column chromatography (PE:MTBE 4:1) as a colorless oil.

^{1}H NMR (400 MHz, C_6D_6): δ = 7.32-7.30 (m, 2H), 6.83-6.80 (m, 2H), 5.74 (m_c, 1H), 5.20 (m_c, 1H), 5.03 (m_c, 1H), 4.58 (m_c, 2H), 3.98 (brt, J = 7.1 Hz, 1H), 3.51 (dd, J = 8.1, 2.3 Hz, 1H), 3.30 (s, 3H), 1.89 (m_c, 1H), 1.76-1.66 (m, 2H), 1.05 (d, J = 6.7 Hz, 3H), 0.97 (d, J = 7.0 Hz, 3H), 0.81 (d, J = 6.8 Hz, 3H) ppm;

^{13}C{^{1}H} NMR (101 MHz, C_6D_6): δ = 159.7, 141.6, 132.0, 129.3, 115.5, 114.1, 84.2, 75.9, 74.1, 54.8, 40.8, 31.5, 20.1, 19.8, 11.0 ppm;

HRMS (ESI): $C_{17}H_{26}O_3Na$ $[M+Na]^+$ calculated: 301.1780, found: 301.1776;

R$_f$ = 0.2 (PE:MTBE 9:1, uv, vanillin);

$[\alpha]_D^{20}$ = +5.45 (c 0.6, $CHCl_3$).

(*R*)-Allylic alcohol 35a

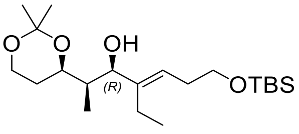

(*R*)-35a
$C_{21}H_{42}O_4Si$
386.65

According to GP9, TIB ester **33a** (625 mg, 1.54 mmol, 1.5 equiv), vinyl boronic ester **11** (350 mg, 1.03 mmol, 1.0 equiv) and TMEDA gave allylic alcohol **(*R*)-35a** (385 mg, 1.00 mmol, ≥ 95% o2s, dr 10:1) after purification by flash column chromatography (PE:MTBE 3:1) as a colorless oil.
According to GP10, carbamate **33b** (133 mg, 0.44 mmol, 1.5 equiv), vinyl boronic ester **11** (100 mg, 0.29 mmol, 1.0 equiv) and TMEDA gave allylic alcohol **(*R*)-35a** (88 mg, 0.23 mmol, 79% o2s, dr 7:1) after purification by flash column chromatography (PE:MTBE 5:1) as a colorless oil.

Analytical data are given for allylic alcohol **(*R*)-35a** obtained by the reaction of TIB ester **33a** and TMEDA (dr 10:1).

^{1}H NMR (400 MHz, C_6D_6): δ = 5.78 (m$_c$, 1H), 4.27 (brs, 1H), 3.82 (m$_c$, 1H), 3.68-3.62 (m, 4H), 2.47-2.35 (m, 2H), 2.33 (d, J = 2.0 Hz, 1H), 2.16-2.06 (m, 1H), 1.91-1.82 (m, 1H), 1.73-1.62 (m, 1H), 1.58-1.51 (m, 1H), 1.40 (s, 3H), 1.26 (s, 3H), 1.09 (d, J = 7.1 Hz, 3H), 1.04-1.00 (m, 12H), 0.84 (m$_c$, 1H), 0.10 (s, 6H) ppm;

^{13}C{^{1}H} NMR (101 MHz, C_6D_6): δ = 143.2, 121.6, 98.4, 76.7, 73.3, 63.5, 60.0, 40.6, 31.8, 30.2, 28.9, 26.2, 21.7, 19.4, 18.6, 14.3, 7.2, –5.1 ppm;

HRMS (ESI): $C_{21}H_{42}O_4SiNa$ [M+Na]$^+$ calculated: 409.2750, found: 409.2741;

R$_f$ = 0.3 (PE:MTBE 3:1, vanillin);

$[\alpha]_D^{20}$ = –13.9 (*c* 1.0, $CHCl_3$).

(*R*)-Allylic alcohol 36a

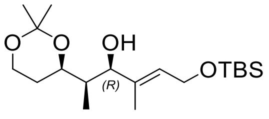

(*R*)-36a
$C_{19}H_{38}O_4Si$
358.59

According to GP9, TIB ester **33a** (180 mg, 0.44 mmol, 1.5 equiv), vinyl boronic ester **12** (91 mg, 0.29 mmol, 1.0 equiv) and TMEDA gave allylic alcohol **(*R*)-36a** (79 mg, contaminated with 6% TIBOH, 0.21 mmol, 72% o2s, dr 14:1) after purification by flash column chromatography (PE:MTBE 3:1) as a colorless oil.
According to GP10, carbamate **33b** (133 mg, 0.44 mmol, 1.5 equiv), vinyl boronic ester **12** (91 mg, 0.29 mmol, 1.0 equiv) and TMEDA gave allylic alcohol **(*R*)-36a** (88 mg,

0.25 mmol, 86% o2s, dr 5:1) after purification by flash column chromatography (PE:MTBE 4:1) as a colorless oil.

Analytical data are given for allylic alcohol **(*R*)-36a** obtained by the reaction of TIB ester **33a** and TMEDA (dr 14:1).

^{1}H NMR (400 MHz, C_6D_6): δ = 5.97 (m$_c$, 1H), 4.32 (m$_c$, 2H), 4.07 (d, J = 3.9 Hz, 1H), 3.75 (m$_c$, 1H), 3.64-3.61 (m, 2H), 1.70-1.59 (m, 1H), 1.52-1.45 (m, 4H), 1.40 (s, 3H), 1.29-1.27 (m, 4H), 1.05 (d, J = 7.0 Hz, 3H), 1.02 (s, 9H), 0.77 (m$_c$, 1H), 0.13 (s, 6H) ppm;

^{13}C{^{1}H} NMR (101 MHz, C_6D_6): δ = 136.9, 125.8, 98.4, 78.1, 72.6, 60.3, 60.0, 40.4, 30.2, 28.8, 26.2, 19.4, 18.6, 13.4, 7.3, –4.89, –4.90 ppm;

HRMS (ESI): $C_{19}H_{38}O_4SiNa$ $[M+Na]^+$ calculated: 381.2437, found: 381.2425;

R*f* = 0.3 (PE:MTBE 3:1, vanillin);

$[\alpha]_D^{20}$ = –6.98 (*c* 1.1, $CHCl_3$).

(*S*)-Allylic alcohol 37a

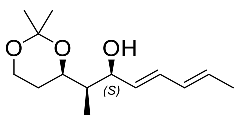

(*S*)-37a
$C_{14}H_{24}O_3$
240.34

According to GP9, TIB ester **33a** (180 mg, 0.44 mmol, 1.5 equiv), vinyl boronic ester **13** (57 mg, 0.29 mmol, 1.0 equiv) and TMEDA gave allylic alcohol **(*S*)-37a** (64 mg, contaminated with 6% TIBOH, 0.25 mmol, 86% o2s, dr 14:1) after purification by flash column chromatography (PE:MTBE 3:1) as a colorless oil.
According to GP10, carbamate **33b** (133 mg, 0.44 mmol, 1.5 equiv), vinyl boronic ester **13** (57 mg, 0.29 mmol, 1.0 equiv) and TMEDA gave allylic alcohol **(*S*)-37a** (40 mg, 0.17 mmol, 59% o2s, dr 3:1) after purification by flash column chromatography (PE:MTBE 3:1) as a colorless oil.

Analytical data are given for allylic alcohol **(*S*)-37a** obtained by the reaction of TIB ester **33a** and TMEDA (dr 14:1).

^{1}H NMR (400 MHz, C_6D_6): δ = 6.44-6.37 (m, 1H), 6.13-6.07 (m, 1H), 5.63-5.54 (m, 2H), 4.32-4.30 (m, 1H), 3.78 (m$_c$, 1H), 3.62-3.58 (m, 2H), 1.70-1.60 (m, 4H), 1.39 (s, 3H), 1.38-1.32 (m, 1H), 1.29-1.24 (m, 4H), 1.09 (d, J = 7.0 Hz, 3H), 0.77 (m$_c$, 1H) ppm;

^{13}C{^{1}H} NMR (101 MHz, C_6D_6): δ = 133.2, 131.9, 130.7, 128.7, 98.4, 75.0, 72.6, 60.0, 43.4, 30.2, 28.8, 19.4, 18.2, 7.6 ppm;

HRMS (ESI): $C_{14}H_{24}O_3Na$ $[M+Na]^+$ calculated: 263.1623, found: 263.1619;

R*f* = 0.3 (PE:MTBE 3:1, uv, vanillin);

$[\alpha]_D^{20}$ = –28.5 (*c* 0.9, $CHCl_3$).

(*S*)-Allylic alcohol 38a

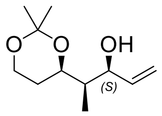

(*S*)-38a
$C_{11}H_{20}O_3$
200.28

According to GP9, TIB ester **33a** (180 mg, 0.44 mmol, 1.5 equiv), vinyl boronic ester **14** (45 mg, 0.29 mmol, 1.0 equiv) and TMEDA gave allylic alcohol **(*S*)-38a** (45 mg, 0.22 mmol, 76% o2s, dr 13:1) after purification by flash column chromatography (PE:MTBE 4:1) as a colorless oil.
According to GP10, carbamate **33b** (133 mg, 0.44 mmol, 1.5 equiv), vinyl boronic ester **14** (45 mg, 0.29 mmol, 1.0 equiv) and TMEDA gave allylic alcohol **(*S*)-38a** (14 mg, 0.07 mmol, 24% o2s, dr 2:1) after purification by flash column chromatography (PE:MTBE 3:1) as a colorless oil.
According to GP10, carbamate **33b** (133 mg, 0.44 mmol, 1.5 equiv), vinyl boronic ester **14** (45 mg, 0.29 mmol, 1.0 equiv) and (–)-sparteine gave allylic alcohol **(*S*)-38a** (19 mg, 0.09 mmol, 31% o2s, dr ≥ 19:1) after purification by flash column chromatography (PE:MTBE 3:1) as a colorless oil.

Analytical data are given for allylic alcohol **(*S*)-38a** obtained by the reaction of carbamate **33b** and (–)-sparteine (dr ≥ 19:1).

^{1}H NMR (400 MHz, C_6D_6): δ = 5.82-5.74 (m, 1H), 5.40 (m_c, 1H), 5.11 (m_c, 1H), 4.24 (brs, 1H), 3.74 (m_c, 1H), 3.62-3.55 (m, 2H), 2.39 (brs, 1H), 1.67-1.57 (m, 1H), 1.37 (s, 3H), 1.35-1.28 (m, 1H), 1.22 (s, 3H), 1.04 (d, J = 7.1 Hz, 3H), 0.76 (m_c, 1H) ppm;

^{13}C{^{1}H} NMR (101 MHz, C_6D_6): δ = 140.6, 114.2, 98.4, 75.2, 72.8, 59.9, 42.8, 30.1, 28.7, 19.3, 7.3 ppm;

HRMS (EI): $C_{10}H_{17}O_3$ $[M–CH_3]^+$ calculated: 185.1178, found: 185.1170;

R*f* = 0.3 (PE:MTBE 3:1, vanillin);

$[\alpha]_D^{20}$ = –34.9 (*c* 0.8, $CHCl_3$).

(*R*)-Allylic alcohol 38b

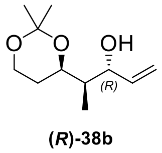

(*R*)-38b
$C_{11}H_{20}O_3$
200.28

According to GP10, carbamate **33b** (133 mg, 0.44 mmol, 1.5 equiv), vinyl boronic ester **14** (45 mg, 0.29 mmol, 1.0 equiv) and (+)-sparteine gave allylic alcohol **(*R*)-38b** (11 mg, 0.05 mmol, 17% o2s, dr 4:1) after purification by flash column chromatography (PE:MTBE 3:1) as a colorless oil.

^{1}H NMR (400 MHz, C_6D_6): δ = 5.83-5.75 (m, 1H), 5.40 (m_c, 1H), 5.12 (m_c, 1H), 4.11-4.07 (m, 1H), 3.98 (m_c, 1H), 3.60-3.54 (m, 2H), 3.04 (d, J = 5.4 Hz, 1H), 1.71-1.61 (m, 1H), 1.41-1.29 (m, 4H), 1.23 (s, 3H), 0.93 (d, J = 7.1 Hz, 3H), 0.62 (m_c, 1H) ppm;

^{13}C{^{1}H} NMR (101 MHz, C_6D_6): δ = 141.4, 114.7, 98.5, 75.7, 70.5, 60.0, 42.1, 30.2, 27.6, 19.2, 11.6 ppm;

HRMS (EI): $C_{10}H_{17}O_3$ $[M{-}CH_3]^+$ calculated: 185.1178, found: 185.1176;

R$_f$ = 0.3 (PE:MTBE 3:1, vanillin);

$[\alpha]_D^{20}$ = –4.84 (c 0.3, $CHCl_3$).

(*R*)-Allylic alcohol 39a

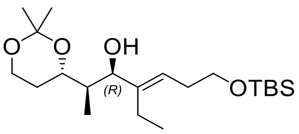

(*R*)-39a
$C_{21}H_{42}O_4Si$
386.65

According to GP9, TIB ester **34a** (625 mg, 1.54 mmol, 1.5 equiv), vinyl boronic ester **11** (350 mg, 1.03 mmol, 1.0 equiv) and TMEDA gave allylic alcohol **(*R*)-39a** (356 mg, contaminated with 12% TIBOH, 0.81 mmol, 79% o2s, dr ≥ 19:1) after purification by flash column chromatography (PE:MTBE 5:1) as a colorless oil.

^{1}H NMR (400 MHz, C_6D_6): δ = 5.73 (m_c, 1H), 4.60 (brs, 1H), 3.76 (m_c, 1H), 3.68-3.58 (m, 4H), 2.47-2.33 (m, 2H), 2.19-2.09 (m, 1H), 1.89-1.80 (m, 1H), 1.65-1.54 (m, 2H), 1.44 (s, 3H), 1.28 (s, 3H), 1.04-1.00 (m, 12H), 0.96-0.89 (m, 4H), 0.10 (s, 6H) ppm;

^{13}C{^{1}H} NMR (101 MHz, C_6D_6): δ = 143.7, 120.8, 98.6, 72.0, 71.7, 63.6, 60.0, 41.0, 31.8, 30.3, 29.7, 26.2, 22.0, 19.2, 18.6, 14.0, 9.3, –5.1 ppm;

HRMS (ESI): $C_{21}H_{42}O_4SiNa$ $[M+Na]^+$ calculated: 409.2750, found: 409.2742;

R$_f$ = 0.3 (PE:MTBE 3:1, vanillin);

$[\alpha]_D^{20}$ = +8.63 (c 1.2, $CHCl_3$).

(*S*)-Allylic alcohol 39b

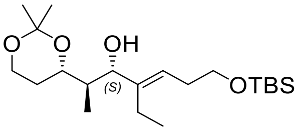

(*S*)-39b
$C_{21}H_{42}O_4Si$
386.65

According to GP10, carbamate **34b** (133 mg, 0.44 mmol, 1.5 equiv), vinyl boronic ester **11** (100 mg, 0.29 mmol, 1.0 equiv) and TMEDA gave allylic alcohol **(*S*)-39b** (69 mg, 0.18 mmol, 62% o2s, dr 1.3:1) after purification by flash column chromatography (PE:MTBE 5:1) as a colorless oil.

^{1}H NMR (400 MHz, C_6D_6): δ = 5.73 (m$_c$, 0.43H), 5.43 (m$_c$, 0.57H), 4.59 (brs, 0.38H), 3.99 (d, J = 8.9 Hz, 0.58H), 3.81-3.58 (m, 5H), 2.47-2.33 (m, 2H), 2.28-1.97 (m, 2H), 1.90-1.80 (m, 1H), 1.64-1.31 (m, 4H), 1.29-1.20 (m, 4.3H), 1.04-0.95 (m, 10.7H), 0.94-0.89 (m, 2.2H), 0.62 (d, J = 7.0 Hz, 1.8H), 0.094-0.087 (m, 6H) ppm;

^{13}C{^{1}H} NMR (101 MHz, C_6D_6): δ = 144.4, 125.1, 98.5, 84.0, 74.6, 63.4, 59.9, 42.0, 31.8, 30.1, 29.1, 26.2, 20.4, 19.3, 18.6, 15.3, 12.8, –5.10, –5.11 ppm;

HRMS (ESI): $C_{21}H_{42}O_4SiNa$ [M+Na]$^+$ calculated: 409.2750, found: 409.2744;

R*f* = 0.3 (PE:MTBE 3:1, vanillin).

(*R*)-Allylic alcohol 40a

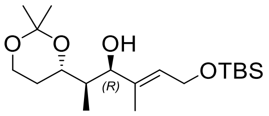

(*R*)-40a
$C_{19}H_{38}O_4Si$
358.59

According to GP9, TIB ester **34a** (180 mg, 0.44 mmol, 1.5 equiv), vinyl boronic ester **12** (91 mg, 0.29 mmol, 1.0 equiv) and TMEDA gave allylic alcohol **(*R*)-40a** (72 mg, contaminated with 10% TIBOH, 0.18 mmol, 62% o2s, dr ≥ 19:1) after purification by flash column chromatography (PE:MTBE 3:1) as a colorless oil.

^{1}H NMR (400 MHz, C_6D_6): δ = 5.97 (m$_c$, 1H), 4.36-4.31 (m, 3H), 3.72-3.57 (m, 3H), 1.63-1.50 (m, 2H), 1.48 (s, 3H), 1.44 (s, 3H), 1.26 (s, 3H), 1.02 (s, 9H), 0.91 (m$_c$, 1H), 0.84 (d, J = 7.1 Hz, 3H), 0.13 (s, 6H) ppm;

^{13}C{^{1}H} NMR (101 MHz, C_6D_6): δ = 137.5, 124.9, 98.6, 73.8, 71.7, 60.4, 60.0, 40.9, 30.3, 29.5, 26.2, 19.2, 18.6, 14.1, 9.3, –4.85, –4.87 ppm;

HRMS (ESI): $C_{19}H_{38}O_4SiNa$ [M+Na]$^+$ calculated: 381.2437, found: 381.2432;

R*f* = 0.3 (PE:MTBE 3:1, vanillin);

$[\alpha]_D^{20}$ = +16.8 (*c* 0.9, $CHCl_3$).

(*S*)-Allylic alcohol 40b

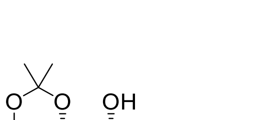

(*S*)-40b
$C_{19}H_{38}O_4Si$
358.59

According to GP10, carbamate **34b** (133 mg, 0.44 mmol, 1.5 equiv), vinyl boronic ester **12** (91 mg, 0.29 mmol, 1.0 equiv) and TMEDA gave allylic alcohol **(*S*)-40b** (67 mg, 0.19 mmol, 66% o2s, dr 2:1) after purification by flash column chromatography (PE:MTBE 3:1) as a colorless oil.

^{1}H NMR (400 MHz, C_6D_6): δ = 5.68 (m$_c$, 1H), 4.27 (m$_c$, 2H), 3.92 (d, J = 9.1 Hz, 1H), 3.72-3.69 (m, 1H), 3.60-3.55 (m, 2H), 2.07 (d, J = 3.4 Hz, 1H), 1.79 (m$_c$, 1H), 1.67 (d, J = 1.2 Hz, 3H), 1.61-1.51 (m, 1H), 1.37 (s, 3H), 1.25 (s, 3H), 1.01 (s, 9H), 0.97 (m$_c$, 1H), 0.55 (d, J = 7.0 Hz, 3H), 0.112 (s, 3H), 0.106 (s, 3H) ppm;

^{13}C{^{1}H} NMR (101 MHz, C_6D_6): δ = 137.7, 128.5, 98.5, 83.1, 74.5, 60.1, 59.9, 41.4, 30.1, 29.1, 26.2, 19.3, 18.5, 12.3, 11.3, –4.9, –5.0 ppm;

HRMS (ESI): $C_{19}H_{38}O_4SiNa$ [M+Na]$^+$ calculated: 381.2437, found: 381.2434;

R$_f$ = 0.3 (PE:MTBE 3:1, vanillin);

$[\alpha]_D^{20}$ = +15.7 (c 0.9, $CHCl_3$).

(*S*)-Allylic alcohol 41a

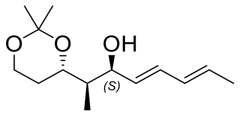

(*S*)-41a
$C_{14}H_{24}O_3$
240.34

According to GP9, TIB ester **34a** (180 mg, 0.44 mmol, 1.5 equiv), vinyl boronic ester **13** (57 mg, 0.29 mmol, 1.0 equiv) and TMEDA gave allylic alcohol **(*S*)-41a** (55 mg, contaminated with 13% TIBOH, 0.20 mmol, 69% o2s, dr 14:1) after purification by flash column chromatography (PE:MTBE 3:1) as a colorless oil.

^{1}H NMR (400 MHz, C_6D_6): δ = 6.47-6.40 (m, 1H), 6.16-6.09 (m, 1H), 5.64-5.54 (m, 2H), 4.48-4.45 (m, 1H), 3.67-3.50 (m, 3H), 1.75-1.67 (m, 1H), 1.61 (m$_c$, 3H), 1.49-1.41 (m, 1H), 1.39 (s, 3H), 1.23 (s, 3H), 0.87 (m$_c$, 1H), 0.70 (d, J = 7.2 Hz, 3H) ppm;

^{13}C{^{1}H} NMR (101 MHz, C_6D_6): δ = 132.4, 132.0, 130.8, 128.4, 98.5, 73.6, 72.3, 59.9, 44.0, 30.1, 29.8, 19.1, 18.2, 11.2 ppm;

HRMS (ESI): $C_{14}H_{24}O_3Na$ [M+Na]$^+$ calculated: 263.1623, found: 263.1613;

R$_f$ = 0.3 (PE:MTBE 3:1, uv, vanillin);

$[\alpha]_D^{20}$ = +8.10 (c 0.8, $CHCl_3$).

(*R*)-Allylic alcohol 41b

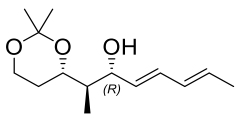

(*R*)-41b
$C_{14}H_{24}O_3$
240.34

According to GP10, carbamate **34b** (133 mg, 0.44 mmol, 1.5 equiv), vinyl boronic ester **13** (57 mg, 0.29 mmol, 1.0 equiv) and TMEDA gave allylic alcohol **(*R*)-41b** (36 mg,

0.15 mmol, 52% o2s, dr 4:1) after purification by flash column chromatography (PE:MTBE 3:1) as a colorless oil.

^{1}H NMR (400 MHz, C_6D_6): δ = 6.30-6.23 (m, 1H), 6.10-6.03 (m, 1H), 5.63-5.53 (m, 2H), 4.29 (t, J = 6.9 Hz, 1H), 3.66-3.54 (m, 3H), 2.67 (brs, 1H), 1.81 (m_c, 1H), 1.60 (m_c, 3H), 1.48-1.39 (m, 4H), 1.27 (s, 3H), 1.01 (m_c, 1H), 0.72 (d, J = 7.0 Hz, 3H) ppm;

^{13}C{^{1}H} NMR (101 MHz, C_6D_6): δ = 132.2, 132.0, 131.9, 128.9, 98.4, 74.7, 72.5, 60.0, 44.7, 30.3, 29.2, 19.3, 18.2, 10.8 ppm;

HRMS (ESI): $C_{14}H_{24}O_3Na$ $[M+Na]^+$ calculated: 263.1623, found: 263.1615;

R$_f$ = 0.3 (PE:MTBE 3:1, uv, vanillin);

$[\alpha]_D^{20}$ = +38.8 (c 3.2, $CHCl_3$).

(*S*)-Allylic alcohol 42a

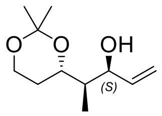

(*S*)-42a
$C_{11}H_{20}O_3$
200.28

According to GP9, TIB ester **34a** (180 mg, 0.44 mmol, 1.5 equiv), vinyl boronic ester **14** (45 mg, 0.29 mmol, 1.0 equiv) and TMEDA gave allylic alcohol **(*S*)-42a** (42 mg, 0.21 mmol, 72% o2s, dr ≥ 19:1) after purification by flash column chromatography (PE:MTBE 4:1) as a colorless oil.
According to GP10, carbamate **34b** (133 mg, 0.44 mmol, 1.5 equiv), vinyl boronic ester **14** (45 mg, 0.29 mmol, 1.0 equiv) and TMEDA gave allylic alcohol **(*S*)-42a** (7 mg, 0.03 mmol, 10% o2s, dr 2:1) after purification by flash column chromatography (PE:MTBE 3:1) as a colorless oil.
According to GP10, carbamate **34b** (133 mg, 0.44 mmol, 1.5 equiv), vinyl boronic ester **14** (45 mg, 0.29 mmol, 1.0 equiv) and (–)-sparteine gave allylic alcohol **(*S*)-42a** (9 mg, 0.04 mmol, 14% o2s, dr 1:1) after purification by flash column chromatography (PE:MTBE 3:1) as a colorless oil.

Analytical data are given for allylic alcohol **(*S*)-42a** obtained by the reaction of TIB ester **34a** and TMEDA (dr ≥ 19:1).

^{1}H NMR (400 MHz, C_6D_6): δ = 5.86-5.78 (m, 1H), 5.42 (m_c, 1H), 5.15 (m_c, 1H), 4.41 (m_c, 1H), 3.64-3.50 (m, 3H), 2.92 (d, J = 6.5 Hz, 1H), 1.65 (m_c, 1H), 1.47-1.38 (m, 1H), 1.36 (d, J = 0.6 Hz, 3H), 1.20 (d, J = 0.4 Hz, 3H), 0.86 (m_c, 1H), 0.67 (d, J = 7.2 Hz, 3H) ppm;

^{13}C{^{1}H} NMR (101 MHz, C_6D_6): δ = 139.9, 114.4, 98.4, 73.7, 72.2, 59.9, 43.4, 30.1, 29.7, 19.1, 10.9 ppm;

HRMS (EI): $C_{10}H_{17}O_3$ $[M–CH_3]^+$ calculated: 185.1178, found: 185.1176;

R$_f$ = 0.3 (PE:MTBE 3:1, vanillin);

$[\alpha]_D^{20}$ = –8.03 (*c* 0.9, $CHCl_3$).

(*R*)-Allylic alcohol 42b

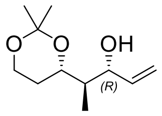

(*R*)-42b
$C_{11}H_{20}O_3$
200.28

According to GP10, carbamate **34b** (133 mg, 0.44 mmol, 1.5 equiv), vinyl boronic ester **14** (45 mg, 0.29 mmol, 1.0 equiv) and (+)-sparteine gave allylic alcohol **(*R*)-42b** (4 mg, 0.02 mmol, 7% o2s, dr ≥ 19:1) after purification by flash column chromatography (PE:MTBE 3:1) as a colorless oil.

^{1}H NMR (400 MHz, C_6D_6): δ = 5.83 (m_c, 1H), 5.26 (m_c, 1H), 5.08 (m_c, 1H), 4.18 (m_c, 1H), 3.63-3.52 (m, 3H), 2.52 (brs, 1H), 1.73 (m_c, 1H), 1.45-1.36 (m, 4H), 1.23 (s, 3H), 0.97 (m_c, 1H), 0.65 (d, *J* = 7.0 Hz, 3H) ppm;

^{13}C{^{1}H} NMR (101 MHz, C_6D_6): δ = 139.7, 115.5, 98.4, 75.6, 72.8, 59.9, 44.2, 30.2, 29.3, 19.3, 10.9 ppm;

HRMS (EI): $C_{10}H_{17}O_3$ $[M–CH_3]^+$ calculated: 185.1178, found: 185.1170;

R*f* = 0.3 (PE:MTBE 3:1, vanillin);

$[\alpha]_D^{20}$ = +23.7 (*c* 0.3, $CHCl_3$).

(*S*)-Allylic alcohol 44a

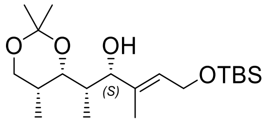

(*S*)-44a
$C_{20}H_{40}O_4Si$
372.62

According to GP9, TIB ester **43a** (185 mg, 0.44 mmol, 1.5 equiv), vinyl boronic ester **12** (91 mg, 0.29 mmol, 1.0 equiv) and TMEDA gave allylic alcohol **(*S*)-44a** (65 mg, 0.17 mmol, 59% o2s, dr ≥ 19:1) after purification by flash column chromatography (PE:MTBE 2:1) as a colorless oil.

^{1}H NMR (400 MHz, C_6D_6): δ = 5.80-5.76 (m, 1H), 4.31-4.20 (m, 2H), 3.95-3.89 (m, 2H), 3.76 (brs, 1H), 3.50 (dd, *J* = 11.4, 1.6 Hz, 1H), 1.80-1.72 (m, 1H), 1.51 (s, 3H), 1.44-1.39 (m, 1H), 1.35 (s, 3H), 1.31 (s, 3H), 1.13 (d, *J* = 6.8 Hz, 3H), 1.02 (d, *J* = 6.7 Hz, 3H), 1.02 (s, 9H), 0.89 (d, *J* = 4.0 Hz, 1H), 0.12 (s, 6H) ppm;

^{13}C{^{1}H} NMR (101 MHz, C_6D_6): δ = 138.0, 124.1, 98.9, 74.2, 73.3, 67.1, 60.2, 36.9, 30.3, 30.1, 26.2, 19.1, 18.6, 14.1, 11.3, 8.7, –4.9, –4.9 ppm;

HRMS (ESI): $C_{20}H_{40}O_4SiNa$ $[M+Na]^+$ calculated: 395.2594, found: 395.2601;

R$_f$ = 0.5 (PE:MTBE 2:1, vanillin);

$[\alpha]_D^{31}$ = +1.4 (*c* 0.7, $CHCl_3$).

(*R*)-Allylic alcohol 45a

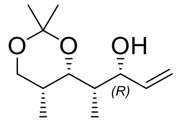

(*R*)-45a
$C_{12}H_{22}O_3$
214.31

According to GP9, TIB ester **43a** (185 mg, 0.44 mmol, 1.5 equiv), vinyl boronic ester **14** (45 mg, 0.29 mmol, 1.0 equiv) and TMEDA gave allylic alcohol **(*R*)-45a** (50 mg, 0.23 mmol, 79% o2s, dr ≥ 19:1) after purification by flash column chromatography (PE:MTBE 2:1) as a colorless oil.
According to GP10, carbamate **43b** (139 mg, 0.44 mmol, 1.5 equiv), vinyl boronic ester **14** (45 mg, 0.29 mmol, 1.0 equiv) and TMEDA gave allylic alcohol **(*R*)-45a** (14 mg, 0.07 mmol, 22% o2s, dr 3:1) after purification by flash column chromatography (PE:MTBE 2:1) as a colorless oil.

Analytical data are given for allylic alcohol **(*R*)-45a** obtained by the reaction of TIB ester **43a** and TMEDA (dr ≥ 19:1).

^{1}H NMR (400 MHz, C_6D_6): δ = 5.65 (ddd, *J* = 17.2, 10.6, 4.6 Hz, 1H), 5.10 (dt, *J* = 17.2, 1.7 Hz, 1H), 4.99 (dt, *J* = 10.6, 1.7 Hz, 1H), 4.00-3.99 (m, 1H), 3.90-3.86 (m, 2H), 3.47 (dd, *J* = 11.4, 1.7 Hz, 1H), 1.68-1.60 (m, 1H), 1.49 (s, 3H), 1.43-1.36 (m, 1H), 1.30 (s, 3H), 1.09 (d, *J* = 6.8 Hz, 3H), 1.05 (d, *J* = 6.8 Hz, 3H), 0.85 (d, *J* = 4.6 Hz, 1H) ppm;

^{13}C{^{1}H} NMR (101 MHz, C_6D_6): δ = 140.9, 113.7, 98.9, 73.8, 71.3, 67.1, 39.8, 30.4, 30.3, 19.1, 11.3, 9.2 ppm;

HRMS (ESI): $C_{12}H_{22}O_3Na$ $[M+Na]^+$ calculated: 237.1467, found: 237.1475;

R$_f$ = 0.2 (PE:MTBE 3:1, vanillin);

$[\alpha]_D^{26}$ = +40.9 (*c* 0.2, $CHCl_3$).

(*S*)-Allylic alcohol 47a

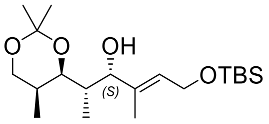

(*S*)-47a
$C_{20}H_{40}O_4Si$
372.62

According to GP9, TIB ester **46a** (139 mg, 0.33 mmol, 1.5 equiv), vinyl boronic ester **12** (69 mg, 0.22 mmol, 1.0 equiv) and TMEDA gave allylic alcohol **(*S*)-47a** (65 mg,

0.17 mmol, 79% o2s, dr ≥ 19:1) after purification by flash column chromatography (cyclohexane:EtOAc 10:1 → 5:1) as a colorless oil.
According to GP10, carbamate **46b** (139 mg, 0.44 mmol, 1.5 equiv), vinyl boronic ester **12** (91 mg, 0.29 mmol, 1.0 equiv) and TMEDA gave allylic alcohol **(*S*)-47a** (85 mg, 0.23 mmol, 77% o2s, dr 5:1) after purification by flash column chromatography (PE:MTBE 2:1) as a colorless oil.

Analytical data are given for allylic alcohol **(*S*)-47a** obtained by the reaction of TIB ester **46a** and TMEDA (dr ≥ 19:1).

^{1}H NMR (400 MHz, C_6D_6): δ = 5.89-5.85 (m, 1H), 4.33-4.25 (m, 3H), 3.87 (dd, J = 9.9, 2.2 Hz, 1H), 3.80 (dd, J = 11.4, 2.5 Hz, 1H), 3.44 (dd, J = 11.4, 1.5 Hz, 1H), 1.80-1.71 (m, 1H), 1.48 (s, 3H), 1.45 (s, 3H), 1.34 (s, 3H), 1.22 (d, J = 4.4 Hz, 1H), 1.15-1.09 (m, 1H), 1.06 (d, J = 6.2 Hz, 3H), 1.02 (s, 9H), 0.69 (d, J = 7.0 Hz, 3H), 0.12 (s, 6H) ppm;

^{13}C{^{1}H} NMR (101 MHz, C_6D_6): δ = 138.4, 124.4, 98.8, 73.2, 72.3, 67.2, 60.4, 37.5, 30.3, 30.3, 26.2, 19.2, 18.6, 14.3, 10.5, 7.7, –4.9, –4.9 ppm;

HRMS (ESI): $C_{20}H_{40}O_4SiNa$ [M+Na]$^+$ calculated: 395.2594, found: 395.2598;

R*f* = 0.7 (PE:MTBE 2:1, vanillin);

$[\alpha]_D^{31}$ = –7.5 (*c* 0.3, $CHCl_3$).

(*R*)-Allylic alcohol 48a

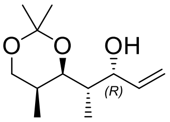

(*R*)-48a
$C_{12}H_{22}O_3$
214.31

According to GP9, TIB ester **46a** (185 mg, 0.44 mmol, 1.5 equiv), vinyl boronic ester **14** (45 mg, 0.29 mmol, 1.0 equiv) and TMEDA gave allylic alcohol **(*R*)-48a** (48 mg, 0.22 mmol, 76% o2s, dr ≥ 19:1) after purification by flash column chromatography (PE:MTBE 2:1) as a colorless oil.
According to GP10, carbamate **46b** (139 mg, 0.44 mmol, 1.5 equiv), vinyl boronic ester **14** (45 mg, 0.29 mmol, 1.0 equiv) and TMEDA gave allylic alcohol **(*R*)-48a** (14 mg, 0.07 mmol, 22% o2s, dr 1:1.3) after purification by flash column chromatography (PE:MTBE 2:1) as a colorless oil.

Analytical data are given for allylic alcohol **(*R*)-48a** obtained by the reaction of TIB ester **46a** and TMEDA (dr ≥ 19:1).

^{1}H NMR (400 MHz, C_6D_6): δ = 5.82 (ddd, J = 17.2, 10.6, 4.6 Hz, 1H), 5.38 (dt, J = 17.2, 2.0 Hz, 1H), 5.14 (dt, J = 10.6, 2.0 Hz, 1H), 4.35-4.33 (m, 1H), 3.74-3.69 (m, 2H), 3.36 (dd, J = 11.5, 1.4 Hz, 1H), 2.75 (d, J = 8.0 Hz, 1H), 1.91-1.82 (m, 1H), 1.36 (s, 3H), 1.23 (s, 3H), 1.01-0.94 (m, 4H), 0.53 (d, J = 7.2 Hz, 3H) ppm;

^{13}C{^{1}H} NMR (101 MHz, C_6D_6): δ = 139.7, 114.4, 98.7, 74.3, 73.8, 67.0, 39.4, 30.1, 30.0, 19.0, 10.5, 10.3 ppm;

HRMS (ESI): $C_{12}H_{22}O_3Na$ [M+Na]$^+$ calculated: 237.1467, found: 237.1471;

R$_f$ = 0.4 (PE:MTBE 3:1, vanillin);

$[\alpha]_D^{27}$ = +37.0 (*c* 0.5, $CHCl_3$).

Allylic alcohol 50

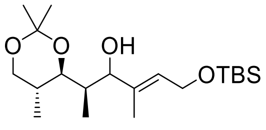

50
$C_{20}H_{40}O_4Si$
372.62

According to GP9, TIB ester **49a** (185 mg, 0.44 mmol, 1.5 equiv), vinyl boronic ester **12** (91 mg, 0.29 mmol, 1.0 equiv) and TMEDA gave allylic alcohol **50** (83 mg, 0.22 mmol, 76% o2s, dr 1.5:1) after purification by flash column chromatography (PE:MTBE 2:1) as a colorless oil.

Chemical shift values are given for the major diastereoisomer.

^{1}H NMR (400 MHz, C_6D_6): δ = 5.94-5.90 (m, 1H), 4.29-4.26 (m, 2H), 4.04 (t, *J* = 5.4 Hz, 1H), 3.84 (dd, *J* = 10.4, 1.9 Hz, 1H), 3.55 (dd, *J* = 11.4, 5.0 Hz, 1H), 3.31 (t, *J* = 11.4 Hz, 1H), 2.62 (d, *J* = 6.4 Hz, 1H), 1.83-1.70 (m, 2H), 1.46 (d, *J* = 0.7 Hz, 3H), 1.41 (s, 3H), 1.37 (s, 3H), 1.06 (d, *J* = 7.1 Hz, 3H), 1.00 (s, 9H), 0.33 (d, *J* = 6.7 Hz, 3H), 0.11 (s, 3H), 0.10 (s, 3H) ppm;

^{13}C{^{1}H} NMR (101 MHz, C_6D_6): δ = 137.9, 126.7, 98.4, 79.4, 74.7, 66.4, 60.1, 35.1, 30.7, 30.0, 26.2, 19.5, 18.5, 12.6, 12.0, 10.7, –4.9, –5.0 ppm;

HRMS (ESI): $C_{20}H_{40}O_4SiNa$ [M+Na]$^+$ calculated: 395.2594, found: 395.2590;

R$_f$ = 0.7 (PE:MTBE 2:1, vanillin).

(*R*)-Allylic alcohol 51b

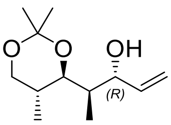

(*R*)-51b
$C_{12}H_{22}O_3$
214.31

According to GP9, TIB ester **49a** (185 mg, 0.44 mmol, 1.5 equiv), vinyl boronic ester **14** (45 mg, 0.29 mmol, 1.0 equiv) and TMEDA gave allylic alcohol **(*R*)-51b** (46 mg, 0.21 mmol, 72% o2s, dr 6:1) after purification by flash column chromatography (PE:MTBE 3:1) as a colorless oil.

According to GP9, TIB ester **49a** (215 mg, 0.52 mmol, 1.5 equiv), vinyl boronic ester **14** (53 mg, 0.34 mmol, 1.0 equiv) and (+)-sparteine surrogate gave allylic alcohol **(*R*)-51b** (22 mg, 0.10 mmol, 30% o2s, dr 7:1) after purification by flash column chromatography (PE:MTBE 3:1) as a colorless oil.
According to GP10, carbamate **49b** (139 mg, 0.44 mmol, 1.5 equiv), vinyl boronic ester **14** (45 mg, 0.29 mmol, 1.0 equiv) and TMEDA gave allylic alcohol **(*R*)-51b** (9 mg, 0.04 mmol, 14% o2s, dr 2:1) after purification by flash column chromatography (PE:MTBE 2:1) as a colorless oil.

Analytical data are given for allylic alcohol **(*R*)-51b** obtained by the reaction of TIB ester **49a** and (+)-sparteine surrogate (dr 7:1).

^{1}H NMR (400 MHz, C_6D_6): δ = 5.77 (ddd, *J* = 17.1, 10.4, 4.8 Hz, 1H), 5.42 (dt, *J* = 17.1, 1.9 Hz, 1H), 5.13 (ddd, *J* = 10.4, 1.9 Hz, 1H), 4.14-4.10 (m, 1H), 3.82 (dd, *J* = 10.4, 2.1 Hz, 1H), 3.52 (dd, *J* = 11.4, 4.9 Hz, 1H), 3.26 (t, *J* = 11.4 Hz, 1H), 2.76 (d, *J* = 6.0 Hz, 1H), 1.79-1.68 (m, 1H), 1.52-1.45 (m, 1H), 1.37 (s, 3H), 1.26 (s, 3H), 1.06 (d, *J* = 7.1 Hz, 3H), 0.28 (d, *J* = 6.7 Hz, 3H) ppm;

^{13}C{^{1}H} NMR (101 MHz, C_6D_6): δ = 141.8, 114.4, 98.3, 76.0, 75.2, 66.4, 37.8, 30.7, 30.0, 19.3, 11.9, 10.6 ppm;

HRMS (ESI): $C_{12}H_{22}O_3Na$ $[M+Na]^+$ calculated: 237.1467, found: 237.1478;

R*f* = 0.4 (PE:MTBE 3:1, vanillin);

$[\alpha]_D^{23}$ = –22.9 (*c* 0.4, $CHCl_3$).

(*R*)-Allylic alcohol 53b

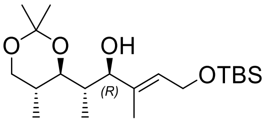

(*R*)-53b
$C_{20}H_{40}O_4Si$
372.62

According to GP9, TIB ester **52a** (139 mg, 0.33 mmol, 1.5 equiv), vinyl boronic ester **12** (69 mg, 0.22 mmol, 1.0 equiv) and TMEDA gave allylic alcohol **(*R*)-53b** (82 mg, 0.22 mmol, 75% o2s, dr 2:1) after purification by flash column chromatography (cyclohexane:EtOAc 10:1 → 5:1) as a colorless oil.
According to GP10, carbamate **52b** (139 mg, 0.44 mmol, 1.5 equiv), vinyl boronic ester **12** (91 mg, 0.29 mmol, 1.0 equiv) and TMEDA gave allylic alcohol **(*R*)-53b** (66 mg, 0.18 mmol, 60% o2s, dr 6:1) after purification by flash column chromatography (PE:MTBE 10:1 → 5:1) as a colorless oil.

Analytical data are given for allylic alcohol **(*R*)-53b** obtained by the reaction of carbamate **52b** and TMEDA (dr 6:1).

^{1}H NMR (400 MHz, C_6D_6): δ = 5.68-5.66 (m, 1H), 4.27-4.17 (m, 2H), 4.12 (dd, J = 8.8, 2.5 Hz, 1H), 3.61 (dd, J = 11.4, 5.0 Hz, 1H), 3.50 (dd, J = 10.1, 3.0 Hz, 1H), 3.24 (dd, J = 11.4, 10.0 Hz, 1H), 2.23 (d, J = 3.0 Hz, 1H), 2.23-2.12 (m, 1H), 1.99-1.91 (m, 1H), 1.60 (d, J = 1.1 Hz, 3H), 1.45 (s, 3H), 1.28 (s, 3H), 1.00 (s, 9H), 0.86 (d, J = 7.1 Hz, 3H), 0.61 (d, J = 6.6 Hz, 3H), 0.10 (s, 3H), 0.09 (s, 3H) ppm;

^{13}C{^{1}H} NMR (101 MHz, C_6D_6): δ = 138.1, 127.9, 98.7, 80.1, 79.6, 66.5, 60.1, 38.3, 33.2, 29.6, 26.2, 19.5, 18.5, 15.8, 13.7, 11.3, –5.0, –5.0 ppm;

HRMS (ESI): $C_{20}H_{40}O_4SiNa$ $[M+Na]^+$ calculated: 395.2594, found: 395.2596;

R*f* = 0.3 (PE:MTBE 5:1, vanillin);

$[\alpha]_D^{25}$ = –16.0 (*c* 1.0, $CHCl_3$).

(*S*)-Allylic alcohol 54b

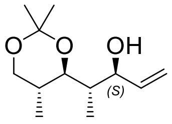

(*S*)-54b
$C_{12}H_{22}O_3$
214.31

According to GP9, TIB ester **52a** (185 mg, 0.44 mmol, 1.5 equiv), vinyl boronic ester **14** (45 mg, 0.29 mmol, 1.0 equiv) and TMEDA gave allylic alcohol **(*S*)-54b** (23 mg, 0.11 mmol) and allylic alcohol **(*R*)-54a** (24 mg, 0.11 mmol, 73% o2s in total, dr 1:1) after purification by flash column chromatography (PE:MTBE 5:1 → 3:1) as colorless oils.
According to GP10, carbamate **52b** (139 mg, 0.44 mmol, 1.5 equiv), vinyl boronic ester **14** (45 mg, 0.29 mmol, 1.0 equiv) and TMEDA gave allylic alcohol **(*S*)-54b** (12 mg, 0.06 mmol, 19% o2s, dr ≥ 19:1) after purification by flash column chromatography (PE:MTBE 5:1 → 2:1) as a colorless oil.

Analytical data for allylic alcohol **(*S*)-54b**:

^{1}H NMR (400 MHz, C_6D_6): δ = 5.95 (ddd, J = 17.2, 10.5, 5.8 Hz, 1H), 5.29 (dt, J = 17.2, 1.8 Hz, 1H), 5.05 (dt, J = 10.5, 1.8 Hz, 1H), 4.23 (t, J = 5.6 Hz, 1H), 3.56 (dd, J = 11.4, 5.0 Hz, 1H), 3.35 (dd, J = 10.1, 3.1 Hz, 1H), 3.19 (dd, J = 11.4, 10.1 Hz, 1H), 2.23 (brs, 1H), 2.13-2.02 (m, 1H), 1.85-1.78 (m, 1H), 1.42 (s, 3H), 1.22 (s, 3H), 0.97 (d, J = 7.2 Hz, 3H), 0.50 (d, J = 6.6 Hz, 3H) ppm;

^{13}C{^{1}H} NMR (101 MHz, C_6D_6): δ = 141.5, 114.2, 98.6, 79.8, 74.8, 66.4, 40.2, 33.0, 29.6, 19.2, 15.4, 13.3 ppm;

HRMS (ESI): $C_{12}H_{22}O_3Na$ $[M+Na]^+$ calculated: 237.1467, found: 237.1469;

R*f* = 0.3 (PE:MTBE 3:1, vanillin);

$[\alpha]_D^{24}$ = –28.6 (*c* 0.1, $CHCl_3$).

Analytical data for allylic alcohol **(*R*)-54a**:

^{1}**H NMR** (400 MHz, C_6D_6): δ = 5.82 (ddd, J = 17.1, 10.5, 4.1 Hz, 1H), 5.64 (dt, J = 17.1, 2.1 Hz, 1H), 5.21 (dt, J = 10.5, 2.1 Hz, 1H), 4.68-4.67 (m, 1H), 3.67 (brs, 1H), 3.48 (dd, J = 11.4, 5.0 Hz, 1H), 3.29 (dd, J = 10.4, 2.3 Hz, 1H), 3.15 (t, J = 11.4 Hz, 1H), 1.89-1.77 (m, 1H), 1.62-1.56 (m, 1H), 1.29 (s, 3H), 1.11 (s, 3H), 1.09 (d, J = 7.2 Hz, 3H), 0.27 (d, J = 6.7 Hz, 3H) ppm;

^{13}**C{^{1}H} NMR** (101 MHz, C_6D_6): δ = 140.6, 114.1, 98.9, 81.1, 70.8, 66.0, 37.8, 31.4, 29.7, 18.6, 12.4, 11.1 ppm;

HRMS (ESI): $C_{12}H_{22}O_3Na$ [M+Na]$^+$ calculated: 237.1467, found: 237.1469;

R$_f$ = 0.4 (PE:MTBE 5:1, vanillin);

$[\alpha]_D^{24}$ = –13.2 (c 0.4, $CHCl_3$).

(*S*)-Allylic alcohol 56a

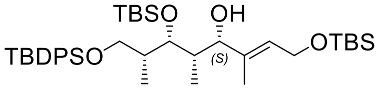

(*S*)-56a
$C_{39}H_{68}O_4Si_3$
685.22

According to GP9, TIB ester **55a** (323 mg, 0.44 mmol, 1.5 equiv), vinyl boronic ester **12** (91 mg, 0.29 mmol, 1.0 equiv) and TMEDA gave allylic alcohol **(*S*)-56a** (112 mg, contaminated with 2% ketone, 0.16 mmol, 55% o2s, dr 13:1) after purification by flash column chromatography (PE:MTBE 95:5) as a colorless oil.
According to GP10, carbamate **55b** (278 mg, 0.44 mmol, 1.5 equiv), vinyl boronic ester **12** (91 mg, 0.29 mmol, 1.0 equiv) and TMEDA gave allylic alcohol **(*S*)-56a** (126 mg, contaminated with 1% ketone, 0.18 mmol, 62% o2s, dr 1:1) after purification by flash column chromatography (cyclohexane:EtOAc 40:1) as a colorless oil.

Analytical data are given for allylic alcohol **(*S*)-56a** obtained by the reaction of TIB ester **55a** and TMEDA (dr 13:1).

^{1}**H NMR** (400 MHz, C_6D_6): δ = 7.84-7.80 (m, 4H), 7.28-7.22 (m, 6H), 5.79 (m$_c$, 1H), 4.27 (m$_c$, 2H), 4.01 (dd, J = 5.8, 2.5 Hz, 1H), 3.90 (brs, 1H), 3.83 (dd, J = 9.8, 6.8 Hz, 1H), 3.66 (dd, J = 9.8, 7.4 Hz, 1H), 2.11 (m$_c$, 1H), 1.85-1.78 (m, 1H), 1.41 (s, 3H), 1.23 (s, 9H), 1.02-0.97 (m, 25H), 0.13 (s, 3H), 0.12 (s, 6H), 0.06 (s, 3H) ppm;

^{13}**C{^{1}H} NMR** (101 MHz, C_6D_6): δ = 138.2, 136.14, 136.12, 134.42, 134.38, 130.05, 130.02, 128.1, 125.4, 76.5, 74.5, 67.6, 60.2, 40.4, 39.7, 27.3, 26.5, 26.2, 19.5, 18.8, 18.6, 13.6, 11.5, 10.0, −3.5, −3.6, −4.91, −4.92 ppm;

HRMS (ESI): $C_{39}H_{68}O_4Si_3Na$ [M+Na]$^+$ calculated: 707.4323, found: 707.4319;

R$_f$ = 0.3 (PE:MTBE 95:5, uv, vanillin);

$[\alpha]_D^{20}$ = −1.60 (c 1.0, $CHCl_3$).

(*S*)-Allylic alcohol 57b

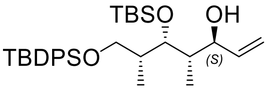

(*S*)-57b
$C_{31}H_{50}O_3Si_2$
526.91

According to GP9, TIB ester **55a** (164 mg, 0.22 mmol, 1.5 equiv), vinyl boronic ester **14** (23 mg, 0.15 mmol, 1.0 equiv) and TMEDA gave allylic alcohol **(*S*)-57b** (36 mg, 0.07 mmol, 47% o2s, dr 1.2:1) after purification by flash column chromatography (PE:MTBE 95:5) as a colorless oil.
According to GP10, carbamate **55b** (122 mg, 0.19 mmol, 1.5 equiv), vinyl boronic ester **14** (20 mg, 0.13 mmol, 1.0 equiv) and TMEDA gave allylic alcohol **(*S*)-57b** (31 mg, 0.06 mmol, 45% o2s, dr 3:1) after purification by flash column chromatography (PE:MTBE 95:5) as a colorless oil.
According to GP10, carbamate **55b** (278 mg, 0.44 mmol, 1.5 equiv), vinyl boronic ester **14** (45 mg, 0.29 mmol, 1.0 equiv) and (−)-sparteine gave allylic alcohol **(*S*)-57b** (53 mg, 0.10 mmol, 34% o2s, dr ≥ 19:1) after purification by flash column chromatography (cyclohexane:EtOAc 40:1) as a colorless oil.

Analytical data are given for allylic alcohol **(*S*)-57b** obtained by the reaction of carbamate **55b** and (−)-sparteine (dr ≥ 19:1).

^{1}H NMR (400 MHz, C_6D_6): δ = 7.81-7.78 (m, 4H), 7.28-7.23 (m, 6H), 5.80-5.71 (m, 1H), 5.24-5.19 (m, 1H), 5.04-5.01 (m, 1H), 4.28 (t, J = 3.4 Hz, 1H), 3.98 (t, J = 7.9 Hz, 1H), 3.67 (dd, J = 9.9, 7.0 Hz, 1H), 3.57 (dd, J = 9.9, 6.2 Hz, 1H), 2.54 (brs, 1H), 2.03-1.94 (m, 1H), 1.80-1.72 (m, 1H), 1.20 (s, 9H), 1.08 (d, J = 6.9 Hz, 3H), 0.97 (s, 9H), 0.81 (d, J = 7.0 Hz, 3H), 0.15 (s, 3H), 0.08 (s, 3H) ppm;

^{13}C{^{1}H} NMR (101 MHz, C_6D_6): δ = 141.2, 136.1, 134.24, 134.21, 130.1, 128.1, 115.5, 75.8, 74.1, 67.2, 43.6, 39.3, 27.2, 26.3, 19.5, 18.6, 13.2, 12.7, −3.9, −4.1 ppm;

HRMS (ESI): $C_{31}H_{50}O_3Si_2Na$ $[M+Na]^+$ calculated: 549.3196, found: 549.3184;

R$_f$ = 0.5 (PE:MTBE 9:1, uv, $KMnO_4$);

$[\alpha]_D^{31}$ = −9.4 (*c* 0.8, $CHCl_3$).

(*R*)-Allylic alcohol 57a

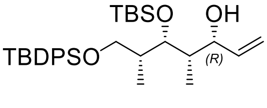

(*R*)-57a
$C_{31}H_{50}O_3Si_2$
526.91

According to GP10, carbamate **55b** (305 mg, 0.48 mmol, 1.5 equiv), vinyl boronic ester **14** (50 mg, 0.32 mmol, 1.0 equiv) and (+)-sparteine gave allylic alcohol **(*R*)-57a** (46 mg,

0.09 mmol, 28% o2s, dr ≥ 19:1) after purification by flash column chromatography (PE:MTBE 95:5) as a colorless oil.

^{1}H NMR (400 MHz, C_6D_6): δ = 7.84-7.76 (m, 4H), 7.28-7.20 (m, 6H), 5.74 (ddd, J = 17.2, 10.5, 5.1 Hz, 1H), 5.18 (dt, J = 17.2, 1.8 Hz, 1H), 5.01 (dt, J = 10.5, 1.7 Hz, 1H), 4.11 (brs, 1H), 4.03 (dd, J = 5.7, 2.6 Hz, 1H), 3.78 (dd, J = 10.1, 7.2 Hz, 1H), 3.63 (dd, J = 9.9, 6.9 Hz, 1H), 2.09 (m_c, 1H), 1.69 (m_c, 1H), 1.22 (s, 9H), 1.83 (d, J = 7.0 Hz, 1H), 0.99 (d, J = 6.8 Hz, 3H), 0.98-0.94 (m, 12H), 0.11 (s, 3H), 0.06 (s, 3H) ppm;

^{13}C{^{1}H} NMR (101 MHz, C_6D_6): δ = 141.4, 136.13, 136.12, 134.4, 134.3, 130.04, 130.02, 114.1, 74.1, 73.5, 67.4, 42.8, 40.2, 27.2, 26.4, 19.5, 18.7, 11.6, 10.2, −3.60, −3.64 ppm;

HRMS (ESI): $C_{31}H_{50}O_3Si_2$ [M+Na]$^+$ calculated: 549.3196, found: 549.3188;

R$_f$ = 0.5 (PE:MTBE 9:1, uv, vanillin);

$[\alpha]_D^{20}$ = +8.3 (c 0.8, $CHCl_3$).

(*S*)-Allylic alcohol 59a

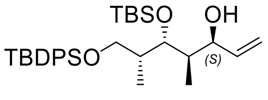

(*S*)-59a
$C_{31}H_{50}O_3Si_2$
526.91

According to GP9, TIB ester **58a** (323 mg, 0.44 mmol, 1.5 equiv), vinyl boronic ester **14** (45 mg, 0.29 mmol, 1.0 equiv) and TMEDA gave allylic alcohol **(*S*)-59a** (101 mg, 0.19 mmol, 66% o2s, dr ≥ 19:1) after purification by flash column chromatography (PE:MTBE 98:2) as a colorless oil.

^{1}H NMR (400 MHz, C_6D_6): δ = 7.80-7.77 (m, 4H), 7.27-7.22 (m, 6H), 5.73 (m_c, 1H), 5.33 (m_c, 1H), 5.07 (m_c, 1H), 4.58 (m_c, 1H), 4.01 (m_c, 1H), 3.70 (dd, J = 10.0, 6.8 Hz, 1H), 3.59 (dd, J = 10.0, 6.6 Hz, 1H), 2.05-1.97 (m, 2H), 1.63 (m_c, 1H), 1.20 (s, 9H), 0.98 (d, J = 6.9 Hz, 3H), 0.95 (s, 9H), 0.93 (d, J = 7.1 Hz, 3H), 0.13 (s, 3H), 0.06 (s, 3H) ppm;

^{13}C{^{1}H} NMR (101 MHz, C_6D_6): δ = 141.4, 136.10, 136.07, 134.2, 130.10, 130.08, 128.14, 128.13, 113.6, 75.9, 71.3, 67.4, 42.1, 39.5, 27.2, 26.5, 19.5, 18.7, 11.9, 10.7, −3.6, −3.8 ppm;

HRMS (ESI): $C_{31}H_{50}O_3Si_2Na$ [M+Na]$^+$ calculated: 549.3196, found: 549.3180;

R$_f$ = 0.3 (PE:MTBE 95:5, uv, vanillin);

$[\alpha]_D^{20}$ = −8.72 (c 1.2, $CHCl_3$).

(*R*)-Allylic alcohol 59b

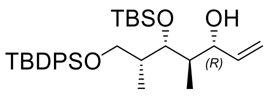

(*R*)-59b
$C_{31}H_{50}O_3Si_2$
526.91

According to GP10, carbamate **58b** (278 mg, 0.44 mmol, 1.5 equiv), vinyl boronic ester **14** (45 mg, 0.29 mmol, 1.0 equiv) and TMEDA gave allylic alcohol **(*R*)-59b** (23 mg, 0.04 mmol, 14% o2s, dr 1.2:1) after purification by flash column chromatography (PE:MTBE 95:5) as a colorless oil.

^{1}H NMR (400 MHz, C_6D_6): δ = 7.82-7.76 (m, 4H), 7.25-7.22 (m, 6H), 5.85 (m$_c$, 0.55H), 5.73 (m$_c$, 0.45H), 5.33 (m$_c$, 0.45H), 5.25 (m$_c$, 0.55H), 5.09-5.03 (m, 1H), 4.58 (brs, 0.45H), 4.23 (dd, *J* = 5.5, 2.3 Hz, 0.55H), 4.02-3.97 (m, 1H), 3.73-3.68 (m, 1H), 3.63-3.57 (m, 1H), 2.17 (brs, 0.52H), 2.05-1.97 (m, 1.48H), 1.86 (m$_c$, 0.57H), 1.63 (m$_c$, 0.43H), 1.21-1.20 (m, 9H), 0.99-0.86 (m, 15H), 0.13-0.12 (m, 3H), 0.06 (s, 3H) ppm;

^{13}C{^{1}H} NMR (101 MHz, C_6D_6): δ = 140.3, 136.12, 136.09, 134.23, 134.15, 130.08, 130.07, 128.125, 128.115, 115.3, 75.3, 73.4, 67.8, 45.1, 38.8, 27.2, 26.3, 19.5, 18.6, 13.0, 11.8, −3.8, −4.2 ppm;

HRMS (ESI): $C_{31}H_{50}O_3Si_2Na$ [M+Na]$^+$ calculated: 549.3196, found: 549.3189;

R*f* = 0.3 (PE:MTBE 95:5, uv, vanillin).

(*S*)-Allylic alcohol 61a

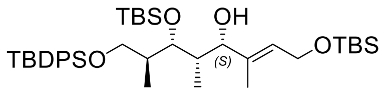

(*S*)-61a
$C_{39}H_{68}O_4Si_3$
685.22

According to GP9, *ent*-TIB ester **60a** (323 mg, 0.44 mmol, 1.5 equiv), vinyl boronic ester **12** (91 mg, 0.29 mmol, 1.0 equiv) and TMEDA gave allylic alcohol **(*S*)-61a** (44 mg, 0.06 mmol, 21% o2s, dr 11:1) after purification by flash column chromatography (PE:MTBE 95:5) as a colorless oil.

^{1}H NMR (400 MHz, C_6D_6): δ = 7.83-7.76 (m, 4H), 7.25-7.23 (m, 6H), 5.85 (m$_c$, 1H), 4.28 (d, *J* = 6.1 Hz, 2H), 3.99-3.95 (m, 2H), 3.82 (dd, *J* = 10.2, 6.5 Hz, 1H), 3.62 (dd, *J* = 10.2, 7.3 Hz, 1H), 2.20-2.09 (m, 1H), 1.92-1.84 (m, 1H), 1.48-1.47 (m, 4H), 1.19 (s, 9H), 1.05-1.00 (m, 15H), 0.95 (s, 9H), 0.14 (s, 3H), 0.12 (s, 6H), 0.06 (s, 3H) ppm;

^{13}C{^{1}H} NMR (101 MHz, C_6D_6): δ = 137.7, 136.1, 134.3, 134.1, 130.06, 130.05, 128.13, 128.12, 126.4, 78.6, 76.1, 66.6, 60.3, 41.9, 38.2, 27.2, 26.4, 26.2, 19.5, 18.7, 18.6, 14.0, 13.2, 9.7, −3.5, −3.7, −4.899, −4.904 ppm;

HRMS (ESI): $C_{39}H_{68}O_4Si_3Na$ [M+Na]$^+$ calculated: 707.4323, found: 707.4322;

$\mathbf{R}_f$ = 0.3 (PE:MTBE 95:5, uv, vanillin);

$[\alpha]_D^{20}$ = −3.75 (*c* 0.4, $CHCl_3$).

(*R*)-Allylic alcohol 61b

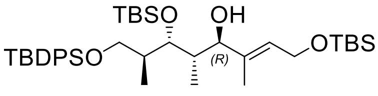

(*R*)-61b
$C_{39}H_{68}O_4Si_3$
685.22

According to GP10, *ent*-carbamate **60b** (278 mg, 0.44 mmol, 1.5 equiv), vinyl boronic ester **12** (91 mg, 0.29 mmol, 1.0 equiv) and TMEDA gave allylic alcohol **(*R*)-61b** (62 mg, contaminated with 8% ketone, 0.08 mmol, 28% o2s, dr 11:1) after purification by flash column chromatography (PE:MTBE 95:5) as a colorless oil.

^{1}H NMR (400 MHz, C_6D_6): δ = 7.84-7.81 (m, 4H), 7.26-7.23 (m, 6H), 5.65 (m_c, 1H), 4.30-4.16 (m, 3H), 4.02 (m_c, 1H), 3.87 (m_c, 1H), 3.56 (m_c, 1H), 2.15-2.10 (m, 1H), 1.86 (m_c, 1H), 1.55 (d, *J* = 0.9 Hz, 3H), 1.42 (brs, 1H), 1.22 (s, 9H), 1.07 (d, *J* = 6.9 Hz, 3H), 1.00 (s, 9H), 0.92 (s, 9H), 0.80 (d, *J* = 7.0 Hz, 3H), 0.105-0.096 (m, 9H), −0.04 (s, 3H) ppm;

^{13}C{^{1}H} NMR (101 MHz, C_6D_6): δ = 138.0, 136.19, 136.16, 134.40, 134.38, 130.0, 128.8, 128.1 (covered by C_6D_6), 79.5, 73.3, 67.4, 60.0, 41.4, 39.0, 27.3, 26.4, 26.2, 19.6, 18.6, 18.5, 14.4, 11.2, 10.8, −3.9, −4.94, −4.95 ppm;

HRMS (ESI): $C_{39}H_{68}O_4Si_3Na$ $[M+Na]^+$ calculated: 707.4323, found: 707.4321;

$\mathbf{R}_f$ = 0.3 (PE:MTBE 95:5, uv, vanillin);

$[\alpha]_D^{20}$ = −12.2 (*c* 1.0, $CHCl_3$).

(*R*)-Allylic alcohol 62b

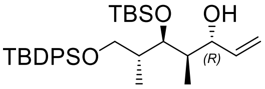

(*R*)-62b
$C_{31}H_{50}O_3Si_2$
526.91

According to GP9, TIB ester **60a** (184 mg, 0.25 mmol, 1.5 equiv), vinyl boronic ester **14** (26 mg, 0.17 mmol, 1.0 equiv) and TMEDA gave allylic alcohol **(*R*)-62b** (48 mg, 0.09 mmol, 53% o2s, dr 2:1) after purification by flash column chromatography (PE:MTBE 95:5) as a colorless oil.
According to GP10, carbamate **60b** (305 mg, 0.48 mmol, 1.5 equiv), vinyl boronic ester **14** (50 mg, 0.32 mmol, 1.0 equiv) and TMEDA gave allylic alcohol **(*R*)-62b** (60 mg, 0.11 mmol, 34% o2s, dr ≥ 19:1) after purification by flash column chromatography (PE:MTBE 95:5) as a colorless oil.

According to GP10, carbamate **60b** (305 mg, 0.48 mmol, 1.5 equiv), vinyl boronic ester **14** (50 mg, 0.32 mmol, 1.0 equiv) and (+)-sparteine gave allylic alcohol ***(R)*-62b** (33 mg, 0.06 mmol, 19% o2s, dr ≥ 19:1) after purification by flash column chromatography (PE:MTBE 95:5) as a colorless oil.

Analytical data are given for allylic alcohol ***(R)*-62b** obtained by the reaction of carbamate **60b** and TMEDA (dr ≥ 19:1).

^{1}H NMR (400 MHz, C_6D_6): δ = 7.84-7.76 (m, 4H), 7.28-7.20 (m, 6H), 5.70 (m_c, 1H), 5.13 (d, J = 17.2 Hz, 1H), 4.98 (m_c, 1H), 4.18 (m_c, 1H), 3.97 (m_c, 1H), 3.85 (t, J = 7.6 Hz, 1H), 3.56 (dd, J = 9.9, 8.3 Hz, 1H), 2.10 (m_c, 1H), 1.66 (m_c, 1H), 1.44 (brs, 1H), 1.22 (s, 9H), 1.83 (d, J = 6.9 Hz, 3H), 0.93 (s, 9H), 0.85 (d, J = 6.9 Hz, 3H), 0.11 (s, 3H), −0.02 (s, 3H) ppm;

^{13}C{^{1}H} NMR (101 MHz, C_6D_6): δ = 141.3, 136.17, 136.15, 134.4, 128.9, 115.7, 75.3, 73.1, 67.3, 41.7, 41.4, 27.24, 27.14, 26.4, 19.6, 18.6, 14.2, 11.2, −3.93, −3.96 ppm;

HRMS (ESI): $C_{31}H_{50}O_3Si_2Na$ $[M+Na]^+$ calculated: 549.3196, found: 549.3197;

R*f* = 0.4 (PE:MTBE 95:5, uv, vanillin);

$[\alpha]_D^{20}$ = −11.3 (*c* 1.1, $CHCl_3$).

(*S*)-Allylic alcohol 62a

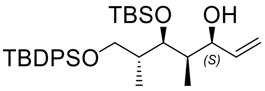

(*S*)-62a
$C_{31}H_{50}O_3Si_2$
526.91

According to GP9, TIB ester **60a** (323 mg, 0.44 mmol, 1.5 equiv), vinyl boronic ester **14** (45 mg, 0.29 mmol, 1.0 equiv) and (−)-sparteine gave allylic alcohol ***(S)*-62a** (55 mg, 0.10 mmol, 34% o2s, dr 14:1) after purification by flash column chromatography (PE:MTBE 95:5) as a colorless oil.

^{1}H NMR (400 MHz, C_6D_6): δ = 7.84-7.76 (m, 4H), 7.28-7.20 (m, 6H), 5.82 (ddd, J = 17.2, 10.5, 5.1 Hz, 1H), 5.31 (dt, J = 17.2, 1.8 Hz, 1H), 5.09 (dt, J = 10.5, 1.7 Hz, 1H), 4.17 (brt, J = 4.6 Hz, 1H), 4.04 (t, J = 3.9 Hz, 1H), 3.80 (dd, J = 10.3, 7.3 Hz, 1H), 3.58 (dd, J = 10.3, 6.8 Hz, 1H), 2.21-2.11 (m, 1H), 1.84-1.75 (m, 1H), 1.66 (brs, 1H), 1.19 (s, 9H), 1.03 (d, J = 7.0 Hz, 3H), 0.95 (s, 9H), 0.92 (d, J = 7.0 Hz, 3H), 0.11 (s, 3H), 0.04 (s, 3H) ppm;

^{13}C{^{1}H} NMR (101 MHz, C_6D_6): δ = 141.0, 136.08, 136.07, 134.13, 134.11, 130.1, 128.1, 114.6, 75.4, 75.3, 66.6, 41.8, 40.9, 27.2, 26.3, 19.5, 18.6, 13.5, 10.2, −3.6, −3.9 ppm;

HRMS (ESI): $C_{31}H_{50}O_3Si_2Na$ $[M+Na]^+$ calculated: 549.3196, found: 549.3197;

R*f* = 0.2 (PE:MTBE 95:5, uv, vanillin);

$[\alpha]_D^{20}$ = +6.9 (*c* 0.7, $CHCl_3$).

(*S*)-Allylic alcohol 64a

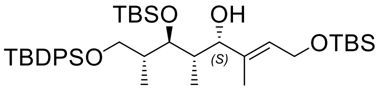

(*S*)-64a
$C_{39}H_{68}O_4Si_3$
685.22

According to GP9, *ent*-TIB ester **63a** (323 mg, 0.44 mmol, 1.5 equiv), vinyl boronic ester **12** (91 mg, 0.29 mmol, 1.0 equiv) and TMEDA gave allylic alcohol **(*S*)-64a** (148 mg, 0.22 mmol, 76% o2s, dr 10:1) after purification by flash column chromatography (PE:MTBE 95:5) as a colorless oil.

^{1}H NMR (400 MHz, C_6D_6): δ = 7.80-7.77 (m, 4H), 7.25-7.24 (m, 6H), 6.10 (m$_c$, 1H), 4.45 (brs, 1H), 4.33 (m$_c$, 2H), 3.94 (dd, J = 10.0, 6.0 Hz, 1H), 3.80 (dd, J = 5.7, 4.0 Hz, 1H), 3.58 (dd, J = 10.0, 7.6 Hz, 1H), 2.94 (d, J = 1.8 Hz, 1H), 2.29-2.18 (m, 1H), 1.90 (m$_c$, 1H), 1.47 (s, 3H), 1.20 (s, 9H), 1.02-0.98 (m, 15H), 0.89 (s, 9H), 0.13 (s, 6H), 0.05 (s, 3H), −0.05 (s, 3H) ppm;

^{13}C{^{1}H} NMR (101 MHz, C_6D_6): δ = 137.1, 136.102, 136.096, 134.14, 134.13, 130.10, 130.06, 128.15, 128.13, 124.8, 79.3, 74.3, 66.7, 60.4, 40.7, 37.3, 27.2, 26.3, 26.2, 19.5, 18.6, 18.5, 14.5, 14.1, 11.7, −3.85, −3.88, −4.8, −4.9 ppm;

HRMS (ESI): $C_{39}H_{68}O_4Si_3Na$ [M+Na]$^+$ calculated: 707.4323, found: 707.4333;

R$_f$ = 0.3 (PE:MTBE 95:5, uv, vanillin);

$[\alpha]_D^{20}$ = +3.74 (*c* 0.9, $CHCl_3$).

(*R*)-Allylic alcohol 64b

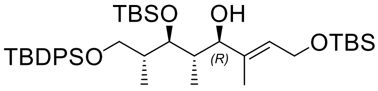

(*R*)-64b
$C_{39}H_{68}O_4Si_3$
685.22

According to GP10, *ent*-carbamate **63b** (278 mg, 0.44 mmol, 1.5 equiv), vinyl boronic ester **12** (91 mg, 0.29 mmol, 1.0 equiv) and TMEDA gave allylic alcohol **(*R*)-64b** (101 mg, 0.15 mmol, 50% o2s, dr 2:1) after purification by flash column chromatography (PE:MTBE 20:1) as a colorless oil.

^{1}H NMR (400 MHz, C_6D_6): δ = 7.83-7.77 (m, 4H), 7.26-7.24 (m, 6H), 5.66-5.63 (m, 1H), 4.25-4.22 (m, 2H), 4.04-4.00 (m, 2H), 3.90 (d, J = 10.7 Hz, 1H), 3.63 (dd, J = 10.2, 8.2 Hz, 1H), 2.28 (d, J = 1.6 Hz, 1H), 2.28-2.16 (m, 1H), 2.03-1.94 (m, 1H), 1.61 (s, 3H), 1.22 (s, 9H), 1.17 (d, J = 6.9 Hz, 3H), 1.01 (s, 9H), 0.88 (s, 9H), 0.80 (d, J = 7.0 Hz, 3H), 0.11 (s, 6H), 0.09 (s, 3H), −0.01 (s, 3H) ppm;

^{13}C{^{1}H} NMR (101 MHz, C_6D_6): δ = 137.5, 136.1, 134.43, 134.41, 130.04, 130.02, 128.6, 80.8, 77.6, 66.9, 60.1, 40.8, 40.3, 27.3, 26.2, 26.1, 19.6, 18.5, 18.3, 14.9, 14.5, 11.0, −4.1, −4.4, −4.9, −5.0 ppm;

HRMS (ESI): $C_{39}H_{68}O_4Si_3Na$ [M+Na]$^+$ calculated: 707.4323, found: 707.4333;

R*f* = 0.3 (PE:MTBE 95:5, uv, vanillin);

$[\alpha]_D^{31}$ = +4.6 (*c* 1.0, $CHCl_3$).

(*S*)-Allylic alcohol 65a

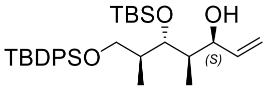

(*S*)-65a
$C_{31}H_{50}O_3Si_2$
526.91

According to GP9, TIB ester **63a** (323 mg, 0.44 mmol, 1.5 equiv), vinyl boronic ester **14** (45 mg, 0.29 mmol, 1.0 equiv) and TMEDA gave allylic alcohol **(*S*)-65a** (65 mg) and allylic alcohol **(*R*)-65b** (32 mg, 97 mg in total, 0.18 mmol, 62% o2s, dr 2:1) after purification by flash column chromatography (PE:MTBE 98:2 → 93:7) as a colorless oil.

Analytical data are given for the pure allylic alcohol **(*S*)-65a**.

^{1}H NMR (400 MHz, C_6D_6): δ = 7.79-7.77 (m, 4H), 7.25-7.23 (m, 6H), 5.74 (m$_c$, 1H), 5.45 (m$_c$, 1H), 5.11 (m$_c$, 1H), 4.67 (m$_c$, 1H), 3.88 (dd, *J* = 10.0, 6.1 Hz, 1H), 3.76 (dd, *J* = 5.4, 4.1 Hz, 1H), 3.56 (dd, *J* = 10.0, 7.6 Hz, 1H), 2.72 (d, *J* = 2.4 Hz, 1H), 2.19 (m$_c$, 1H), 1.77-1.70 (m, 1H), 1.20 (s, 9H), 1.01-0.95 (m, 6H), 0.89 (s, 9H), 0.07 (s, 3H), −0.05 (s, 3H) ppm;

^{13}C{^{1}H} NMR (101 MHz, C_6D_6): δ = 141.0, 136.1, 134.2, 134.1, 130.10, 130.06, 128.14, 128.12, 113.7, 79.1, 71.6, 66.6, 40.9, 39.8, 27.2, 26.3, 19.5, 18.5, 14.3, 11.9, −3.87, −3.91 ppm;

HRMS (ESI): $C_{31}H_{50}O_3Si_2Na$ [M+Na]$^+$ calculated: 549.3196, found: 549.3184;

R*f* = 0.3 (PE:MTBE 95:5, uv, vanillin);

$[\alpha]_D^{20}$ = −10.5 (*c* 0.9, $CHCl_3$).

(*R*)-Allylic alcohol 65b

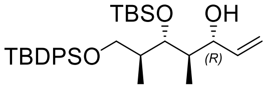

(*R*)-65b
$C_{31}H_{50}O_3Si_2$
526.91

According to GP10, carbamate **63b** (278 mg, 0.44 mmol, 1.5 equiv), vinyl boronic ester **14** (45 mg, 0.29 mmol, 1.0 equiv) and TMEDA gave allylic alcohol **(*R*)-65b** (23 mg, 0.04 mmol, 14% o2s, dr ≥ 19:1) after purification by flash column chromatography (PE:MTBE 95:5) as a colorless oil.

^{1}H NMR (400 MHz, C_6D_6): δ = 7.81-7.79 (m, 4H), 7.25-7.23 (m, 6H), 5.82 (m$_c$, 1H), 5.25 (m$_c$, 1H), 5.05 (m$_c$, 1H), 4.01 (m$_c$, 1H), 3.97-3.93 (m, 2H), 3.59 (dd, *J* = 10.2, 8.1 Hz, 1H), 2.25-2.16

(m, 2H), 1.84 (m_c, 1H), 1.21 (s, 9H), 1.12 (d, J = 6.9 Hz, 3H), 0.88 (s, 9H), 0.84 (d, J = 7.1 Hz, 3H), 0.06 (s, 3H), −0.01 (s, 3H) ppm;

$^{13}C\{^1H\}$ NMR (101 MHz, C_6D_6): δ = 140.5, 136.10, 136.08, 134.33, 134.31, 130.03, 130.00, 128.1, 115.2, 77.6, 75.4, 66.7, 42.9, 41.0, 27.2, 26.2, 19.5, 18.4, 14.5, 14.2, −4.1, −4.3 ppm;

HRMS (ESI): $C_{31}H_{50}O_3Si_2Na$ $[M+Na]^+$ calculated: 549.3196, found: 549.3180;

R_f = 0.1 (PE:MTBE 95:5, uv, vanillin);

$[\alpha]_D^{20}$ = +2.31 (c 0.8, $CHCl_3$).

2-11. Analysis of stereochemistry

General Procedure 11 (GP11): Mosher ester[9]

Note: Since the scale of the performed reactions differs due to need and availability, this general procedure reports the relative molar quantities, equivalents and relative solvent volumes of a representative experiment, whereby the exact volumes/weights of the structure-building (or key) chemicals are listed in the corresponding individual procedure.

To a stirred solution of allylic alcohol (26.2 µmol, 1.0 equiv) in CH_2Cl_2 (1.3 mL, 0.02 M) were added Et_3N (262 µmol, 10.0 equiv), DMAP (157 µmol, 6.0 equiv) and MTPACl (157 µmol, 6.0 equiv) successively. The reaction mixture was stirred at rt until TLC showed full conversion. After the addition of MTBE and aq. NaOH (2.0 M) the organic layer was separated. The organic layer was washed with aq. NaOH (2.0 M, 3x), sat. aq. $NaHCO_3$ (3x), aq. $CuSO_4$ (1.0 M) and sat. aq. NaCl, dried over Na_2SO_4 and concentrated *in vacuo*.

General Procedure 12 (GP12): PMP acetal formation

A suspension of allylic alcohol (144 µmol, 1.0 equiv) and activated molecular sieve (4Å, 5 mg/mg) in CH_2Cl_2 (5.0 mL, 0.03 M) was stirred for 1 h at rt. The suspension was then cooled to 0 °C and DDQ (360 µmol, 2.5 equiv) was added. After addition of sat. aq. $NaHCO_3$ and sat. aq. $Na_2S_2O_3$ the mixture was warmed to rt. The biphasic mixture was filtered through Celite and the filtrate was extracted with CH_2Cl_2 (3x). The combined organic layers were washed with sat. aq. NaCl. The organic layer was dried over Na_2SO_4 and concentrated *in vacuo*. The crude product was purified by flash column chromatography to afford the corresponding PMP acetal.

9 Unless otherwise noted, the stereochemistry of at least one result (per stereo-motif) of the branched vinyl boronic ester(s) was determined via Mosher-analysis. The stereochemistry of the other results of the same motif were assigned in analogy to the one determined via Mosher-analysis.

(*S*)-Mosher ester S17

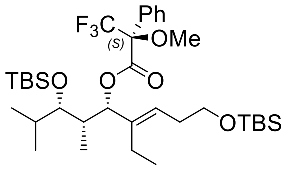

S17
$C_{35}H_{61}F_3O_5Si_2$
675.03

Following GP11, allylic alcohol **(*S*)-15a** (12 mg, 26.2 µmol, 1.0 equiv) and (*R*)-MTPACl gave (*S*)-mosher ester **S17** (16 mg, 23.7 µmol, 90%) as a yellow oil.

^{1}H NMR (400 MHz, C_6D_6): δ = 7.72-7.70 (m, 2H), 7.14-7.06 (m, 3H), 5.76 (d, J = 6.2 Hz, 1H), 5.68 (m_c, 1H), 3.57 (m_c, 2H), 3.49 (d, J = 0.9 Hz, 3H), 3.44 (m_c, 1H), 2.27 (m_c, 2H), 2.11-2.05 (m, 1H), 2.00 (m_c, 1H), 1.91 (m_c, 1H), 1.86-1.80 (m, 1H), 1.06 (m_c, 6H), 1.03 (s, 9H), 0.98 (s, 9H), 0.88 (d, J = 6.9 Hz, 6H), 0.17 (s, 3H), 0.08 (s, 3H), 0.07 (s, 6H) ppm.

(*R*)-Mosher ester S18

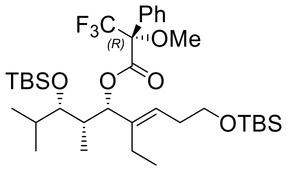

S18
$C_{35}H_{61}F_3O_5Si_2$
675.03

Following GP11, allylic alcohol **(*S*)-15a** (12 mg, 26.2 µmol, 1.0 equiv) and (*S*)-MTPACl gave (*R*)-mosher ester **S18** (17 mg, 25.2 µmol, ≥ 95%) as a yellow oil.

^{1}H NMR (400 MHz, C_6D_6): δ = 7.70-7.68 (m, 2H), 7.15-7.10 (m, 2H), 7.08-7.04 (m, 1H), 5.70 (d, J = 5.8 Hz, 1H), 5.55 (m_c, 1H), 3.56-3.52 (m, 3H), 3.48 (d, J = 1.0 Hz, 3H), 2.24 (m_c, 2H), 2.02 (m_c, 2H), 1.89-1.77 (m, 2H), 1.16 (d, J = 6.8 Hz, 3H), 1.03 (s, 9H), 0.98 (s, 9H), 0.96 (t, J = 7.5 Hz, 3H), 0.92 (d, J = 7.0 Hz, 3H), 0.90 (d, J = 6.8 Hz, 3H), 0.18 (s, 3H), 0.10 (s, 3H), 0.071 (s, 3H), 0.066 (s, 3H) ppm.

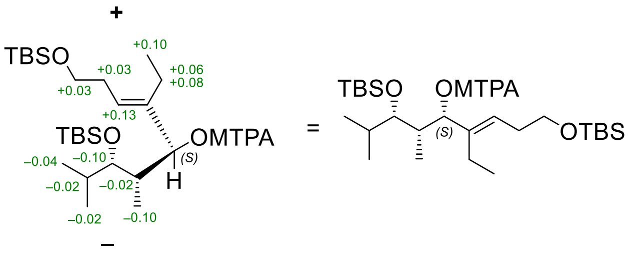

Figure 1. Mosher-analysis of allylic alcohol **(*S*)-15a**.

(*S*)-Mosher ester S19

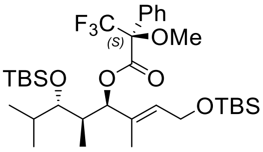

S19
$C_{33}H_{57}F_3O_5Si_2$
646.98

Following GP11, allylic alcohol **(*R*)-20a** (10 mg, 23.2 µmol, 1.0 equiv) and (*R*)-MTPACl gave (*S*)-mosher ester **S19** (14 mg, 21.6 µmol, 93%) as a yellow oil.

^{1}H NMR (400 MHz, C_6D_6): δ = 7.74-7.72 (m, 2H), 7.14-7.12 (m, 2H), 7.09-7.05 (m, 1H), 5.75 (m_c, 1H), 5.59 (d, *J* = 6.7 Hz, 1H), 4.12 (m_c, 2H), 3.49 (m_c, 4H), 2.06 (m_c, 1H), 1.75 (m_c, 1H), 1.50 (d, *J* = 0.9 Hz, 3H), 1.06 (d, *J* = 7.0 Hz, 3H), 0.99 (s, 9H), 0.97 (s, 9H), 0.94 (d, *J* = 7.0 Hz, 3H), 0.92 (d, *J* = 6.7 Hz, 3H), 0.08 (s, 3H), 0.07 (s, 3H), 0.06 (s, 6H) ppm.

(*R*)-Mosher ester S20

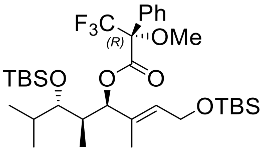

S20
$C_{33}H_{57}F_3O_5Si_2$
646.98

Following GP11, allylic alcohol **(*R*)-20a** (10 mg, 23.2 µmol, 1.0 equiv) and (*S*)-MTPACl gave (*R*)-mosher ester **S20** (13 mg, 20.1 µmol, 87%) as a yellow oil.

^{1}H NMR (400 MHz, C_6D_6): δ = 7.74-7.72 (m, 2H), 7.13-7.06 (m, 3H), 5.83 (m_c, 1H), 5.65 (d, *J* = 7.2 Hz, 1H), 4.12 (m_c, 2H), 3.49 (d, *J* = 1.0 Hz, 3H), 3.45 (m_c, 1H), 2.06 (m_c, 1H), 1.73 (m_c, 1H), 1.63 (s, 3H), 0.99 (s, 9H), 0.97 (s, 9H), 0.95 (d, *J* = 7.1 Hz, 3H), 0.92 (d, *J* = 6.4 Hz, 3H), 0.91 (d, *J* = 6.5 Hz, 3H), 0.11 (s, 3H), 0.08 (s, 3H), 0.06 (s, 6H) ppm.

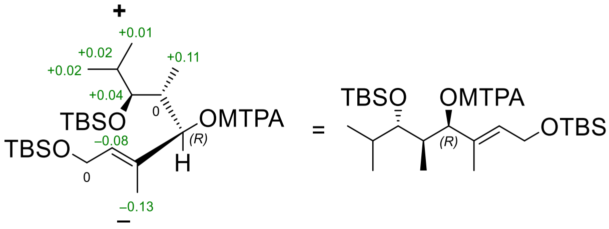

Figure 2. Mosher-analysis of allylic alcohol **(*R*)-20a**.

(*S*)-Mosher ester S21

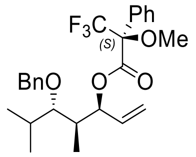

S21
$C_{26}H_{31}F_3O_4$
464.53

Following GP11, allylic alcohol **(*S*)-27a** (10 mg, 40.3 µmol, 1.0 equiv) and (*R*)-MTPACl gave (*S*)-mosher ester **S21** (17 mg, 36.6 µmol, 91%) as a yellow oil.

^{1}H NMR (400 MHz, C_6D_6): δ = 7.76 (d, J = 7.6 Hz, 2H), 7.46 (d, J = 7.1 Hz, 2H), 7.21-7.00 (m, 5H), 6.18 (m$_c$, 1H), 5.49 (ddd, J = 17.1, 10.6 Hz, 6.4 Hz, 1H), 5.09 (d, J = 17.3 Hz, 1H), 4.93 (d, J = 10.7 Hz, 1H), 4.58 (d, J = 10.7 Hz, 1H), 4.48 (d, J = 10.6 Hz, 1H), 3.42 (s, 3H), 3.10 (dd, J = 9.1, 2.3 Hz, 2H), 1.84-1.71 (m, 2H), 0.99 (d, J = 6.9 Hz, 3H), 0.89 (d, J = 6.8 Hz, 3H), 0.83 (d, J = 7.1 Hz, 3H) ppm.

(*R*)-Mosher ester S22

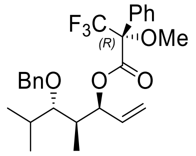

S22
$C_{26}H_{31}F_3O_4$
464.53

Following GP11, allylic alcohol **(*S*)-27a** (15 mg, 60.4 µmol, 1.0 equiv) and (*S*)-MTPACl gave (*R*)-mosher ester **S22** (25 mg, 53.8 µmol, 89%) as a yellow oil.

^{1}H NMR (400 MHz, C_6D_6): δ = 7.75 (d, J = 7.8 Hz, 2H), 7.48 (d, J = 7.2 Hz, 2H), 7.22-6.99 (m, 5H), 6.14 (m$_c$, 1H), 5.68-5.58 (ddd, J = 17.1, 10.6, 6.4 Hz, 1H), 5.18 (dt, J = 17.3, 1.2 Hz, 1H), 4.98 (dt, J = 10.6, 1.2 Hz, 1H), 4.61 (d, J = 10.6 Hz, 1H), 4.47 (d, J = 10.6 Hz, 1H), 3.45 (s, 3H), 3.01 (dd, J = 9.3, 2.3 Hz, 2H), 1.84-1.74 (m, 1H), 1.74-1.64 (m, 1H), 0.96 (d, J = 6.9 Hz, 3H), 0.86 (d, J = 6.8 Hz, 3H), 0.78 (d, J = 7.1 Hz, 3H) ppm.

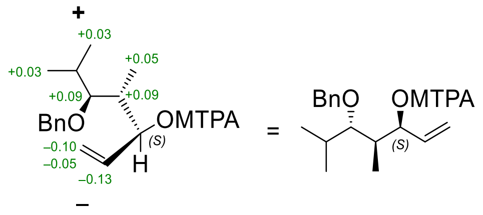

Figure 3. Mosher-analysis of allylic alcohol **(*S*)-27a**.

(*S*)-Mosher ester S23

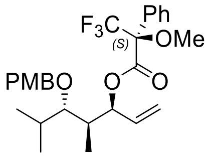

S23
$C_{27}H_{33}F_3O_5$
494.55

Following GP11, allylic alcohol **(*S*)-28a** (10 mg, 35.9 µmol, 1.0 equiv) and (*R*)-MTPACl gave (*S*)-mosher ester **S23** (14 mg, 28.3 µmol, 79%) as a yellow oil.

^{1}H NMR (400 MHz, C_6D_6): δ = 7.79 (d, *J* = 7.6 Hz, 2H), 7.42 (d, *J* = 8.5 Hz, 2H), 7.12-7.00 (m, 3H), 6.80 (d, *J* = 8.5 Hz, 2H), 6.21 (d, *J* = 4.1 Hz, 1H), 5.52 (ddd, *J* = 16.9, 10.3, 5.5 Hz, 1H), 5.11 (d, *J* = 17.2 Hz, 1H), 4.94 (d, *J* = 10.7 Hz, 1H), 4.54 (d, *J* = 10.1 Hz, 1H), 4.48 (d, *J* = 10.1 Hz, 1H), 3.44 (s, 3H), 3.27 (s, 3H), 3.12 (d, *J* = 9.4, 2.4 Hz, 1H), 1.85-1.72 (m, 2H), 1.03 (d, *J* = 6.8 Hz, 3H), 0.91 (d, *J* = 7.0 Hz, 3H), 0.85 (d, *J* = 6.9 Hz, 3H) ppm.

(*R*)-Mosher ester S24

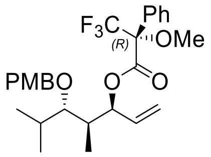

S24
$C_{27}H_{33}F_3O_5$
494.55

Following GP11, allylic alcohol **(*S*)-28a** (14 mg, 50.3 µmol, 1.0 equiv) and (*S*)-MTPACl gave (*R*)-mosher ester **S24** (20 mg, 40.4 µmol, 80%) as a yellow oil.

^{1}H NMR (400 MHz, C_6D_6): δ = 7.77 (d, *J* = 7.6 Hz, 2H), 7.43 (d, *J* = 8.4 Hz, 2H), 7.07-7.03 (m, 3H), 6.82 (d, *J* = 8.6 Hz, 2H), 6.19 (d, *J* = 6.3 Hz, 1H), 5.65 (ddd, *J* = 16.9, 10.3, 6.6 Hz, 1H), 5.22 (d, *J* = 17.3 Hz, 1H), 4.99 (d, *J* = 10.6 Hz, 1H), 4.56 (d, *J* = 10.2 Hz, 1H), 4.46 (d, *J* = 10.2 Hz, 1H), 3.46 (s, 3H), 3.29 (s, 3H), 3.02 (dd, *J* = 9.4, 2.2 Hz, 1H), 1.84-1.75 (m, 1H), 1.75-1.66 (m, 1H), 0.99 (d, *J* = 6.8 Hz, 3H), 0.87 (d, *J* = 6.9 Hz, 3H), 0.80 (d, *J* = 6.9 Hz, 3H) ppm.

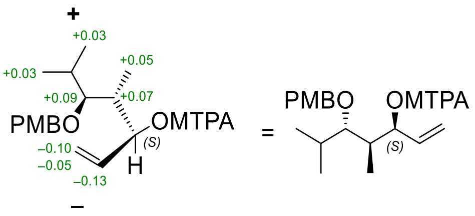

Figure 4. Mosher-analysis of allylic alcohol **(*S*)-28a**.

PMP acetal S25

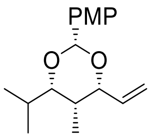

S25
$C_{17}H_{24}O_3$
276.38

Following GP12, allylic alcohol ***(R)*-32a** (40 mg, 144 µmol, 1.0 equiv, dr 3:1) gave PMP acetal **S25** (31 mg, 112 µmol, 78%, dr 5:1) after purification by flash column chromatography (PE:MTBE 95:5) as a colorless oil.

Chemical shift values are given for the major diastereoisomer.

^{1}H NMR (400 MHz, C_6D_6): δ = 7.65-7.61 (m, 2H), 6.85-6.81 (m, 2H), 5.74 (m_c, 1H), 5.48-5.43 (m, 2H), 5.15 (m_c, 1H), 4.17 (m_c, 1H), 3.28 (s, 3H), 3.10 (dd, J = 9.8, 2.1 Hz, 1H), 1.85-1.75 (m, 1H), 1.43-1.38 (m, 1H), 1.10 (d, J = 6.5 Hz, 3H), 1.01 (d, J = 6.8 Hz, 3H), 0.67 (d, J = 6.8 Hz, 3H) ppm;

^{13}C{^{1}H} NMR (101 MHz, C_6D_6): δ = 160.4, 137.5, 132.4, 128.0, 114.8, 113.8, 101.6, 86.6, 81.1, 54.8, 34.0, 29.7, 20.0, 17.4, 6.4 ppm;

HRMS (ESI): $C_{17}H_{24}O_3Na$ $[M+Na]^+$ calculated: 299.1623, found: 299.1626;

R*f* = 0.6 (PE:MTBE 95:5, uv, CAN);

$[\alpha]_D^{20}$ = −7.10 (*c* 1.8, $CHCl_3$).

The stereochemistry of the formed allylic alcohol was determined by NOE correlations.

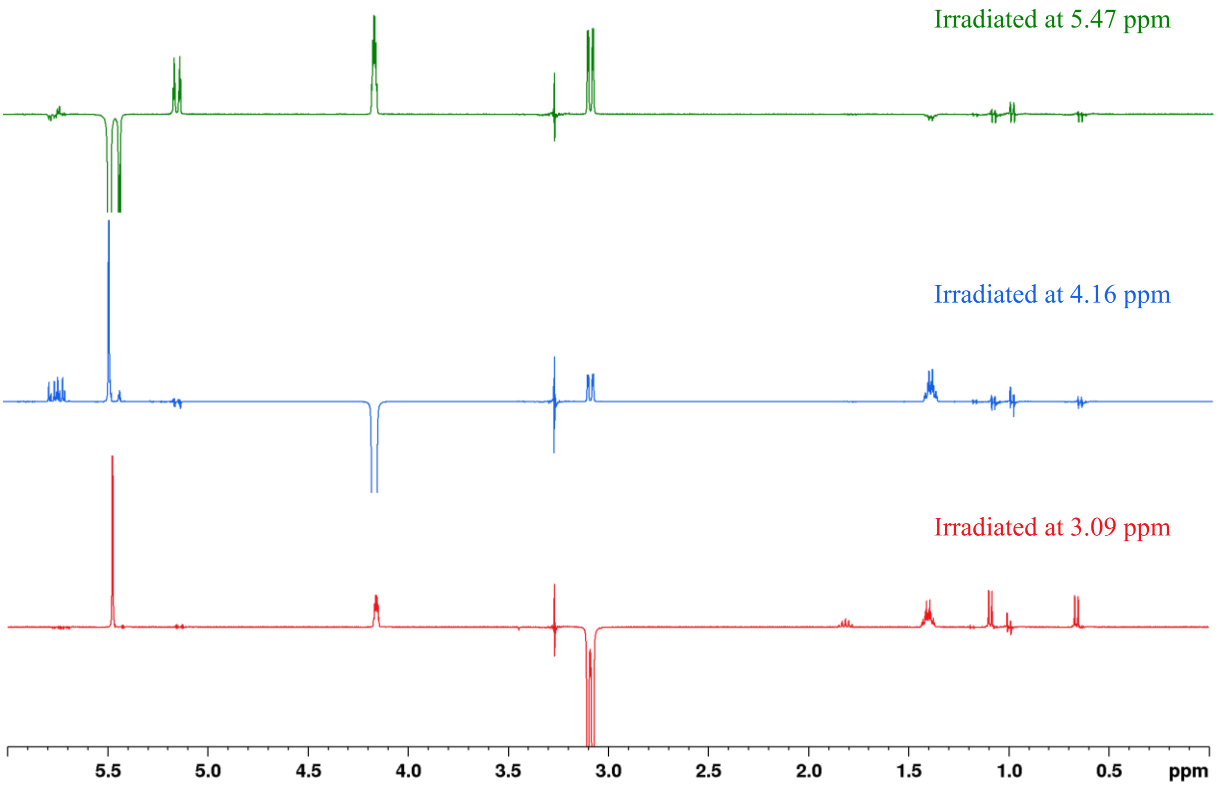

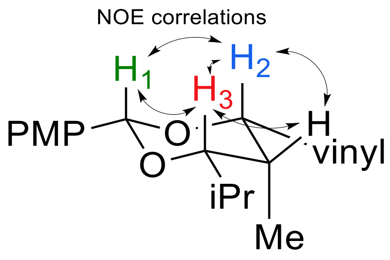

Figure 5. NOE correlations in PMP acetal **S25**.

(*S*)-Mosher ester S26

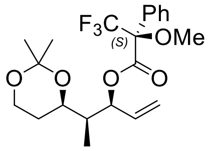

S26
$C_{21}H_{27}F_3O_5$
416.44

Following GP11, allylic alcohol **(*S*)-38a** (7.0 mg, 35.0 µmol, 1.0 equiv) and (*R*)-MTPACl gave (*S*)-mosher ester **S26** (13 mg, 31.2 µmol, 89%) as a yellow oil.

^{1}H NMR (400 MHz, C_6D_6): δ = 7.70-7.68 (m, 2H), 7.12-7.02 (m, 3H), 5.60-5.56 (m, 1H), 5.52 (m_c, 1H), 5.19 (m_c, 1H), 5.02 (m_c, 1H), 3.69 (m_c, 1H), 3.61-3.57 (m, 2H), 3.43 (d, J = 1.1 Hz, 3H), 1.58-1.46 (m, 2H), 1.44 (s, 3H), 1.22 (s, 3H), 1.02 (d, J = 7.0 Hz, 3H), 0.80 (m_c, 1H) ppm.

(*R*)-Mosher ester S27

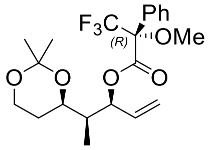

S27
$C_{21}H_{27}F_3O_5$
416.44

Following GP11, allylic alcohol **(*S*)-38a** (7.0 mg, 35.0 µmol, 1.0 equiv) and (*S*)-MTPACl gave (*R*)-mosher ester **S27** (14 mg, 33.6 µmol, ≥ 95%) as a yellow oil.

^{1}H NMR (400 MHz, C_6D_6): δ = 7.69-7.68 (m, 2H), 7.10-7.01 (m, 3H), 5.68-5.58 (m, 2H), 5.28-5.24 (m, 1H), 5.06-5.04 (m, 1H), 3.62-3.53 (m, 3H), 3.45 (d, J = 1.2 Hz, 3H), 1.53-1.44 (m, 2H), 1.42 (s, 3H), 1.17 (s, 3H), 0.94 (d, J = 6.9 Hz, 3H), 0.78 (m_c, 1H) ppm.

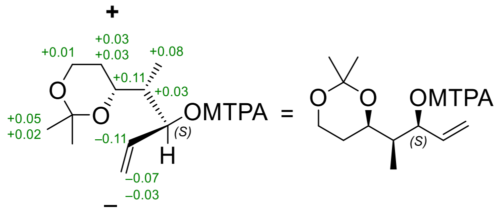

Figure 6. Mosher-analysis of allylic alcohol **(*S*)-38a**.

(*S*)-Mosher ester S28

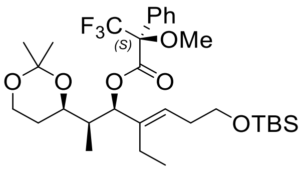

S28
$C_{31}H_{49}F_3O_6Si$
602.81

Following GP11, allylic alcohol **(*R*)-35a** (12 mg, 31.0 µmol, 1.0 equiv) and (*R*)-MTPACl gave (*S*)-mosher ester **S28** (17 mg, 28.2 µmol, 91%) as a yellow oil.

^{1}H NMR (400 MHz, C_6D_6): δ = 7.69-7.68 (m, 2H), 7.13-7.06 (m, 3H), 5.66 (d, J = 6.1 Hz, 1H), 5.47 (m_c, 1H), 3.75 (m_c, 1H), 3.65-3.63 (m, 2H), 3.51 (t, J = 6.9 Hz, 2H), 3.48 (d, J = 1.0 Hz, 3H), 2.23 (m_c, 2H), 2.00 (m_c, 1H), 1.78 (m_c, 1H), 1.71 (m_c, 1H), 1.59 (m_c, 1H), 1.49 (s, 3H), 1.29 (s, 3H), 1.13 (d, J = 6.8 Hz, 3H), 0.97-0.90 (m, 13H), 0.06 (s, 3H), 0.05 (s, 3H) ppm.

(*R*)-Mosher ester S29

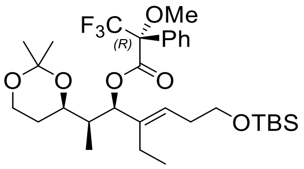

S29
$C_{31}H_{49}F_3O_6Si$
602.81

Following GP11, allylic alcohol **(*R*)-35a** (12 mg, 31.0 µmol, 1.0 equiv) and (*S*)-MTPACl gave (*R*)-mosher ester **S29** (16 mg, 26.5 µmol, 85%) as a yellow oil.

^{1}H NMR (400 MHz, C_6D_6): δ = 7.70-7.68 (m, 2H), 7.12-7.05 (m, 3H), 5.71 (d, J = 6.2 Hz, 1H), 5.62 (m_c, 1H), 3.67-3.61 (m, 3H), 3.54 (m_c, 1H), 3.48 (d, J = 1.0 Hz, 3H), 2.26 (m_c, 2H), 2.07 (m_c, 1H), 1.89 (m_c, 1H), 1.69 (m_c, 1H), 1.60-1.50 (m, 1H), 1.47 (s, 3H), 1.26 (s, 3H), 1.04 (d, J = 6.9 Hz, 3H), 1.01 (t, J = 7.6 Hz, 3H), 0.97 (s, 9H), 0.94-0.90 (m, 2H), 0.05 (s, 6H) ppm.

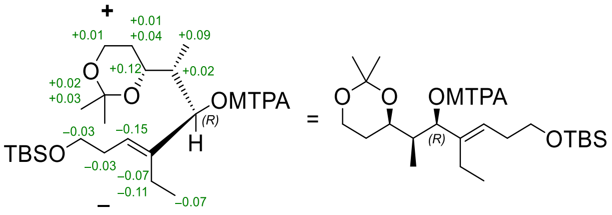

Figure 7. Mosher-analysis of allylic alcohol **(*R*)-35a**.

(*S*)-Mosher ester S30

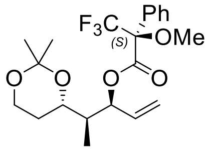

S30
$C_{21}H_{27}F_3O_5$
416.44

Following GP11, allylic alcohol **(*S*)-42a** (7.0 mg, 35.0 µmol, 1.0 equiv) and (*R*)-MTPACl gave (*S*)-mosher ester **S30** (14 mg, 33.6 µmol, ≥ 95%) as a yellow oil.

^{1}H NMR (400 MHz, C_6D_6): δ = 7.75-7.73 (m, 2H), 7.13-7.04 (m, 3H), 6.24 (m$_c$, 1H), 5.54 (m$_c$, 1H), 5.11 (m$_c$, 1H), 4.95 (m$_c$, 1H), 3.66-3.54 (m, 3H), 3.42 (d, J = 1.0 Hz, 3H), 1.60 (m$_c$, 1H), 1.50 (s, 3H), 1.34 (s, 3H), 1.32-1.22 (m, 1H), 0.98 (m$_c$, 1H), 0.73 (d, J = 7.1 Hz, 3H) ppm.

(*R*)-Mosher ester S31

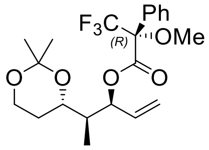

S31
$C_{21}H_{27}F_3O_5$
416.44

Following GP11, allylic alcohol **(*S*)-42a** (6.0 mg, 30.0 µmol, 1.0 equiv) and (*S*)-MTPACl gave (*R*)-mosher ester **S31** (11 mg, 26.4 µmol, 88%) as a yellow oil.

^{1}H NMR (400 MHz, C_6D_6): δ = 7.73-7.71 (m, 2H), 7.11-7.02 (m, 3H), 6.23 (m$_c$, 1H), 5.64 (m$_c$, 1H), 5.21 (m$_c$, 1H), 4.99 (m$_c$, 1H), 3.64-3.57 (m, 2H), 3.51 (m$_c$, 1H), 3.45 (d, J = 1.0 Hz, 3H), 1.59 (m$_c$, 1H), 1.52 (s, 3H), 1.35 (s, 3H), 1.25 (m$_c$, 1H), 0.95-0.91 (m, 1H), 0.69 (d, J = 7.0 Hz, 3H) ppm.

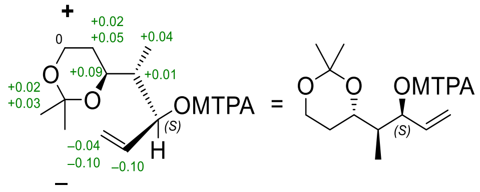

Figure 8. Mosher-analysis of allylic alcohol **(*S*)-42a**.

(*S*)-Mosher ester S32

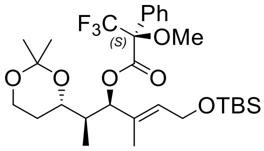

S32
$C_{29}H_{45}F_3O_6Si$
574.75

Following GP11, allylic alcohol **(*R*)-40a** (10 mg, 27.9 µmol, 1.0 equiv) and (*R*)-MTPACl gave (*S*)-mosher ester **S32** (15 mg, 26.1 µmol, 94%) as a yellow oil.

^{1}H NMR (400 MHz, C_6D_6): δ = 7.74-7.72 (m, 2H), 7.14-7.06 (m, 3H), 5.91 (d, J = 2.6 Hz, 1H), 5.66 (m_c, 1H), 4.12 (m_c, 2H), 3.65-3.60 (m, 2H), 3.59-3.57 (m, 1H), 3.50 (d, J = 0.9 Hz, 3H), 1.77 (m_c, 1H), 1.49 (s, 3H), 1.45 (s, 3H), 1.37-1.25 (m, 4H), 0.97-0.95 (m, 10H), 0.83 (d, J = 7.0 Hz, 3H), 0.055 (s, 3H), 0.046 (s, 3H) ppm.

(*R*)-Mosher ester S33

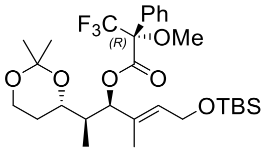

S33
$C_{29}H_{45}F_3O_6Si$
574.75

Following GP11, allylic alcohol **(*R*)-40a** (10 mg, 27.9 µmol, 1.0 equiv) and (*S*)-MTPACl gave (*R*)-mosher ester **S33** (16 mg, 27.8 µmol, ≥ 95%) as a yellow oil.

^{1}H NMR (400 MHz, C_6D_6): δ = 7.75-7.73 (m, 2H), 7.17-7.14 (m, 2H), 7.10-7.06 (m, 1H), 5.92 (d, J = 3.0 Hz, 1H), 5.75 (m_c, 1H), 4.14 (m_c, 2H), 3.64-3.60 (m, 2H), 3.53 (m_c, 1H), 3.48 (d, J = 0.9 Hz, 3H), 1.75 (m_c, 1H), 1.51 (s, 3H), 1.49 (s, 3H), 1.35 (s, 3H), 1.33-1.27 (m, 1H), 0.97-0.96 (m, 10H), 0.74 (d, J = 7.0 Hz, 3H), 0.06 (s, 6H) ppm.

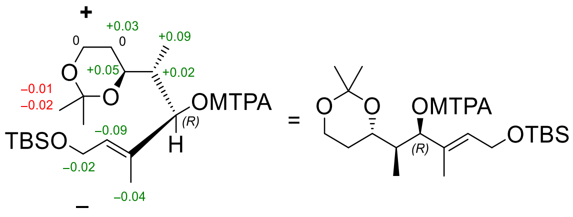

Figure 9. Mosher-analysis of allylic alcohol **(*R*)-40a**.

(*S*)-Mosher ester S34

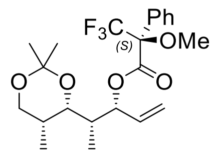

S34
$C_{22}H_{29}F_3O_5$
430.46

Following GP11, allylic alcohol **(*R*)-45a** (10 mg, 46.7 µmol, 1.0 equiv) and (*R*)-MTPACl gave (*S*)-mosher ester **S34** (19 mg, 44.1 µmol, ≥ 95%) after purification by flash column chromatography (PE:EtOAc 10:1) as a colorless oil.

^{1}H NMR (400 MHz, C_6D_6): δ = 7.68-7.65 (m, 2H), 7.09-7.00 (m, 3H), 5.66-5.57 (m, 2H), 5.21-5.15 (m, 1H), 5.01-4.96 (m, 1H), 3.74 (dd, J = 11.5, 2.6 Hz, 1H), 3.48 (dd, J = 9.5, 2.0 Hz, 1H), 3.42-3.41 (m, 3H), 3.40 (dd, J = 12.1, 1.4 Hz, 1H), 1.79-1.71 (m, 1H), 1.43 (s, 3H), 1.43-1.37 (m, 1H), 1.18 (s, 3H), 1.03 (d, J = 6.8 Hz, 6H) ppm.

(*R*)-Mosher ester S35

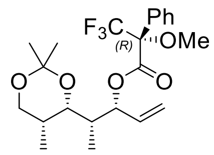

S35
$C_{22}H_{29}F_3O_5$
430.46

Following GP11, allylic alcohol **(*R*)-45a** (10 mg, 46.7 µmol, 1.0 equiv) and (*S*)-MTPACl gave (*R*)-mosher ester **S35** (19 mg, 44.1 µmol, ≥ 95%) after purification by flash column chromatography (PE:EtOAc 10:1) as a colorless oil.

^{1}H NMR (400 MHz, C_6D_6): δ = 7.67 (d, J = 7.6 Hz, 2H), 7.12-7.02 (m, 3H), 5.64-5.62 (m, 1H), 5.56-5.47 (m, 1H), 5.14-5.09 (m, 1H), 4.98-4-95 (m, 1H), 3.83 (dd, J = 11.6, 2.6 Hz, 1H), 3.74 (dd, J = 9.5, 2.1 Hz, 1H), 3.40 (dd, J = 13.7, 1.5 Hz, 1H), 3.39-3.38 (m, 3H), 1.81-1.73 (m, 1H), 1.49-1.42 (m, 1H), 1.46 (s, 3H), 1.26 (s, 3H), 1.10 (d, J = 6.7 Hz, 3H), 1.02 (d, J = 6.8 Hz, 3H) ppm.

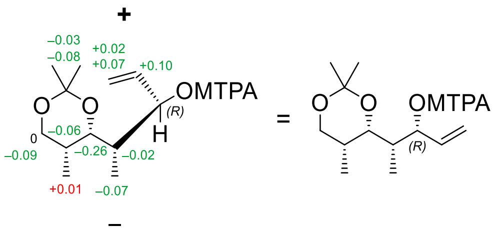

Figure 10. Mosher-analysis of allylic alcohol **(*R*)-45a**.

(*S*)-Mosher ester S36

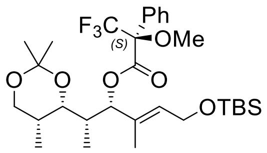

S36
$C_{30}H_{47}F_3O_6Si$
588.78

Following GP11, allylic alcohol **(*S*)-44a** (6.0 mg, 16.1 µmol, 1.0 equiv) and (*R*)-MTPACl gave (*S*)-mosher ester **S36** (8.0 mg, 13.6 µmol, 84%) after purification by flash column chromatography (cyclohexane:EtOAc 40:1) as a colorless oil.

^{1}H NMR (400 MHz, C_6D_6): δ = 7.70 (d, J = 7.7 Hz, 2H), 7.16-7.10 (m, 2H), 7.08-7.04 (m, 1H), 5.72 (t, J = 6.1 Hz, 1H), 5.44 (brs, 1H), 4.13 (d, J = 6.2 Hz, 2H), 3.76 (dd, J = 11.4, 2.6 Hz, 1H), 3.55 (dd, J = 9.6, 2.1 Hz, 1H), 3.52-3.51 (m, 3H), 3.42 (dd, J = 11.4, 1.6 Hz, 1H), 1.91-1.83 (m, 1H), 1.46 (s, 3H), 1.46-1.40 (m, 1H), 1.40 (s, 3H), 1.22 (s, 3H), 1.08 (d, J = 6.8 Hz, 3H), 1.06 (d, J = 6.6 Hz, 3H), 0.96 (s, 9H), 0.06 (s, 6H) ppm.

(*R*)-Mosher ester S37

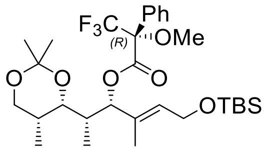

S37
$C_{30}H_{47}F_3O_6Si$
588.78

Following GP11, allylic alcohol **(*S*)-44a** (6.0 mg, 16.1 µmol, 1.0 equiv) and (*S*)-MTPACl gave (*R*)-mosher ester **S37** (8.0 mg, 13.6 µmol, 84%) after purification by flash column chromatography (cyclohexane:EtOAc 40:1 → 10:1) as a colorless oil.

^{1}H NMR (400 MHz, C_6D_6): δ = 7.69 (d, J = 7.7 Hz, 2H), 7.16-7.10 (m, 2H), 7.09-7.04 (m, 1H), 5.66 (t, J = 6.0 Hz, 1H), 5.46 (brs, 1H), 4.12 (d, J = 6.4 Hz, 2H), 3.87 (dd, J = 11.5, 2.6 Hz, 1H), 3.79 (dd, J = 9.5, 2.2 Hz, 1H), 3.47-3.47 (m, 3H), 3.43 (dd, J = 11.5, 1.6 Hz, 1H), 1.96-1.88 (m, 1H), 1.48 (s, 3H), 1.48-1.39 (m, 1H), 1.39 (s, 3H), 1.30 (s, 3H), 1.15 (d, J = 6.7 Hz, 3H), 1.07 (d, J = 6.8 Hz, 3H), 0.96 (s, 9H), 0.05 (s, 3H), 0.05 (s, 3H) ppm.

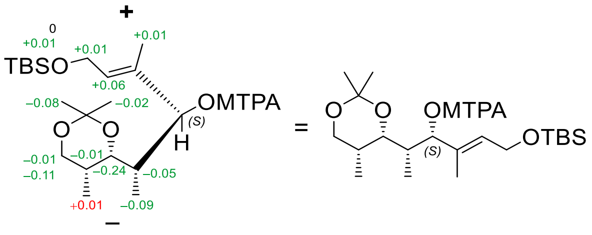

Figure 11. Mosher-analysis of allylic alcohol **(*S*)-44a**.

(*S*)-Mosher ester S38

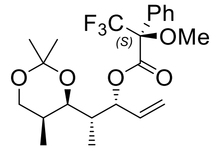

S38
$C_{22}H_{29}F_3O_5$
430.46

Following GP11, allylic alcohol **(*R*)-48a** (6.0 mg, 28.0 µmol, 1.0 equiv) and (*R*)-MTPACl gave (*S*)-mosher ester **S38** (12 mg, 27.9 µmol, ≥ 95%) after purification by flash column chromatography (PE:EtOAc 10:1) as a colorless oil.

^{1}H NMR (400 MHz, C_6D_6): δ = 7.72 (d, *J* = 7.6 Hz, 2H), 7.12-7.02 (m, 3H), 6.25 (dd, *J* = 6.3, 0.8 Hz, 1H), 5.70-5.61 (m, 1H), 5.21 (dt, *J* = 17.3, 1.3 Hz, 1H), 4.99 (dt, *J* = 10.7, 1.3 Hz, 1H), 3.77 (dd, *J* = 11.6, 2.3 Hz, 1H), 3.61 (dd, *J* = 10.1, 2.0 Hz, 1H), 3.45-3.45 (m, 3H), 3.39 (dd, *J* = 11.6, 1.3 Hz, 1H), 1.73-1.65 (m, 1H), 1.50 (s, 3H), 1.38 (s, 3H), 1.03-0.95 (m, 1H), 0.96 (d, *J* = 5.6 Hz, 3H), 0.69 (d, *J* = 7.2 Hz, 3H) ppm.

(*R*)-Mosher ester S39

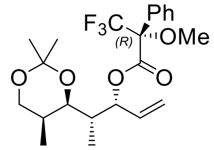

S39
$C_{22}H_{29}F_3O_5$
430.46

Following GP11, allylic alcohol **(*R*)-48a** (5.0 mg, 23.3 µmol, 1.0 equiv) and (*S*)-MTPACl gave (*R*)-mosher ester **S39** (10 mg, 23.2 µmol, ≥ 95%) after purification by flash column chromatography (PE:EtOAc 10:1) as a colorless oil.

^{1}H NMR (400 MHz, C_6D_6): δ = 7.76 (d, *J* = 7.5 Hz, 2H), 7.13-7.03 (m, 3H), 6.26 (dd, *J* = 5.5, 1.4 Hz, 1H), 5.59-5.50 (m, 1H), 5.11 (dt, *J* = 17.3, 1.4 Hz, 1H), 4.94 (dt, *J* = 10.7, 1.4 Hz, 1H), 3.76 (dd, *J* = 11.6, 2.5 Hz, 1H), 3.66 (dd, *J* = 10.2, 2.2 Hz, 1H), 3.43-3.40 (m, 4H), 1.74-1.66 (m, 1H), 1.48 (s, 3H), 1.34 (s, 3H), 1.08-1.02 (m, 1H), 0.98 (d, *J* = 6.2 Hz, 3H), 0.72 (d, *J* = 7.3 Hz, 3H) ppm.

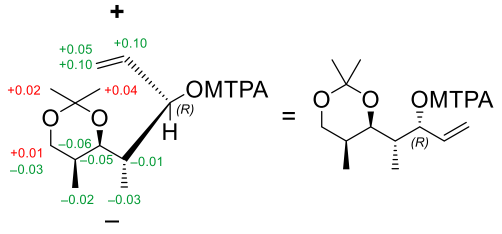

Figure 12. Mosher-analysis of allylic alcohol **(*R*)-48a**.

(*S*)-Mosher ester S40

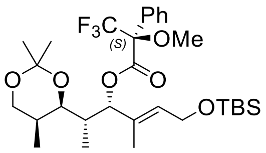

S40
$C_{30}H_{47}F_3O_6Si$
588.78

Following GP11, allylic alcohol **(*S*)-47a** (7.0 mg, 18.8 µmol, 1.0 equiv) and (*R*)-MTPACl gave (*S*)-mosher ester **S40** (11 mg, 18.7 µmol, ≥ 95%) as a yellow oil.

^{1}H NMR (400 MHz, C_6D_6): δ = 7.76 (d, J = 7.7 Hz, 2H), 7.19-7.14 (m, 2H), 7.11-7.07 (m, 1H), 5.97 (brs, 1H), 5.75 (tt, J = 6.1, 1.4 Hz, 1H), 4.17-4.14 (m, 2H), 3.78 (dd, J = 11.5, 2.3 Hz, 1H), 3.60 (dd, J = 9.9, 1.9 Hz, 1H), 3.50-3.49 (m, 3H), 3.40 (dd, J = 11.5, 1.3 Hz, 1H), 1.83-1.75 (m, 1H), 1.49 (s, 3H), 1.49 (s, 3H), 1.38 (s, 3H), 0.98-0.96 (m, 4H), 0.97 (s, 9H), 0.72 (d, J = 7.2 Hz, 3H), 0.06 (s, 6H) ppm.

(*R*)-Mosher ester S41

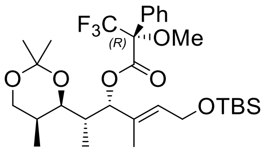

S41
$C_{30}H_{47}F_3O_6Si$
588.78

Following GP11, allylic alcohol **(*S*)-47a** (7.0 mg, 18.8 µmol, 1.0 equiv) and (*S*)-MTPACl gave (*R*)-mosher ester **S41** (11 mg, 18.7 µmol, ≥ 95%) as a yellow oil.

^{1}H NMR (400 MHz, C_6D_6): δ = 7.76 (d, J = 7.7 Hz, 2H), 7.16-7.12 (m, 2H), 7.11-7.09 (m, 1H), 5.98 (brs, 1H), 5.65 (tt, J = 6.1, 1.3 Hz, 1H), 4.19-4.15 (m, 1H), 4.12-4.07 (m, 1H), 3.77 (dd, J = 11.5, 2.5 Hz, 1H), 3.66 (dd, J = 10.1, 2.1 Hz, 1H), 3.52-3.51 (m, 3H), 3.41 (dd, J = 11.5, 1.1 Hz, 1H), 1.87-1.78 (m, 1H), 1.47 (s, 6H), 1.34 (s, 3H), 1.09-1.04 (m, 1H), 1.01-0.98 (m, 3H), 0.96 (s, 9H), 0.82 (d, J = 7.2 Hz, 3H), 0.06 (s, 3H), 0.05 (s, 3H) ppm.

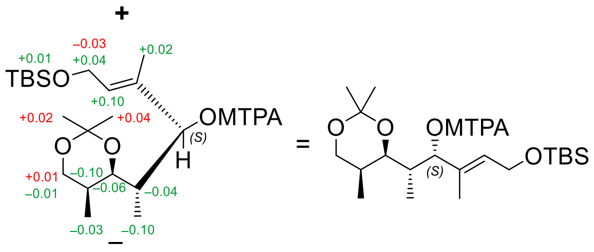

Figure 13. Mosher-analysis of allylic alcohol **(*S*)-47a**.

(*S*)-Mosher ester S42

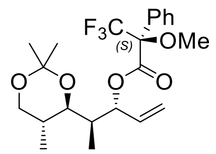

S42
$C_{22}H_{29}F_3O_5$
430.46

Following GP11, allylic alcohol **(*R*)-51b** (5.0 mg, 23.3 µmol, 1.0 equiv) and (*R*)-MTPACl gave (*S*)-mosher ester **S42** (9.0 mg, 20.9 µmol, 90%) after purification by flash column chromatography (PE:EtOAc 10:1) as a colorless oil.

^{1}H NMR (400 MHz, C_6D_6): δ = 7.73-7.70 (m, 2H), 7.13-7.08 (m, 2H), 7.06-7.02 (m, 1H), 5.71 (t, *J* = 9.0 Hz, 1H), 5.56-5.47 (m, 1H), 5.26 (dd, *J* = 17.1, 1.3 Hz, 1H), 5.02 (dd, *J* = 10.2, 1.3 Hz, 1H), 3.58-3.54 (m, 2H), 3.46-3.45 (m, 3H), 3.29 (t, *J* = 11.2 Hz, 1H), 1.86-1.78 (m, 1H), 1.72-1.61 (m, 1H), 1.50 (s, 3H), 1.36 (s, 3H), 0.82 (d, *J* = 7.1 Hz, 3H), 0.29 (d, *J* = 6.7 Hz, 3H) ppm.

(*R*)-Mosher ester S43

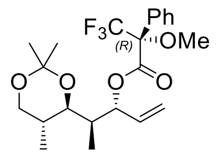

S43
$C_{22}H_{29}F_3O_5$
430.46

Following GP11, allylic alcohol **(*R*)-51b** (6.0 mg, 28.0 µmol, 1.0 equiv) and (*S*)-MTPACl gave (*R*)-mosher ester **S43** (12 mg, 27.9 µmol, ≥ 95%) after purification by flash column chromatography (PE:EtOAc 10:1) as a colorless oil.

^{1}H NMR (400 MHz, C_6D_6): δ = 7.75-7.71 (m, 2H), 7.13-7.03 (m, 3H), 5.68 (t, *J* = 8.6 Hz, 1H), 5.57-5.48 (m, 1H), 5.22-5.18 (m, 1H), 4.99 (dd, *J* = 10.3, 1.0 Hz, 1H), 3.67 (dd, *J* = 10.3, 1.5 Hz, 1H), 3.57 (dd, *J* = 11.4, 5.0 Hz, 1H), 3.44-3.43 (m, 3H), 3.30 (t, *J* = 11.4 Hz, 1H), 1.92-1.84 (m, 1H), 1.81-1.58 (m, 1H), 1.49 (s, 3H), 1.32 (s, 3H), 0.85 (d, *J* = 7.1 Hz, 3H), 0.36 (d, *J* = 6.7 Hz, 3H) ppm.

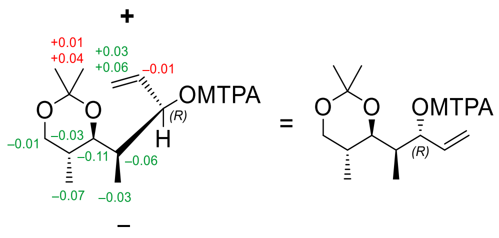

Figure 14. Mosher-analysis of allylic alcohol **(*R*)-51b**.

(*S*)-Mosher ester S44

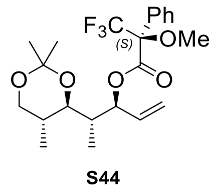

S44
$C_{22}H_{29}F_3O_5$
430.46

Following GP11, allylic alcohol **(*S*)-54b** (5.0 mg, 28.0 µmol, 1.0 equiv) and (*R*)-MTPACl gave (*S*)-mosher ester **S44** (10 mg, 23.2 µmol, ≥ 95%) after purification by flash column chromatography (PE:EtOAc 10:1) as a colorless oil.

^{1}H NMR (400 MHz, C_6D_6): δ = 7.73 (d, *J* = 7.8 Hz, 2H), 7.14-7.10 (m, 2H), 7.07-7.02 (m, 1H), 5.97-5.95 (m, 1H), 5.92-5.83 (m, 1H), 5.27-5.22 (m, 1H), 5.07-5.04 (m, 1H), 3.52 (dd, *J* = 11.4, 4.9 Hz, 1H), 3.45-3.44 (m, 3H), 3.23 (dd, *J* = 10.1, 3.5 Hz, 1H), 3.17 (dd, *J* = 11.4, 10.1 Hz, 1H), 2.16-2.09 (m, 1H), 1.92-1.81 (m, 1H), 1.41 (s, 3H), 1.19 (s, 3H), 0.94 (d, *J* = 7.2 Hz, 3H), 0.53 (d, *J* = 6.7 Hz, 3H) ppm.

(*R*)-Mosher ester S45

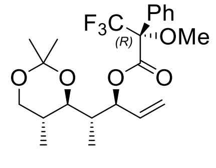

S45
$C_{22}H_{29}F_3O_5$
430.46

Following GP11, allylic alcohol **(*S*)-54b** (5.0 mg, 23.3 µmol, 1.0 equiv) and (*S*)-MTPACl gave (*R*)-mosher ester **S45** (10 mg, 23.2 µmol, ≥ 95%) after purification by flash column chromatography (PE:EtOAc 10:1) as a colorless oil.

^{1}H NMR (400 MHz, C_6D_6): δ = 7.71 (d, *J* = 7.9 Hz, 2H), 7.12-7.07 (m, 2H), 7.05-7.01 (m, 1H), 5.92-5.83 (m, 2H), 5.38-5.31 (m, 1H), 5.11-5.06 (m, 1H), 3.48 (dd, *J* = 11.4, 4.9 Hz, 1H), 3.46-3.45 (m, 3H), 3.19 (dd, *J* = 10.3, 3.4 Hz, 1H), 3.14 (dd, *J* = 11.4, 10.2 Hz, 1H), 2.15-2.07 (m, 1H), 1.78-1.66 (m, 1H), 1.41 (s, 3H), 1.16 (s, 3H), 0.86 (d, *J* = 7.1 Hz, 3H), 0.52 (d, *J* = 6.7 Hz, 3H) ppm.

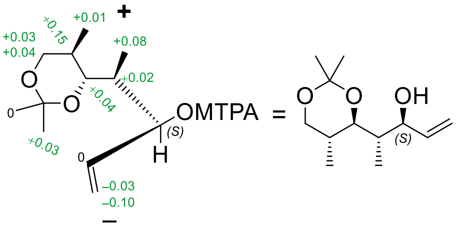

Figure 15. Mosher-analysis of allylic alcohol **(*S*)-54b**.

(*S*)-Mosher ester S46

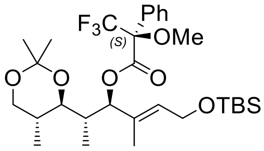

S46
$C_{30}H_{47}F_3O_6Si$
588.78

Following GP11, allylic alcohol **(*R*)-53b** (6.0 mg, 16.1 µmol, 1.0 equiv) and (*R*)-MTPACl gave (*S*)-mosher ester **S46** (8.0 mg, 13.6 µmol, 84%) after purification by flash column chromatography (PE:MTBE 20:1) as a colorless oil.

^{1}H NMR (400 MHz, C_6D_6): δ = 7.76-7.74 (m, 2H), 7.22-7.17 (m, 2H), 7.10-7.06 (m, 1H), 5.91-5.88 (m, 1H), 5.70 (d, *J* = 10.2 Hz, 1H), 4.14-4.12 (m, 2H), 3.53-3.52 (m, 3H), 3.48 (dd, *J* = 11.4, 4.9 Hz, 1H), 3.43 (dd, *J* = 10.4, 1.4 Hz, 1H), 3.20 (t, *J* = 11.4 Hz, 1H), 2.24-2.15 (m, 1H), 2.07-1.95 (m, 1H), 1.48 (s, 3H), 1.43 (d, *J* = 0.8 Hz, 3H), 1.27 (s, 3H), 0.97 (s, 9H), 0.86 (d, *J* = 7.0 Hz, 3H), 0.48 (d, *J* = 6.6 Hz, 3H), 0.07 (s, 3H), 0.06 (s, 3H) ppm.

(*R*)-Mosher ester S47

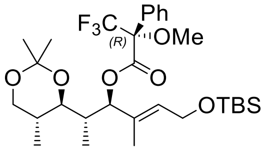

S47
$C_{30}H_{47}F_3O_6Si$
588.78

Following GP11, allylic alcohol **(*R*)-53b** (5.0 mg, 13.4 µmol, 1.0 equiv) and (*S*)-MTPACl gave (*R*)-mosher ester **S47** (7.0 mg, 11.9 µmol, 89%) after purification by flash column chromatography (PE:MTBE 20:1) as a colorless oil.

^{1}H NMR (400 MHz, C_6D_6): δ = 7.73 (d, *J* = 7.8 Hz, 2H), 7.16-7.10 (m, 2H), 7.06-7.02 (m, 1H), 5.95-5.91 (m, 1H), 5.67 (d, *J* = 10.4 Hz, 1H), 4.12 (d, *J* = 5.9 Hz, 2H), 3.51-3.50 (m, 3H), 3.39 (dd, *J* = 11.4, 5.0 Hz, 1H), 3.35 (dd, *J* = 10.7, 1.8 Hz, 1H), 3.14 (t, *J* = 11.4 Hz, 1H), 2.25-2.17 (m, 1H), 1.72-1.61 (m, 1H), 1.52 (d, *J* = 0.9 Hz, 3H), 1.45 (s, 3H), 1.17 (s, 3H), 0.96 (s, 9H), 0.85 (d, *J* = 7.1 Hz, 3H), 0.46 (d, *J* = 6.7 Hz, 3H), 0.06 (s, 3H), 0.05 (s, 3H) ppm.

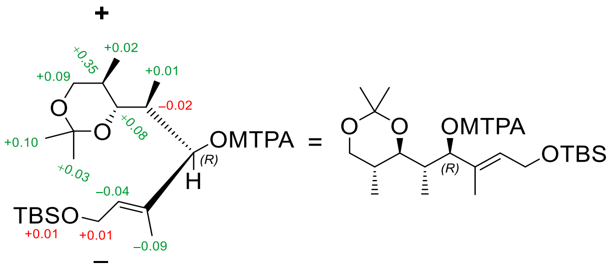

Figure 16. Mosher-analysis of allylic alcohol **(*R*)-53b**.

(*S*)-Mosher ester S48

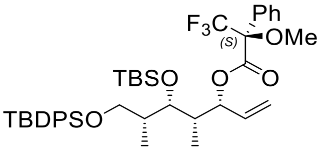

S48
$C_{41}H_{57}F_3O_5Si_2$
743.07

Following GP11, allylic alcohol **(*R*)-57a** (10 mg, 19.0 µmol, 1.0 equiv) and (*R*)-MTPACl gave (*S*)-mosher ester **S48** (12 mg, 16.2 µmol, 85%) as a yellow oil.

^{1}H NMR (400 MHz, C_6D_6): δ = 7.80-7.76 (m, 4H), 7.71 (d, J = 7.5 Hz, 2H), 7.29-7.21 (m, 6H), 7.04 (dd, J = 13.0, 7.3 Hz, 3H), 5.75-5.65 (m, 2H), 5.38 (dd, J = 16.3, 1.2 Hz, 1H), 5.10 (dd, J = 10.1, 1.5 Hz, 1H), 3.76 (t, J = 4.0 Hz, 1H), 3.67-3.63 (m, 1H), 3.54 (dd, J = 9.8, 7.1 Hz, 1H), 3.46 (d, J = 0.7 Hz, 3H), 1.96 (dt, J = 10.4, 6.8 Hz, 1H), 1.88 (td, J = 6.7, 4.4 Hz, 1H), 1.18 (s, 9H), 0.96-0.92 (m, 6H), 0.94 (s, 9H), −0.07 (s, 3H), −0.04 (s, 3H) ppm.

(*R*)-Mosher ester S49

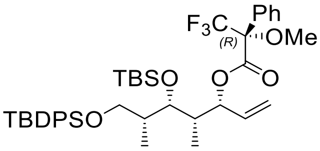

S49
$C_{41}H_{57}F_3O_5Si_2$
743.07

Following GP11, allylic alcohol **(*R*)-57a** (10 mg, 19.0 µmol, 1.0 equiv) and (*S*)-MTPACl gave (*R*)-mosher ester **S49** (11 mg, 14.8 µmol, 78%) as a yellow oil.

^{1}H NMR (400 MHz, C_6D_6): δ = 7.80-7.76 (m, 4H), 7.69 (d, J = 7.6 Hz, 2H), 7.27-7.22 (m, 6H), 7.06 (dd, J = 14.2, 7.3 Hz, 3H), 5.67-5.58 (m, 2H), 5.36-5.29 (m, 1H), 5.08 (dd, J = 9.5, 1.3 Hz, 1H), 3.85 (t, J = 3.9 Hz, 1H), 3.68 (dd, J = 9.9, 6.0 Hz, 1H), 3.55 (dd, J = 9.8, 6.9 Hz, 1H), 3.42 (d, J = 0.9 Hz, 3H), 1.98 (qd, J = 6.7, 3.5 Hz, 1H), 1.91 (td, J = 6.7, 4.3 Hz, 1H), 1.18 (s, 9H), 1.03 (d, J = 6.9 Hz, 3H), 0.96-0.94 (m, 3H), 0.95 (s, 9H), −0.09 (s, 3H), −0.01 (s, 3H) ppm.

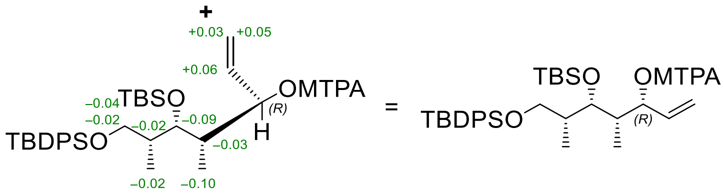

Figure 17. Mosher-analysis of allylic alcohol **(*R*)-57a**.

(*S*)-Mosher ester S50

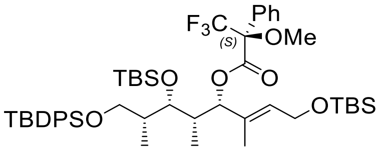

S50
$C_{49}H_{75}F_3O_6Si_3$
901.38

Following GP11, allylic alcohol **(*S*)-56a** (12 mg, 17.5 µmol, 1.0 equiv) and (*R*)-MTPACl gave (*S*)-mosher ester **S50** (15 mg, 16.6 µmol, ≥ 95%) as a yellow oil.

^{1}H NMR (400 MHz, C_6D_6): δ = 7.80-7.76 (m, 4H), 7.71-7.69 (m, 2H), 7.28-7.23 (m, 6H), 7.12-7.02 (m, 3H), 6.02 (m_c, 1H), 5.68 (d, J = 8.5 Hz, 1H), 4.19 (d, J = 6.0 Hz, 2H), 3.76 (m_c, 1H), 3.72 (m_c, 1H), 3.55 (dd, J = 9.7, 7.5 Hz, 1H), 3.48 (d, J = 0.9 Hz, 3H), 2.04-1.94 (m, 2H), 1.52 (s, 3H), 1.19 (s, 9H), 0.99-0.94 (m, 24H), 0.18 (s, 3H), 0.07 (s, 6H), 0.03 (s, 3H) ppm.

(*R*)-Mosher ester S51

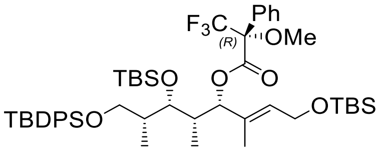

S51
$C_{49}H_{75}F_3O_6Si_3$
901.38

Following GP11, allylic alcohol **(*S*)-56a** (12 mg, 17.5 µmol, 1.0 equiv) and (*S*)-MTPACl gave (*R*)-mosher ester **S51** (13 mg, 14.4 µmol, 82%) as a yellow oil.

^{1}H NMR (400 MHz, C_6D_6): δ = 7.80-7.76 (m, 4H), 7.70-7.68 (m, 2H), 7.27-7.23 (m, 6H), 7.14-7.09 (m, 2H), 7.07-7.03 (m, 1H), 5.96 (m_c, 1H), 5.60 (d, J = 8.4 Hz, 1H), 4.17 (m_c, 2H), 3.80-3.76 (m, 2H), 3.57 (dd, J = 9.8, 7.5 Hz, 1H), 3.46 (d, J = 0.8 Hz, 3H), 2.07-1.96 (m, 2H), 1.35 (s, 3H), 1.19 (s, 9H), 1.07 (d, J = 6.8 Hz, 3H), 0.99-0.96 (m, 21H), 0.20 (s, 3H), 0.09 (s, 3H), 0.08 (s, 3H), 0.05 (s, 3H) ppm.

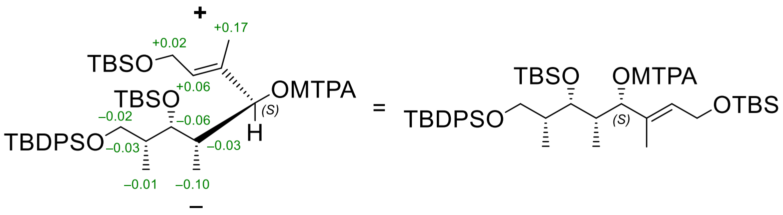

Figure 18. Mosher-analysis of allylic alcohol **(*S*)-56a**.

(*S*)-Mosher ester S52

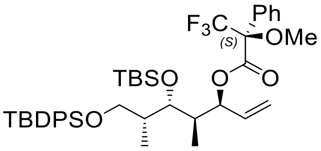

S52
$C_{41}H_{57}F_3O_5Si_2$
743.07

Following GP11, allylic alcohol **(*S*)-59a** (15 mg, 28.5 µmol, 1.0 equiv) and (*R*)-MTPACl gave (*S*)-mosher ester **S52** (19 mg, 25.6 µmol, 90%) as a yellow oil.

^{1}H NMR (400 MHz, C_6D_6): δ = 7.81-7.73 (m, 6H), 7.28-7.22 (m, 6H), 7.12-7.02 (m, 3H), 5.82-5.79 (m, 1H), 5.69 (m$_c$, 1H), 5.21 (m$_c$, 1H), 5.00 (m$_c$, 1H), 4.04 (dd, *J* = 7.5, 1.6 Hz, 1H), 3.70 (dd, *J* = 10.0, 7.9 Hz, 1H), 3.60 (dd, *J* = 10.0, 6.8 Hz, 1H), 3.47 (d, *J* = 1.1 Hz, 3H), 2.01-1.93 (m, 1H), 1.92-1.85 (m, 1H), 1.19 (s, 9H), 0.98 (d, *J* = 7.0 Hz, 3H), 0.96 (m, 9H), 0.85 (d, *J* = 6.8 Hz, 3H), 0.16 (s, 3H), 0.05 (s, 3H) ppm.

(*R*)-Mosher ester S53

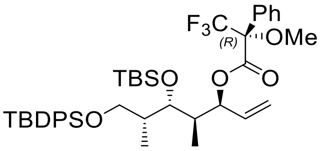

S53
$C_{41}H_{57}F_3O_5Si_2$
743.07

Following GP11, allylic alcohol **(*S*)-59a** (15 mg, 28.5 µmol, 1.0 equiv) and (*S*)-MTPACl gave (*R*)-mosher ester **S53** (15 mg, 20.2 µmol, 71%) as a yellow oil.

^{1}H NMR (400 MHz, C_6D_6): δ = 7.81-7.78 (m, 4H), 7.74-7.72 (m, 2H), 7.29-7.22 (m, 6H), 7.11-7.01 (m, 3H), 5.84-5.75 (m, 2H), 5.37-5.31 (m, 1H), 5.07-5.03 (m, 1H), 3.99 (dd, *J* = 7.1, 1.6 Hz, 1H), 3.68 (dd, *J* = 9.9, 7.9 Hz, 1H), 3.58 (dd, *J* = 9.9, 6.9 Hz, 1H), 3.47 (d, *J* = 0.9 Hz, 3H), 1.99-1.93 (m, 1H), 1.92-1.88 (m, 1H), 1.20 (s, 9H), 0.95-0.93 (m, 12H), 0.85 (d, *J* = 6.8 Hz, 3H), 0.15 (s, 3H), 0.05 (s, 3H) ppm.

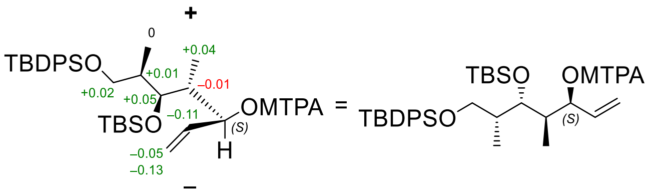

Figure 19. Mosher-analysis of allylic alcohol **(*S*)-59a**.

(*S*)-Mosher ester S54

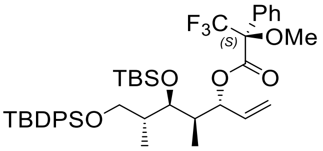

S54
$C_{41}H_{57}F_3O_5Si_2$
743.07

Following GP11, allylic alcohol **(*R*)-62b** (10 mg, 19.0 µmol, 1.0 equiv) and (*R*)-MTPACl gave (*S*)-mosher ester **S54** (13 mg, 18.7 µmol, 92%) as a yellow oil.

^{1}H NMR (400 MHz, C_6D_6): δ = 7.68-7.62 (m, 10H), 7.44-7.29 (m, 5H), 5.68 (m$_c$, 1H), 5.43-5.34 (m, 1H), 5.34-5.30 (m, 1H), 5.22 (t, *J* = 8.7 Hz, 1H), 3.74 (dd, *J* = 9.7, 5.0 Hz, 1H), 3.52-3.49 (brs, 3H), 3.42 (dd, *J* = 6.5, 1.9 Hz, 1H), 3.23 (dd, *J* = 9.8, 8.7 Hz, 1H), 1.96 (m$_c$, 1H), 1.80 (m$_c$, 1H), 1.05 (s, 9H), 0.82 (d, *J* = 6.7 Hz, 3H), 0.79 (s, 9H), 0.75 (d, *J* = 7.1 Hz, 3H), 0.01 (s, 3H), −0.17 (s, 3H) ppm.

(*R*)-Mosher ester S55

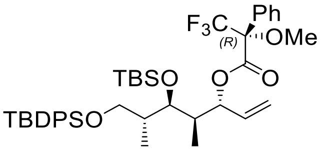

S55
$C_{41}H_{57}F_3O_5Si_2$
743.07

Following GP11, allylic alcohol **(*R*)-62b** (10 mg, 19.0 µmol, 1.0 equiv) and (*S*)-MTPACl gave (*R*)-mosher ester **S55** (13 mg, 18.7 µmol, 90%) as a yellow oil.

^{1}H NMR (400 MHz, C_6D_6): δ = 7.68-7.62 (m, 4H), 7.42-7.32 (m, 2H), 7.44-7.29 (m, 9H), 5.62 (m$_c$, 1H), 5.35-5.24 (m, 2H), 5.22 (t, *J* = 8.7 Hz, 1H), 3.78 (dd, *J* = 9.9, 5.0 Hz, 1H), 3.55 (dd, *J* = 6.5, 1.9 Hz, 1H), 3.47-3.43 (brs, 3H), 3.31 (dd, *J* = 9.9, 8.7 Hz, 1H), 2.00 (m$_c$, 1H), 1.88 (m$_c$, 1H), 1.05 (s, 9H), 0.92 (d, *J* = 6.8 Hz, 3H), 0.81-0.77 (m, 12H), −0.02 (s, 3H), −0.16 (s, 3H) ppm.

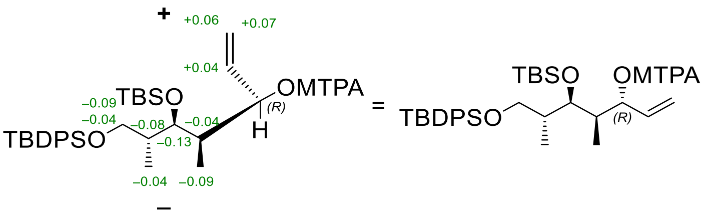

Figure 20. Mosher-analysis of allylic alcohol **(*R*)-62b**.

(*S*)-Mosher ester S56

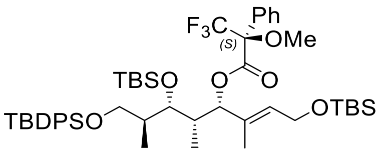

S56
$C_{49}H_{75}F_3O_6Si_3$
901.38

Following GP11, allylic alcohol **(*S*)-61a** (10 mg, 14.6 µmol, 1.0 equiv) and (*R*)-MTPACl gave (*S*)-mosher ester **S56** (13 mg, 14.4 µmol, ≥ 95%) as a yellow oil.

^{1}H NMR (400 MHz, C_6D_6): δ = 7.76-7.72 (m, 6H), 7.27-7.22 (m, 6H), 7.14-7.10 (m, 2H), 7.08-7.04 (m, 1H), 6.09 (m_c, 1H), 5.74 (d, J = 9.9 Hz, 1H), 4.23 (m_c, 2H), 3.76 (m_c, 1H), 3.70 (dd, J = 10.3, 5.8 Hz, 1H), 3.53 (dd, J = 10.3, 6.5 Hz, 1H), 3.50 (d, J = 1.0 Hz, 3H), 2.07 (m_c, 1H), 1.91 (m_c, 1H), 1.57 (d, J = 0.9 Hz, 3H), 1.18 (s, 9H), 0.98 (s, 9H), 0.96 (s, 9H), 0.90 (m_c, 6H), 0.22 (s, 3H), 0.08 (s, 6H), 0.01 (s, 3H) ppm.

(*R*)-Mosher ester S57

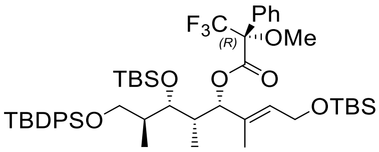

S57
$C_{49}H_{75}F_3O_6Si_3$
901.38

Following GP11, allylic alcohol **(*S*)-61a** (10 mg, 14.6 µmol, 1.0 equiv) and (*S*)-MTPACl gave (*R*)-mosher ester **S57** (11 mg, 12.2 µmol, 84%) as a yellow oil.

^{1}H NMR (400 MHz, C_6D_6): δ = 7.75-7.70 (m, 6H), 7.25-7.22 (m, 6H), 7.14-7.12 (m, 2H), 7.09-7.04 (m, 1H), 6.05 (m_c, 1H), 5.65 (d, J = 9.9 Hz, 1H), 4.22 (m_c, 2H), 3.78 (m_c, 1H), 3.73 (dd, J = 10.3, 5.7 Hz, 1H), 3.54 (dd, J = 10.2, 6.7 Hz, 1H), 3.47 (d, J = 0.9 Hz, 3H), 2.10 (m_c, 1H), 1.94 (m_c, 1H), 1.36 (d, J = 0.9 Hz, 3H), 1.17 (s, 9H), 1.11 (d, J = 6.7 Hz, 3H), 0.99 (s, 9H), 0.96 (s, 9H), 0.94 (d, J = 7.1 Hz, 3H), 0.22 (s, 3H), 0.10 (s, 3H), 0.09 (s, 3H), 0.02 (s, 3H) ppm.

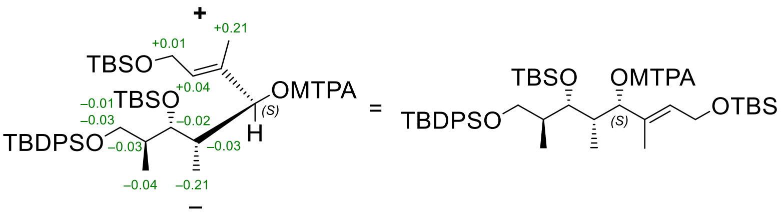

Figure 21. Mosher-analysis of allylic alcohol **(*S*)-61a**.

(*S*)-Mosher ester S58

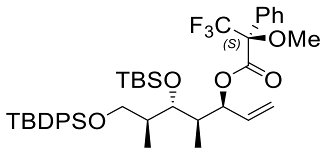

S58

$C_{41}H_{57}F_3O_5Si_2$

743.07

Following GP11, allylic alcohol **(*S*)-65a** (12 mg, 22.8 µmol, 1.0 equiv) and (*R*)-MTPACl gave (*S*)-mosher ester **S58** (14 mg, 18.8 µmol, 82%) as a yellow oil.

^{1}H NMR (400 MHz, C_6D_6): δ = 7.81-7.77 (m, 4H), 7.74-7.72 (m, 2H), 7.29-7.21 (m, 6H), 7.13-7.02 (m, 3H), 5.82-5.79 (m, 1H), 5.61 (m$_c$, 1H), 5.21 (m$_c$, 1H), 5.00 (m$_c$, 1H), 3.98 (dd, *J* = 10.1, 5.5 Hz, 1H), 3.70 (dd, *J* = 6.8, 3.1 Hz, 1H), 3.64 (dd, *J* = 10.0, 8.0 Hz, 1H), 3.44 (d, *J* = 1.0 Hz, 3H), 2.19-2.10 (m, 1H), 2.01-1.91 (m, 1H), 1.19 (s, 9H), 1.06 (d, *J* = 6.9 Hz, 3H), 0.96 (d, *J* = 7.0 Hz, 3H), 0.91 (s, 9H), 0.08 (s, 3H), 0.01 (s, 3H) ppm.

(*R*)-Mosher ester S59

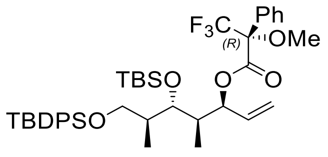

S59

$C_{41}H_{57}F_3O_5Si_2$

743.07

Following GP11, allylic alcohol **(*S*)-65a** (14 mg, 26.6 µmol, 1.0 equiv) and (*S*)-MTPACl gave (*R*)-mosher ester **S59** (19 mg, 25.6 µmol, ≥ 95%) as a yellow oil.

^{1}H NMR (400 MHz, C_6D_6): δ = 7.81-7.78 (m, 4H), 7.73-7.71 (m, 2H), 7.28-7.23 (m, 6H), 7.12-7.01 (m, 3H), 5.84-5.79 (m, 1H), 5.71 (m$_c$, 1H), 5.32 (m$_c$, 1H), 5.04 (m$_c$, 1H), 3.96 (dd, *J* = 10.1, 5.3 Hz, 1H), 3.64-3.58 (m, 2H), 3.46 (d, *J* = 0.9 Hz, 3H), 2.16-2.06 (m, 1H), 2.01-1.91 (m, 1H), 1.19 (s, 9H), 1.05 (d, *J* = 7.0 Hz, 3H), 0.90 (s, 9H), 0.88 (d, *J* = 7.1 Hz, 3H), 0.10 (s, 3H), 0.01 (s, 3H) ppm.

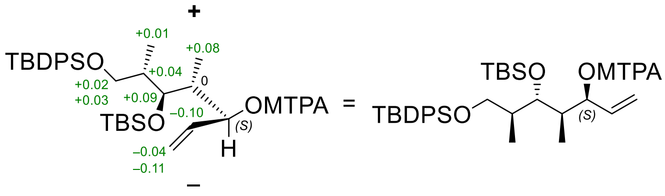

Figure 22. Mosher-analysis of allylic alcohol **(*S*)-65a**.

(*S*)-Mosher ester S60

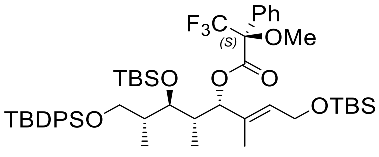

S60
$C_{49}H_{75}F_3O_6Si_3$
901.38

Following GP11, allylic alcohol **(*S*)-64a** (10 mg, 14.6 µmol, 1.0 equiv) and (*R*)-MTPACl gave (*S*)-mosher ester **S60** (12 mg, 13.3 µmol, 91%) as a yellow oil.

^{1}H NMR (400 MHz, C_6D_6): δ = 7.84-7.80 (m, 4H), 7.73-7.71 (m, 2H), 7.32-7.24 (m, 6H), 7.13-7.12 (m, 2H), 7.07-7.06 (m, 1H), 5.83 (m$_c$, 1H), 5.65 (d, J = 7.0 Hz, 1H), 4.12 (d, J = 6.1 Hz, 2H), 4.07 (dd, J = 10.2, 4.9 Hz, 1H), 3.66-3.64 (m, 1H), 3.63-3.60 (m, 1H), 3.48 (d, J = 0.9 Hz, 3H), 2.26-2.06 (m, 2H), 1.62 (s, 3H), 1.20 (s, 9H), 1.17 (d, J = 7.0 Hz, 3H), 0.97 (s, 9H), 0.89 (s, 9H), 0.88 (d, J = 6.8 Hz, 3H), 0.07 (s, 3H), 0.063 (s, 3H), 0.059 (s, 3H), 0.02 (s, 3H) ppm.

(*R*)-Mosher ester S61

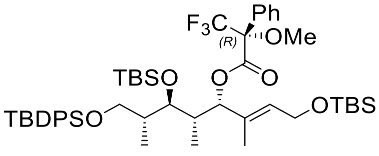

S61
$C_{49}H_{75}F_3O_6Si_3$
901.38

Following GP11, allylic alcohol **(*S*)-64a** (10 mg, 14.6 µmol, 1.0 equiv) and (*S*)-MTPACl gave (*R*)-mosher ester **S61** (11 mg, 12.2 µmol, 84%) as a yellow oil.

^{1}H NMR (400 MHz, C_6D_6): δ = 7.85-7.81 (m, 4H), 7.73-7.72 (m, 2H), 7.30-7.24 (m, 6H), 7.14-7.05 (m, 3H), 5.74 (m$_c$, 1H), 5.58 (d, J = 6.6 Hz, 1H), 4.12-4.10 (m, 2H), 4.08 (m$_c$, 1H), 3.69-3.65 (m, 2H), 3.50 (s, 3H), 2.24-2.09 (m, 2H), 1.48 (s, 3H), 1.20 (s, 9H), 1.17 (d, J = 7.0 Hz, 3H), 0.99 (d, J = 7.1 Hz, 3H), 0.97 (s, 9H), 0.90 (s, 9H), 0.07 (s, 3H), 0.06 (s, 3H), 0.04 (s, 3H), 0.01 (s, 3H) ppm.

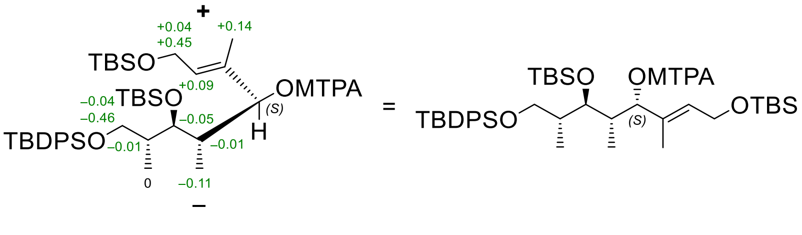

Figure 23. Mosher-analysis of allylic alcohol **(*S*)-64a**.

2-12. Rationalization of substrate control

General Procedure 13 (GP13): Stannanes

Note: Since the scale of the performed reactions differs due to need and availability, this general procedure reports the relative molar quantities, equivalents and relative solvent volumes of a representative experiment, whereby the exact volumes/weights of the structure-building (or key) chemicals are listed in the corresponding individual procedure.

The required TIB ester or carbamate (0.54 mmol, 1.0 equiv) and diamine (0.80 mmol, 1.5 equiv) were dissolved in Et_2O (2.7 mL, 0.2 M). The solution was cooled to −78 °C, *s*BuLi (1.3 M in hexanes, 0.80 mmol, 1.5 equiv) was added and the reaction mixture was stirred for 5 h at this temperature. Then a freshly prepared solution of Me_3SnCl (0.92 mmol, 1.7 equiv) in Et_2O (0.92 mL, 1.0 M refers to Me_3SnCl) was added dropwise and the reaction mixture was stirred for 1 h at −78 °C before being warmed to rt. After stirring overnight at rt sat. aq. NH_4Cl was added and the biphasic mixture was stirred for further 15 min. The phases were separated, the organic layer was washed with sat. aq. NH_4Cl (3x) and the combined aqueous phases were extracted with MTBE (3x). The combined organic phases were dried over Na_2SO_4 and concentrated *in vacuo* and the crude material was purified by flash column chromatography.

General Procedure 14 (GP14): Transmetalation of TIB-derived stannanes

Note: Since the scale of the performed reactions differs due to need and availability, this general procedure reports the relative molar quantities, equivalents and relative solvent volumes of a representative experiment, whereby the exact volumes/weights of the structure-building (or key) chemicals are listed in the corresponding individual procedure.

To a stirred solution of stannane (0.24 mmol, 1.5 equiv) in Et_2O (1.2 mL, 0.2 M refers to stannane) at −78 °C was added *n*BuLi (2.5 M in hexanes, 0.26 mmol, 1.65 equiv). The reaction mixture was stirred for 1 h at that temperature before TMEDA (0.24 mmol, 1.5 equiv) was added. Stirring was continued at −78 °C for 20 min before a solution of vinyl boronic ester (0.16 mmol, 1.0 equiv) in Et_2O (0.32 mL, 0.5 M) was added. After stirring for further 3 h at −78 °C, the reaction mixture was warmed to 45 °C and stirred overnight. The reaction mixture was cooled to rt, sat. aq. NH_4Cl was added and the biphasic mixture was stirred for 15 min. The phases were separated, the organic layer was washed with sat. aq. NH_4Cl (3x) and the combined aqueous phases were extracted with MTBE (3x). The combined organic phases were dried over Na_2SO_4, concentrated *in vacuo* and the crude material was purified by a short flash column chromatography (to remove TIBOH).
The residue was dissolved in THF (0.80 mL, 0.2 M) and cooled to −20 °C. A premixed, ice-cooled solution of NaOH (2.0 M)/H_2O_2 (35%, 2/1 v/v, 1.3 mL, 0.12 M) was added dropwise. The reaction mixture was stirred at rt before being diluted with MTBE and quenched by the slow addition of sat. aq. $Na_2S_2O_3$ at 0 °C after TLC showed full conversion. The solution was diluted with MTBE, the phases were separated and the aqueous phase was extracted with MTBE (3x). The combined organic layers were dried over Na_2SO_4 and concentrated *in vacuo*. The crude product was purified by flash column chromatography to afford allylic alcohol.

General Procedure 15 (GP15): Transmetalation of Cb-derived stannanes

Note: Since the scale of the performed reactions differs due to need and availability, this general procedure reports the relative molar quantities, equivalents and relative solvent volumes of a representative experiment, whereby the exact volumes/weights of the structure-building (or key) chemicals are listed in the corresponding individual procedure.

To a stirred solution of stannane (0.24 mmol, 1.5 equiv) in Et_2O (1.2 mL, 0.2 M refers to stannane) at −78 °C was added *n*BuLi (2.5 M in hexanes, 0.26 mmol, 1.65 equiv). The reaction mixture was stirred for 1 h at that temperature before TMEDA (0.24 mmol, 1.5 equiv) was added. Stirring was continued at −78 °C for 20 min before a solution of vinyl boronic ester (0.16 mmol, 1.0 equiv) in Et_2O (0.5 M) was added. The reaction mixture was stirred for 3 h at −78 °C.

In parallel, magnesium turnings were activated (2x 1.0 M HCl, 2x H_2O, 2x acetone, drying under high vacuum). The required amount (0.32 mmol, 2.0 equiv) was dissolved in Et_2O (0.40 mL, 0.8 M refers to Mg turnings) and 1,2-dibromoethane (0.32 mmol, 2.0 equiv) was added under water bath cooling. The reaction mixture was stirred for 2 h at this temperature.

The biphasic $MgBr_2·OEt_2$ solution was added dropwise to the main reaction mixture, which was then stirred for another 30 min at −78 °C before being warmed to 45 °C and stirred overnight. The reaction mixture was cooled to rt, sat. aq. NH_4Cl was added and the biphasic mixture was stirred for 15 min. The phases were separated, the organic layer was washed with sat. aq. NH_4Cl (3x) and the combined aqueous phases were extracted with MTBE (3x). The combined organic phases were dried over Na_2SO_4 and concentrated *in vacuo* and the crude material was purified by a short flash column chromatography (to remove excess of the carbamate).

The residue was dissolved in THF (0.80 mL, 0.2 M) and cooled to −20 °C. A premixed, ice-cooled solution of NaOH (2.0 M)/H_2O_2 (35%, 2/1 v/v, 1.3 mL, 0.12 M) was added dropwise. The reaction mixture was stirred at rt before being diluted with MTBE and quenched by the slow addition of sat. aq. $Na_2S_2O_3$ at 0 °C after TLC showed full conversion. The solution was diluted with MTBE, the phases were separated and the aqueous phase was extracted with MTBE (3x). The combined organic layers were dried over Na_2SO_4 and concentrated *in vacuo*. The crude product was purified by flash column chromatography to afford allylic alcohol.

General Procedure 16 (GP16): Deuteration of acetonide TIB esters

Note: Since the scale of the performed reactions differs due to need and availability, this general procedure reports the relative molar quantities, equivalents and relative solvent volumes of a representative experiment, whereby the exact volumes/weights of the structure-building (or key) chemicals are listed in the corresponding individual procedure.

To a stirred solution of acetonide TIB ester (128 µmol, 1.0 equiv) and TMEDA (192 µmol, 1.5 equiv) in Et_2O (0.64 mL, 0.2 M) at –78 °C was added *s*BuLi (1.3 M in hexanes, 179 µmol, 1.4 equiv).[10] The reaction mixture was stirred for 5 h at that temperature before methanol-d_4 (25.6 mmol, 200 equiv) was added. After warming to rt, sat. aq. NH_4Cl was added, and the biphasic mixture was stirred for 15 min. The phases were separated, the organic layer was washed with sat. aq. NH_4Cl (3x) and the combined aqueous phases were extracted with MTBE (3x). The combined organic phases were dried over Na_2SO_4 and concentrated in vacuo to afford the crude deuterated acetonide TIB ester.

General Procedure 17 (GP17): Deuteration of Carbamates

Note: Since the scale of the performed reactions differs due to need and availability, this general procedure reports the relative molar quantities, equivalents and relative solvent volumes of a representative experiment, whereby the exact volumes/weights of the structure-building (or key) chemicals are listed in the corresponding individual procedure.

To a stirred solution of carbamate (189 µmol, 1.0 equiv) and TMEDA (321 µmol, 1.7 equiv) in Et_2O (0.95 mL, 0.2 M) at –78 °C was added *s*BuLi (1.3 M in hexanes, 302 µmol, 1.6 equiv).[10] The reaction mixture was stirred for 5 h at that temperature before methanol-d_4 (37.8 mmol, 200 equiv) was added. After warming to rt, sat. aq. NH_4Cl was added, and the biphasic mixture was stirred for 15 min. The phases were separated, the organic layer was washed with sat. aq. NH_4Cl (3x) and the combined aqueous phases were extracted with MTBE (3x). The combined organic phases were dried over Na_2SO_4 and concentrated *in vacuo* to afford the deuterated carbamate.

[10] To enable significant lithiation.

Stannane 23

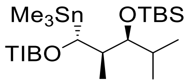

23
$C_{32}H_{60}O_3SiSn$
639.62

According to GP13, TIB ester **9a** (250 mg, 0.52 mmol, 1.0 equiv) and (+)-sparteine (0.18 mL, 0.79 mmol, 1.5 equiv) gave stannane (+)-sp-**23** (260 mg, 0.41 mmol, 79%, dr ≥ 19:1) after purification by flash column chromatography (PE:MTBE 200:1 → 150:1) as a colorless oil. According to GP13, TIB ester **9a** (158 mg, 0.33 mmol, 1.0 equiv) and TMEDA (0.08 mL, 0.50 mmol, 1.5 equiv) gave stannane TMEDA-**23** (173 mg, 0.27 mmol, 82%, dr 1.4:1) after purification by flash column chromatography (PE:MTBE 200:1 → 150:1) as a colorless oil.

Analytical data are given for stannane (+)-sp-**23** obtained by the reaction of TIB ester **9** and (+)-sparteine (dr ≥ 19:1).

^{1}H NMR (400 MHz, C_6D_6): δ = 7.08 (s, 2H), 5.16 (m$_c$, 1H), 3.71 (dd, J = 8.2, 2.2 Hz, 1H), 3.12 (sep, J = 6.8 Hz, 2H), 2.72 (sep, J = 6.9 Hz, 1H), 2.20 (m$_c$, 1H), 2.10 (m$_c$, 1H), 1.34-1.29 (m, 15H), 1.16 (d, J = 6.9 Hz, 6H), 1.01-0.98 (m, 12H), 0.94 (d, J = 6.7 Hz, 3H), 0.36 (m$_c$, 9H), 0.02 (s, 3H), 0.01 (s, 3H) ppm;

^{13}C{^{1}H} NMR (101 MHz, C_6D_6): δ = 171.8, 150.4, 145.7, 131.4, 121.2, 79.4, 75.6, 41.3, 34.8, 32.1, 31.2, 26.5, 24.7, 24.6, 24.1, 21.5, 18.8, 16.3, 15.7, −3.1, −3.3, −7.5 ppm;

HRMS (ESI): $C_{32}H_{60}O_3SiSnNa$ $[M+Na]^+$ calculated: 663.3231, found: 663.3231;

R_f = 0.4 (PE:MTBE 98:2, uv, vanillin);

$[\alpha]_D^{20}$ = −39.9 (c 0.7, $CHCl_3$).

Stannane S62

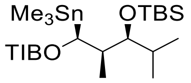

S62
$C_{32}H_{60}O_3SiSn$
639.62

According to GP13, TIB ester **9a** (175 mg, 0.37 mmol, 1.0 equiv) and (−)-sparteine (0.13 mL, 0.55 mmol, 1.5 equiv) gave stannane (−)-sp-**S62** (198 mg, contaminated with 51% TIB ester, 0.15 mmol, 41%, dr 4:1)[11] after purification by flash column chromatography (PE:MTBE 200:1 → 150:1) as a colorless oil.

^{1}H NMR (400 MHz, C_6D_6): δ = 7.10 (s, 2H), 5.15 (m$_c$, 1H), 3.64 (m$_c$, 1H), 3.24-3.19 (m, 2H), 2.77-2.70 (m, 1H), 2.61 (m$_c$, 1H), 1.80 (m$_c$, 1H), 1.36-1.33 (m, 12H), 1.17 (d, J = 6.9 Hz, 6H), 1.14 (d, J = 6.9 Hz, 3H), 1.04 (d, J = 6.7 Hz, 3H), 1.01 (s, 9H), 0.92 (d, J = 6.9 Hz, 3H), 0.35 (m$_c$, 9H), 0.19 (s, 3H), 0.05 (s, 3H) ppm;

[11] This mixture was only used to determine the stereochemistry of the formed stannane.

$^{13}C\{^1H\}$ **NMR** (101 MHz, C_6D_6): δ = 171.2, 150.5, 146.2, 131.0, 121.4, 77.8, 75.3, 41.1, 34.8, 33.1, 31.9, 26.6, 24.9, 24.7, 24.1, 20.1, 18.9, 18.4, 14.5, −3.1, −3.5, −7.0 ppm;

HRMS (ESI): $C_{32}H_{60}O_3SiSnNa$ $[M+Na]^+$ calculated: 663.3231, found: 663.3233;

$\mathbf{R_f}$ = 0.3 (PE:MTBE 98:2, uv, vanillin).

Stannane S63

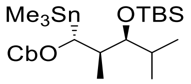

S63
$C_{23}H_{51}NO_3SiSn$
536.46

According to GP13, carbamate **9b** (200 mg, 0.54 mmol, 1.0 equiv) and (+)-sparteine (0.18 mL, 0.80 mmol, 1.5 equiv) gave stannane (+)-sp-**S63** (125 mg, 0.23 mmol, 43%, dr ≥ 19:1) after purification by flash column chromatography (PE:MTBE 100:1) as a colorless oil.

^{1}**H NMR** (400 MHz, C_6D_6): δ = 4.69 (m_c, 1H), 3.85-3.68 (m, 3H), 2.25 (m_c, 1H), 1.97 (m_c, 1H), 1.27 (d, J = 7.0 Hz, 3H), 1.10-1.06 (m, 12H), 1.03 (s, 9H), 0.99 (d, J = 7.0 Hz, 3H), 0.93 (d, J = 6.8 Hz, 3H), 0.34 (m_c, 9H), 0.18 (s, 3H), 0.14 (s, 3H) ppm;

$^{13}C\{^1H\}$ **NMR** (101 MHz, C_6D_6): δ = 156.5, 79.9, 74.7, 46.1 (brs), 41.4, 31.5, 26.5, 21.5, 21.3 (brs), 20.7 (brs), 18.9, 16.3, 16.1, −3.1, −3.3, −7.3 ppm;

HRMS (ESI): $C_{23}H_{51}NO_3SiSnNa$ $[M+Na]^+$ calculated: 560.2558, found: 560.2560;

$\mathbf{R_f}$ = 0.5 (PE:MTBE 95:5, vanillin);

$[\boldsymbol{\alpha}]_{\mathbf{D}}^{\mathbf{20}}$ = −40.4 (c 0.9, $CHCl_3$).

Stannane 24

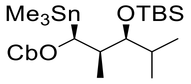

24
$C_{23}H_{51}NO_3SiSn$
536.46

According to GP13, carbamate **9b** (175 mg, 0.47 mmol, 1.0 equiv) and (−)-sparteine (0.16 mL, 0.70 mmol, 1.5 equiv) gave stannane (−)-sp-**24** (154 mg, 0.29 mmol, 62%, dr ≥ 19:1) after purification by flash column chromatography (PE:MTBE 100:1) as a colorless oil.
According to GP13, carbamate **9b** (200 mg, 0.54 mmol, 1.0 equiv) and TMEDA (0.12 mL, 0.80 mmol, 1.5 equiv) gave stannane TMEDA-**24** (174 mg, 0.32 mmol, 59%, dr 5:1) after purification by flash column chromatography (PE:MTBE 100:1) as a colorless oil.

Analytical data are given for stannane (−)-sp-**24** obtained by the reaction of carbamate **9b** and (−)-sparteine (dr ≥ 19:1).

^{1}**H NMR** (400 MHz, C_6D_6): δ = 4.32 (m_c, 1H), 3.91 (brs, 1H), 3.77 (dd, J = 6.8, 1.2 Hz, 1H), 3.61 (brs, 1H), 2.63 (m_c, 1H), 1.77 (m_c, 1H), 1.16 (d, J = 6.7 Hz, 6H), 1.08-1.03 (m, 15H), 1.01

(d, J = 6.9 Hz, 3H), 0.98 (d, J = 6.8 Hz, 3H), 0.90 (d, J = 6.8 Hz, 3H), 0.33 (m_c, 9H), 0.25 (s, 3H), 0.16 (s, 3H) ppm;

$^{13}C\{^1H\}$ NMR (101 MHz, C_6D_6): δ = 155.8, 76.5, 75.0, 46.0, 45.9, 38.4, 33.8, 26.5, 21.3 (brs), 20.7 (brs), 20.2, 19.6, 18.9, 12.1, −3.4, −3.6, −7.3 ppm;

HRMS (ESI): $C_{23}H_{51}NO_3SiSnNa$ $[M+Na]^+$ calculated: 560.2558, found: 560.2552;

R_f = 0.5 (PE:MTBE 95:5, vanillin);

$[\alpha]_D^{20}$ = −9.98 (c 1.4, $CHCl_3$).

Stannane S64

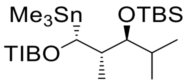

S64
$C_{32}H_{60}O_3SiSn$
639.62

According to GP13, TIB ester **10a** (175 mg, 0.37 mmol, 1.0 equiv) and (+)-sparteine (0.13 mL, 0.55 mmol, 1.5 equiv) gave stannane (+)-sp-**S64** (166 mg, contaminated with 66% TIB ester, 0.09 mmol, 24%, dr 17:1)[12] after purification by flash column chromatography (PE:MTBE 200:1 → 150:1) as a colorless oil.

^1H NMR (400 MHz, C_6D_6): δ = 7.09 (s, 2H), 5.67 (m_c, 1H), 3.57 (dd, J = 7.8, 2.3 Hz, 1H), 3.19 (covered by TIB ester, sep, J = 6.8 Hz, 2H), 2.82-2.71 (covered by TIB ester, m, 1H), 2.52 (m_c, 1H), 1.85 (m_c, 1H), 1.33 (d, J = 6.9 Hz, 12H), 1.18 (partly covered by TIB ester, d, J = 6.8 Hz, 6H), 1.06-1.04 (m, 12H), 0.98 (d, J = 6.9 Hz, 3H), 0.94 (d, J = 6.8 Hz, 3H), 0.38 (m_c, 9H), 0.25 (s, 3H), 0.10 (s, 3H) ppm;

$^{13}C\{^1H\}$ NMR (101 MHz, C_6D_6): δ = 171.0, 150.4, 145.9, 131.6, 121.3, 77.6, 77.1, 44.5, 34.8, 32.0, 30.6, 26.4, 24.7, 24.5, 24.2, 21.7, 18.7, 16.1, 15.7, −3.1, −3.2, −6.9 ppm;

HRMS (ESI): $C_{32}H_{60}O_3SiSnNa$ $[M+Na]^+$ calculated: 663.3231, found: 663.3231;

R_f = 0.3 (PE:MTBE 98:2, uv, vanillin).

Stannane S65

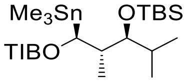

S65
$C_{32}H_{60}O_3SiSn$
639.62

According to GP13, TIB ester **10a** (175 mg, 0.37 mmol, 1.0 equiv) and (−)-sparteine (0.13 mL, 0.55 mmol, 1.5 equiv) gave stannane (−)-sp-**S65** (201 mg, contaminated with 53% TIB ester, 0.15 mmol, 41%, dr 19:1)[13] after purification by flash column chromatography (PE:MTBE 200:1 → 150:1) as a colorless oil.

[12] This mixture was only used to determine the stereochemistry of the formed stannane.
[13] This mixture was only used to determine the stereochemistry of the formed stannane.

According to GP13, TIB ester **10a** (210 mg, 0.44 mmol, 1.0 equiv) and TMEDA (0.10 mL, 0.66 mmol, 1.5 equiv) gave stannane TMEDA-**S65** (258 mg, 0.40 mmol, 91%, dr 3:1) after purification by flash column chromatography (PE:MTBE 200:1 → 150:1) as a colorless oil.

Analytical data are given for stannane (−)-sp-**S65** obtained by the reaction of TIB ester **10a** and (−)-sparteine (dr 19:1).

^{1}H NMR (400 MHz, C_6D_6): δ = 7.11 (s, 2H), 5.21 (m_c, 1H), 3.68 (dd, J = 8.4, 1.8 Hz, 1H), 3.23-3.15 (covered by TIB ester, m, 2H), 2.82-2.69 (covered by TIB ester, m, 1H), 2.20-2.10 (covered by TIB ester, m, 1H), 1.78-1.70 (covered by TIB ester, m, 1H), 1.35-1.31 (partly covered by TIB ester, m, 12H), 1.18 (dd, J = 6.9, 1.0 Hz, 6H), 1.10 (d, J = 7.2 Hz, 3H), 1.03-1.01 (m, 12H), 0.93 (d, J = 7.0 Hz, 3H), 0.39 (m_c, 9H), 0.23 (s, 3H), 0.09 (s, 3H) ppm;

^{13}C{^{1}H} NMR (101 MHz, C_6D_6): δ = 171.9, 150.5, 146.1, 131.1, 121.4, 78.8, 75.3, 42.4, 34.8, 32.0, 31.0, 26.5, 25.0, 24.7, 24.1, 21.5, 18.9, 14.8, 14.4, −3.1, −3.4, −7.3 ppm;

HRMS (ESI): $C_{32}H_{60}O_3SiSnNa$ $[M+Na]^+$ calculated: 663.3231, found: 663.3231;

R*f* = 0.3 (PE:MTBE 98:2, uv, vanillin).

Stannane S66

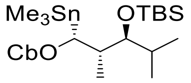

S66
$C_{23}H_{51}NO_3SiSn$
536.46

According to GP13, carbamate **10b** (175 mg, 0.47 mmol, 1.0 equiv) and (+)-sparteine (0.16 mL, 0.70 mmol, 1.5 equiv) gave stannane (+)-sp-**S66** (72 mg, 0.13 mmol, 28%, dr ≥ 19:1) after purification by flash column chromatography (PE:MTBE 100:1) as a colorless oil.
According to GP13, carbamate **10b** (175 mg, 0.47 mmol, 1.0 equiv) and TMEDA (0.11 mL, 0.70 mmol, 1.5 equiv) gave stannane TMEDA-**S66** (101 mg, 0.19 mmol, 40%, dr 5:1) after purification by flash column chromatography (PE:MTBE 100:1) as a colorless oil.

Analytical data are given for stannane (+)-sp-**S66** obtained by the reaction of carbamate **10b** and (+)-sparteine (dr ≥ 19:1).

^{1}H NMR (400 MHz, C_6D_6): δ = 4.22 (m_c, 1H), 3.86-3.60 (m, 3H), 2.77 (m_c, 1H), 1.99 (m_c, 1H), 1.17 (brs, 6H), 1.07 (d, J = 6.9 Hz, 3H), 1.05-1.01 (m, 18H), 0.99 (d, J = 7.2 Hz, 3H), 0.30 (m_c, 9H), 0.18 (s, 3H), 0.14 (s, 3H) ppm;

^{13}C{^{1}H} NMR (101 MHz, C_6D_6): δ = 155.9, 76.7, 75.1, 45.9, 42.8, 29.1, 26.3, 22.9, 21.3 (brs), 20.7 (brs), 18.8, 18.6, 12.6, −3.8, −4.4, −7.5 ppm;

HRMS (ESI): $C_{23}H_{51}NO_3SiSnNa$ $[M+Na]^+$ calculated: 560.2558, found: 560.2564;

R*f* = 0.5 (PE:MTBE 95:5, vanillin);

$[\alpha]_D^{20}$ = +4.83 (*c* 1.0, $CHCl_3$).

Stannane S67

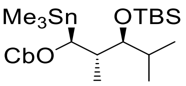

S67
$C_{23}H_{51}NO_3SiSn$
536.46

According to GP13, carbamate **10b** (175 mg, 0.47 mmol, 1.0 equiv) and (−)-sparteine (0.16 mL, 0.70 mmol, 1.5 equiv) gave stannane (−)-sp-**S67** (170 mg, 0.32 mmol, 68%, dr ≥ 19:1) after purification by flash column chromatography (PE:MTBE 100:1) as a colorless oil.

^{1}H NMR (400 MHz, C_6D_6): δ = 4.99 (m_c, 1H), 3.88-3.68 (m, 3H), 2.06 (m_c, 1H), 1.85 (m_c, 1H), 1.17-1.01 (m, 27H), 0.95 (d, J = 6.8 Hz, 3H), 0.32 (m_c, 9H), 0.19 (s, 3H), 0.14 (s, 3H) ppm;

^{13}C{^{1}H} NMR (101 MHz, C_6D_6): δ = 156.4, 78.5, 74.4, 45.9 (brs), 41.7, 30.8, 26.6, 22.0, 21.4 (brs), 20.7 (brs), 18.9, 15.4, 14.7, −2.8, −4.3, −7.9 ppm;

HRMS (ESI): $C_{23}H_{51}NO_3SiSnNa$ $[M+Na]^+$ calculated: 560.2558, found: 560.2562;

R$_f$ = 0.5 (PE:MTBE 95:5, vanillin);

$[\alpha]_D^{20}$ = +50.2 (c 1.3, $CHCl_3$).

(*R*)-Allylic alcohol 18a

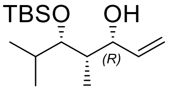

(*R*)-18a
$C_{15}H_{32}O_2Si$
272.50

According to GP14, stannane TMEDA-**23** (156 mg, 0.24 mmol, 1.5 equiv) and vinyl boronic ester **14** (25 mg, 0.16 mmol, 1.0 equiv) gave allylic alcohol **(*R*)-18a** (23 mg, 0.08 mmol, 50% o2s, dr 3:1) after purification by flash column chromatography (PE:MTBE 95:5) as a colorless oil.
Analytical data are in accordance with those given for allylic alcohol **(*R*)-18a** obtained by the reaction of TIB ester **9a** and (+)-sparteine (dr ≥ 19:1).

^{1}H NMR (400 MHz, C_6D_6): δ = 5.74 (ddd, J = 17.4, 10.5, 5.7 Hz, 1H), 5.22 (dt, J = 17.2, 1.6 Hz, 1H), 5.03 (dt, J = 10.6, 1.6 Hz, 1H), 4.01 (brs, 1H), 3.59 (t, J = 4.1 Hz, 1H), 1.85-1.75 (m, 2H), 1.70-1.60 (m, 1H), 1.05 (d, J = 7.0 Hz, 3H), 1.01 (s, 9H), 0.91 (d, J = 6.9 Hz, 3H), 0.87 (d, J = 7.1 Hz, 3H), 0.10 (s, 3H), 0.07 (s, 3H) ppm;

^{13}C{^{1}H} NMR (101 MHz, C_6D_6): δ = 141.2, 114.6, 78.5, 75.0, 41.3, 33.3, 26.4, 19.4, 18.7, 18.2, 10.2, −3.4, −3.7 ppm;

HRMS (ESI): $C_{15}H_{32}O_2SiNa$ $[M+Na]^+$ calculated: 295.2069, found: 295.2077;

R$_f$ = 0.2 (PE:MTBE 9:1, vanillin);

$[\alpha]_D^{20}$ = +17.4 (c 0.8, $CHCl_3$).

(*S*)-Allylic alcohol 18b

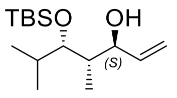

(*S*)-18b
$C_{15}H_{32}O_2Si$
272.50

According to GP15, stannane TMEDA-**24** (131 mg, 0.24 mmol, 1.5 equiv) and vinyl boronic ester **14** (25 mg, 0.16 mmol, 1.0 equiv) gave allylic alcohol **(*S*)-18b** (24 mg, 0.09 mmol, 56% o2s, dr 8:1) after purification by flash column chromatography (PE:MTBE 9:1) as a colorless oil.
Analytical data are in accordance with those given for allylic alcohol **(*S*)-18b** obtained by the reaction of carbamate **9b** and (−)-sparteine (dr ≥ 19:1).

^{1}H NMR (400 MHz, C_6D_6): δ = 5.71 (m$_c$, 1H), 5.15 (m$_c$, 1H), 4.99 (m$_c$, 1H), 3.91-3.85 (m, 2H), 1.78 (m$_c$, 1H), 1.69-1.61 (m, 2H), 1.02 (s, 9H), 0.99 (d, J = 6.7 Hz, 3H), 0.87-0.84 (m, 6H), 0.15 (s, 3H), 0.11 (s, 3H) ppm;

^{13}C{^{1}H} NMR (101 MHz, C_6D_6): δ = 141.4, 115.5, 77.2, 75.5, 42.1, 32.6, 26.5, 20.1, 19.9, 18.7, 11.6, −3.75, −3.79 ppm;

HRMS (ESI): $C_{15}H_{32}O_2SiNa$ [M+Na]$^+$ calculated: 295.2066, found: 295.2069;

R$_f$ = 0.2 (PE:MTBE 95:5, vanillin);

$[\alpha]_D^{20}$ = −10.2 (c 0.9, $CHCl_3$).

(*R*)-Allylic alcohol 17a

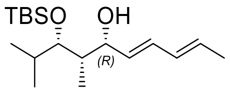

(*R*)-17a
$C_{18}H_{36}O_2Si$
312.57

According to GP14, stannane TMEDA-**23** (156 mg, 0.24 mmol, 1.5 equiv) and vinyl boronic ester **13** (32 mg, 0.16 mmol, 1.0 equiv) gave allylic alcohol **(*R*)-17a** (37 mg, 0.12 mmol, 75% o2s, dr 3:1) after purification by flash column chromatography (PE:MTBE 95:5) as a colorless oil.
Analytical data are in accordance with those given for allylic alcohol allylic alcohol **(*R*)-17a** obtained by the reaction of TIB ester **9a** and TMEDA (dr 13:1).

^{1}H NMR (400 MHz, C_6D_6): δ = 6.30-6.23 (m, 1H), 6.08-6.01 (m, 1H), 5.59-5.50 (m, 2H), 4.08 (brs, 1H), 3.63 (t, J = 4.1 Hz, 1H), 1.86-1.78 (m, 1H), 1.74-1.68 (m, 1H), 1.59 (dd, J = 6.6, 1.3 Hz, 3H), 1.22-1.20 (m, 1H), 1.10 (d, J = 6.9 Hz, 3H), 1.02 (s, 9H), 0.92 (d, J = 6.9 Hz, 3H), 0.88 (d, J = 6.8 Hz, 3H), 0.13 (s, 3H), −0.08 (s, 3H) ppm;

^{13}**C{^{1}H} NMR** (101 MHz, C_6D_6): δ = 133.7, 131.7, 131.3, 129.0, 78.4, 74.9, 41.8, 33.5, 26.4, 19.3, 18.7, 18.3, 18.2, 10.5, −3.4, −3.7 ppm;

HRMS (ESI): $C_{18}H_{36}O_2SiNa$ $[M+Na]^+$ calculated: 335.2382, found: 335.2375;

R*f* = 0.3 (PE:MTBE 95:5, uv, vanillin);

$[\alpha]_D^{20}$ = +21.8 (*c* 1.0, $CHCl_3$).

(*S*)-Allylic alcohol 17b

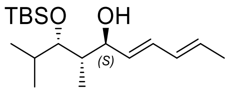

(*S*)-17b
$C_{18}H_{36}O_2Si$
312.57

According to GP15, stannane TMEDA-**24** (131 mg, 0.24 mmol, 1.5 equiv) and vinyl boronic ester **13** (32 mg, 0.16 mmol, 1.0 equiv) gave allylic alcohol **(*S*)-17b** (38 mg, 0.12 mmol, 75% o2s, dr 6:1) after purification by flash column chromatography (PE:MTBE 95:5) as a colorless oil.

^{1}**H NMR** (400 MHz, C_6D_6): δ = 6.23-6.17 (m, 1H), 6.05-5.98 (m, 1H), 5.56-5.43 (m, 2H), 3.97-3.90 (m, 2H), 1.84-1.76 (m, 1H), 1.74-1.66 (m, 1H), 1.60-1.58 (m, 4H), 1.04 (s, 9H), 1.01 (d, *J* = 6.8 Hz, 3H), 0.90-0.87 (m, 6H), 0.19 (s, 3H), 0.13 (s, 3H) ppm;

^{13}**C{^{1}H} NMR** (101 MHz, C_6D_6): δ = 133.9, 132.2, 131.7, 129.3, 77.1, 74.8, 42.4, 32.8, 26.5, 20.04, 20.03, 18.8, 18.2, 11.6, −3.72, −3.73 ppm;

HRMS (ESI): $C_{18}H_{36}O_2SiNa$ $[M+Na]^+$ calculated: 335.2382, found: 335.2375;

R*f* = 0.3 (PE:MTBE 95:5, uv, vanillin);

$[\alpha]_D^{20}$ = −18.9 (*c* 0.9, $CHCl_3$).

(*S*)-Allylic alcohol 16a

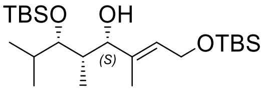

(*S*)-16a
$C_{23}H_{50}O_3Si_2$
430.82

According to GP14, stannane TMEDA-**23** (156 mg, 0.24 mmol, 1.5 equiv) and vinyl boronic ester **12** (50 mg, 0.16 mmol, 1.0 equiv) gave allylic alcohol **(*S*)-16a** (41 mg, 0.10 mmol, 63% o2s, dr ≥ 19:1) after purification by flash column chromatography (PE:MTBE 95:5) as a colorless oil.
Analytical data are in accordance with those given for allylic alcohol **(*S*)-16a** obtained by the reaction of TIB ester **9a** and TMEDA (dr 10:1).

^{1}H NMR (400 MHz, C_6D_6): δ = 5.80 (m$_c$, 1H), 4.26 (m$_c$, 2H), 3.87 (d, J = 4.9 Hz, 1H), 3.59 (t, J = 4.0 Hz, 1H), 1.85-1.77 (m, 2H), 1.46 (s, 3H), 1.18-1.17 (m, 1H), 1.08 (d, J = 6.9 Hz, 3H), 1.02 (s, 9H), 1.01 (s, 9H), 0.95 (d, J = 6.9 Hz, 3H), 0.89 (d, J = 6.9 Hz, 3H), 0.15 (s, 3H), 0.12 (s, 6H), 0.10 (s, 3H) ppm;

^{13}C{^{1}H} NMR (101 MHz, C_6D_6): δ = 138.0, 126.5, 78.6, 78.5, 60.1, 38.3, 33.4, 26.5, 26.2, 19.7, 18.8, 18.5, 18.1, 13.0, 10.1, −3.4, −3.5, −4.9, −5.0 ppm;

HRMS (ESI): $C_{23}H_{50}O_3Si_2Na$ [M+Na]$^+$ calculated: 453.3196, found: 453.3200;

R*f* = 0.3 (PE:MTBE 9:1, vanillin);

$[\alpha]_D^{20}$ = +6.52 (*c* 1.0, $CHCl_3$).

(*R*)-Allylic alcohol 16b

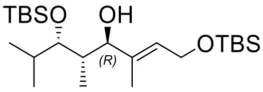

(*R*)-16b
$C_{23}H_{50}O_3Si_2$
430.82

According to GP15, stannane TMEDA-**24** (131 mg, 0.24 mmol, 1.5 equiv) and vinyl boronic ester **12** (50 mg, 0.16 mmol, 1.0 equiv) gave allylic alcohol **(*R*)-16b** (52 mg, 0.12 mmol, 75% o2s, dr 4:1) after purification by flash column chromatography (PE:MTBE 95:5) as a colorless oil.
Analytical data are in accordance with those given for allylic alcohol **(*R*)-16b** obtained by the reaction of carbamate **9b** and TMEDA (dr 4:1).

^{1}H NMR (400 MHz, C_6D_6): δ = 5.68 (m$_c$, 1H), 4.28-4.21 (m, 2H), 3.94-3.87 (m, 2H), 1.89-1.77 (m, 2H), 1.70 (brs, 1H), 1.57 (d, J = 1.2 Hz, 3H), 1.02-1.01 (m, 12H), 1.00 (s, 9H), 0.89 (d, J = 6.8 Hz, 3H), 0.79 (d, J = 7.1 Hz, 3H), 0.15 (s, 3H), 0.12-0.10 (m, 9H) ppm;

^{13}C{^{1}H} NMR (101 MHz, C_6D_6): δ = 138.0, 128.6, 79.8, 77.5, 60.1, 39.2, 32.6, 26.4, 26.2, 20.3, 20.2, 18.7, 18.5, 11.7, 10.8, −3.7, −3.8, −4.941, −4.945 ppm;

HRMS (ESI): $C_{23}H_{50}O_3Si_2Na$ $[M+Na]^+$ calculated: 453.3196, found: 453.3199;

$\mathbf{R}_f$ = 0.3 (PE:MTBE 9:1, vanillin);

$[\alpha]_D^{20}$ = −8.63 (*c* 1.0, $CHCl_3$).

(*S*)-Allylic alcohol 15a

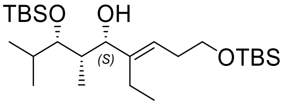

(*S*)-15a
$C_{25}H_{54}O_3Si_2$
458.87

According to GP14, stannane TMEDA-**23** (156 mg, 0.24 mmol, 1.5 equiv) and vinyl boronic ester **11** (55 mg, 0.16 mmol, 1.0 equiv) gave allylic alcohol **(*S*)-15a** (53 mg, 0.12 mmol, 75% o2s, dr ≥ 19:1) after purification by flash column chromatography (PE:MTBE 95:5) as a colorless oil.
Analytical data are in accordance with those given for allylic alcohol **(*S*)-15a** obtained by the reaction of TIB ester **9a** and TMEDA (dr 25:1).

^{1}H NMR (400 MHz, C_6D_6): δ = 5.58 (m_c, 1H), 4.09 (brs, 1H), 3.68-3.62 (m, 3H), 2.40-2.30 (m, 2H), 2.15-2.02 (m, 1H), 1.94-1.81 (m, 3H), 1.26 (d, *J* = 3.2 Hz, 1H), 1.07 (d, *J* = 7.0 Hz, 3H), 1.03 (s, 9H), 1.00-0.96 (m, 15H), 0.93 (d, *J* = 6.8 Hz, 3H), 0.16 (s, 3H), 0.10 (s, 3H), 0.09 (s, 6H) ppm;

^{13}C{^{1}H} NMR (101 MHz, C_6D_6): δ = 144.5, 121.7, 79.6, 76.9, 63.5, 38.8, 33.1, 31.8, 26.5, 26.2, 21.8, 19.9, 18.8, 18.6, 17.8, 14.4, 9.8, −3.3, −3.6, −5.097, −5.105 ppm;

HRMS (ESI): $C_{25}H_{54}O_3Si_2Na$ $[M+Na]^+$ calculated: 481.3509, found: 481.3514;

$\mathbf{R}_f$ = 0.3 (PE:MTBE 95:5, vanillin);

$[\alpha]_D^{20}$ = +10.3 (*c* 0.9, $CHCl_3$).

(*R*)-Allylic alcohol 15b

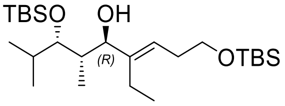

(*R*)-15b
$C_{25}H_{54}O_3Si_2$
458.87

According to GP15, stannane TMEDA-**24** (131 mg, 0.24 mmol, 1.5 equiv) and vinyl boronic ester **11** (55 mg, 0.16 mmol, 1.0 equiv) gave allylic alcohol **(*R*)-15b** (51 mg, contaminated with 5% ketone, 0.11 mmol, 69% o2s, dr 4:1) after purification by flash column chromatography (PE:MTBE 95:5) as a colorless oil.

Analytical data are in accordance with those given for allylic alcohol **(*R*)-15b** obtained by the reaction of carbamate **9b** and TMEDA (dr 4:1).

^{1}H NMR (400 MHz, C_6D_6): δ = 5.45 (m$_c$, 1H), 3.99-3.95 (m, 2H), 3.59 (t, *J* = 6.7 Hz, 2H), 2.31 (q, *J* = 6.8 Hz, 2H), 2.16-2.05 (m, 2H), 1.94-1.82 (m, 2H), 1.66 (brs, 1H), 1.10 (t, *J* = 7.6 Hz, 3H), 1.05-1.03 (m, 12H), 1.00 (s, 9H), 0.92 (d, *J* = 6.9 Hz, 3H), 0.85 (d, *J* = 7.0 Hz, 3H), 0.21 (s, 3H), 0.14 (s, 3H), 0.08 (s, 6H) ppm;

^{13}C{^{1}H} NMR (101 MHz, C_6D_6): δ = 144.8, 125.3, 80.1, 77.5, 63.2, 39.9, 32.7, 31.7, 26.5, 26.2, 20.34, 20.32, 20.29, 18.8, 18.5, 15.3, 12.0, −3.71, −3.74, −5.13, −5.14 ppm;

HRMS (ESI): $C_{25}H_{54}O_3Si_2Na$ [M+Na]$^+$ calculated: 481.3509, found: 481.3506;

R*f* = 0.3 (PE:MTBE 95:5, vanillin);

$[\alpha]_D^{20}$ = −3.80 (*c* 1.0, $CHCl_3$).

(*S*)-Allylic alcohol 22a

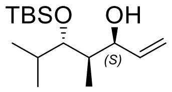

(*S*)-22a
$C_{15}H_{32}O_2Si$
272.50

According to GP14, stannane TMEDA-**S65** (156 mg, 0.24 mmol, 1.5 equiv) and vinyl boronic ester **14** (25 mg, 0.16 mmol, 1.0 equiv) gave allylic alcohol **(*S*)-22a** (29 mg, 0.11 mmol, 69% o2s, dr 3:1) after purification by flash column chromatography (PE:MTBE 95:5) as a colorless oil.
Analytical data are in accordance with those given for allylic alcohol **(*S*)-22a** obtained by the reaction of carbamate **10b** and (−)-sparteine (dr 19:1).

^{1}H NMR (400 MHz, C_6D_6): δ = 5.77 (m$_c$, 1H), 5.44 (dt, *J* = 17.3, 1.9 Hz, 1H), 5.12 (dt, *J* = 10.6, 1.9 Hz, 1H), 4.68-4.64 (m, 1H), 3.40 (t, *J* = 4.9 Hz, 1H), 1.84-1.73 (m, 1H), 1.65-1.57 (m, 1H), 0.97 (s, 9H), 0.94 (d, *J* = 7.1 Hz, 3H), 0.87 (d, *J* = 7.1 Hz, 3H), 0.82 (d, *J* = 6.9 Hz, 3H), 0.09 (s, 3H), 0.04 (s, 3H) ppm;

^{13}C{^{1}H} NMR (101 MHz, C_6D_6): δ = 141.2, 113.7, 81.9, 71.4, 40.4, 32.1, 26.4, 20.2, 18.7, 18.5, 11.5, −3.56, −3.64 ppm;

HRMS (ESI): $C_{15}H_{32}O_2SiNa$ [M+Na]$^+$ calculated: 295.2069, found: 295.2077;

R*f* = 0.2 (PE:MTBE 95:5, vanillin);

$[\alpha]_D^{20}$ = −3.0 (*c* 0.8, $CHCl_3$).

(*R*)-Allylic alcohol 22b

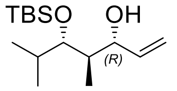

(*R*)-22b
$C_{15}H_{32}O_2Si$
272.50

According to GP15, stannane TMEDA-**S66** (131 mg, 0.24 mmol, 1.5 equiv) and vinyl boronic ester **14** (25 mg, 0.16 mmol, 1.0 equiv) gave allylic alcohol **(*R*)-22b** (24 mg, 0.09 mmol, 56% o2s, dr 6:1) after purification by flash column chromatography (PE:MTBE 9:1) as a colorless oil.
Analytical data are in accordance with those given for the pure allylic alcohol **(*R*)-22b** obtained by the reaction of carbamate **10b** and TMEDA.

^{1}H NMR (400 MHz, C_6D_6): δ = 5.84 (m_c, 1H), 5.27 (ddd, *J* = 17.1, 10.5, 4.1 Hz, 1H), 5.06 (m_c, 1H), 4.04 (t, *J* = 7.2 Hz, 1H), 3.57 (dd, *J* = 5.3, 4.2 Hz, 1H), 2.22 (brs, 1H), 1.88-1.78 (m, 2H), 0.98 (s, 9H), 0.95 (d, *J* = 6.7 Hz, 3H), 0.93 (d, *J* = 6.5 Hz, 3H), 0.83 (d, *J* = 5.9 Hz, 3H), 0.09 (s, 3H), 0.05 (s, 3H) ppm;

^{13}C{^{1}H} NMR (101 MHz, C_6D_6): δ = 140.5, 115.1, 80.4, 75.3, 43.5, 32.4, 26.3, 20.5, 18.5, 18.2, 14.4, −3.8, −4.1 ppm;

HRMS (ESI): $C_{15}H_{32}O_2SiNa$ $[M+Na]^+$ calculated: 295.2069, found: 295.2066;

R*f* = 0.2 (PE:MTBE 95:5, vanillin);

$[\alpha]_D^{20}$ = +8.3 (*c* 0.8, $CHCl_3$).

(*S*)-Allylic alcohol 21a

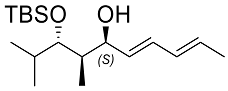

(*S*)-21a
$C_{18}H_{36}O_2Si$
312.57

According to GP14, stannane TMEDA-**S65** (156 mg, 0.24 mmol, 1.5 equiv) and vinyl boronic ester **13** (32 mg, 0.16 mmol, 1.0 equiv) gave allylic alcohol **(*S*)-21a** (38 mg, 0.12 mmol, 75% o2s, dr 4:1) after purification by flash column chromatography (PE:MTBE 98:2 → 10:1) as a colorless oil.

Analytical data are in accordance with those given for allylic alcohol **(*S*)-21a** obtained by the reaction of TIB ester **10a** and TMEDA (dr 5:1).

^{1}H NMR (400 MHz, C_6D_6): δ = 6.48 (m_c, 1H), 6.14-6.07 (m, 1H), 5.59-5.54 (m, 2H), 4.73 (brs, 1H), 3.43 (t, *J* = 4.9 Hz, 1H), 2.56 (d, *J* = 2.3 Hz, 1H), 1.86-1.77 (m, 1H), 1.69-1.58 (m, 4H), 0.99-0.97 (m, 12H), 0.89 (d, *J* = 6.8 Hz, 3H), 0.85 (d, *J* = 6.9 Hz, 3H), 0.11 (s, 3H), 0.05 (s, 3H) ppm;

$^{13}C\{^1H\}$ NMR (101 MHz, C_6D_6): δ = 134.0, 132.0, 130.1, 128.3, 81.9, 71.1, 41.1, 32.1, 26.4, 20.2, 18.7, 18.5, 18.2, 11.8, −3.55, −3.62 ppm;

HRMS (ESI): $C_{18}H_{36}O_2SiNa$ $[M+Na]^+$ calculated: 335.2382, found: 335.2387;

R$_f$ = 0.3 (PE:MTBE 95:5, uv, vanillin);

$[\alpha]_D^{20}$ = −0.92 (*c* 0.8, $CHCl_3$).

(*R*)-Allylic alcohol 21b

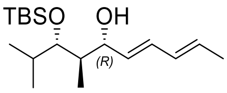

(*R*)-21b
$C_{18}H_{36}O_2Si$
312.57

According to GP15, stannane TMEDA-**S66** (131 mg, 0.24 mmol, 1.5 equiv) and vinyl boronic ester **13** (32 mg, 0.16 mmol, 1.0 equiv) gave allylic alcohol **(*R*)-21b** (29 mg, 0.09 mmol, 56% o2s, dr 4:1) after purification by flash column chromatography (PE:MTBE 95:5) as a colorless oil.
Analytical data are in accordance with those given for allylic alcohol **(*R*)-21b** obtained by the reaction of carbamate **10b** and TMEDA (dr 2:1).

^1H NMR (400 MHz, C_6D_6): δ = 6.30 (dd, *J* = 15.1, 10.4 Hz, 1H), 6.10-6.03 (m, 1H), 5.63-5.52 (m, 2H), 4.09 (t, *J* = 7.6 Hz, 1H), 3.64 (dd, *J* = 5.0, 4.3 Hz, 1H), 2.22 (d, *J* = 2.4 Hz, 1H), 1.92-1.82 (m, 2H) 1.59 (dd, *J* = 6.6, 1.4 Hz, 3H), 1.00-0.98 (m, 12H), 0.95 (d, *J* = 6.8 Hz, 3H), 0.86 (d, *J* = 7.1 Hz, 3H), 0.13 (s, 3H), 0.07 (s, 3H) ppm;

$^{13}C\{^1H\}$ NMR (101 MHz, C_6D_6): δ = 133.1, 131.9, 131.6, 129.0, 80.2, 75.0, 44.1, 32.3, 26.3, 20.6, 18.6, 18.3, 18.2, 14.4, −3.8, −4.1 ppm;

HRMS (ESI): $C_{18}H_{36}O_2SiNa$ $[M+Na]^+$ calculated: 335.2382, found: 335.2387;

R$_f$ = 0.3 (PE:MTBE 95:5, uv, vanillin);

$[\alpha]_D^{20}$ = +13.7 (*c* 0.9, $CHCl_3$).

(*R*)-Allylic alcohol 20a

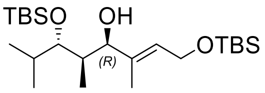

(*R*)-20a
$C_{23}H_{50}O_3Si_2$
430.82

According to GP14, stannane TMEDA-**S65** (156 mg, 0.24 mmol, 1.5 equiv) and vinyl boronic ester **12** (50 mg, 0.16 mmol, 1.0 equiv) gave allylic alcohol **(*R*)-20a** (54 mg, 0.13 mmol, 81% o2s, dr ≥ 19:1) after purification by flash column chromatography (PE:MTBE 95:5) as a colorless oil.

Analytical data are in accordance with those given for allylic alcohol **(*R*)-20a** obtained by the reaction of TIB ester **10a** and TMEDA (dr ≥ 19:1).

^{1}H NMR (400 MHz, C_6D_6): δ = 6.08 (m_c, 1H), 4.43 (brs, 1H), 4.34 (m_c, 2H), 3.43 (m_c, 1H), 2.75 (d, J = 2.0 Hz, 1H), 1.87-1.75 (m, 2H), 1.51 (s, 3H), 1.01 (s, 9H), 0.98 (s, 9H), 0.94 (d, J = 7.1 Hz, 3H), 0.91 (d, J = 6.8 Hz, 3H), 0.86 (d, J = 6.9 Hz, 3H), 0.13 (s, 6H), 0.07 (s, 3H), 0.04 (s, 3H) ppm;

^{13}C{^{1}H} NMR (101 MHz, C_6D_6): δ = 137.3, 124.8, 82.1, 74.1, 60.4, 37.9, 32.1, 26.4, 26.2, 20.3, 18.69, 18.65, 18.6, 14.1, 11.4, −3.5, −3.6, −4.85, −4.88 ppm;

HRMS (ESI): $C_{23}H_{50}O_3Si_2Na$ $[M+Na]^+$ calculated: 453.3196, found: 453.3198;

R*f* = 0.3 (PE:MTBE 9:1, vanillin);

$[\alpha]_D^{20}$ = +10.1 (*c* 1.3, $CHCl_3$).

(*S*)-Allylic alcohol 20b

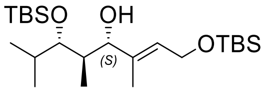

(*S*)-20b
$C_{23}H_{50}O_3Si_2$
430.82

According to GP15, stannane TMEDA-**S66** (131 mg, 0.24 mmol, 1.5 equiv) and vinyl boronic ester **12** (50 mg, 0.16 mmol, 1.0 equiv) gave allylic alcohol **(*S*)-20b** (57 mg, 0.13 mmol, 81% o2s, dr 5:1) after purification by flash column chromatography (PE:MTBE 95:5) as a colorless oil.
Analytical data are in accordance with those given for allylic alcohol **(*S*)-20b** obtained by the reaction of carbamate **10b** and TMEDA (dr 2:1).

^{1}H NMR (400 MHz, C_6D_6): δ = 5.67 (m_c, 1H), 4.25 (m_c, 2H), 3.90 (dd, J = 9.8, 1.4 Hz, 1H), 3.69 (t, J = 4.4 Hz, 1H), 2.37 (d, J = 1.7 Hz, 1H), 2.01-1.92 (m, 1H), 1.90-1.83 (m 1H), 1.65 (m_c, 3H), 1.00 (s, 9H), 0.99-0.96 (m, 15H), 0.80 (d, J = 7.2 Hz, 3H), 0.13 (s, 3H), 0.105 (s, 3H), 0.103 (s, 3H), 0.06 (s, 3H) ppm;

^{13}C{^{1}H} NMR (101 MHz, C_6D_6): δ = 137.6, 128.5, 81.0, 80.3, 60.1, 40.7, 32.4, 26.24, 26.18, 20.7, 18.6, 18.53, 18.46, 15.0, 11.0, −3.9, −4.2, −4.9, −5.0 ppm;

HRMS (ESI): $C_{23}H_{50}O_3Si_2Na$ $[M+Na]^+$ calculated: 453.3196, found: 453.3198;

R*f* = 0.3 (PE:MTBE 9:1, vanillin);

$[\alpha]_D^{20}$ = +2.30 (*c* 1.0, $CHCl_3$).

(*R*)-Allylic alcohol 19a

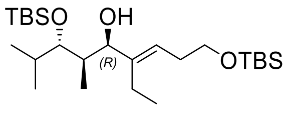

(*R*)-19a
$C_{25}H_{54}O_3Si_2$
458.87

According to GP14, stannane TMEDA-**S65** (156 mg, 0.24 mmol, 1.5 equiv) and vinyl boronic ester **11** (55 mg, 0.16 mmol, 1.0 equiv) gave allylic alcohol **(*R*)-19a** (55 mg, 012 mmol, 75% o2s, dr ≥ 19:1) after purification by flash column chromatography (PE:MTBE 98:2 → 9:1) as a colorless oil.
Analytical data are in accordance with those given for allylic alcohol **(*R*)-19a** obtained by the reaction of TIB ester **10a** and TMEDA (dr ≥ 19:1).

^{1}H NMR (400 MHz, C_6D_6): δ = 5.76 (m_c, 1H), 4.62 (brs, 1H), 3.66 (t, *J* = 7.0 Hz, 2H), 3.51 (t, *J* = 5.1 Hz, 1H), 2.54 (d, *J* = 2.0 Hz, 1H), 2.48-2.35 (m, 2H), 2.16 (m_c, 1H), 1.91-1.77 (m, 3H), 1.06-0.99 (m, 21H), 0.96 (d, *J* = 7.1 Hz, 3H), 0.93 (d, *J* = 6.8 Hz, 3H), 0.90 (d, *J* = 7.0 Hz, 3H), 0.12 (s, 3H), 0.09 (s, 6H), 0.07 (s, 3H) ppm;

^{13}C{^{1}H} NMR (101 MHz, C_6D_6): δ = 143.8, 120.6, 81.9, 72.2, 63.6, 38.1, 32.1, 31.8, 26.5, 26.2, 22.1, 20.4, 18.7, 18.6, 18.3, 14.2, 11.3, −3.46, −3.55, −5.1 ppm;

HRMS (ESI): $C_{25}H_{54}O_3Si_2Na$ $[M+Na]^+$ calculated: 481.3509, found: 481.3511;

R*f* = 0.3 (PE:MTBE 95:5, vanillin);

$[\alpha]_D^{20}$ = +3.19 (*c* 1.2, $CHCl_3$).

(*S*)-Allylic alcohol 19b

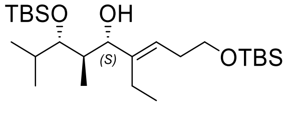

(*S*)-19b
$C_{25}H_{54}O_3Si_2$
458.87

According to GP15, stannane TMEDA-**S66** (131 mg, 0.24 mmol, 1.5 equiv) and vinyl boronic ester **11** (50 mg, 0.16 mmol, 1.0 equiv) gave allylic alcohol **(*S*)-19b** (63 mg, 0.14 mmol, 88% o2s, dr 4:1) after purification by flash column chromatography (PE:MTBE 95:5) as a colorless oil.
Analytical data are in accordance with those given for allylic alcohol **(*S*)-19b** obtained by the reaction of carbamate **10b** and TMEDA (dr 2:1).

^{1}H NMR (400 MHz, C_6D_6): δ = 5.43 (t, *J* = 7.2 Hz, 1H), 3.98 (dd, *J* = 9.8, 1.5 Hz, 1H), 3.71 (t, *J* = 4.4 Hz, 1H), 3.61 (t, *J* = 6.7 Hz, 2H), 2.49 (d, *J* = 1.9 Hz, 1H), 2.34 (q, *J* = 6.7 Hz, 2H), 2.26-2.13 (m, 2H), 2.07-1.99 (m, 1H), 1.94-1.86 (m, 1H), 1.19 (t, *J* = 7.6 Hz, 3H), 1.01-0.98 (m, 24H), 0.85 (d, *J* = 7.1 Hz, 3H), 0.16 (s, 3H), 0.09 (s, 6H), 0.07 (s, 3H) ppm;

^{13}C{^{1}H} NMR (101 MHz, C_6D_6): δ = 144.3, 125.1, 81.7, 80.8, 63.3, 41.3, 32.8, 31.7, 26.3, 26.2, 20.6, 20.4, 18.7, 18.6, 18.5, 15.8, 15.3, −3.9, −4.2, −5.1 ppm;

HRMS (ESI): $C_{25}H_{54}O_3Si_2Na$ $[M+Na]^+$ calculated: 481.3509, found: 481.3511;

R*f* = 0.3 (PE:MTBE 95:5, vanillin);

$[\alpha]_D^{20}$ = +0.81 (*c* 1.0, $CHCl_3$).

Deuterated TIB ester 33a-d1

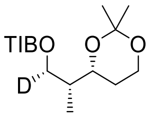

33a-d$_1$
$C_{25}H_{39}DO_4$
405.60

According to GP16, TIB ester **33a** (52 mg, 128 µmol, 1.0 equiv) afforded crude deuterated TIB ester **33a-d1** in a dr of 6:1.[14]

^{1}H NMR (400 MHz, C_6D_6): δ = 7.09 (s, 2H), 4.24 (d, *J* = 6.3 Hz, 1H), 3.72 (m$_c$, 1H), 3.63-3.57 (m, 2H), 3.13 (sep, *J* = 6.8 Hz, 2H), 2.76 (sep, *J* = 6.9 Hz, 1H), 1.83 (m$_c$, 1H), 1.59-1.50 (m, 1H), 1.46 (s, 3H), 1.28 (dd, *J* = 6.8, 1.1 Hz, 12H), 1.24 (s, 3H), 1.19 (d, *J* = 6.9 Hz, 6H), 1.03 (d, *J* = 6.9 Hz, 3H), 0.87 (m$_c$, 1H) ppm.

TIB ester 34a-d1

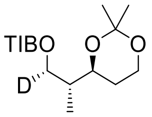

34a-d$_1$
$C_{25}H_{39}DO_4$
405.60

According to GP16, TIB ester **34a** (50 mg, 124 µmol, 1.0 equiv) afforded crude deuterated TIB ester **34a-d1** in a dr of ≥ 19:1.[11]

^{1}H NMR (400 MHz, C_6D_6): δ = 7.10 (s, 2H), 4.38 (d, *J* = 6.3 Hz, 1H), 3.65-3.53 (m, 3H), 3.13 (sep, *J* = 6.8 Hz, 2H), 2.77 (sep, *J* = 6.9 Hz, 1H), 1.87 (m$_c$, 1H), 1.47 (s, 3H), 1.43-1.34 (m, 1H), 1.29 (d, *J* = 6.8 Hz, 12H), 1.25 (s, 3H), 1.20 (d, *J* = 6.9 Hz, 6H), 0.94 (m$_c$, 1H), 0.84 (d, *J* = 7.0 Hz, 3H) ppm.

[14] Absolute stereochemistry was assigned according to determined stereochemistry of lithiation–borylation product.

Carbamate 33b-d1

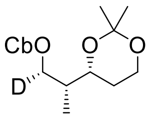

33b-d$_1$
$C_{16}H_{30}DNO_4$
302.43

According to GP17, carbamate **33b** (50 mg, 166 µmol, 1.0 equiv) afforded crude deuterated carbamate **33b-d1** in a dr of 4:1.[11]

^{1}H NMR (400 MHz, C_6D_6): δ = 4.09 (d, J = 6.8 Hz, 1H), 4.04-3.60 (m, 5H), 1.80 (m$_c$, 1H), 1.58-1.47 (m, 1H), 1.46 (s, 3H), 1.29 (s, 3H), 1.11 (d, J = 6.6 Hz, 12H), 1.03 (d, J = 6.9 Hz, 3H), 0.88 (m$_c$, 1H) ppm.

Carbamate 34b-d1

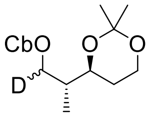

34b-d$_1$
$C_{16}H_{30}DNO_4$
302.43

According to GP17, carbamate **34b** (57 mg, 189 µmol, 1.0 equiv) afforded crude deuterated carbamate **34b-d1** in a dr of 1.1:1.

^{1}H NMR (400 MHz, C_6D_6): δ = 4.37 (d, J = 4.6 Hz, 0.53H), 4.20 (d, J = 6.7 Hz, 0.47H), 4.12-3.54 (m, 5H), 1.89-1.79 (m, 1H), 1.45 (s, 3H), 1.43-1.36 (m, 1H), 1.28 (s, 3H), 1.11 (d, J = 6.7 Hz, 12H), 0.96 (m$_c$, 1H), 0.86 (d, J = 7.0 Hz, 3H) ppm.

3. References

[1] a) Synthesis of vinyl boronic ester **11**: Linne, Y.; Birkner, M.; Flormann, J.; Lücke, D.; Becker, J. A.; Kalesse, M. Sparteine-Free, Highly Stereoselective Construction of Complex Allylic Alcohols Using 1,2-Metallate Rearrangements. *JACS Au* **2023**, *3*, 1695–1710.; b) Synthesis of vinyl boronic ester **12**: Hesse, M. J.; Butts, C. P.; Willis, C. L.; Aggarwal, V. K. Diastereodivergent Synthesis of Trisubstituted Alkenes through Protodeboronation of Allylic Boronic Esters: Application to the Synthesis of the Californian Red Scale Beetle Pheromone. *Angew. Chem. Int. Ed.* **2012**, *51*, 12444–12448; c) Synthesis of vinyl boronic ester **13**: Coombs, J. R.; Zhang, L.; Morken, J. P. Synthesis of Vinyl Boronates from Aldehydes by a Practical Boron–Wittig Reaction. *Org. Lett.* **2015**, *17*, 1708–1711.

[2] a) Evans, D. A.; Connell, B. T. Synthesis of the Antifungal Macrolide Antibiotic (+)-Roxaticin. *J. Am. Chem. Soc.* **2003**, *125*, 10899–10905; b) Zampella, A.; Sepe, V.; D'Orsi, R.; Bifulco, G.; Bassarello, C.; D'Auria, M. V. Stereochemical assignment of the C23–C35 portion of sphinxolide/reidispongiolide class of natural products by asymmetric synthesis. *Tetrahedron: Asymmetry* **2003**, *14*, 1787–1798.

[3] Linne, Y.; Bonandi, E.; Tabet, C.; Geldsetzer, J.; Kalesse, M. The Total Synthesis of Chondrochloren A. *Angew. Chem. Int. Ed.* **2021**, *60*, 6938–6942.

[4] Dias, L. C.; de Lucca Jr, E. C. Total Synthesis of the Oxopolyene Macrolide (−)-Marinisporolide C. *Org. Lett.* **2015**, *17*, 6278–6281.

[5] Fleury, E.; Sorin, G.; Prost, E.; Pancrazi, A.; Sautel, F.; Massiot, G.; Lannou, M-E.; Ardisson, J. Relative Stereochemical Determination and Synthesis of the C17–C20 *δ*-Lactone Fragment of Hemicalide. *J. Org. Chem.* **2013**, *78*, 855–864.

[6] Crimmins, M. T.; King, B. W.; Tabet, E. A.; Chaudhary, K. Asymmetric Aldol Additions: Use of Titanium Tetrachloride and (−)-Sparteine for the Soft Enolization of *N*-Acyl Oxazolidinones, Oxazolidinethiones, and Thiazolidinethiones. *J. Org. Chem.* **2001**, *66*, 894–902.

[7] Sinz, C. J.; Rychnovsky, S. D. Total synthesis of the polyene macrolide dermostatin A. *Tetrahedron* **2002**, *58*, 6561–6576.

[8] Echeverria, P.-G.; Prévost, S.; Cornil, J.; Férard, C.; Reymond, S.; Guérinot, A.; Cossy, J.; Ratovelomanana-Vidal, V.; Phansavath, P. Synthetic Strategy toward the C44–C65 Fragment of Mirabalin. *Org. Lett.* **2014**, *16*, 2390–2393.

[9] Currie, R. H.; Goodman, J. M. In Silico Total Synthesis of (–)-Dolabriferol. *Angew. Chem. Int. Ed.* **2012**, *51*, 4695–4697.

[10] Tsutsumi, R.; Kuranaga, T.; Wright, J. L. C.; Baden, D. G.; Ito, E.; Satake, M.; Tachibana, K. An improved synthesis of (−)-brevisamide, a marine monocyclic ether amide of dinoflagellate origin. *Tetrahedron* **2010**, *66*, 6775–6782.

[11] AnkiReddy, S.; AnkiReddy, P.; Sabitha, G. Studies toward the Total Synthesis of Tianchimycins A and B: Construction of the Complete C1–C16 Framework. *Synthesis* **2015**, *47*, 2860–2868.

[12] Mans, D. M.; Pearson, W. H. Total Synthesis of (+)-Cocaine via Desymmetrization of a *meso*-Dialdehyde. *Org. Lett.* **2004**, *6*, 3305–3308.

[13] Anderson, O. P.; Barrett, A. G M.; Edmunds, J. J.; Hachiya, S.-I.; Hendrix, J. A.; Horita, K.; Malecha, J. W.; Parkinson, C. J.; VanSickle, A. Applications of crotyldiisopinocampheylboranes in synthesis: a formal total synthesis of (+)-calyculin A. *Can. J. Chem.* **2001**, *79*, 1562–1592.

[14] Johns, B. A.; Grant, C. M.; Marshall, J. A.; Holson, E. B.; Roush, W. R. SYNTHESIS AND UTILIZATION OF INDIUM (I) IODIDE FOR IN SITU FORMATION OF ENANTIOENRICHED ALLENYLINDIUM REAGENTS AND THEIR ADDITION TO ALDEHYDES: (2R,3S,4S)-1-(tert-BUTYLDIPHENYLSILYLOXY)-2,4-DIMETHYL-5-HEXYN-3-OL. *Org. Synth.* **2002**, *79*, 59.

[15] Lücke, D.; Kalesse, M. Polyoxygenated Tertiary Alcohols: A Kiyooka Approach. *Chem. Eur. J.* **2019**, *25*, 10080–10083.

[16] Hoppe, D.; Hintze, F.; Tebben, P.; Paetow, M.; Ahrens, H.; Schwerdtfeger, J.; Sommerfeld, P.; Haller, J.; Guarnieri, W.; Kolczewski, S.; Hense, T.; Hoppe, I. Enantioselective synthesis via sparteine-induced asymmetric deprotonation. *Pure & Appl. Chem.* **1994**, *66*, 1479–1486.

[17] Würthwein, E.-U.; Hoppe, D. Enantioselective Lithiation of O-Alkyl and O-Alk-2-enyl Carbamates in the Presence of (−)-Sparteine and (−)-α-Isosparteine. A Theoretical Study. *J. Org. Chem.* ***2005**, 70,* 4443–4451.

4 Spectra

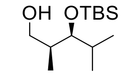

S1

^{1}H NMR (400 MHz, $CDCl_3$)

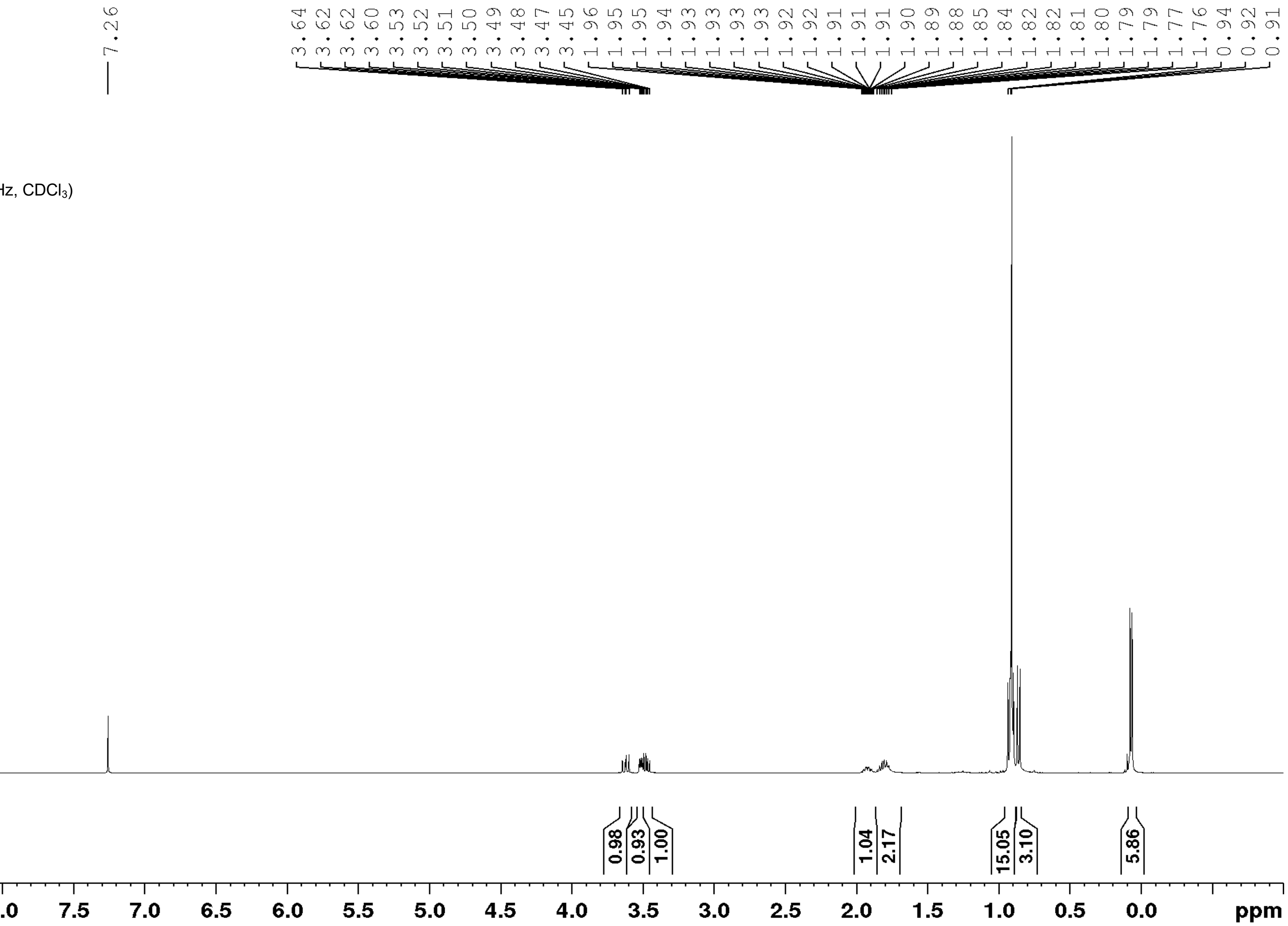

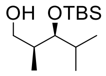

S1

$^{13}C\{^1H\}$ NMR (101 MHz, $CDCl_3$)

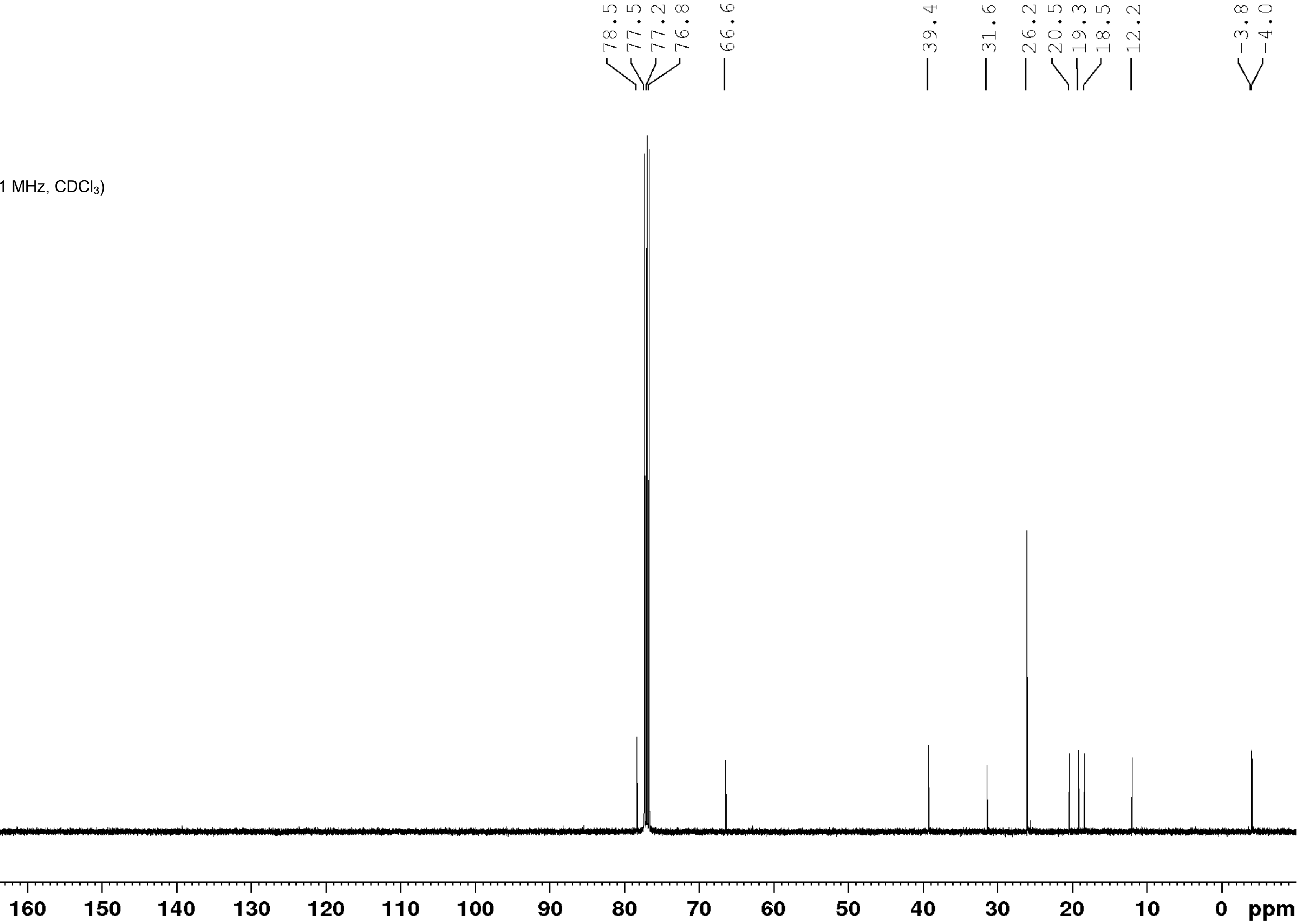

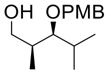

S2

^{1}H NMR (400 MHz, C_6D_6)

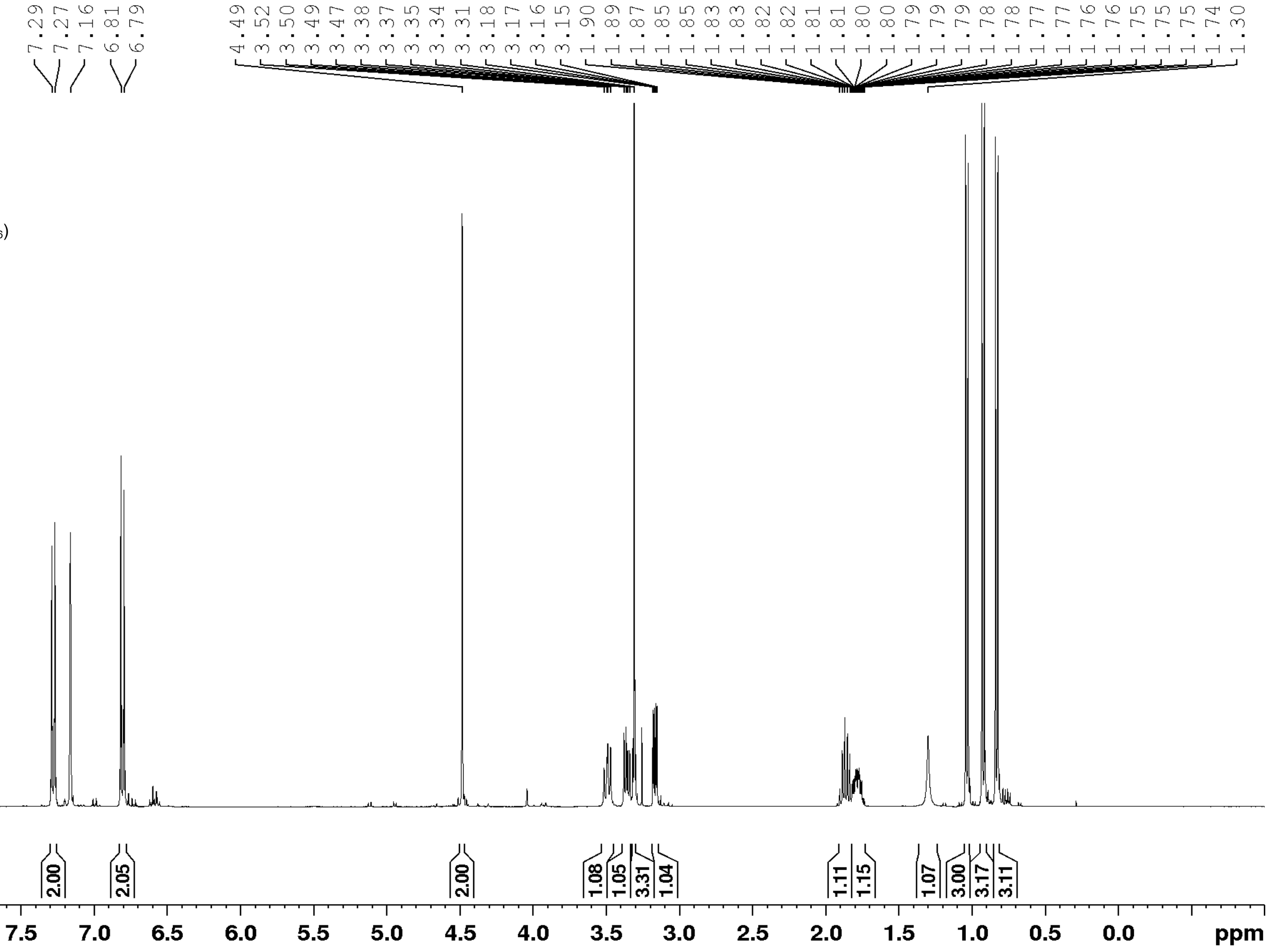

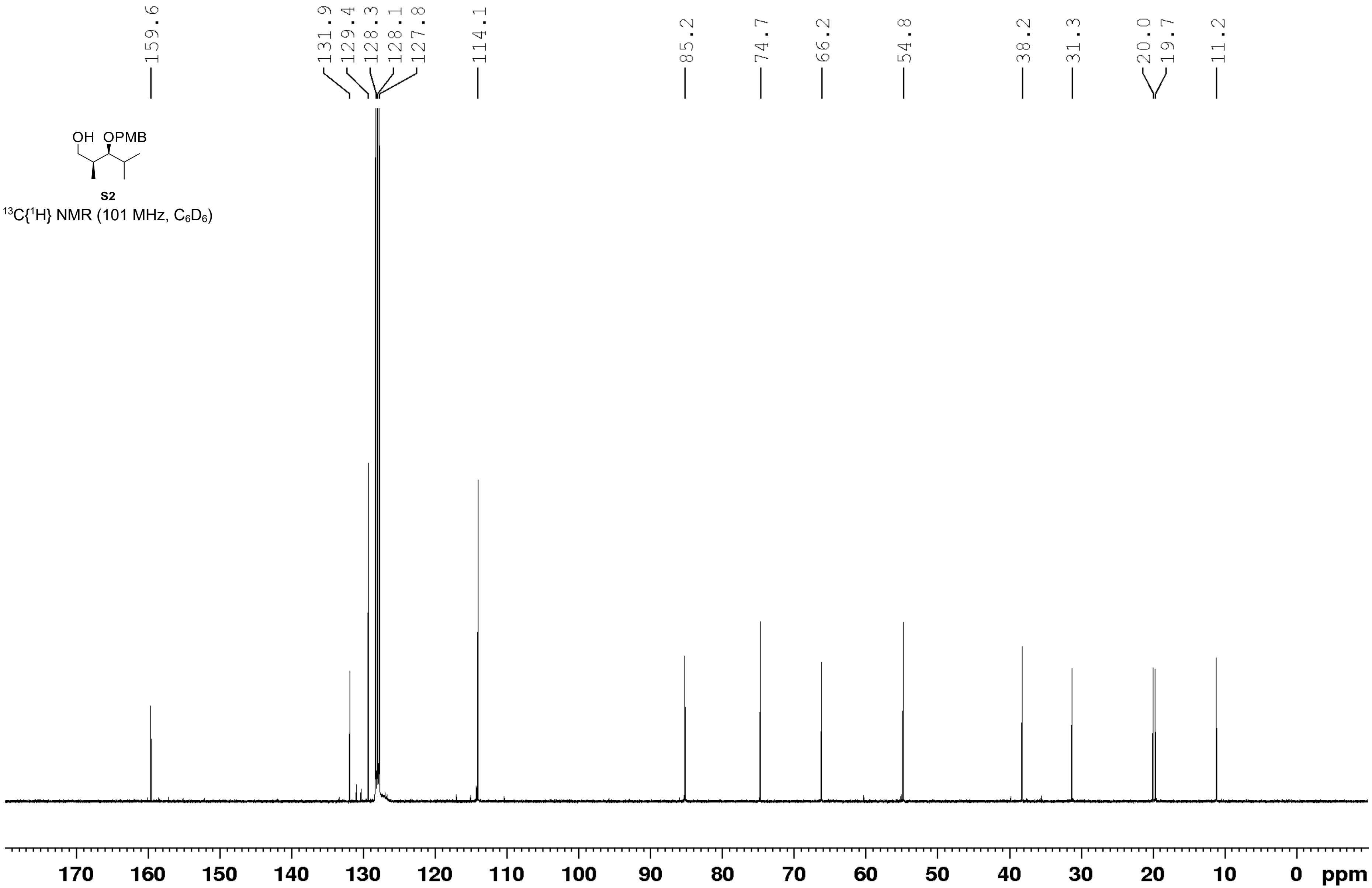

S2

$^{13}C\{^{1}H\}$ NMR (101 MHz, C_6D_6)

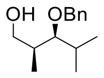

S3

^{1}H NMR (400 MHz, $CDCl_3$)

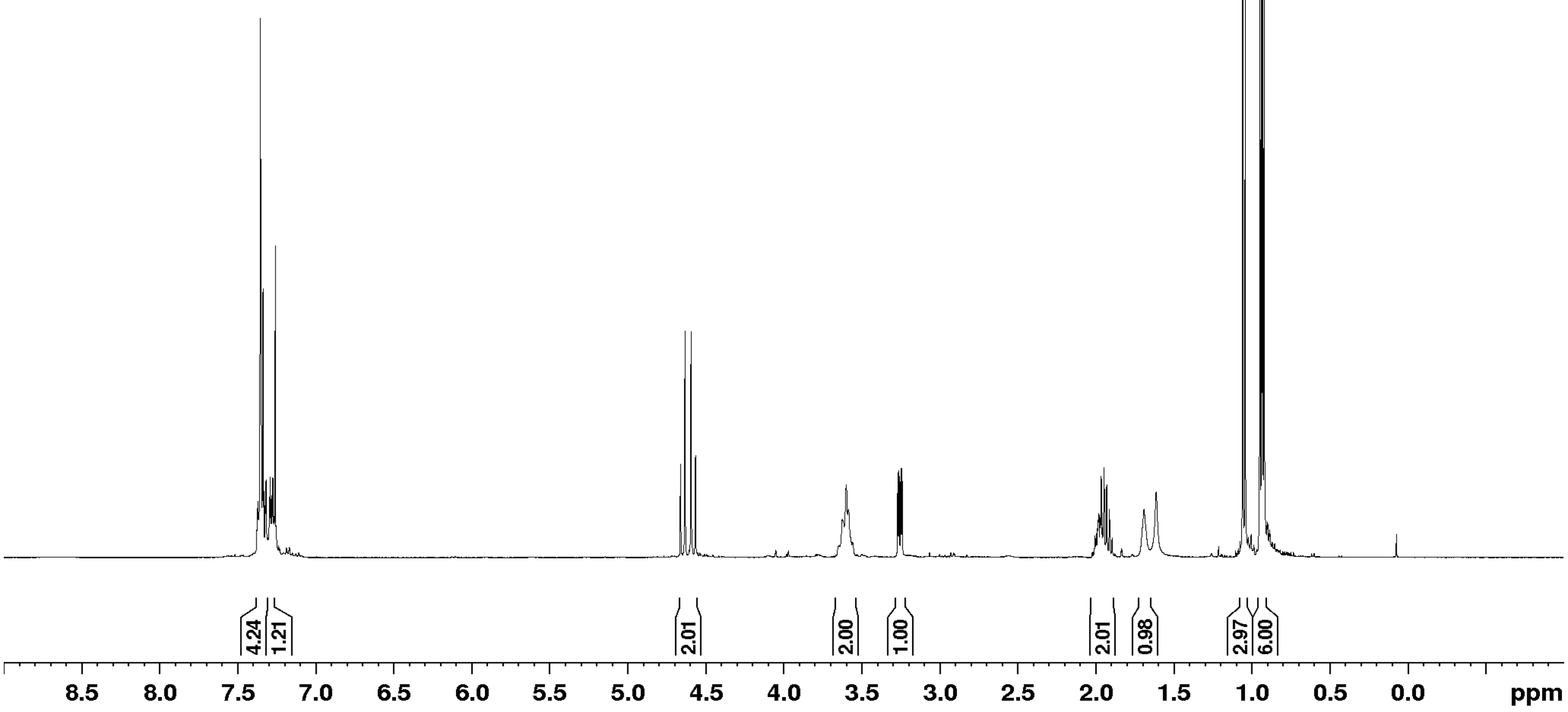

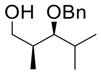

S3

$^{13}C\{^{1}H\}$ NMR (101 MHz, $CDCl_3$)

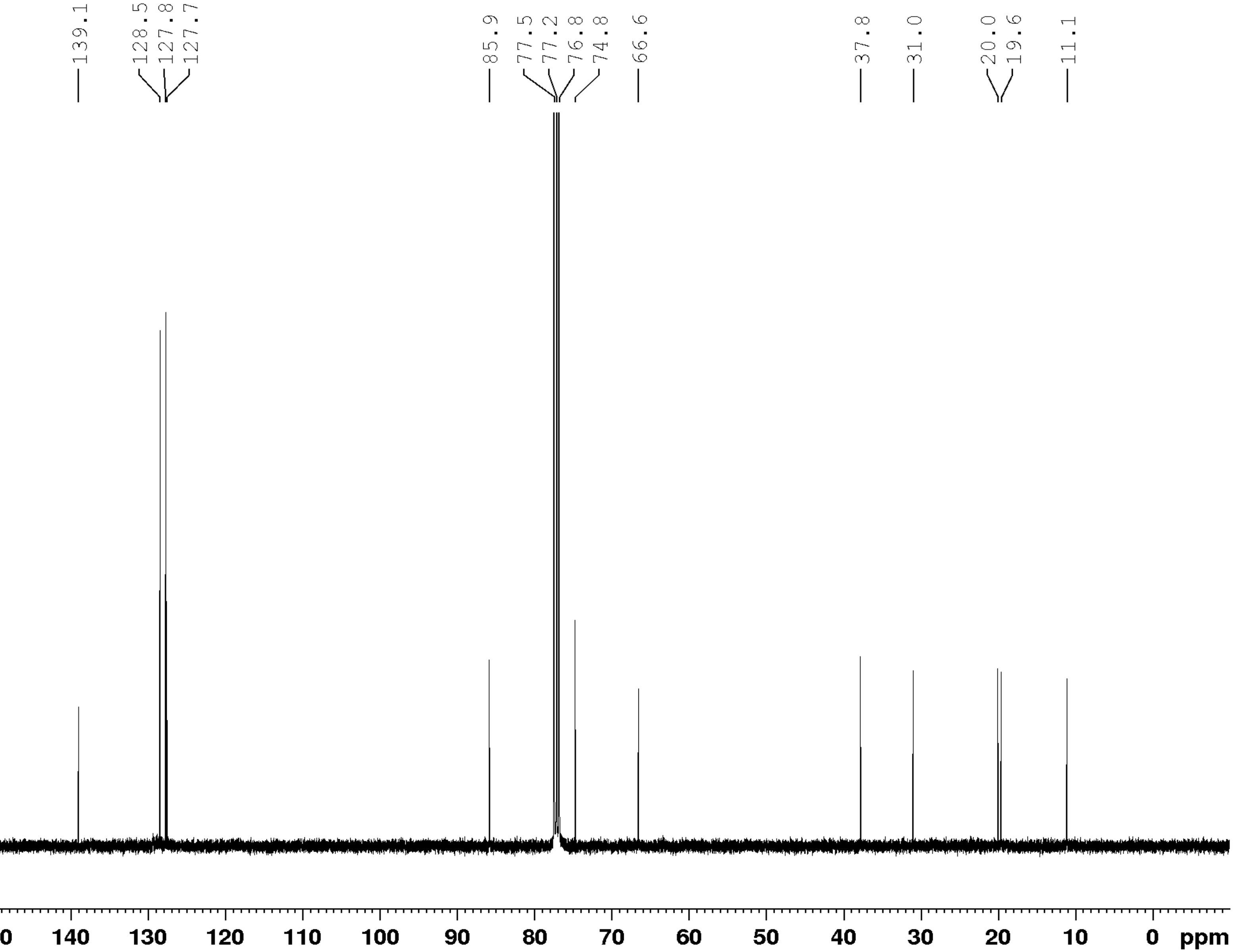

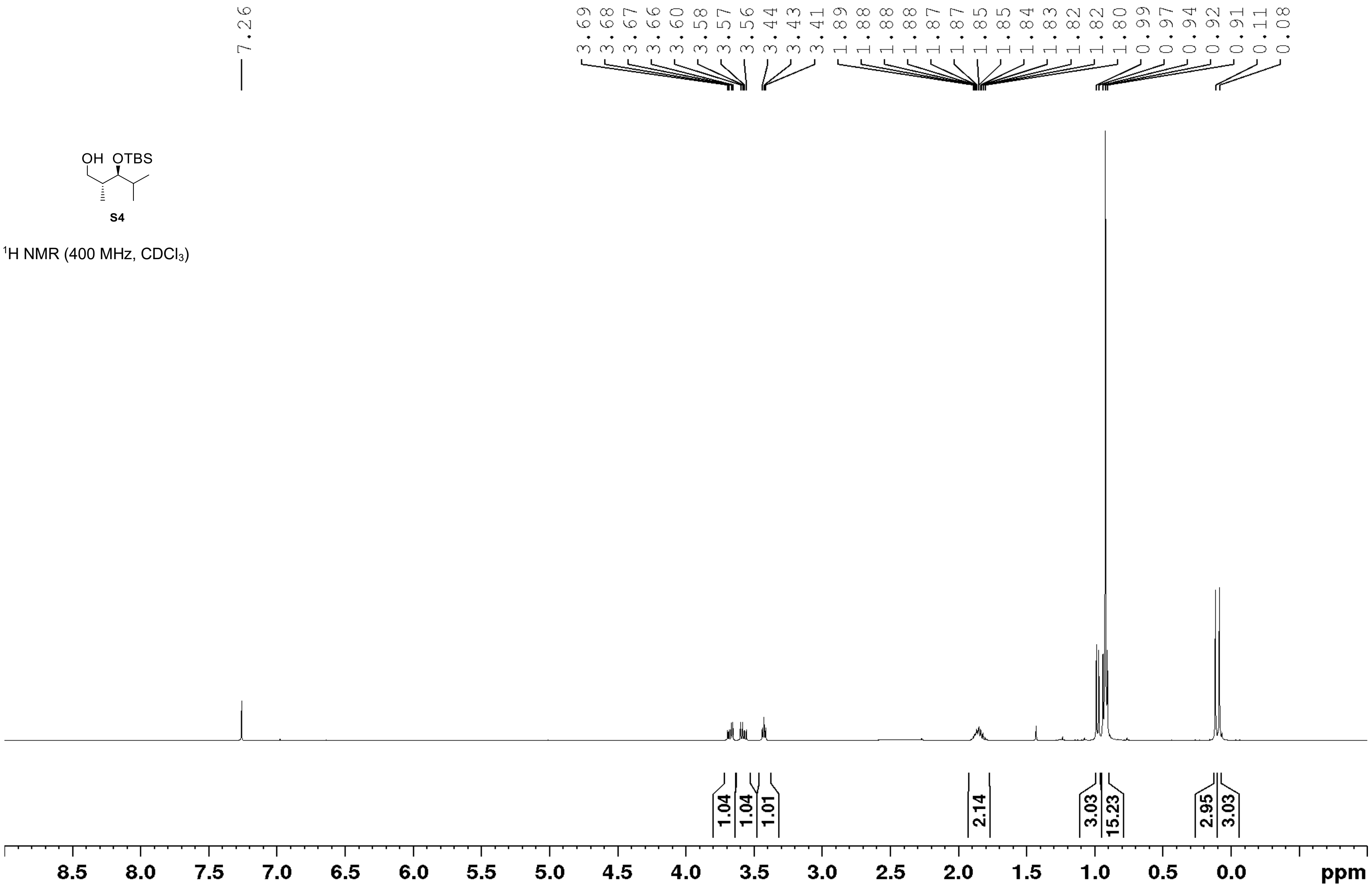
7.26
3.69 3.68 3.67 3.66 3.60 3.58 3.57 3.56 3.44 3.43 3.41 1.89 1.88 1.88 1.88 1.87 1.87 1.85 1.85 1.84 1.83 1.82 1.82 1.80 0.99 0.97 0.94 0.92 0.91 0.11 0.08
OH OTBS
S4
^{1}H NMR (400 MHz, $CDCl_3$)
1.04 1.04 1.01 2.14 3.03 15.23 2.95 3.03
8.5 8.0 7.5 7.0 6.5 6.0 5.5 5.0 4.5 4.0 3.5 3.0 2.5 2.0 1.5 1.0 0.5 0.0 ppm

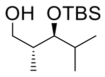

S4

^{13}C{^{1}H} NMR (101 MHz, $CDCl_3$)

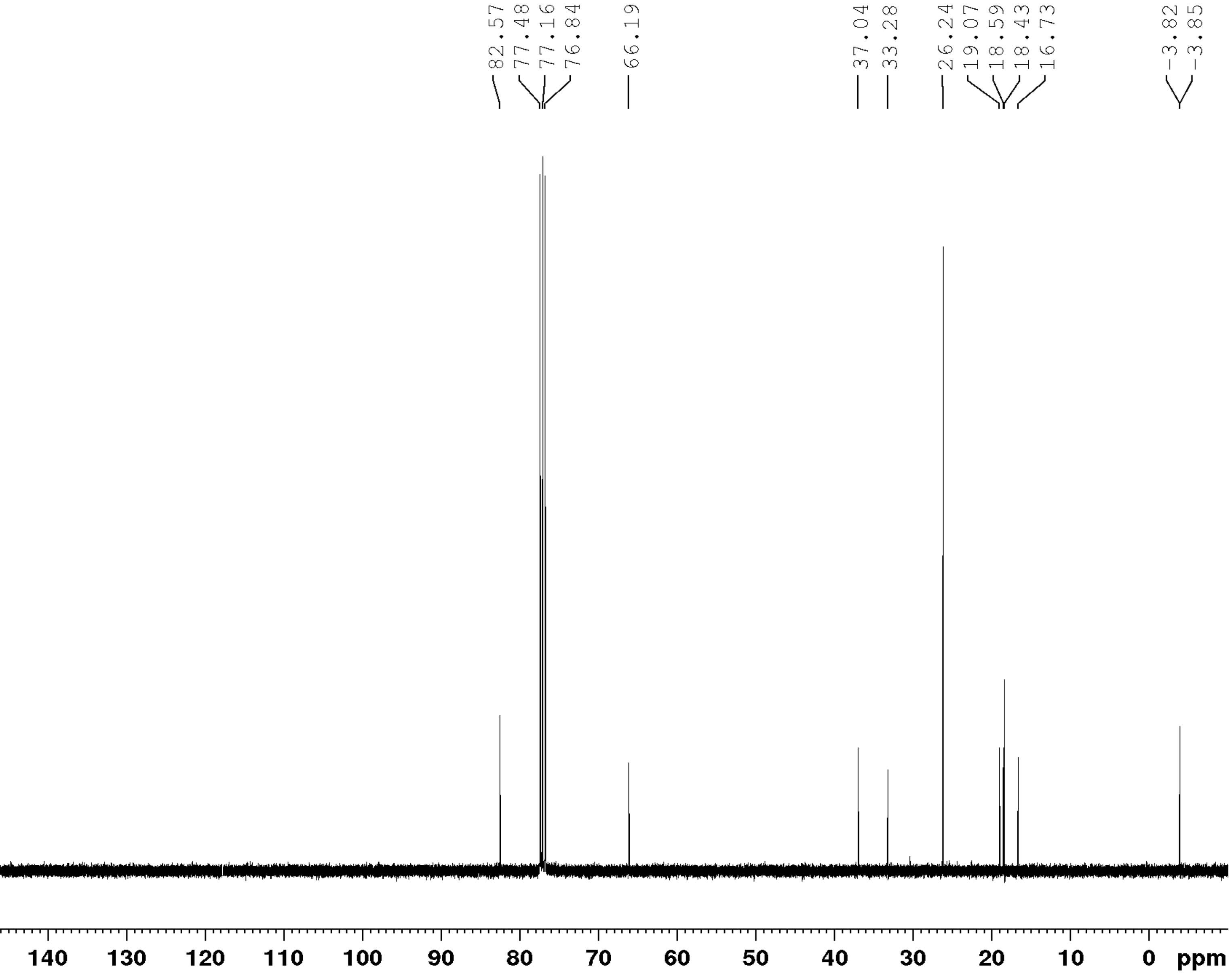

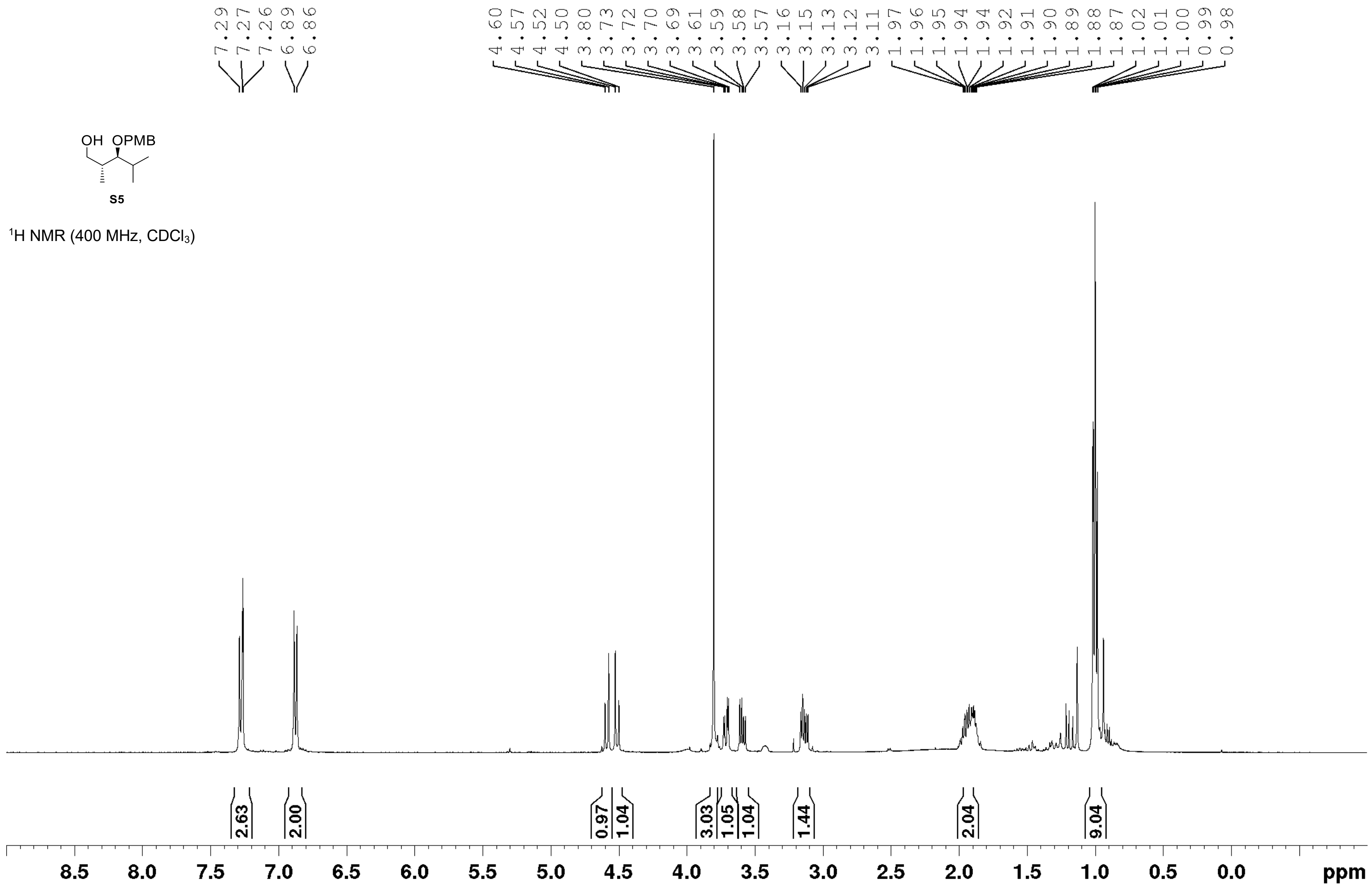
7.29
7.27
7.26
6.89
6.86
4.60
4.57
4.52
4.50
3.80
3.73
3.72
3.70
3.69
3.61
3.59
3.58
3.57
3.16
3.15
3.13
3.12
3.11
1.97
1.96
1.95
1.94
1.94
1.92
1.91
1.90
1.89
1.88
1.87
1.02
1.01
1.00
0.99
0.98
OH OPMB
S5
^{1}H NMR (400 MHz, $CDCl_3$)
2.63
2.00
0.97
1.04
3.03
1.05
1.04
1.44
2.04
9.04
8.5
8.0
7.5
7.0
6.5
6.0
5.5
5.0
4.5
4.0
3.5
3.0
2.5
2.0
1.5
1.0
0.5
0.0
ppm

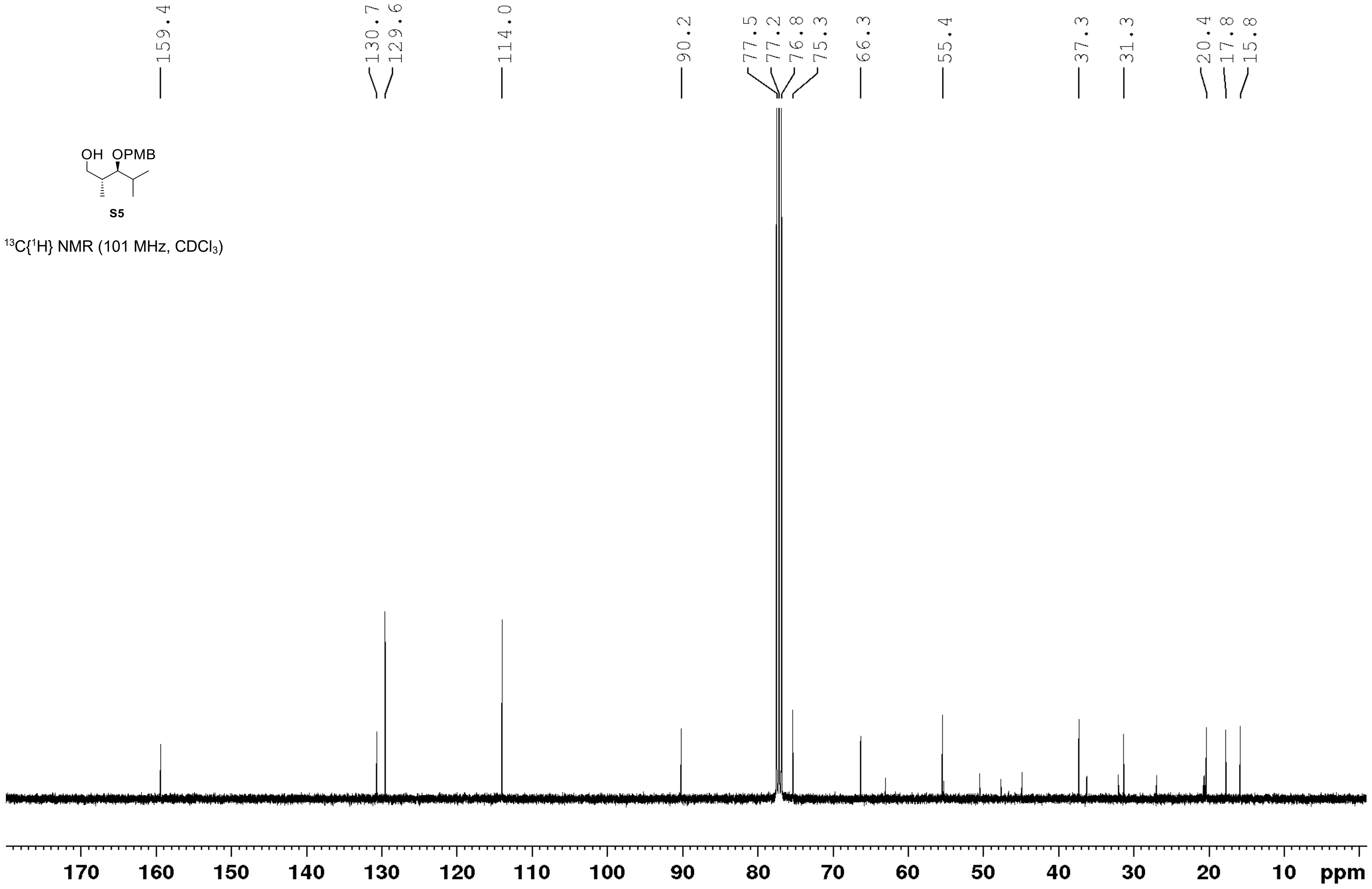
OH OPMB
S5
$^{13}C\{^1H\}$ NMR (101 MHz, CDCl$_3$)
159.4
130.7
129.6
114.0
90.2
77.5
77.2
76.8
75.3
66.3
55.4
37.3
31.3
20.4
17.8
15.8
170 160 150 140 130 120 110 100 90 80 70 60 50 40 30 20 10 ppm

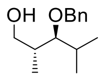

S6

^{1}H NMR (400 MHz, $CDCl_3$)

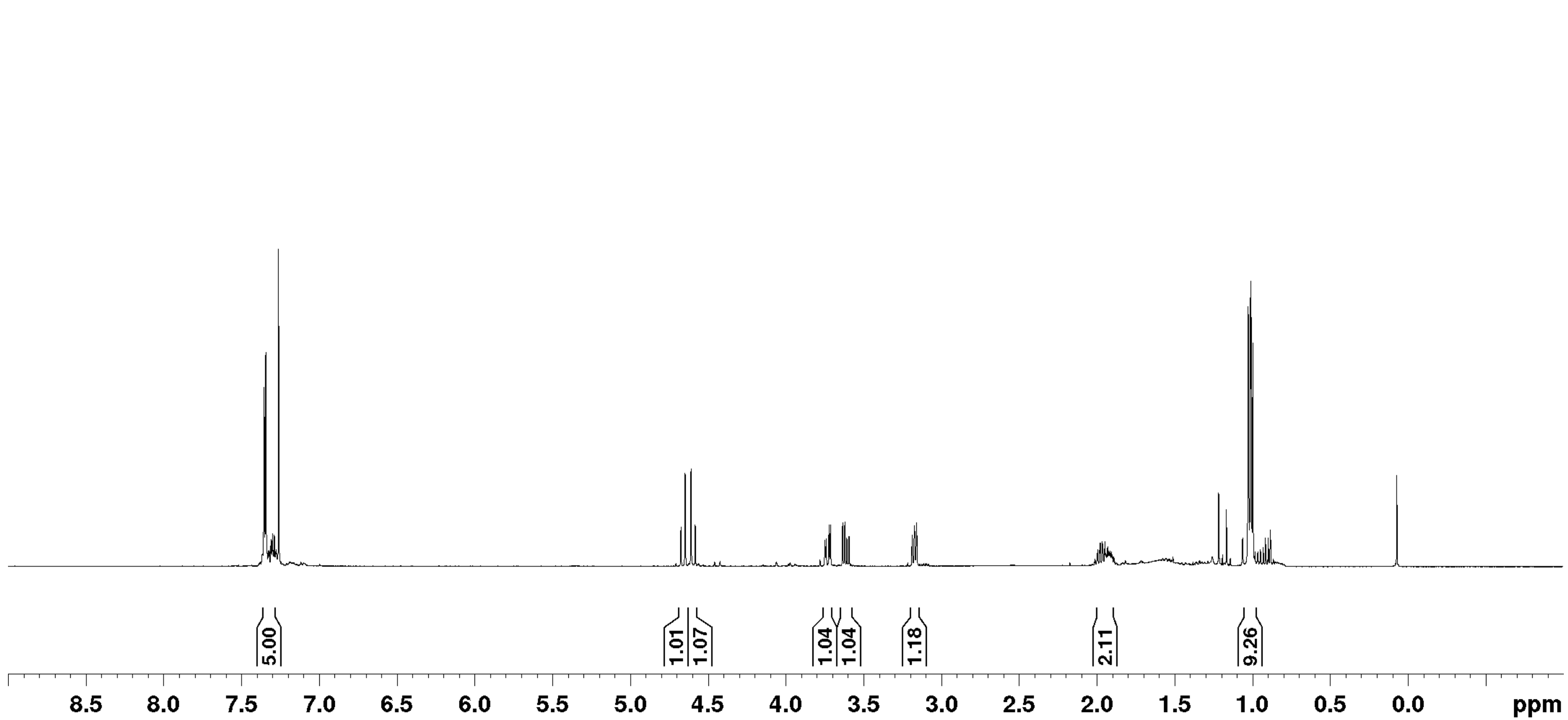

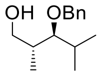

S6

$^{13}C\{^1H\}$ NMR (101 MHz, $CDCl_3$)

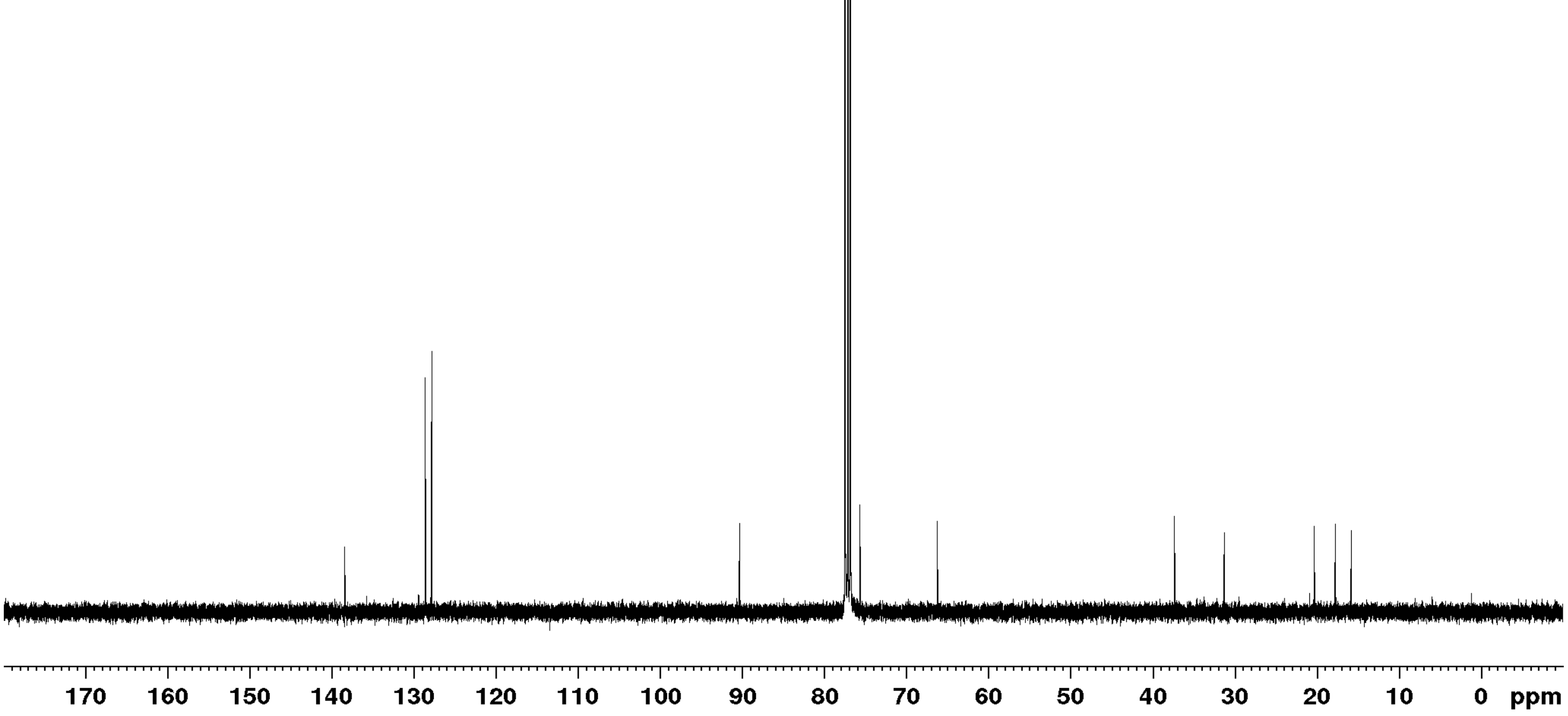

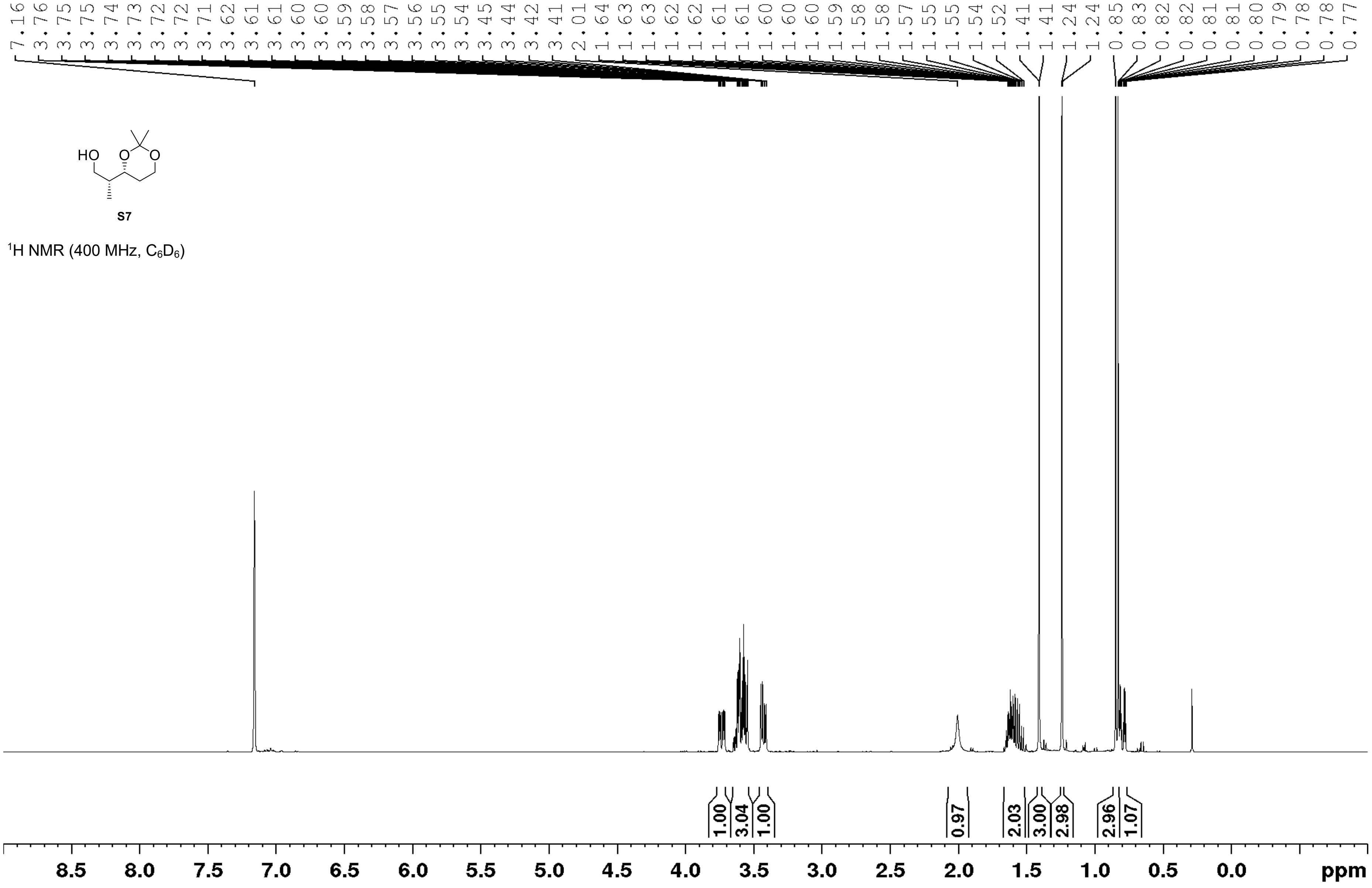

S7

^{1}H NMR (400 MHz, C_6D_6)

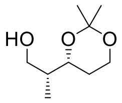

S7

$^{13}C\{^{1}H\}$ NMR (101 MHz, C_6D_6)

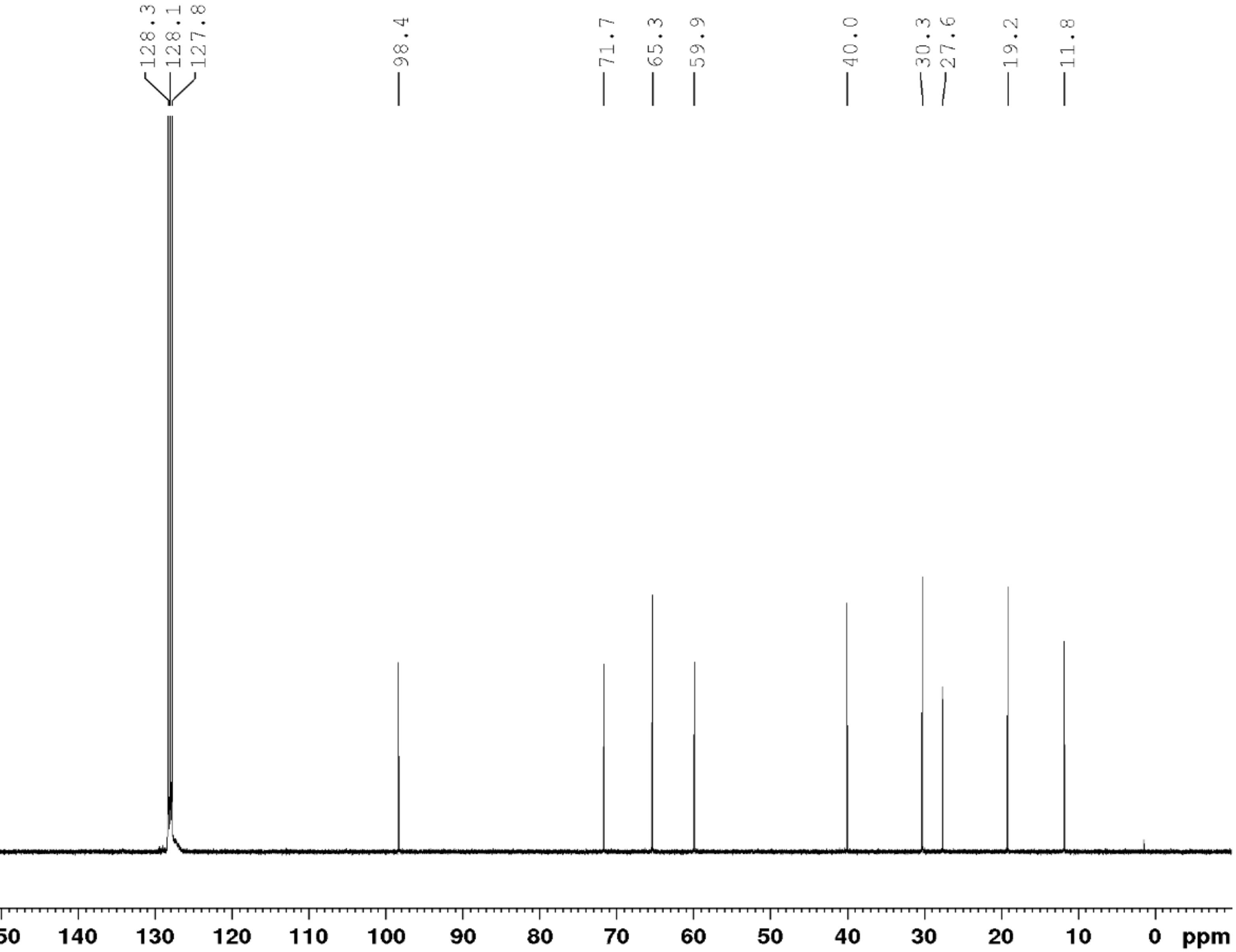

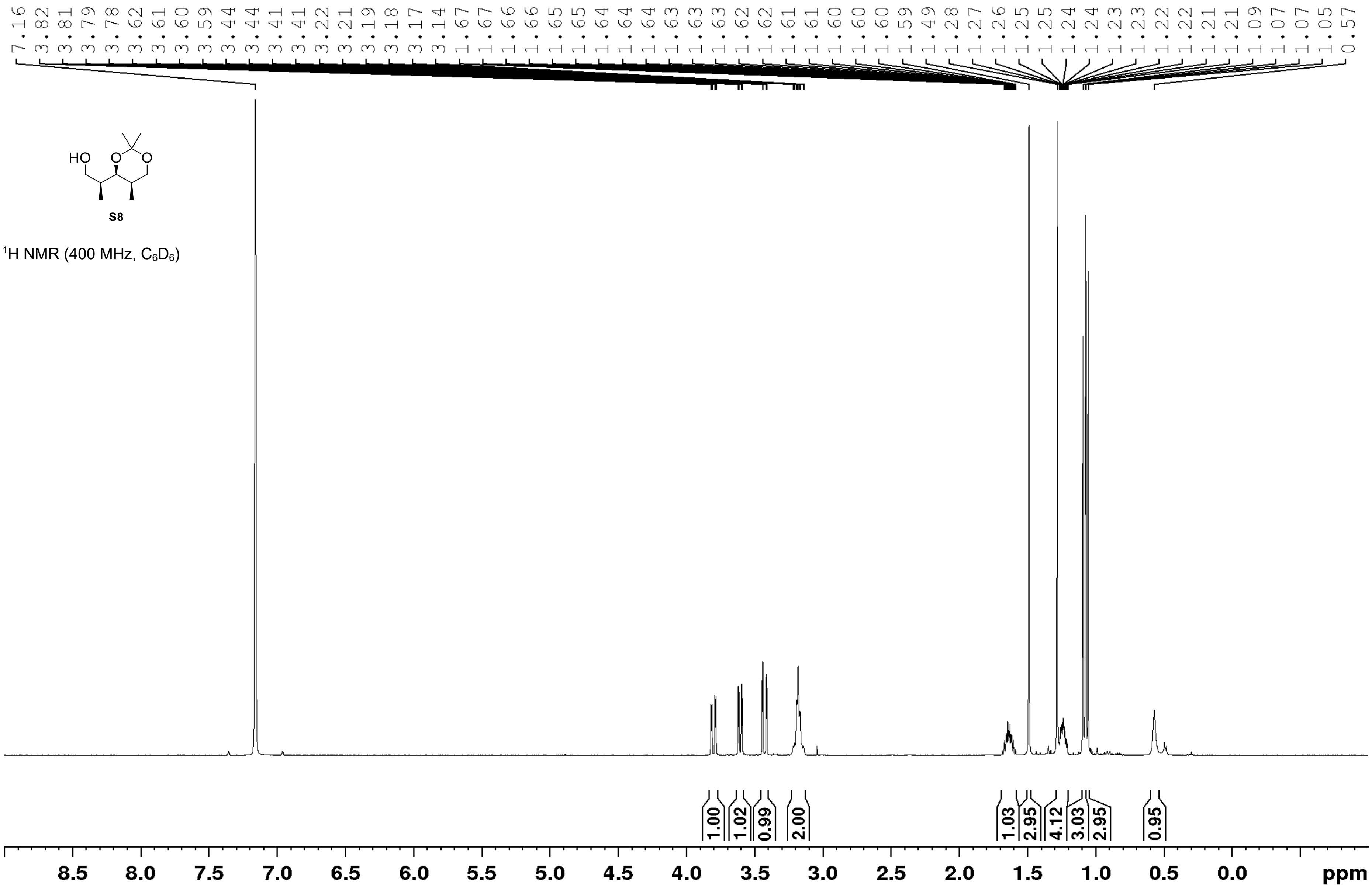
7.16
3.82
3.81
3.79
3.78
3.62
3.61
3.60
3.59
3.44
3.44
3.41
3.41
3.22
3.21
3.19
3.18
3.17
3.14
1.67
1.67
1.66
1.66
1.65
1.65
1.64
1.64
1.64
1.63
1.63
1.63
1.62
1.62
1.61
1.61
1.60
1.60
1.60
1.59
1.49
1.28
1.27
1.26
1.25
1.25
1.24
1.24
1.23
1.23
1.22
1.22
1.21
1.21
1.09
1.07
1.07
1.05
0.57
HO
O
O
S8
^{1}H NMR (400 MHz, C_6D_6)
1.00
1.02
0.99
2.00
1.03
2.95
4.12
3.03
2.95
0.95
8.5
8.0
7.5
7.0
6.5
6.0
5.5
5.0
4.5
4.0
3.5
3.0
2.5
2.0
1.5
1.0
0.5
0.0
ppm

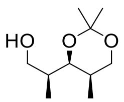

S8

$^{13}C\{^{1}H\}$ NMR (101 MHz, C_6D_6)

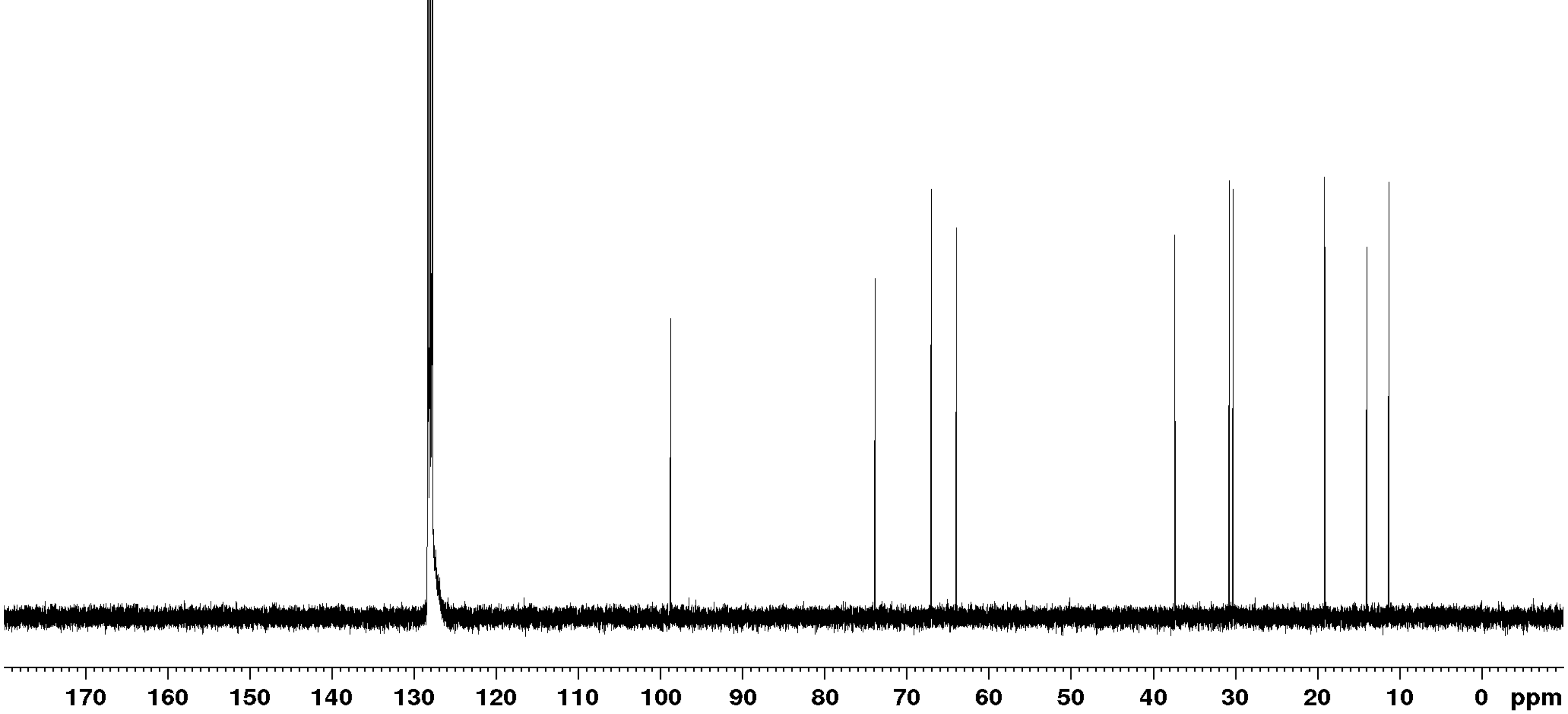

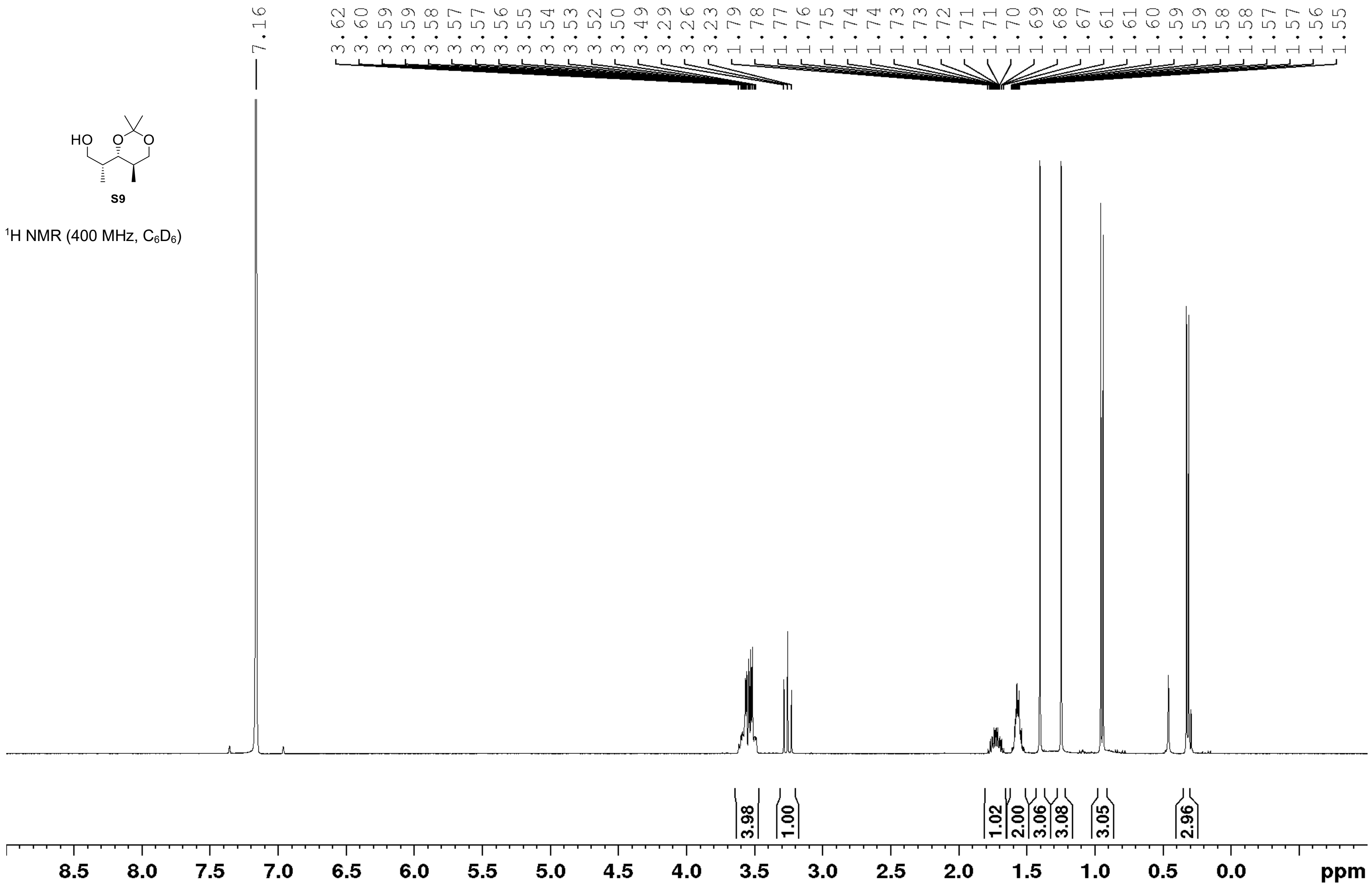
HO
O
O
S9
¹H NMR (400 MHz, C₆D₆)
7.16
3.62
3.60
3.59
3.59
3.58
3.57
3.57
3.56
3.55
3.54
3.53
3.52
3.50
3.49
3.29
3.26
3.23
1.79
1.78
1.77
1.76
1.75
1.74
1.74
1.73
1.73
1.72
1.71
1.71
1.70
1.69
1.68
1.67
1.61
1.61
1.60
1.59
1.59
1.58
1.58
1.57
1.57
1.56
1.55
3.98
1.00
1.02
2.00
3.06
3.08
3.05
2.96
8.5
8.0
7.5
7.0
6.5
6.0
5.5
5.0
4.5
4.0
3.5
3.0
2.5
2.0
1.5
1.0
0.5
0.0
ppm

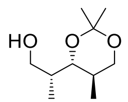

S9

$^{13}C\{^{1}H\}$ NMR (101 MHz, C_6D_6)

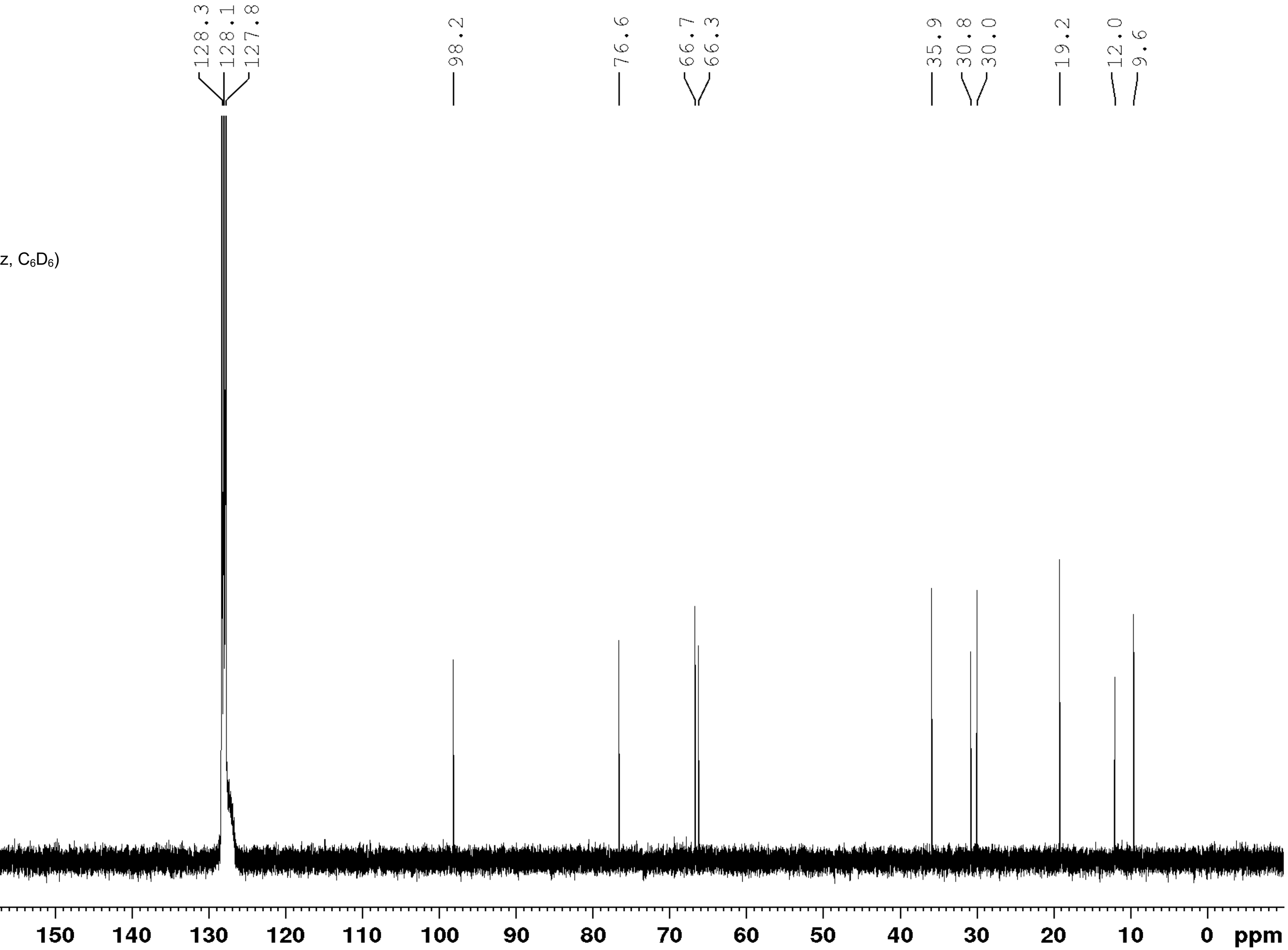

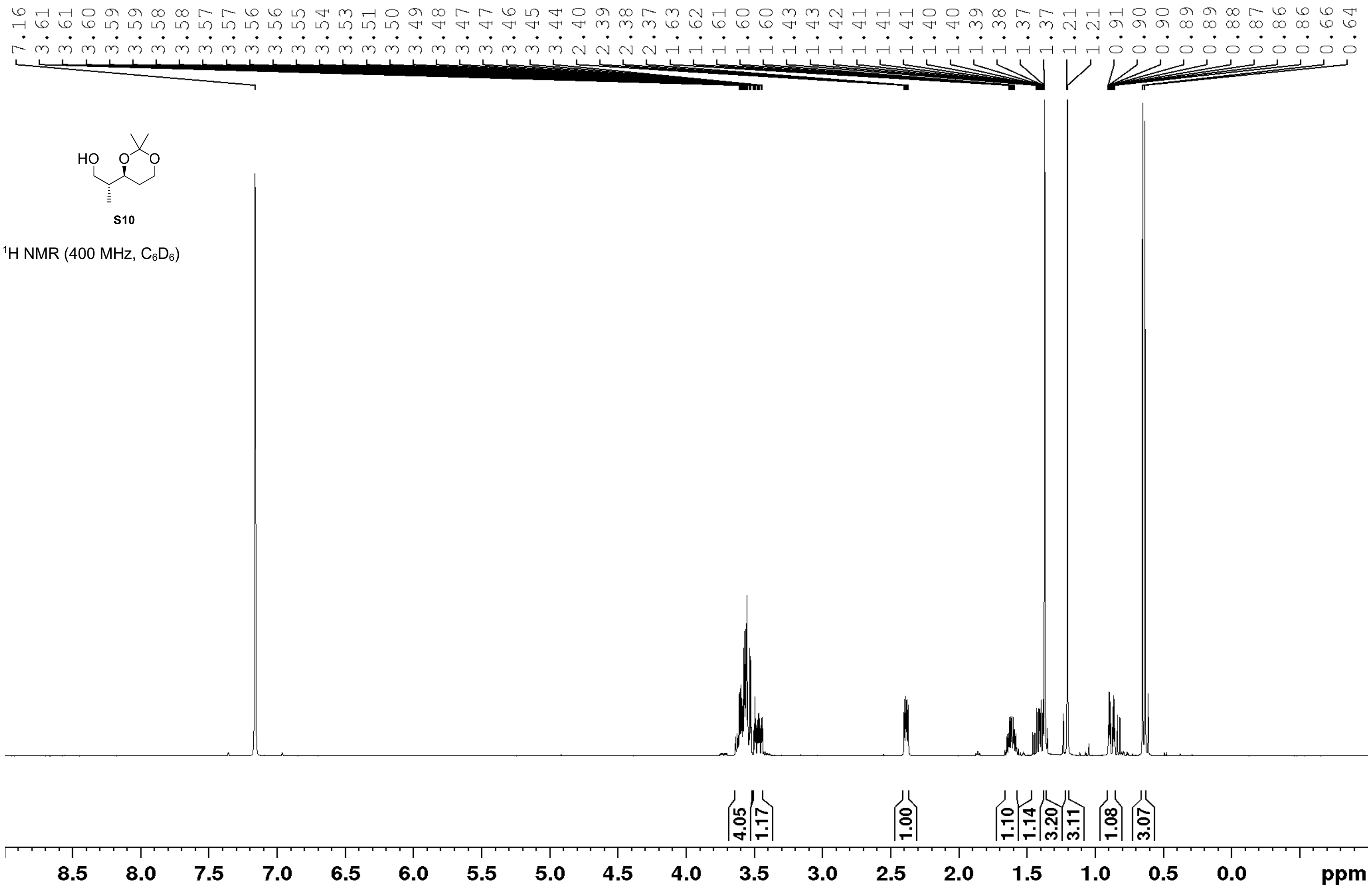
7.16 3.61 3.61 3.60 3.59 3.59 3.58 3.58 3.57 3.57 3.56 3.56 3.55 3.54 3.53 3.51 3.50 3.49 3.48 3.47 3.47 3.46 3.45 3.44 2.40 2.39 2.38 2.37 1.63 1.62 1.61 1.60 1.60 1.43 1.43 1.42 1.41 1.41 1.41 1.40 1.40 1.39 1.38 1.37 1.37 1.21 1.21 0.91 0.90 0.90 0.89 0.89 0.88 0.87 0.86 0.86 0.66 0.64
HO
O
O
S10
1H NMR (400 MHz, C6D6)
4.05 1.17 1.00 1.10 1.14 3.20 3.11 1.08 3.07
8.5 8.0 7.5 7.0 6.5 6.0 5.5 5.0 4.5 4.0 3.5 3.0 2.5 2.0 1.5 1.0 0.5 0.0 ppm

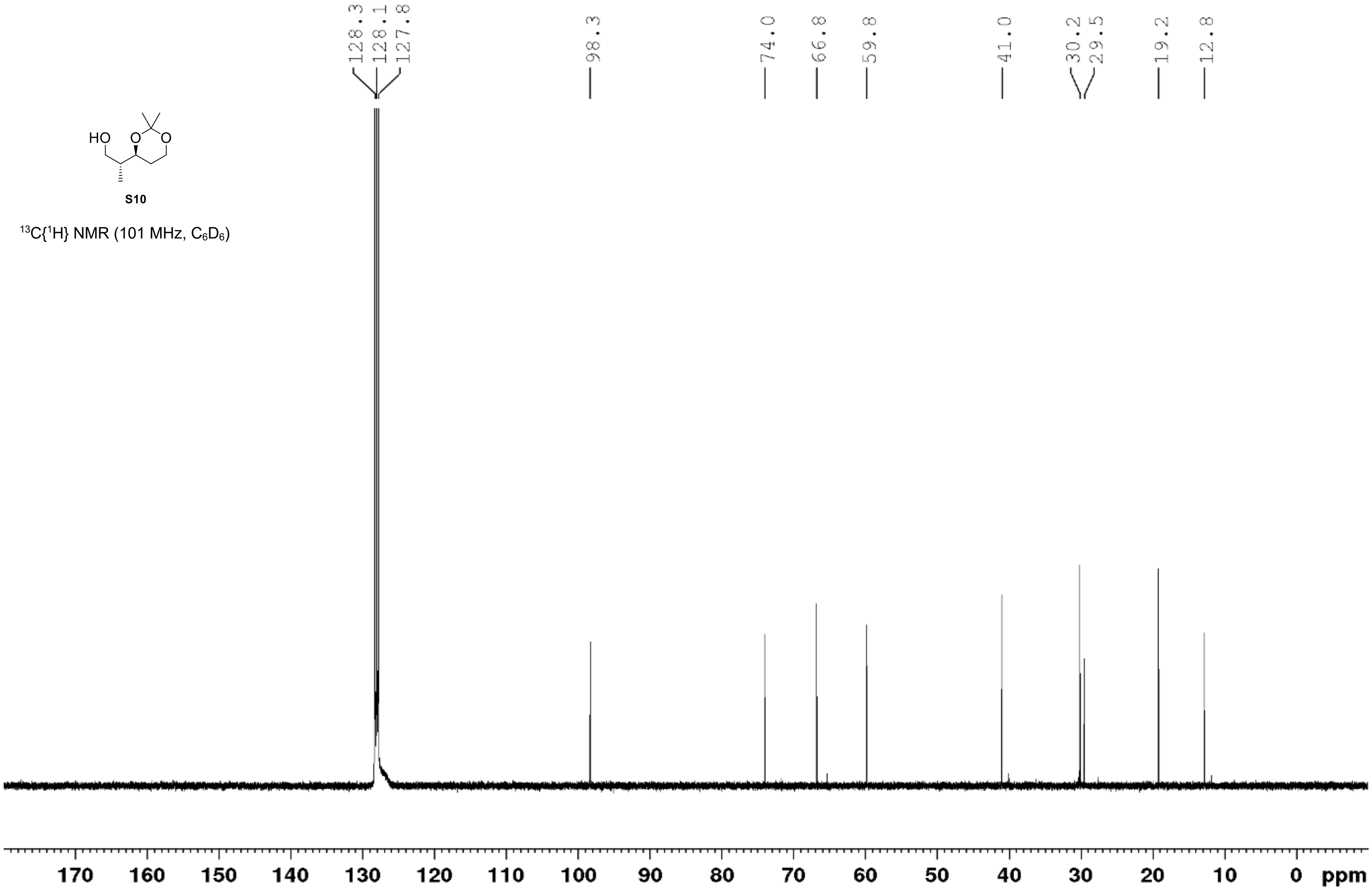

S10

$^{13}C\{^{1}H\}$ NMR (101 MHz, C_6D_6)

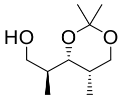

S11

^{1}H NMR (400 MHz, C_6D_6)

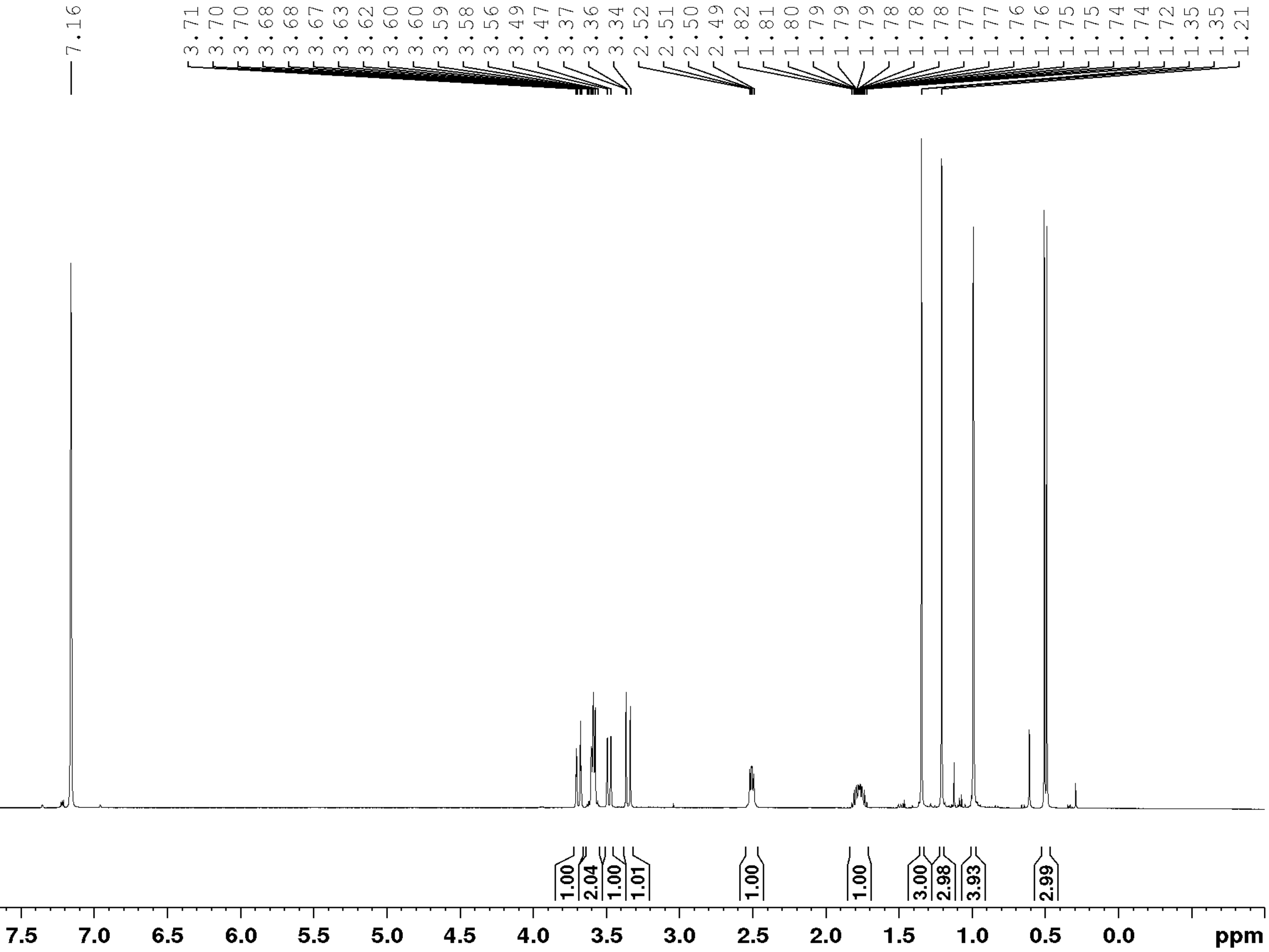

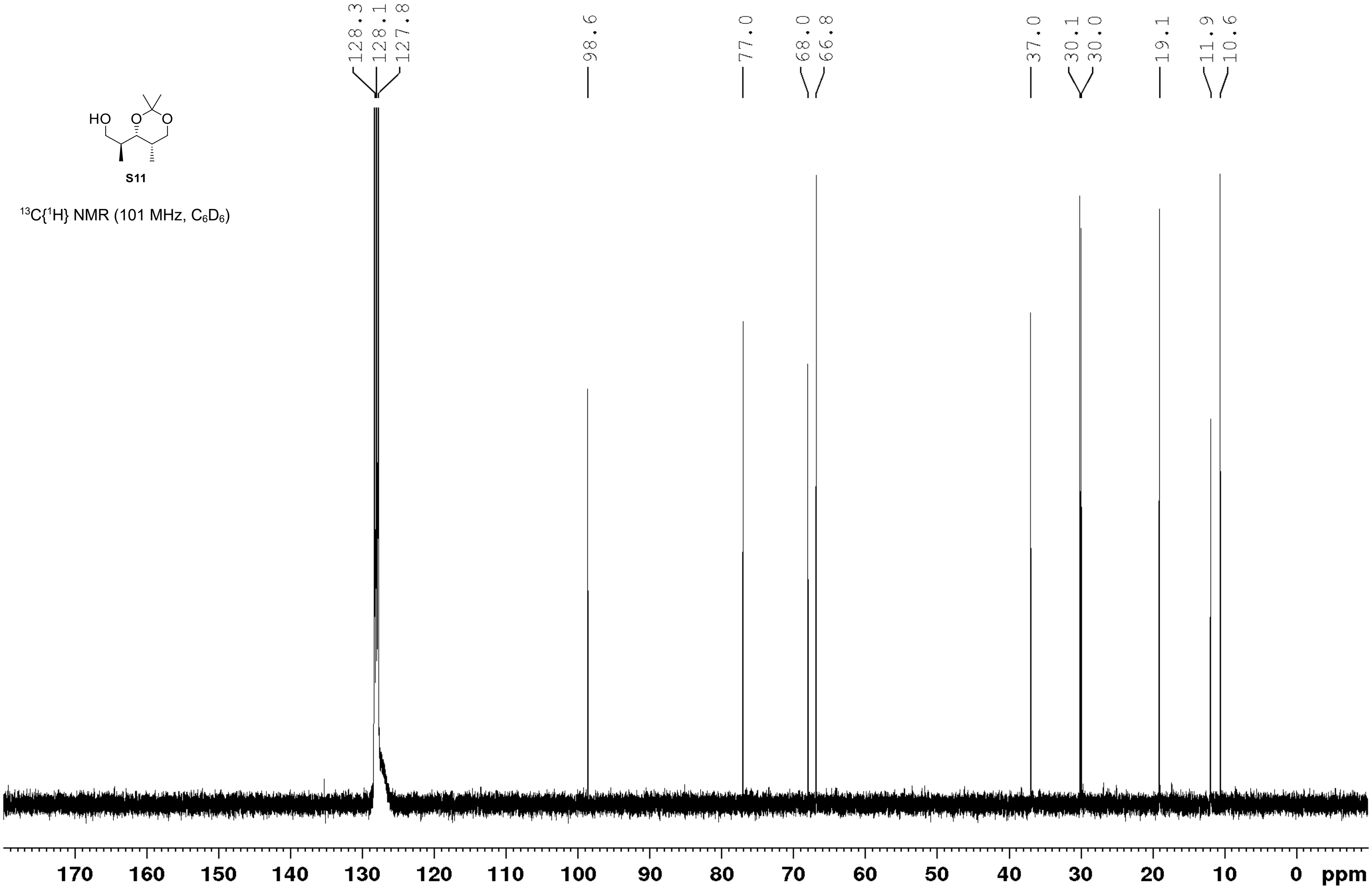

$^{13}C\{^{1}H\}$ NMR (101 MHz, C_6D_6)

S12

^1H NMR (400 MHz, C_6D_6)

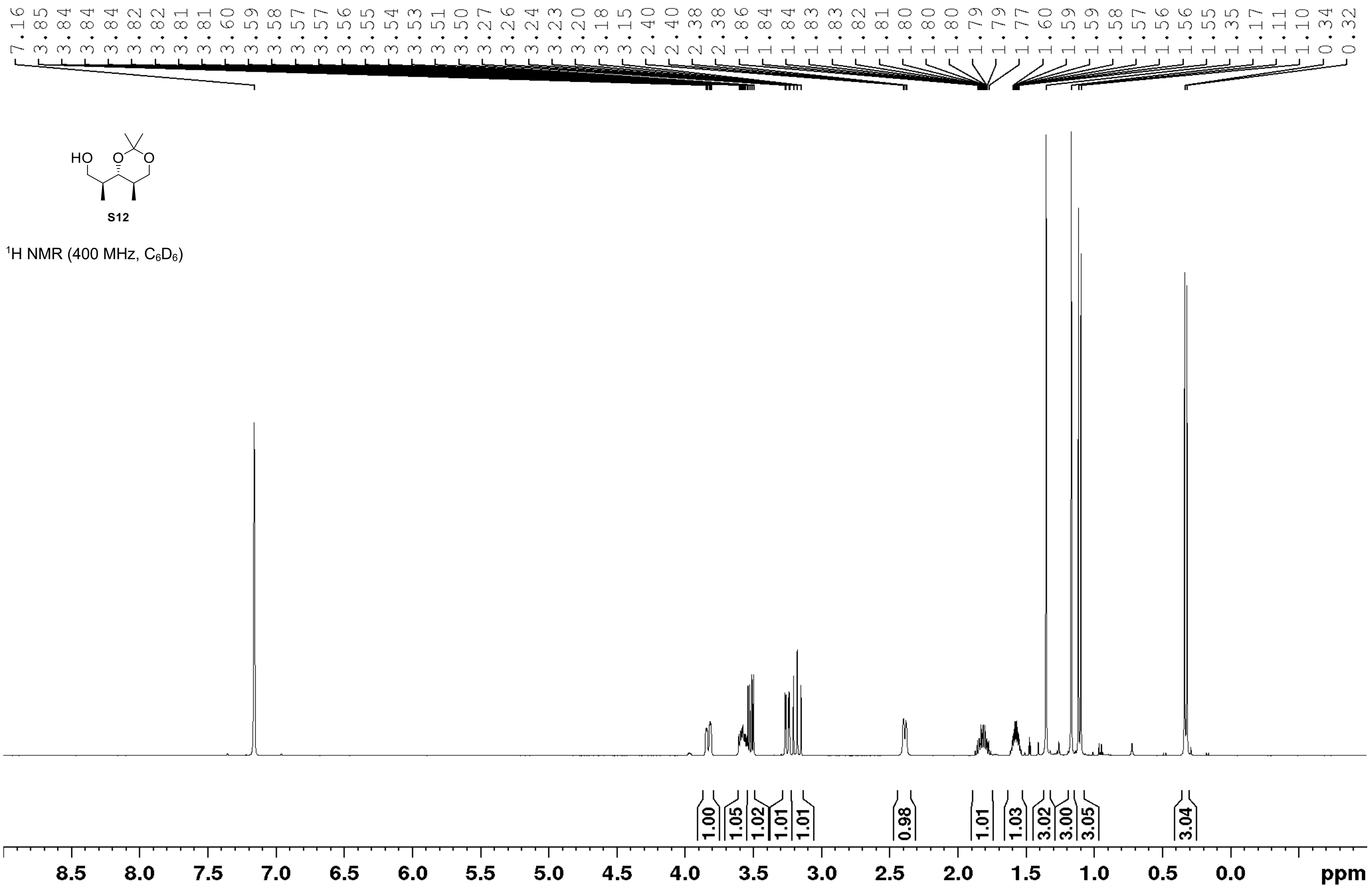

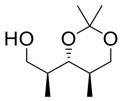

S12

$^{13}C\{^1H\}$ NMR (101 MHz, C_6D_6)

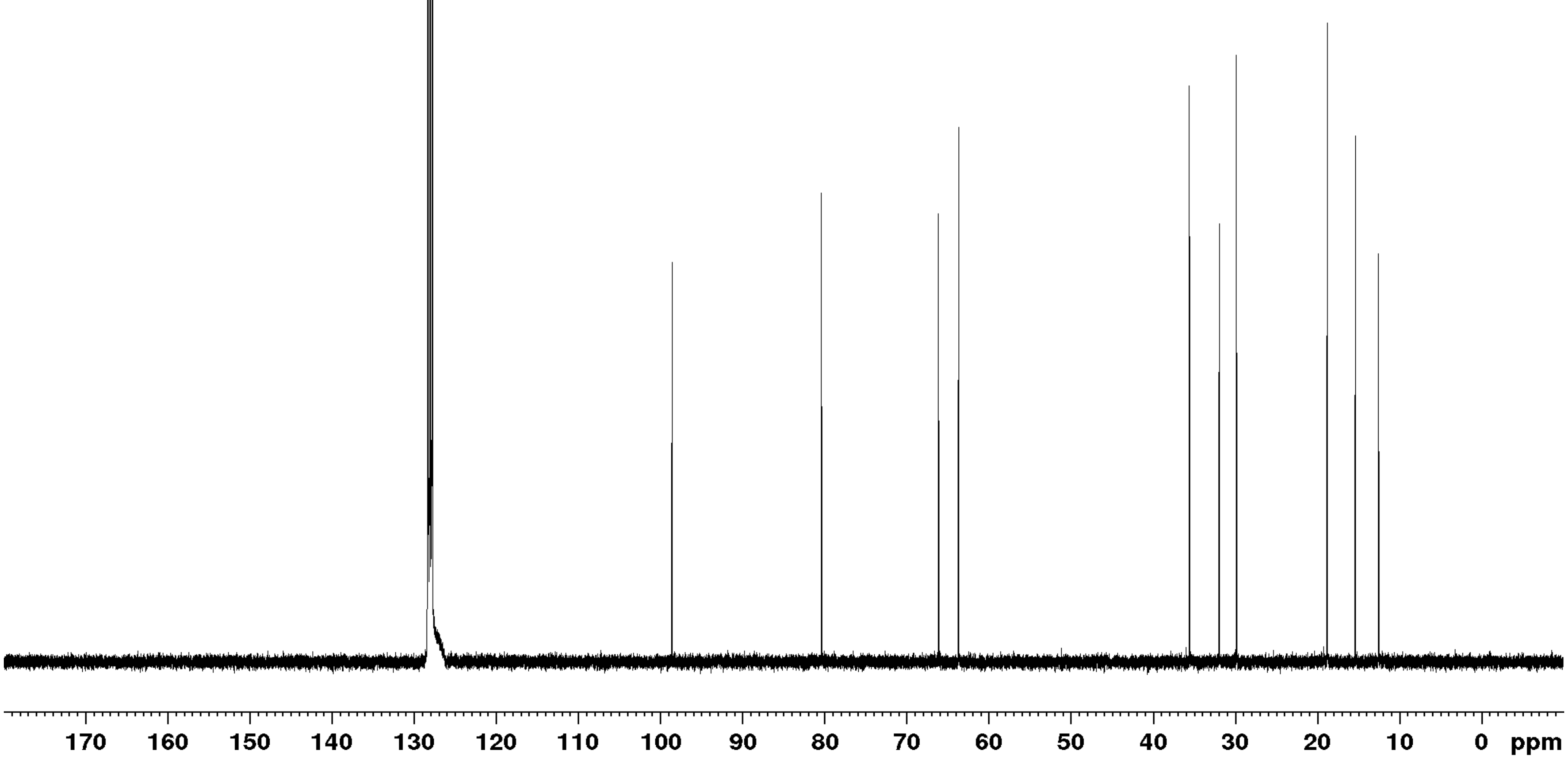

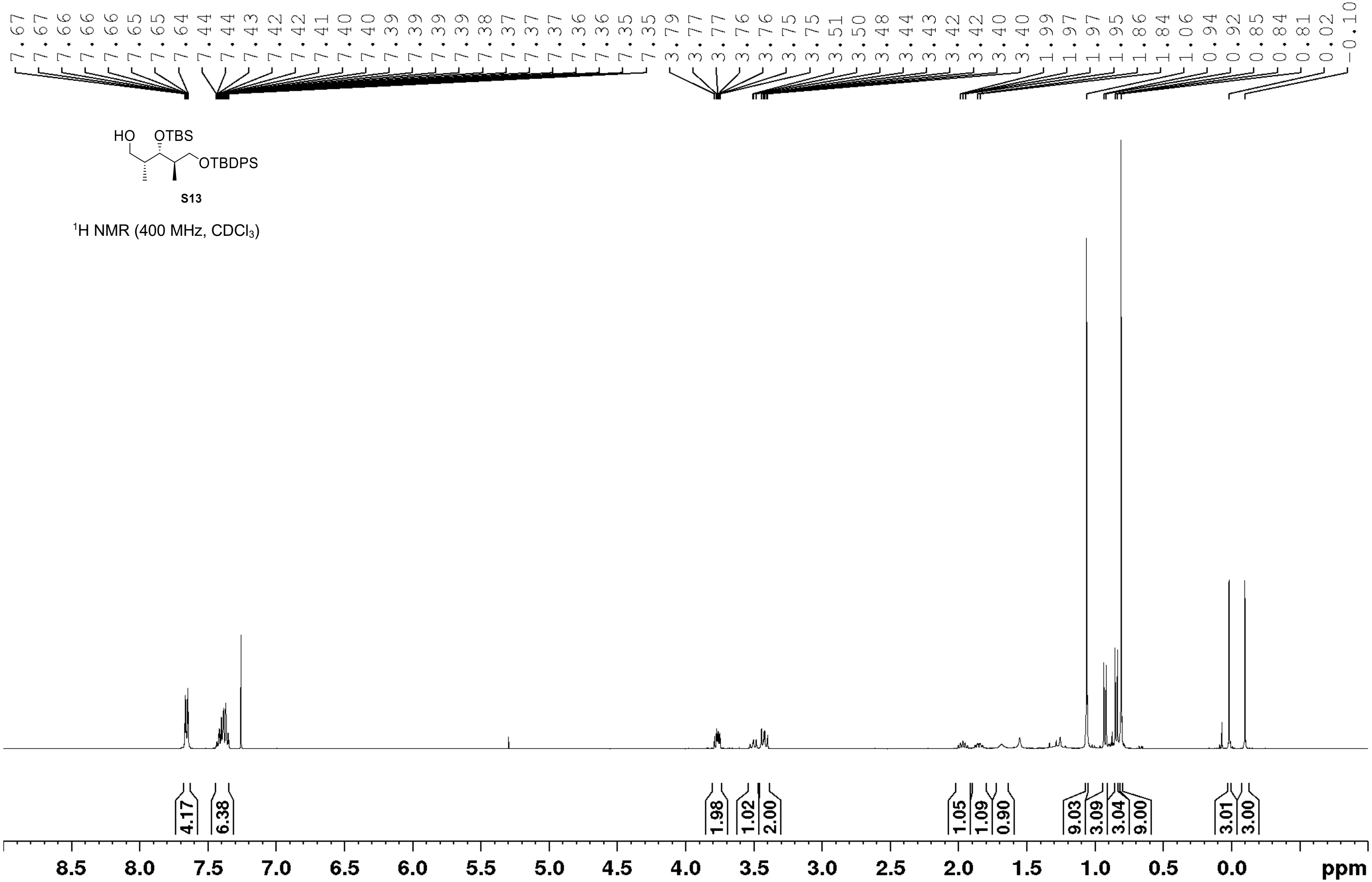

S13

^{1}H NMR (400 MHz, $CDCl_3$)

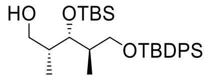

S13

$^{13}C\{^{1}H\}$ NMR (101 MHz, $CDCl_3$)

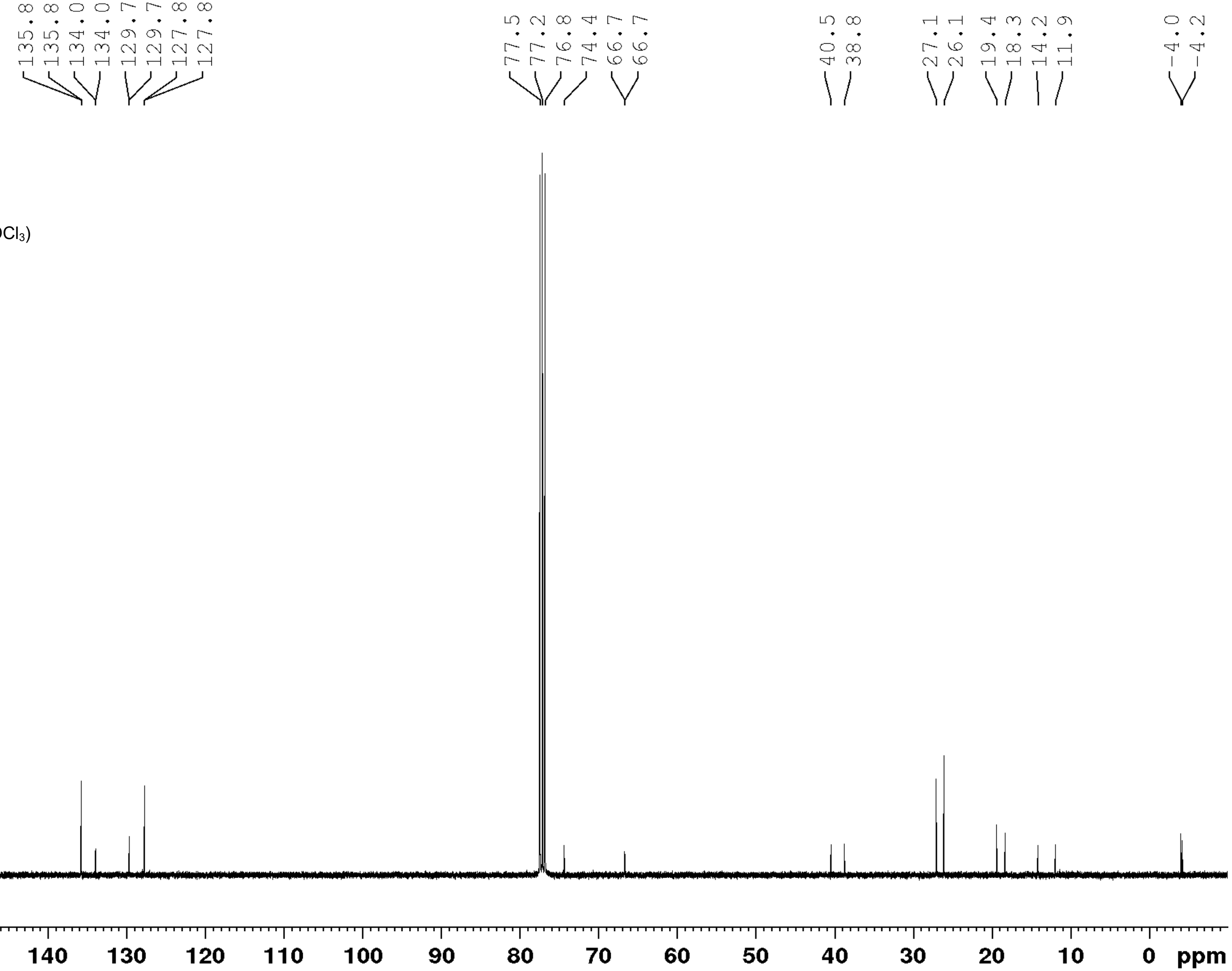

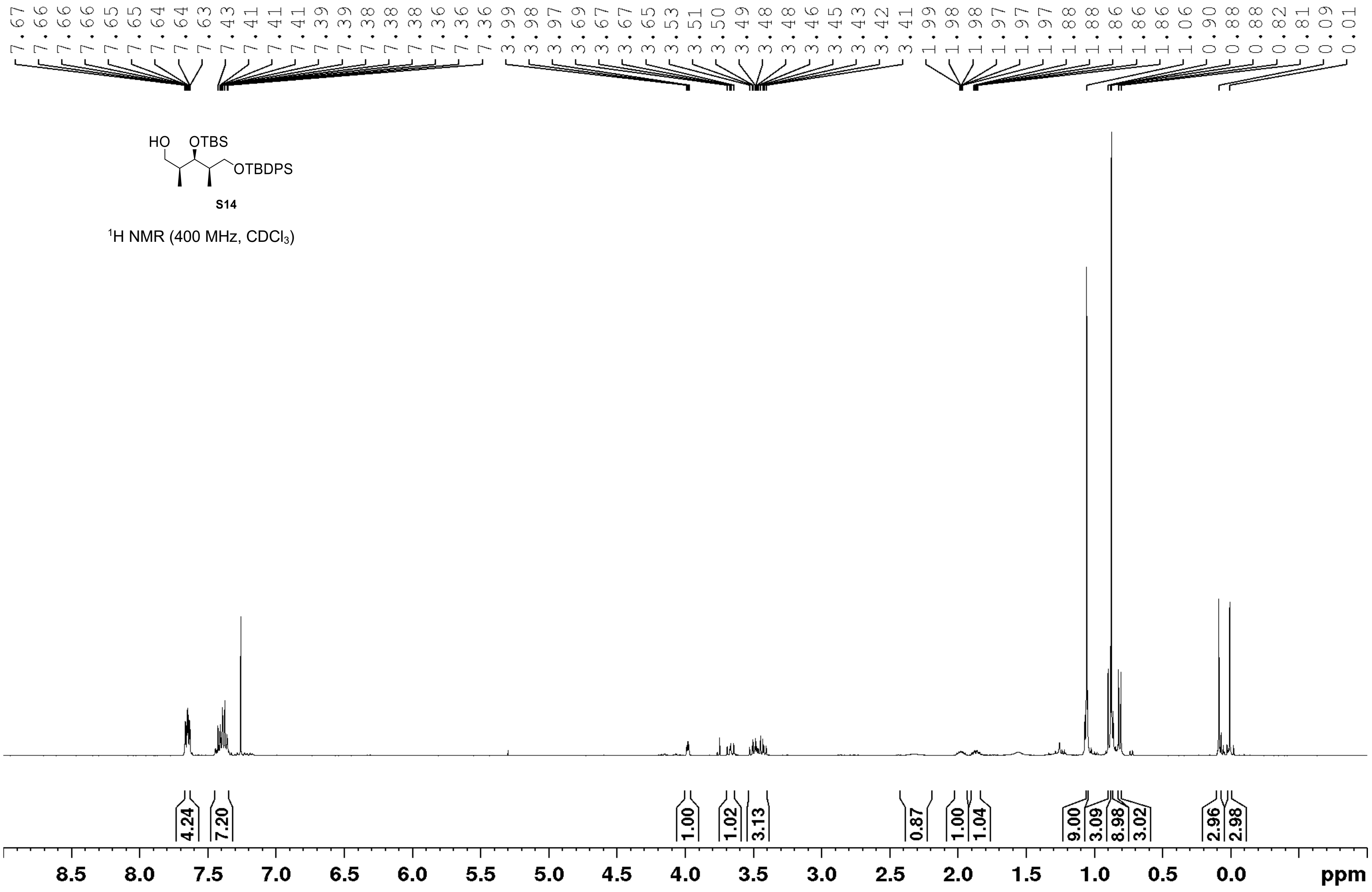
7.67 7.66 7.66 7.66 7.65 7.65 7.64 7.64 7.63 7.43 7.41 7.41 7.41 7.39 7.39 7.38 7.38 7.38 7.36 7.36 7.36 3.99 3.98 3.97 3.69 3.67 3.65 3.53 3.51 3.50 3.49 3.48 3.48 3.46 3.45 3.43 3.42 3.41 1.99 1.98 1.98 1.97 1.97 1.97 1.88 1.88 1.86 1.86 1.86 1.06 0.90 0.88 0.88 0.82 0.81 0.09 0.01
HO
OTBS
OTBDPS
S14
^{1}H NMR (400 MHz, CDCl$_3$)
4.24
7.20
1.00
1.02
3.13
0.87
1.00
1.04
9.00
3.09
8.98
3.02
2.96
2.98
8.5 8.0 7.5 7.0 6.5 6.0 5.5 5.0 4.5 4.0 3.5 3.0 2.5 2.0 1.5 1.0 0.5 0.0 ppm

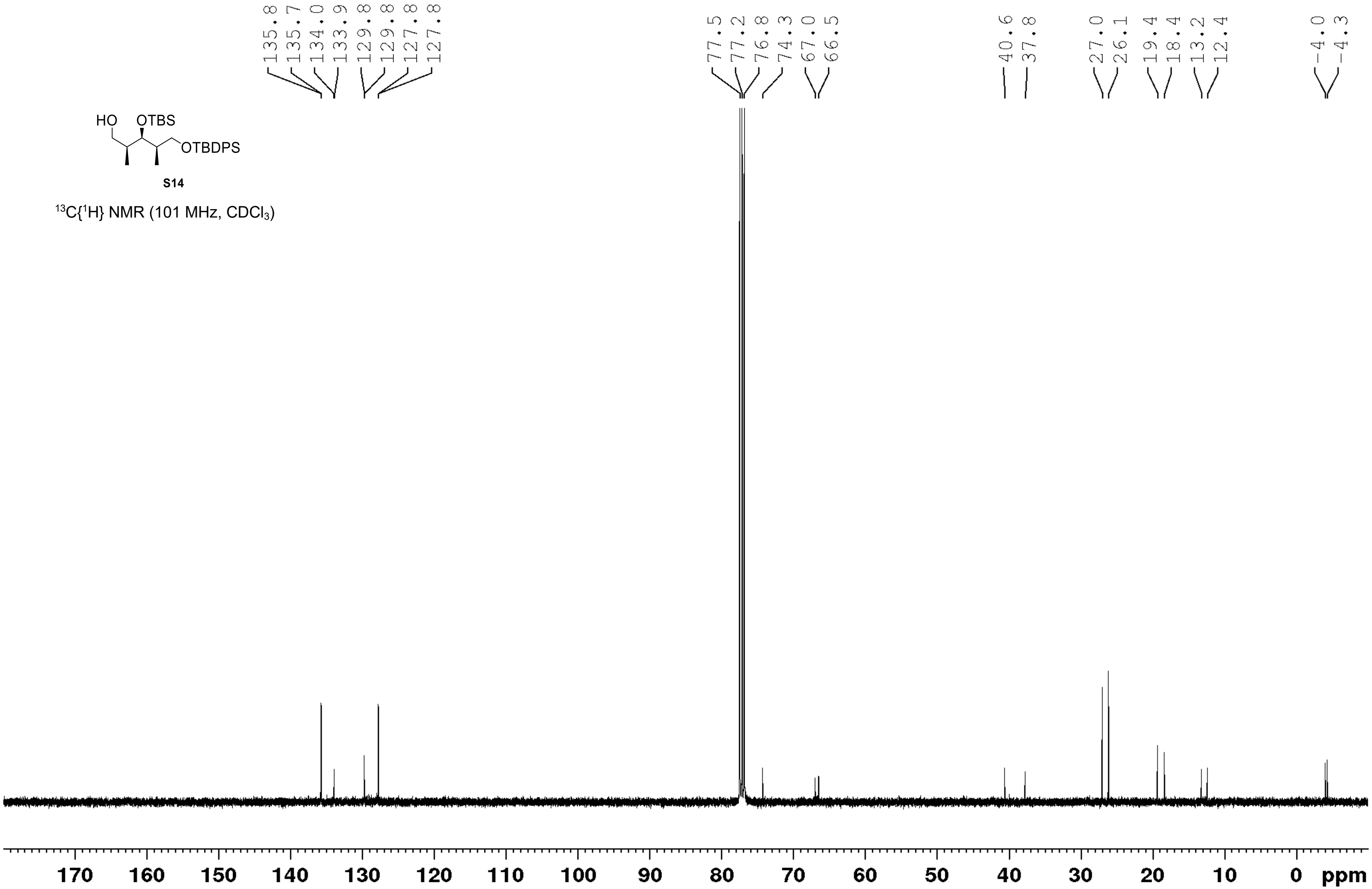
HO
OTBS
OTBDPS
S14
$^{13}C\{^{1}H\}$ NMR (101 MHz, $CDCl_3$)
135.8
135.7
134.0
133.9
129.8
129.8
127.8
127.8
77.5
77.2
76.8
74.3
67.0
66.5
40.6
37.8
27.0
26.1
19.4
18.4
13.2
12.4
-4.0
-4.3
170 160 150 140 130 120 110 100 90 80 70 60 50 40 30 20 10 0 ppm

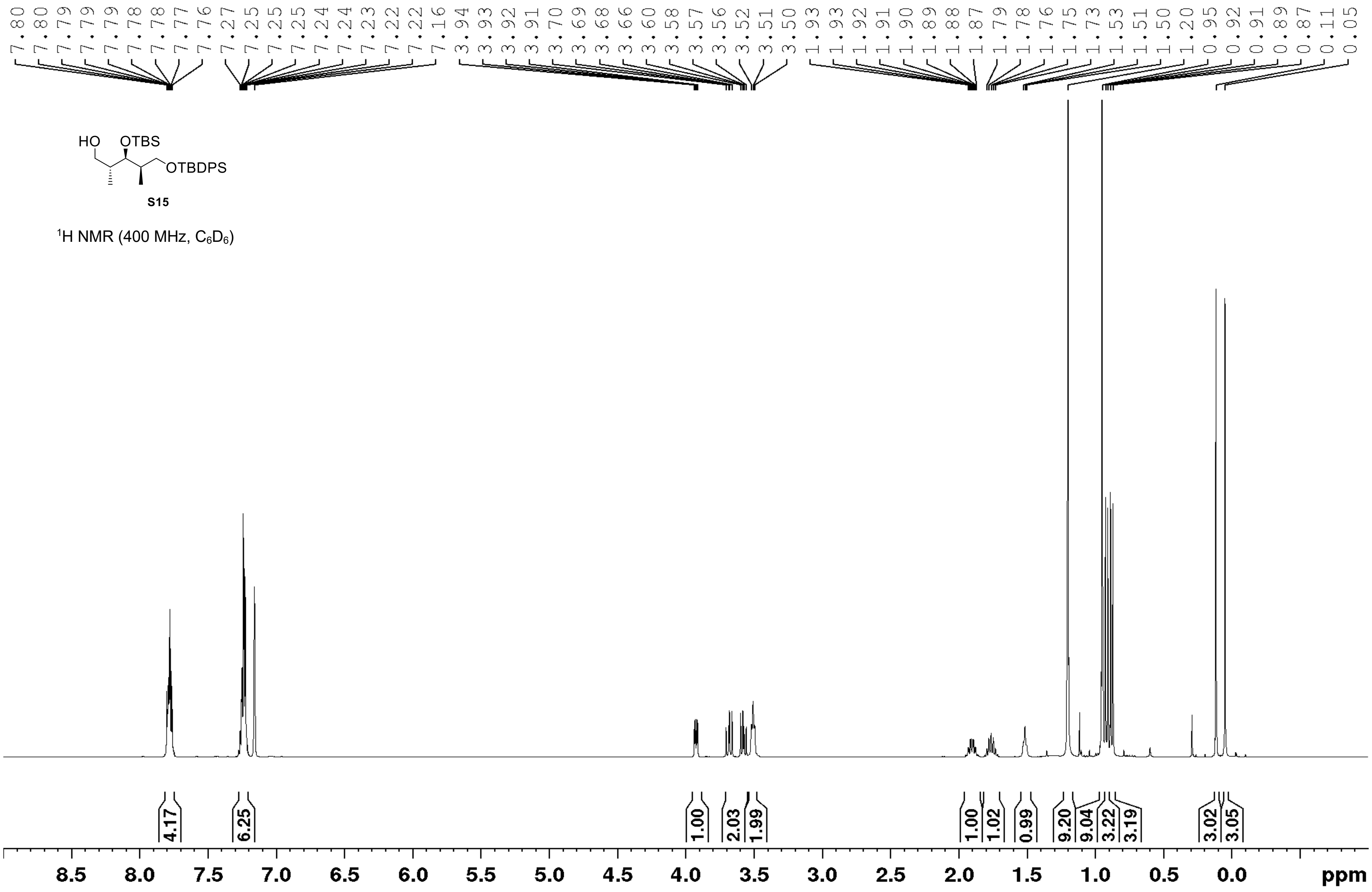
7.80 7.80 7.79 7.79 7.79 7.78 7.78 7.77 7.76 7.27 7.25 7.25 7.25 7.24 7.24 7.23 7.22 7.22 7.16 3.94 3.93 3.92 3.91 3.70 3.69 3.68 3.66 3.60 3.58 3.57 3.56 3.52 3.51 3.50 1.93 1.93 1.92 1.91 1.90 1.89 1.88 1.87 1.79 1.78 1.76 1.75 1.73 1.53 1.51 1.50 1.20 0.95 0.92 0.91 0.89 0.87 0.11 0.05
HO
OTBS
OTBDPS
S15
^{1}H NMR (400 MHz, C_6D_6)
4.17
6.25
1.00
2.03
1.99
1.00
1.02
0.99
9.20
9.04
3.22
3.19
3.02
3.05
8.5 8.0 7.5 7.0 6.5 6.0 5.5 5.0 4.5 4.0 3.5 3.0 2.5 2.0 1.5 1.0 0.5 0.0
ppm

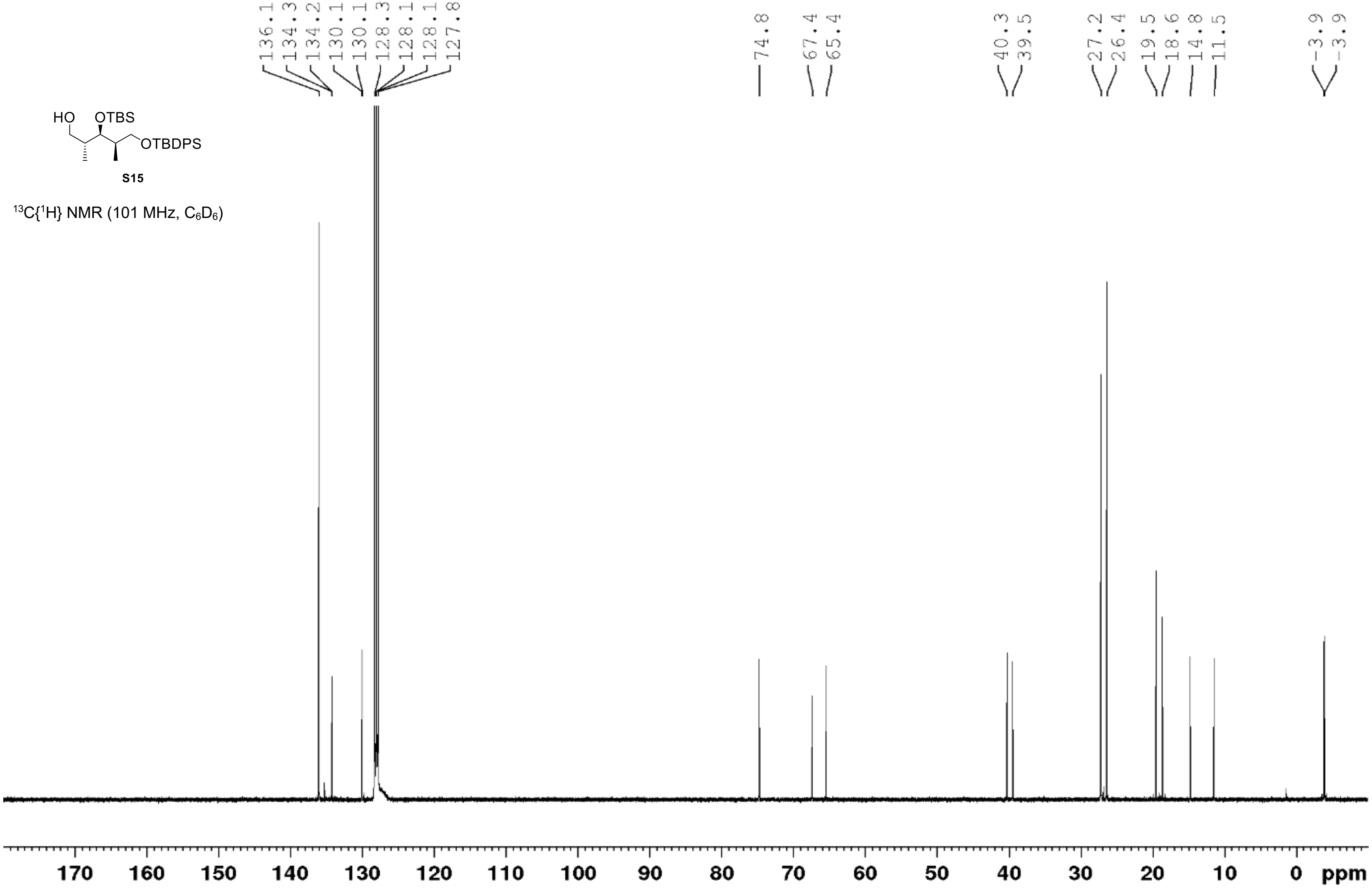
HO
OTBS
OTBDPS
S15
13C{1H} NMR (101 MHz, C6D6)
136.1
134.3
134.2
130.1
130.1
128.3
128.1
128.1
127.8
74.8
67.4
65.4
40.3
39.5
27.2
26.4
19.5
18.6
14.8
11.5
-3.9
-3.9
170
160
150
140
130
120
110
100
90
80
70
60
50
40
30
20
10
0
ppm

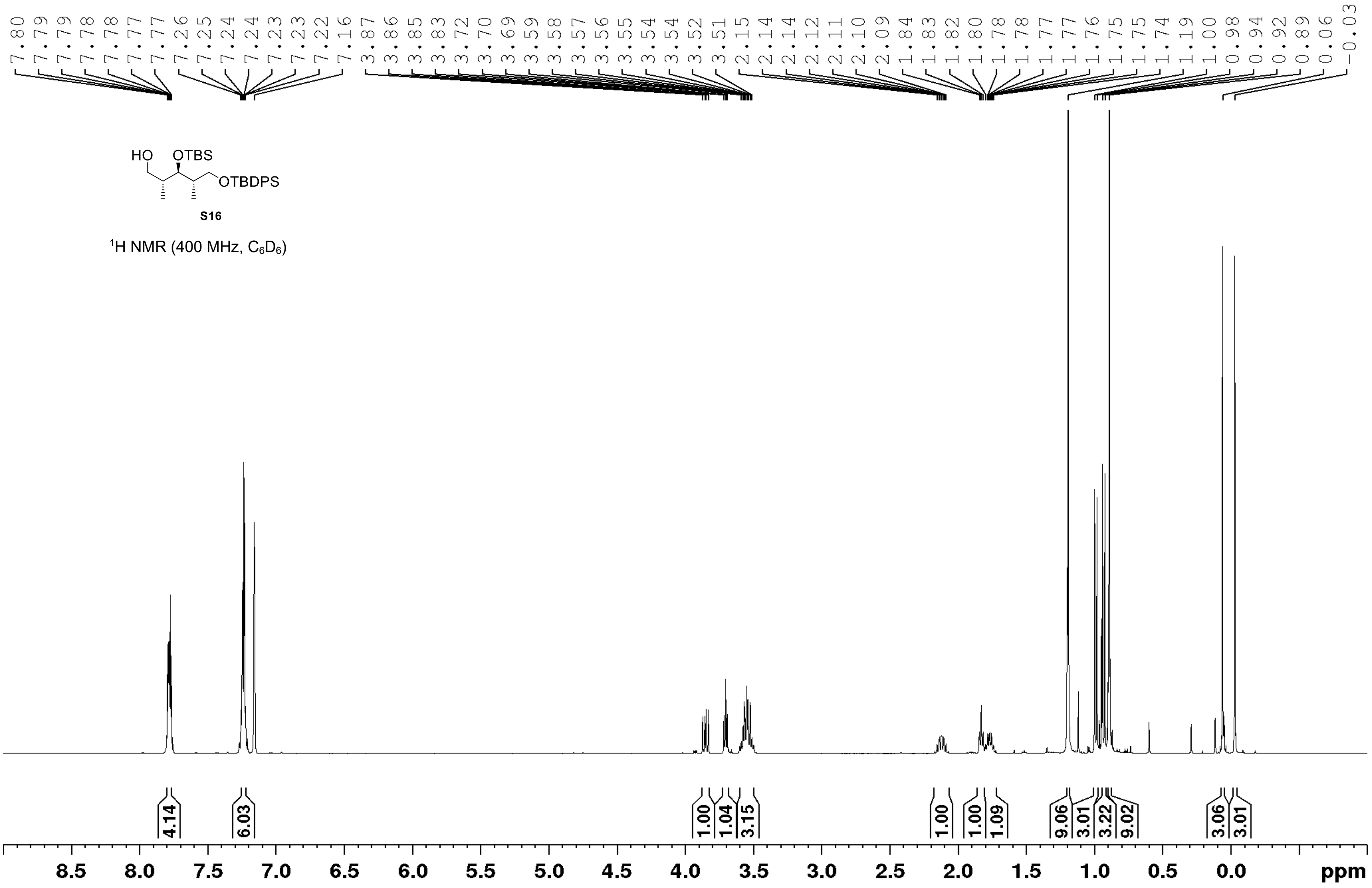
HO
OTBS
OTBDPS
S16
1H NMR (400 MHz, C6D6)
ppm

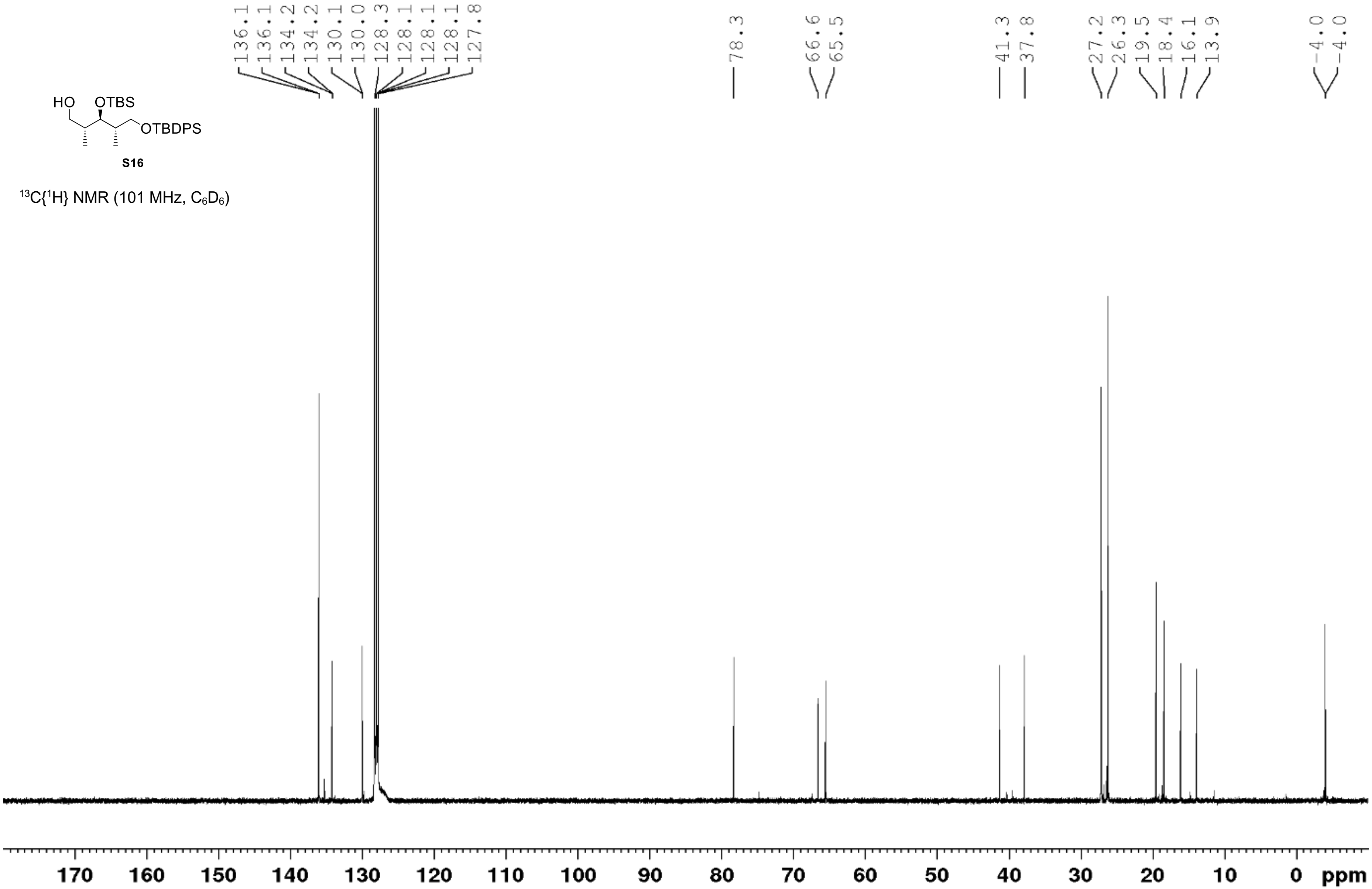

S16

$^{13}C\{^{1}H\}$ NMR (101 MHz, C_6D_6)

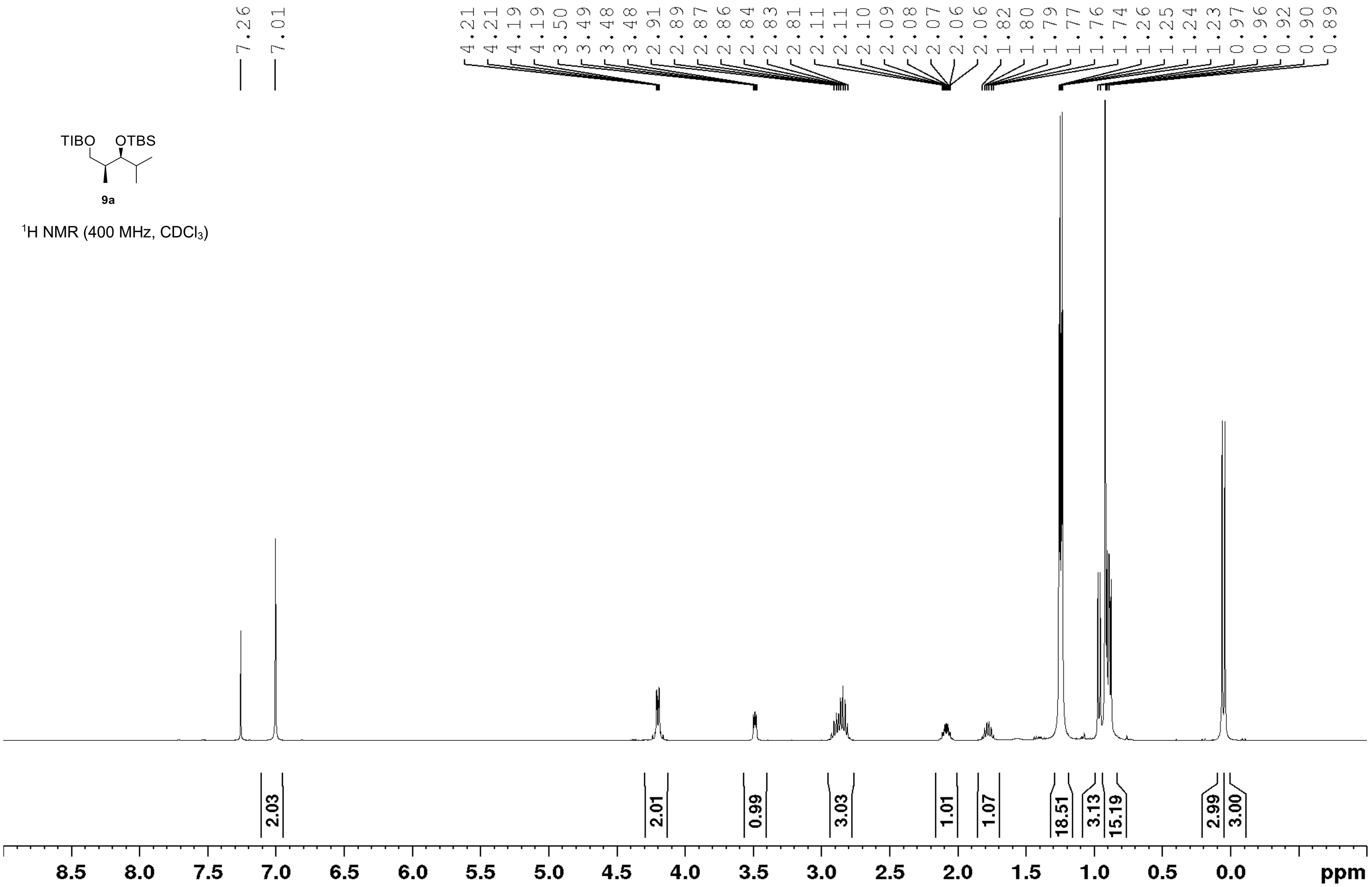
TIBO OTBS
9a
^{1}H NMR (400 MHz, $CDCl_3$)

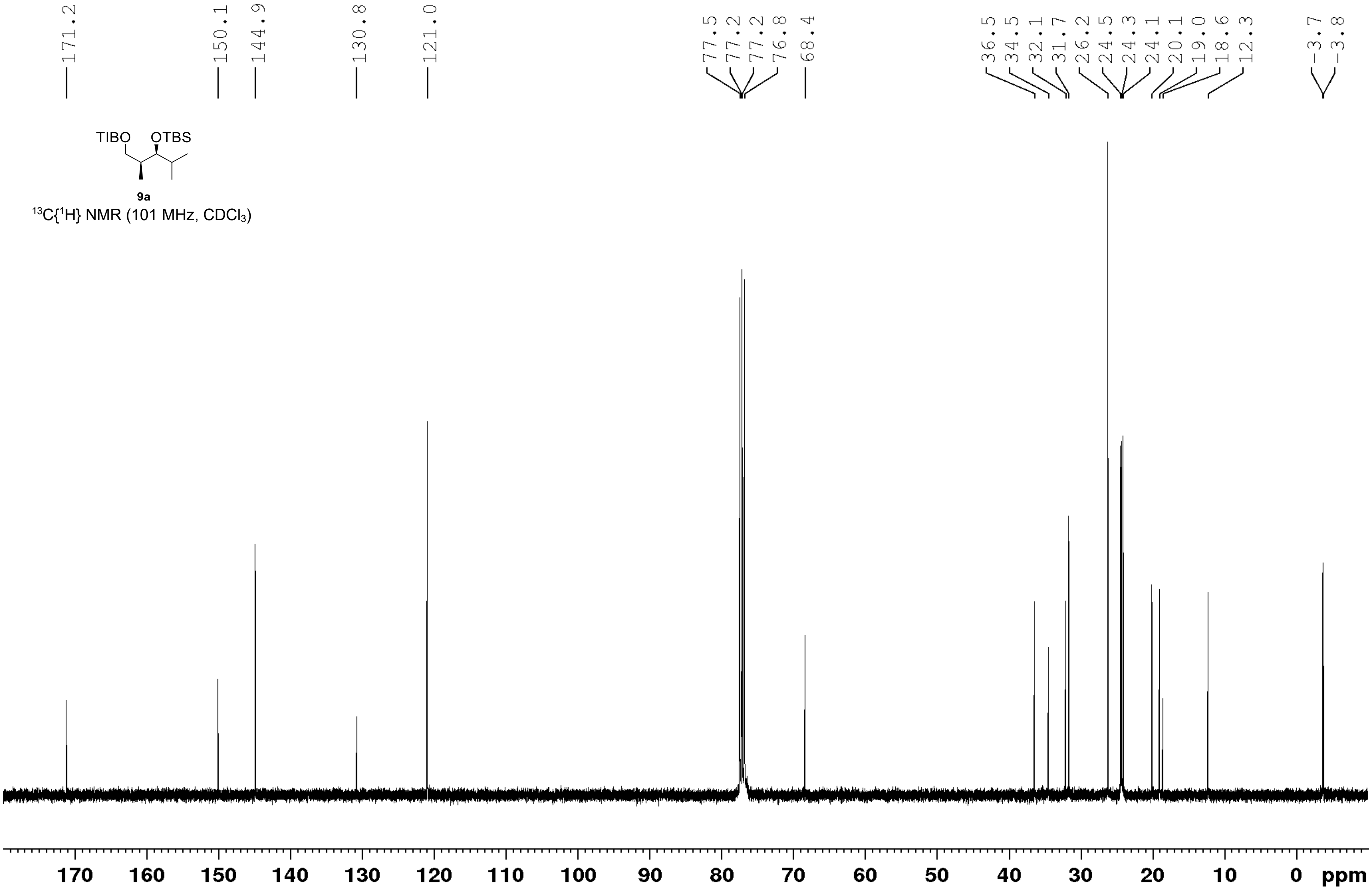
171.2
150.1
144.9
130.8
121.0
77.5
77.2
77.2
76.8
68.4
36.5
34.5
32.1
31.7
26.2
24.5
24.3
24.1
20.1
19.0
18.6
12.3
-3.7
-3.8
TIBO
OTBS
9a
$^{13}C\{^{1}H\}$ NMR (101 MHz, $CDCl_3$)
170 160 150 140 130 120 110 100 90 80 70 60 50 40 30 20 10 0 ppm

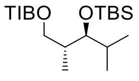

10a

^{1}H NMR (400 MHz, $CDCl_3$)

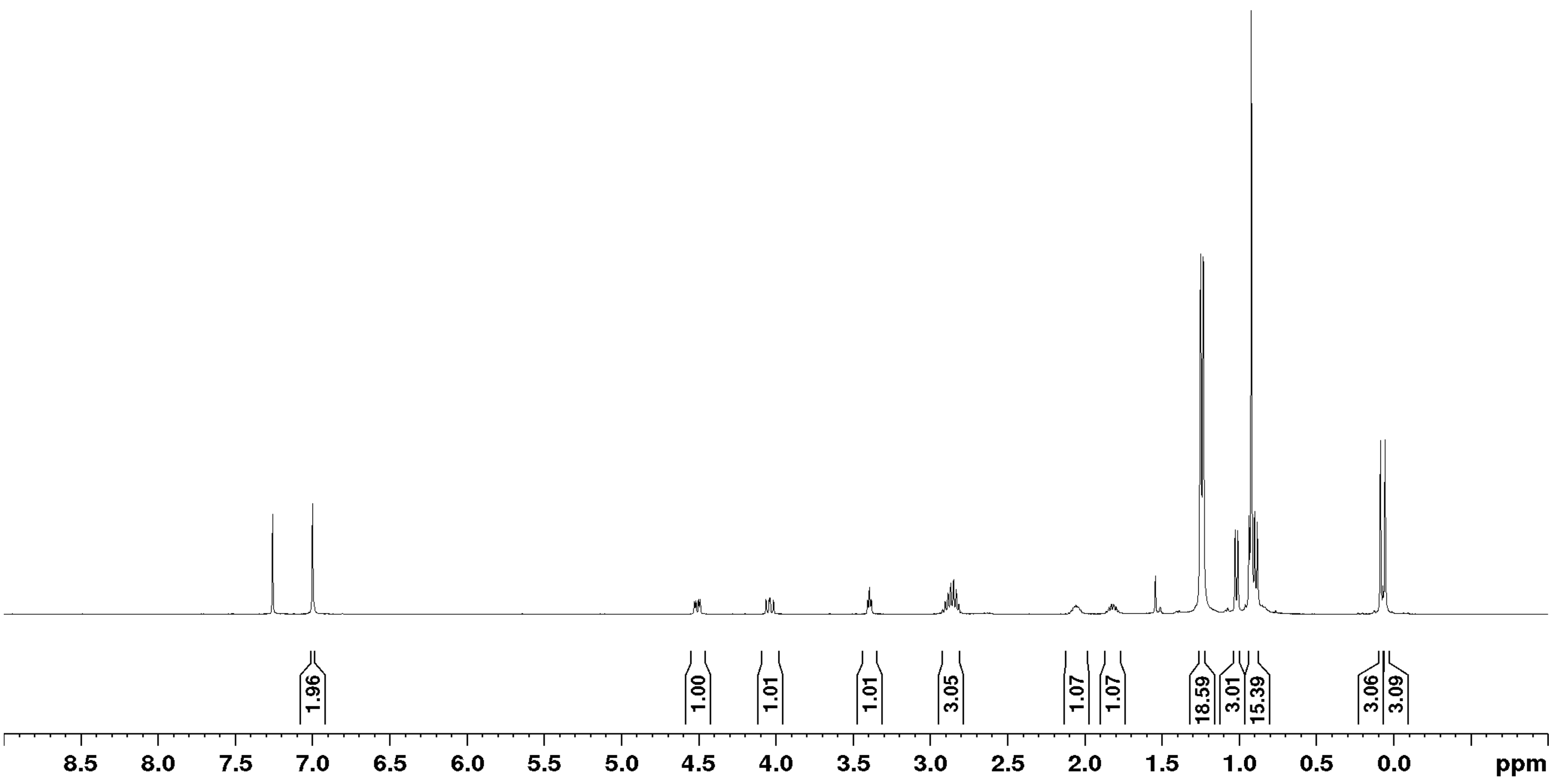

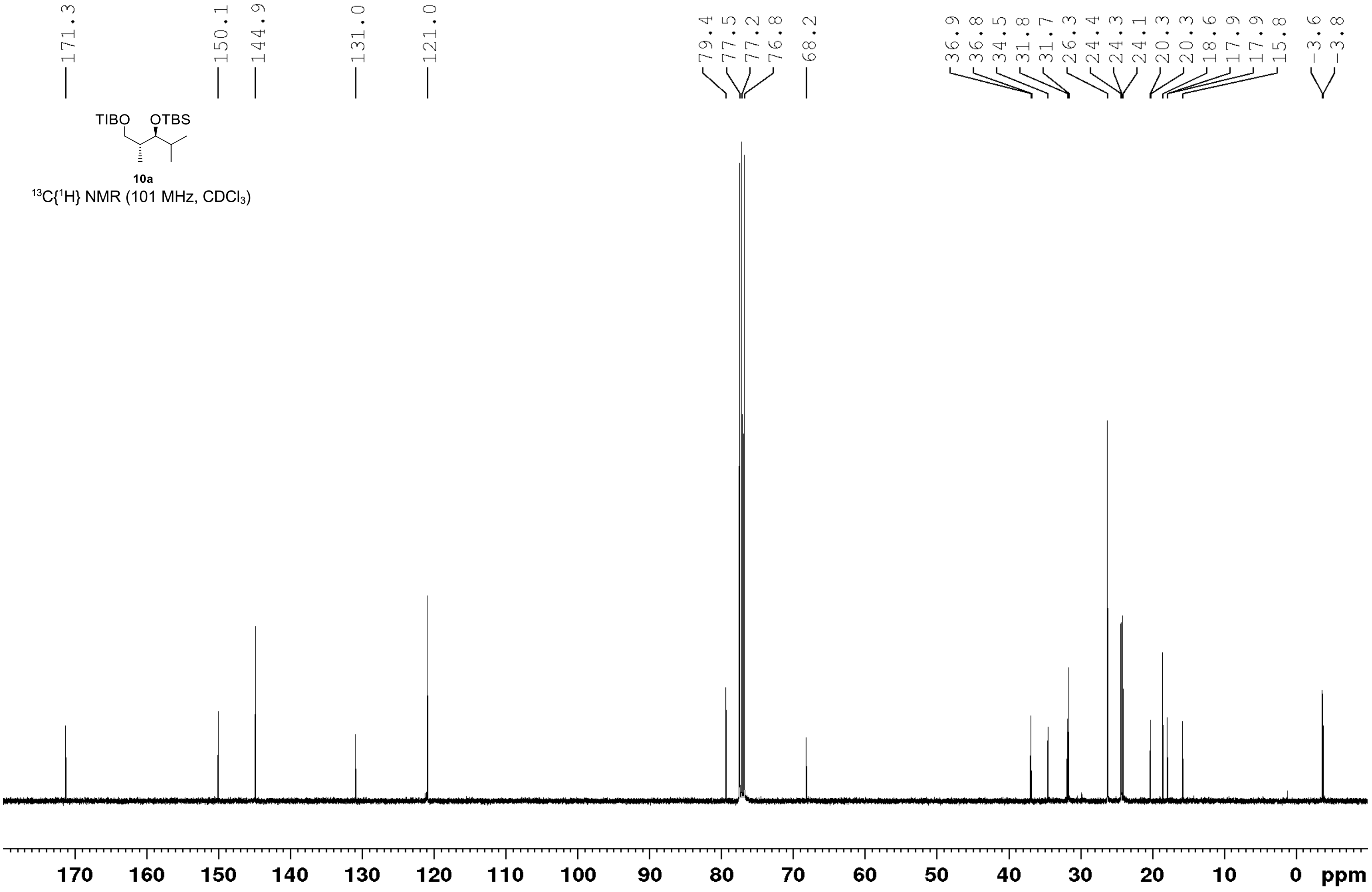

$^{13}C\{^{1}H\}$ NMR (101 MHz, $CDCl_3$)

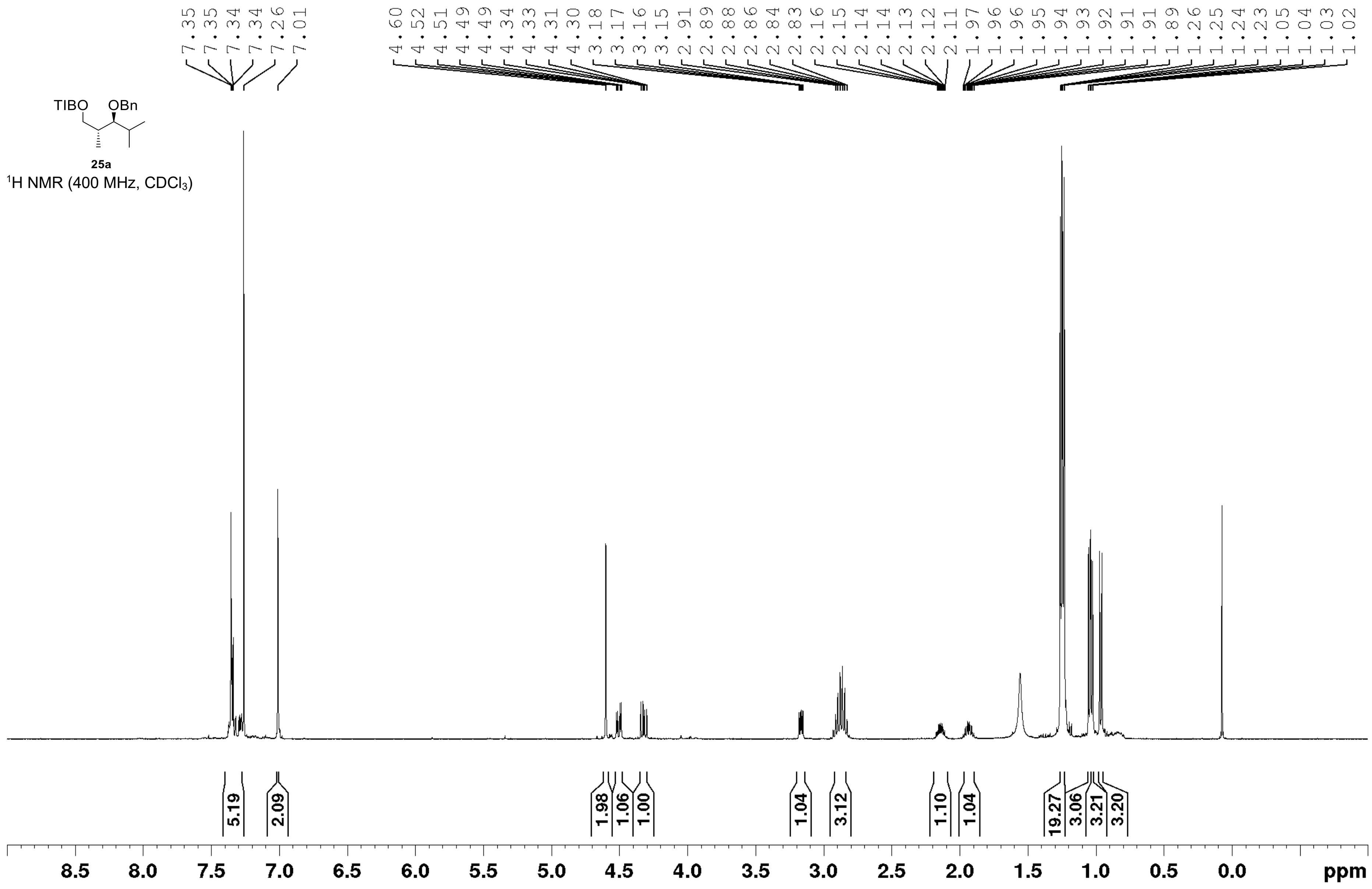

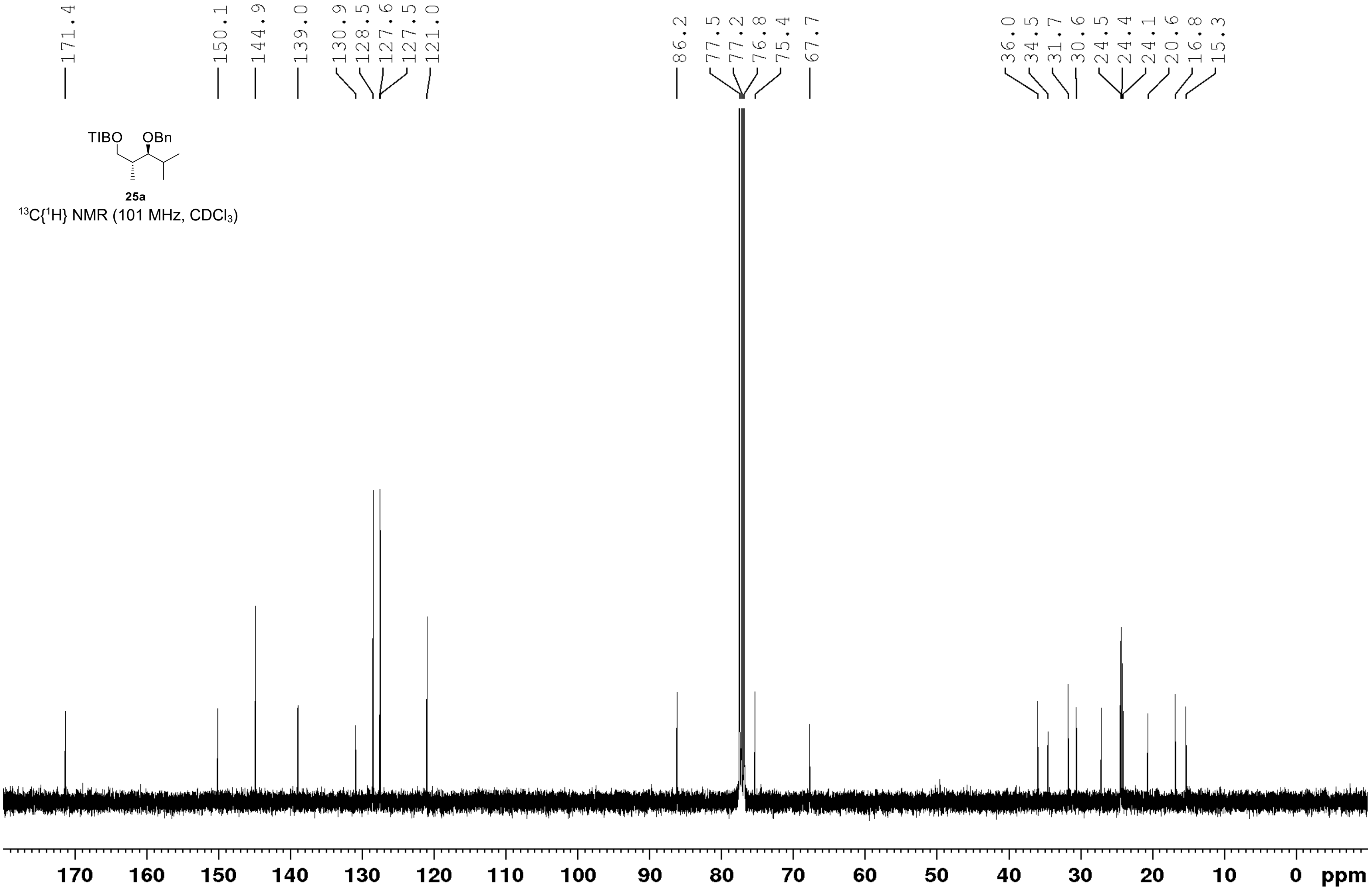
171.4
150.1
144.9
139.0
130.9
128.5
127.6
127.5
121.0
86.2
77.5
77.2
76.8
75.4
67.7
36.0
34.5
31.7
30.6
24.5
24.4
24.1
20.6
16.8
15.3
TIBO
OBn
25a
13C{1H} NMR (101 MHz, CDCl3)
170 160 150 140 130 120 110 100 90 80 70 60 50 40 30 20 10 0 ppm

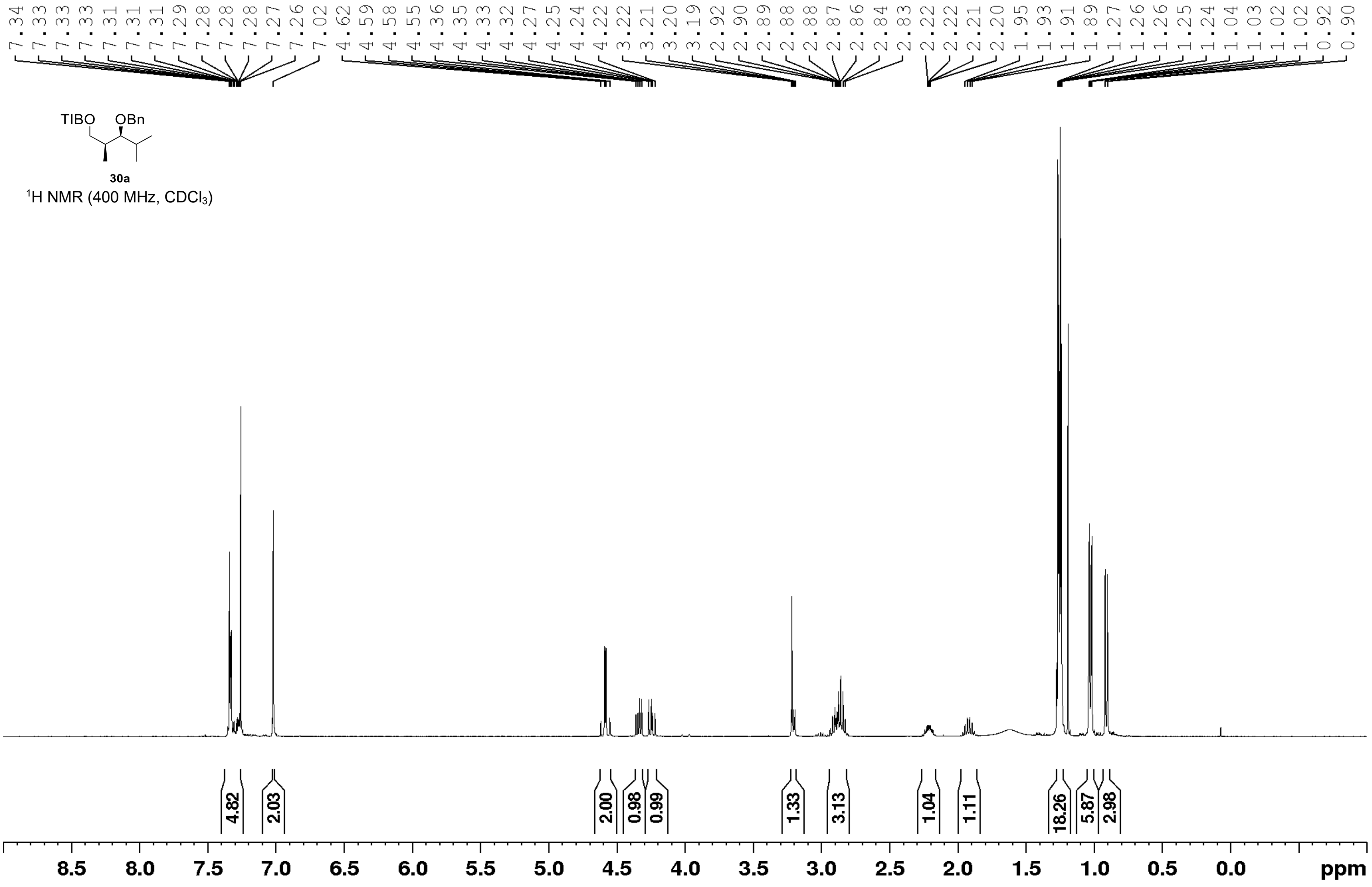
7.34 7.33 7.33 7.33 7.31 7.31 7.31 7.29 7.28 7.28 7.28 7.27 7.26 7.02 4.62 4.59 4.58 4.55 4.36 4.35 4.33 4.32 4.27 4.25 4.24 4.22 3.22 3.21 3.20 3.19 2.92 2.90 2.89 2.88 2.88 2.87 2.86 2.84 2.83 2.22 2.22 2.21 2.20 1.95 1.93 1.91 1.89 1.27 1.26 1.26 1.25 1.24 1.04 1.03 1.02 1.02 0.92 0.90
TIBO
OBn
30a
^{1}H NMR (400 MHz, CDCl$_3$)
4.82
2.03
2.00
0.98
0.99
1.33
3.13
1.04
1.11
18.26
5.87
2.98
8.5 8.0 7.5 7.0 6.5 6.0 5.5 5.0 4.5 4.0 3.5 3.0 2.5 2.0 1.5 1.0 0.5 0.0 ppm

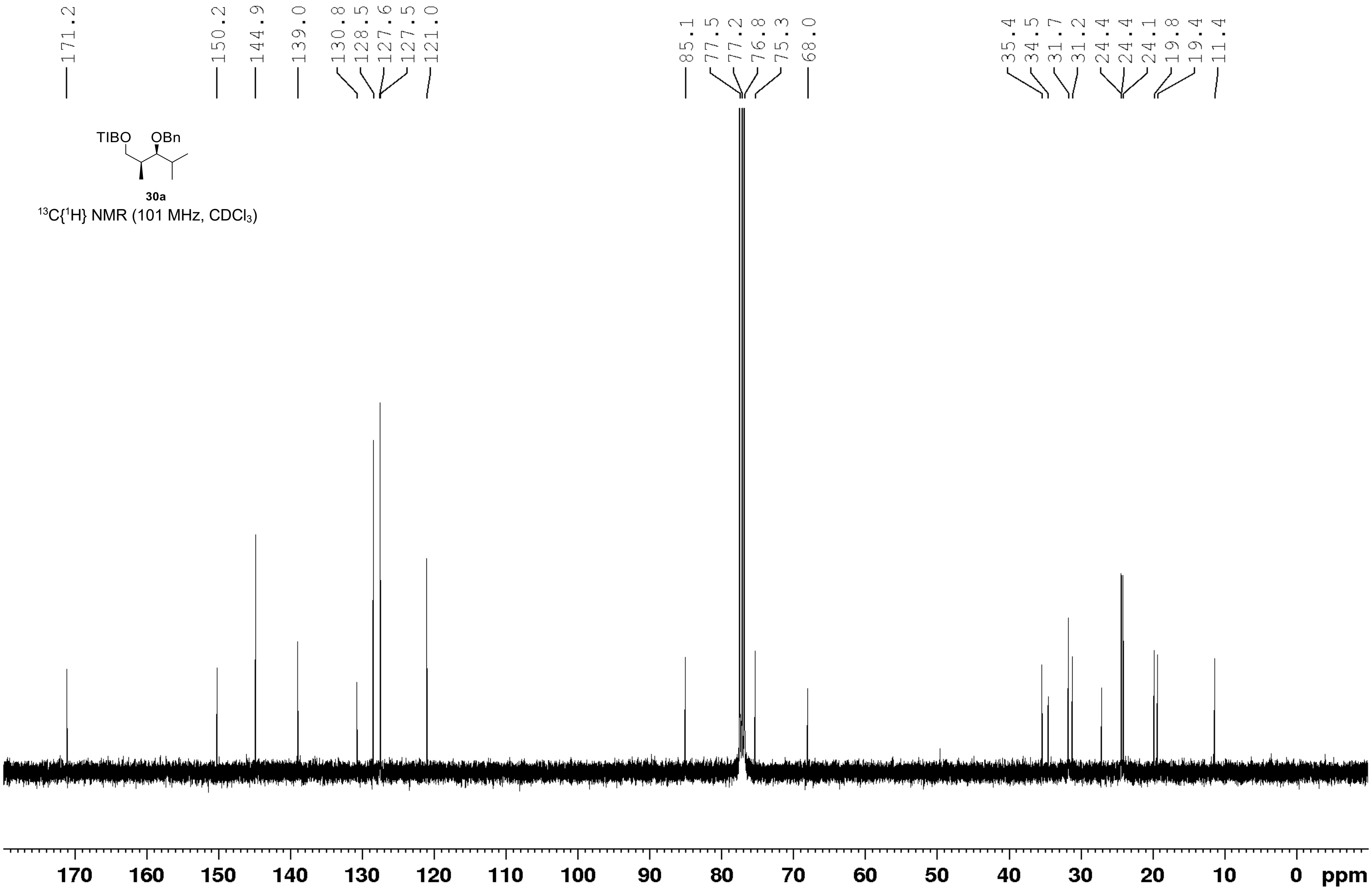
171.2
150.2
144.9
139.0
130.8
128.5
127.6
127.5
121.0
85.1
77.5
77.2
76.8
75.3
68.0
35.4
34.5
31.7
31.2
24.4
24.4
24.1
19.8
19.4
11.4
TIBO
OBn
30a
13C{1H} NMR (101 MHz, CDCl3)
170 160 150 140 130 120 110 100 90 80 70 60 50 40 30 20 10 0 ppm

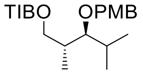

26a

^{1}H NMR (400 MHz, $CDCl_3$)

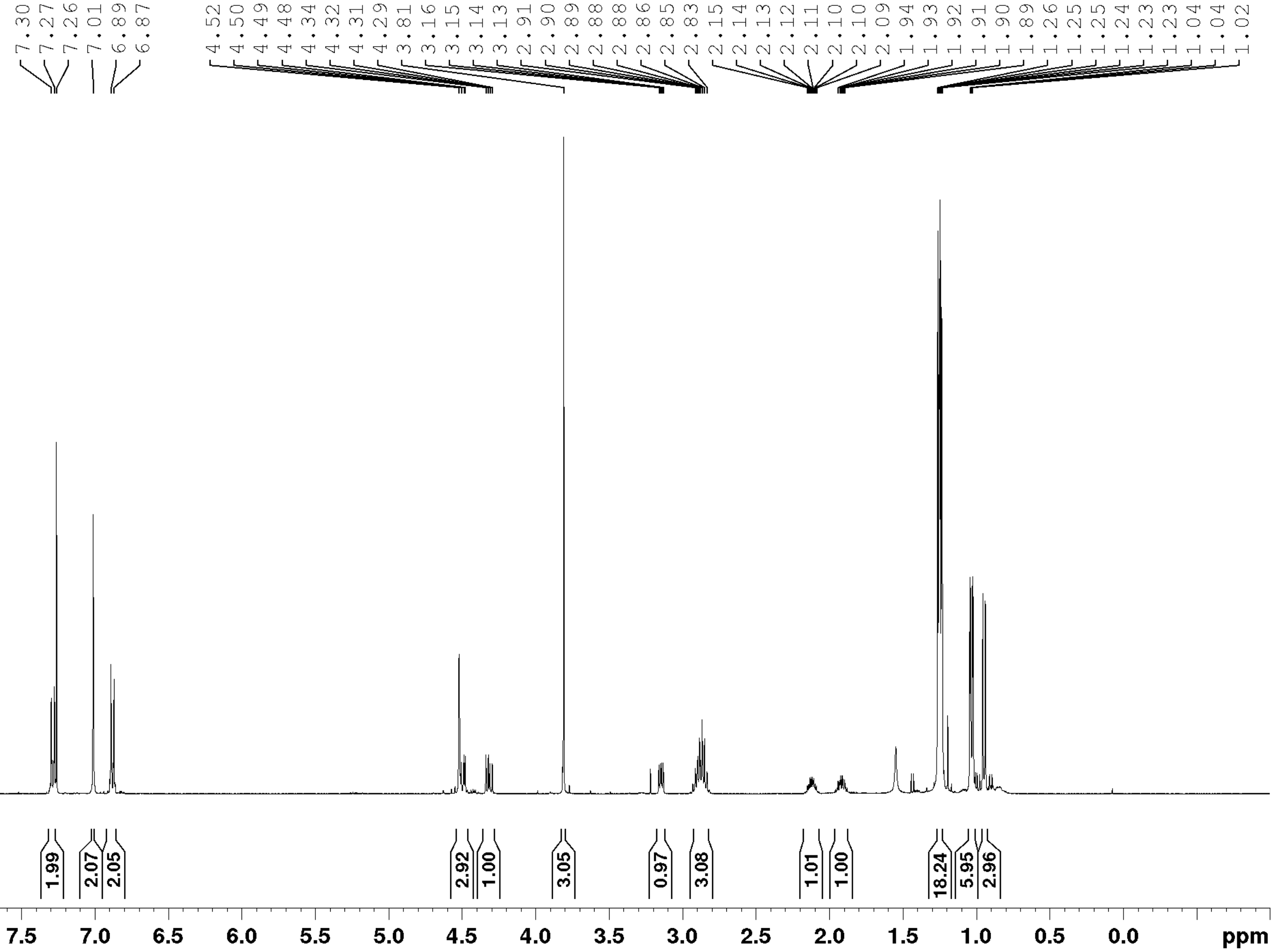

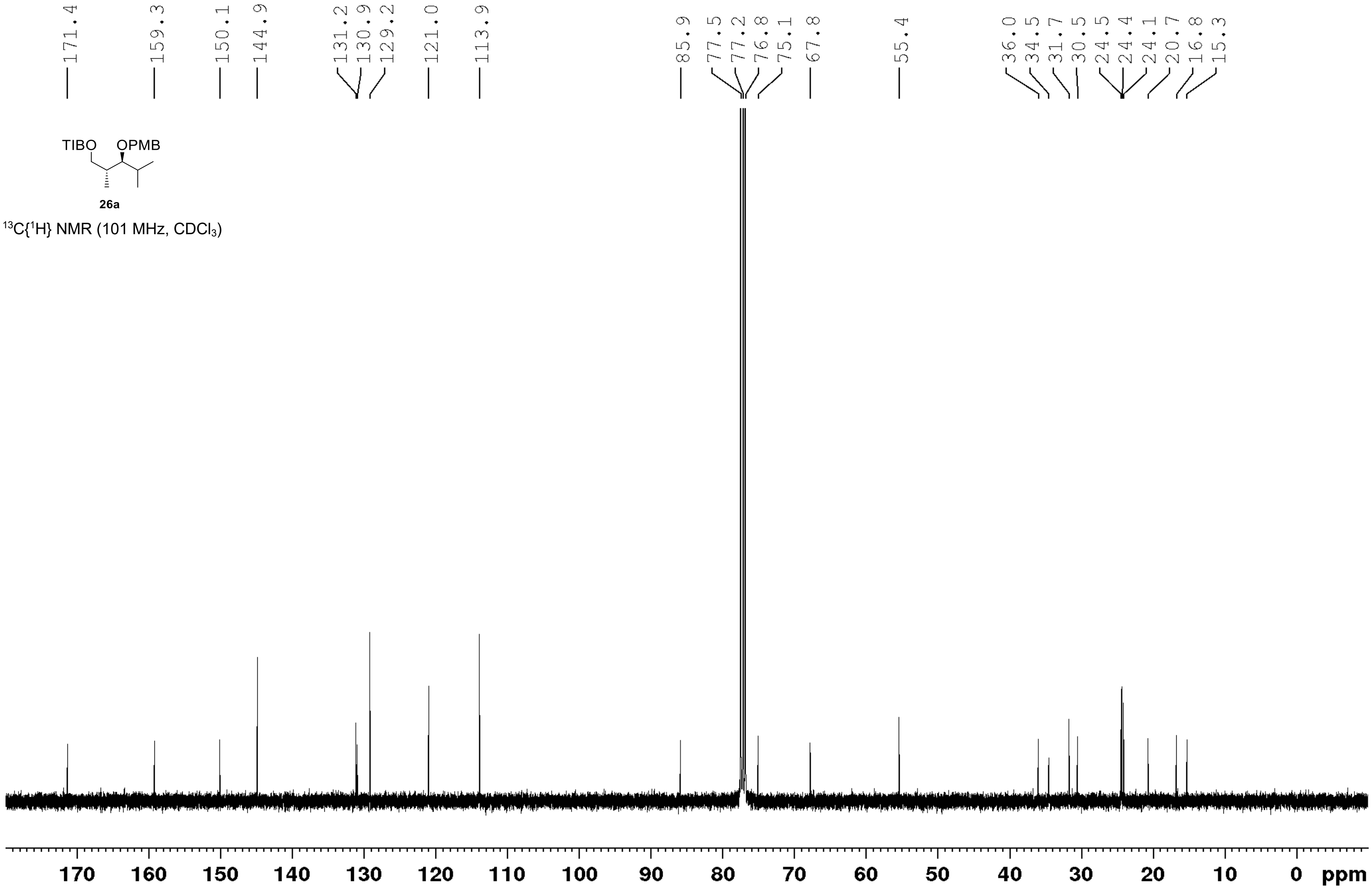
171.4
159.3
150.1
144.9
131.2
130.9
129.2
121.0
113.9
85.9
77.5
77.2
76.8
75.1
67.8
55.4
36.0
34.5
31.7
30.5
24.5
24.4
24.1
20.7
16.8
15.3
TIBO
OPMB
26a
$^{13}C\{^{1}H\}$ NMR (101 MHz, $CDCl_3$)
170
160
150
140
130
120
110
100
90
80
70
60
50
40
30
20
10
0
ppm

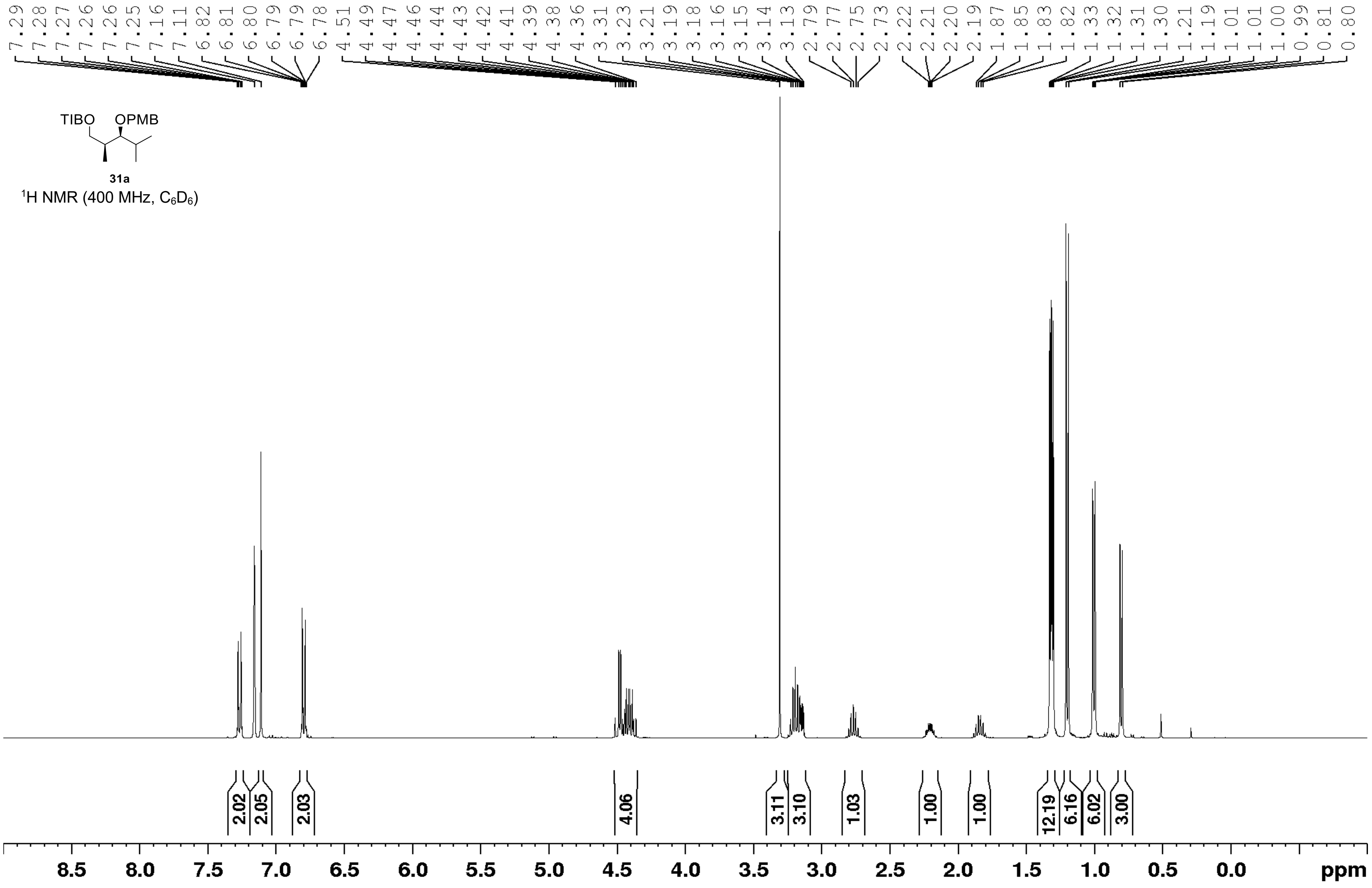

7.29 7.28 7.27 7.26 7.26 7.25 7.16 7.11 6.82 6.81 6.80 6.79 6.79 6.78 4.51 4.49 4.47 4.46 4.44 4.43 4.42 4.41 4.39 4.38 4.36 3.31 3.23 3.21 3.19 3.18 3.16 3.15 3.14 3.13 2.79 2.77 2.75 2.73 2.22 2.21 2.20 2.19 1.87 1.85 1.83 1.82 1.33 1.32 1.31 1.30 1.21 1.19 1.01 1.01 1.00 0.99 0.81 0.80
TIBO
OPMB
31a
¹H NMR (400 MHz, C6D6)
2.02 2.05 2.03 4.06 3.11 3.10 1.03 1.00 1.00 12.19 6.16 6.02 3.00
8.5 8.0 7.5 7.0 6.5 6.0 5.5 5.0 4.5 4.0 3.5 3.0 2.5 2.0 1.5 1.0 0.5 0.0 ppm

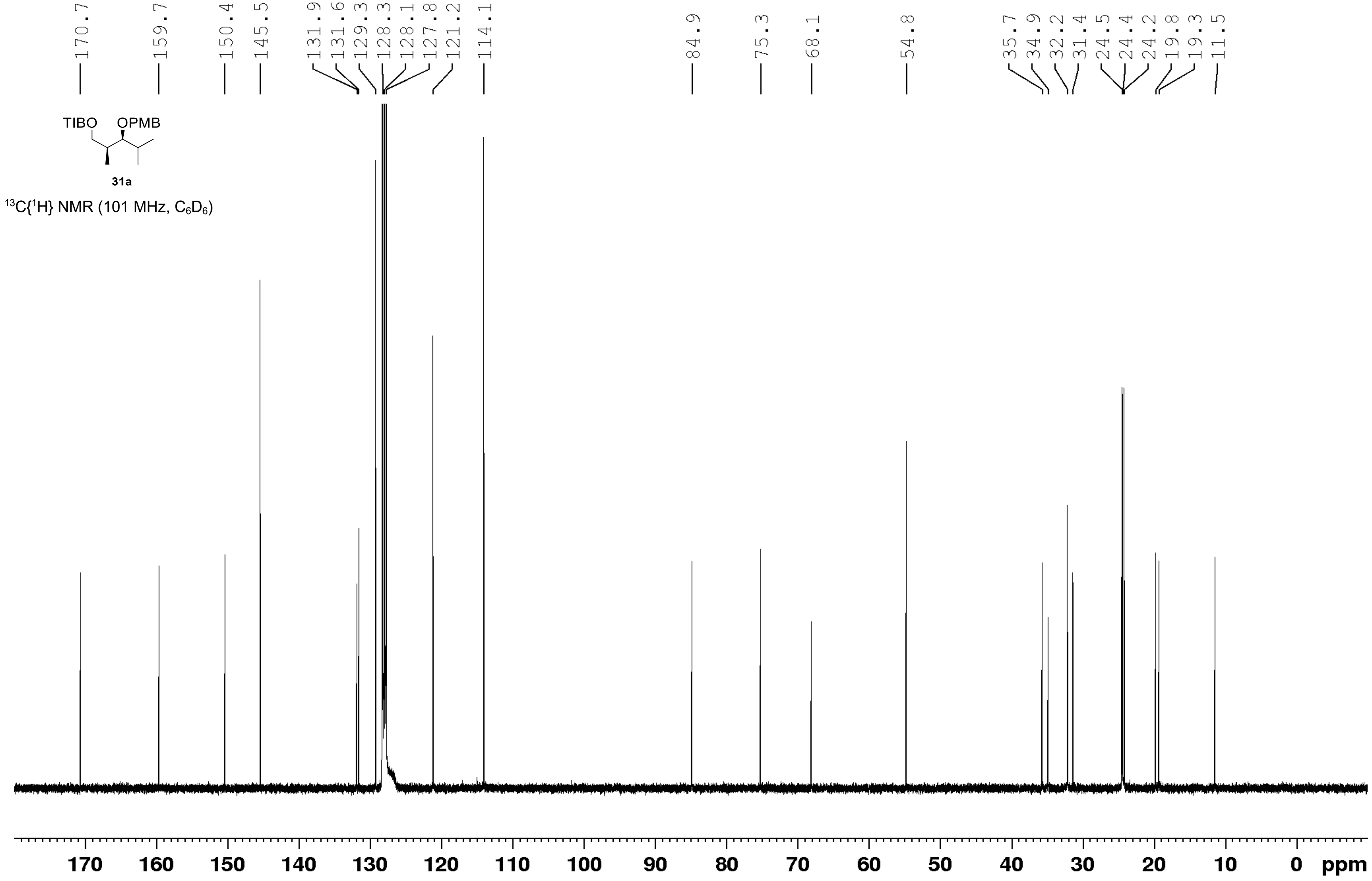

170.7
159.7
150.4
145.5
131.9
131.6
129.3
128.3
128.1
127.8
121.2
114.1
84.9
75.3
68.1
54.8
35.7
34.9
32.2
31.4
24.5
24.4
24.2
19.8
19.3
11.5
TIBO
OPMB
31a
$^{13}C\{^1H\}$ NMR (101 MHz, C_6D_6)
170
160
150
140
130
120
110
100
90
80
70
60
50
40
30
20
10
0
ppm

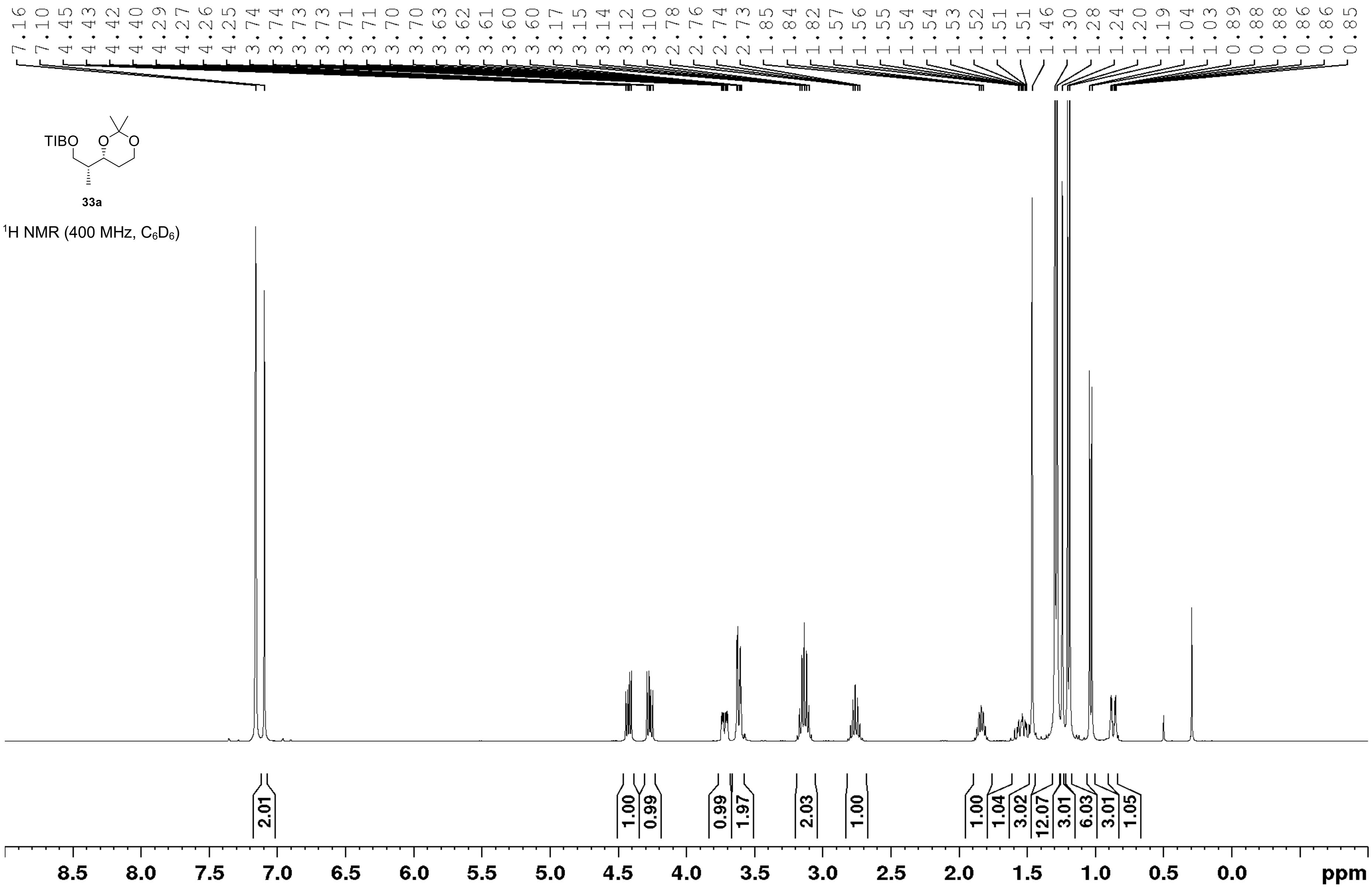
7.16 7.10 4.45 4.43 4.42 4.40 4.29 4.27 4.26 4.25 3.74 3.74 3.73 3.73 3.71 3.71 3.70 3.70 3.63 3.62 3.61 3.60 3.60 3.17 3.15 3.14 3.12 3.10 2.78 2.76 2.74 2.73 1.85 1.84 1.82 1.57 1.56 1.55 1.54 1.54 1.53 1.52 1.51 1.51 1.46 1.30 1.28 1.24 1.20 1.19 1.04 1.03 0.89 0.88 0.86 0.86 0.85
TIBO
O
O
33a
1H NMR (400 MHz, C6D6)
2.01
1.00
0.99
0.99
1.97
2.03
1.00
1.00
1.04
3.02
12.07
3.01
6.03
3.01
1.05
8.5 8.0 7.5 7.0 6.5 6.0 5.5 5.0 4.5 4.0 3.5 3.0 2.5 2.0 1.5 1.0 0.5 0.0 ppm

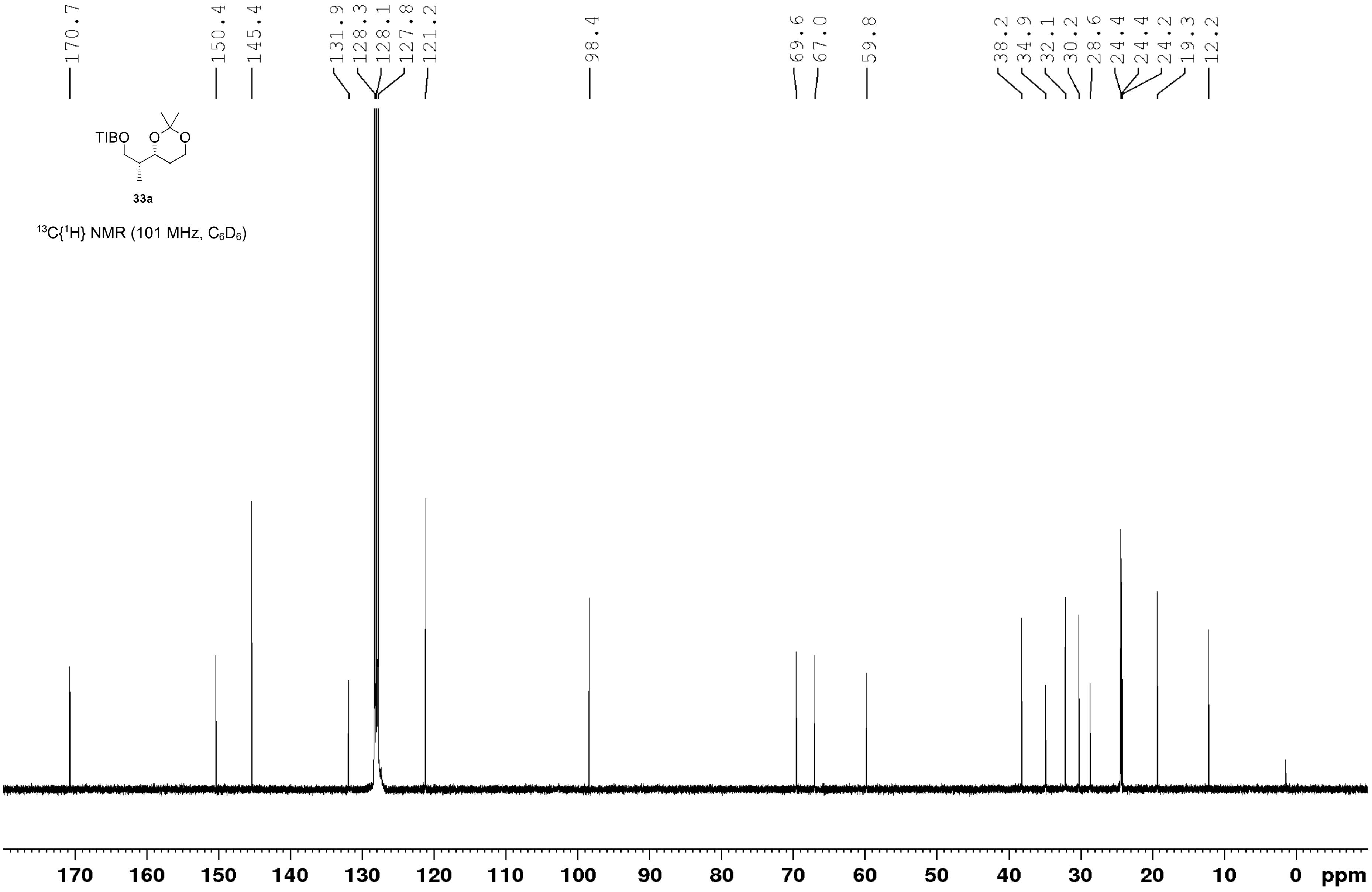

170.7
150.4
145.4
131.9
128.3
128.1
127.8
121.2
98.4
69.6
67.0
59.8
38.2
34.9
32.1
30.2
28.6
24.4
24.4
24.2
19.3
12.2
TIBO
O
O
33a
$^{13}C\{^1H\}$ NMR (101 MHz, C_6D_6)
170 160 150 140 130 120 110 100 90 80 70 60 50 40 30 20 10 0 ppm

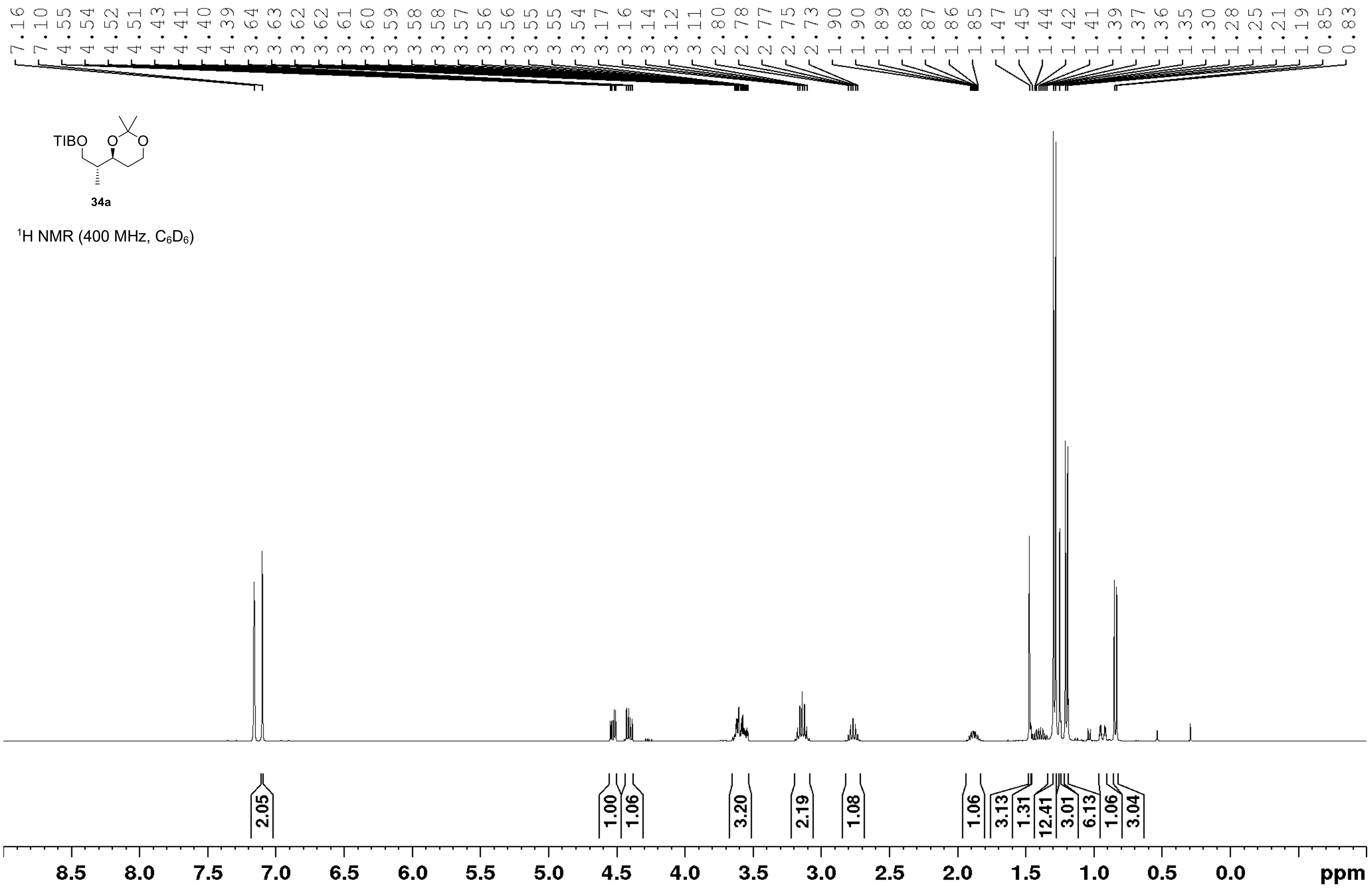

7.16 7.10 4.55 4.54 4.52 4.51 4.43 4.41 4.40 4.39 3.64 3.63 3.62 3.62 3.61 3.60 3.59 3.58 3.58 3.57 3.56 3.56 3.55 3.55 3.54 3.17 3.16 3.14 3.12 3.11 2.80 2.78 2.77 2.75 2.73 1.90 1.90 1.89 1.88 1.87 1.86 1.85 1.47 1.45 1.44 1.42 1.41 1.39 1.37 1.36 1.35 1.30 1.28 1.25 1.21 1.19 0.85 0.83
TIBO
O
O
34a
1H NMR (400 MHz, C6D6)
2.05
1.00
1.06
3.20
2.19
1.08
1.06
3.13
1.31
12.41
3.01
6.13
1.06
3.04
8.5 8.0 7.5 7.0 6.5 6.0 5.5 5.0 4.5 4.0 3.5 3.0 2.5 2.0 1.5 1.0 0.5 0.0 ppm

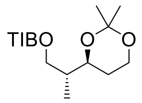

34a

$^{13}C\{^{1}H\}$ NMR (101 MHz, C_6D_6)

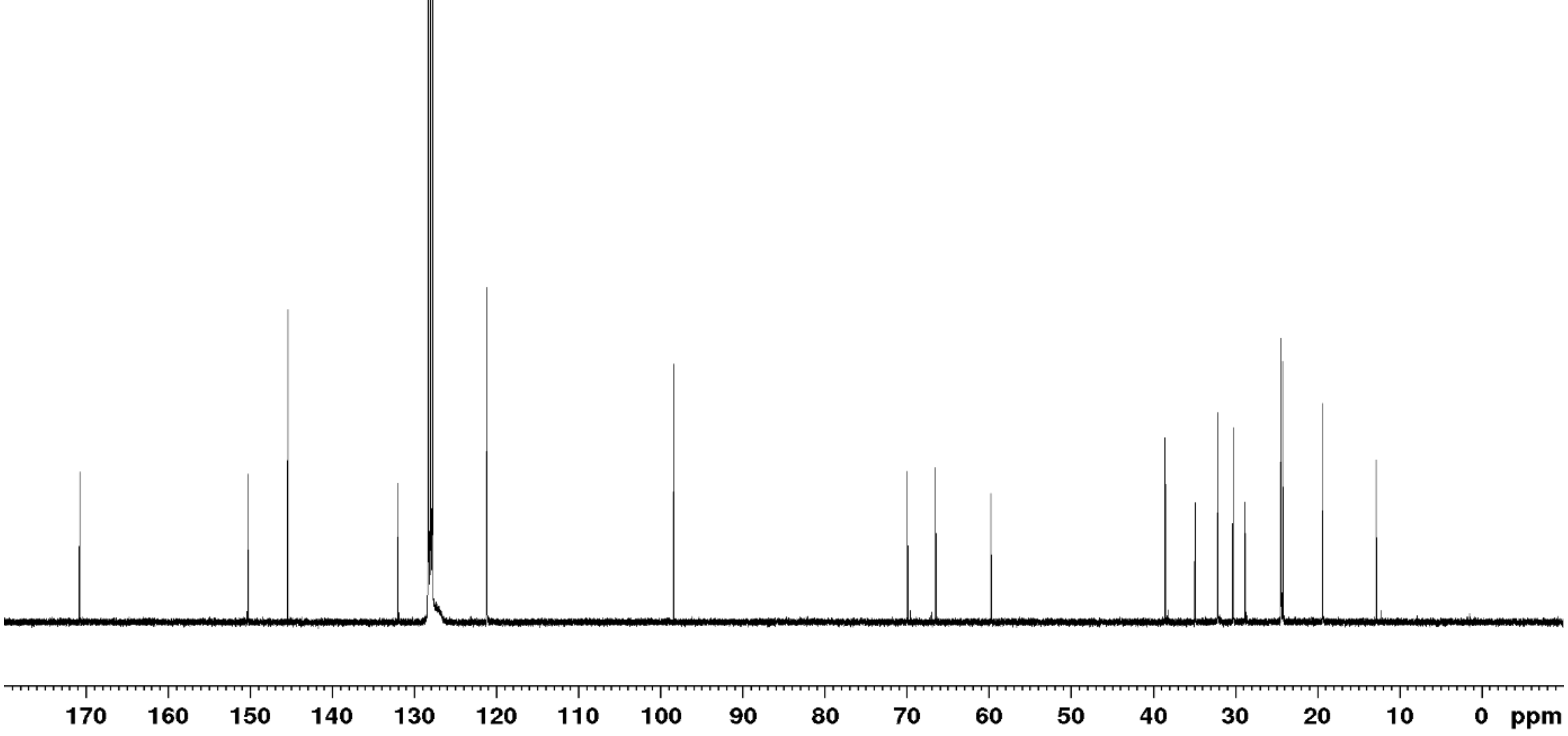

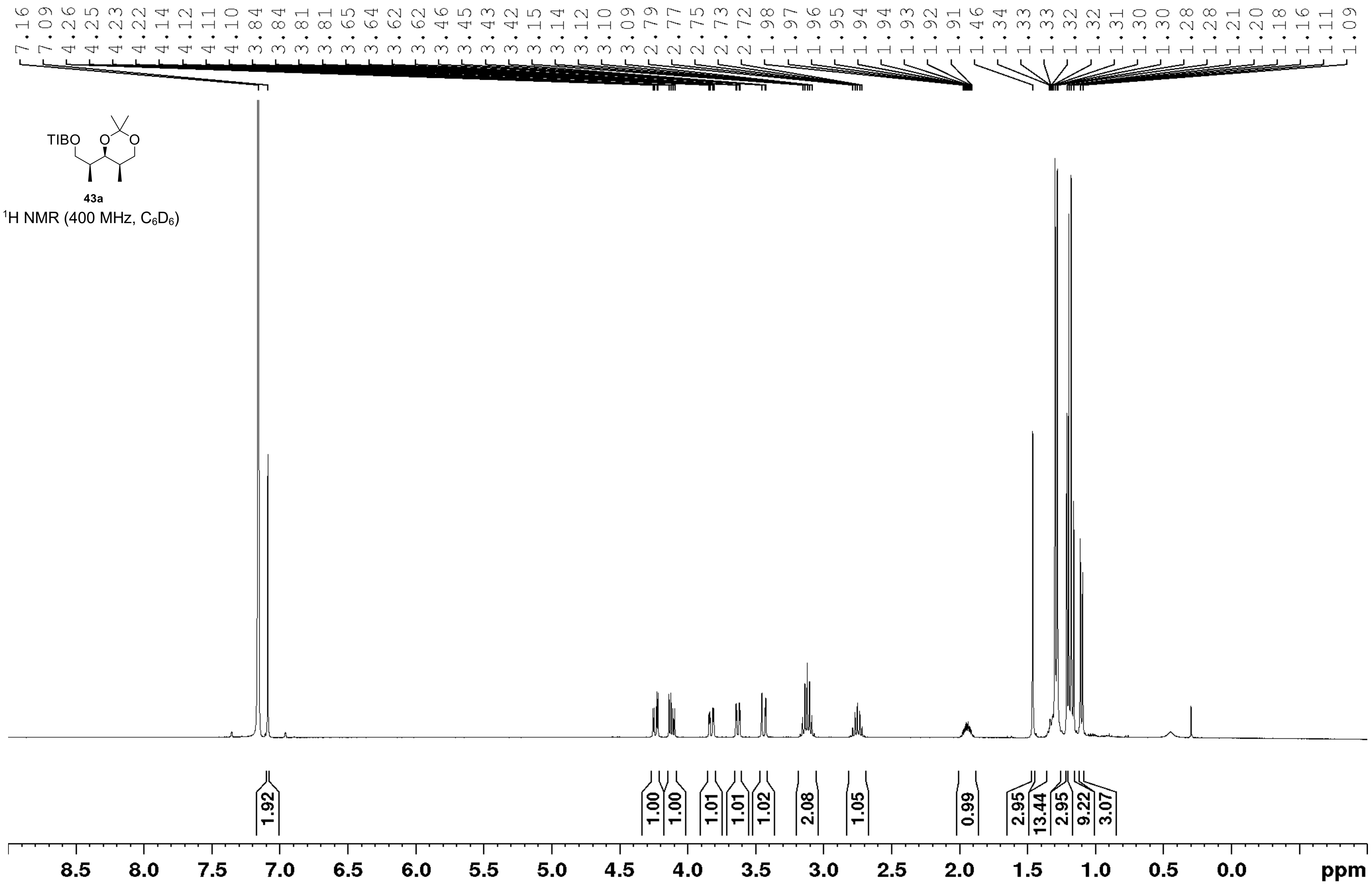
7.16 7.09 4.26 4.25 4.23 4.22 4.14 4.12 4.11 4.10 3.84 3.84 3.81 3.81 3.65 3.64 3.62 3.62 3.46 3.45 3.43 3.42 3.15 3.14 3.12 3.10 3.09 2.79 2.77 2.75 2.73 2.72 1.98 1.97 1.96 1.95 1.94 1.94 1.93 1.92 1.91 1.46 1.34 1.33 1.33 1.32 1.32 1.31 1.30 1.30 1.28 1.28 1.21 1.20 1.18 1.16 1.11 1.09
TIBO
O
O
43a
^{1}H NMR (400 MHz, C_6D_6)
1.92
1.00
1.00
1.01
1.01
1.02
2.08
1.05
0.99
2.95
13.44
2.95
9.22
3.07
8.5 8.0 7.5 7.0 6.5 6.0 5.5 5.0 4.5 4.0 3.5 3.0 2.5 2.0 1.5 1.0 0.5 0.0 ppm

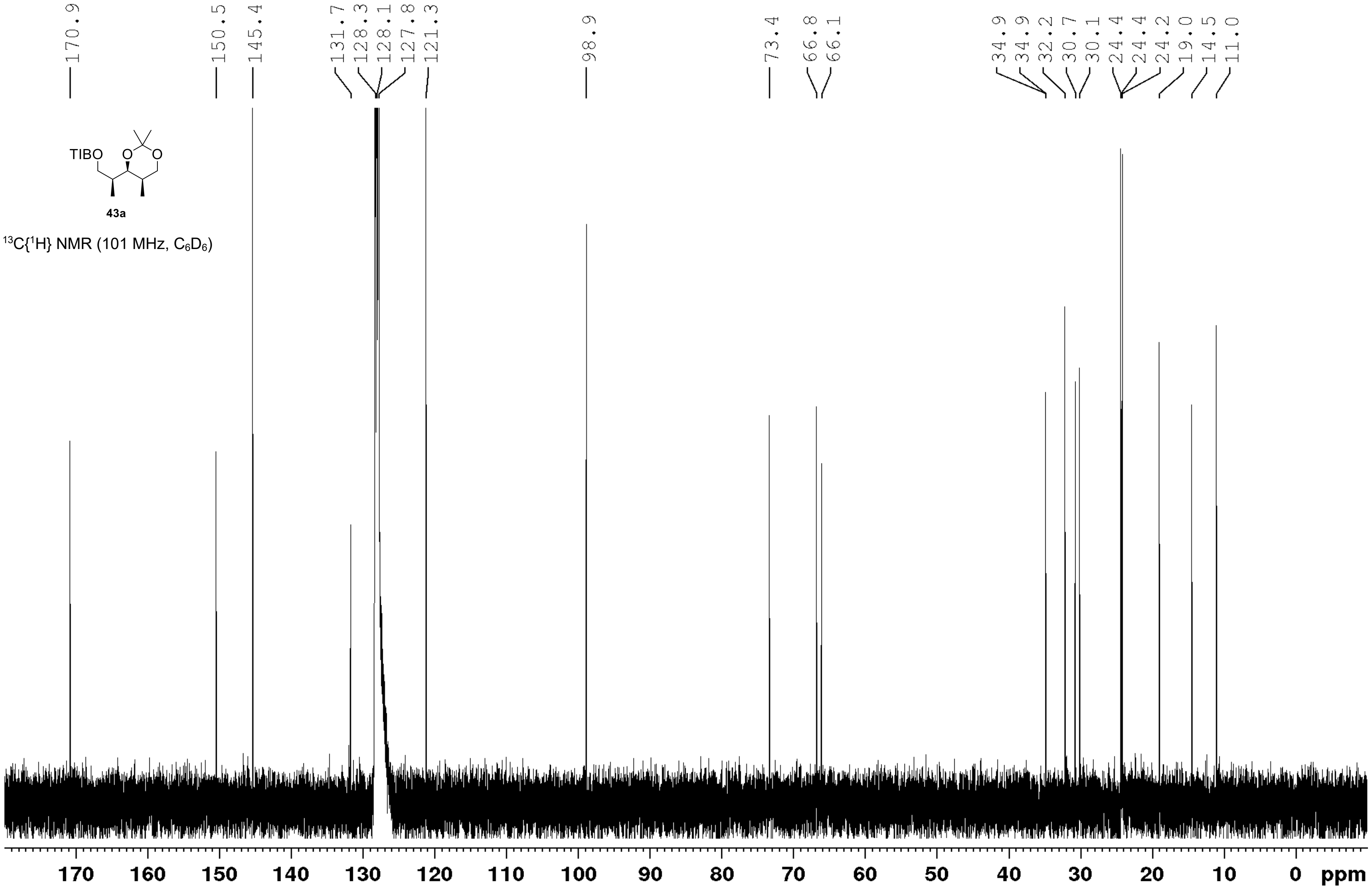
170.9
150.5
145.4
131.7
128.3
128.1
127.8
121.3
98.9
73.4
66.8
66.1
34.9
34.9
32.2
30.7
30.1
24.4
24.4
24.2
19.0
14.5
11.0
TIBO
O
O
43a
$^{13}C\{^{1}H\}$ NMR (101 MHz, C_6D_6)
170
160
150
140
130
120
110
100
90
80
70
60
50
40
30
20
10
0
ppm

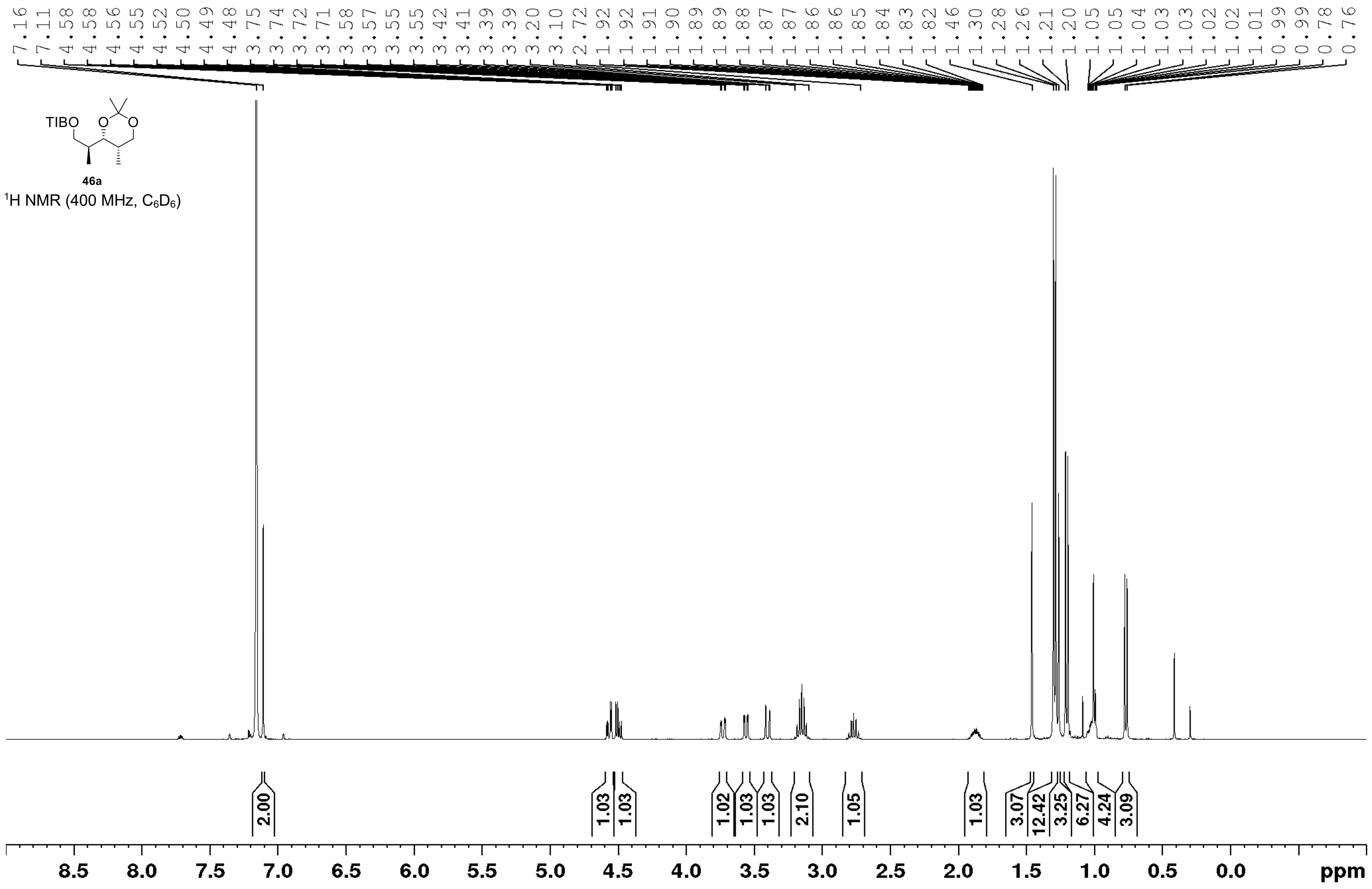
7.16 7.11 4.58 4.58 4.56 4.55 4.52 4.50 4.49 4.48 3.75 3.74 3.72 3.71 3.58 3.57 3.55 3.55 3.42 3.41 3.39 3.39 3.20 3.10 2.72 1.92 1.92 1.91 1.90 1.89 1.89 1.88 1.87 1.87 1.86 1.86 1.85 1.84 1.83 1.82 1.46 1.30 1.28 1.26 1.21 1.20 1.05 1.05 1.04 1.03 1.03 1.02 1.02 1.01 0.99 0.99 0.78 0.76
TIBO
O
O
46a
1H NMR (400 MHz, C6D6)
2.00
1.03
1.03
1.02
1.03
1.03
2.10
1.05
1.03
3.07
12.42
3.25
6.27
4.24
3.09
8.5 8.0 7.5 7.0 6.5 6.0 5.5 5.0 4.5 4.0 3.5 3.0 2.5 2.0 1.5 1.0 0.5 0.0
ppm

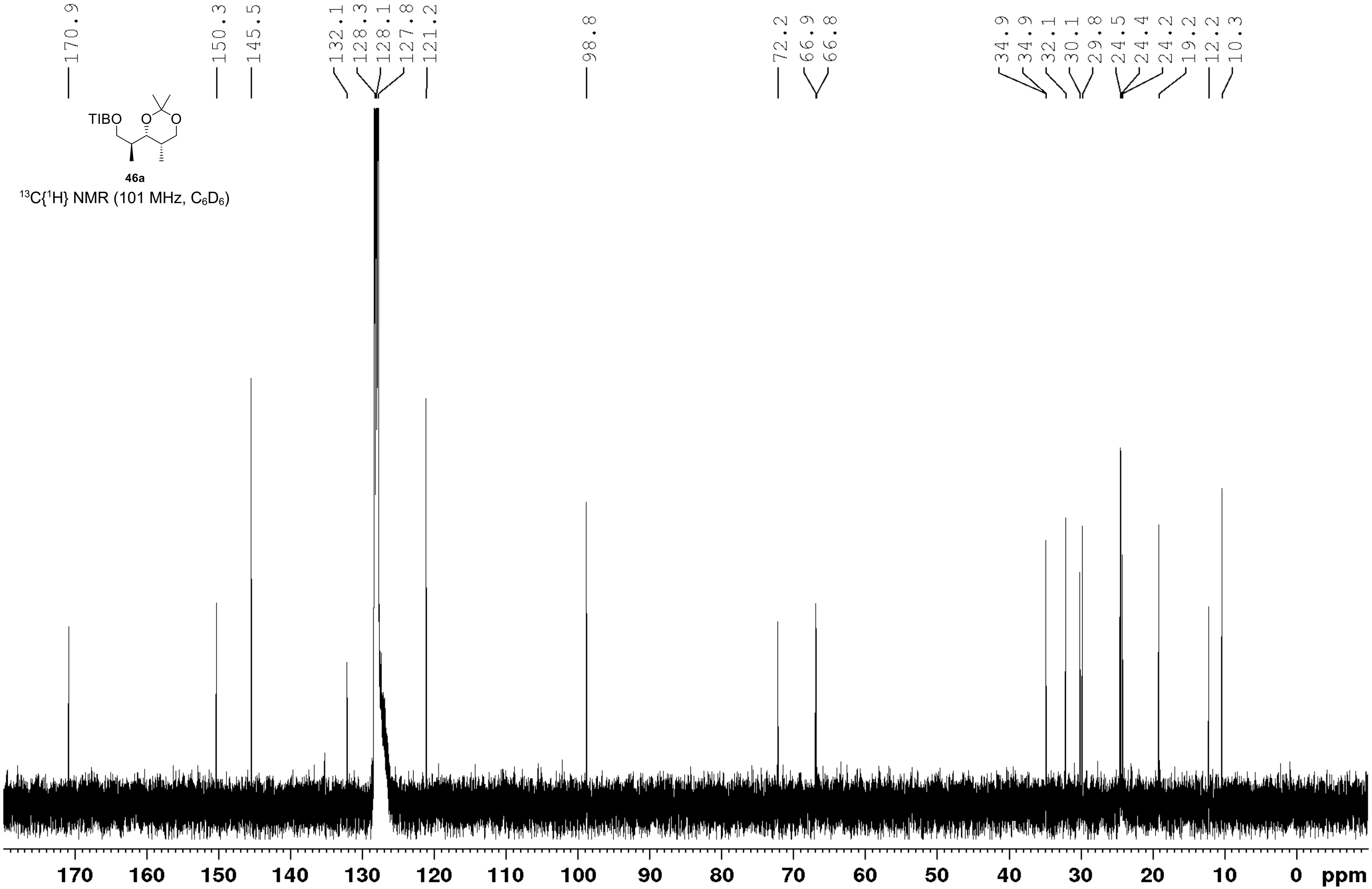
170.9
150.3
145.5
132.1
128.3
128.1
127.8
121.2
98.8
72.2
66.9
66.8
34.9
34.9
32.1
30.1
29.8
24.5
24.4
24.2
19.2
12.2
10.3
TIBO
O
O
46a
$^{13}C\{^1H\}$ NMR (101 MHz, C_6D_6)
170 160 150 140 130 120 110 100 90 80 70 60 50 40 30 20 10 0 ppm

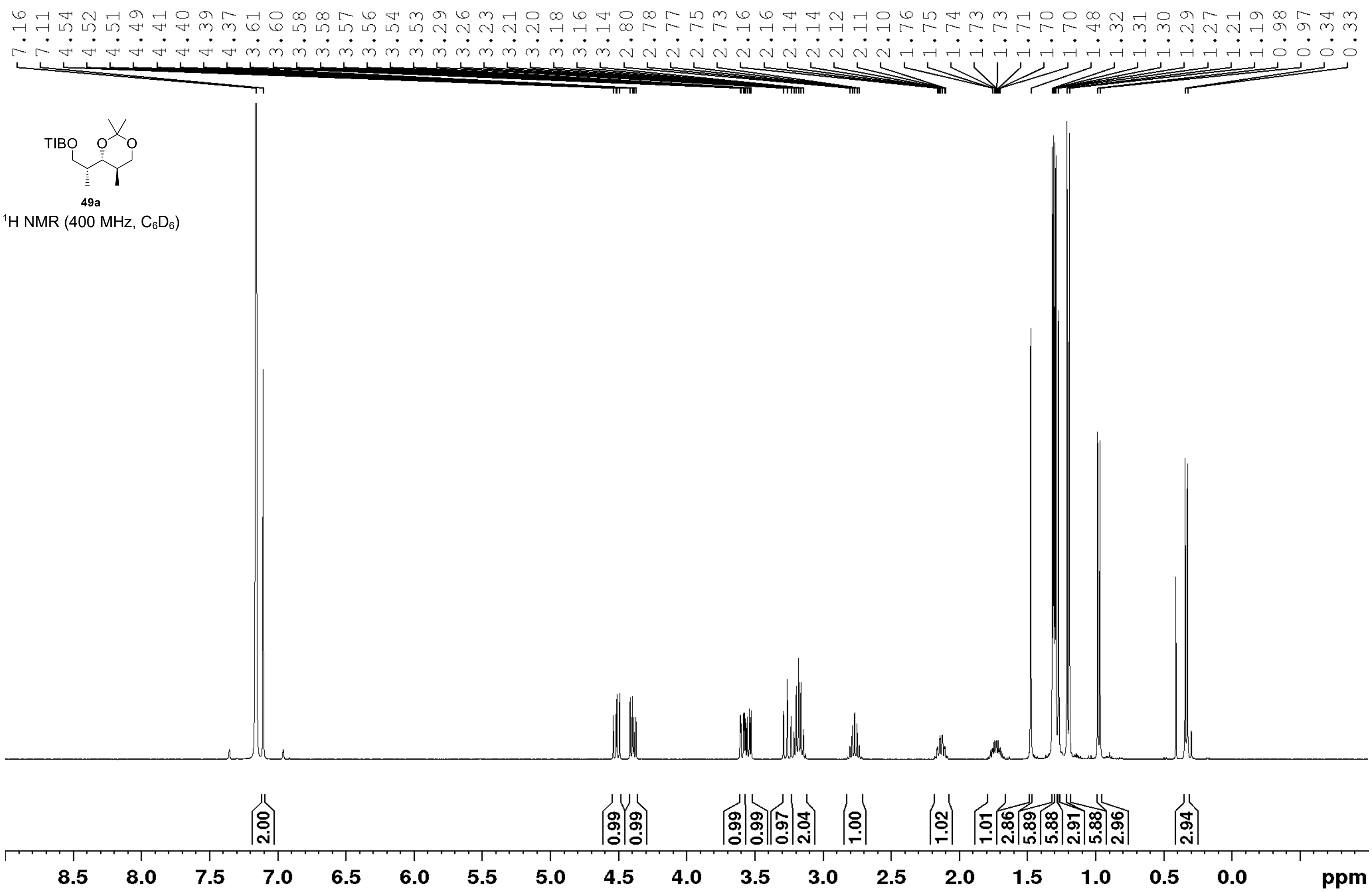
7.16 7.11 4.54 4.52 4.51 4.49 4.41 4.40 4.39 4.37 3.61 3.60 3.58 3.58 3.57 3.56 3.54 3.53 3.29 3.26 3.23 3.21 3.20 3.18 3.16 3.14 2.80 2.78 2.77 2.75 2.73 2.16 2.16 2.14 2.14 2.12 2.11 2.10 1.76 1.75 1.74 1.73 1.73 1.71 1.70 1.70 1.48 1.32 1.31 1.30 1.29 1.27 1.21 1.19 0.98 0.97 0.34 0.33
TIBO
49a
^{1}H NMR (400 MHz, C_6D_6)
2.00 0.99 0.99 0.99 0.99 0.97 2.04 1.00 1.02 1.01 2.86 5.89 5.88 2.91 5.88 2.96 2.94
8.5 8.0 7.5 7.0 6.5 6.0 5.5 5.0 4.5 4.0 3.5 3.0 2.5 2.0 1.5 1.0 0.5 0.0 ppm

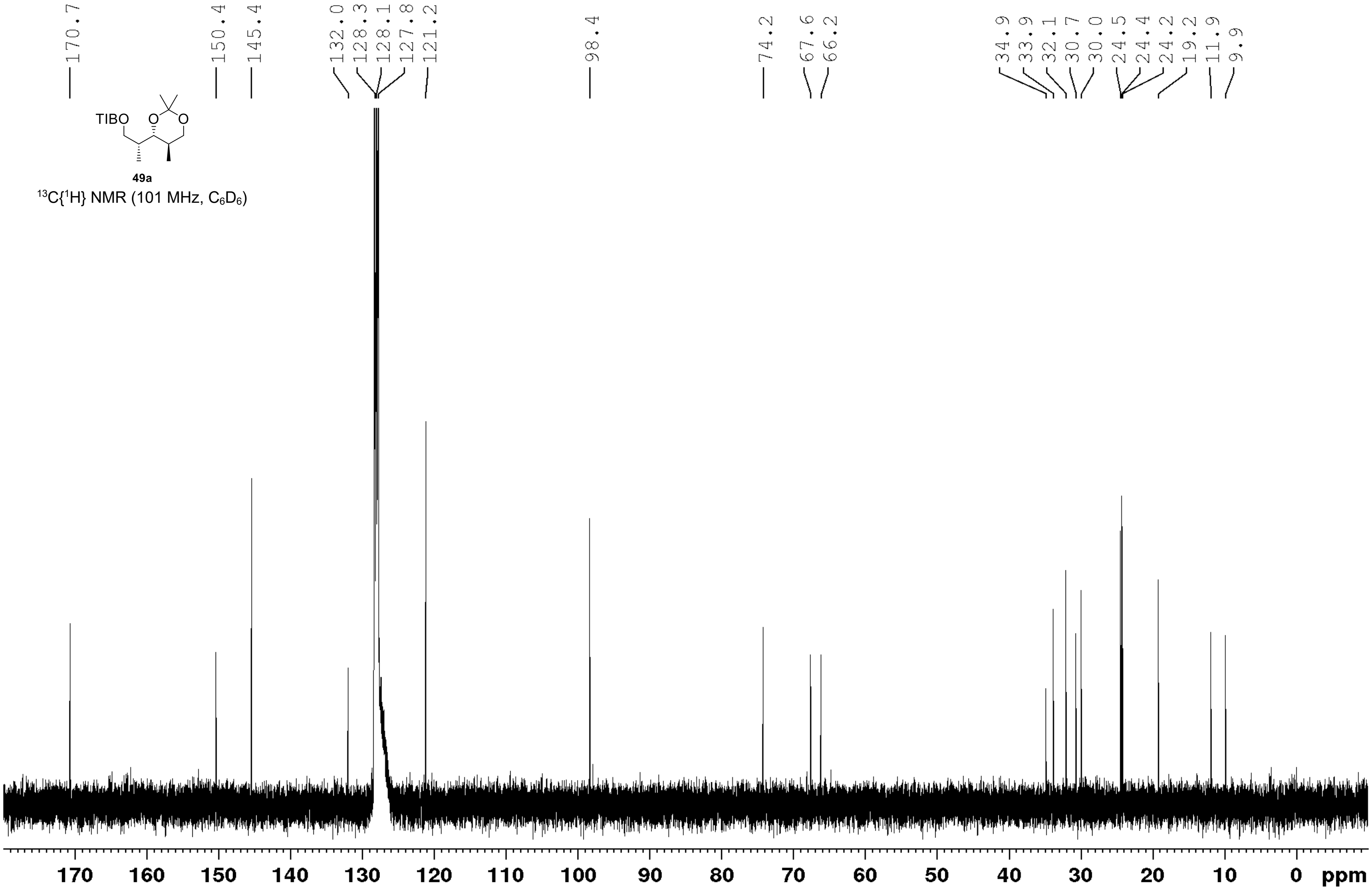
170.7
150.4
145.4
132.0
128.3
128.1
127.8
121.2
98.4
74.2
67.6
66.2
34.9
33.9
32.1
30.7
30.0
24.5
24.4
24.2
19.2
11.9
9.9
TIBO
O
O
49a
$^{13}C\{^{1}H\}$ NMR (101 MHz, C_6D_6)
170 160 150 140 130 120 110 100 90 80 70 60 50 40 30 20 10 0 ppm

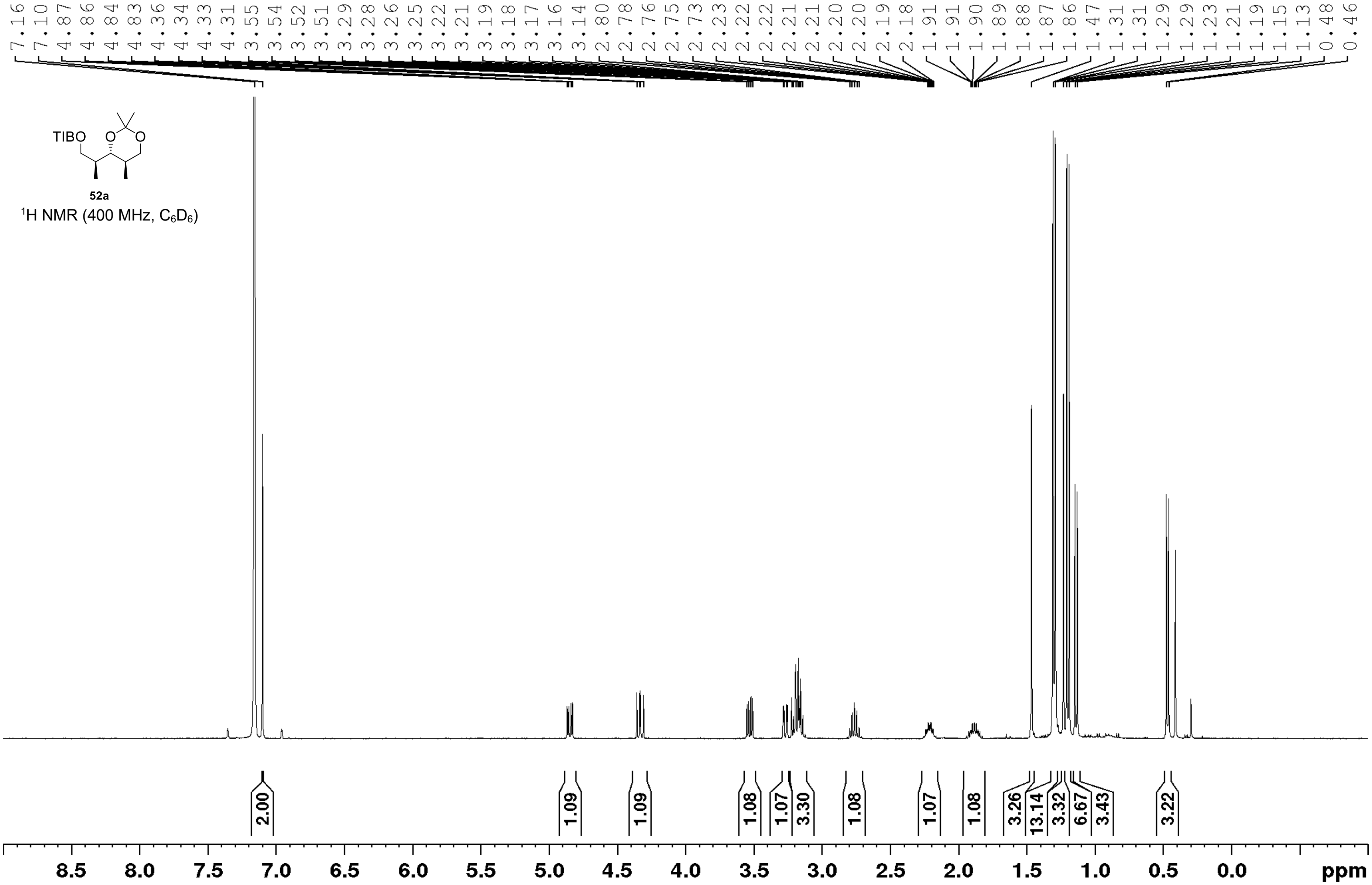
TIBO
52a
1H NMR (400 MHz, C6D6)
7.16 7.10 4.87 4.86 4.84 4.83 4.36 4.34 4.33 4.31 3.55 3.54 3.52 3.51 3.29 3.28 3.26 3.25 3.22 3.21 3.19 3.18 3.17 3.16 3.14 2.80 2.78 2.76 2.75 2.73 2.23 2.22 2.22 2.21 2.21 2.20 2.20 2.19 2.18 1.91 1.91 1.90 1.89 1.88 1.87 1.86 1.47 1.31 1.31 1.29 1.29 1.23 1.21 1.19 1.15 1.13 0.48 0.46
2.00 1.09 1.09 1.08 1.07 3.30 1.08 1.07 1.08 3.26 13.14 3.32 6.67 3.43 3.22
8.5 8.0 7.5 7.0 6.5 6.0 5.5 5.0 4.5 4.0 3.5 3.0 2.5 2.0 1.5 1.0 0.5 0.0 ppm

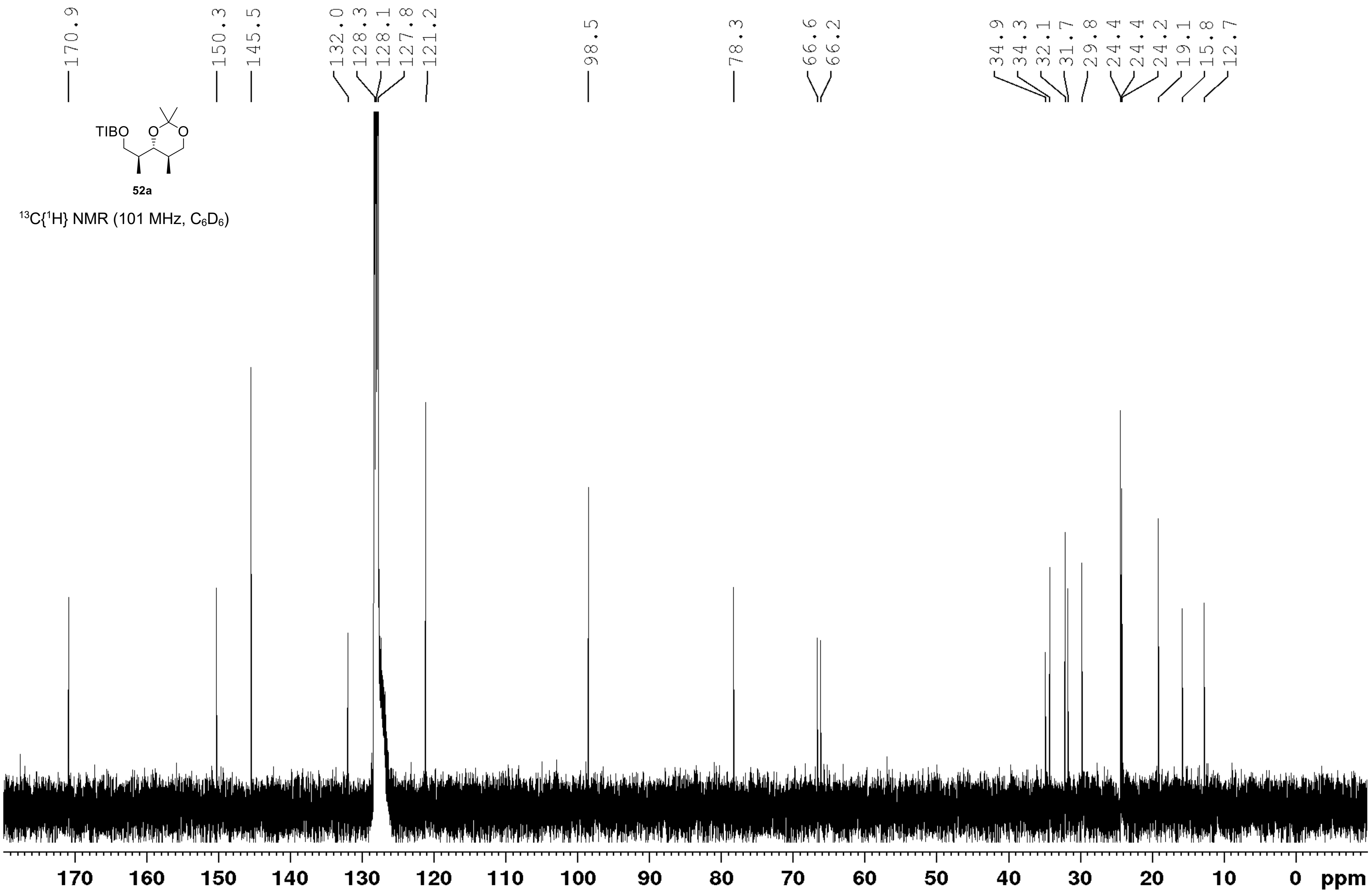

$^{13}C\{^1H\}$ NMR (101 MHz, C_6D_6)

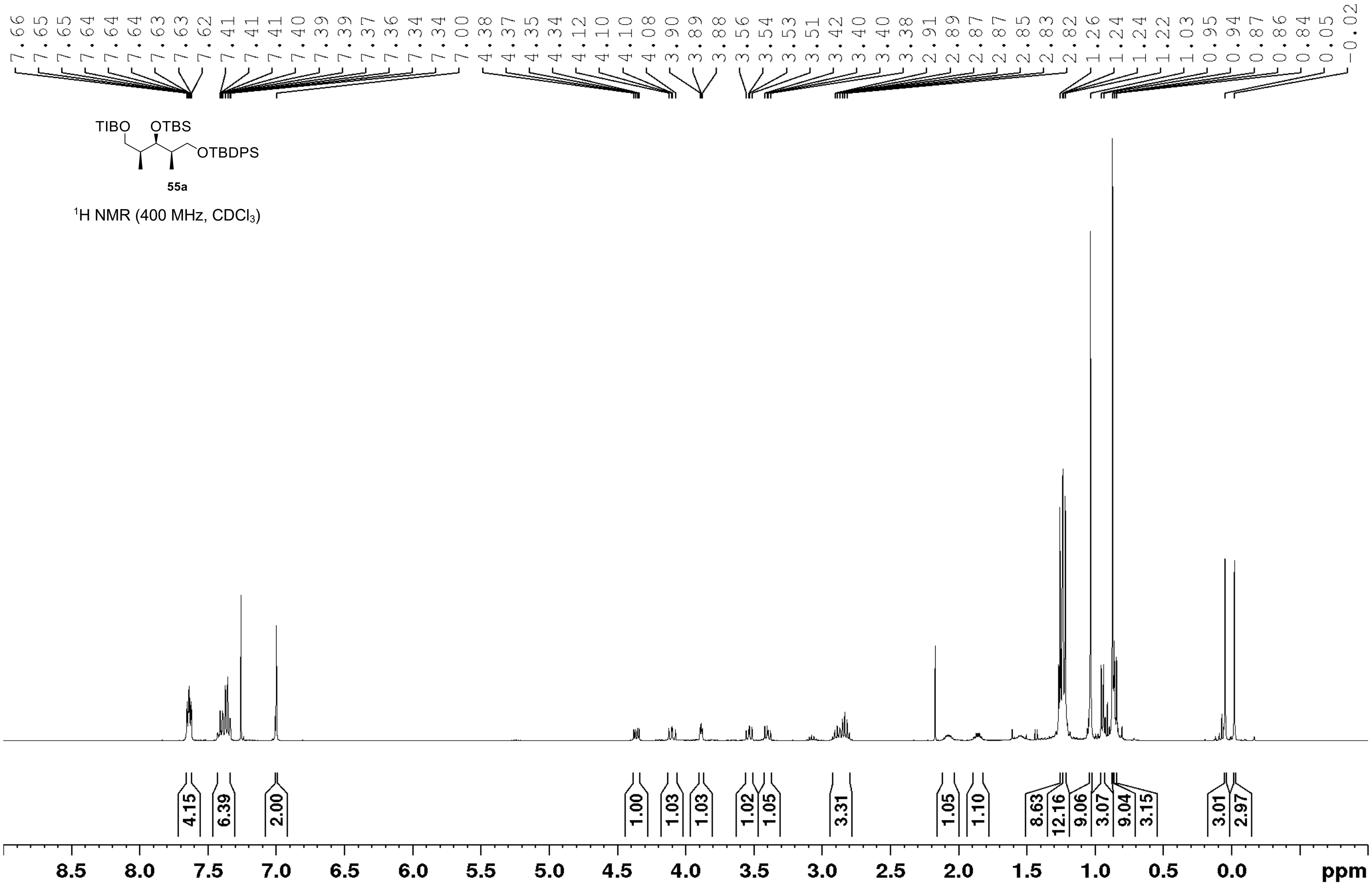

55a

^{1}H NMR (400 MHz, $CDCl_3$)

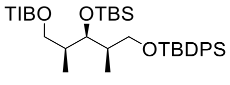

55a

$^{13}C\{^1H\}$ NMR (101 MHz, $CDCl_3$)

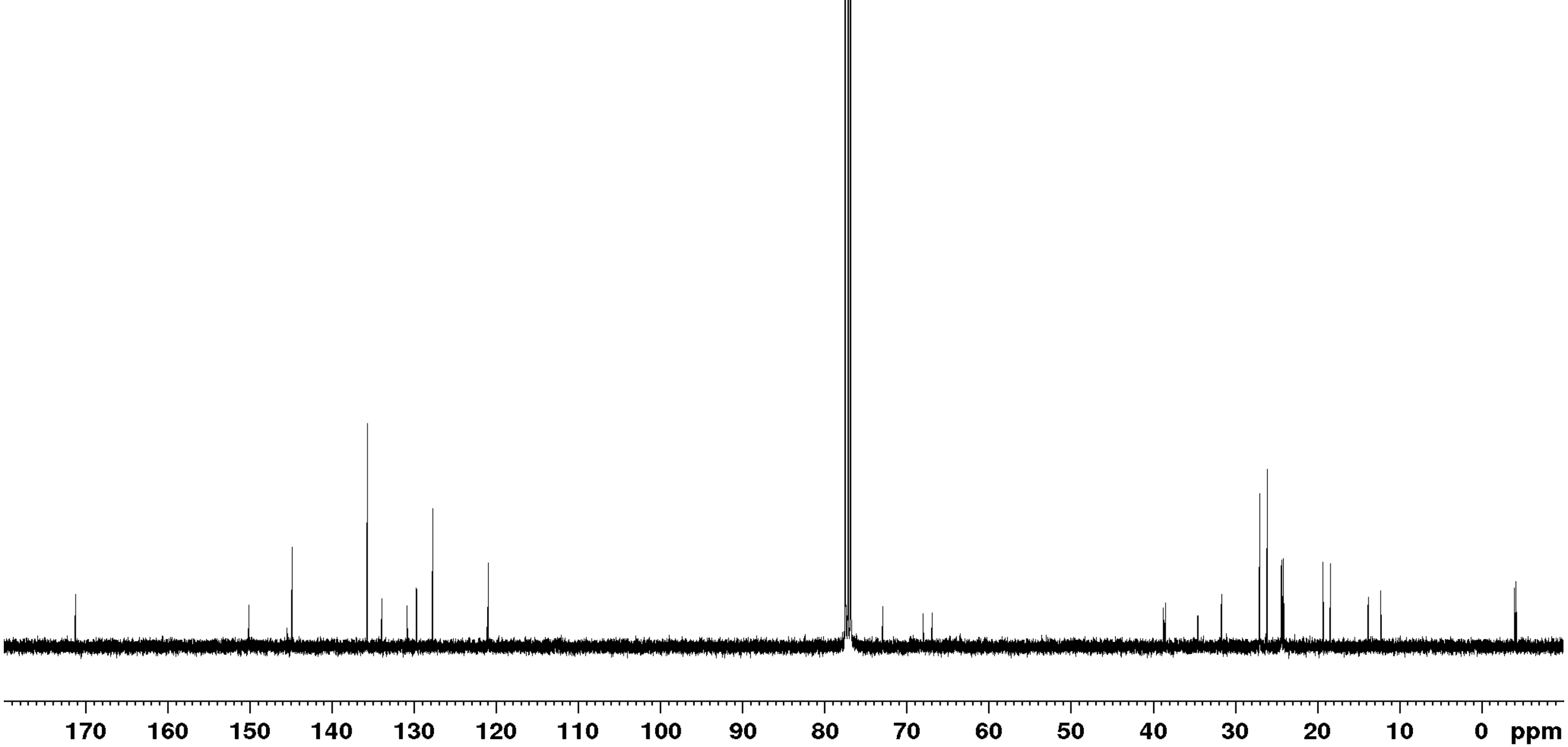

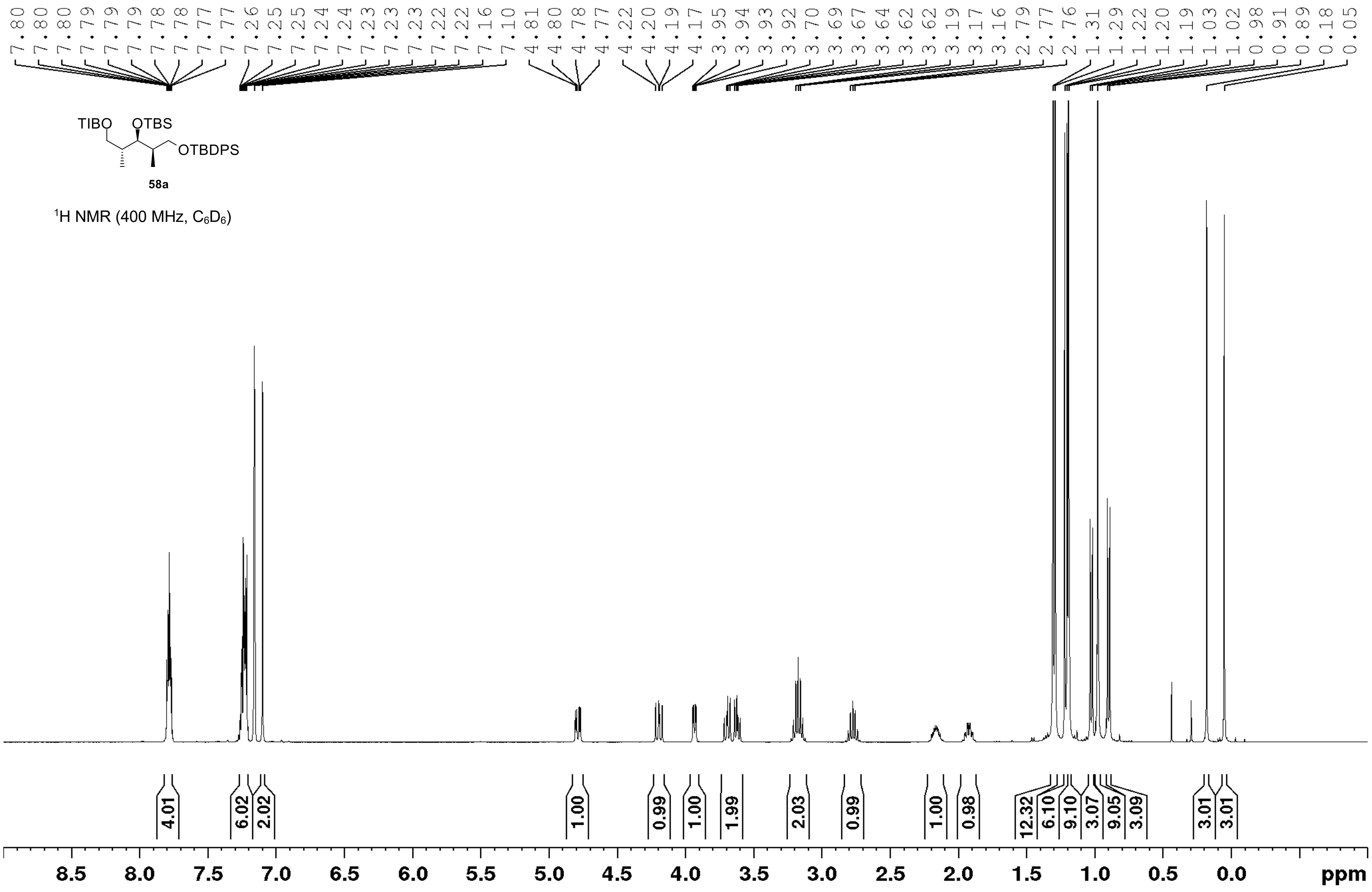
TIBO
OTBS
OTBDPS
58a
^{1}H NMR (400 MHz, C_6D_6)
7.80 7.80 7.80 7.79 7.79 7.79 7.78 7.78 7.77 7.77 7.26 7.25 7.25 7.24 7.24 7.23 7.23 7.23 7.22 7.22 7.16 7.10 4.81 4.80 4.78 4.77 4.22 4.20 4.19 4.17 3.95 3.94 3.93 3.92 3.70 3.69 3.67 3.64 3.62 3.62 3.19 3.17 3.16 2.79 2.77 2.76 1.31 1.29 1.22 1.20 1.19 1.03 1.02 0.98 0.91 0.89 0.18 0.05
4.01 6.02 2.02 1.00 0.99 1.00 1.99 2.03 0.99 1.00 0.98 12.32 6.10 9.10 3.07 9.05 3.09 3.01 3.01
8.5 8.0 7.5 7.0 6.5 6.0 5.5 5.0 4.5 4.0 3.5 3.0 2.5 2.0 1.5 1.0 0.5 0.0 ppm

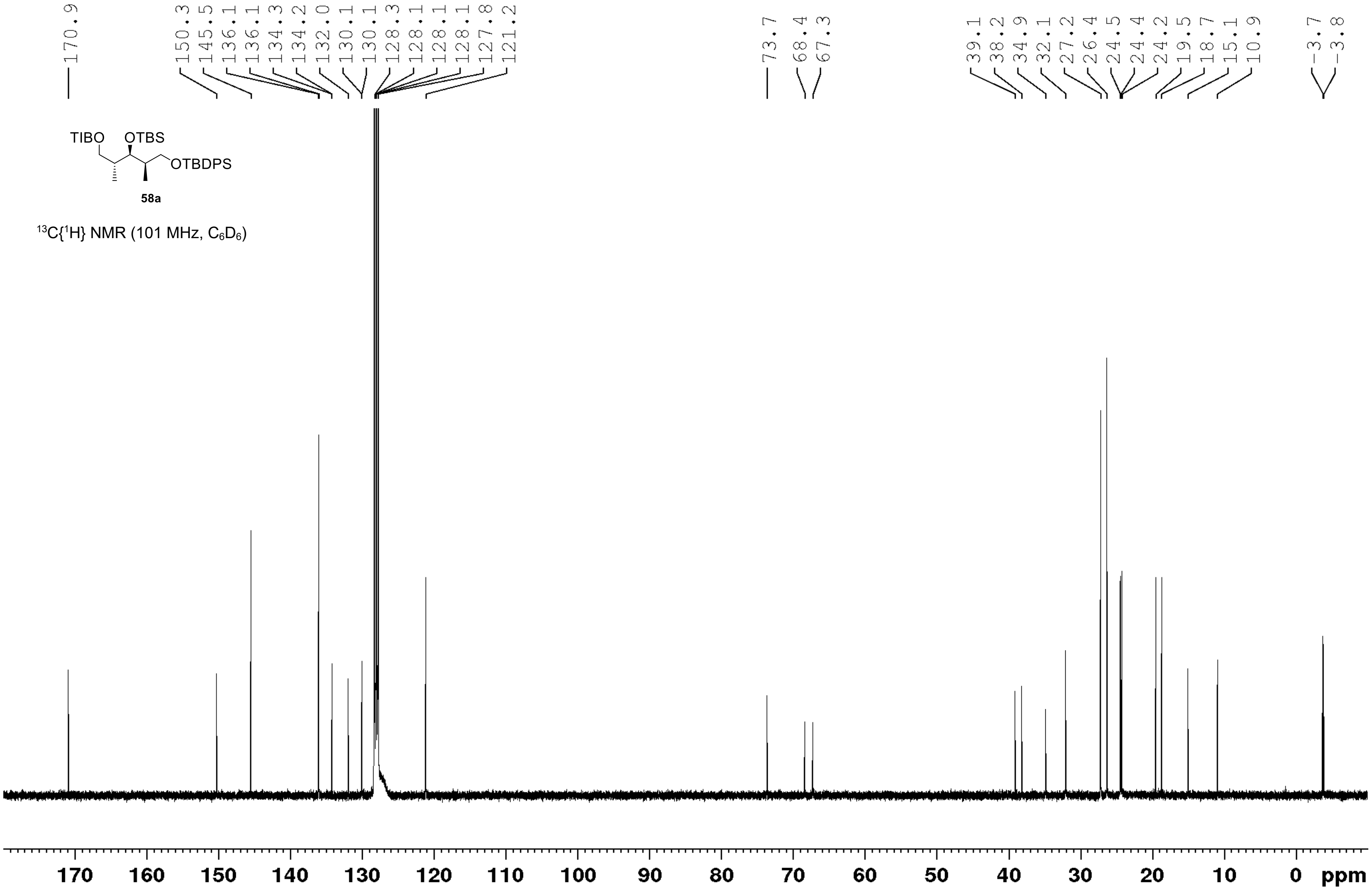

58a

$^{13}C\{^{1}H\}$ NMR (101 MHz, C_6D_6)

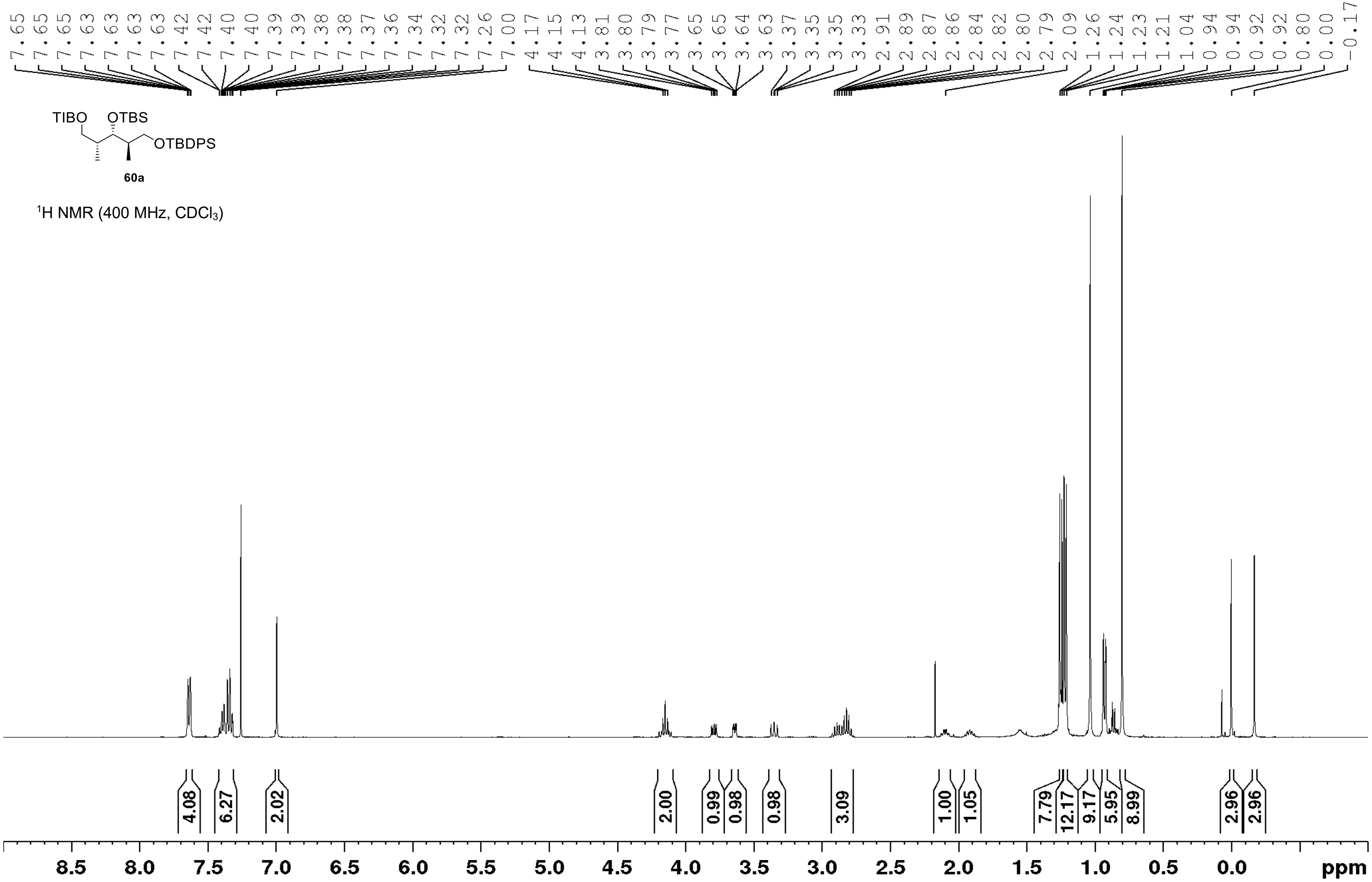

TIBO
OTBS
OTBDPS
60a
^{1}H NMR (400 MHz, CDCl$_3$)
7.65 7.65 7.65 7.63 7.63 7.63 7.63 7.42 7.42 7.40 7.40 7.39 7.39 7.38 7.38 7.37 7.36 7.34 7.32 7.32 7.26 7.00 4.17 4.15 4.13 3.81 3.80 3.79 3.77 3.65 3.65 3.64 3.63 3.37 3.35 3.35 3.33 2.91 2.89 2.87 2.86 2.84 2.82 2.80 2.79 2.09 1.26 1.24 1.23 1.21 1.04 0.94 0.94 0.92 0.92 0.80 0.00 -0.17
4.08 6.27 2.02 2.00 0.99 0.98 0.98 3.09 1.00 1.05 7.79 12.17 9.17 5.95 8.99 2.96 2.96
8.5 8.0 7.5 7.0 6.5 6.0 5.5 5.0 4.5 4.0 3.5 3.0 2.5 2.0 1.5 1.0 0.5 0.0 ppm

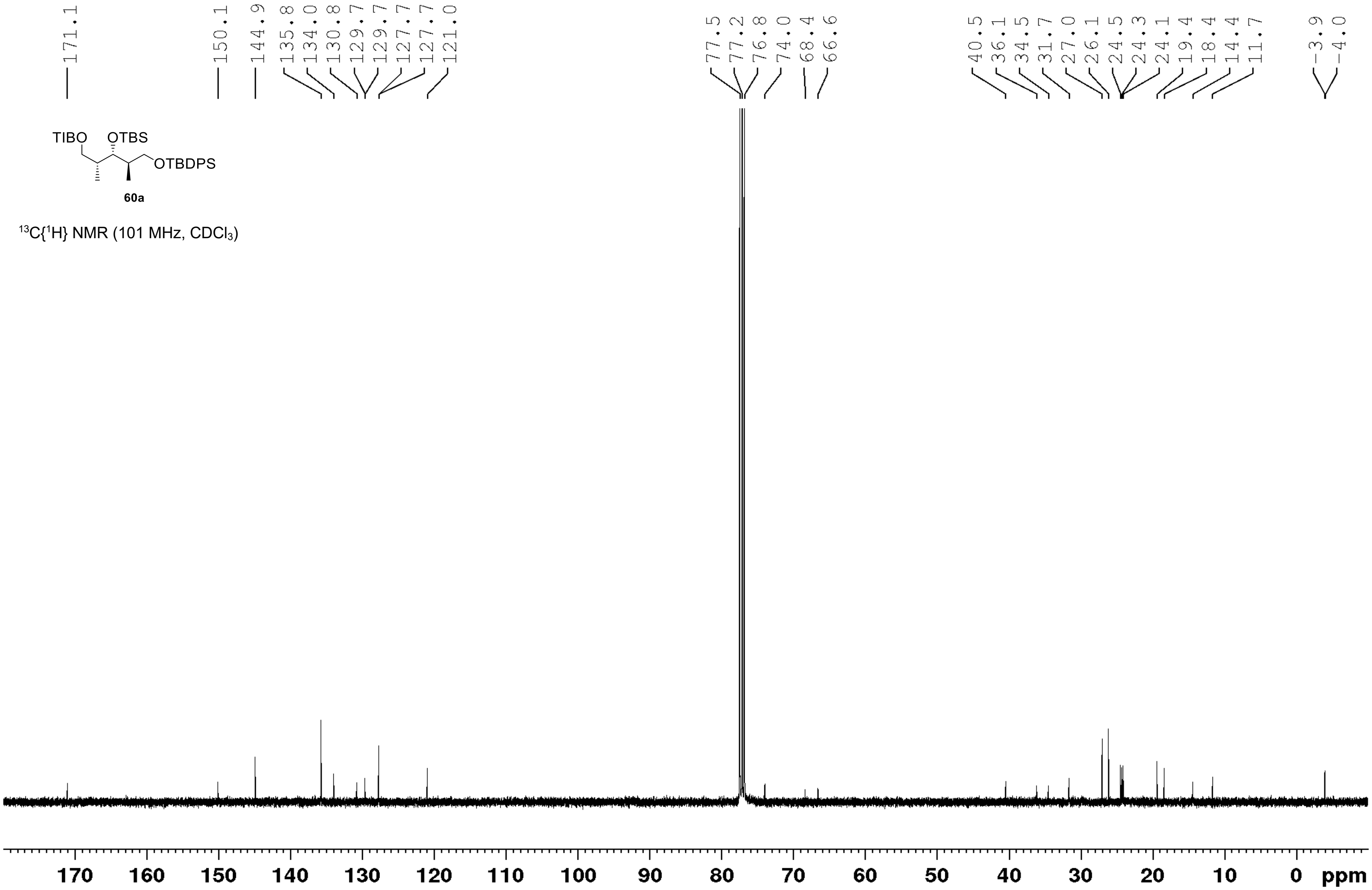

60a

$^{13}C\{^{1}H\}$ NMR (101 MHz, $CDCl_3$)

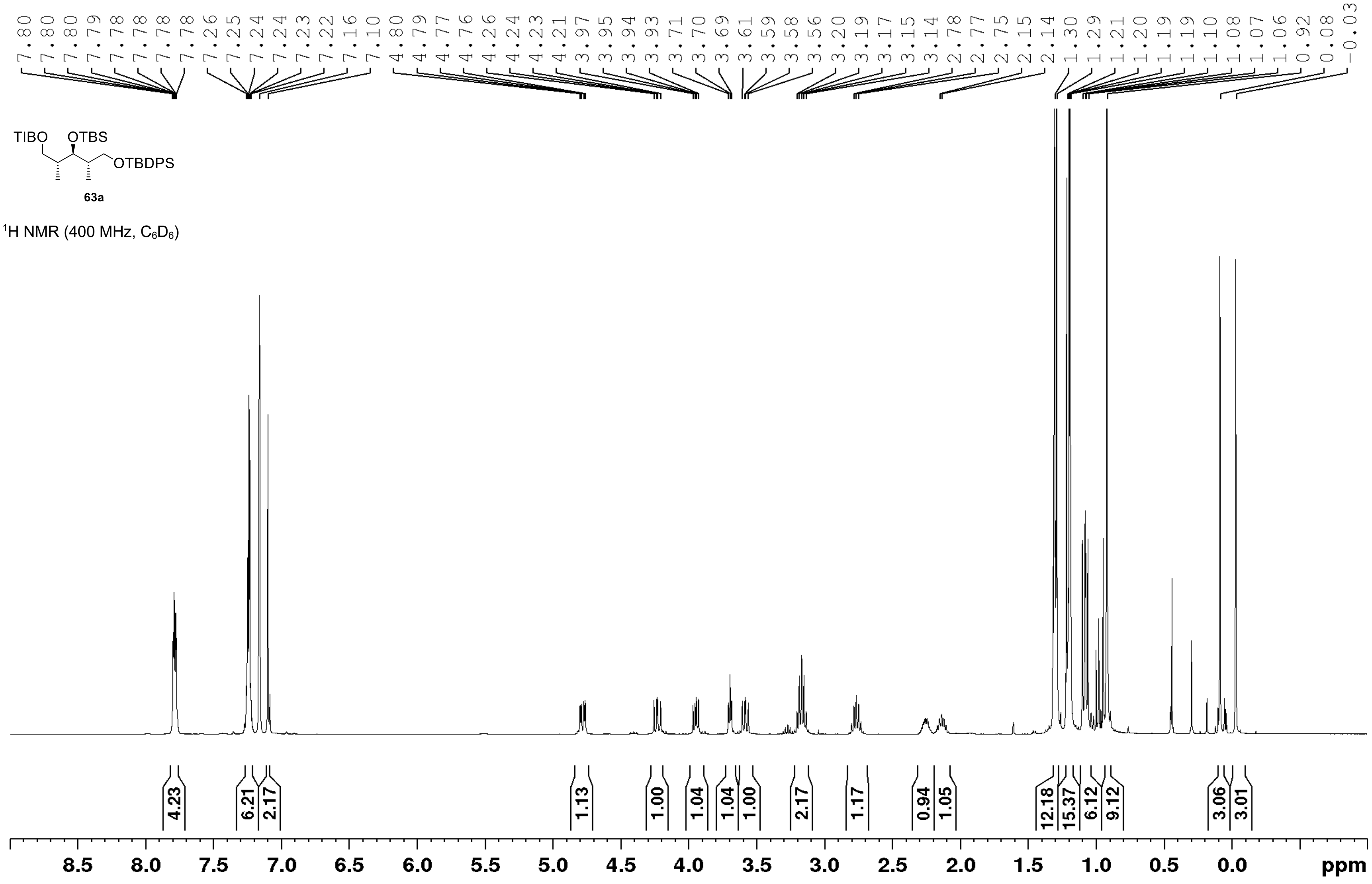
TIBO
OTBS
OTBDPS
63a
1H NMR (400 MHz, C6D6)
7.80 7.80 7.80 7.79 7.78 7.78 7.78 7.78 7.26 7.25 7.24 7.24 7.23 7.22 7.16 7.10 4.80 4.79 4.77 4.76 4.26 4.24 4.23 4.21 3.97 3.95 3.94 3.93 3.71 3.70 3.69 3.61 3.59 3.58 3.56 3.20 3.19 3.17 3.15 3.14 2.78 2.77 2.75 2.15 2.14 1.30 1.29 1.21 1.20 1.19 1.19 1.10 1.08 1.07 1.06 0.92 0.08 -0.03
4.23 6.21 2.17 1.13 1.00 1.04 1.04 1.00 2.17 1.17 0.94 1.05 12.18 15.37 6.12 9.12 3.06 3.01
8.5 8.0 7.5 7.0 6.5 6.0 5.5 5.0 4.5 4.0 3.5 3.0 2.5 2.0 1.5 1.0 0.5 0.0 ppm

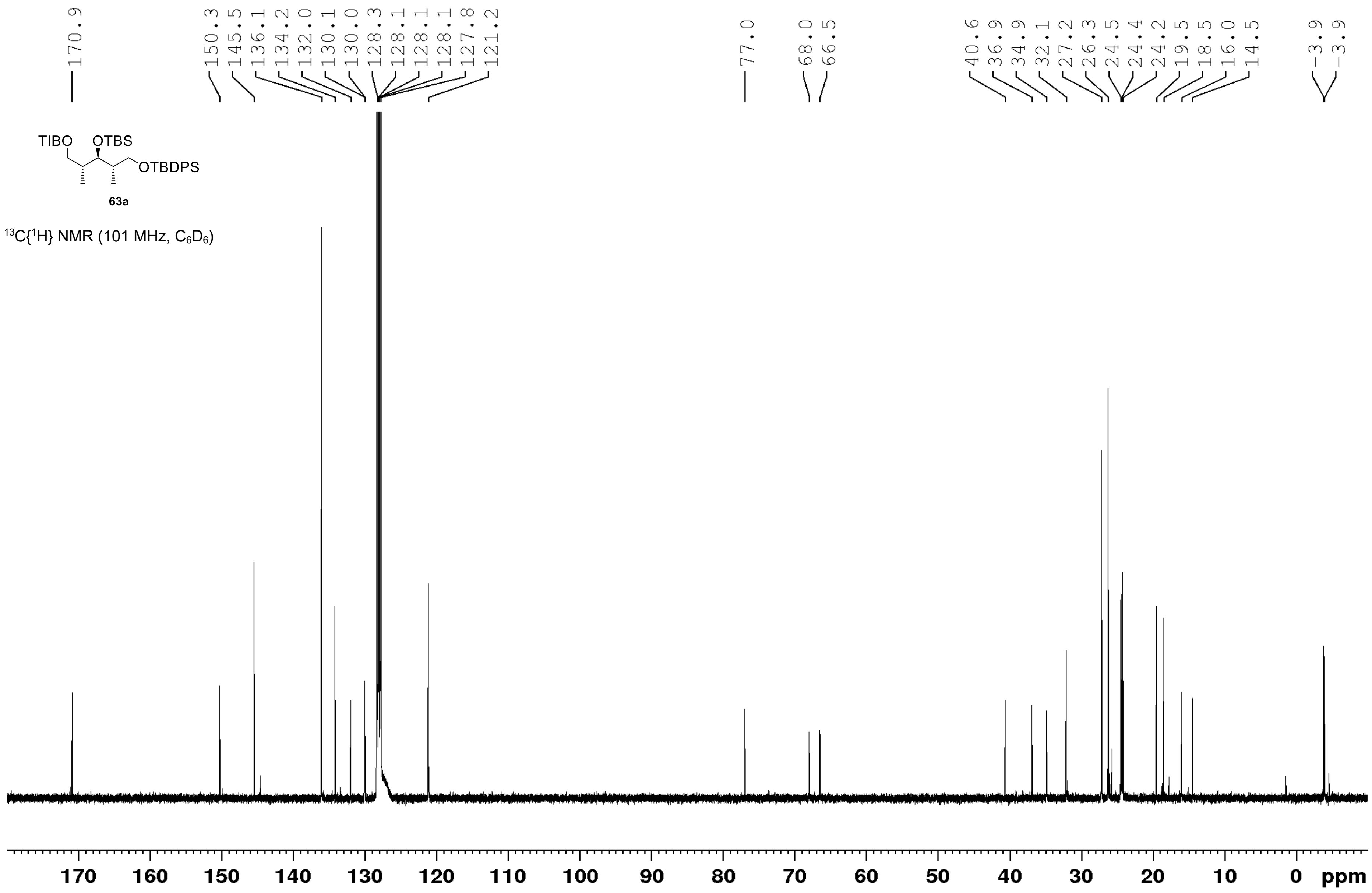
170.9
150.3
145.5
136.1
134.2
132.0
130.1
130.0
128.3
128.1
128.1
128.1
127.8
121.2
77.0
68.0
66.5
40.6
36.9
34.9
32.1
27.2
26.3
24.5
24.4
24.2
19.5
18.5
16.0
14.5
-3.9
-3.9
TIBO
OTBS
OTBDPS
63a
$^{13}C\{^1H\}$ NMR (101 MHz, C_6D_6)
170 160 150 140 130 120 110 100 90 80 70 60 50 40 30 20 10 0 ppm

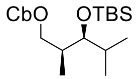

9b

^{1}H NMR (400 MHz, $CDCl_3$)

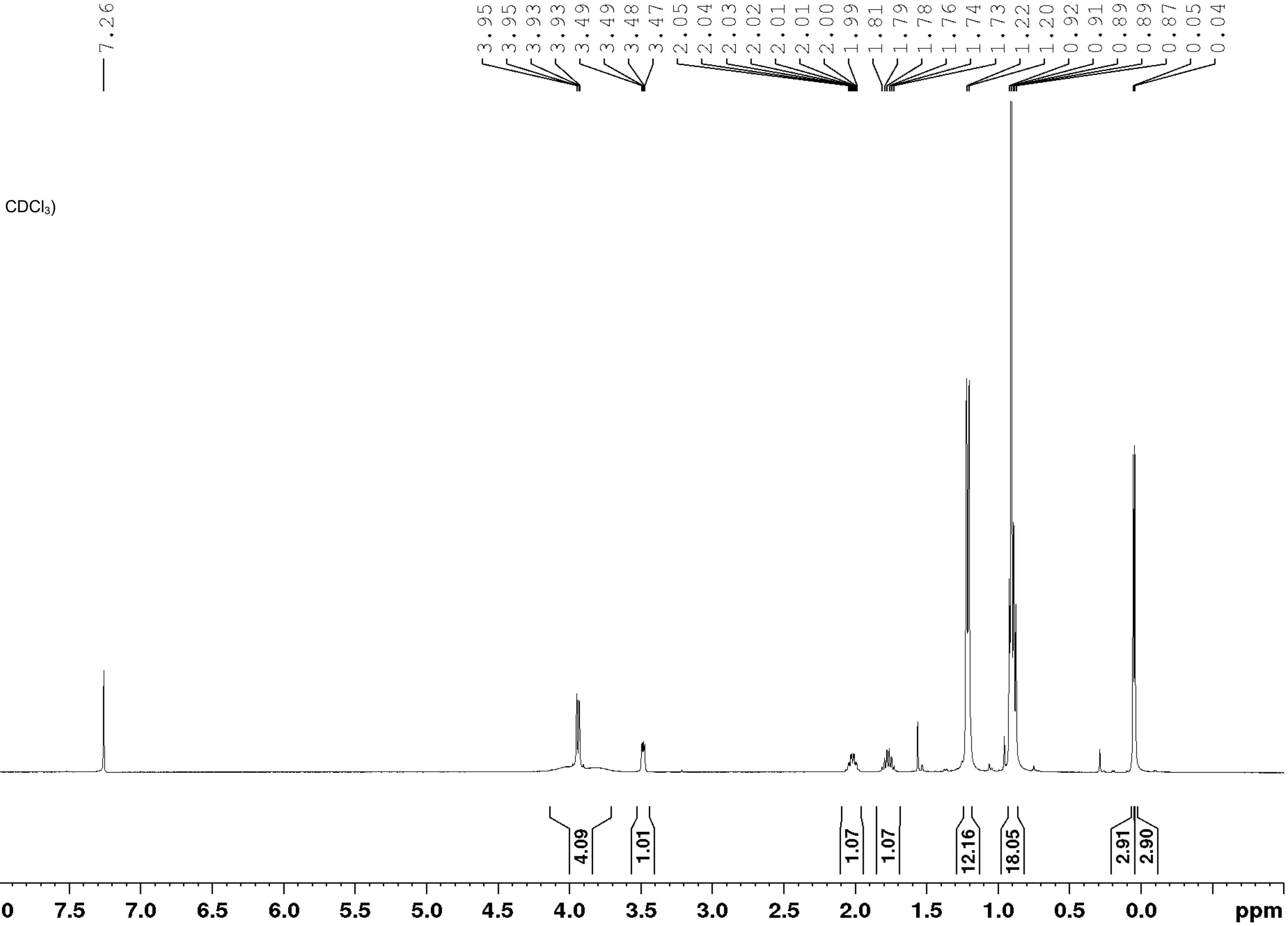

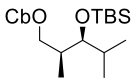

9b

$^{13}C\{^1H\}$ NMR (101 MHz, $CDCl_3$)

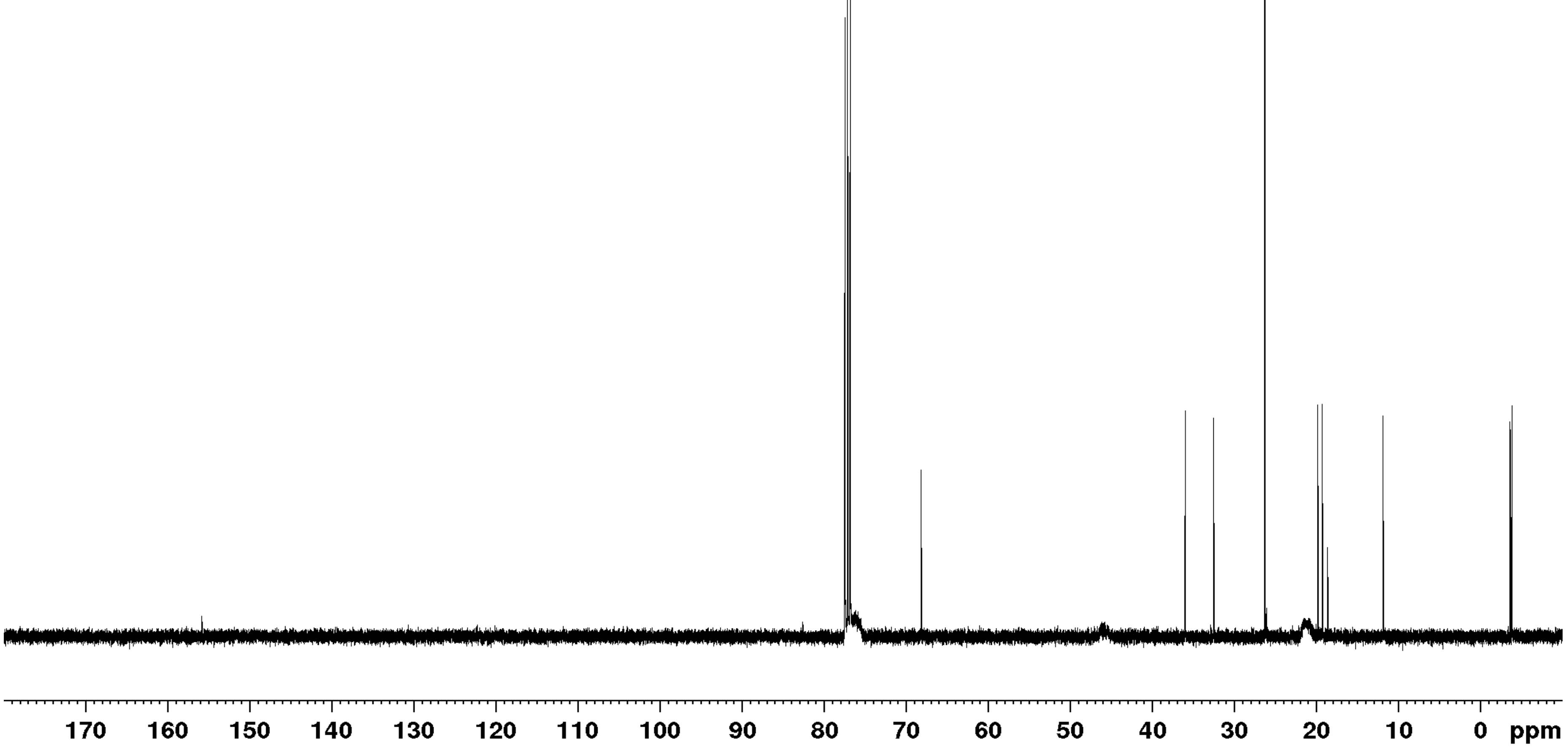

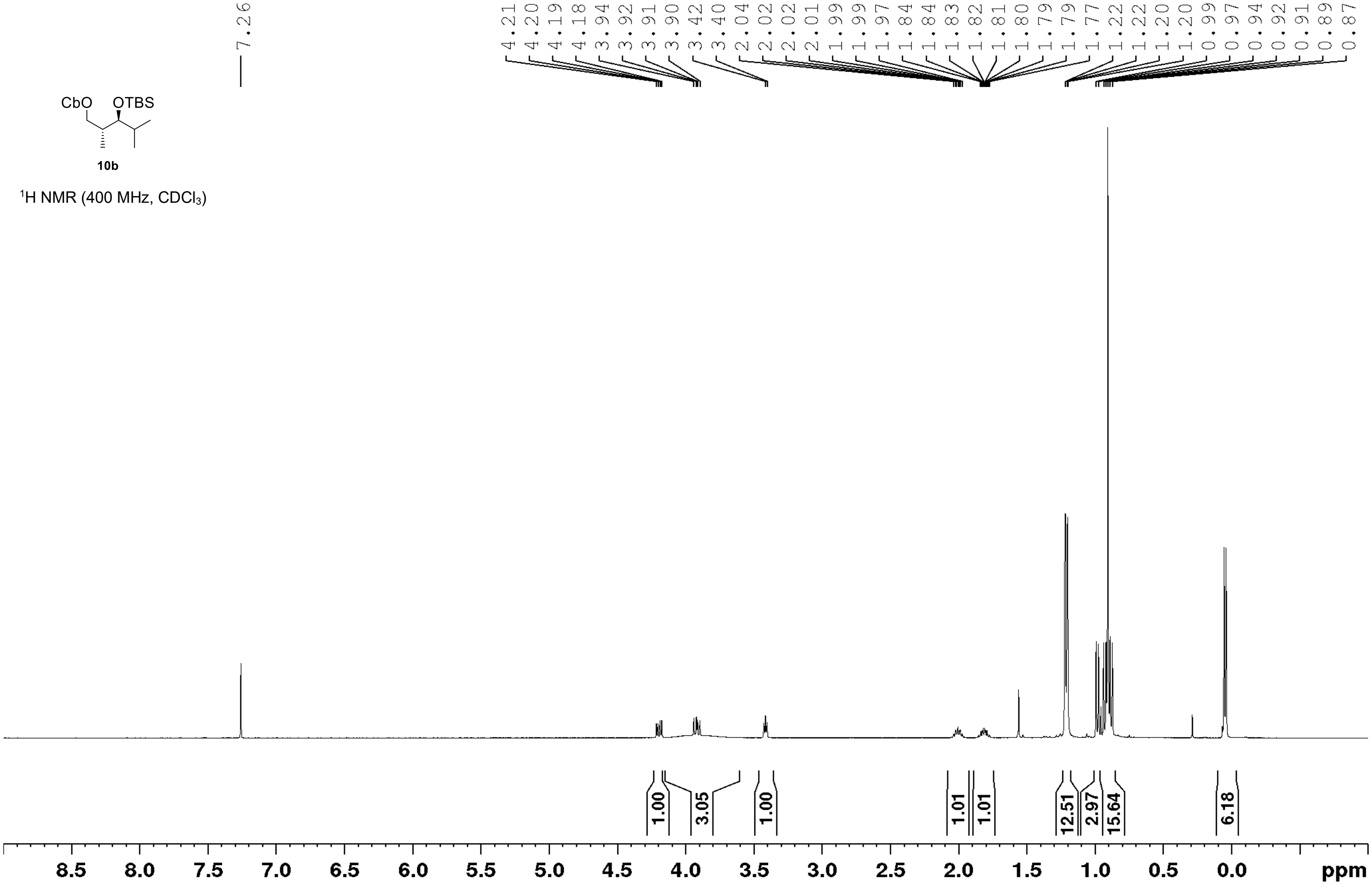

10b

^{1}H NMR (400 MHz, $CDCl_3$)

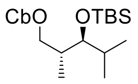

10b

$^{13}C\{^{1}H\}$ NMR (101 MHz, $CDCl_3$)

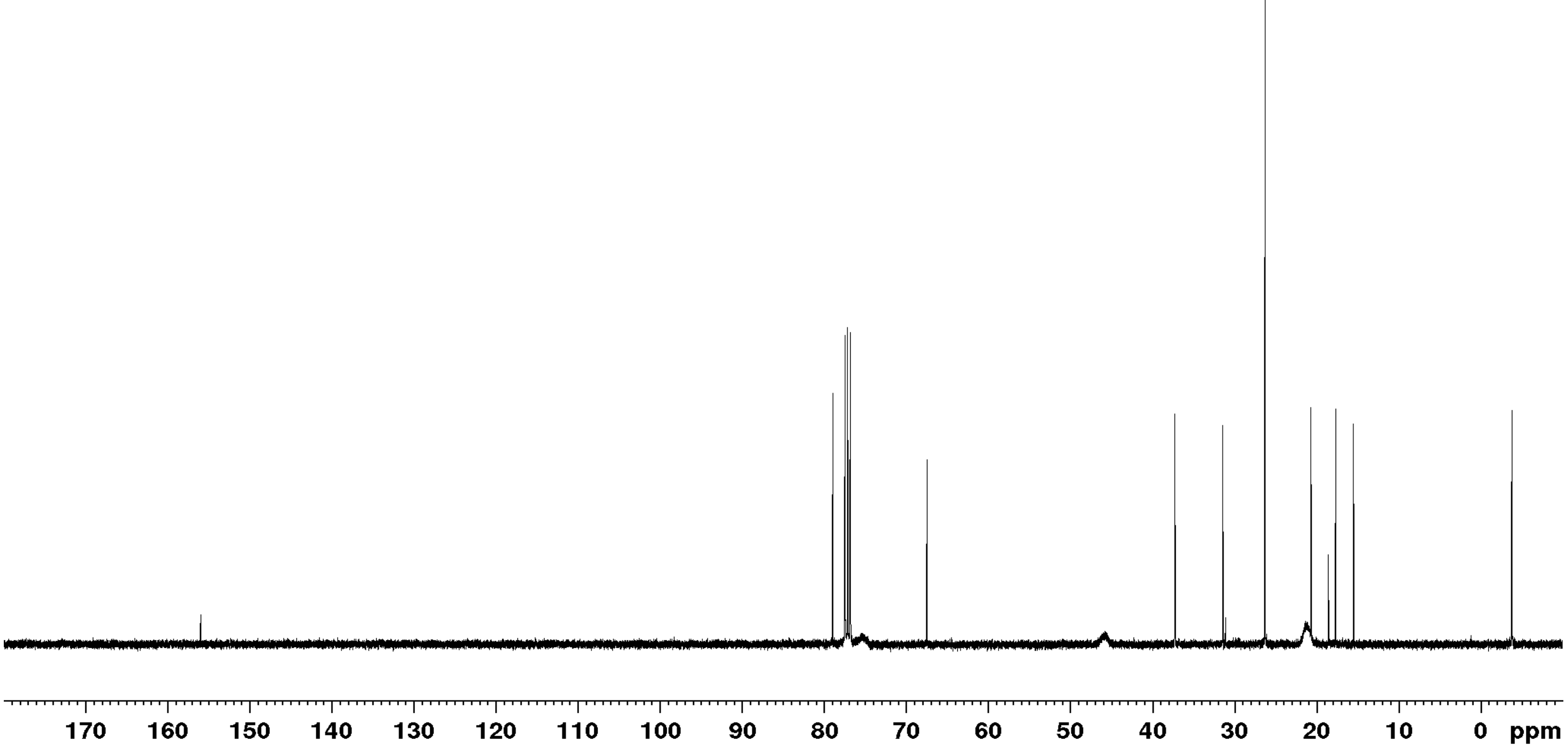

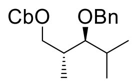

25b

^{1}H NMR (400 MHz, $CDCl_3$)

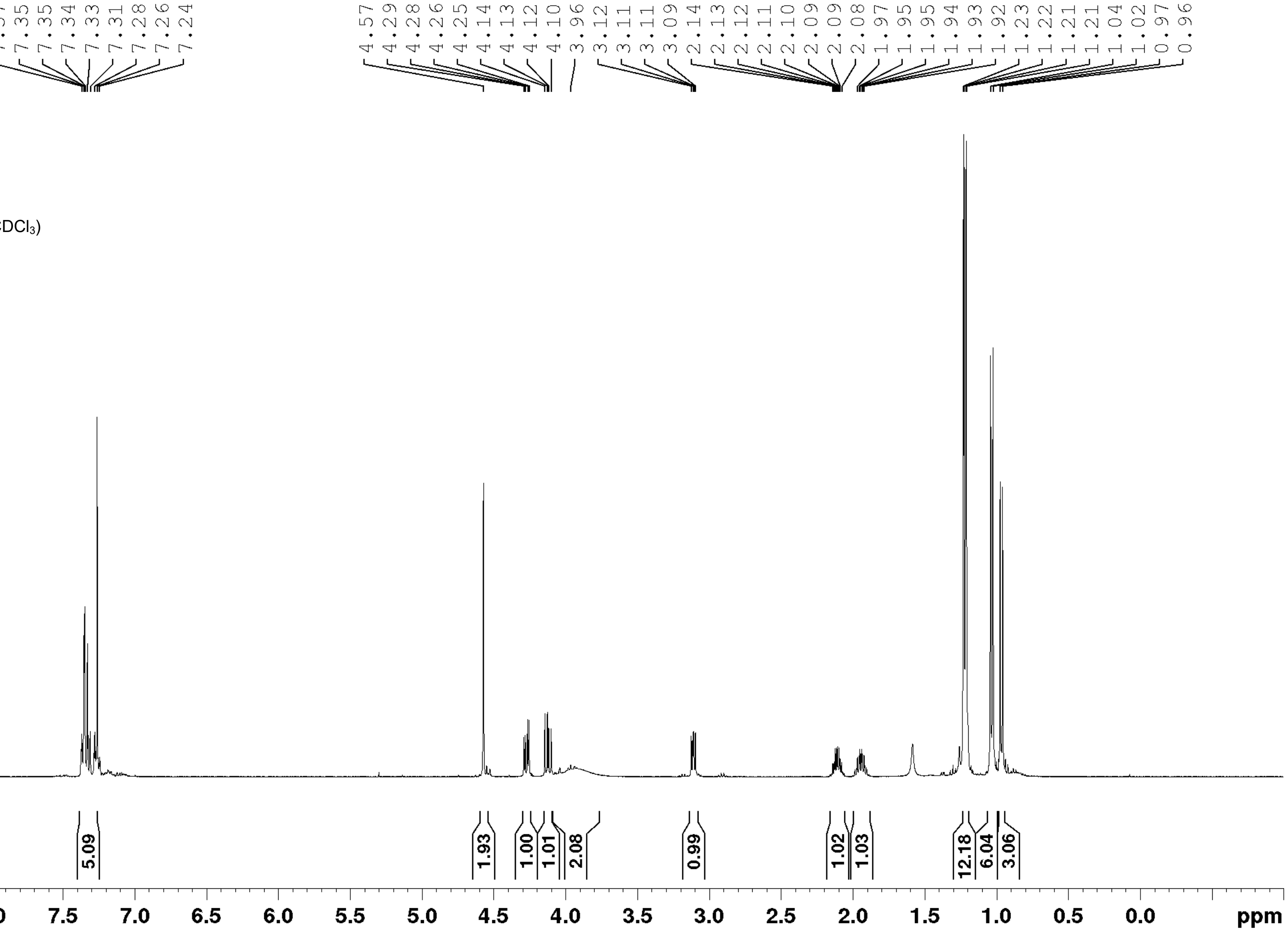

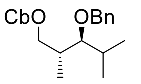

25b

$^{13}C\{^{1}H\}$ NMR (101 MHz, $CDCl_3$)

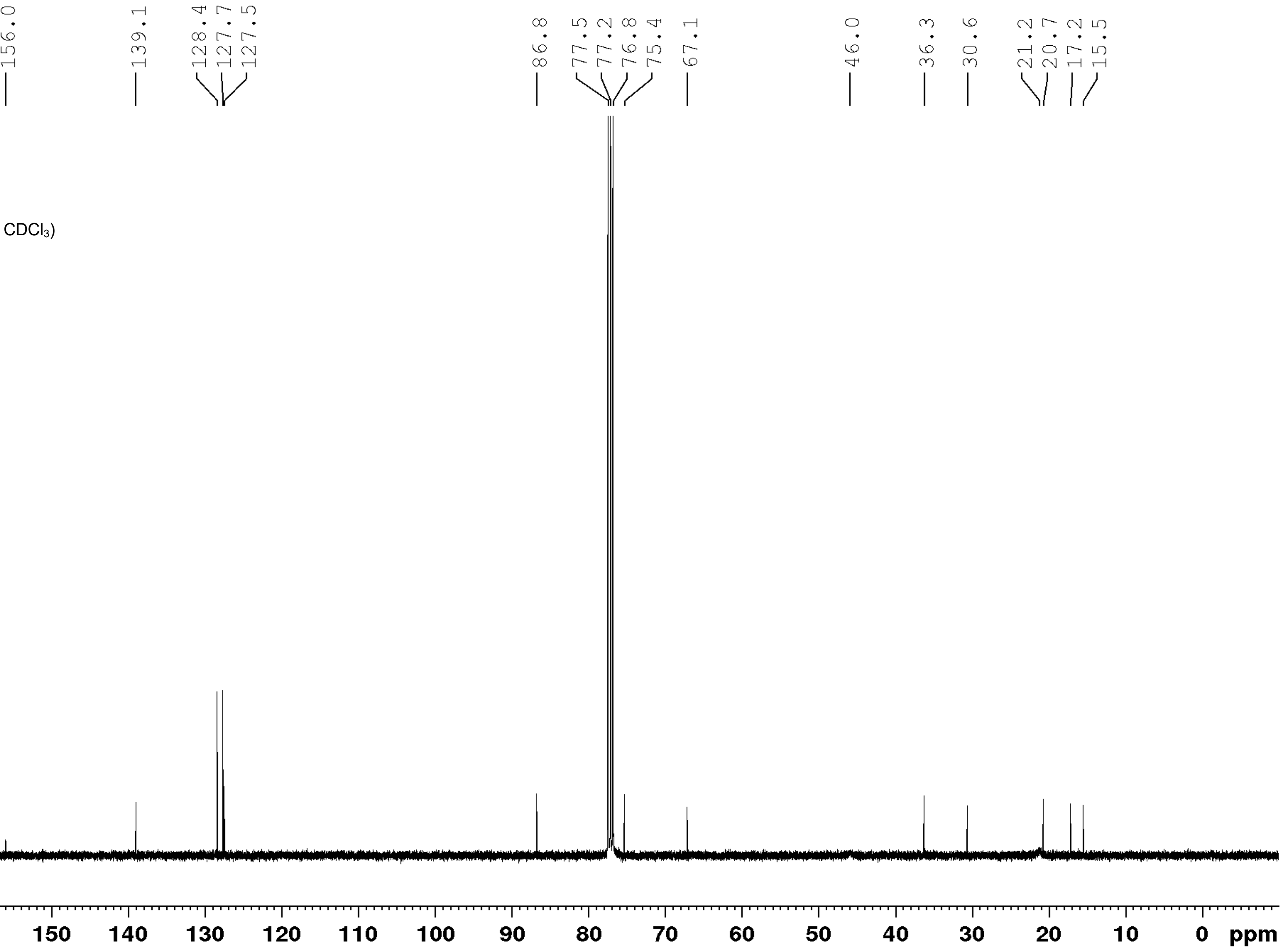

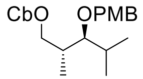

26b

^{1}H NMR (400 MHz, $CDCl_3$)

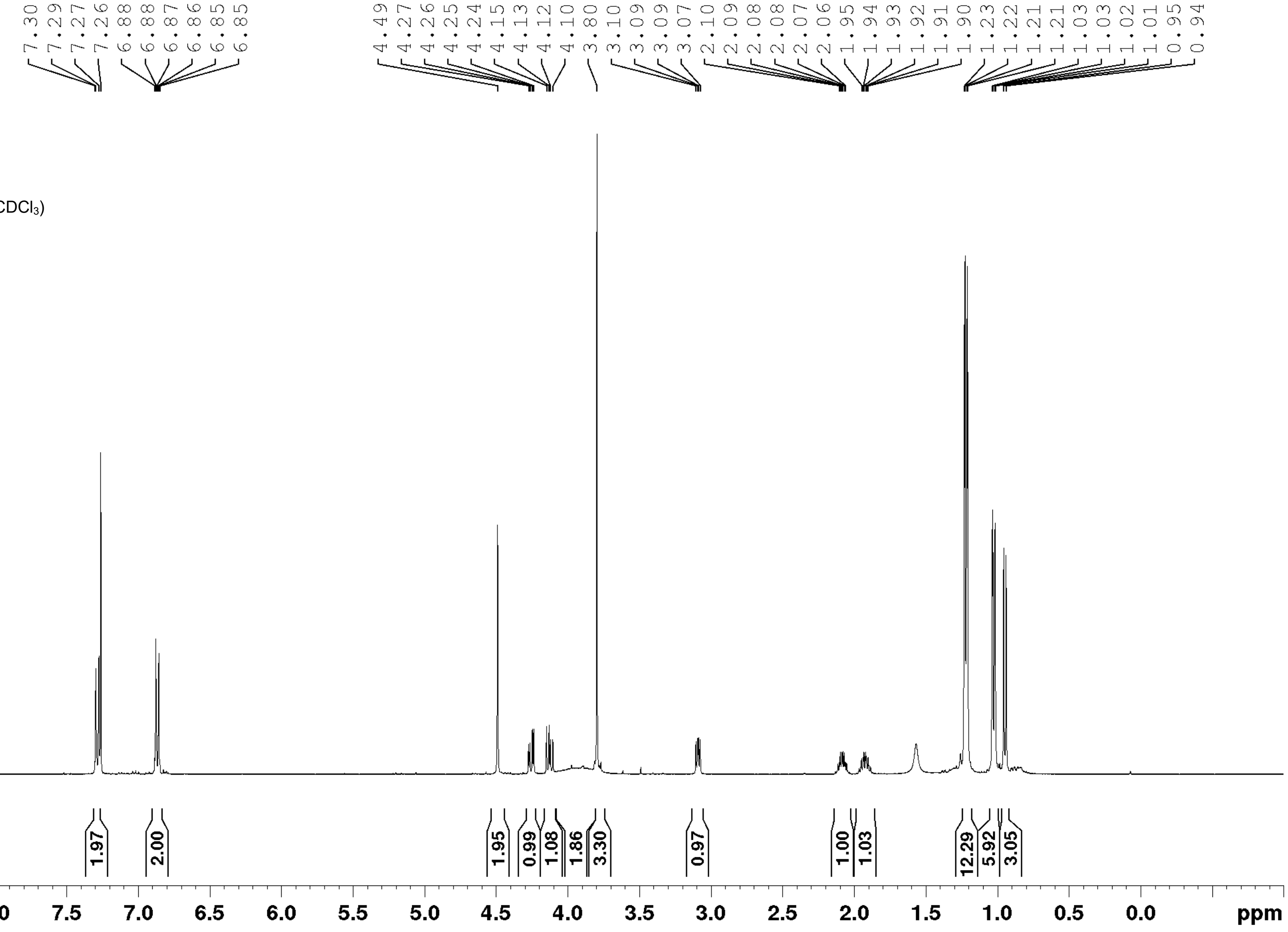

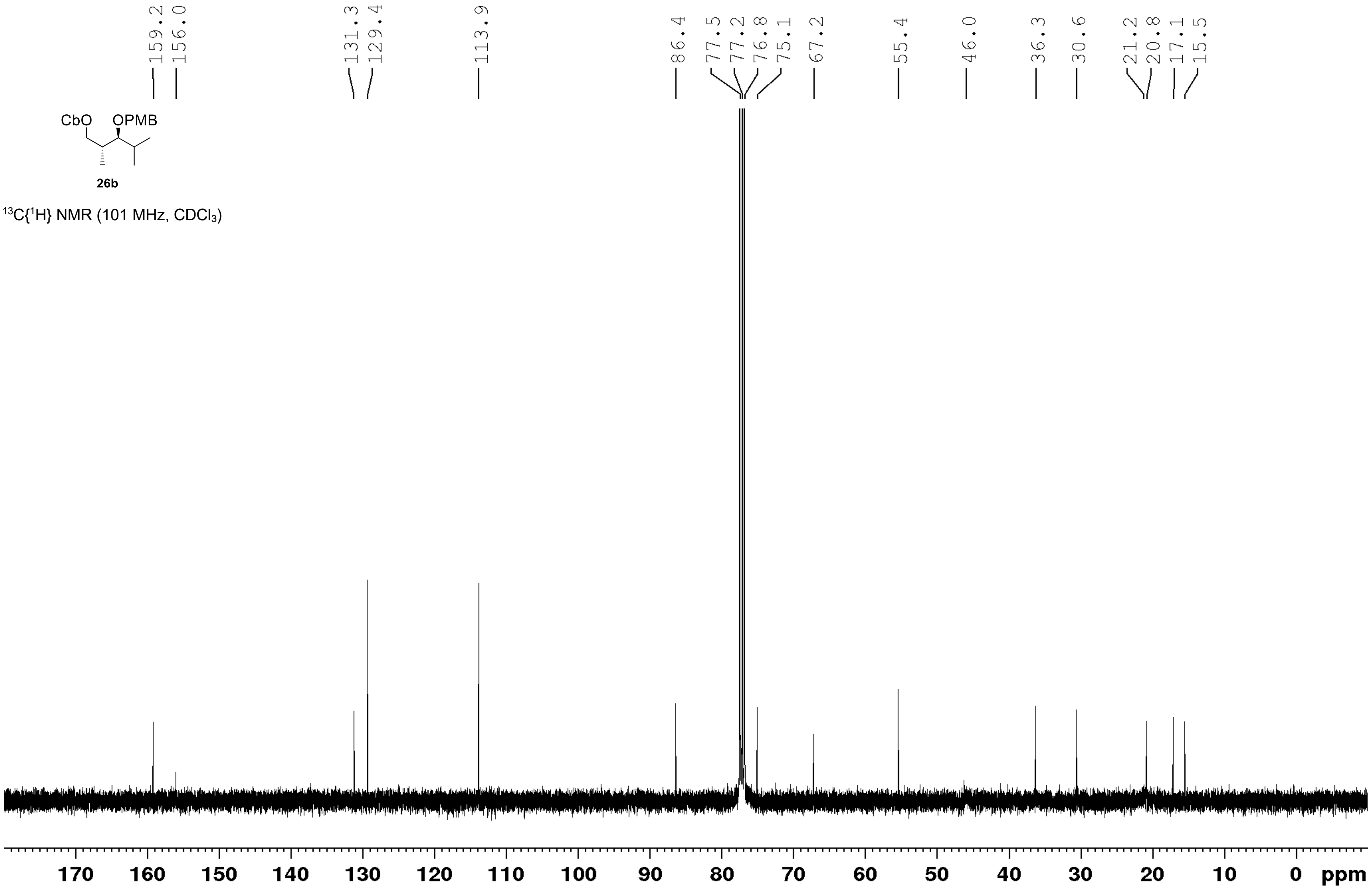
159.2
156.0
131.3
129.4
113.9
86.4
77.5
77.2
76.8
75.1
67.2
55.4
46.0
36.3
30.6
21.2
20.8
17.1
15.5
CbO
OPMB
26b
$^{13}C\{^{1}H\}$ NMR (101 MHz, $CDCl_3$)
170
160
150
140
130
120
110
100
90
80
70
60
50
40
30
20
10
0
ppm

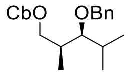

29b

^{1}H NMR (400 MHz, $CDCl_3$)

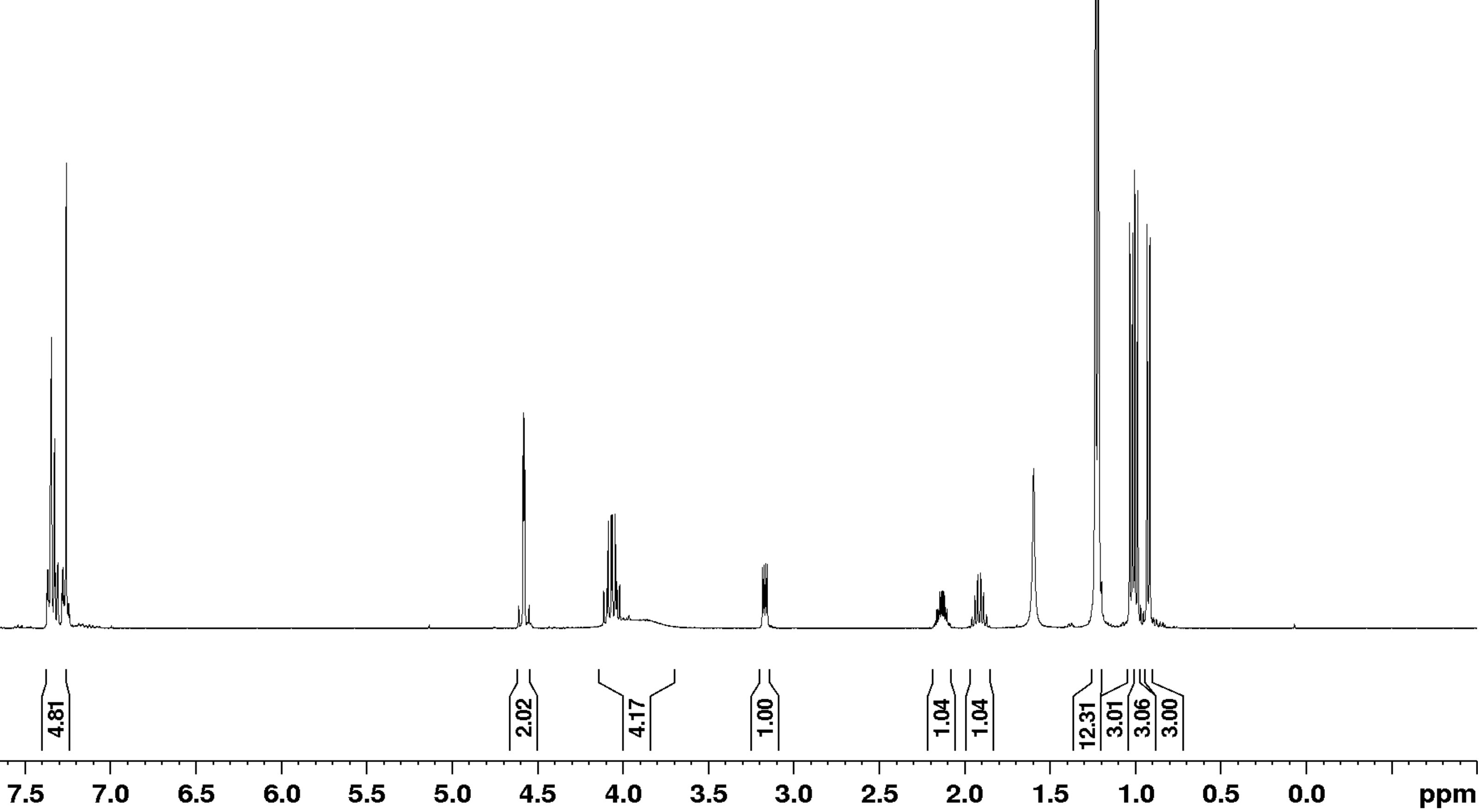

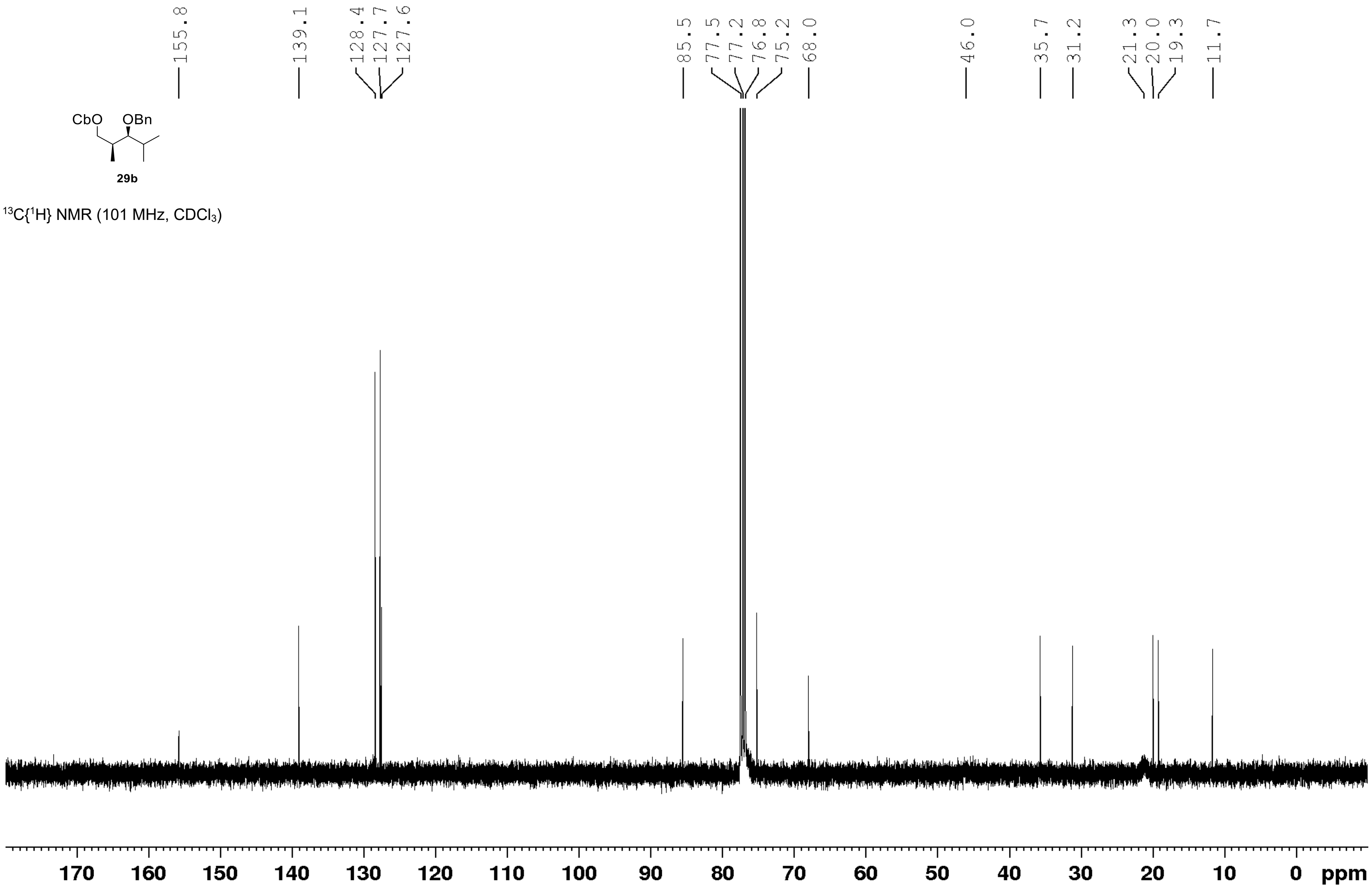

$^{13}C\{^1H\}$ NMR (101 MHz, $CDCl_3$)

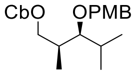

30b

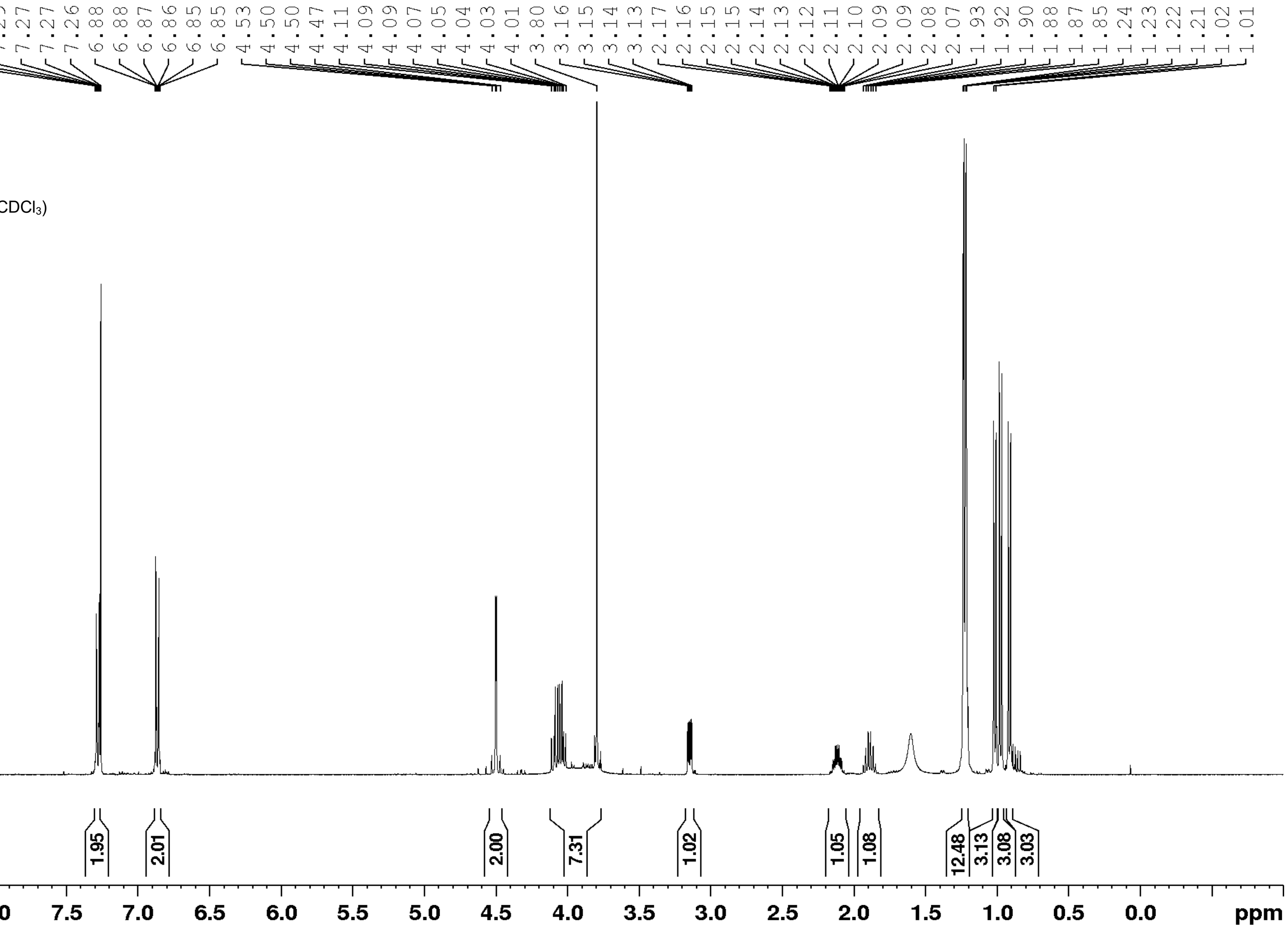

^{1}H NMR (400 MHz, CDCl$_3$)
7.30 7.29 7.29 7.27 7.27 7.26 6.88 6.88 6.87 6.86 6.85 6.85 4.53 4.50 4.50 4.47 4.11 4.09 4.09 4.07 4.05 4.04 4.03 4.01 3.80 3.16 3.15 3.14 3.13 2.17 2.16 2.15 2.15 2.14 2.13 2.12 2.11 2.10 2.09 2.09 2.08 2.07 1.93 1.92 1.90 1.88 1.87 1.85 1.24 1.23 1.22 1.21 1.02 1.01
1.95 2.01 2.00 7.31 1.02 1.05 1.08 12.48 3.13 3.08 3.03
8.5 8.0 7.5 7.0 6.5 6.0 5.5 5.0 4.5 4.0 3.5 3.0 2.5 2.0 1.5 1.0 0.5 0.0 ppm

30b

$^{13}C\{^{1}H\}$ NMR (101 MHz, $CDCl_3$)

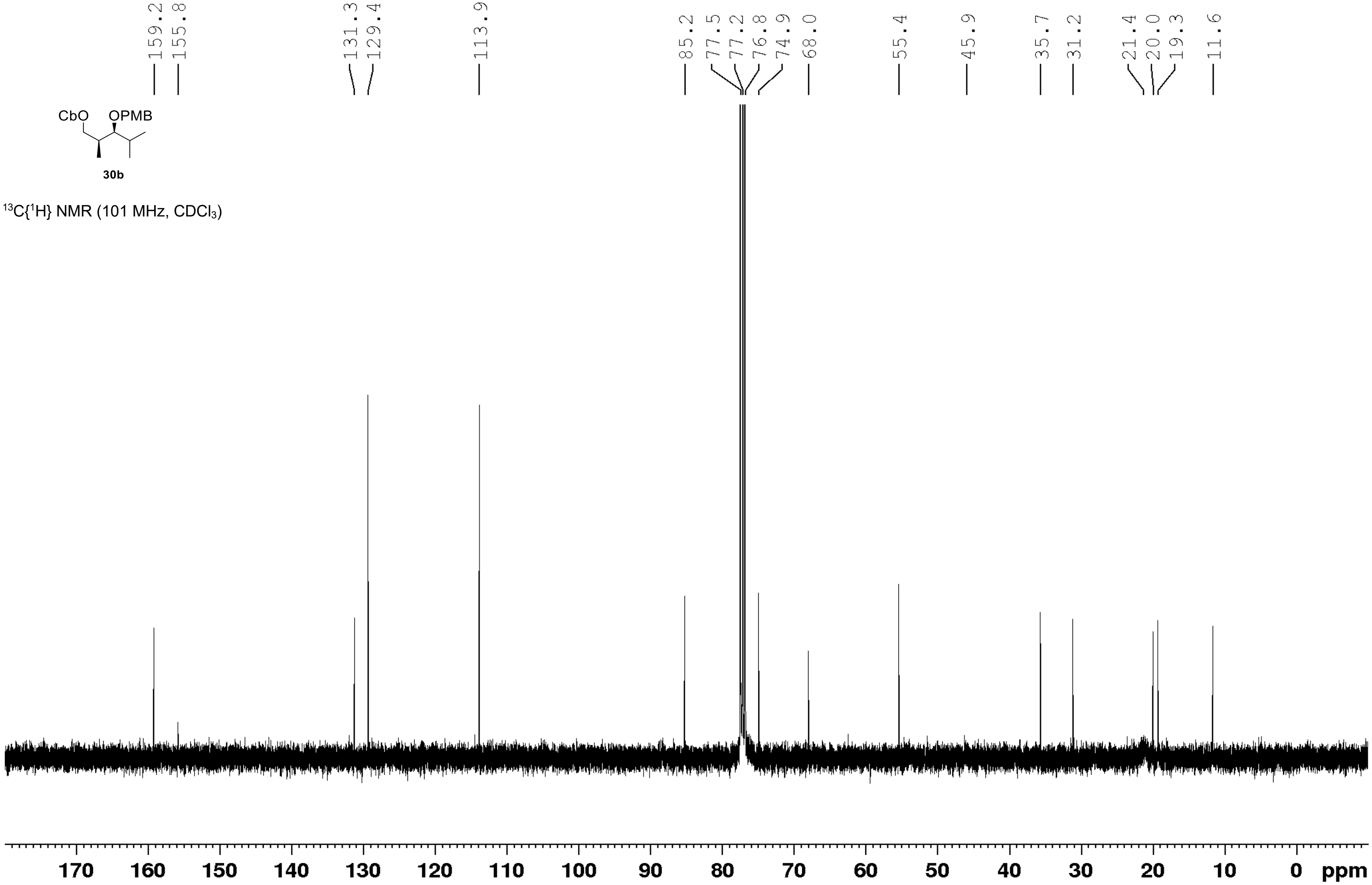

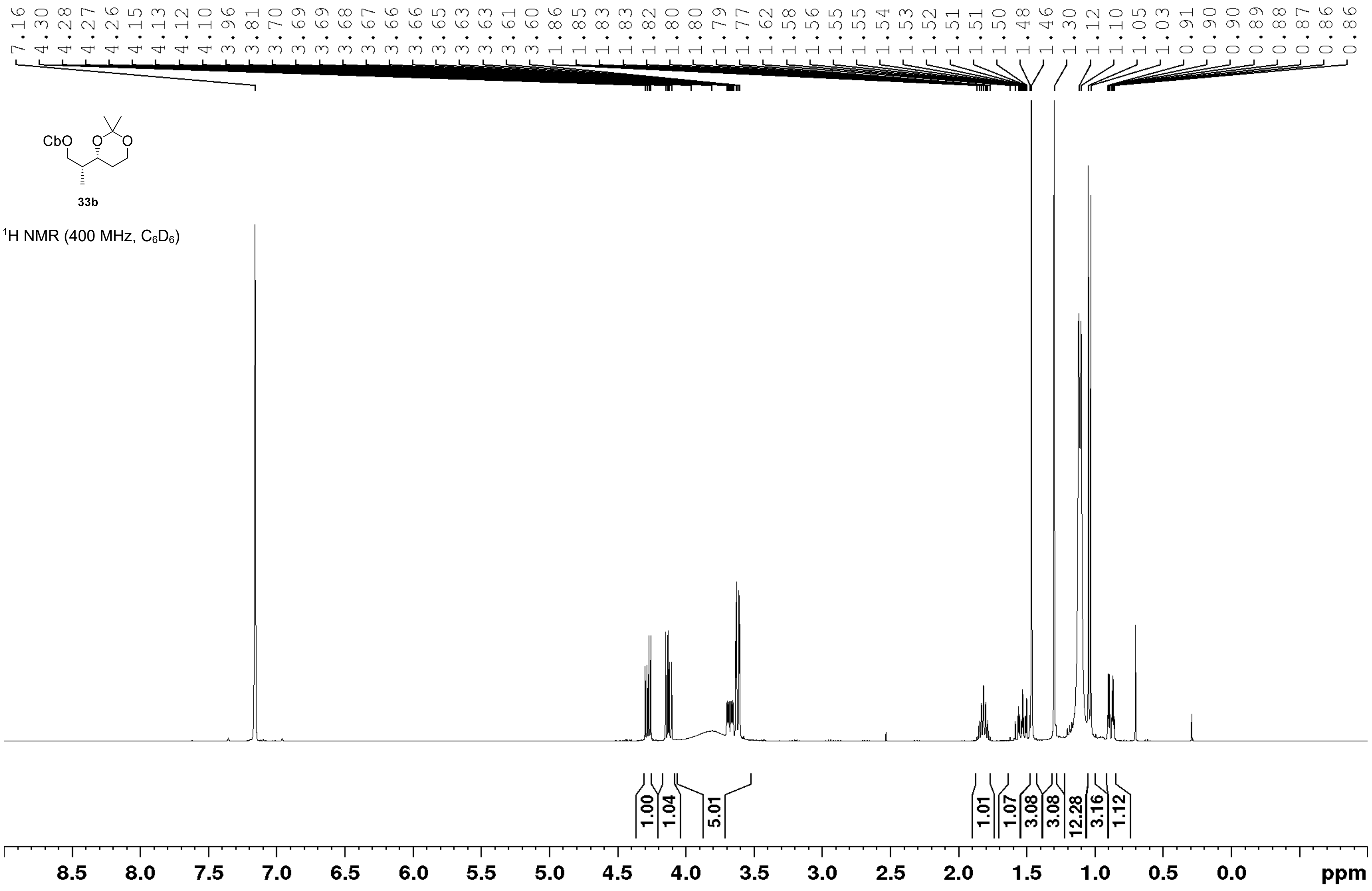
CbO
33b
1H NMR (400 MHz, C6D6)
7.16 4.30 4.28 4.27 4.26 4.15 4.13 4.12 4.10 3.96 3.81 3.70 3.69 3.69 3.68 3.67 3.66 3.66 3.65 3.63 3.63 3.61 3.60 1.86 1.85 1.83 1.83 1.82 1.80 1.80 1.79 1.77 1.62 1.58 1.56 1.55 1.55 1.54 1.53 1.52 1.51 1.51 1.50 1.48 1.46 1.30 1.12 1.10 1.05 1.03 0.91 0.90 0.90 0.89 0.88 0.87 0.86 0.86
1.00 1.04 5.01 1.01 1.07 3.08 3.08 12.28 3.16 1.12
8.5 8.0 7.5 7.0 6.5 6.0 5.5 5.0 4.5 4.0 3.5 3.0 2.5 2.0 1.5 1.0 0.5 0.0 ppm

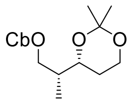

33b

$^{13}C\{^{1}H\}$ NMR (101 MHz, C_6D_6)

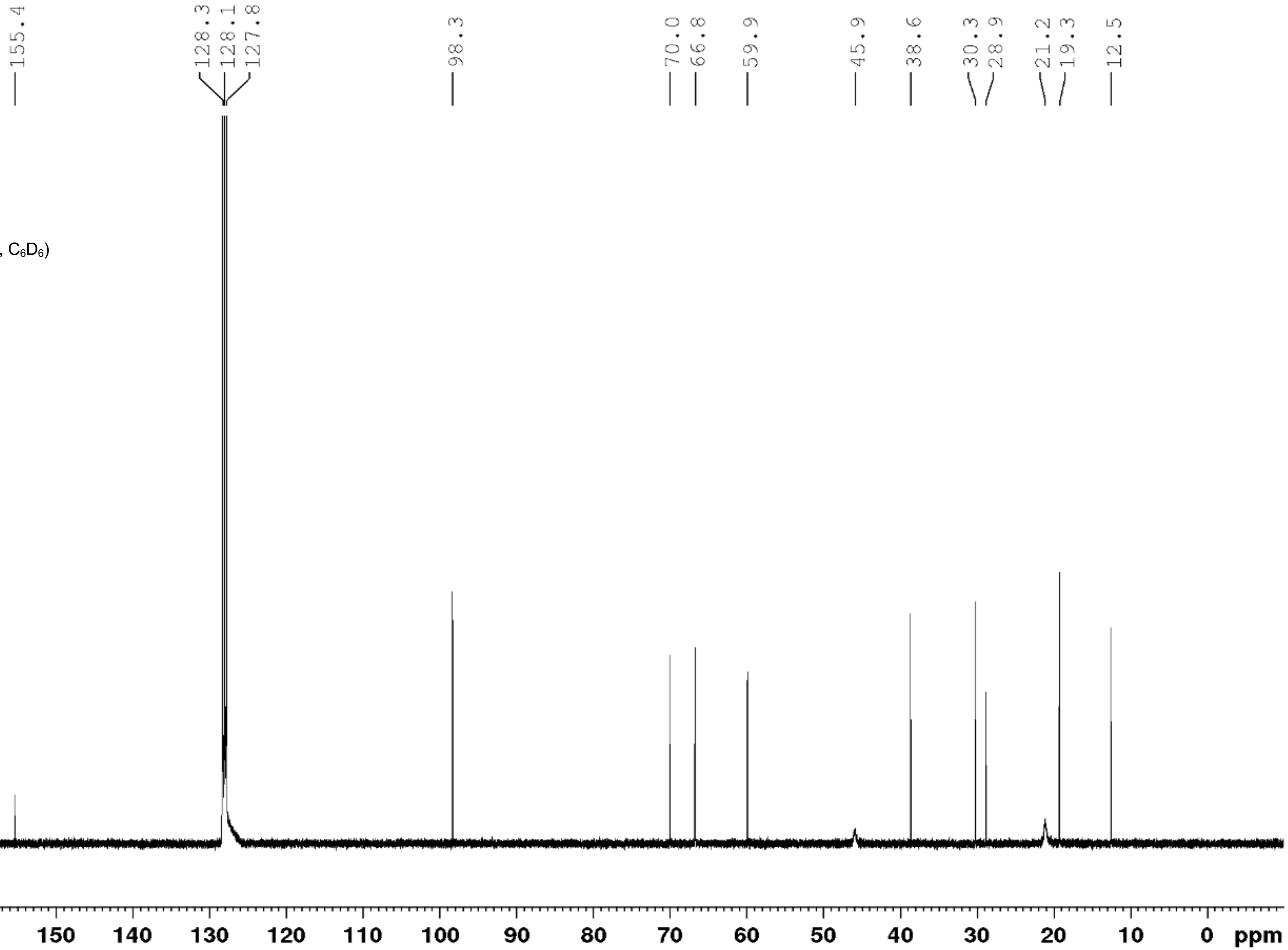

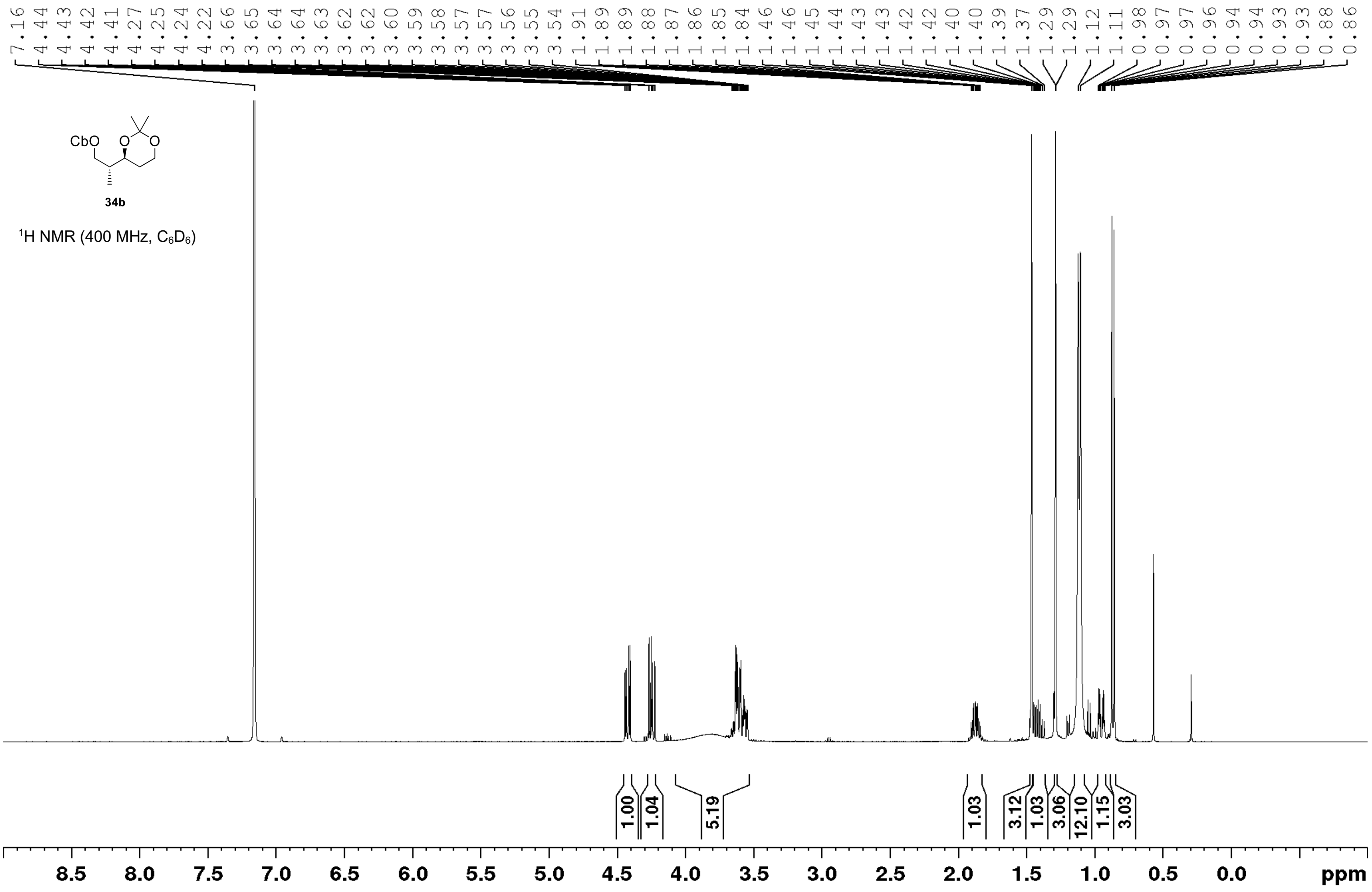
7.16
4.44
4.43
4.42
4.41
4.27
4.25
4.24
4.22
3.66
3.65
3.64
3.64
3.63
3.62
3.62
3.60
3.59
3.58
3.57
3.57
3.56
3.55
3.54
1.91
1.89
1.88
1.87
1.86
1.85
1.84
1.46
1.46
1.45
1.44
1.43
1.43
1.42
1.42
1.40
1.40
1.39
1.37
1.29
1.29
1.12
1.11
0.98
0.97
0.97
0.96
0.94
0.94
0.93
0.93
0.88
0.86
CbO
O
O
34b
^{1}H NMR (400 MHz, C_6D_6)
1.00
1.04
5.19
1.03
3.12
1.03
3.06
12.10
1.15
3.03
8.5
8.0
7.5
7.0
6.5
6.0
5.5
5.0
4.5
4.0
3.5
3.0
2.5
2.0
1.5
1.0
0.5
0.0
ppm

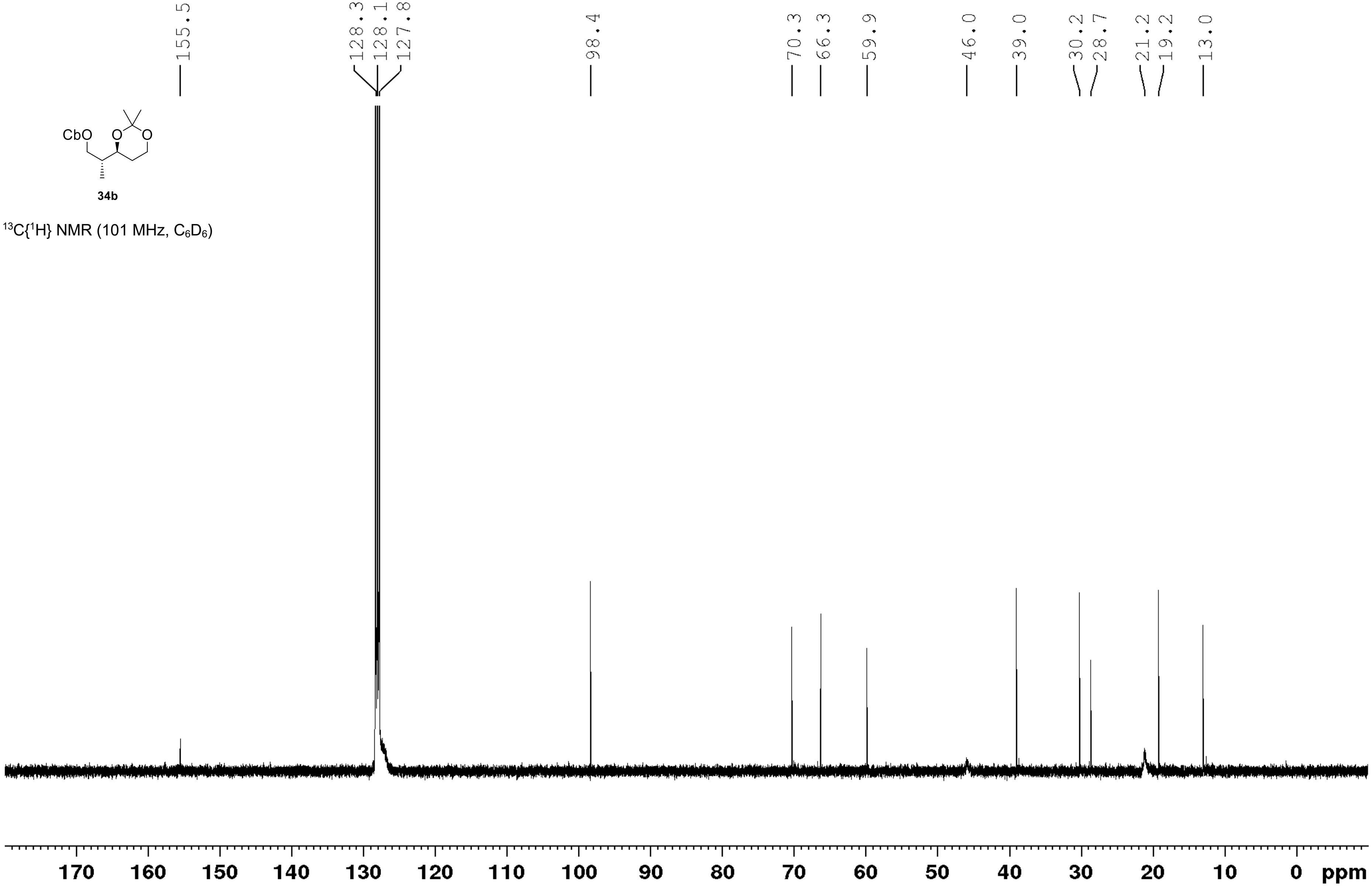

34b

$^{13}C\{^1H\}$ NMR (101 MHz, C_6D_6)

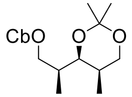

43b

^{1}H NMR (400 MHz, C_6D_6)

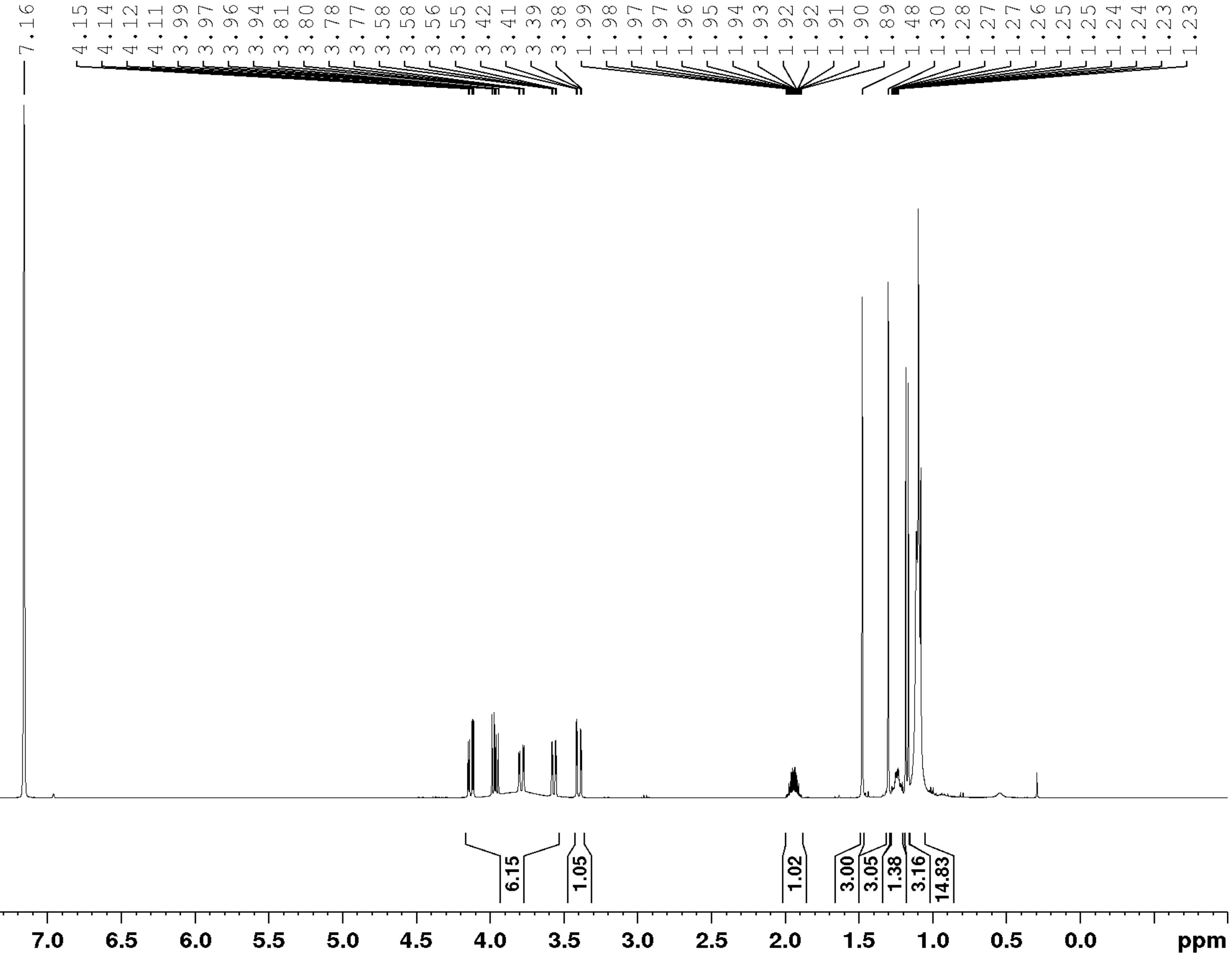

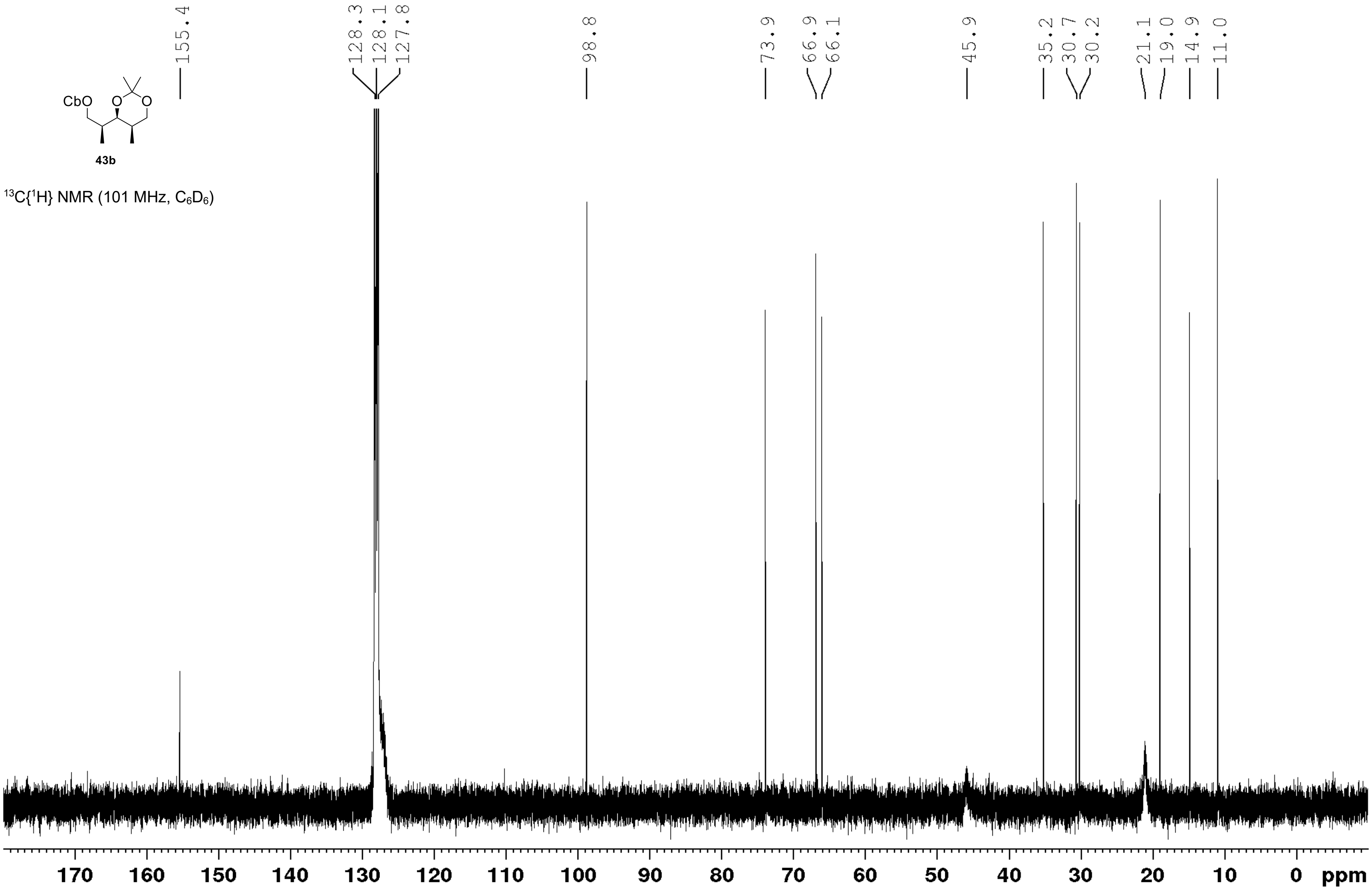

CbO
O
O
43b
$^{13}C\{^1H\}$ NMR (101 MHz, C_6D_6)
155.4
128.3
128.1
127.8
98.8
73.9
66.9
66.1
45.9
35.2
30.7
30.2
21.1
19.0
14.9
11.0
170 160 150 140 130 120 110 100 90 80 70 60 50 40 30 20 10 0 ppm

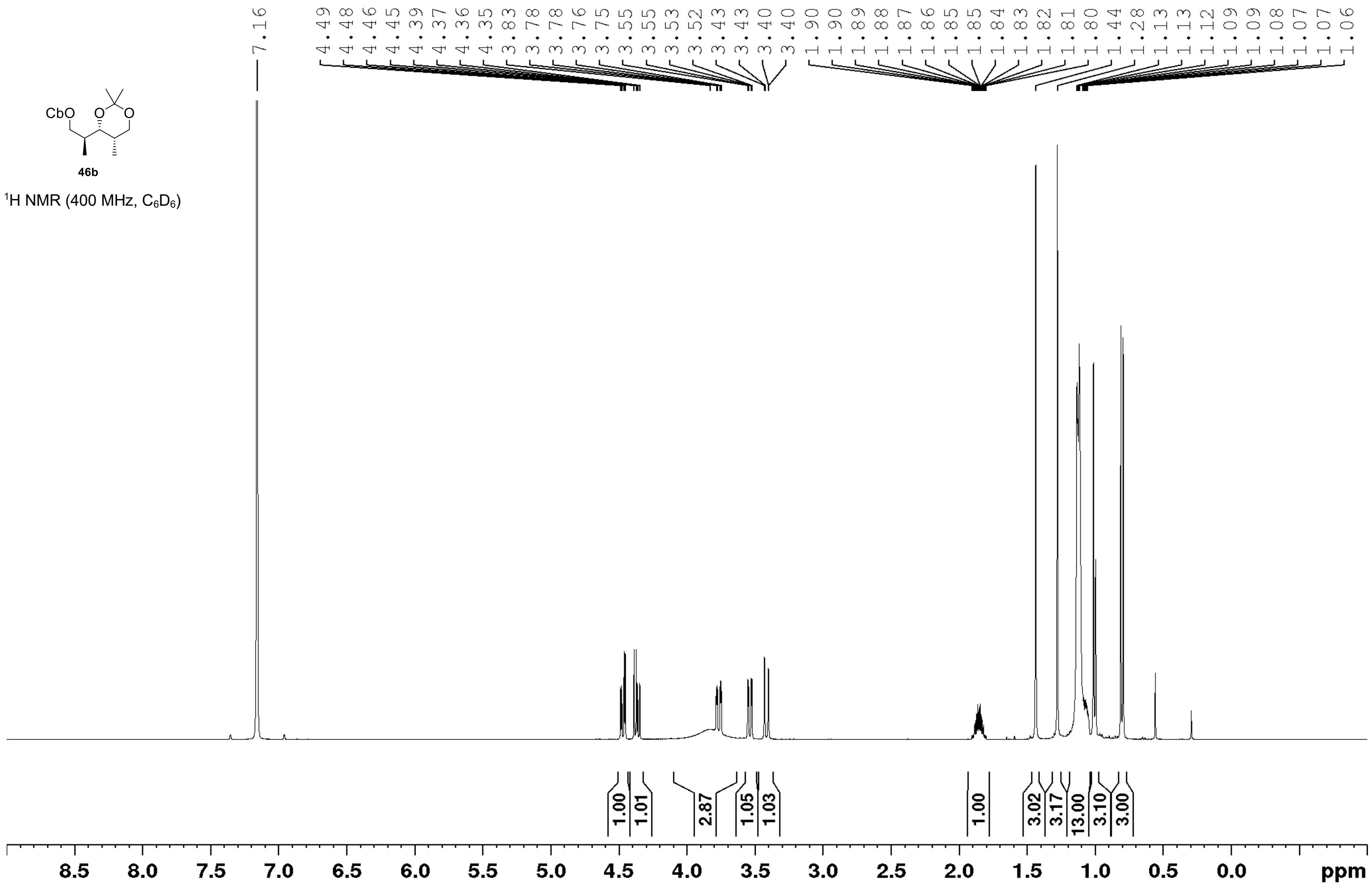
CbO
46b
1H NMR (400 MHz, C6D6)
7.16
4.49 4.48 4.46 4.45 4.39 4.37 4.36 4.35 3.83 3.78 3.78 3.76 3.75 3.55 3.55 3.53 3.52 3.43 3.43 3.40 3.40 1.90 1.90 1.89 1.88 1.87 1.86 1.85 1.85 1.84 1.83 1.82 1.81 1.80 1.44 1.28 1.13 1.13 1.12 1.09 1.09 1.08 1.07 1.07 1.06
1.00 1.01 2.87 1.05 1.03 1.00 3.02 3.17 13.00 3.10 3.00
8.5 8.0 7.5 7.0 6.5 6.0 5.5 5.0 4.5 4.0 3.5 3.0 2.5 2.0 1.5 1.0 0.5 0.0 ppm

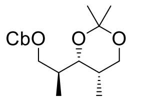

46b

$^{13}C\{^{1}H\}$ NMR (101 MHz, C_6D_6)

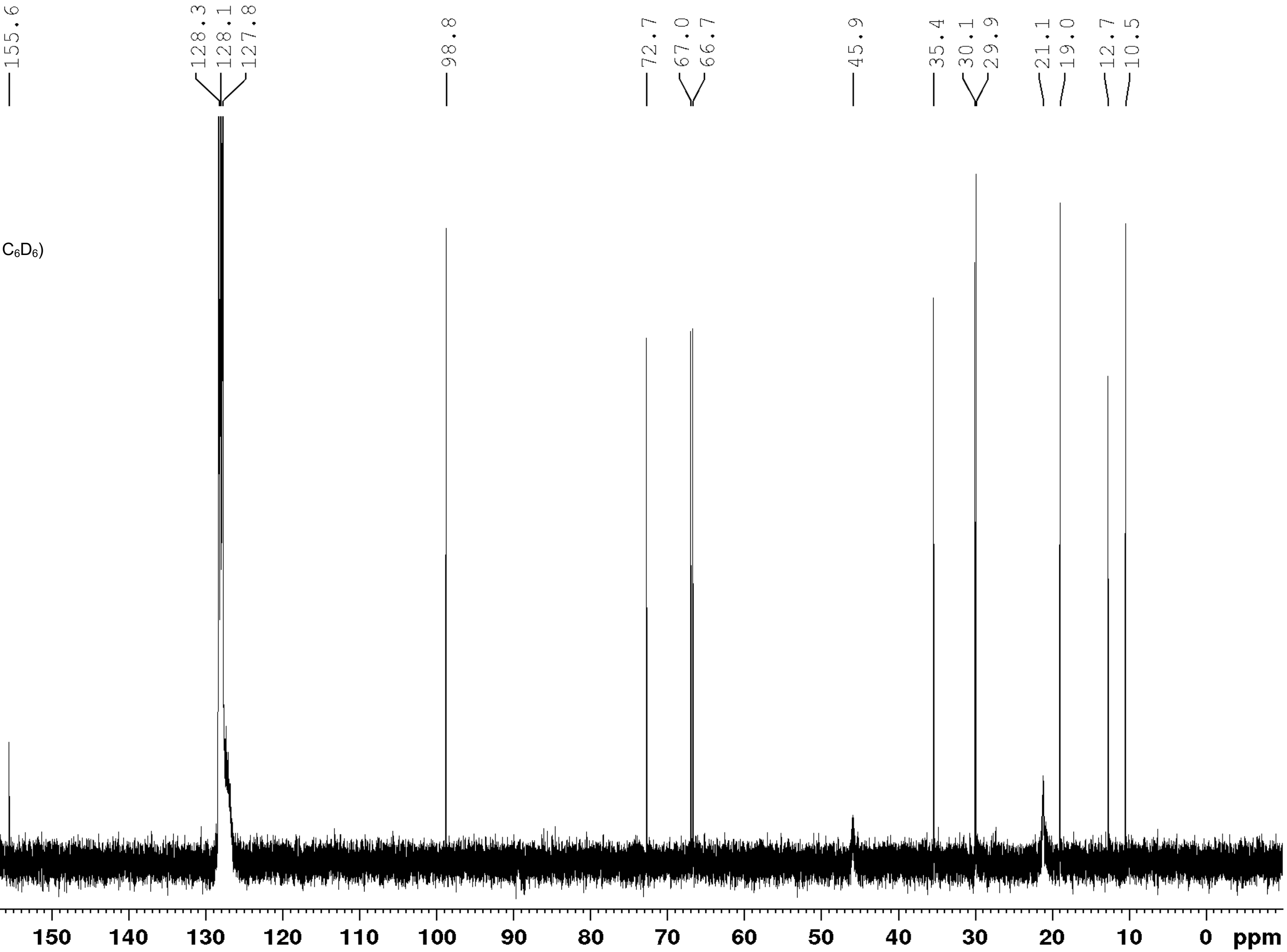

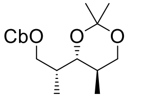

49b

^{1}H NMR (400 MHz, C_6D_6)

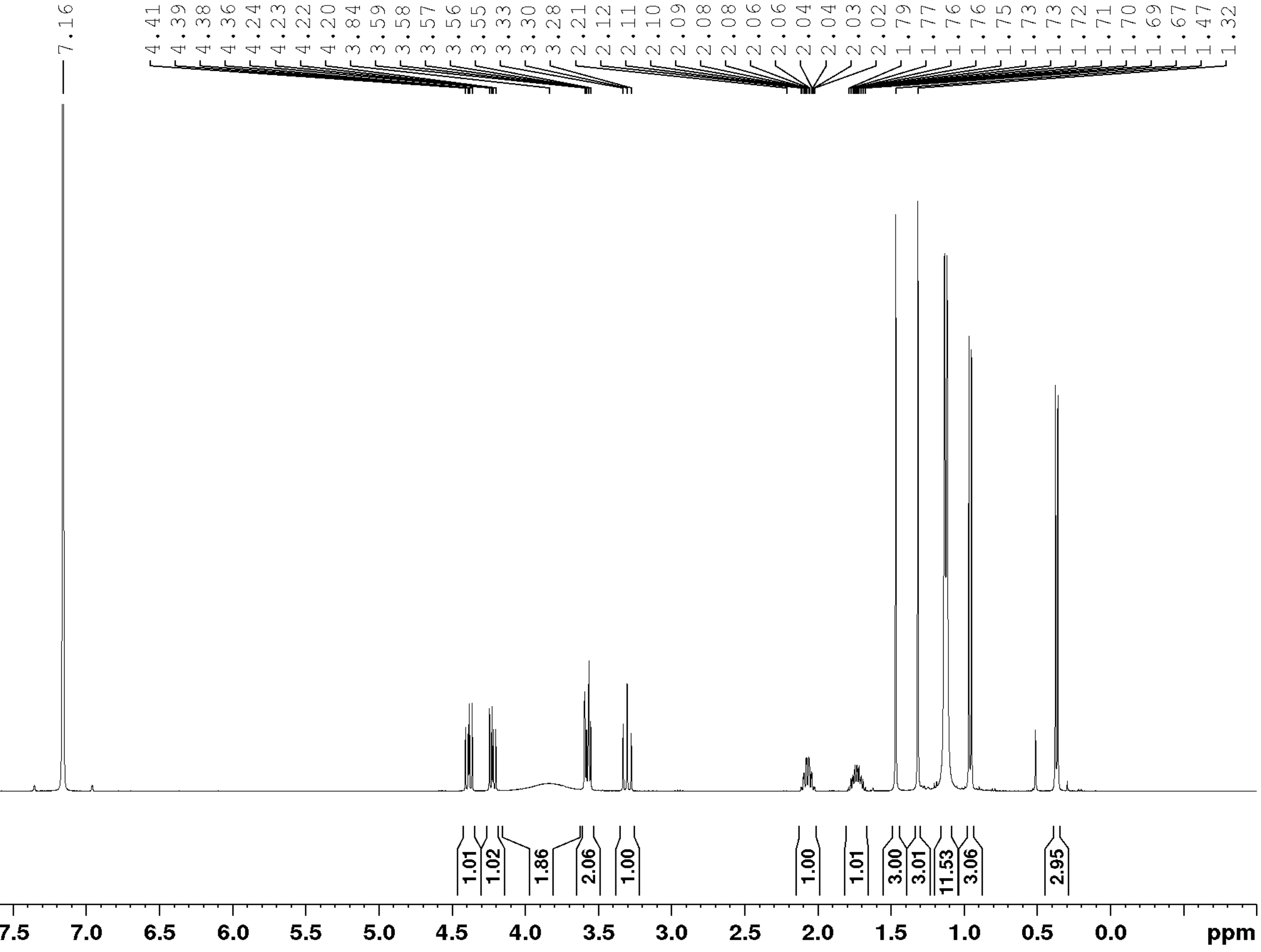

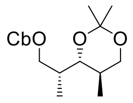

49b

$^{13}C\{^1H\}$ NMR (101 MHz, C_6D_6)

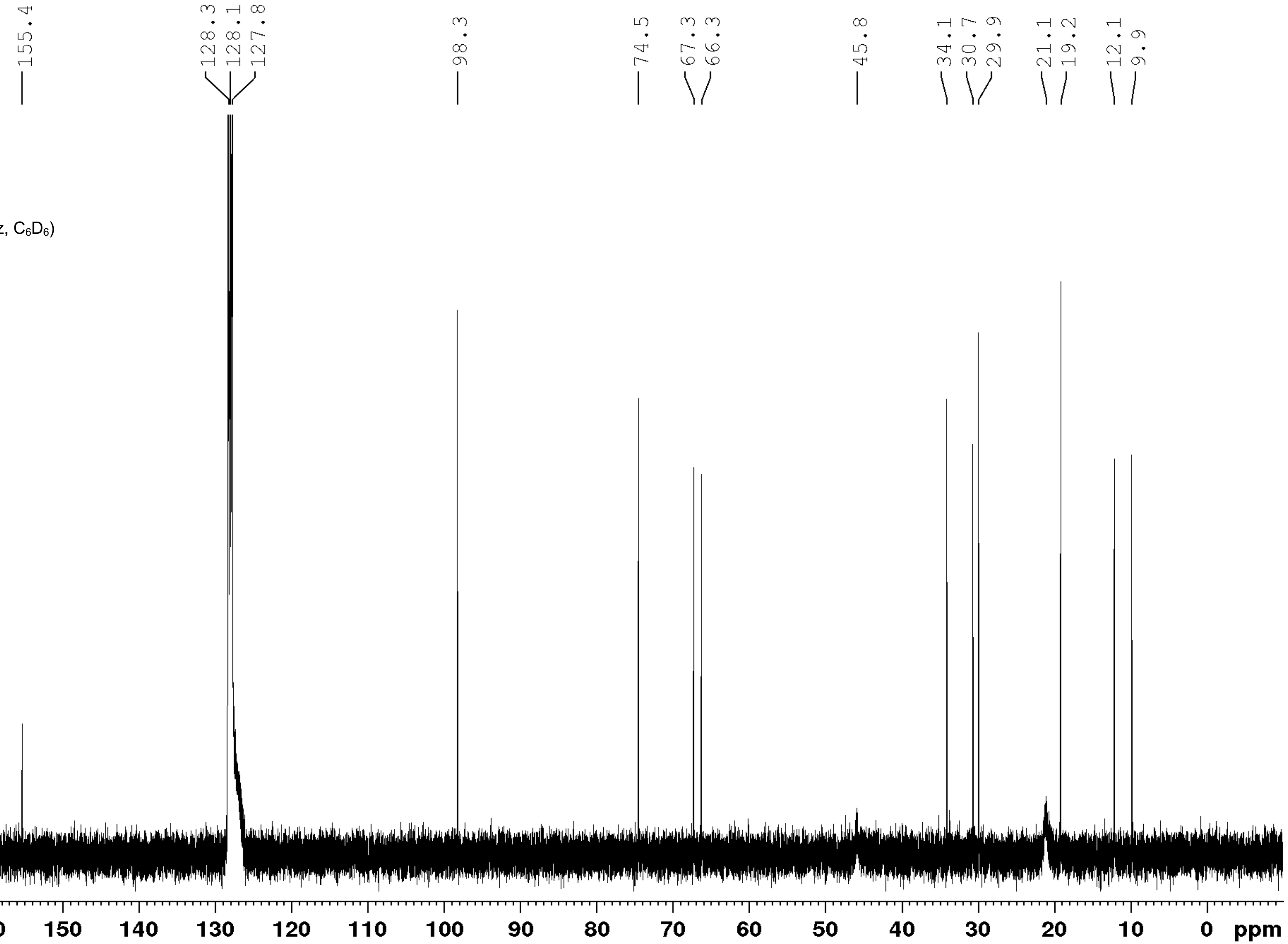

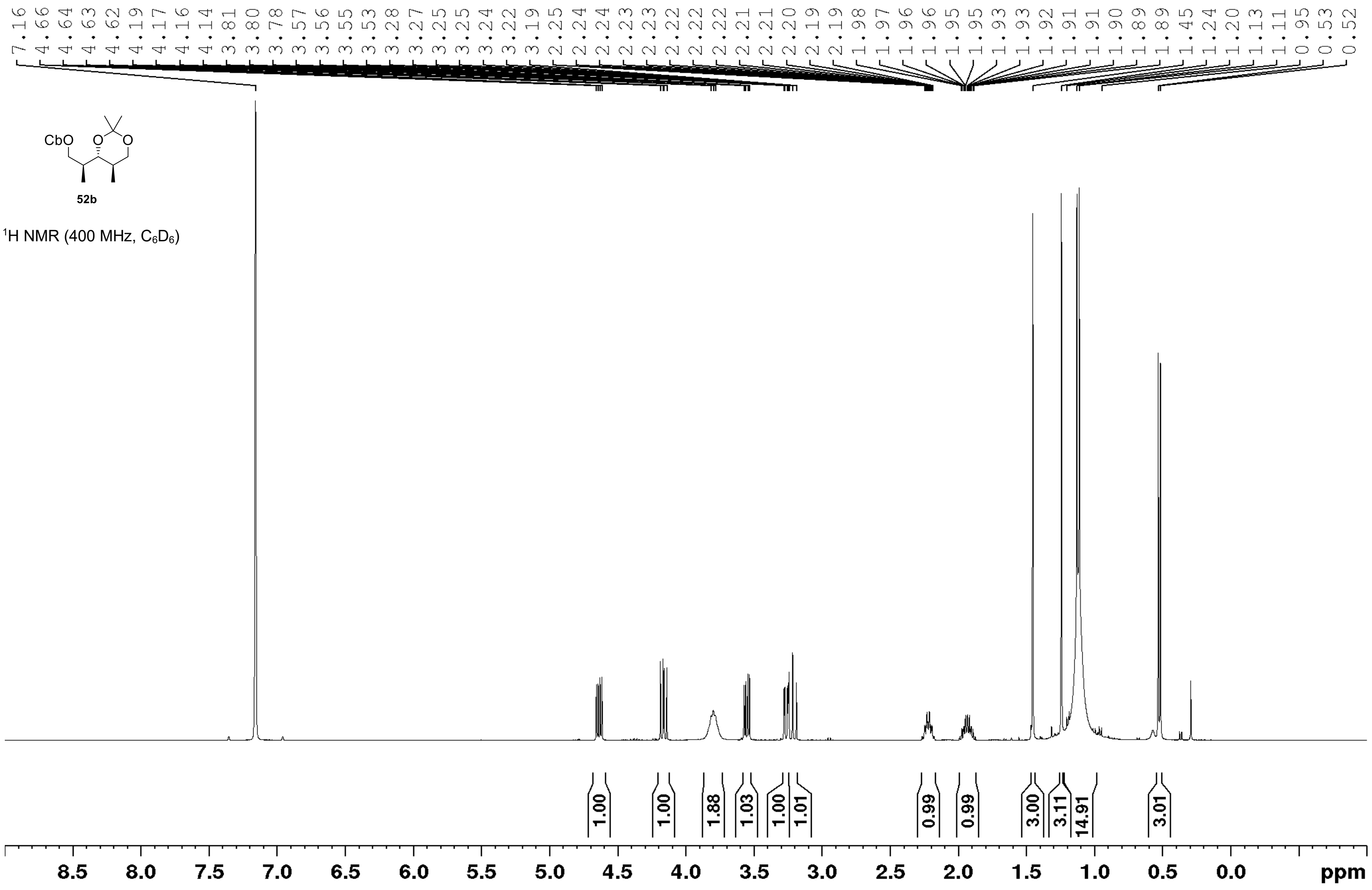
CbO
52b
¹H NMR (400 MHz, C₆D₆)
7.16 4.66 4.64 4.63 4.62 4.19 4.17 4.16 4.14 3.81 3.80 3.78 3.57 3.56 3.55 3.53 3.28 3.27 3.25 3.25 3.24 3.22 3.19 2.25 2.24 2.24 2.23 2.23 2.22 2.22 2.22 2.21 2.21 2.20 2.19 2.19 1.98 1.97 1.96 1.96 1.95 1.95 1.93 1.93 1.92 1.91 1.91 1.90 1.89 1.89 1.45 1.24 1.20 1.13 1.11 0.95 0.53 0.52
1.00 1.00 1.88 1.03 1.00 1.01 0.99 0.99 3.00 3.11 14.91 3.01
8.5 8.0 7.5 7.0 6.5 6.0 5.5 5.0 4.5 4.0 3.5 3.0 2.5 2.0 1.5 1.0 0.5 0.0 ppm

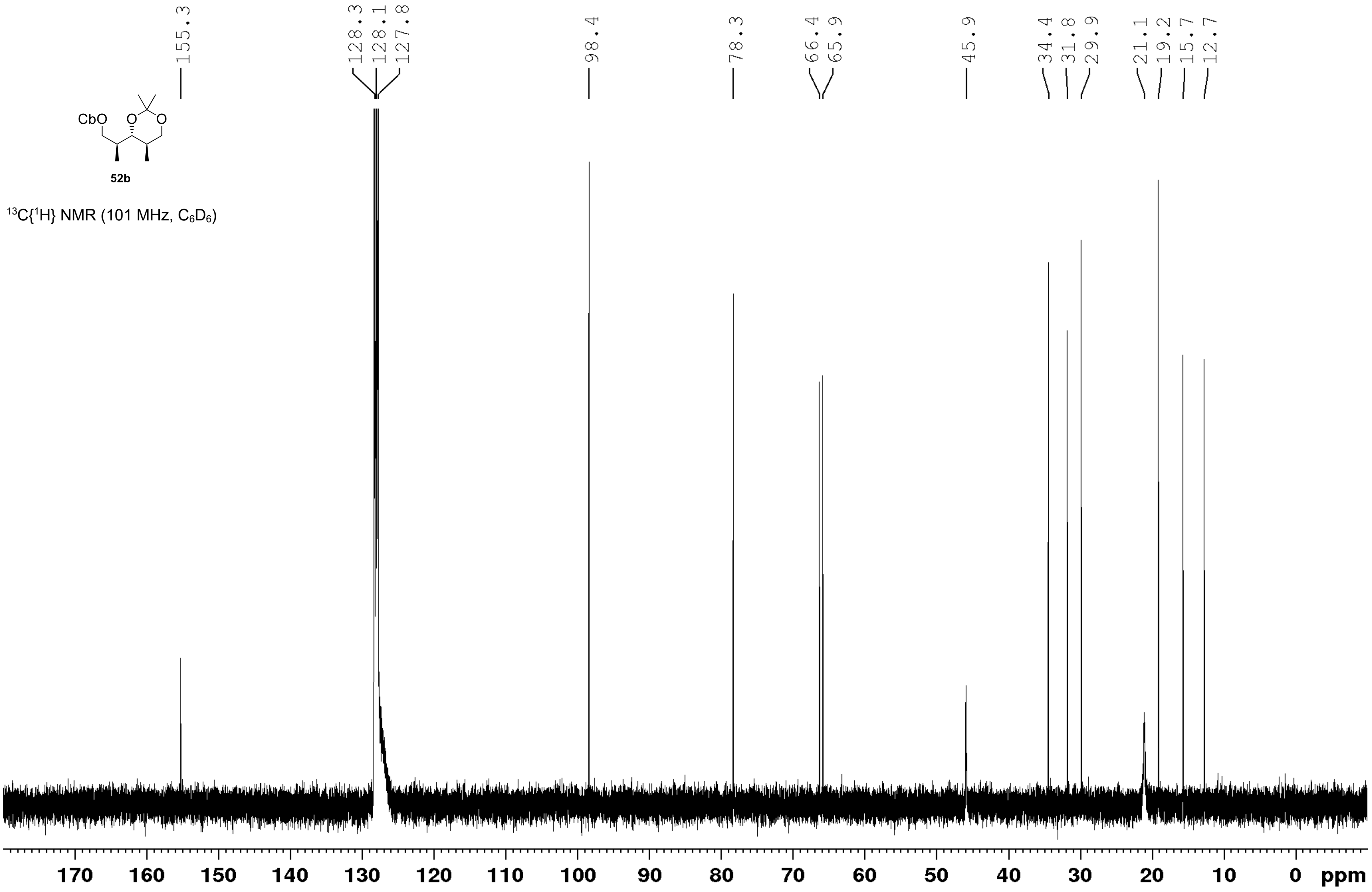
155.3
128.3
128.1
127.8
98.4
78.3
66.4
65.9
45.9
34.4
31.8
29.9
21.1
19.2
15.7
12.7
CbO
O
O
52b
$^{13}C\{^1H\}$ NMR (101 MHz, C_6D_6)
170
160
150
140
130
120
110
100
90
80
70
60
50
40
30
20
10
0
ppm

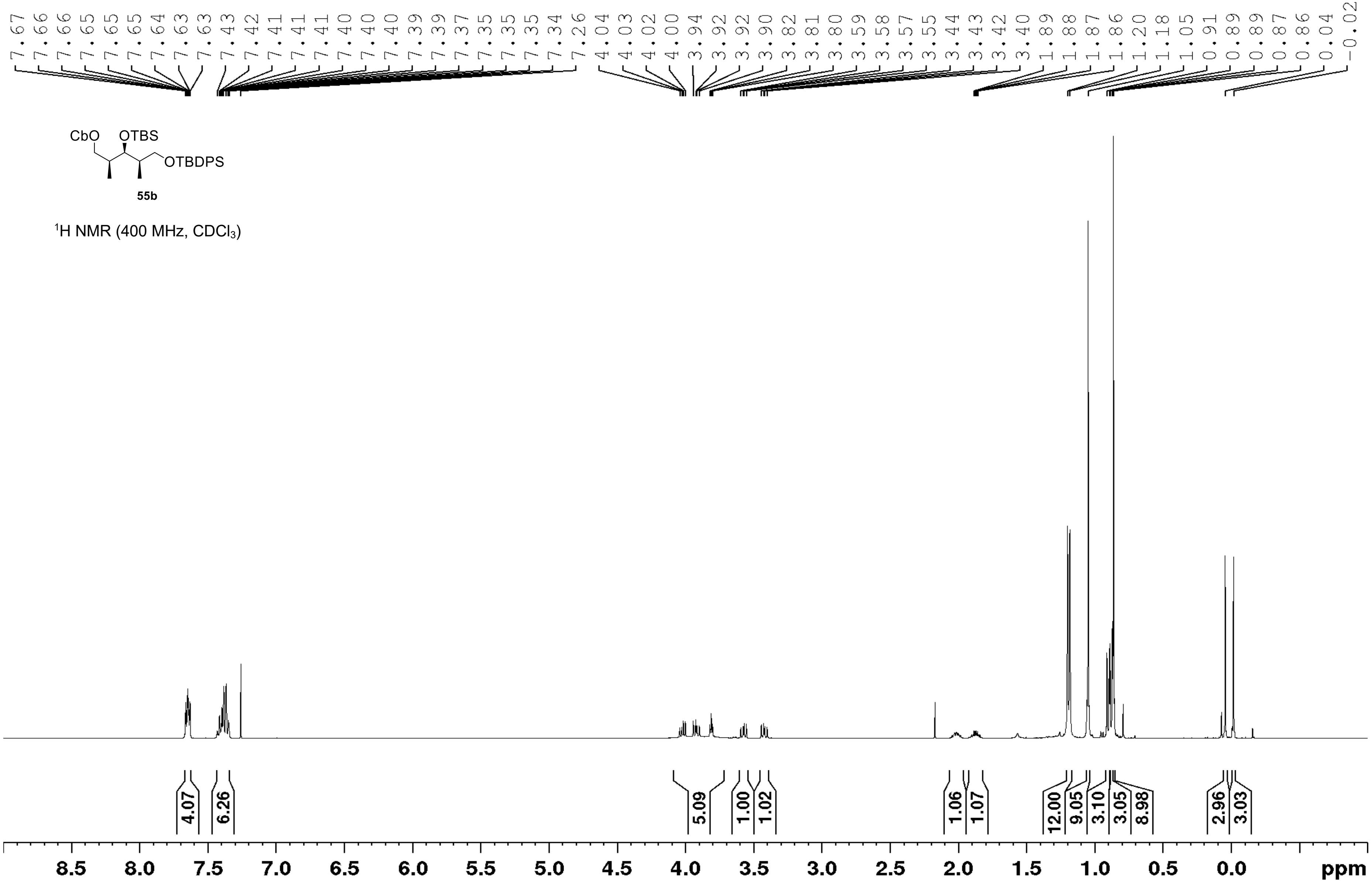

55b

^{1}H NMR (400 MHz, $CDCl_3$)

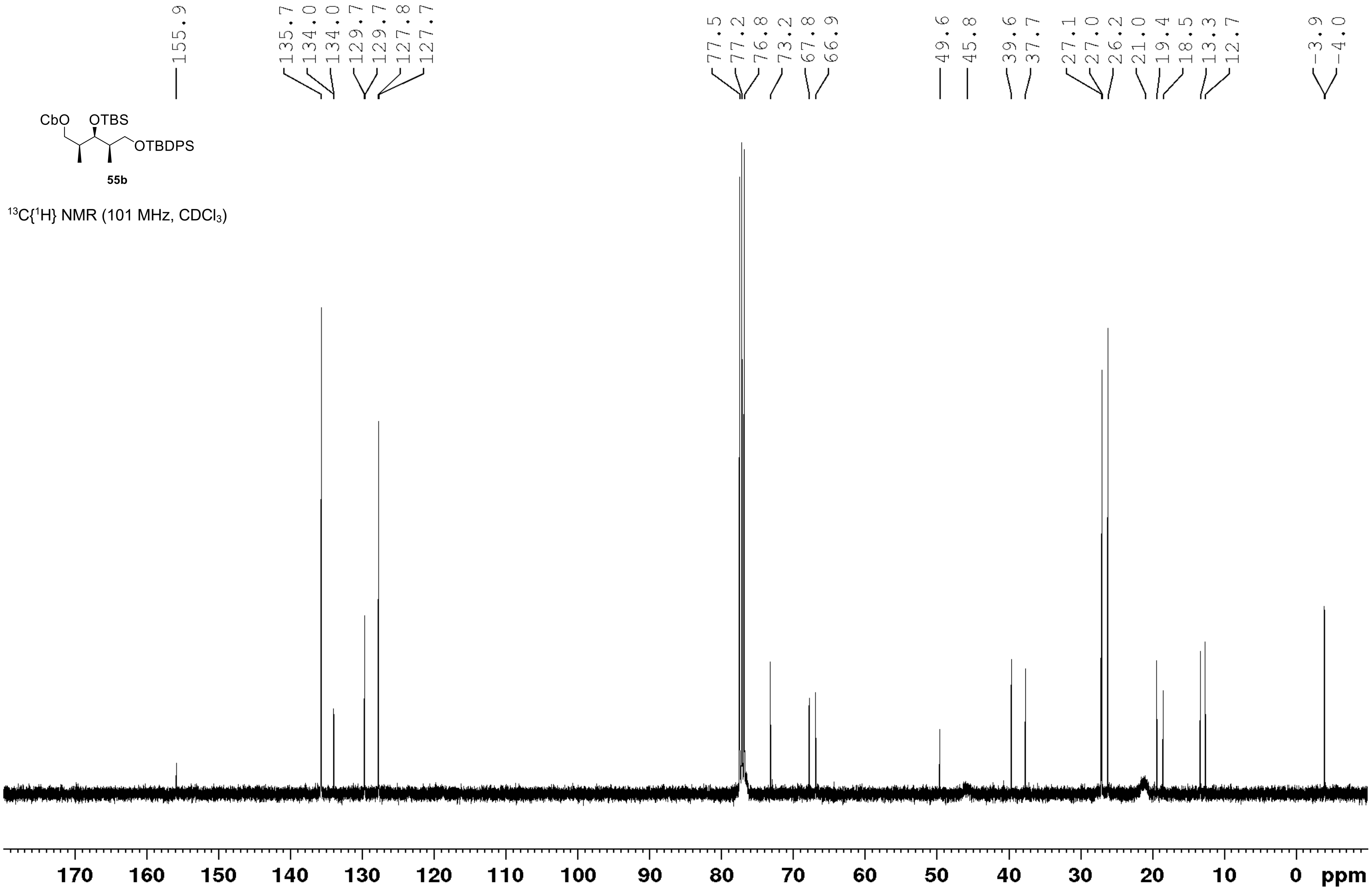

55b

$^{13}C\{^{1}H\}$ NMR (101 MHz, $CDCl_3$)

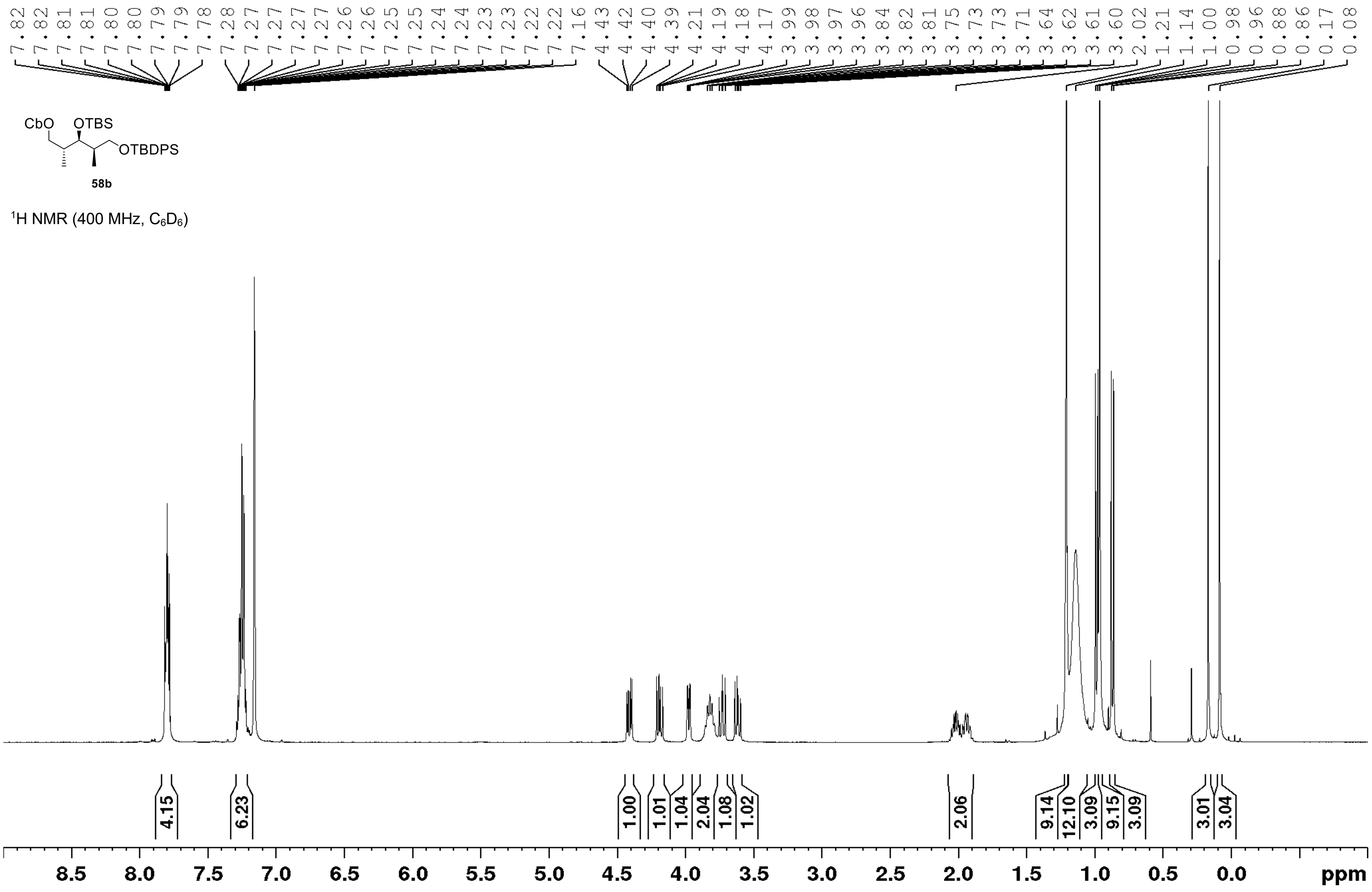

CbO
OTBS
OTBDPS
58b
¹H NMR (400 MHz, C₆D₆)
ppm

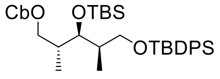

58b

$^{13}C\{^{1}H\}$ NMR (101 MHz, C_6D_6)

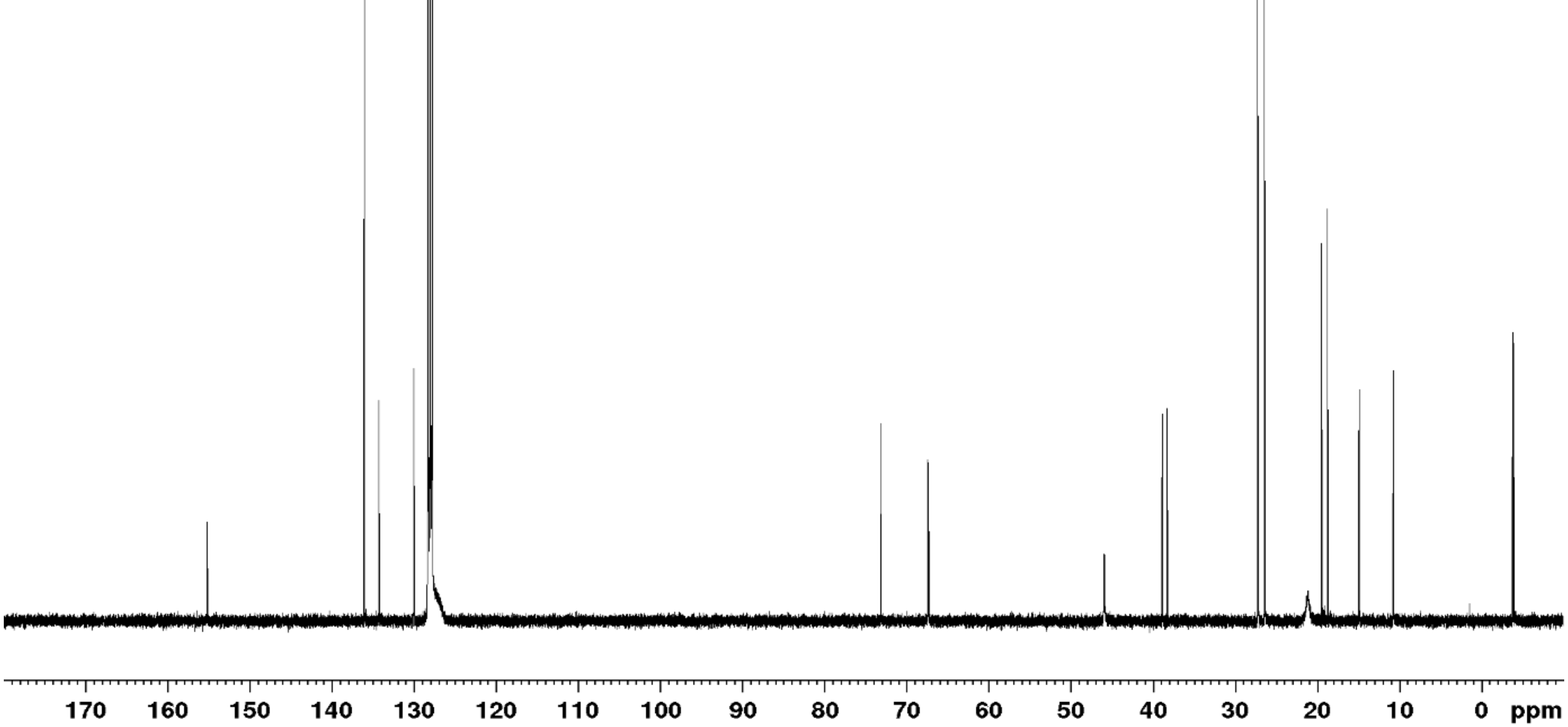

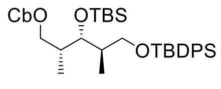

60b

^{1}H NMR (400 MHz, $CDCl_3$)

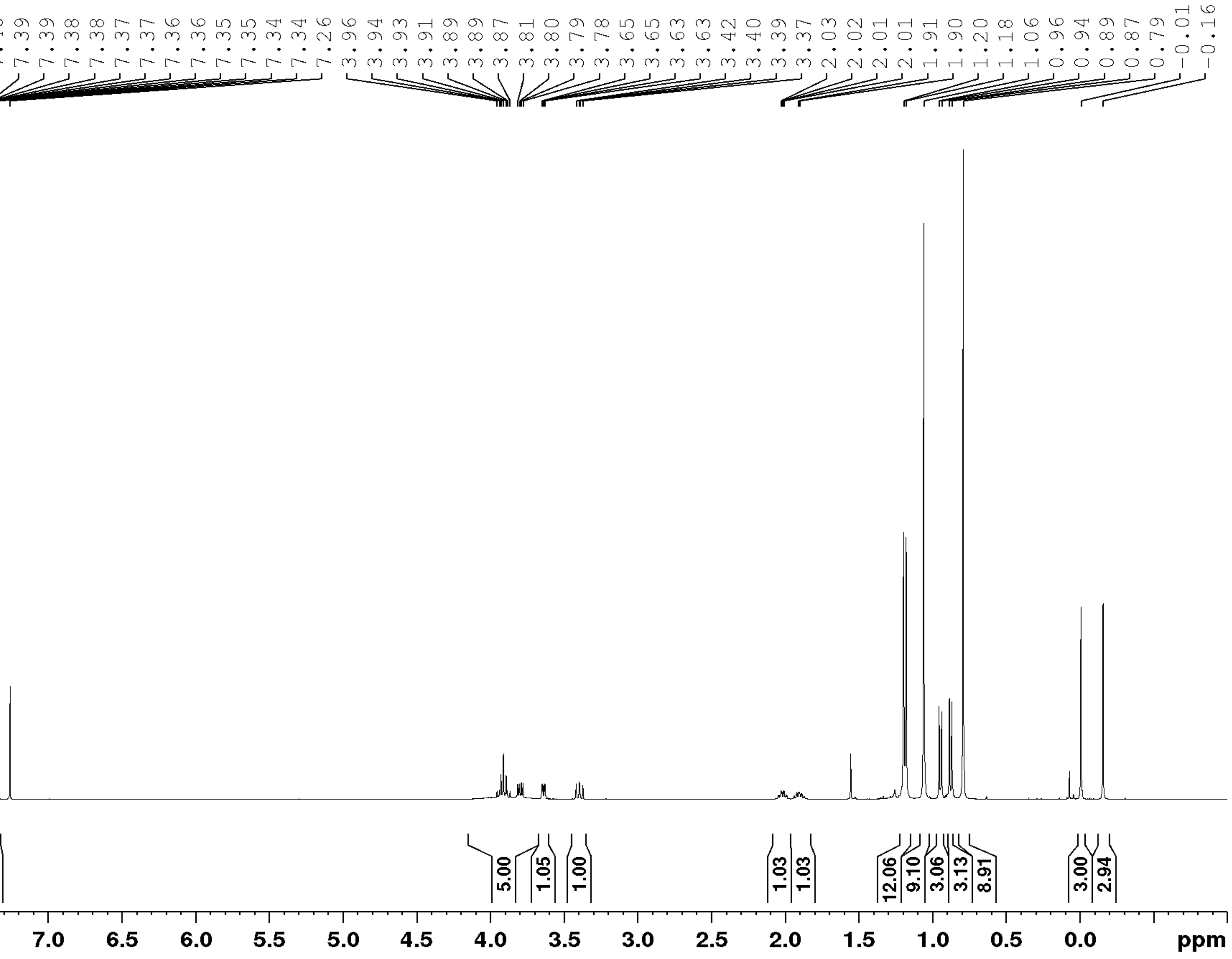

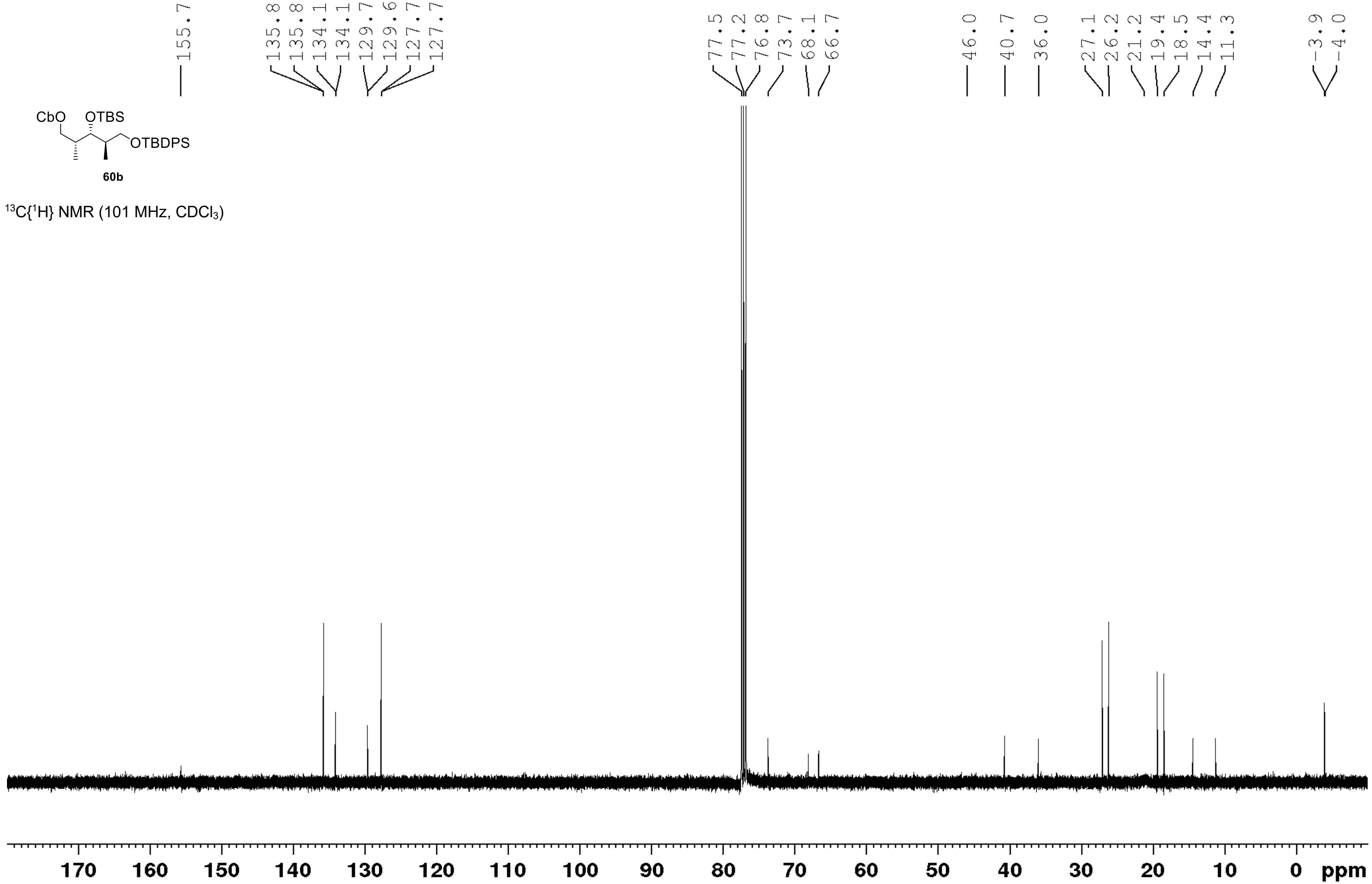

60b

$^{13}C\{^1H\}$ NMR (101 MHz, $CDCl_3$)

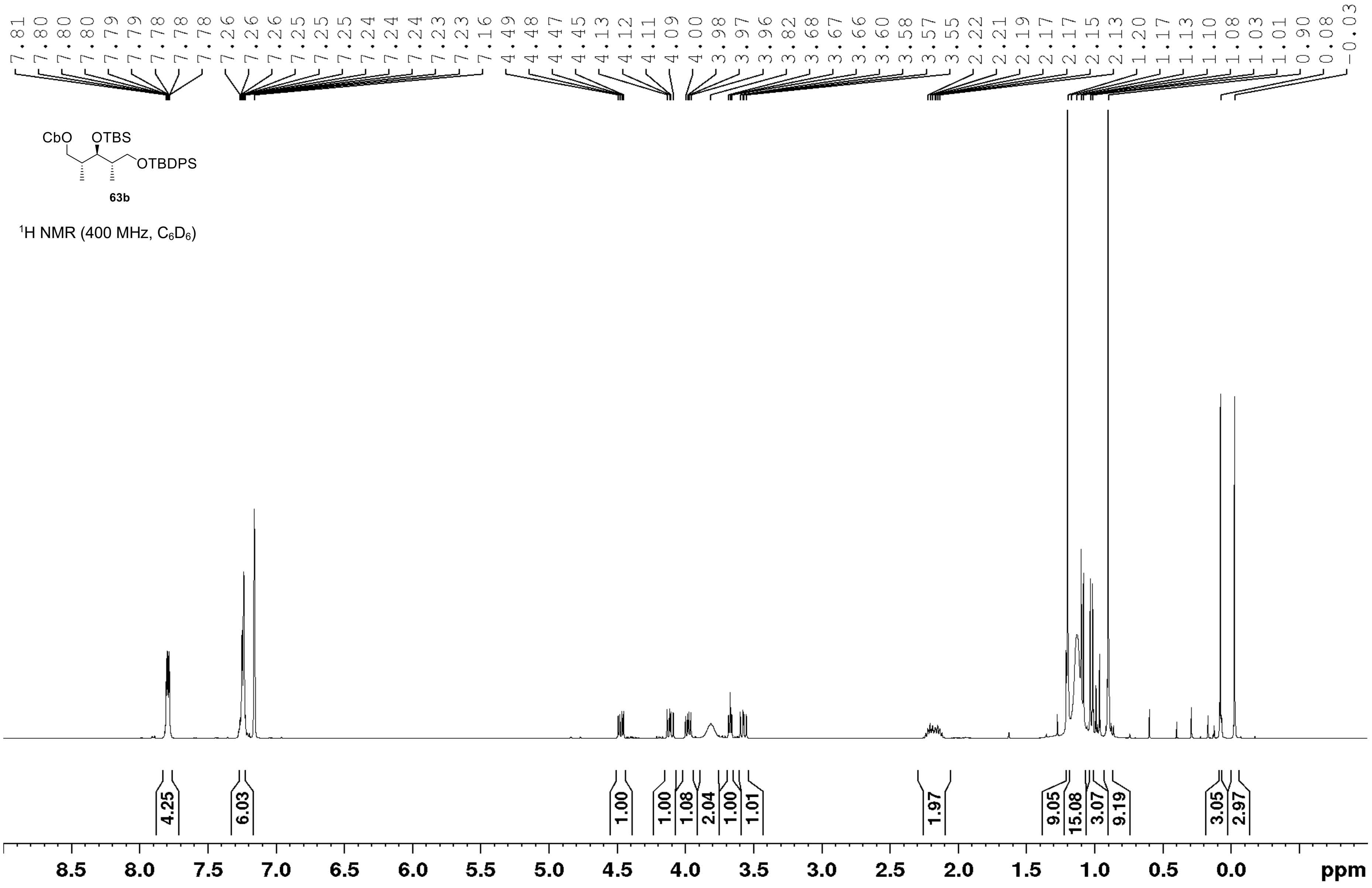
CbO
OTBS
OTBDPS
63b
^{1}H NMR (400 MHz, C6D6)
ppm

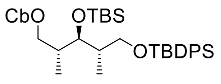

63b

$^{13}C\{^{1}H\}$ NMR (101 MHz, C_6D_6)

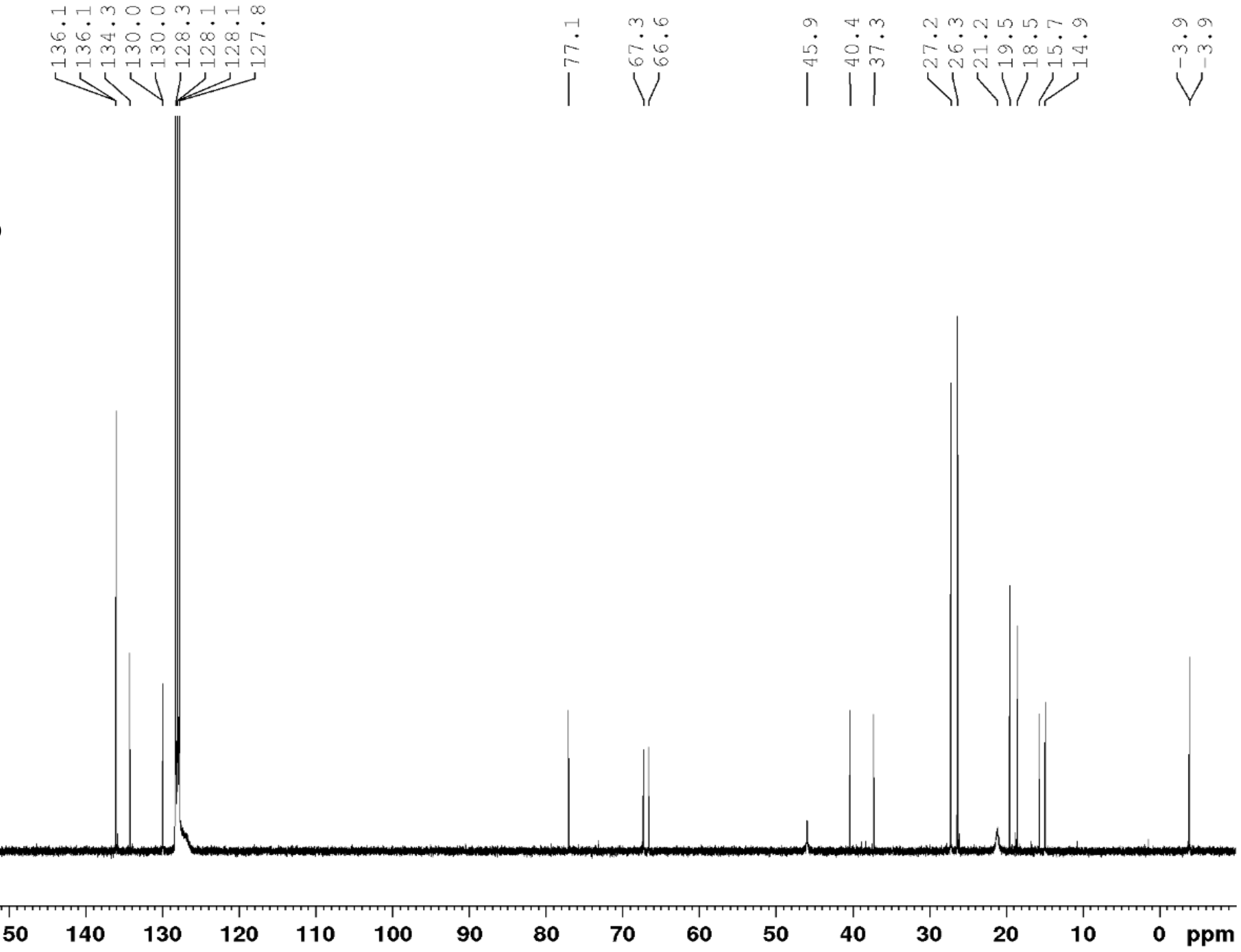

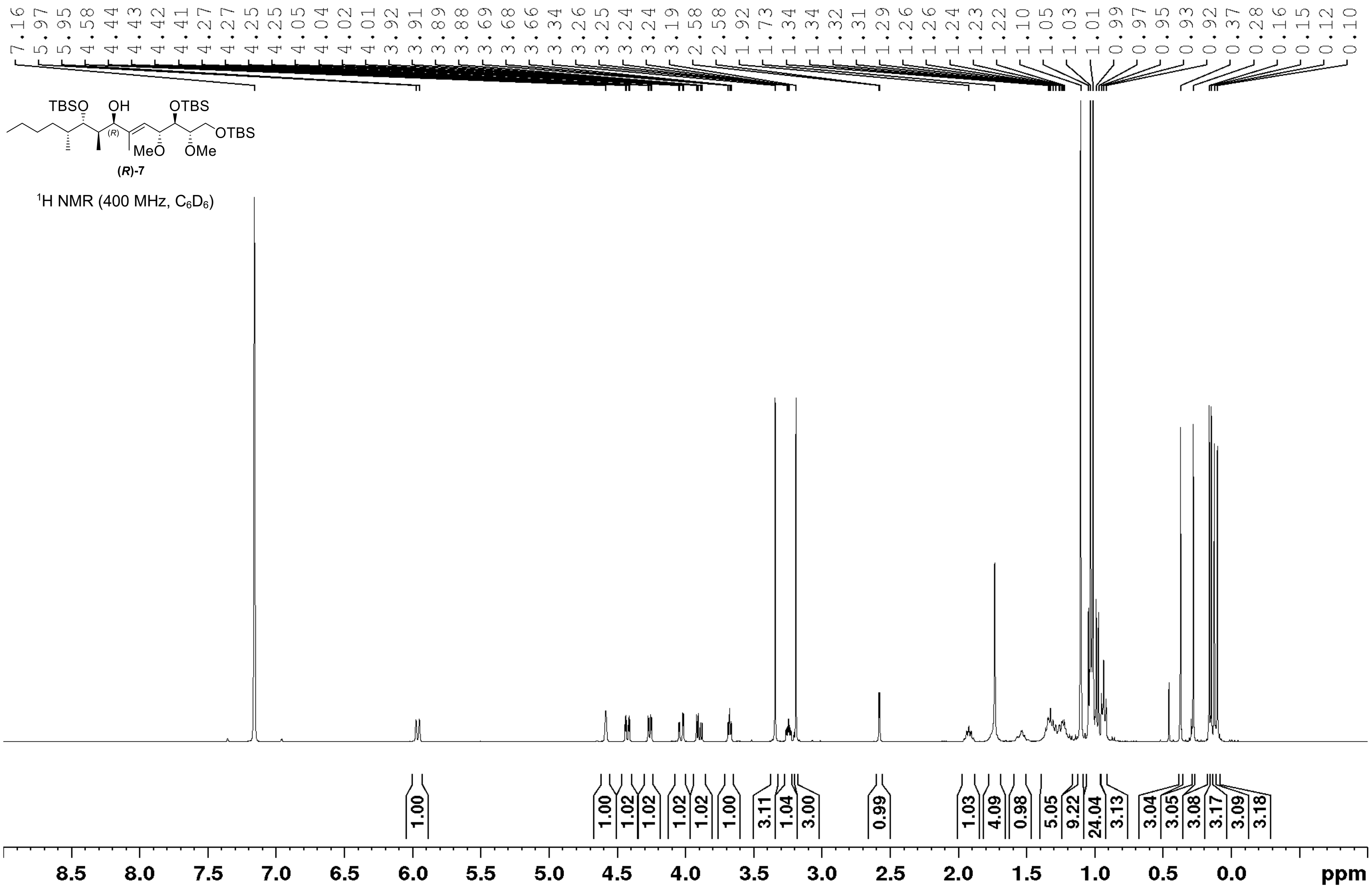

TBSO OH OTBS
OTBS
MeO OMe
(R)-7
^{1}H NMR (400 MHz, C6D6)
7.16 5.97 5.95 4.58 4.44 4.43 4.42 4.41 4.27 4.27 4.25 4.25 4.05 4.04 4.02 4.01 3.92 3.91 3.89 3.88 3.69 3.68 3.66 3.34 3.26 3.25 3.24 3.24 3.19 2.58 2.58 1.92 1.73 1.34 1.34 1.32 1.31 1.29 1.26 1.26 1.24 1.23 1.22 1.10 1.05 1.03 1.01 0.99 0.97 0.95 0.93 0.92 0.37 0.28 0.16 0.15 0.12 0.10
1.00 1.00 1.02 1.02 1.02 1.02 1.00 3.11 1.04 3.00 0.99 1.03 4.09 0.98 5.05 9.22 24.04 3.13 3.04 3.05 3.08 3.17 3.09 3.18
8.5 8.0 7.5 7.0 6.5 6.0 5.5 5.0 4.5 4.0 3.5 3.0 2.5 2.0 1.5 1.0 0.5 0.0 ppm

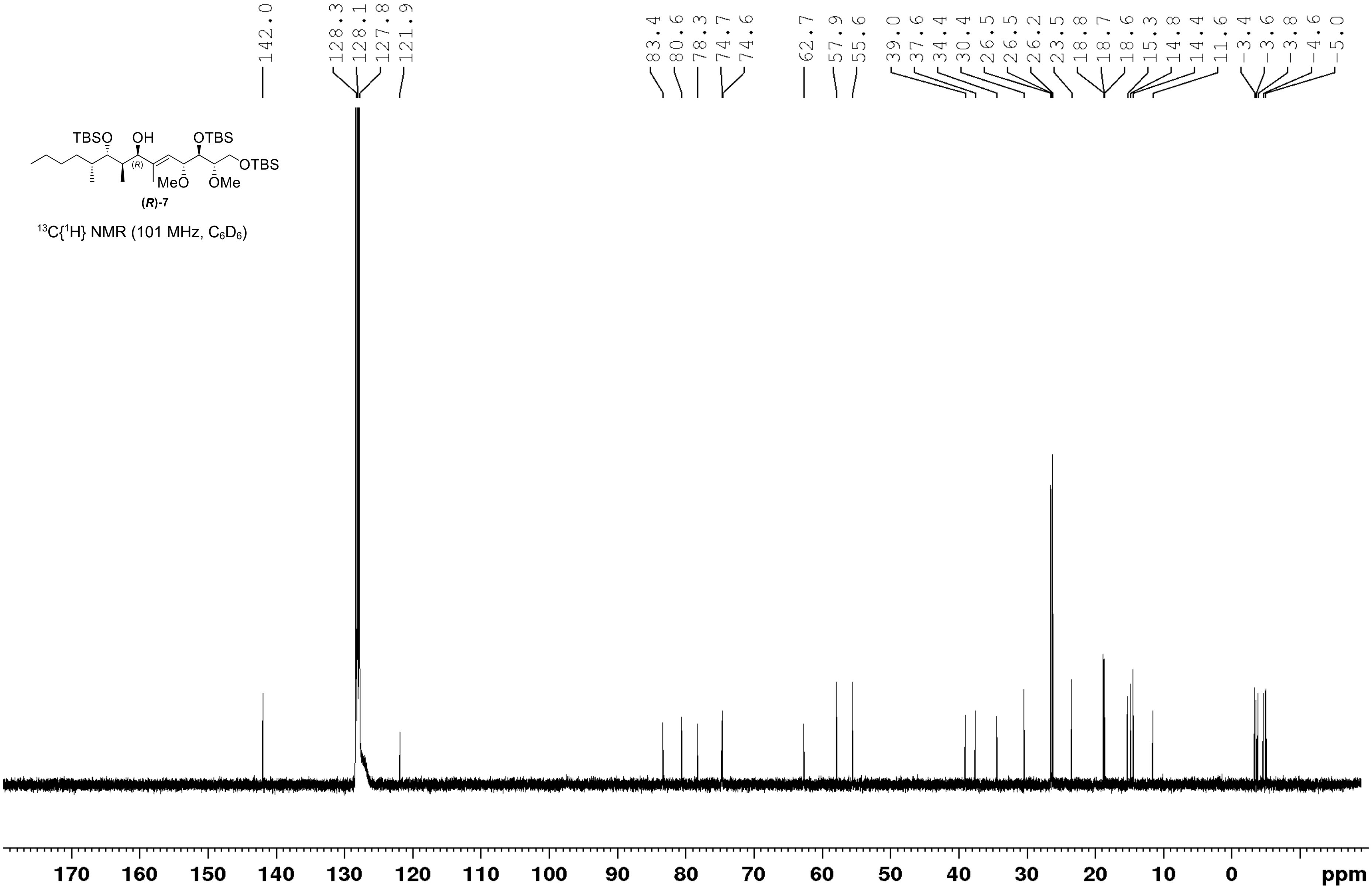
142.0
128.3
128.1
127.8
121.9
83.4
80.6
78.3
74.7
74.6
62.7
57.9
55.6
39.0
37.6
34.4
30.4
26.5
26.5
26.2
23.5
18.8
18.7
18.6
15.3
14.8
14.4
11.6
-3.4
-3.6
-3.8
-4.6
-5.0
TBSO
OH
OTBS
(R)
OTBS
MeO
OMe
(R)-7
$^{13}C\{^1H\}$ NMR (101 MHz, C_6D_6)
170
160
150
140
130
120
110
100
90
80
70
60
50
40
30
20
10
0
ppm

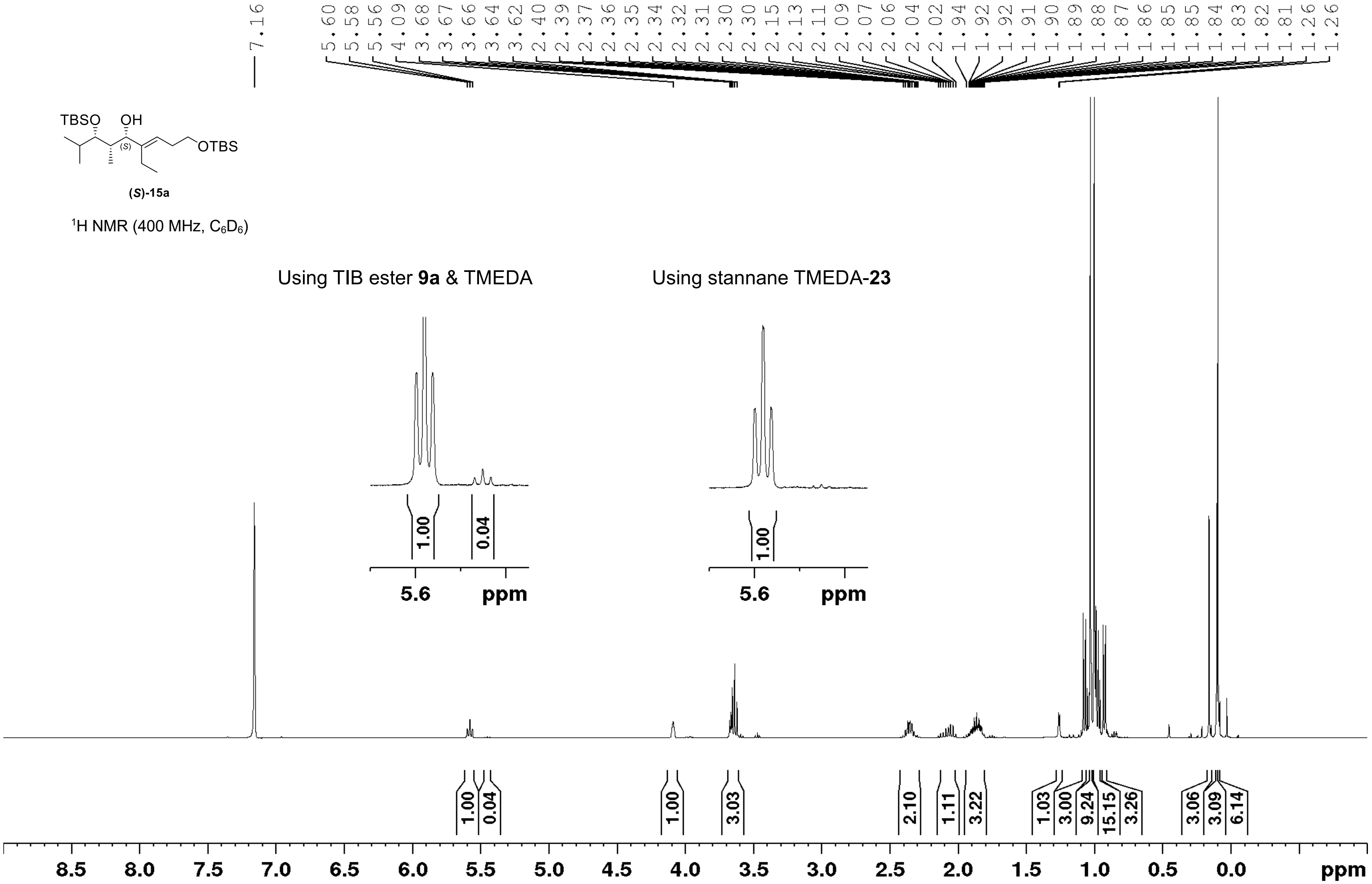

^{1}H NMR (400 MHz, C_6D_6)

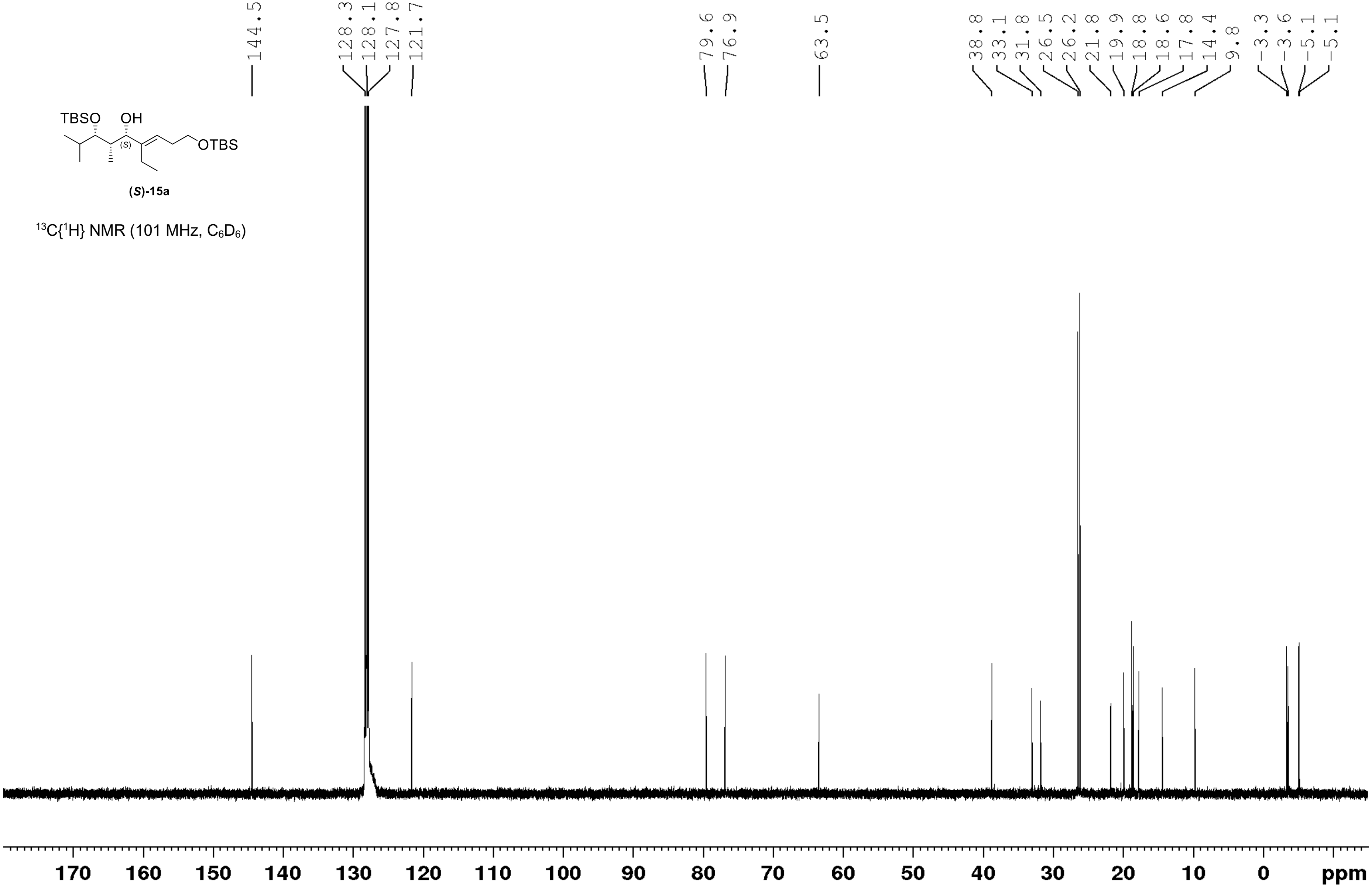
TBSO
OH
OTBS
(S)
(S)-15a
13C{1H} NMR (101 MHz, C6D6)
144.5
128.3
128.1
127.8
121.7
79.6
76.9
63.5
38.8
33.1
31.8
26.5
26.2
21.8
19.9
18.8
18.6
17.8
14.4
9.8
-3.3
-3.6
-5.1
-5.1
170 160 150 140 130 120 110 100 90 80 70 60 50 40 30 20 10 0 ppm

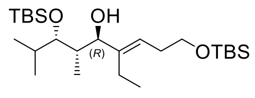

(*R*)-15b

^{1}H NMR (400 MHz, C_6D_6)

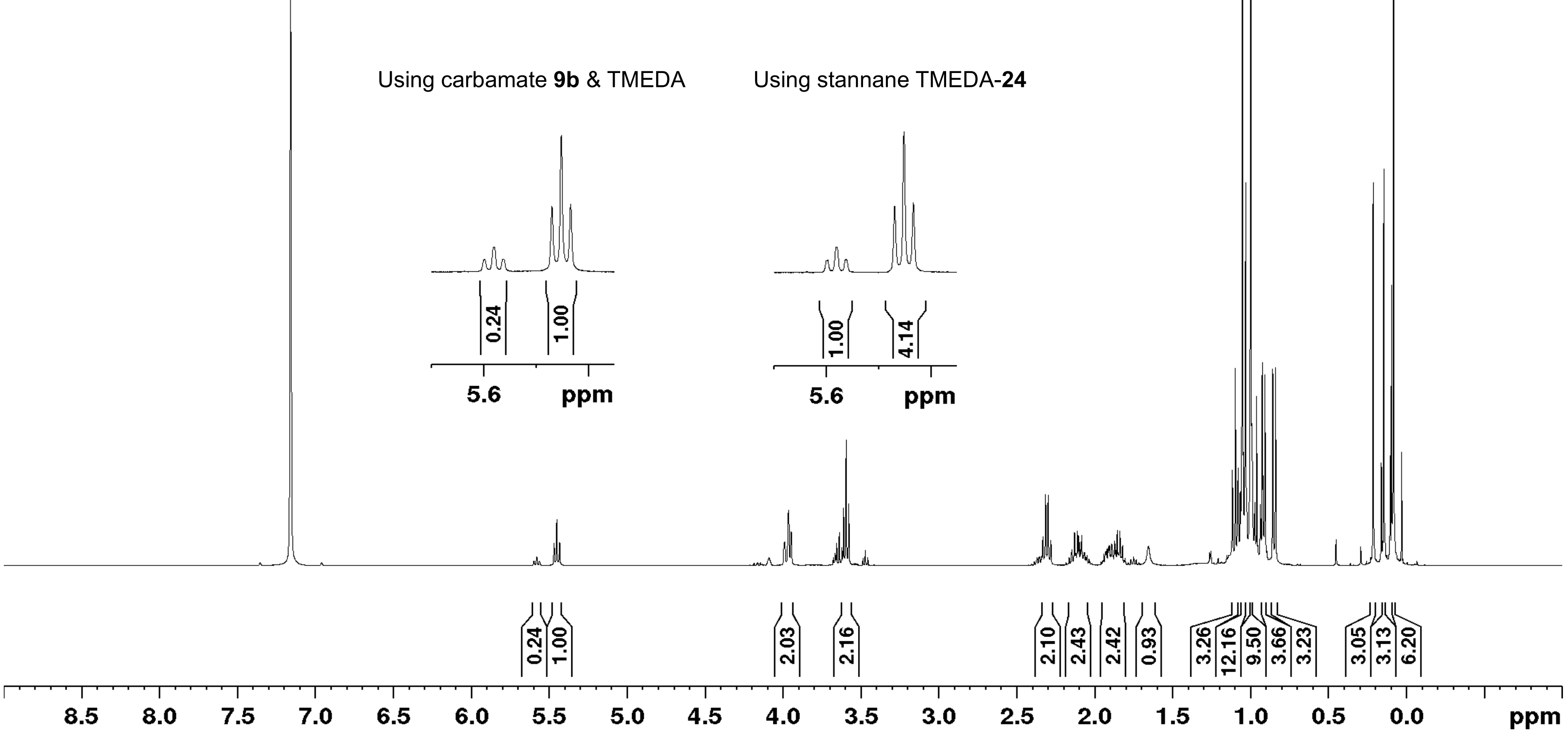

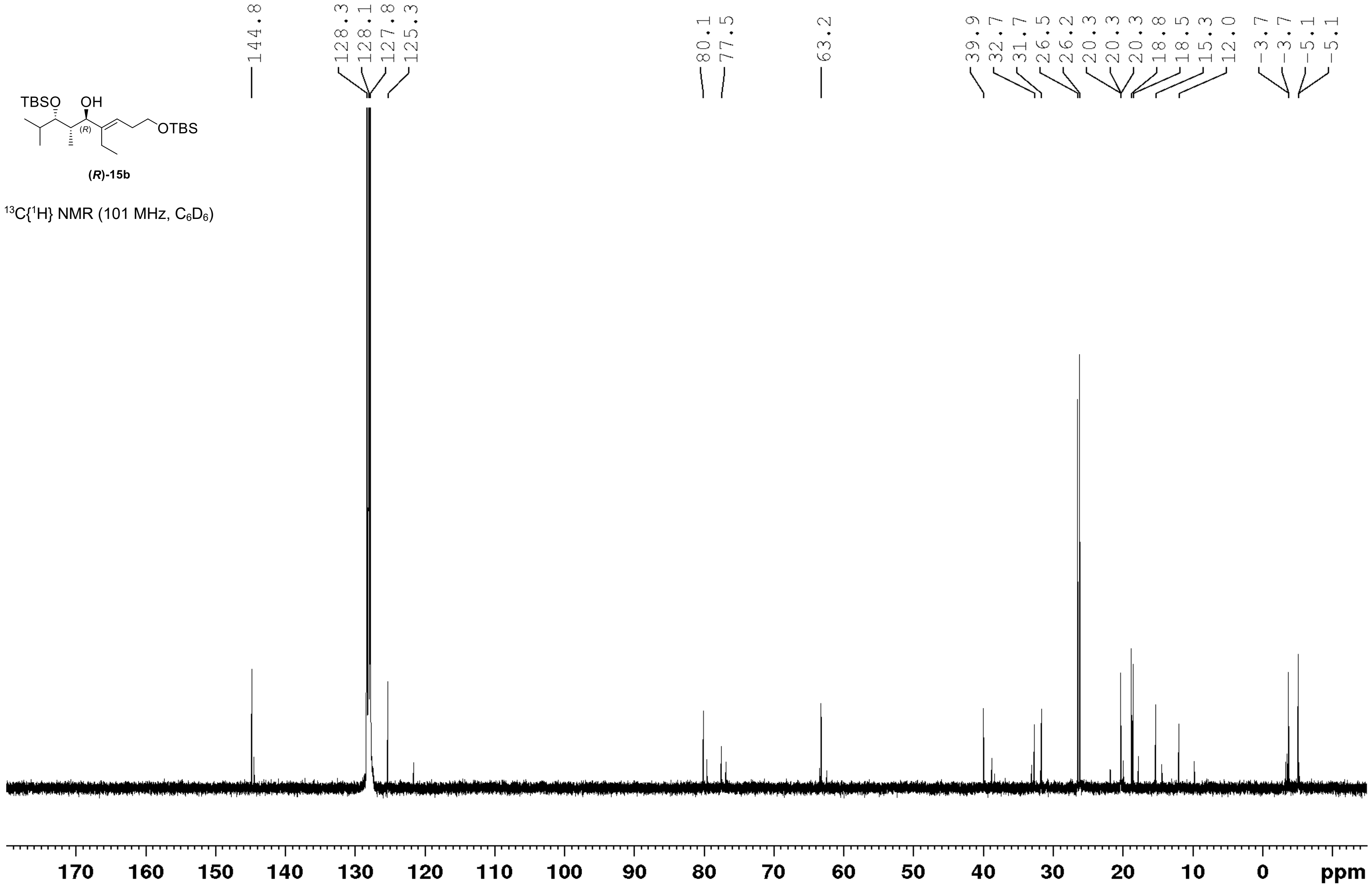
144.8
128.3
128.1
127.8
125.3
80.1
77.5
63.2
39.9
32.7
31.7
26.5
26.2
20.3
20.3
20.3
18.8
18.5
15.3
12.0
-3.7
-3.7
-5.1
-5.1
TBSO
OH
(R)
OTBS
(R)-15b
$^{13}C\{^{1}H\}$ NMR (101 MHz, C_6D_6)
170
160
150
140
130
120
110
100
90
80
70
60
50
40
30
20
10
0
ppm

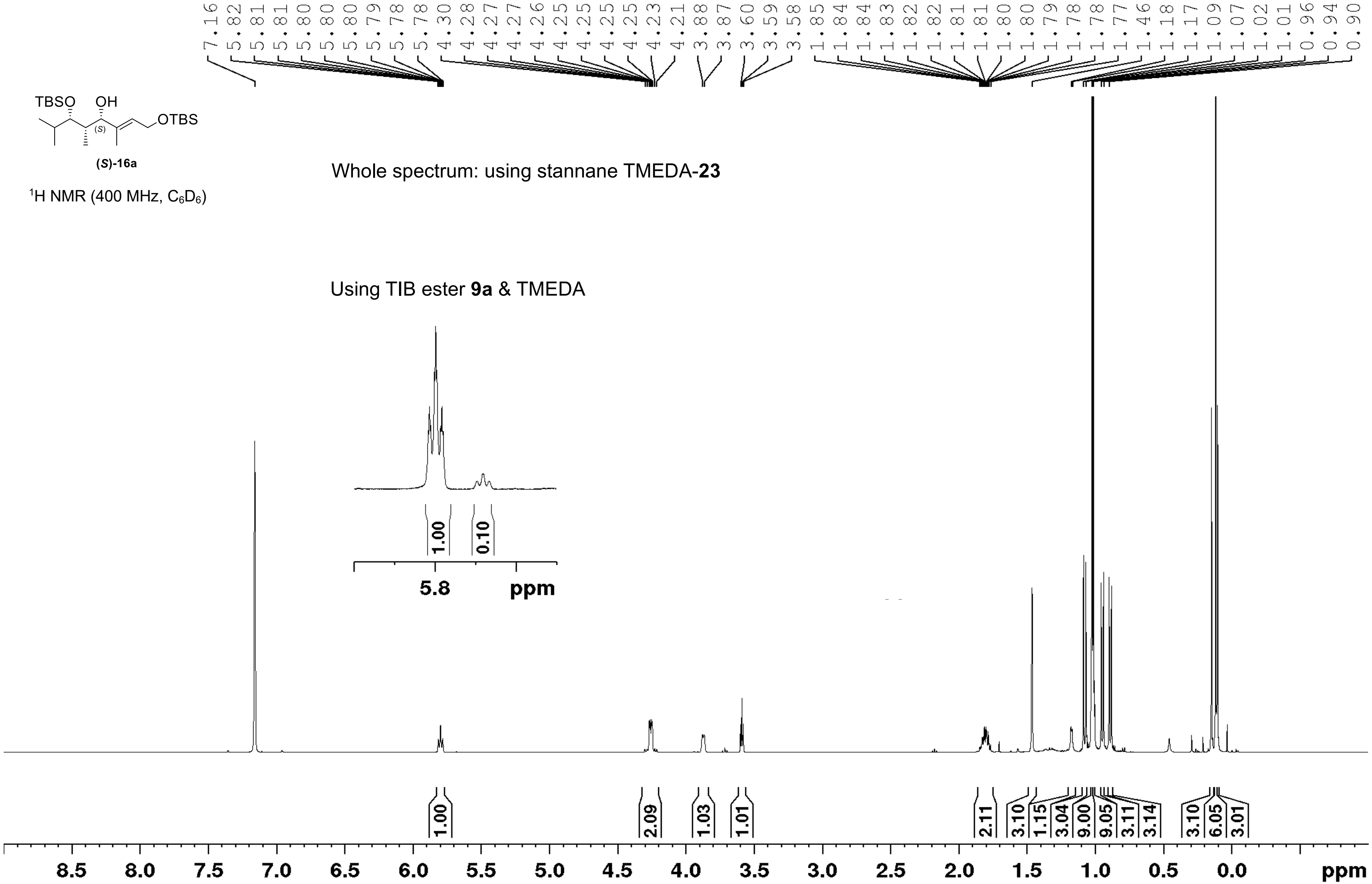

TBSO OH
OTBS
(S)
(S)-16a
^{1}H NMR (400 MHz, C_6D_6)
Whole spectrum: using stannane TMEDA-**23**
Using TIB ester **9a** & TMEDA
7.16 5.82 5.81 5.81 5.80 5.80 5.80 5.79 5.78 5.78 4.30 4.28 4.27 4.27 4.26 4.25 4.25 4.25 4.25 4.23 4.21 3.88 3.87 3.60 3.59 3.58 1.85 1.84 1.84 1.83 1.82 1.82 1.81 1.81 1.80 1.80 1.79 1.78 1.78 1.77 1.46 1.18 1.17 1.09 1.07 1.02 1.01 0.96 0.94 0.90
1.00 0.10
5.8 ppm
1.00 2.09 1.03 1.01 2.11 3.10 1.15 3.04 9.00 9.05 3.11 3.14 3.10 6.05 3.01
8.5 8.0 7.5 7.0 6.5 6.0 5.5 5.0 4.5 4.0 3.5 3.0 2.5 2.0 1.5 1.0 0.5 0.0 ppm

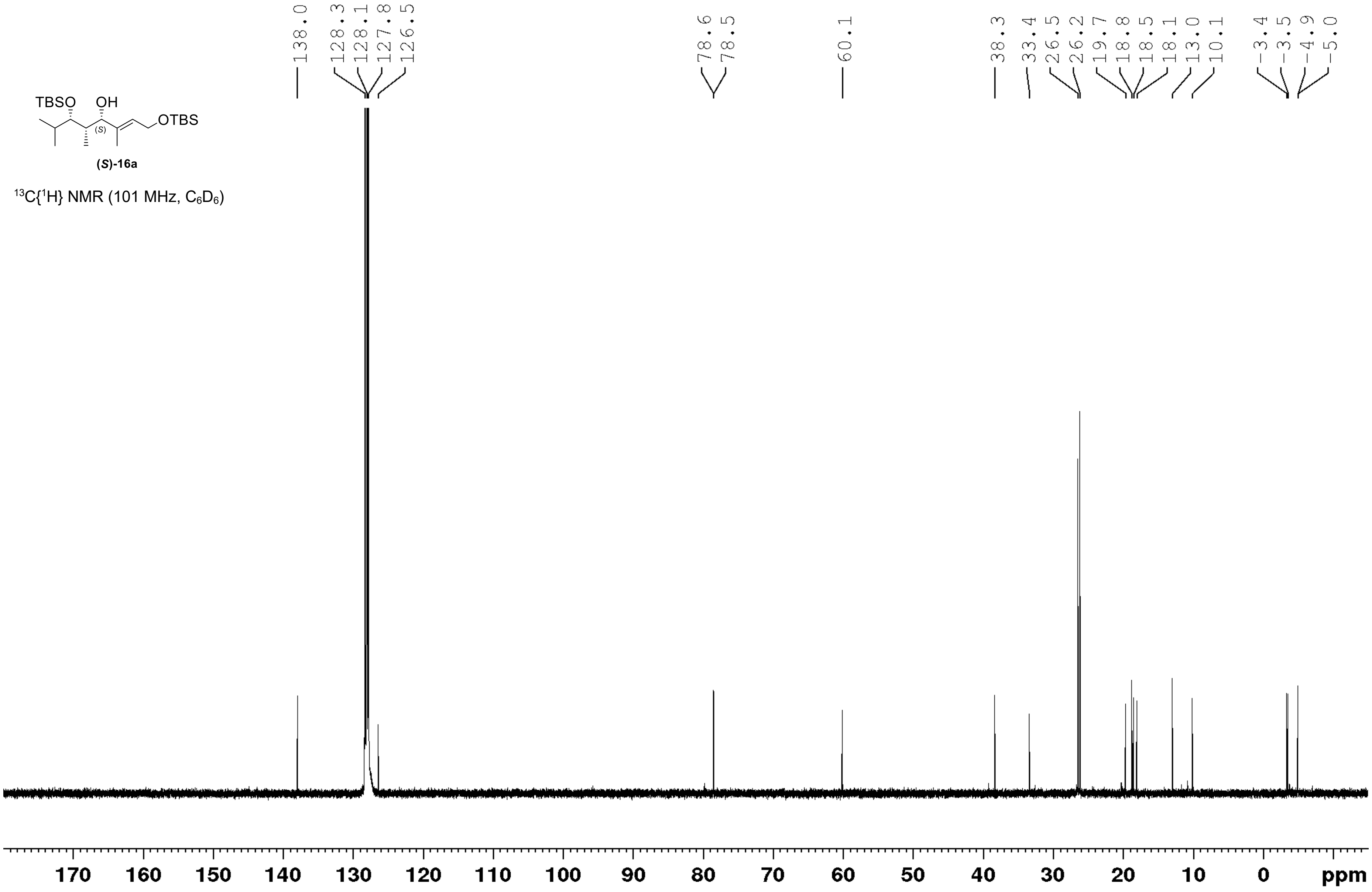
TBSO
OH
OTBS
(S)
(S)-16a
$^{13}C\{^1H\}$ NMR (101 MHz, C_6D_6)
138.0
128.3
128.1
127.8
126.5
78.6
78.5
60.1
38.3
33.4
26.5
26.2
19.7
18.8
18.5
18.1
13.0
10.1
-3.4
-3.5
-4.9
-5.0
170
160
150
140
130
120
110
100
90
80
70
60
50
40
30
20
10
0
ppm

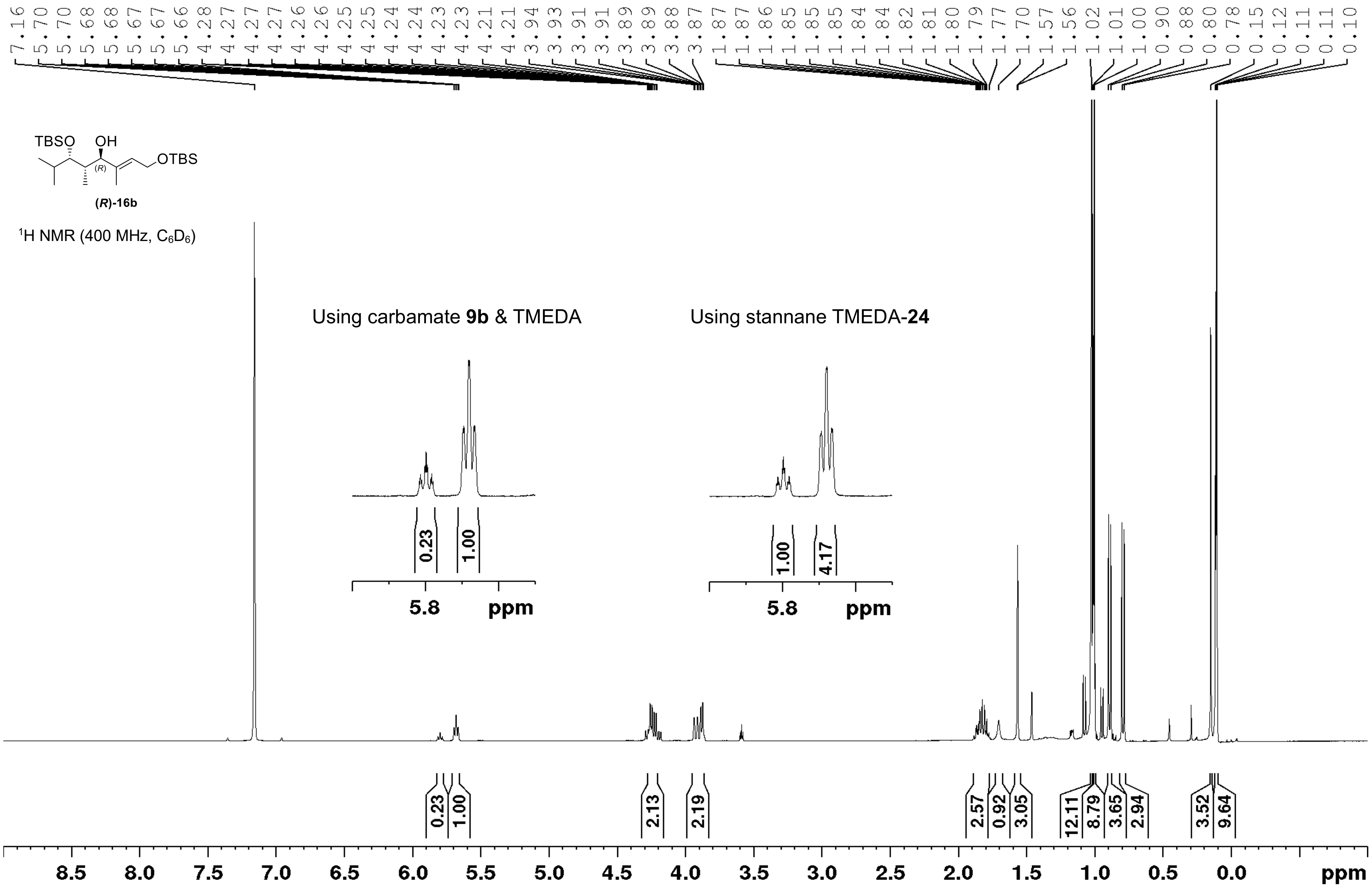

TBSO OH
OTBS
(R)
(R)-16b
1H NMR (400 MHz, C6D6)
Using carbamate 9b & TMEDA
Using stannane TMEDA-24
0.23
1.00
5.8
ppm
1.00
4.17
5.8
ppm
0.23
1.00
2.13
2.19
2.57
0.92
3.05
12.11
8.79
3.65
2.94
3.52
9.64
8.5 8.0 7.5 7.0 6.5 6.0 5.5 5.0 4.5 4.0 3.5 3.0 2.5 2.0 1.5 1.0 0.5 0.0 ppm

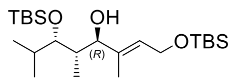

(*R*)-16b

$^{13}C\{^{1}H\}$ NMR (101 MHz, C_6D_6)

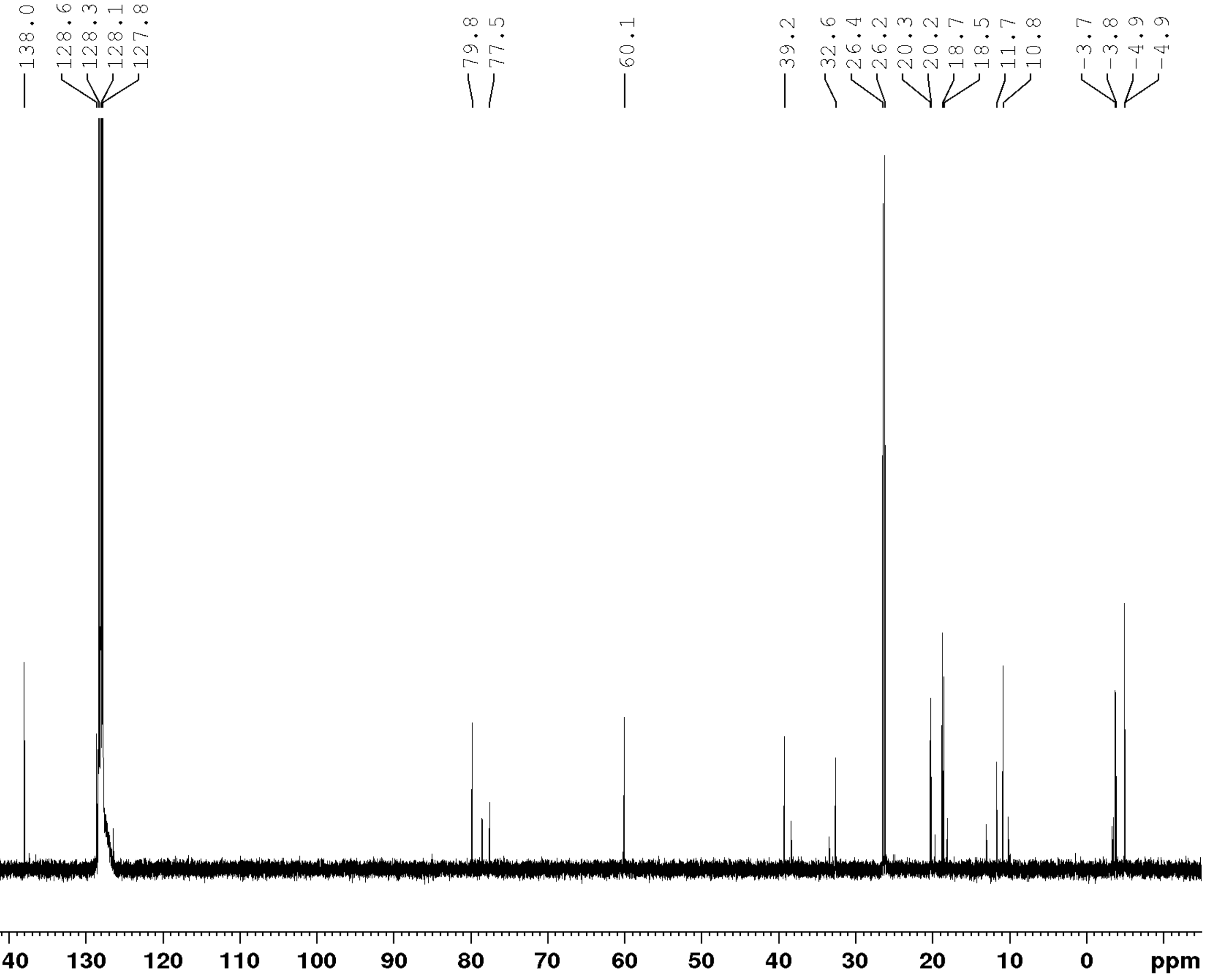

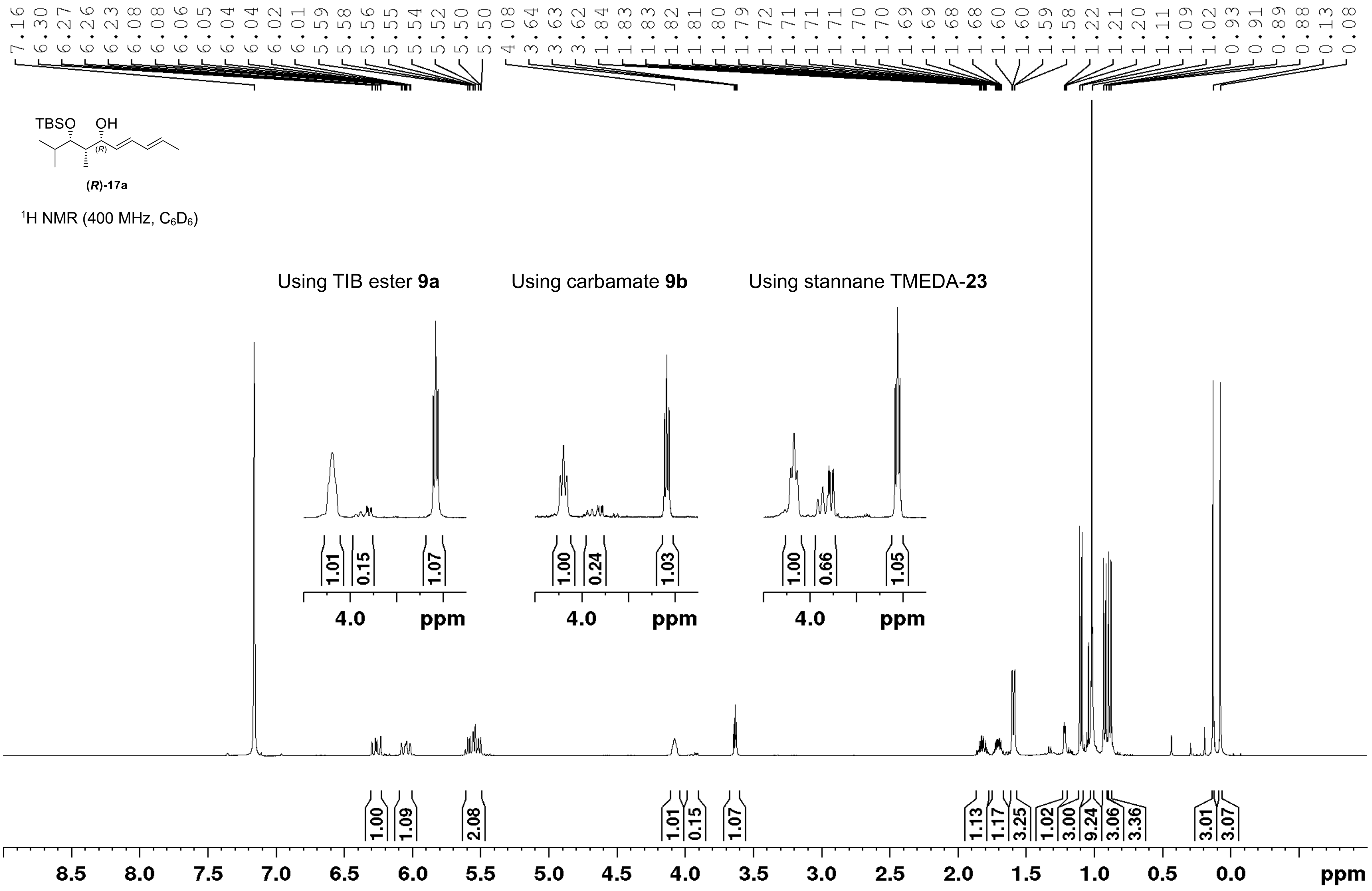

TBSO OH
(R)
(R)-17a
1H NMR (400 MHz, C6D6)
Using TIB ester 9a
Using carbamate 9b
Using stannane TMEDA-23
ppm

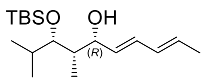

(*R*)-17a

$^{13}C\{^{1}H\}$ NMR (101 MHz, C_6D_6)

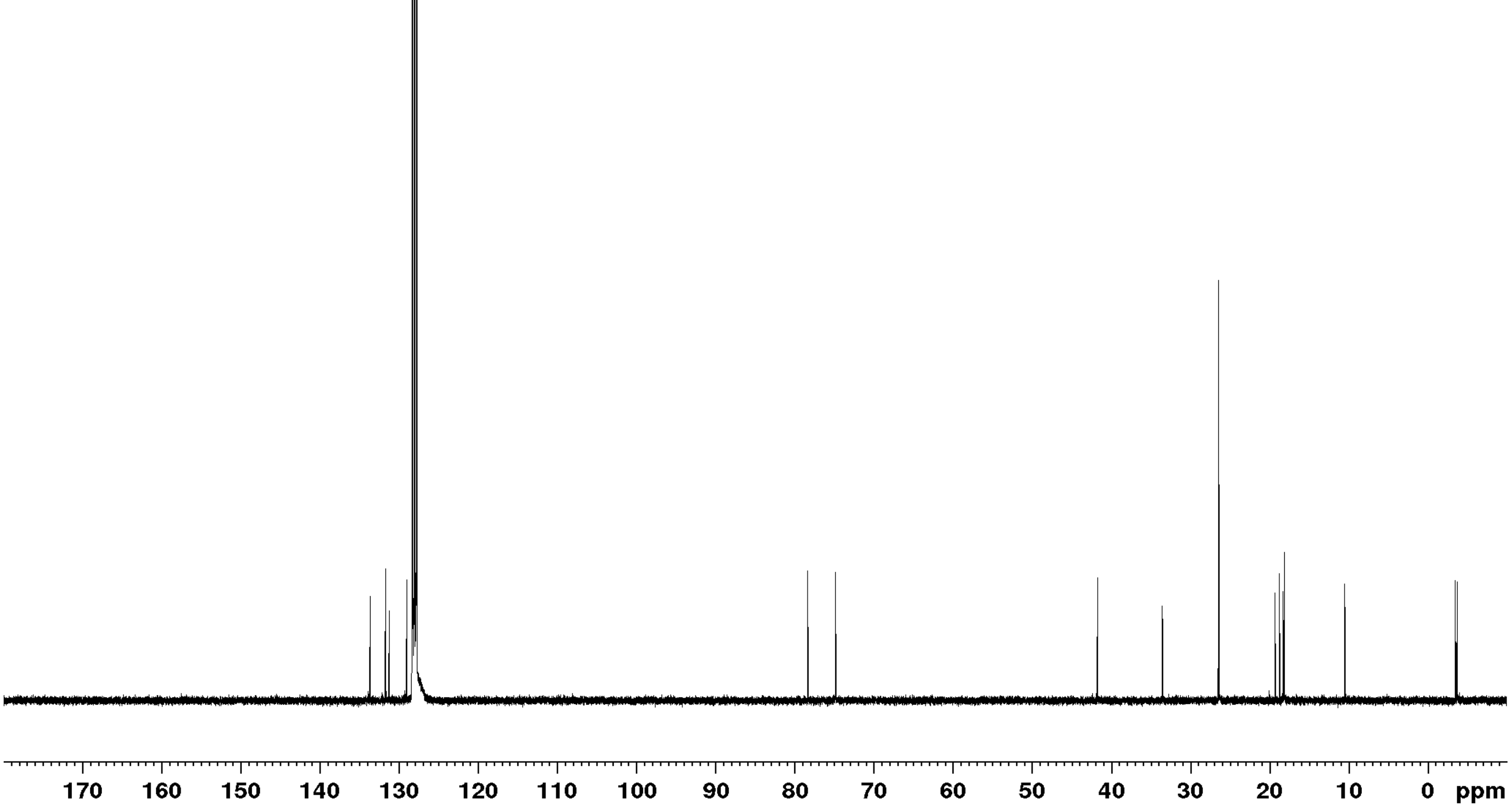

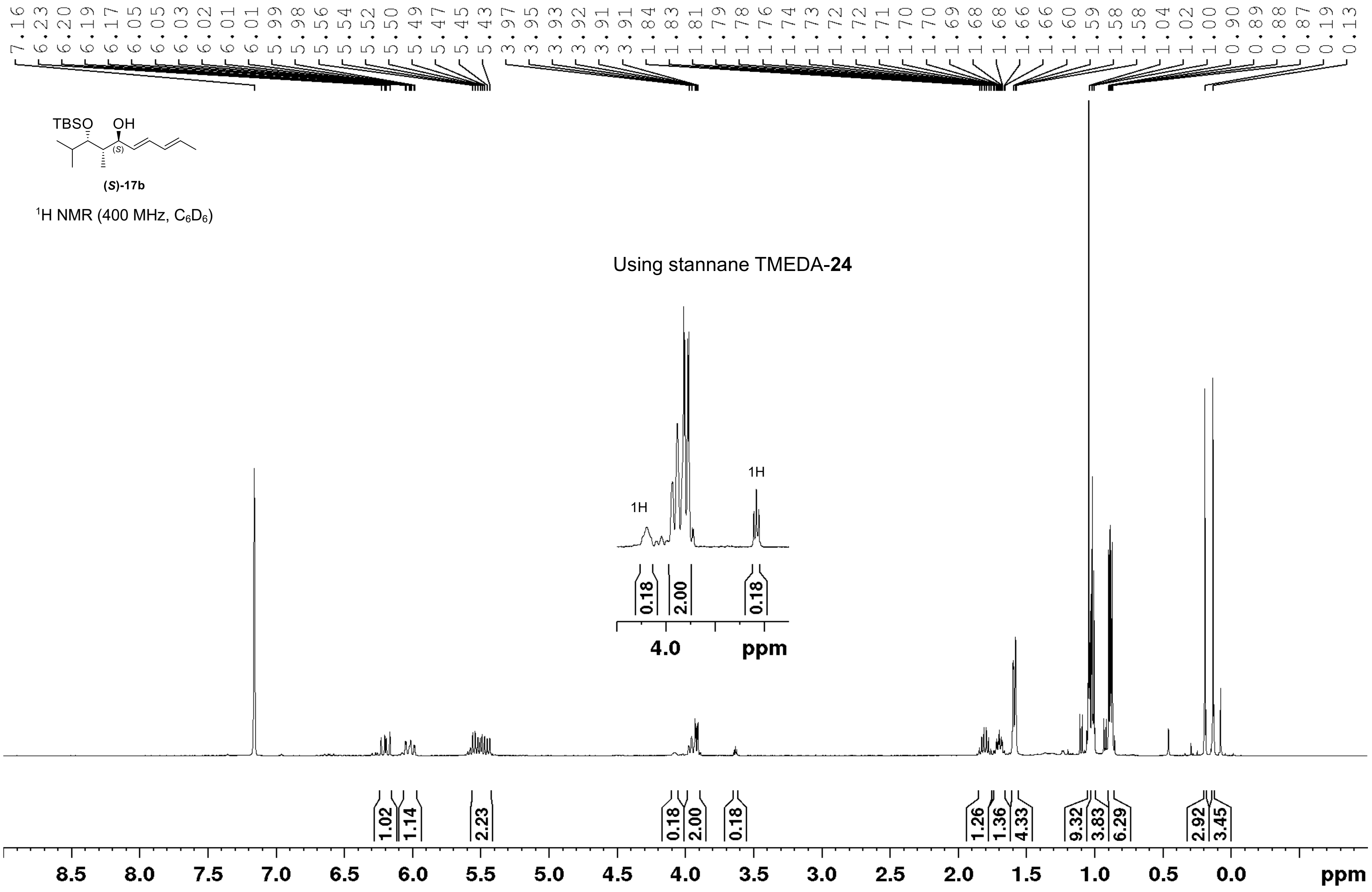
TBSO OH
(S)
(S)-17b
^{1}H NMR (400 MHz, C_6D_6)
Using stannane TMEDA-**24**
1H
1H
0.18
2.00
0.18
4.0
ppm
7.16 6.23 6.20 6.19 6.17 6.05 6.05 6.03 6.02 6.01 6.01 5.99 5.98 5.56 5.54 5.52 5.50 5.49 5.47 5.45 5.43 3.97 3.95 3.93 3.92 3.91 3.91 1.84 1.83 1.81 1.79 1.78 1.76 1.74 1.73 1.72 1.72 1.71 1.70 1.70 1.69 1.68 1.68 1.66 1.66 1.60 1.59 1.58 1.58 1.04 1.02 1.00 0.90 0.89 0.88 0.87 0.19 0.13
1.02 1.14 2.23 0.18 2.00 0.18 1.26 1.36 4.33 9.32 3.83 6.29 2.92 3.45
8.5 8.0 7.5 7.0 6.5 6.0 5.5 5.0 4.5 4.0 3.5 3.0 2.5 2.0 1.5 1.0 0.5 0.0 ppm

(S)-17b

$^{13}C\{^{1}H\}$ NMR (101 MHz, C_6D_6)

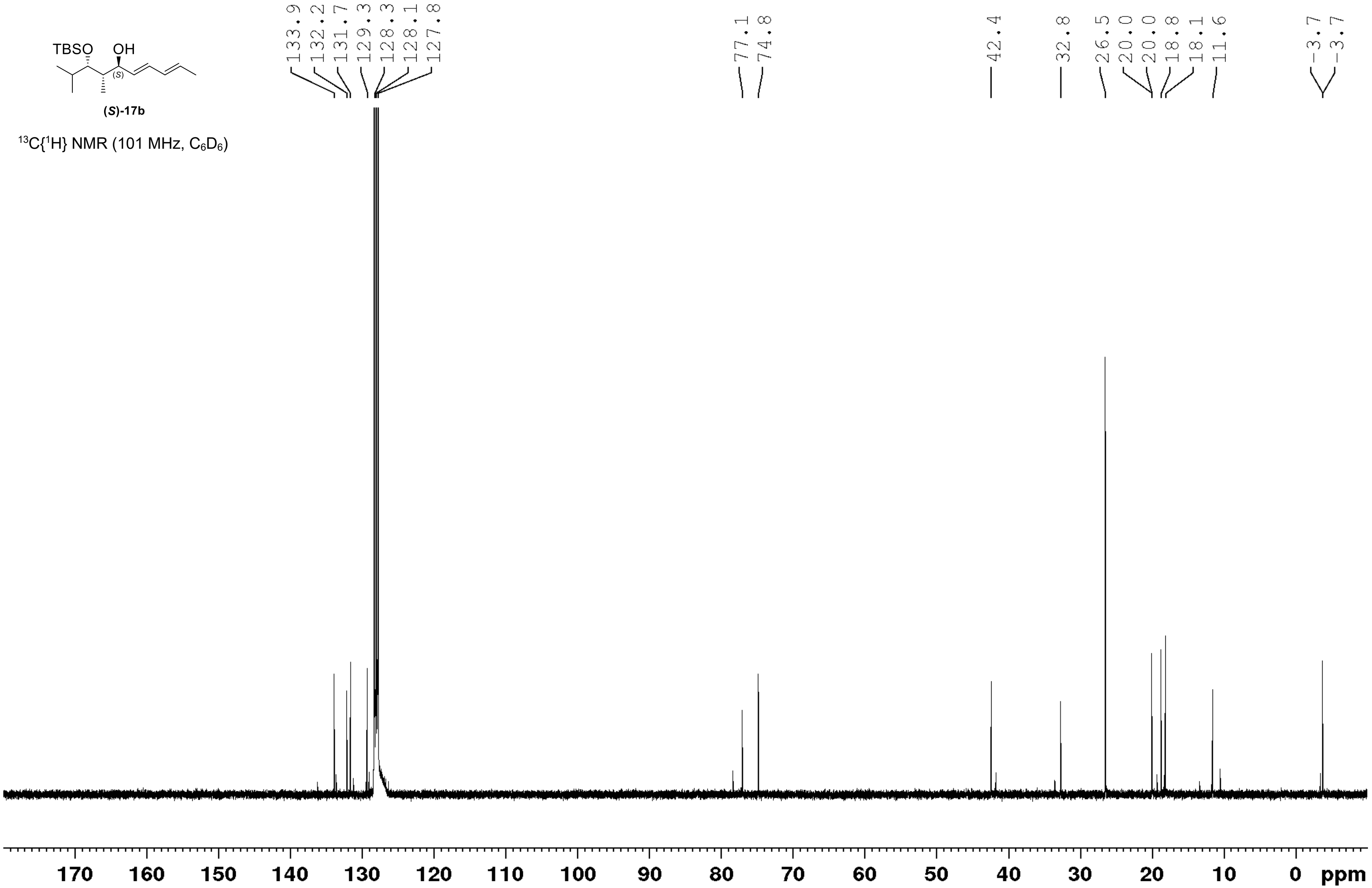

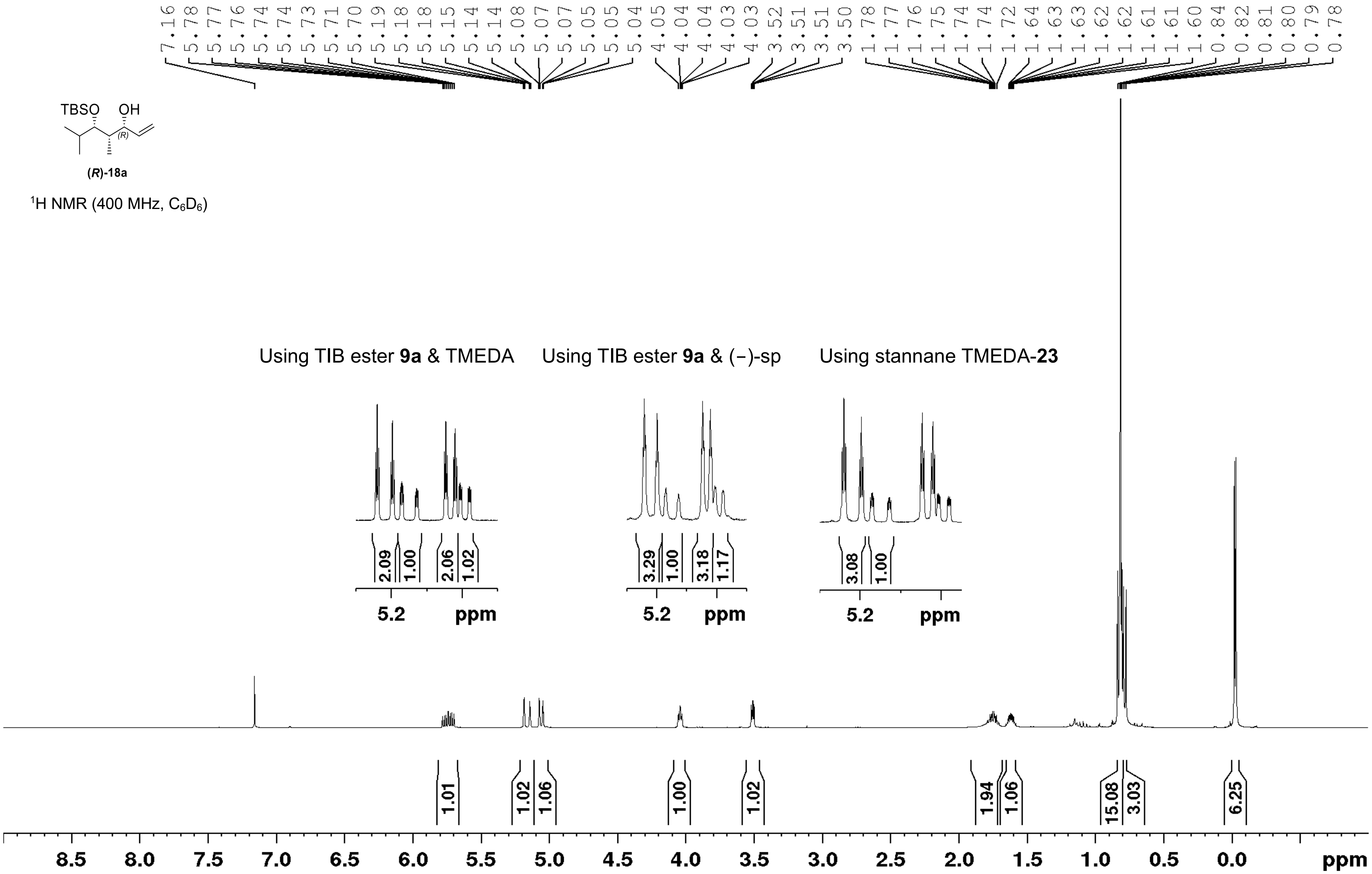

(*R*)-18a

^{1}H NMR (400 MHz, C_6D_6)

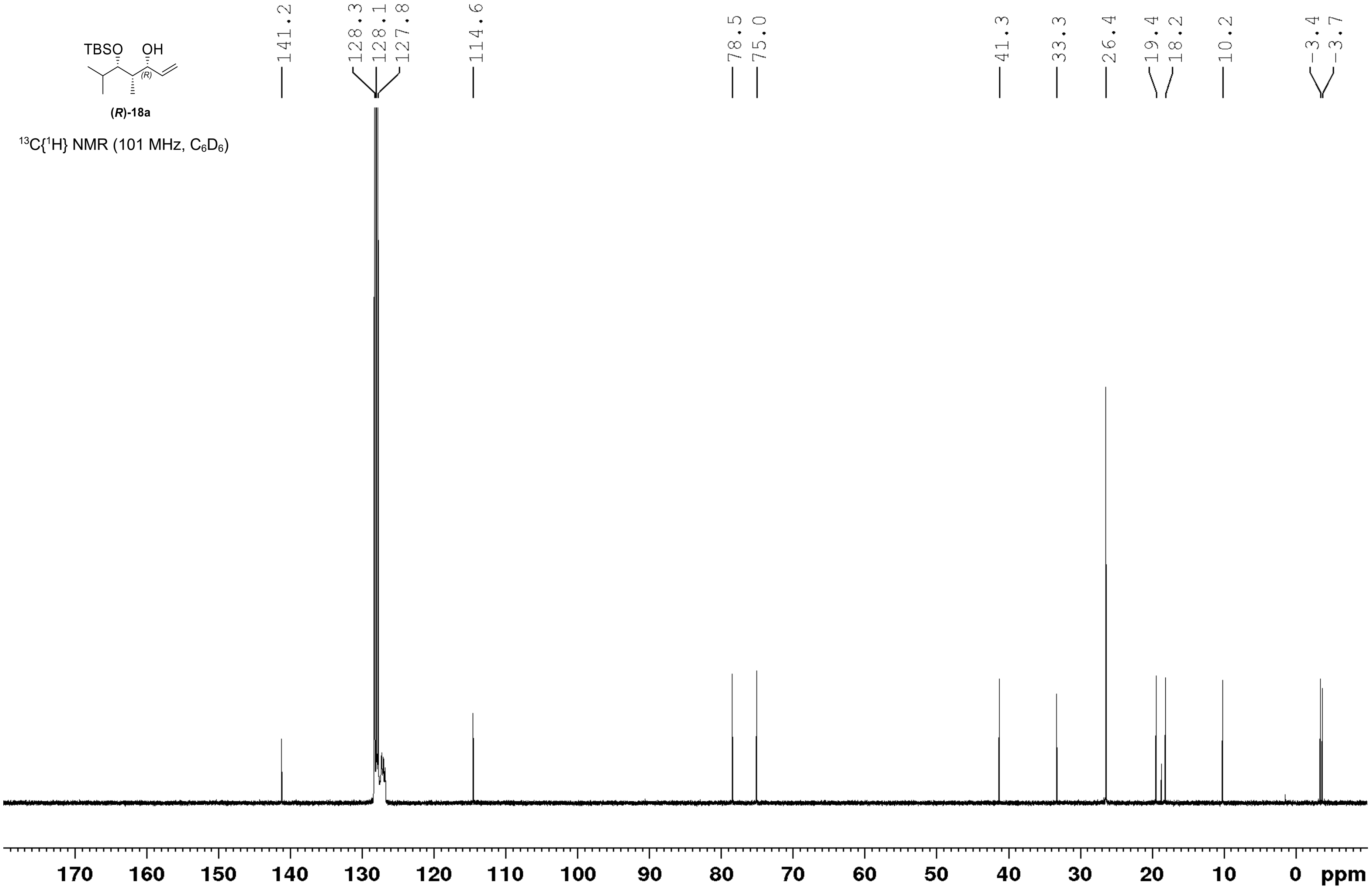

(*R*)-**18a**

$^{13}C\{^1H\}$ NMR (101 MHz, C_6D_6)

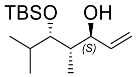

(*S*)-18b

^{1}H NMR (400 MHz, C_6D_6)

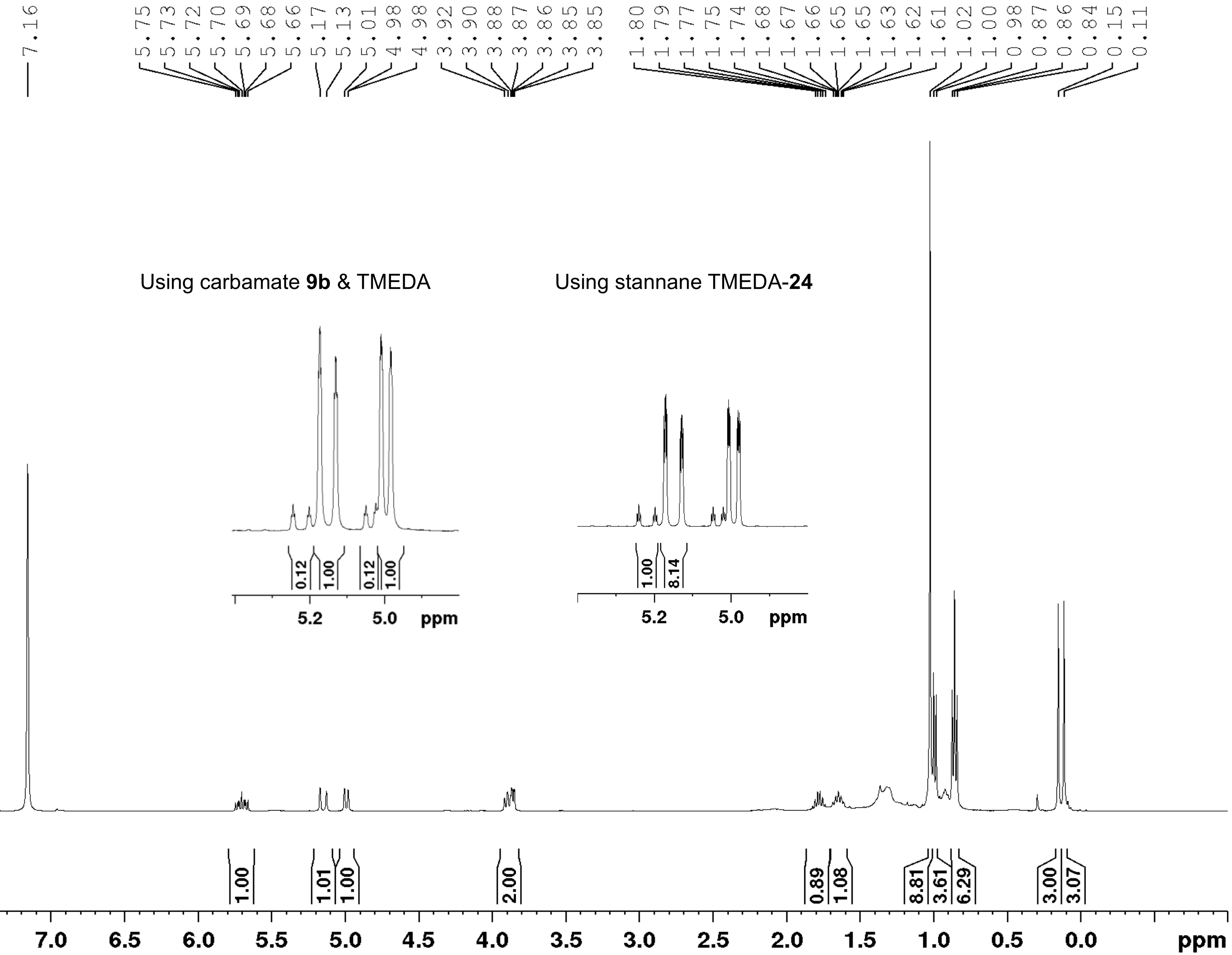

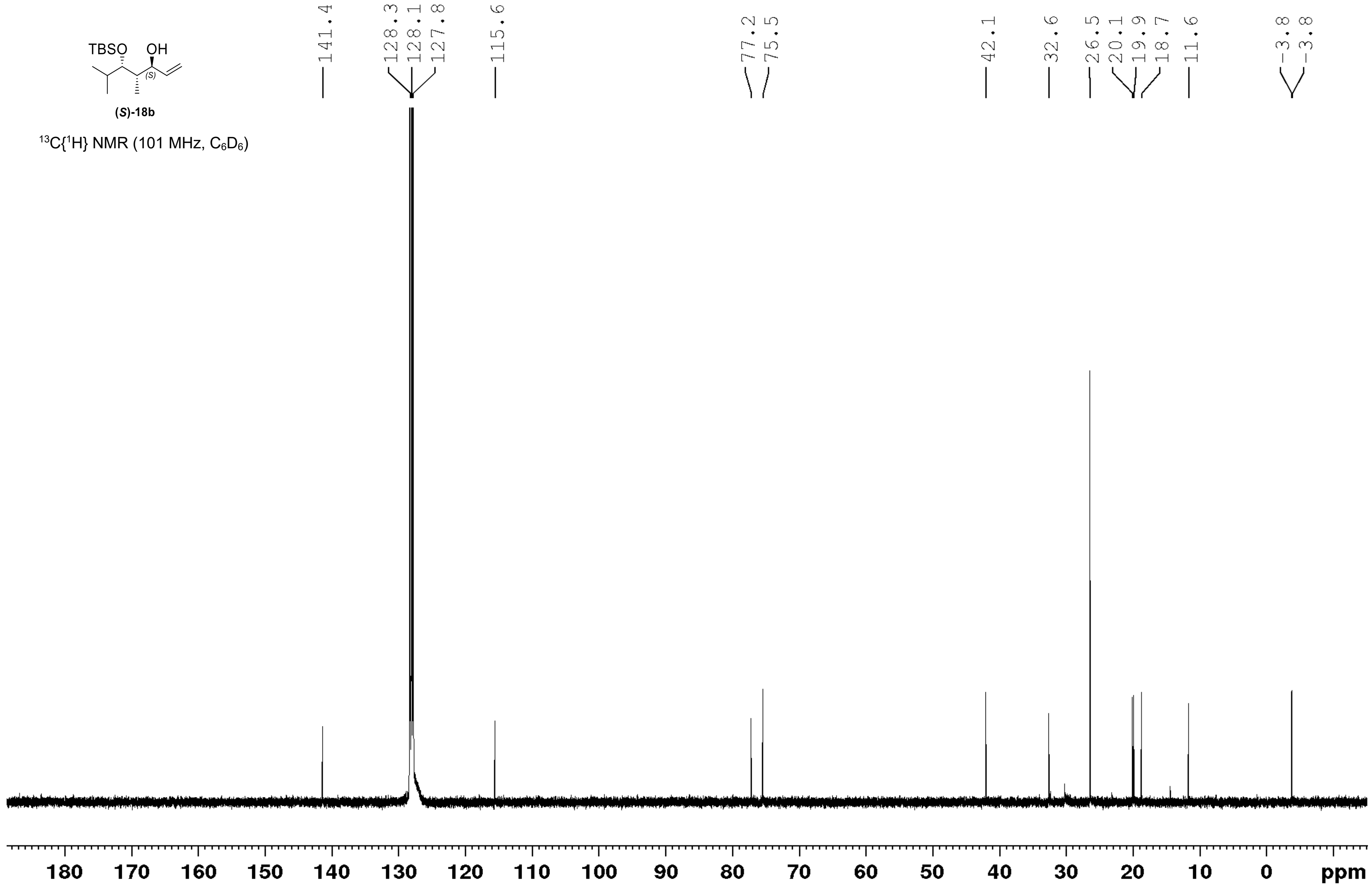
TBSO
OH
(S)
(S)-18b
13C{1H} NMR (101 MHz, C6D6)
141.4
128.3
128.1
127.8
115.6
77.2
75.5
42.1
32.6
26.5
20.1
19.9
18.7
11.6
-3.8
-3.8
180
170
160
150
140
130
120
110
100
90
80
70
60
50
40
30
20
10
0
ppm

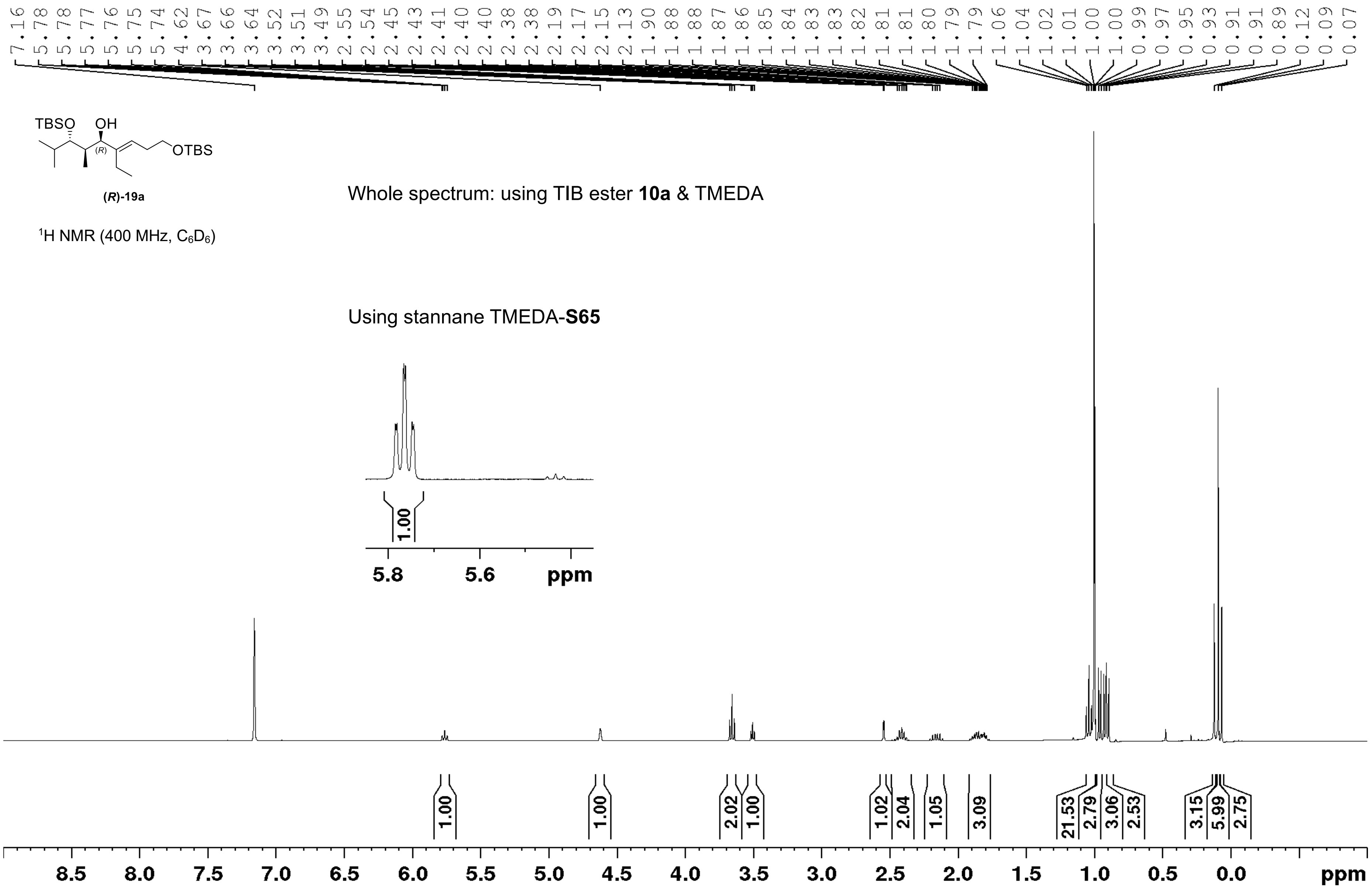

7.16 5.78 5.78 5.77 5.76 5.75 5.74 4.62 3.67 3.66 3.64 3.52 3.51 3.49 2.55 2.54 2.45 2.43 2.41 2.40 2.40 2.38 2.38 2.19 2.17 2.15 2.13 1.90 1.88 1.88 1.87 1.86 1.85 1.84 1.83 1.83 1.82 1.81 1.81 1.80 1.79 1.79 1.06 1.04 1.02 1.01 1.00 1.00 0.99 0.97 0.95 0.93 0.91 0.91 0.89 0.12 0.09 0.07
TBSO
OH
(R)
OTBS
(R)-19a
1H NMR (400 MHz, C6D6)
Whole spectrum: using TIB ester 10a & TMEDA
Using stannane TMEDA-S65
1.00
5.8
5.6
ppm
1.00
1.00
2.02
1.00
1.02
2.04
1.05
3.09
21.53
2.79
3.06
2.53
3.15
5.99
2.75
8.5
8.0
7.5
7.0
6.5
6.0
5.5
5.0
4.5
4.0
3.5
3.0
2.5
2.0
1.5
1.0
0.5
0.0
ppm

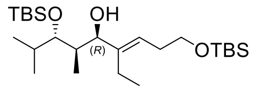

(**R**)-**19a**

$^{13}C\{^{1}H\}$ NMR (101 MHz, C_6D_6)

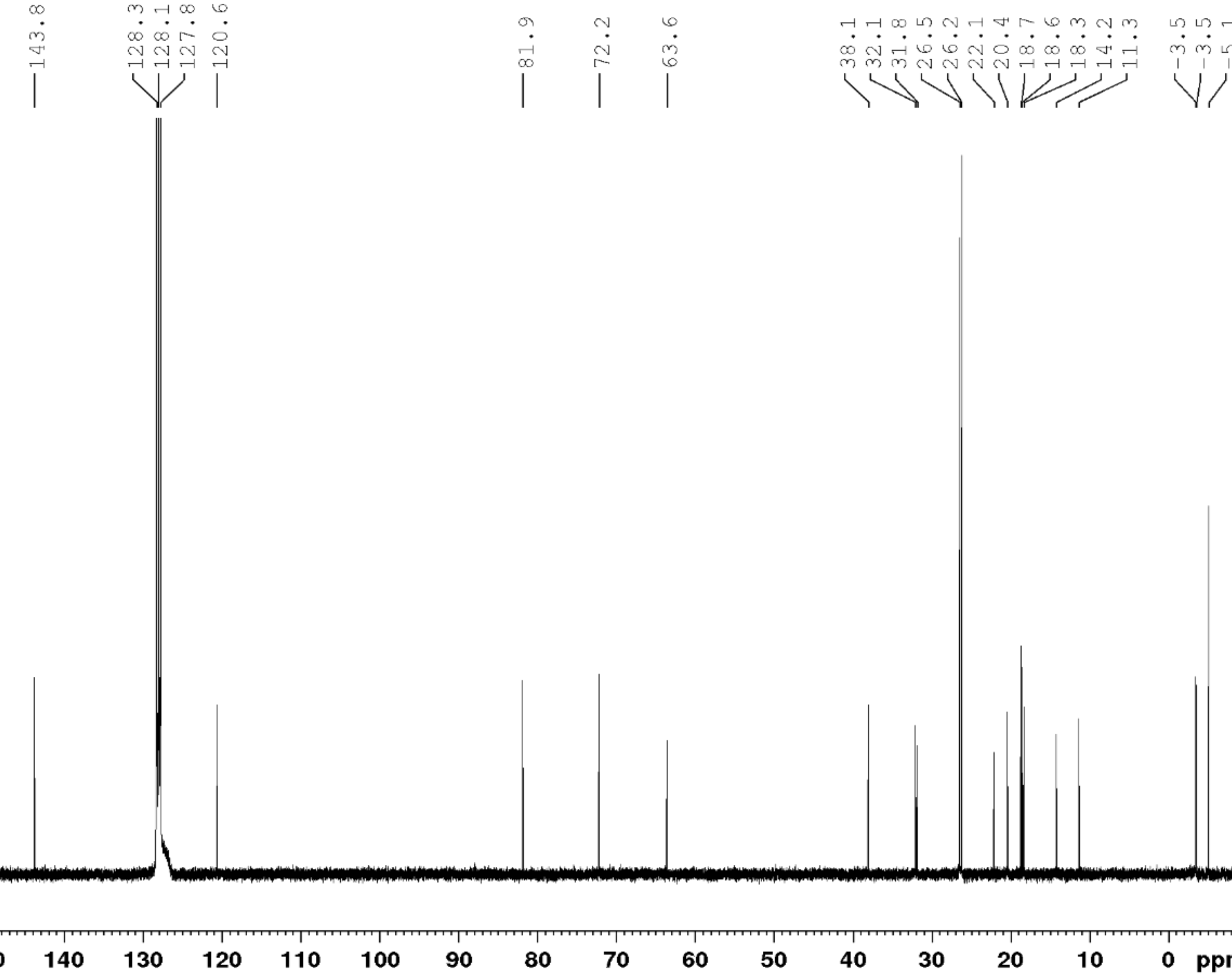

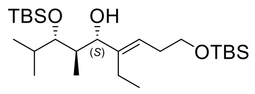

(*S*)-19b

^{1}H NMR (400 MHz, C_6D_6)

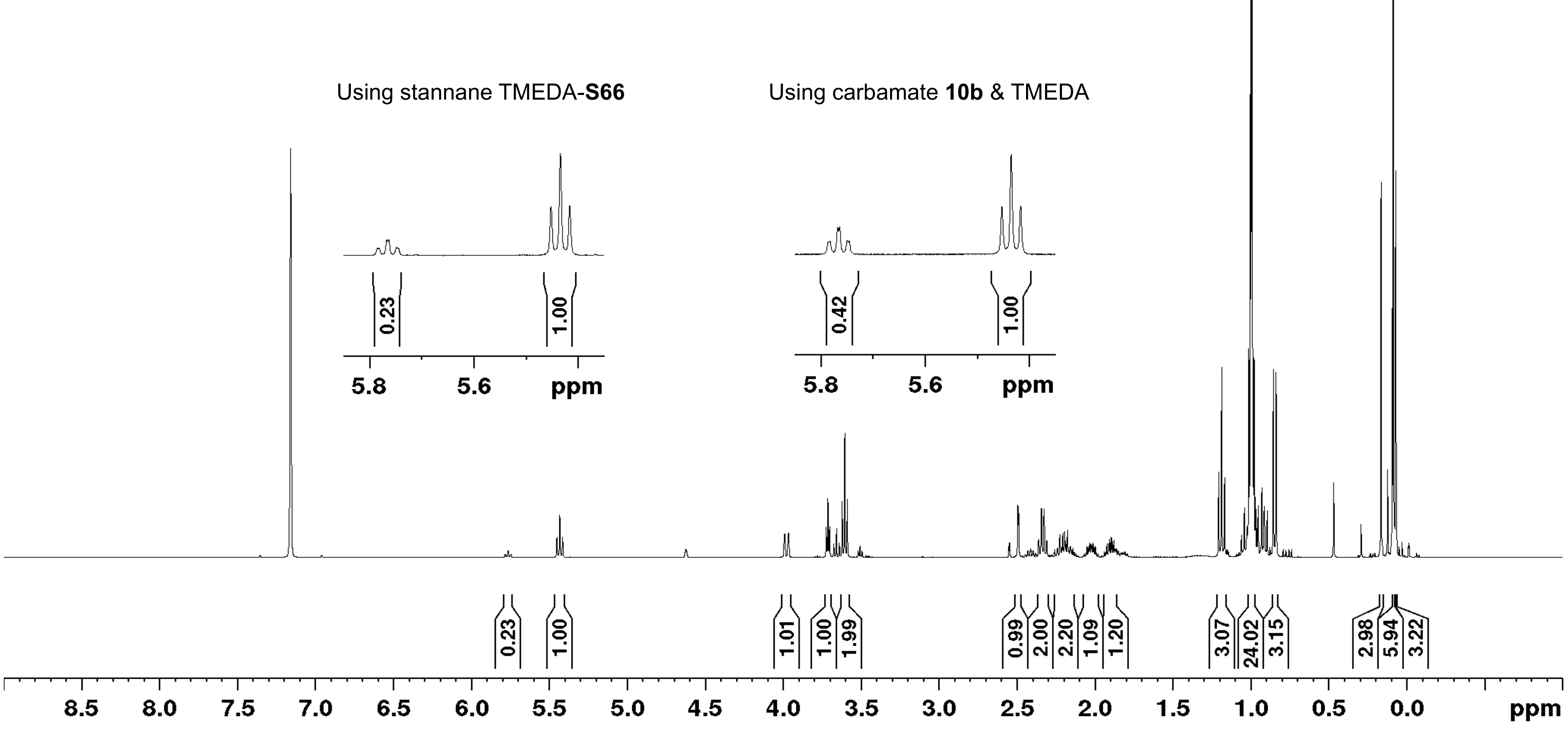

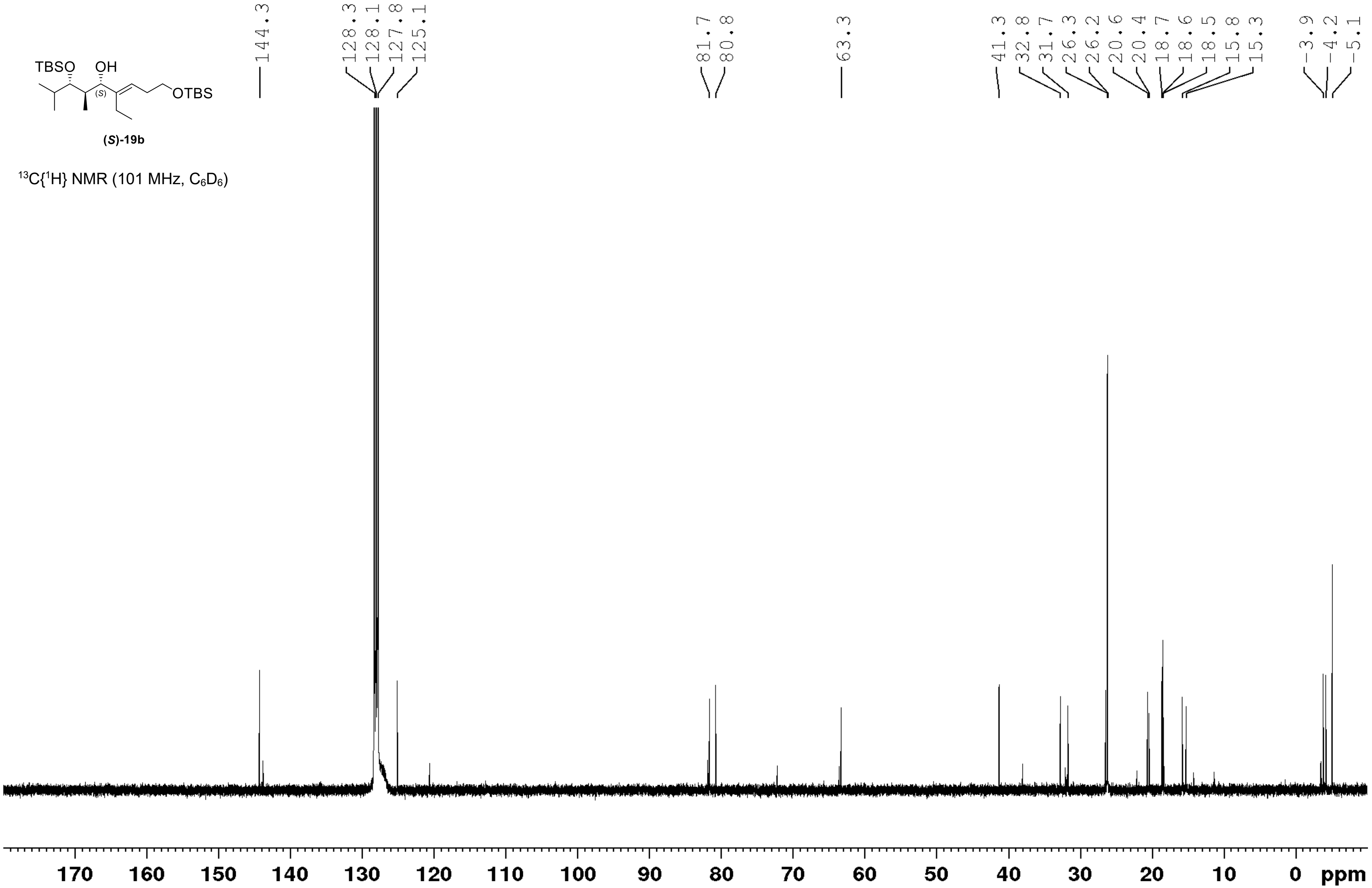

(*S*)-19b

$^{13}C\{^1H\}$ NMR (101 MHz, C_6D_6)

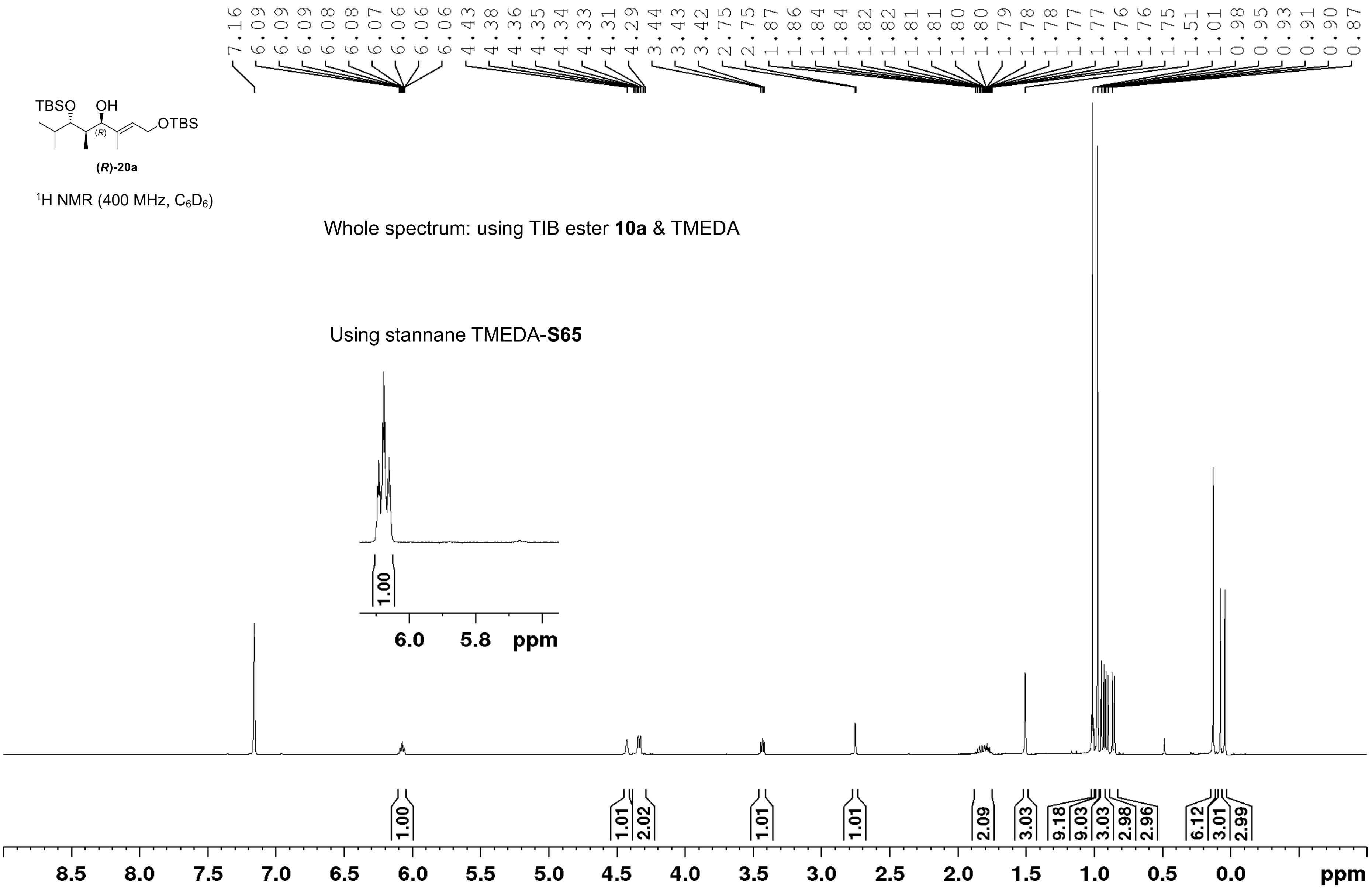

TBSO OH
OTBS
(R)
(R)-20a
1H NMR (400 MHz, C6D6)
Whole spectrum: using TIB ester 10a & TMEDA
Using stannane TMEDA-S65
7.16 6.09 6.09 6.09 6.08 6.08 6.07 6.06 6.06 6.06 4.43 4.38 4.36 4.35 4.34 4.33 4.31 4.29 3.44 3.43 3.42 2.75 2.75 1.87 1.86 1.84 1.84 1.82 1.82 1.81 1.81 1.80 1.80 1.79 1.78 1.78 1.77 1.77 1.76 1.76 1.75 1.51 1.01 0.98 0.95 0.93 0.91 0.90 0.87
1.00
6.0 5.8 ppm
1.00 1.01 2.02 1.01 1.01 2.09 3.03 9.18 9.03 3.03 2.98 2.96 6.12 3.01 2.99
8.5 8.0 7.5 7.0 6.5 6.0 5.5 5.0 4.5 4.0 3.5 3.0 2.5 2.0 1.5 1.0 0.5 0.0 ppm

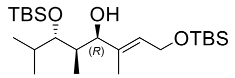

(*R*)-20a

$^{13}C\{^{1}H\}$ NMR (101 MHz, C_6D_6)

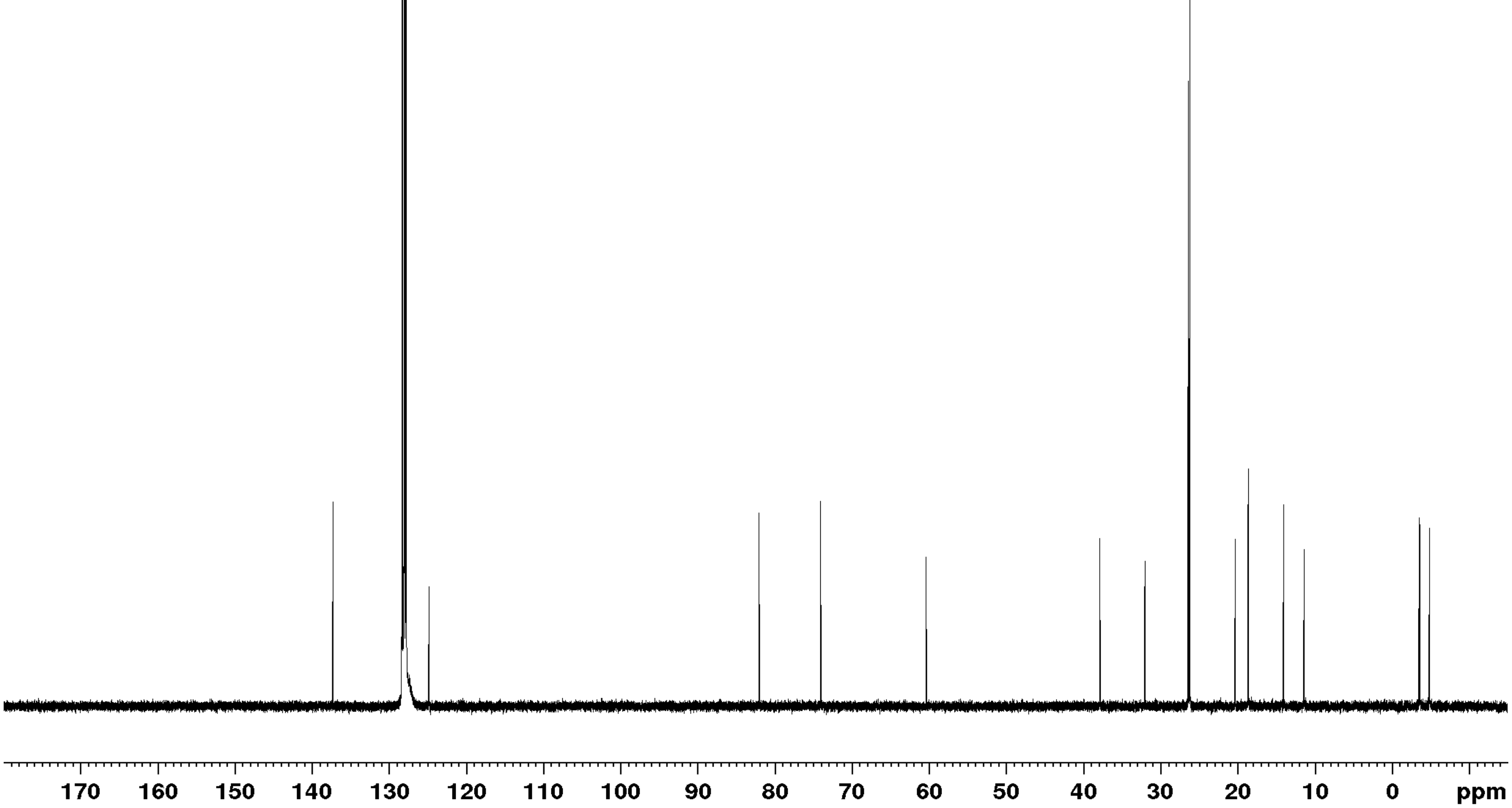

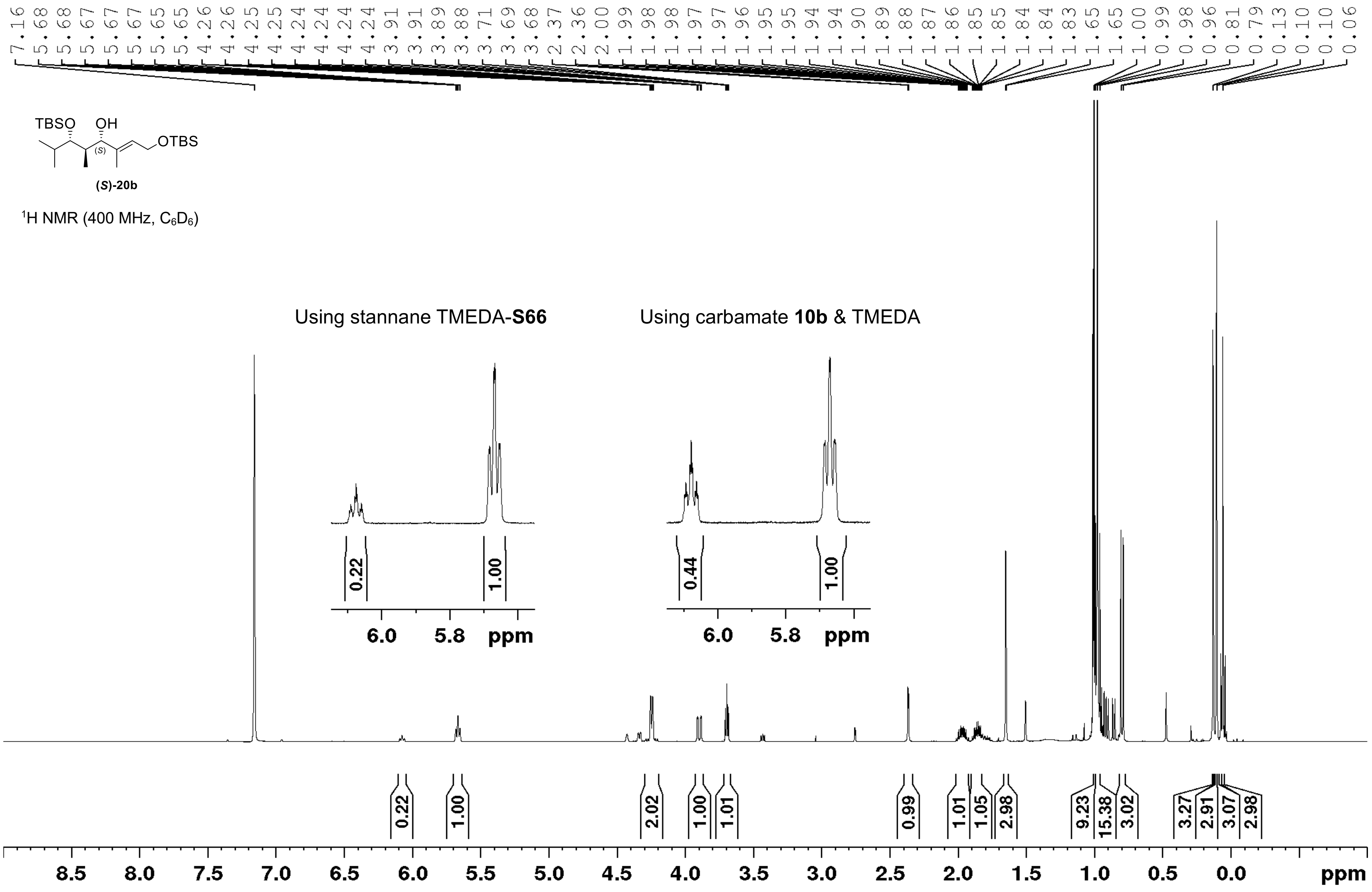
TBSO OH
OTBS
(S)
(S)-20b
^{1}H NMR (400 MHz, C_6D_6)
Using stannane TMEDA-**S66**
Using carbamate **10b** & TMEDA
ppm

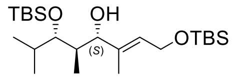

(*S*)-20b

$^{13}C\{^{1}H\}$ NMR (101 MHz, C_6D_6)

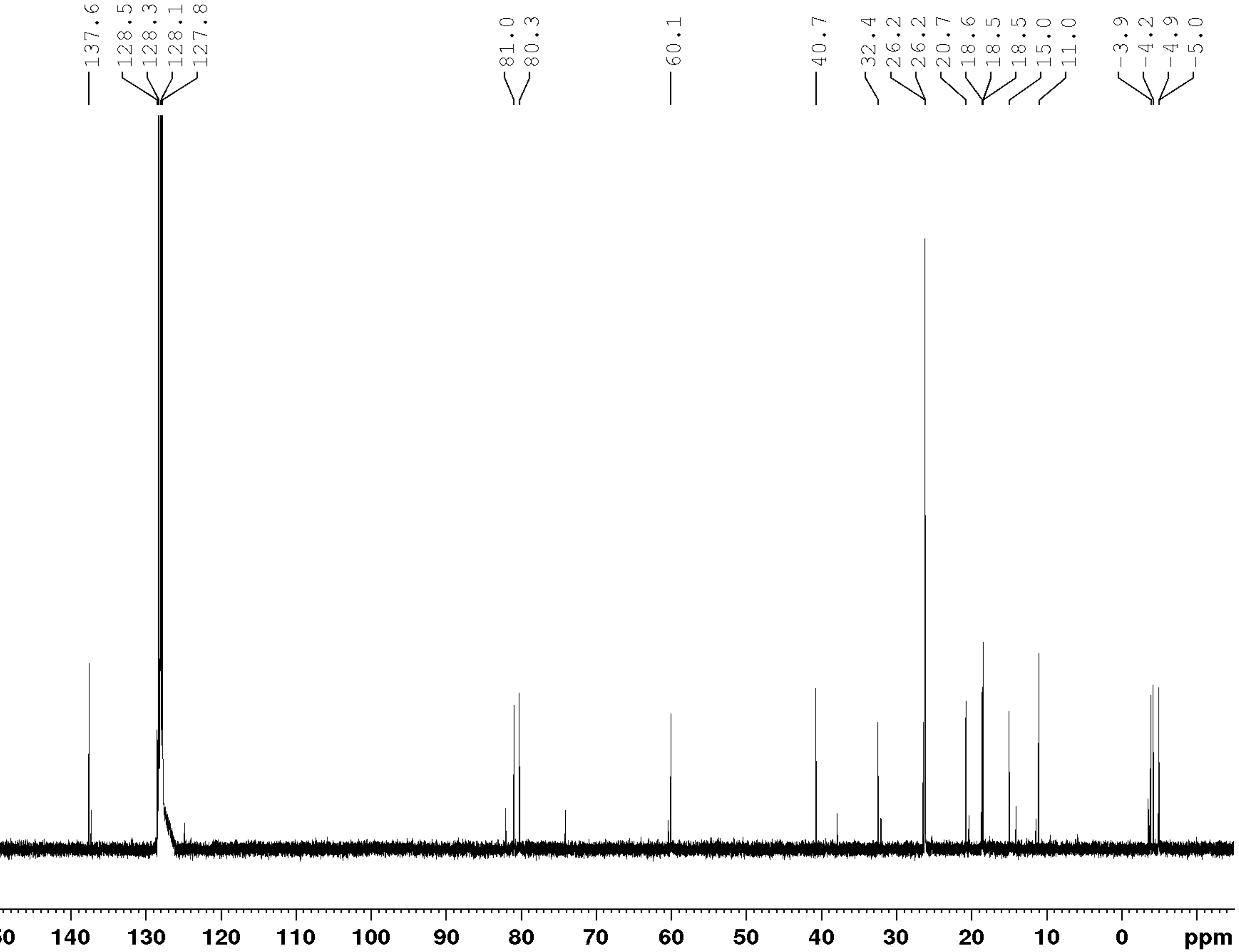

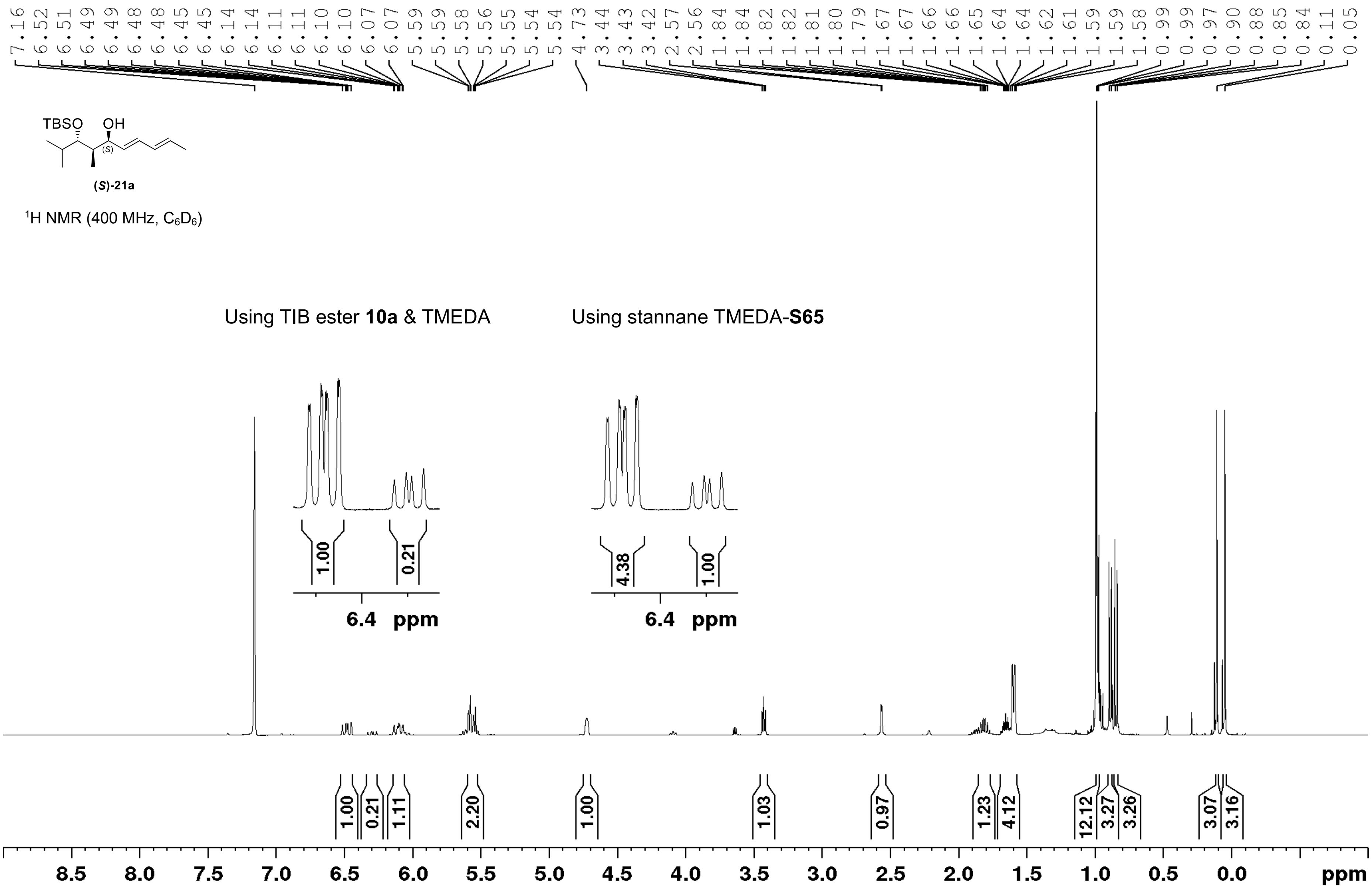
7.16 6.52 6.51 6.49 6.49 6.48 6.48 6.45 6.45 6.14 6.14 6.11 6.11 6.10 6.10 6.07 6.07 5.59 5.59 5.58 5.56 5.55 5.54 5.54 4.73 3.44 3.43 3.42 2.57 2.56 1.84 1.84 1.82 1.82 1.81 1.80 1.79 1.67 1.67 1.66 1.66 1.65 1.64 1.64 1.62 1.61 1.59 1.59 1.58 0.99 0.99 0.97 0.90 0.88 0.85 0.84 0.11 0.05
TBSO
OH
(S)
(S)-21a
1H NMR (400 MHz, C6D6)
Using TIB ester 10a & TMEDA
Using stannane TMEDA-S65
1.00
0.21
6.4 ppm
4.38
1.00
6.4 ppm
1.00
0.21
1.11
2.20
1.00
1.03
0.97
1.23
4.12
12.12
3.27
3.26
3.07
3.16
8.5 8.0 7.5 7.0 6.5 6.0 5.5 5.0 4.5 4.0 3.5 3.0 2.5 2.0 1.5 1.0 0.5 0.0 ppm

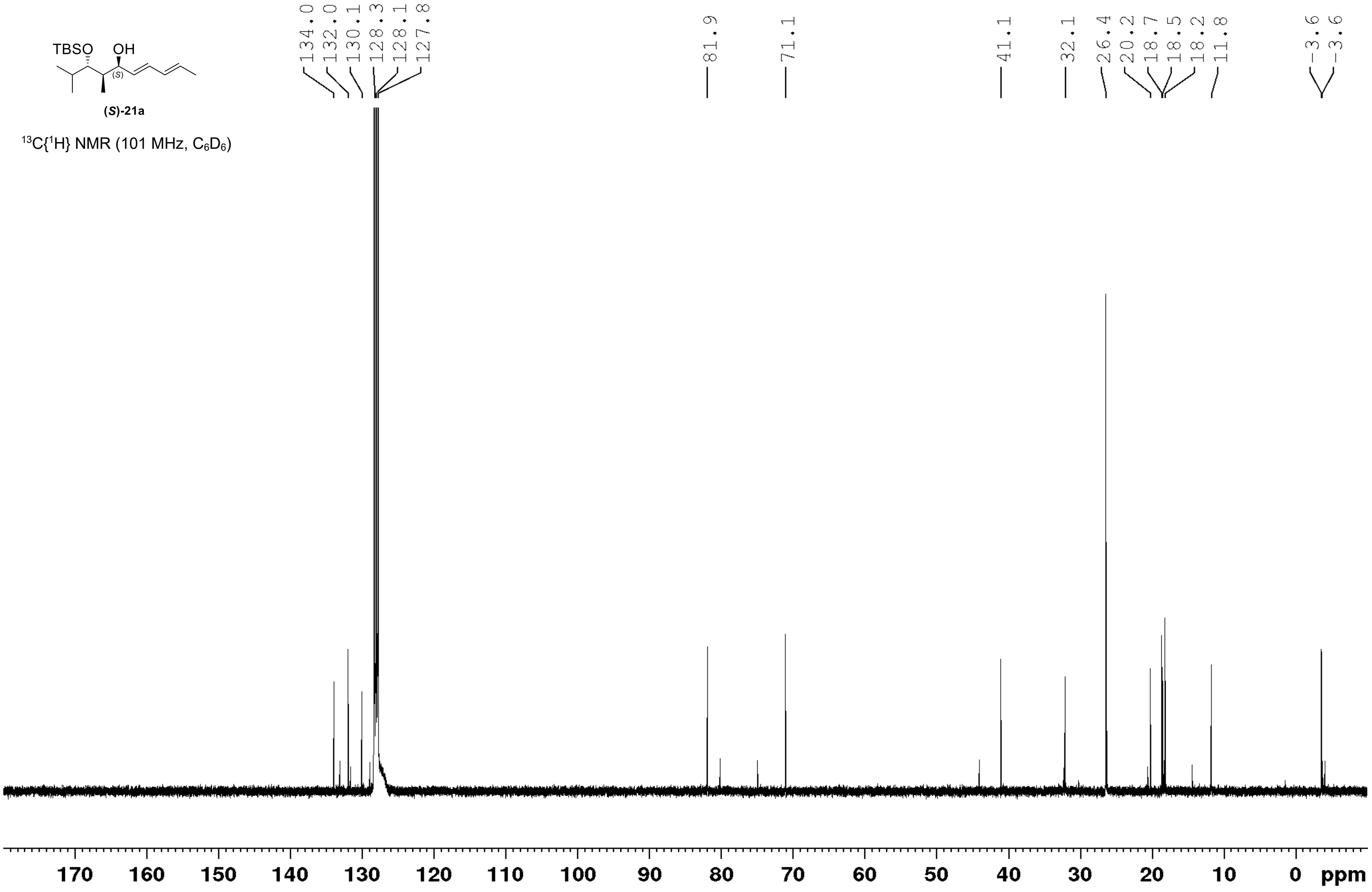
TBSO
OH
(S)
(S)-21a
$^{13}C\{^{1}H\}$ NMR (101 MHz, C_6D_6)
134.0
132.0
130.1
128.3
128.1
127.8
81.9
71.1
41.1
32.1
26.4
20.2
18.7
18.5
18.2
11.8
-3.6
-3.6
170
160
150
140
130
120
110
100
90
80
70
60
50
40
30
20
10
0
ppm

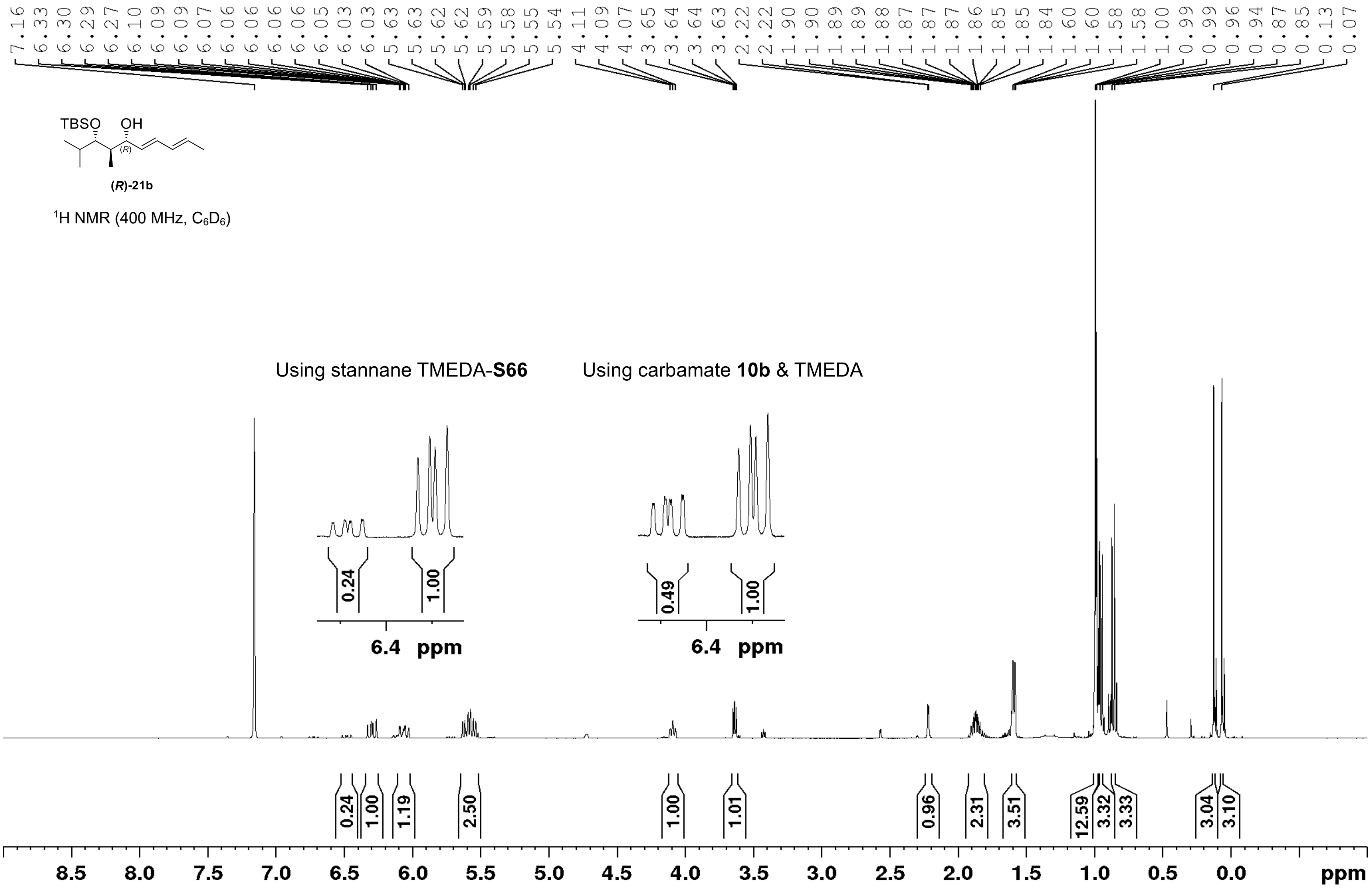

TBSO OH
(R)
(R)-21b
1H NMR (400 MHz, C6D6)
Using stannane TMEDA-S66
Using carbamate 10b & TMEDA
6.4 ppm
6.4 ppm
ppm

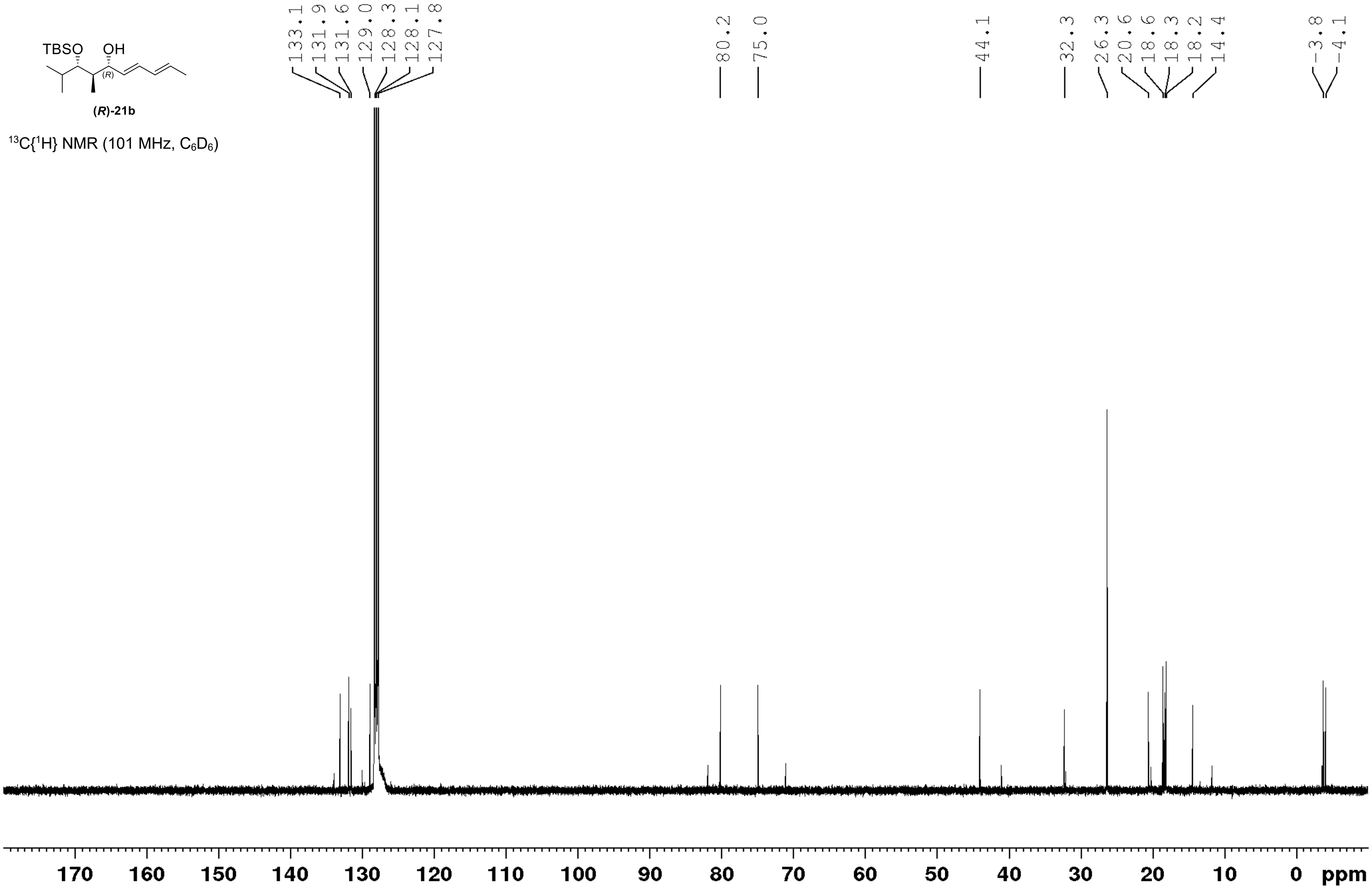

TBSO
OH
(R)
(R)-21b
$^{13}C\{^1H\}$ NMR (101 MHz, C_6D_6)
133.1
131.9
131.6
129.0
128.3
128.1
127.8
80.2
75.0
44.1
32.3
26.3
20.6
18.6
18.3
18.2
14.4
-3.8
-4.1
170
160
150
140
130
120
110
100
90
80
70
60
50
40
30
20
10
0
ppm

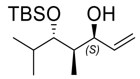

(*S*)-22a

^{1}H NMR (400 MHz, C_6D_6)

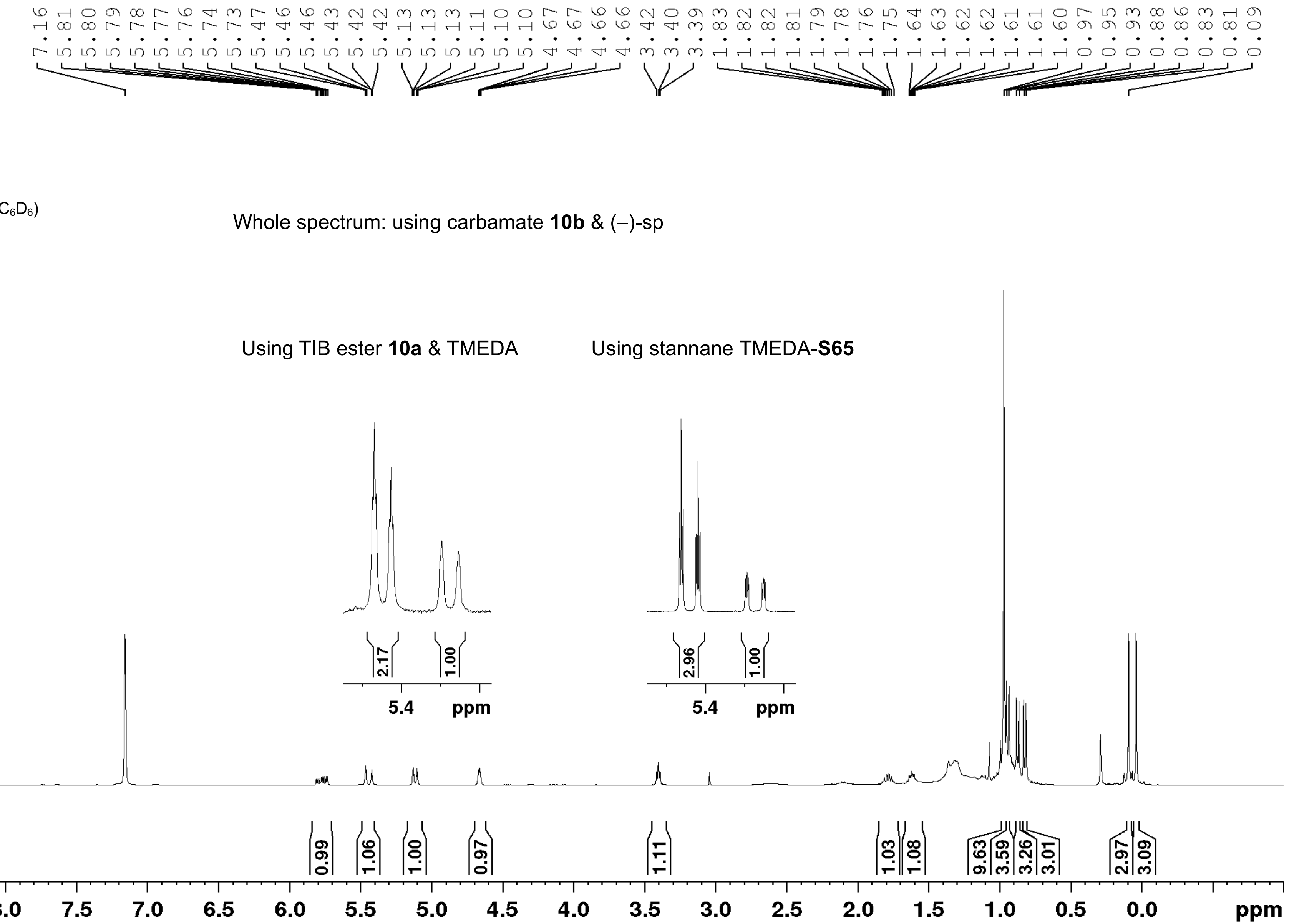

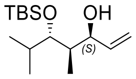

(*S*)-22a

$^{13}C\{^{1}H\}$ NMR (101 MHz, C_6D_6)

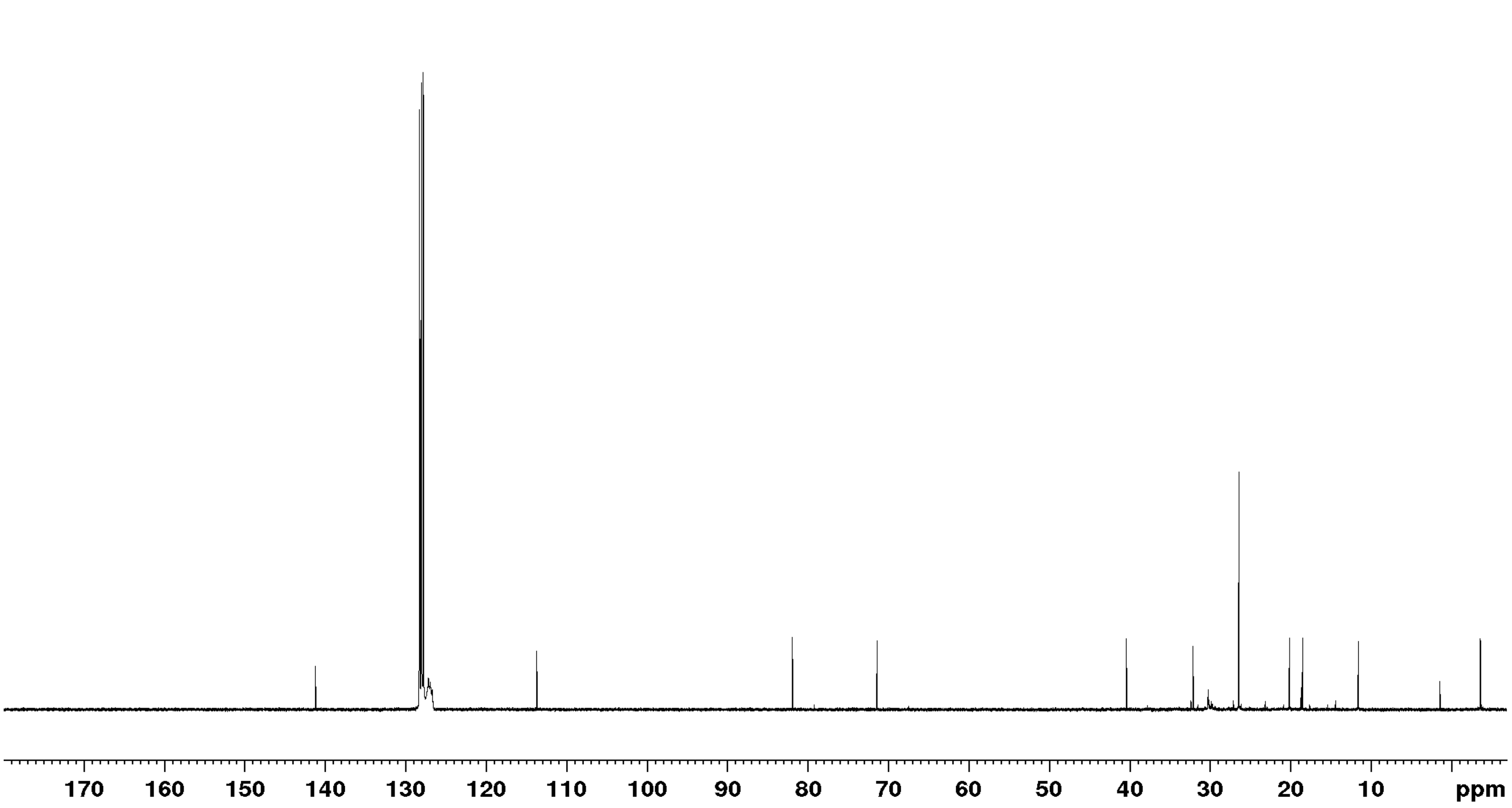

TBSO OH

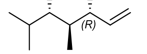

(*R*)-22b

^{1}H NMR (400 MHz, C_6D_6)

Whole spectrum: pure **(*R*)-22b** from using carbamate **10b** & TMEDA

Using stannane TMEDA-**S66**

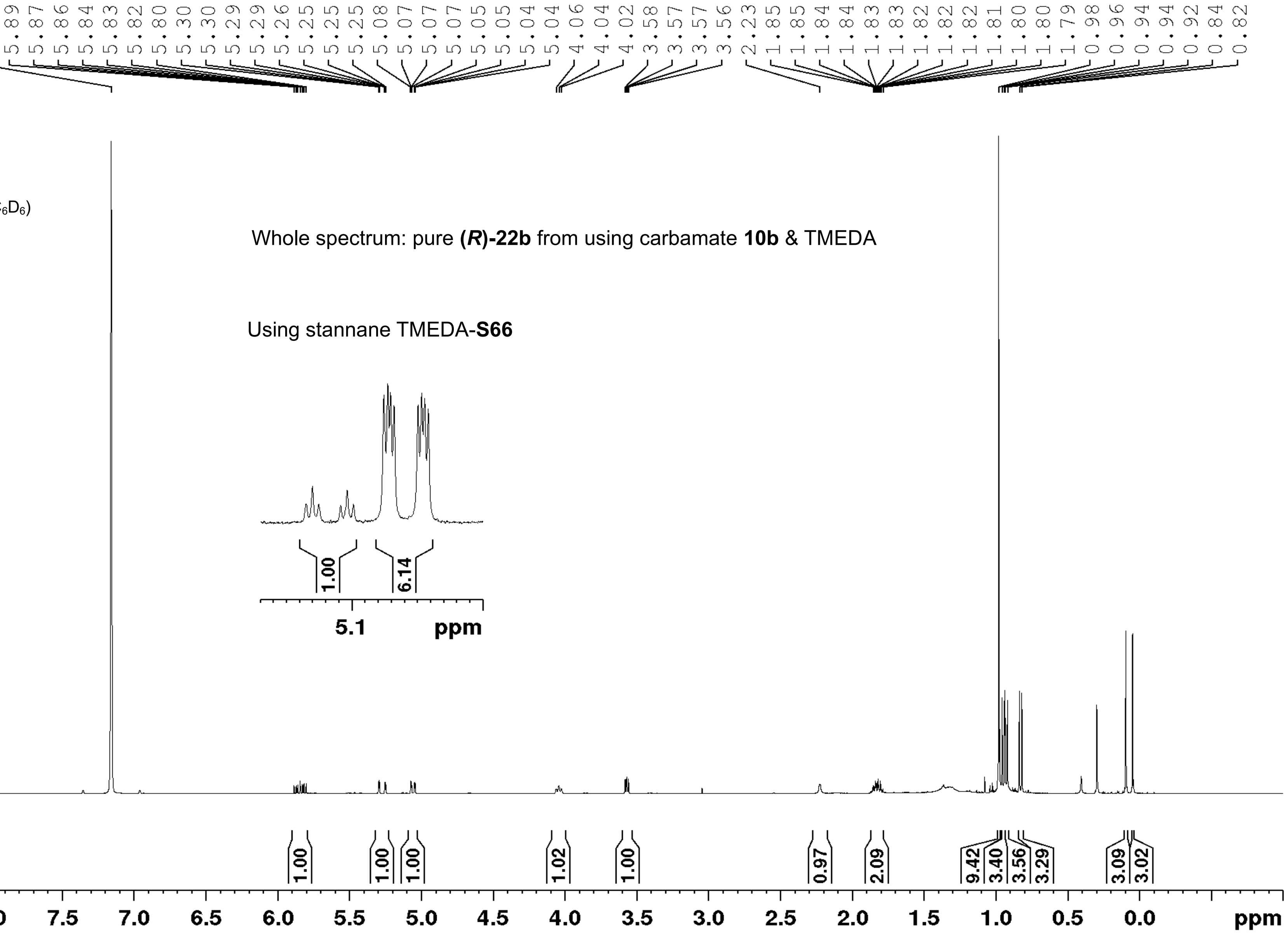

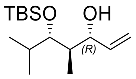

(*R*)-22b

$^{13}C\{^{1}H\}$ NMR (101 MHz, C_6D_6)

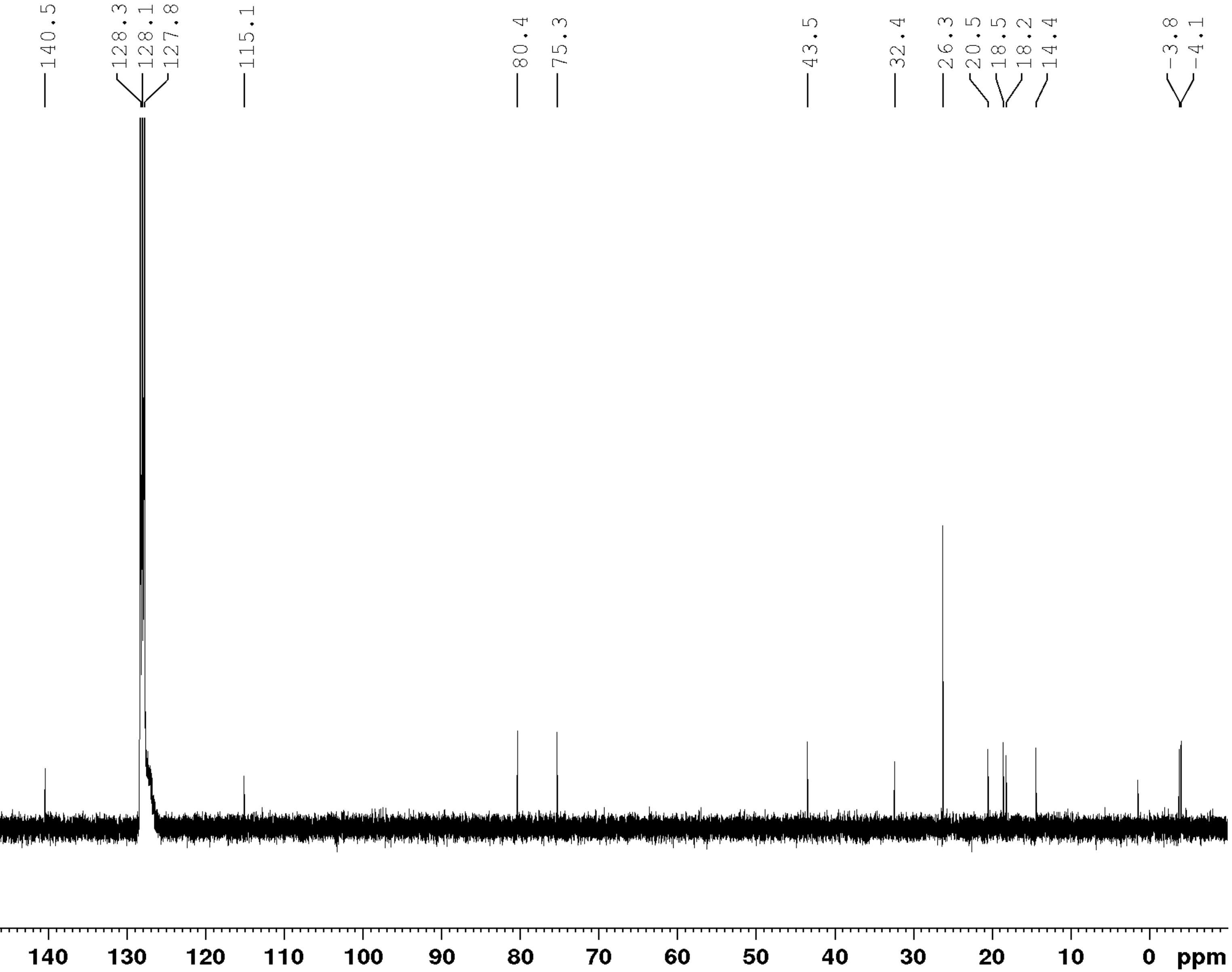

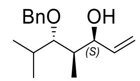

(*S*)-27a

^{1}H NMR (400 MHz, C_6D_6)

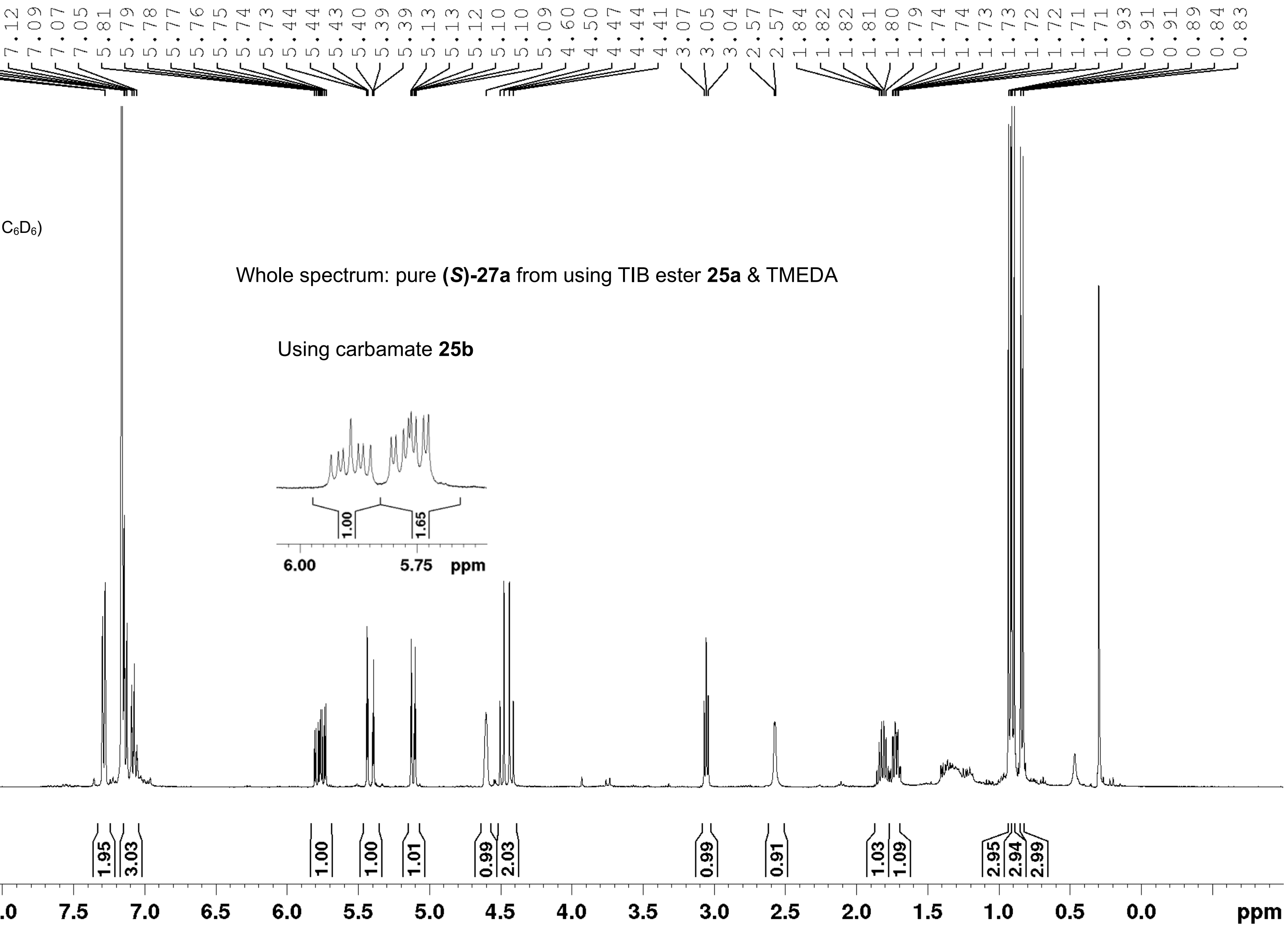

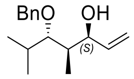

(*S*)-27a

$^{13}C\{^{1}H\}$ NMR (101 MHz, C_6D_6)

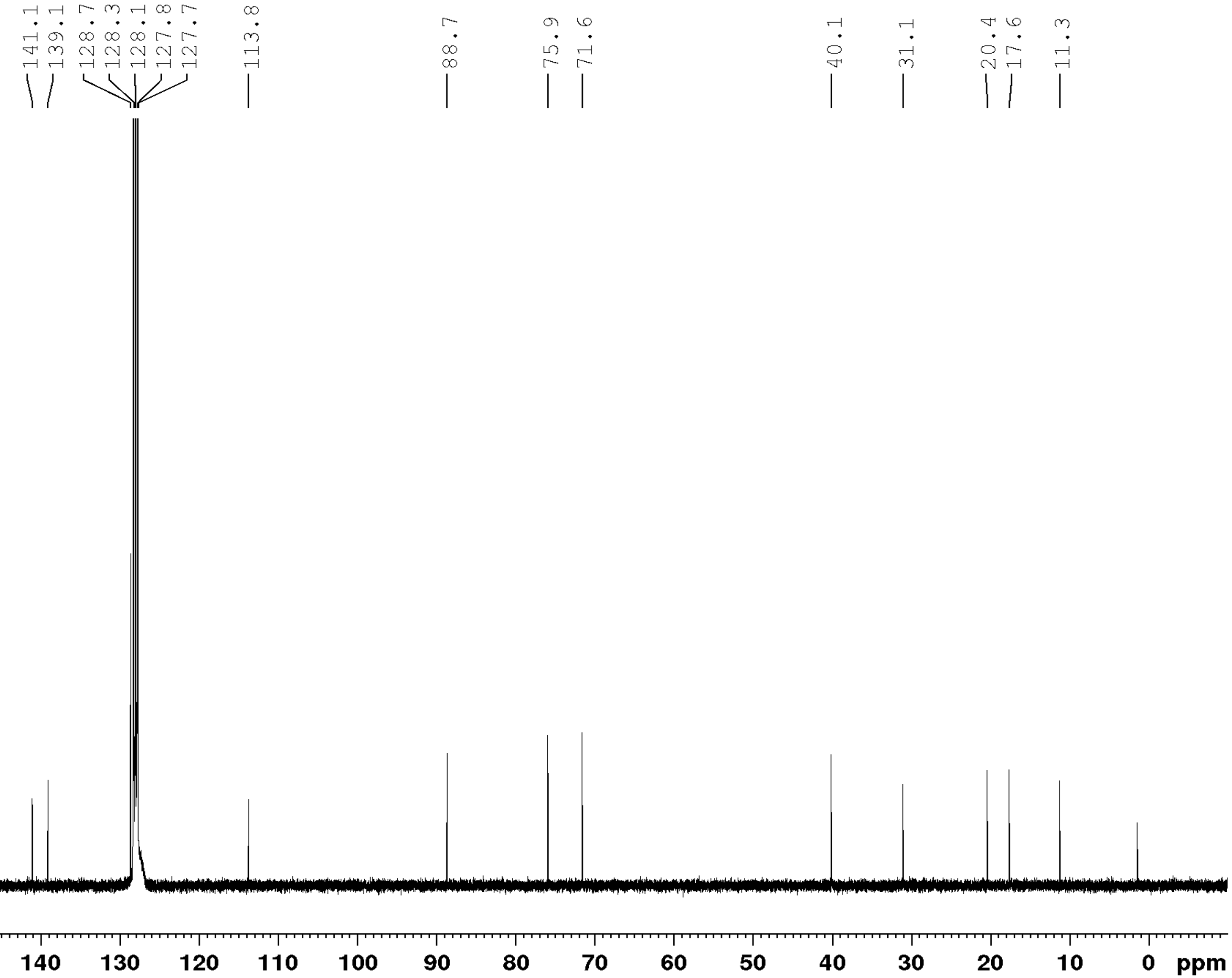

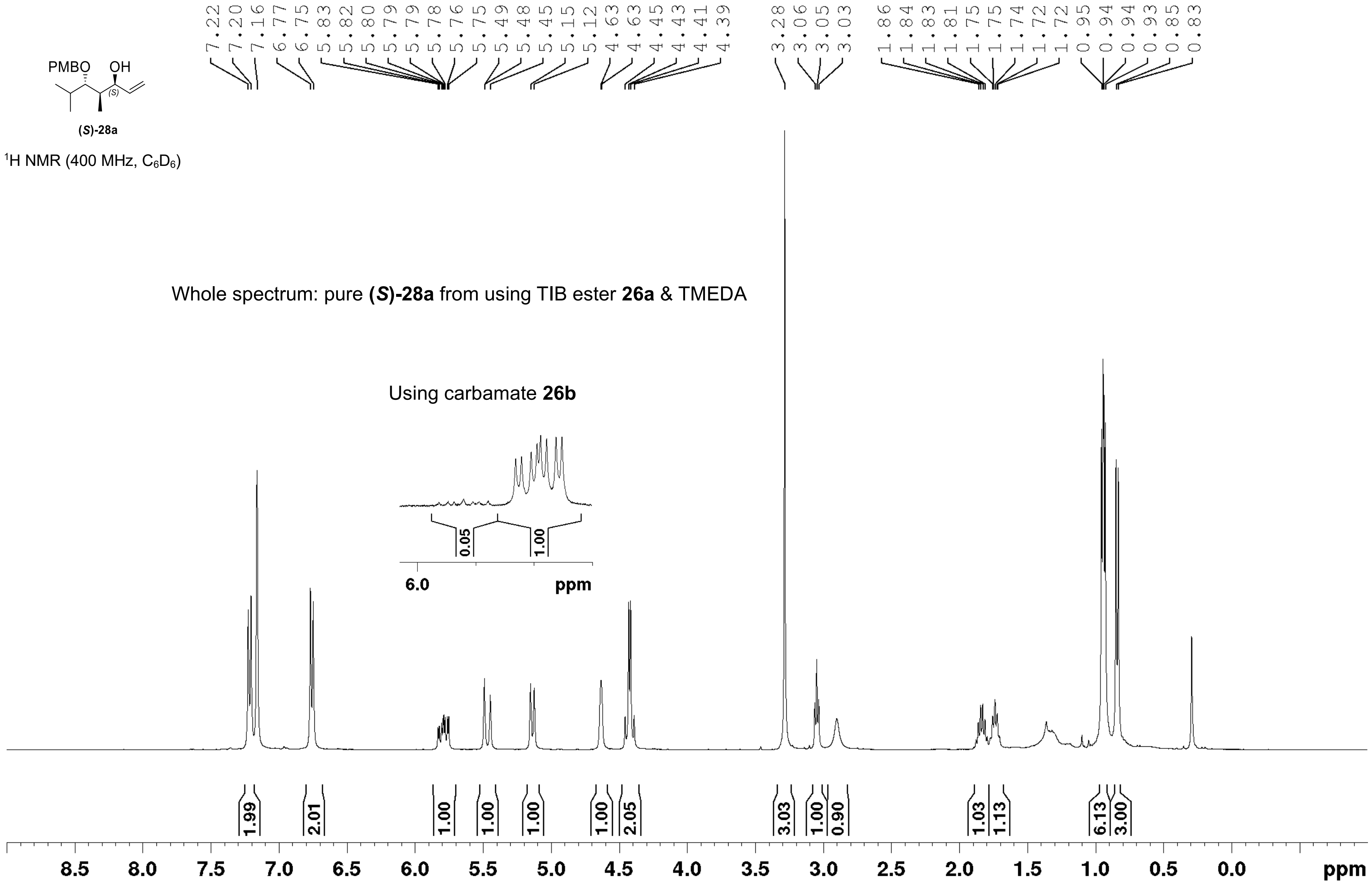

PMBO OH
(S)
(S)-28a
1H NMR (400 MHz, C6D6)
7.22 7.20 7.16 6.77 6.75 5.83 5.82 5.80 5.79 5.79 5.78 5.76 5.75 5.49 5.48 5.45 5.15 5.12 4.63 4.63 4.45 4.43 4.41 4.39 3.28 3.06 3.05 3.03 1.86 1.84 1.83 1.81 1.75 1.75 1.74 1.72 1.72 0.95 0.94 0.94 0.93 0.85 0.83
Whole spectrum: pure (S)-28a from using TIB ester 26a & TMEDA
Using carbamate 26b
0.05
1.00
6.0
ppm
1.99
2.01
1.00
1.00
1.00
1.00
2.05
3.03
1.00
0.90
1.03
1.13
6.13
3.00
8.5 8.0 7.5 7.0 6.5 6.0 5.5 5.0 4.5 4.0 3.5 3.0 2.5 2.0 1.5 1.0 0.5 0.0
ppm

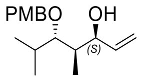

(*S*)-28a

$^{13}C\{^{1}H\}$ NMR (101 MHz, C_6D_6)

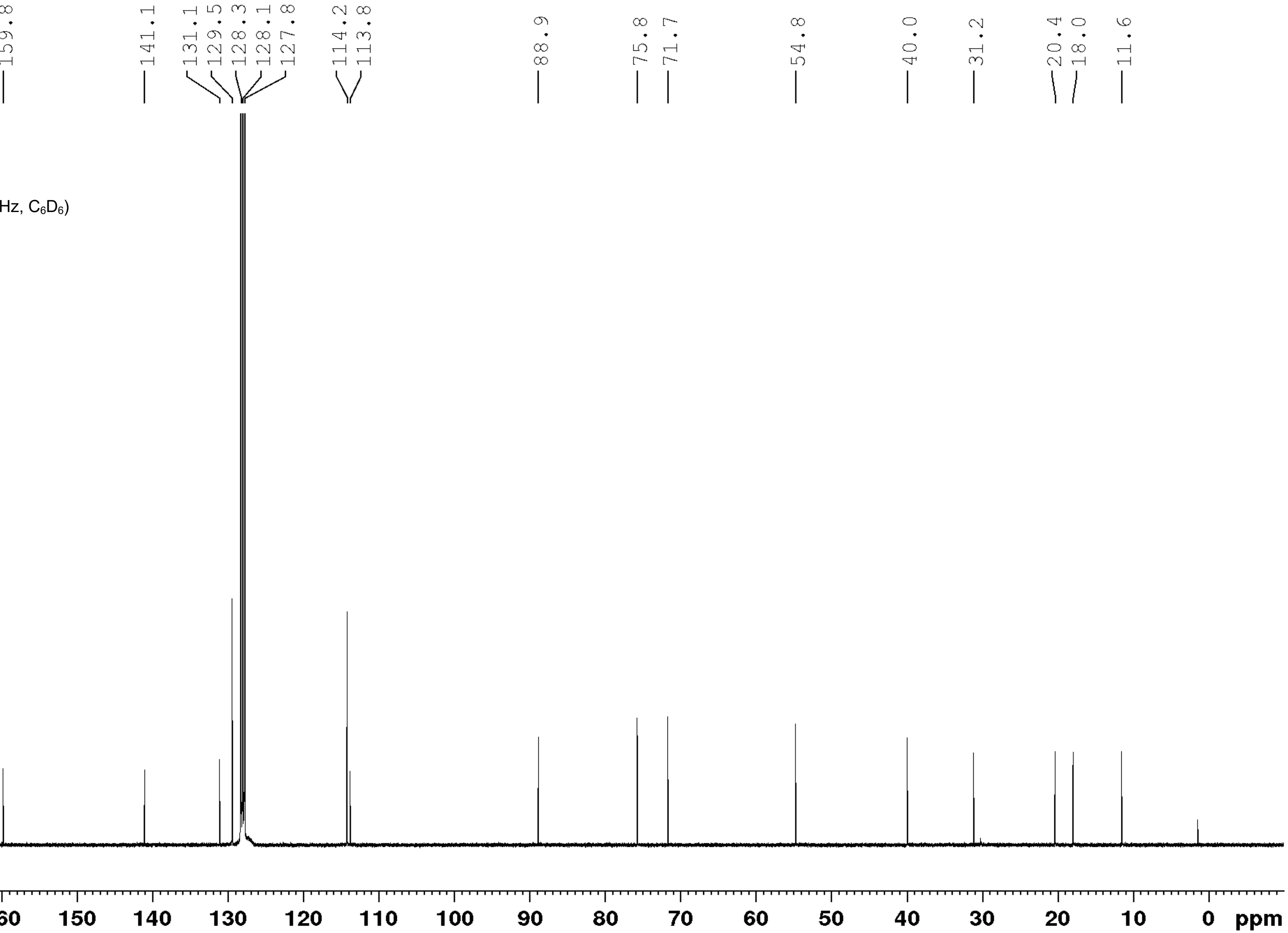

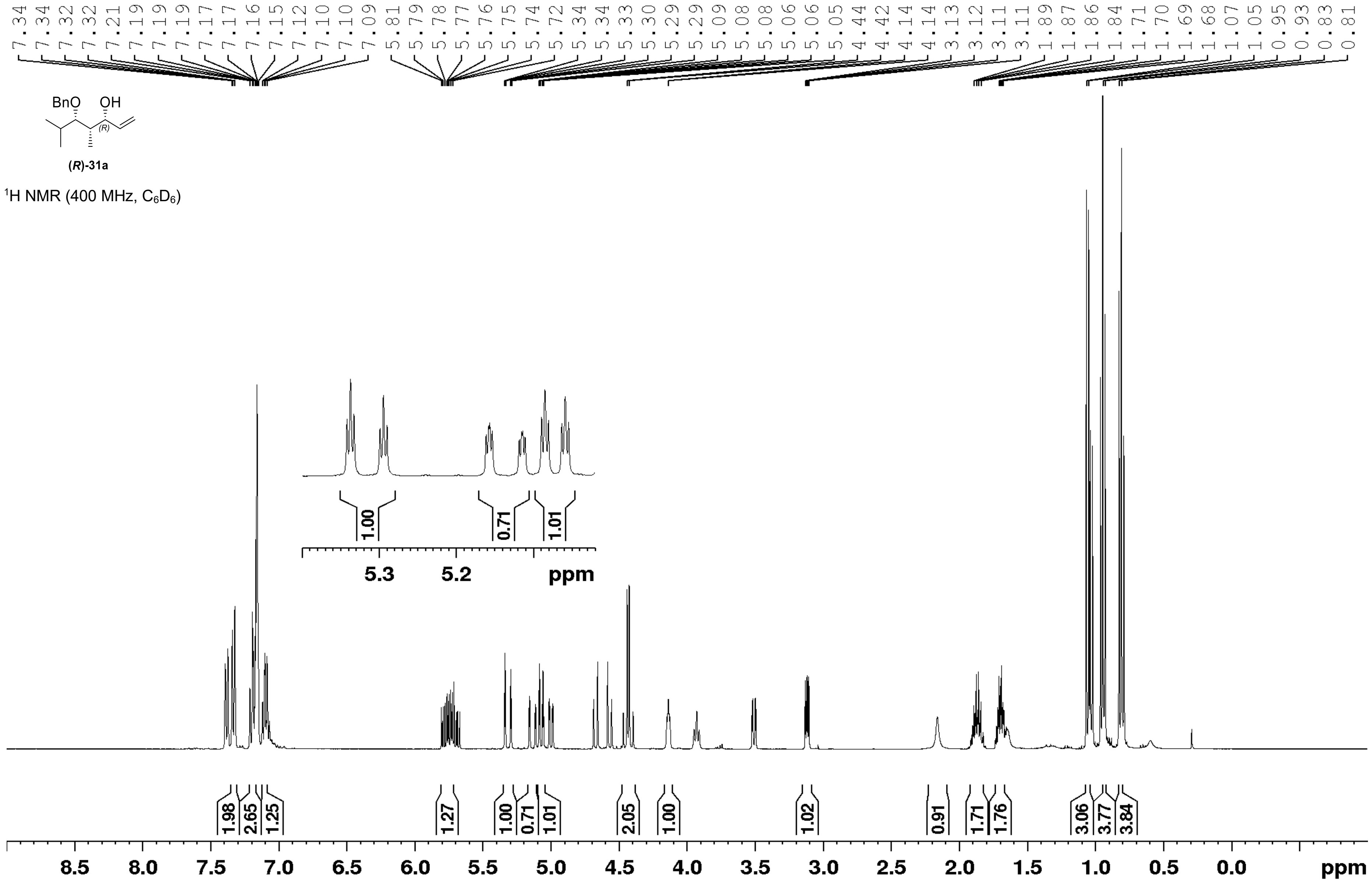
BnO
OH
(R)
(R)-31a
1H NMR (400 MHz, C6D6)
ppm

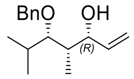

(*R*)-31a

$^{13}C\{^{1}H\}$ NMR (101 MHz, C_6D_6)

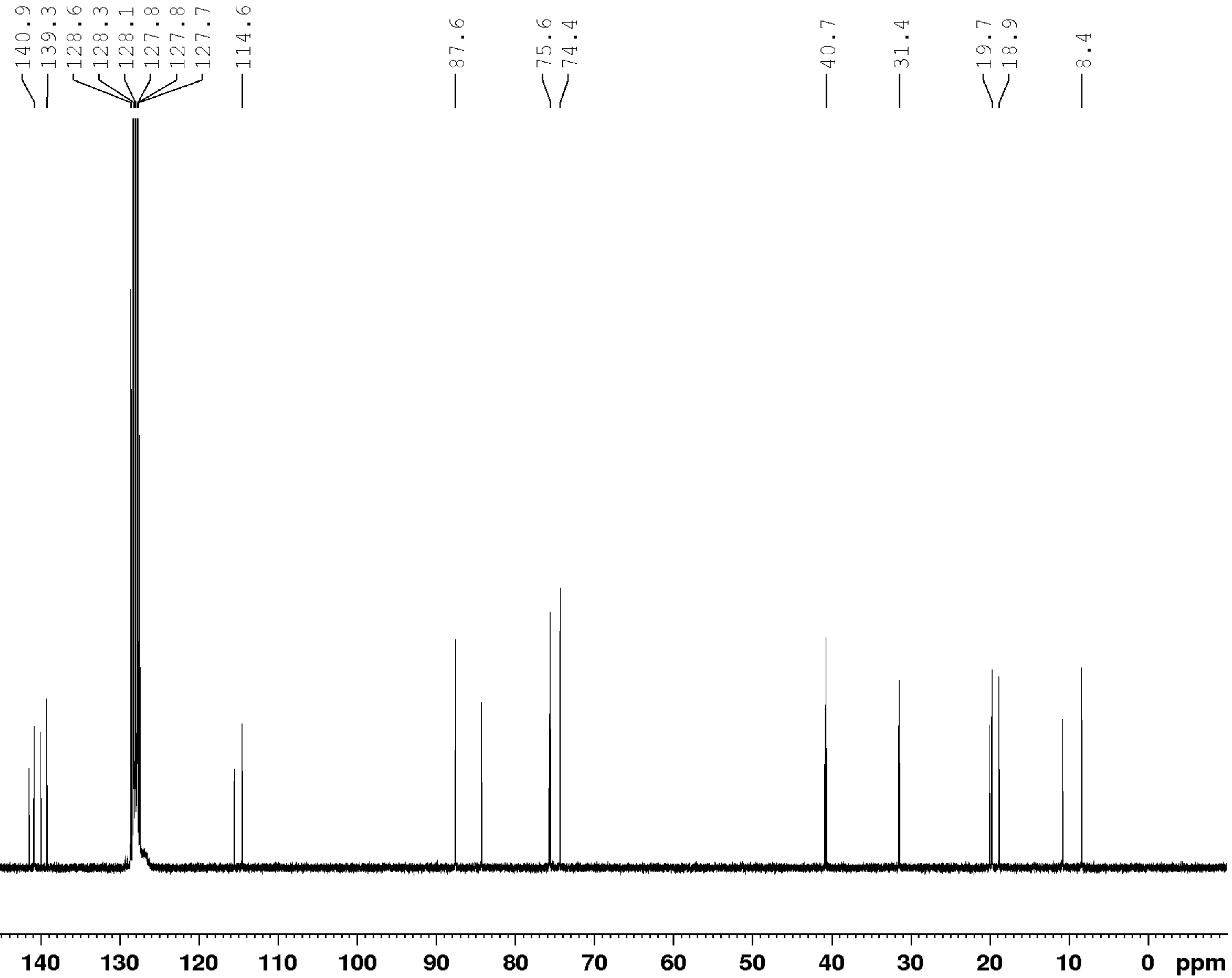

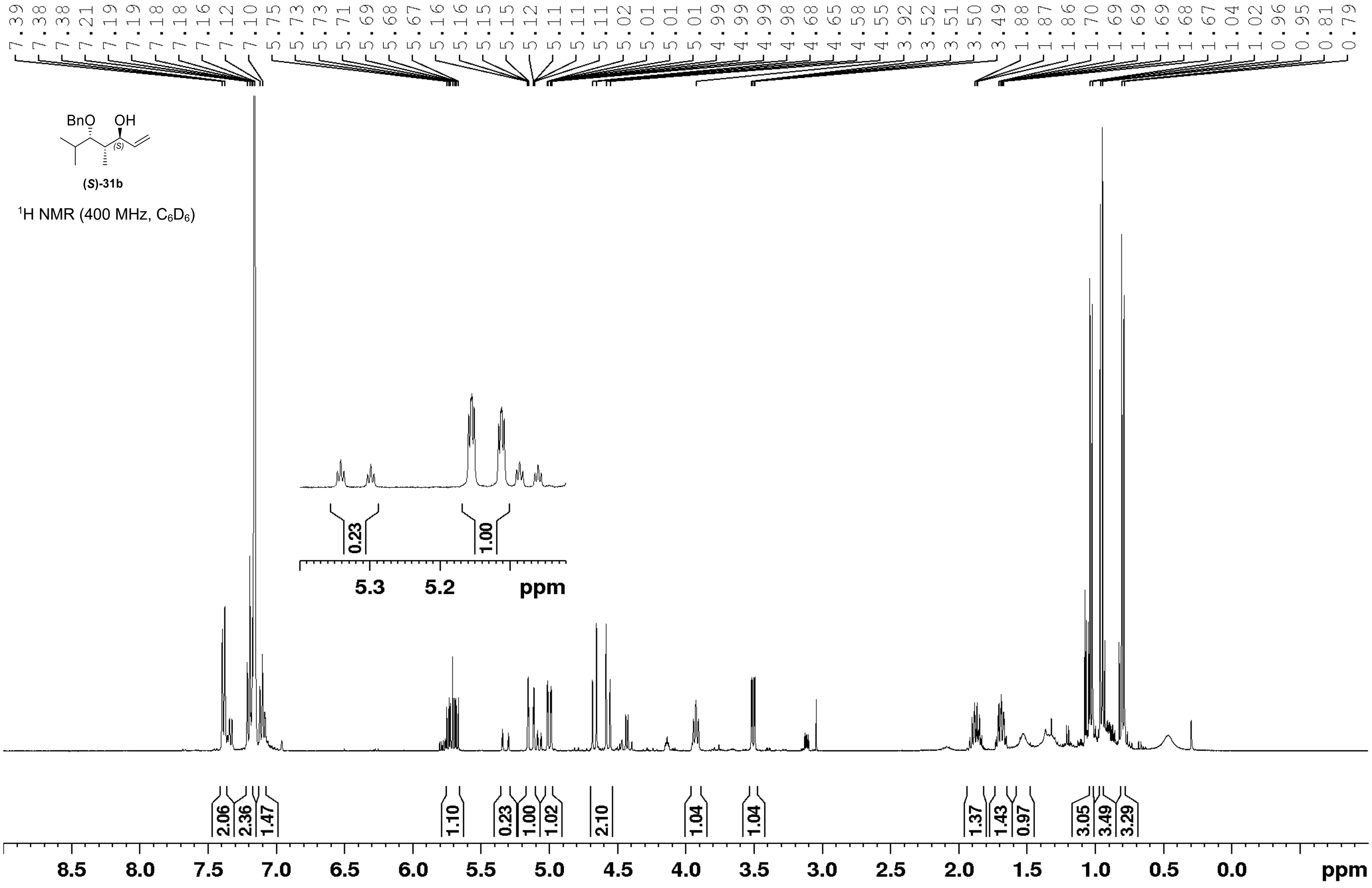

(*S*)-31b

^{1}H NMR (400 MHz, C_6D_6)

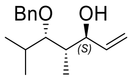

(*S*)-31b

$^{13}C\{^1H\}$ NMR (101 MHz, C_6D_6)

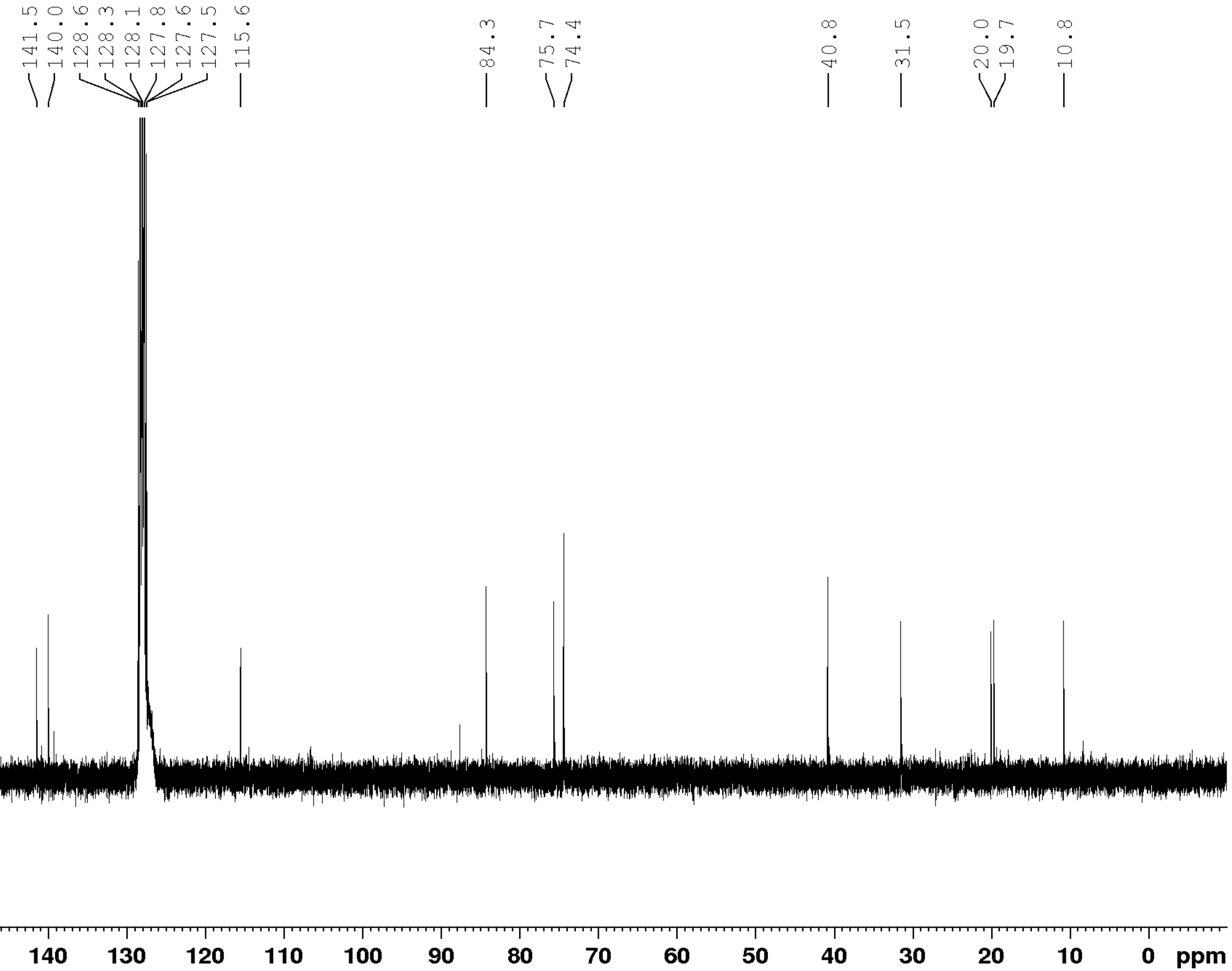

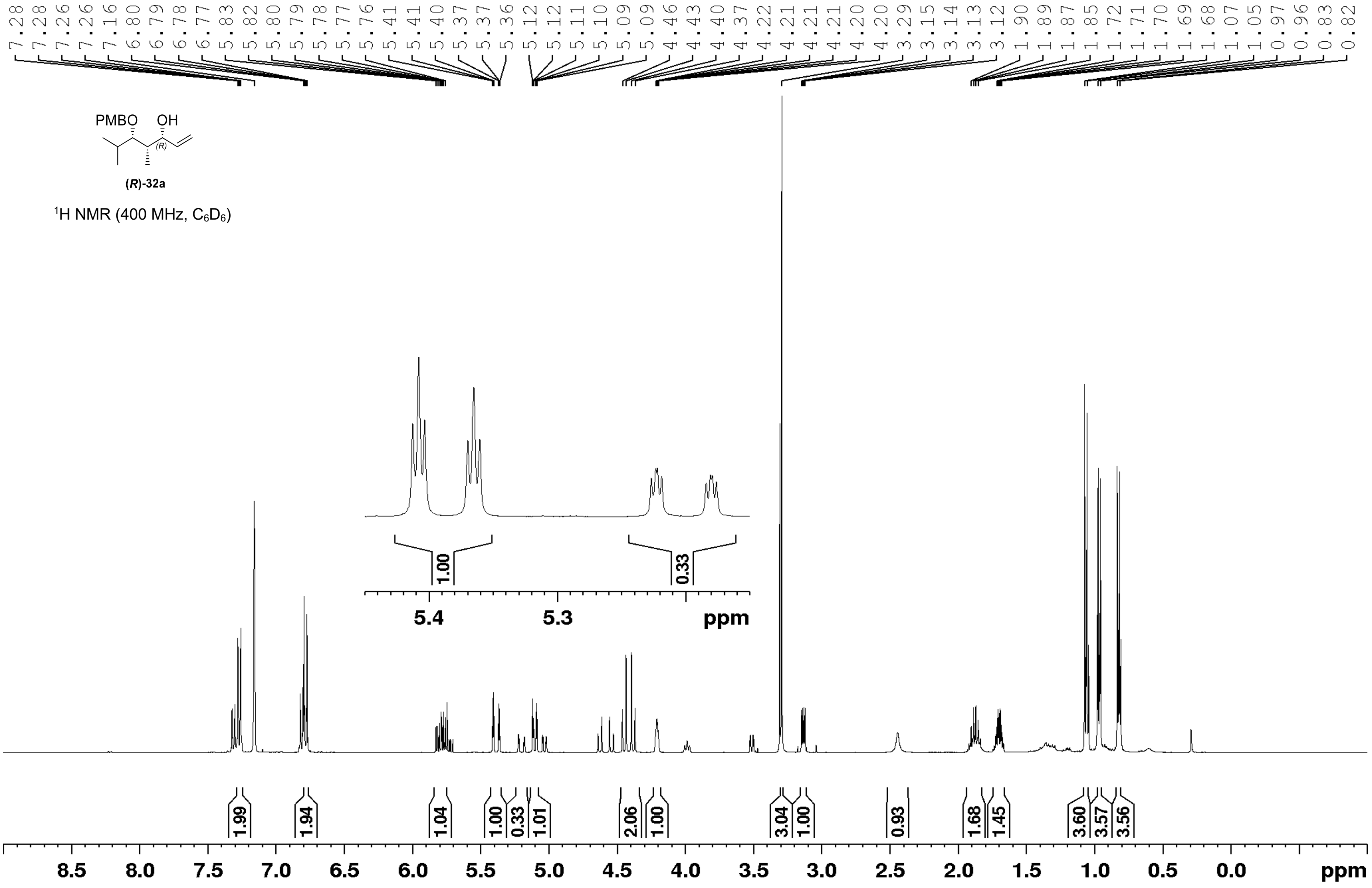
PMBO OH
(R)
(R)-32a
¹H NMR (400 MHz, C6D6)
7.28 7.28 7.26 7.26 7.16 6.80 6.79 6.78 6.77 5.83 5.82 5.80 5.79 5.78 5.77 5.76 5.41 5.41 5.40 5.37 5.37 5.36 5.12 5.12 5.11 5.10 5.09 5.09 4.46 4.43 4.40 4.37 4.22 4.21 4.21 4.21 4.20 4.20 3.29 3.15 3.14 3.13 3.12 1.90 1.89 1.87 1.85 1.72 1.71 1.70 1.69 1.68 1.07 1.05 0.97 0.96 0.83 0.82
1.00
0.33
5.4
5.3
ppm
1.99
1.94
1.04
1.00
0.33
1.01
2.06
1.00
3.04
1.00
0.93
1.68
1.45
3.60
3.57
3.56
8.5 8.0 7.5 7.0 6.5 6.0 5.5 5.0 4.5 4.0 3.5 3.0 2.5 2.0 1.5 1.0 0.5 0.0 ppm

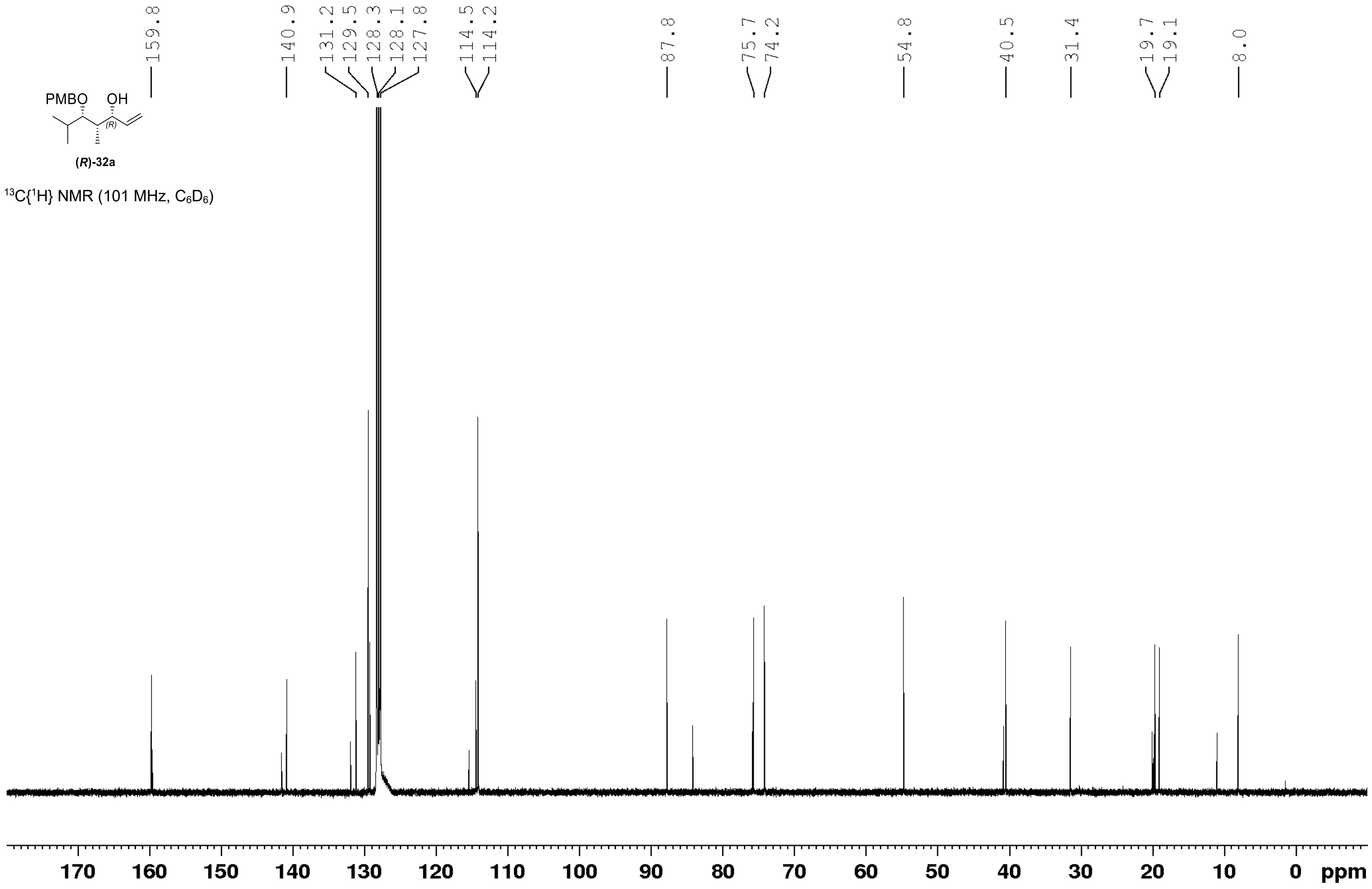
PMBO
OH
(R)
(R)-32a
$^{13}C\{^1H\}$ NMR (101 MHz, C_6D_6)
159.8
140.9
131.2
129.5
128.3
128.1
127.8
114.5
114.2
87.8
75.7
74.2
54.8
40.5
31.4
19.7
19.1
8.0
170
160
150
140
130
120
110
100
90
80
70
60
50
40
30
20
10
0
ppm

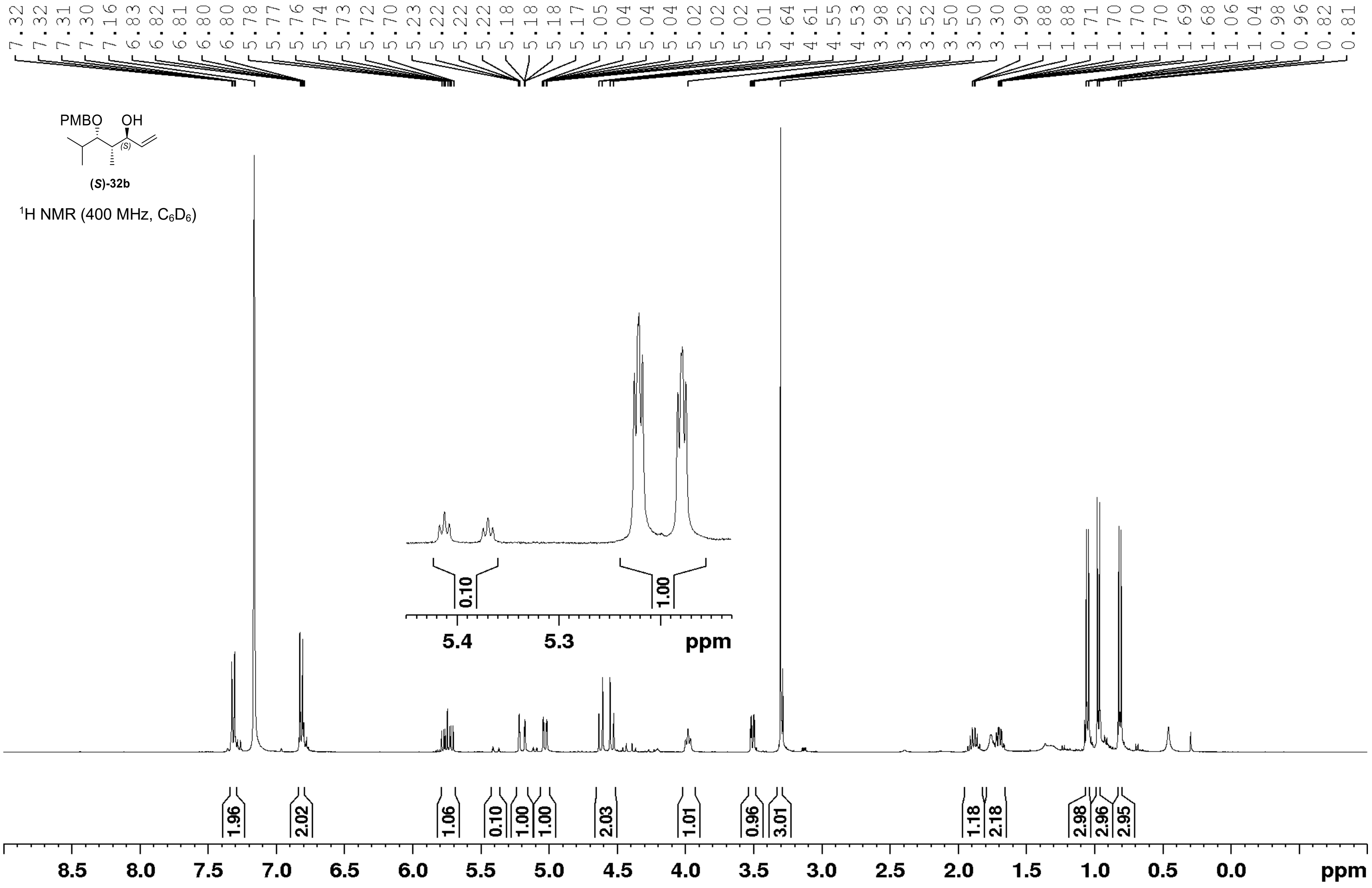
(S)-32b
1H NMR (400 MHz, C6D6)
ppm

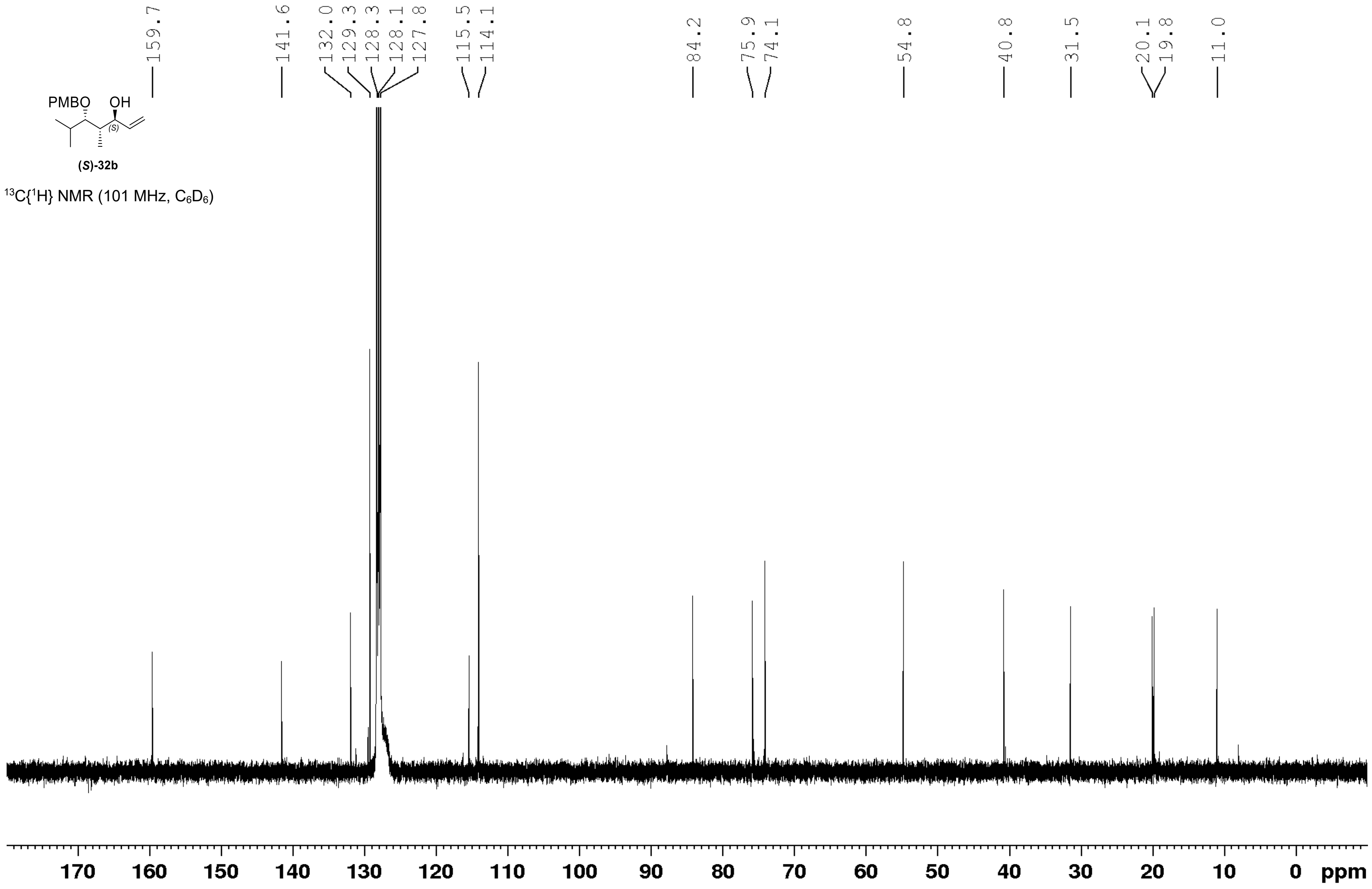
PMBO OH
(S)
(S)-32b
$^{13}C\{^1H\}$ NMR (101 MHz, C_6D_6)
159.7
141.6
132.0
129.3
128.3
128.1
127.8
115.5
114.1
84.2
75.9
74.1
54.8
40.8
31.5
20.1
19.8
11.0
170 160 150 140 130 120 110 100 90 80 70 60 50 40 30 20 10 0 ppm

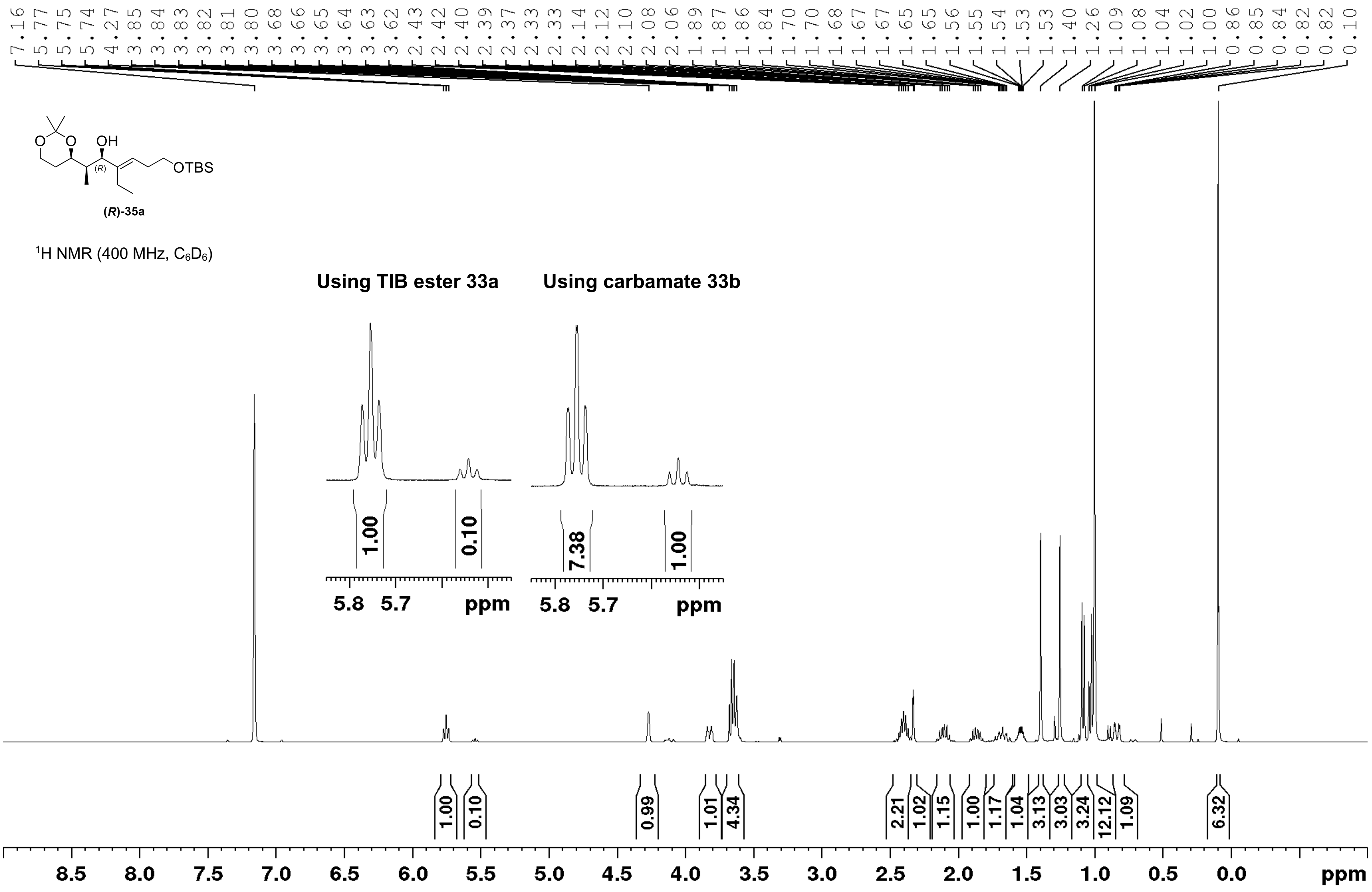
OH
OTBS
(R)
(R)-35a
^{1}H NMR (400 MHz, C_6D_6)
Using TIB ester 33a
Using carbamate 33b
1.00
0.10
7.38
1.00
5.8 5.7 ppm
5.8 5.7 ppm
7.16 5.77 5.75 5.74 4.27 3.85 3.84 3.83 3.82 3.81 3.80 3.68 3.66 3.65 3.64 3.63 3.62 2.43 2.42 2.40 2.39 2.37 2.33 2.33 2.14 2.12 2.10 2.08 2.06 1.89 1.87 1.86 1.84 1.70 1.70 1.68 1.67 1.67 1.65 1.65 1.56 1.55 1.54 1.53 1.53 1.40 1.26 1.09 1.08 1.04 1.02 1.00 0.86 0.85 0.84 0.82 0.82 0.10
1.00 0.10 0.99 1.01 4.34 2.21 1.02 1.15 1.00 1.17 1.04 3.13 3.03 3.24 12.12 1.09 6.32
8.5 8.0 7.5 7.0 6.5 6.0 5.5 5.0 4.5 4.0 3.5 3.0 2.5 2.0 1.5 1.0 0.5 0.0 ppm

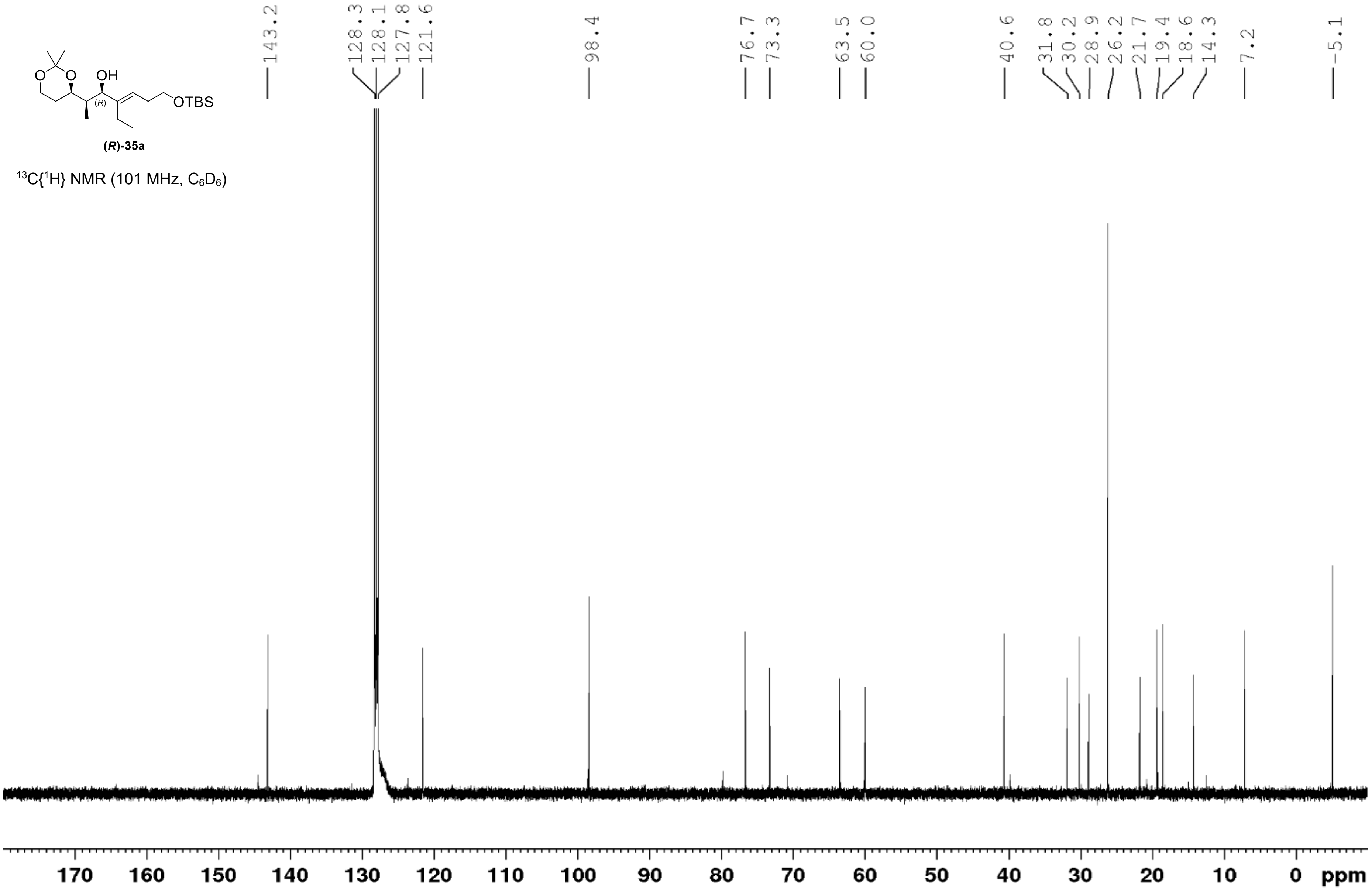

OH
OTBS
(R)
O
O
(R)-35a
$^{13}C\{^{1}H\}$ NMR (101 MHz, C_6D_6)
143.2
128.3
128.1
127.8
121.6
98.4
76.7
73.3
63.5
60.0
40.6
31.8
30.2
28.9
26.2
21.7
19.4
18.6
14.3
7.2
-5.1
170 160 150 140 130 120 110 100 90 80 70 60 50 40 30 20 10 0 ppm

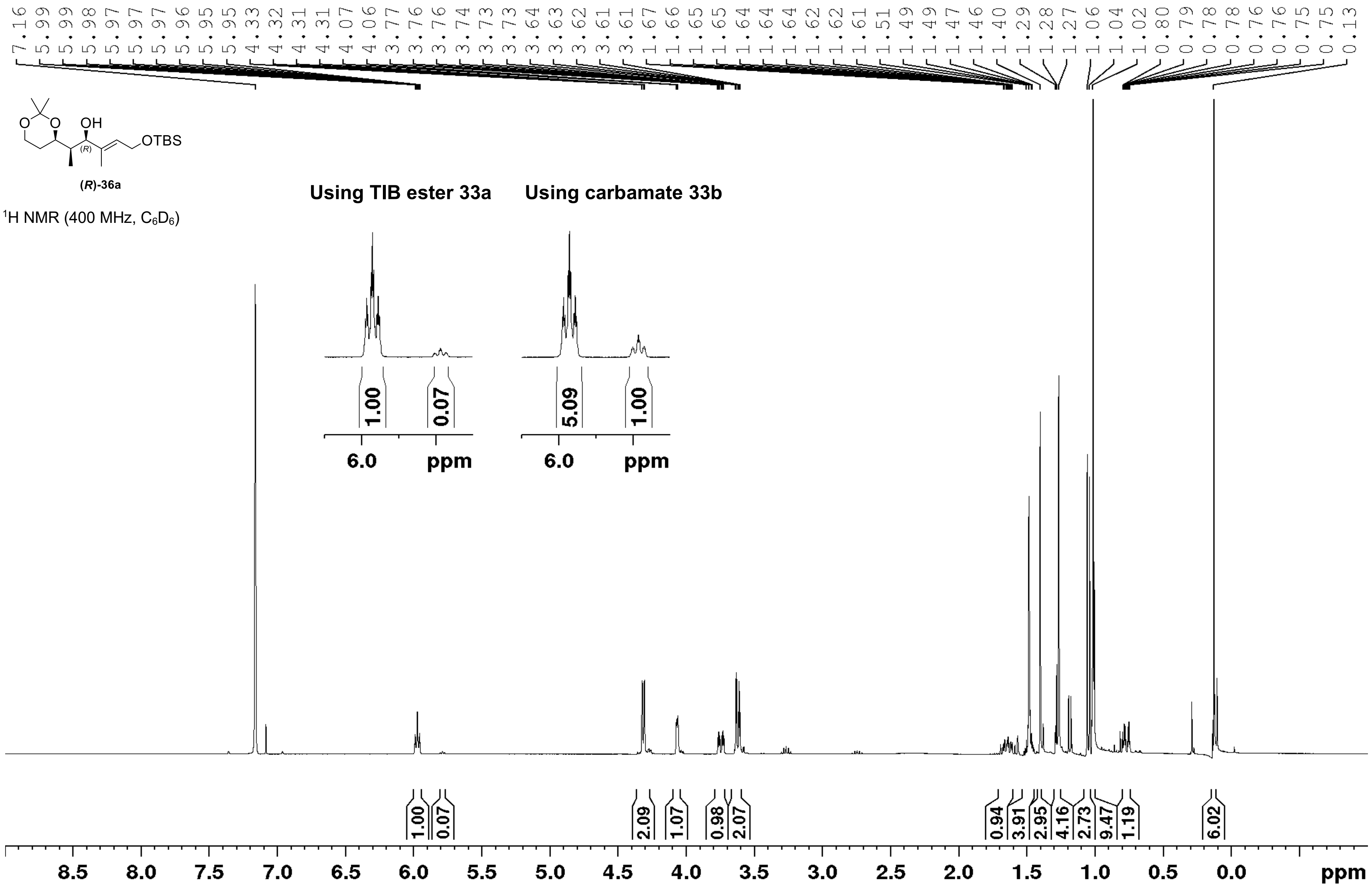
7.16 5.99 5.99 5.98 5.97 5.97 5.97 5.96 5.95 5.95 4.33 4.32 4.31 4.31 4.07 4.06 3.77 3.76 3.76 3.74 3.73 3.73 3.64 3.63 3.62 3.61 3.61 1.67 1.66 1.65 1.65 1.64 1.64 1.64 1.62 1.62 1.61 1.51 1.49 1.49 1.47 1.46 1.40 1.29 1.28 1.27 1.06 1.04 1.02 0.80 0.79 0.78 0.78 0.76 0.76 0.75 0.75 0.13
OH
OTBS
(R)-36a
1H NMR (400 MHz, C6D6)
Using TIB ester 33a
1.00 0.07
6.0 ppm
Using carbamate 33b
5.09 1.00
6.0 ppm
1.00 0.07 2.09 1.07 0.98 2.07 0.94 3.91 2.95 4.16 2.73 9.47 1.19 6.02
8.5 8.0 7.5 7.0 6.5 6.0 5.5 5.0 4.5 4.0 3.5 3.0 2.5 2.0 1.5 1.0 0.5 0.0 ppm

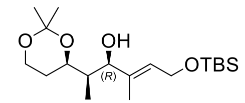

(*R*)-36a

$^{13}C\{^{1}H\}$ NMR (101 MHz, C_6D_6)

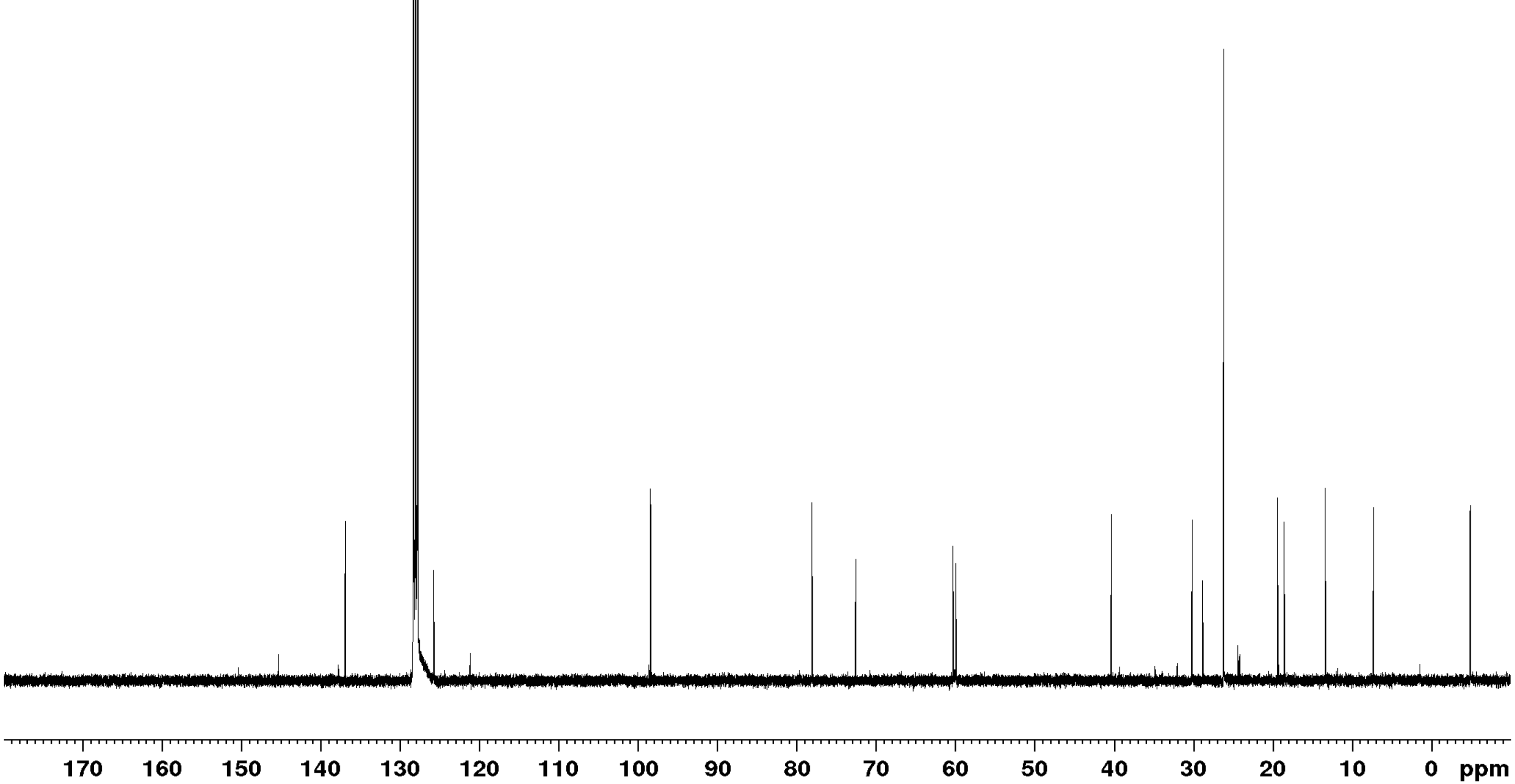

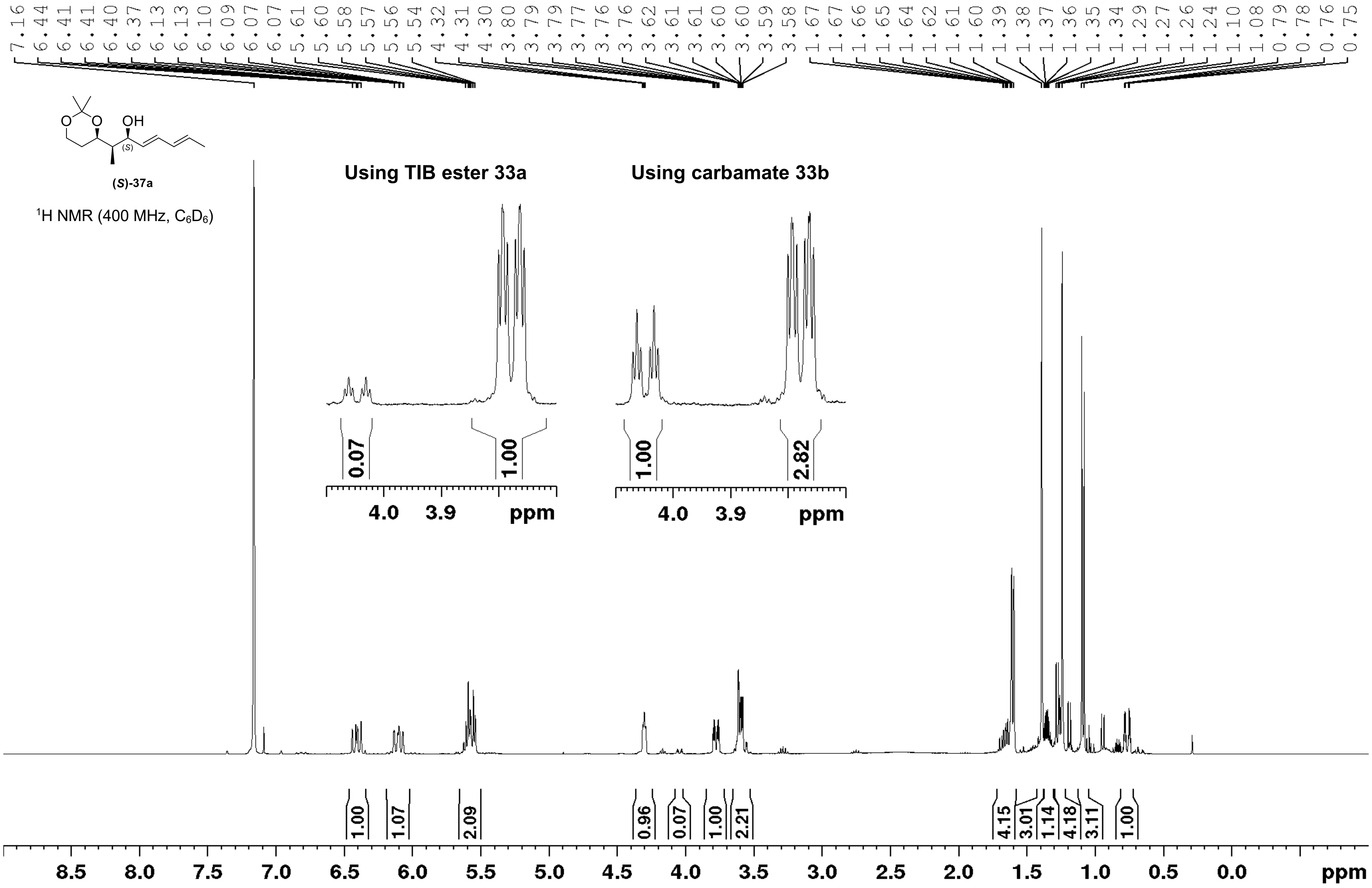
7.16 6.44 6.41 6.41 6.40 6.37 6.13 6.13 6.10 6.09 6.07 6.07 5.61 5.60 5.58 5.57 5.56 5.54 4.32 4.31 4.30 3.80 3.79 3.79 3.77 3.76 3.76 3.62 3.61 3.61 3.60 3.60 3.59 3.58 1.67 1.67 1.66 1.65 1.64 1.62 1.61 1.60 1.39 1.38 1.37 1.36 1.35 1.34 1.29 1.27 1.26 1.24 1.10 1.08 0.79 0.78 0.76 0.75
OH
(S)
(S)-37a
1H NMR (400 MHz, C6D6)
Using TIB ester 33a
0.07
1.00
4.0
3.9
ppm
Using carbamate 33b
1.00
2.82
4.0
3.9
ppm
1.00
1.07
2.09
0.96
0.07
1.00
2.21
4.15
3.01
1.14
4.18
3.11
1.00
8.5
8.0
7.5
7.0
6.5
6.0
5.5
5.0
4.5
4.0
3.5
3.0
2.5
2.0
1.5
1.0
0.5
0.0
ppm

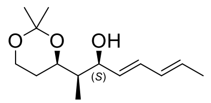

(*S*)-37a

$^{13}C\{^1H\}$ NMR (101 MHz, C_6D_6)

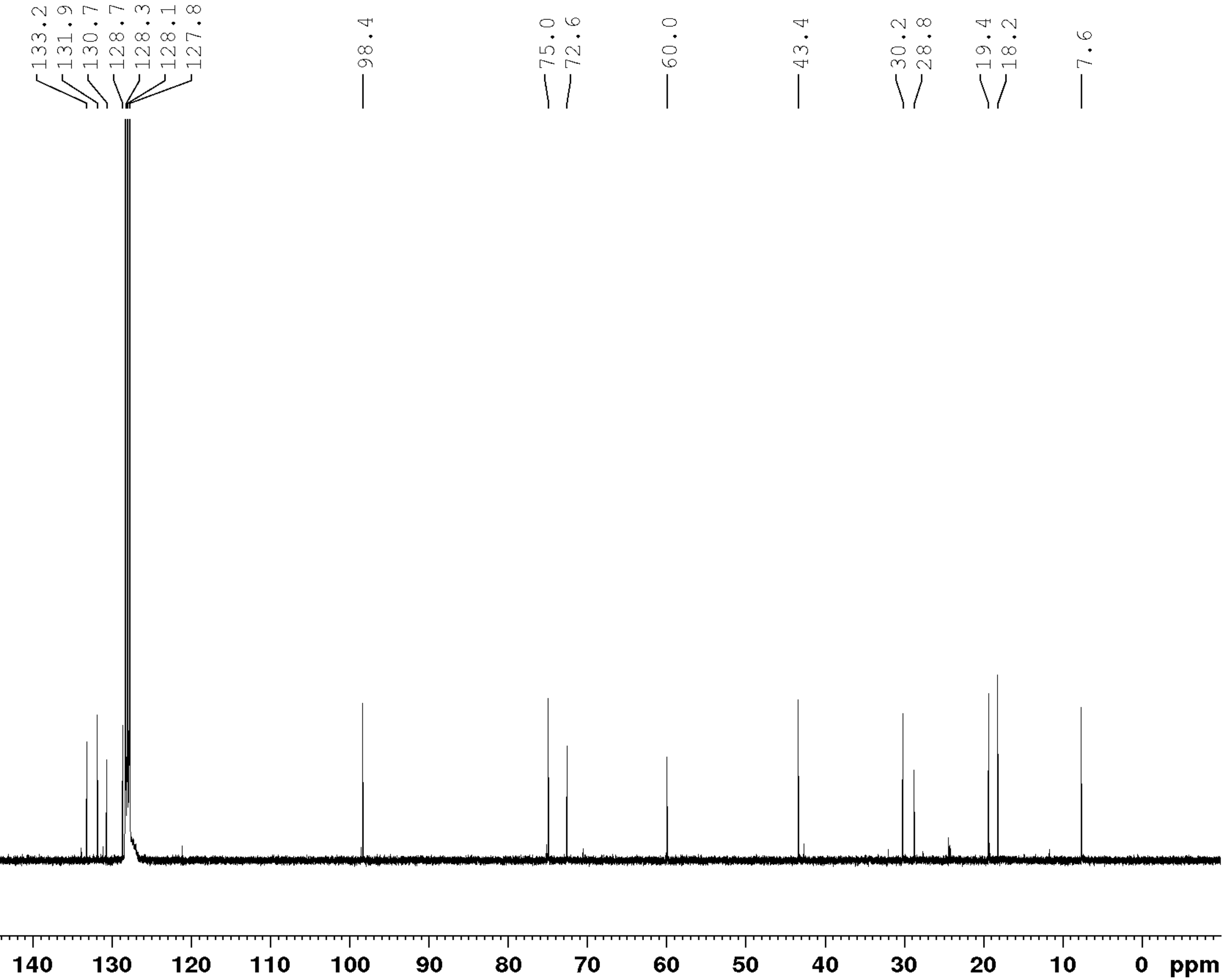

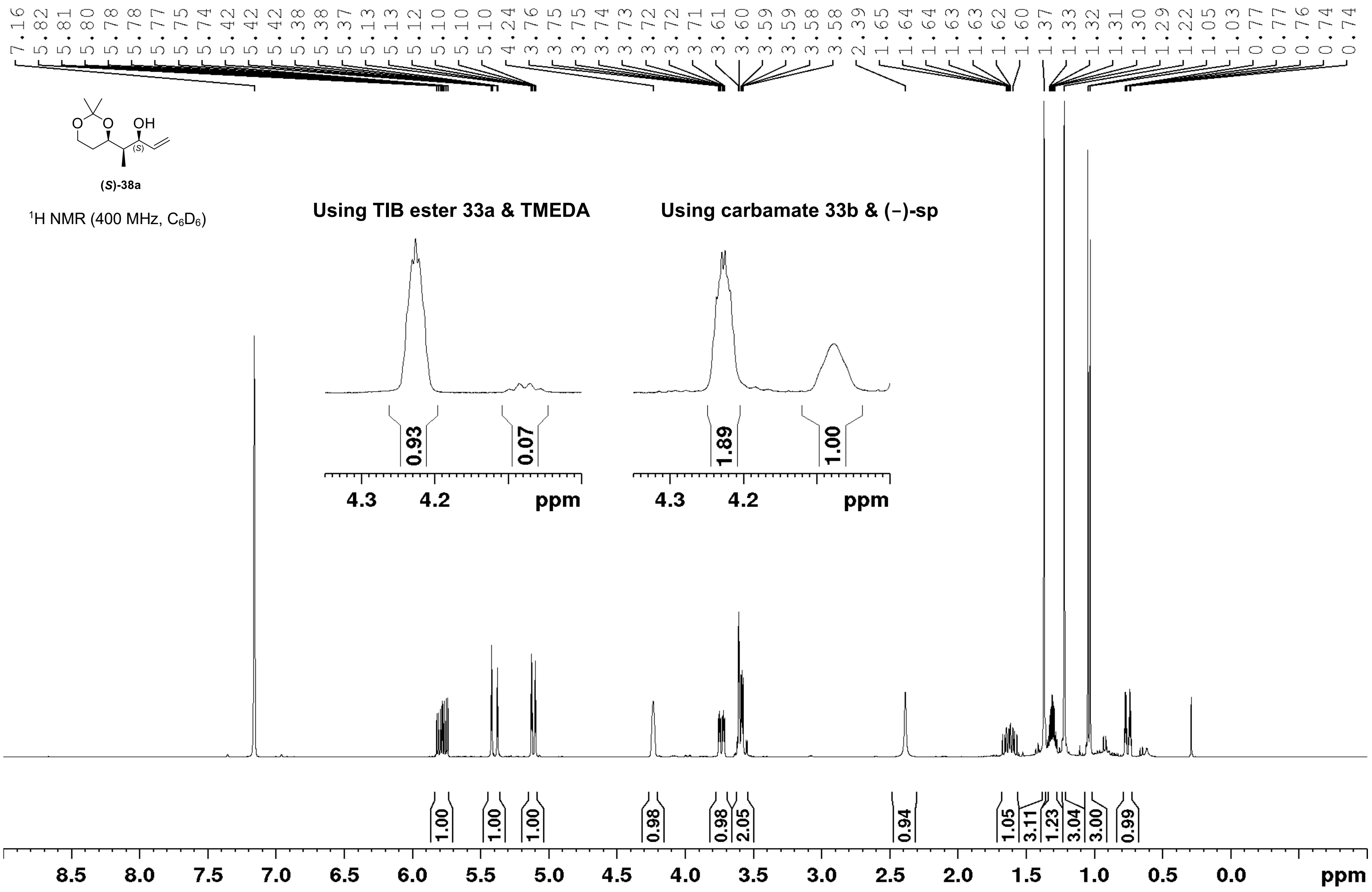
(S)-38a
1H NMR (400 MHz, C6D6)
Using TIB ester 33a & TMEDA
Using carbamate 33b & (−)-sp
0.93
0.07
1.89
1.00
4.3
4.2
ppm
7.16
5.82
5.81
5.80
5.78
5.78
5.77
5.75
5.74
5.42
5.42
5.42
5.38
5.38
5.37
5.13
5.13
5.12
5.10
5.10
5.10
4.24
3.76
3.75
3.75
3.74
3.73
3.72
3.72
3.71
3.61
3.60
3.59
3.59
3.58
3.58
2.39
1.65
1.64
1.64
1.63
1.63
1.62
1.60
1.37
1.33
1.32
1.31
1.30
1.29
1.22
1.05
1.03
0.77
0.77
0.76
0.74
0.74
1.00
1.00
1.00
0.98
0.98
2.05
0.94
1.05
3.11
1.23
3.04
3.00
0.99
8.5
8.0
7.5
7.0
6.5
6.0
5.5
5.0
4.5
4.0
3.5
3.0
2.5
2.0
1.5
1.0
0.5
0.0
ppm

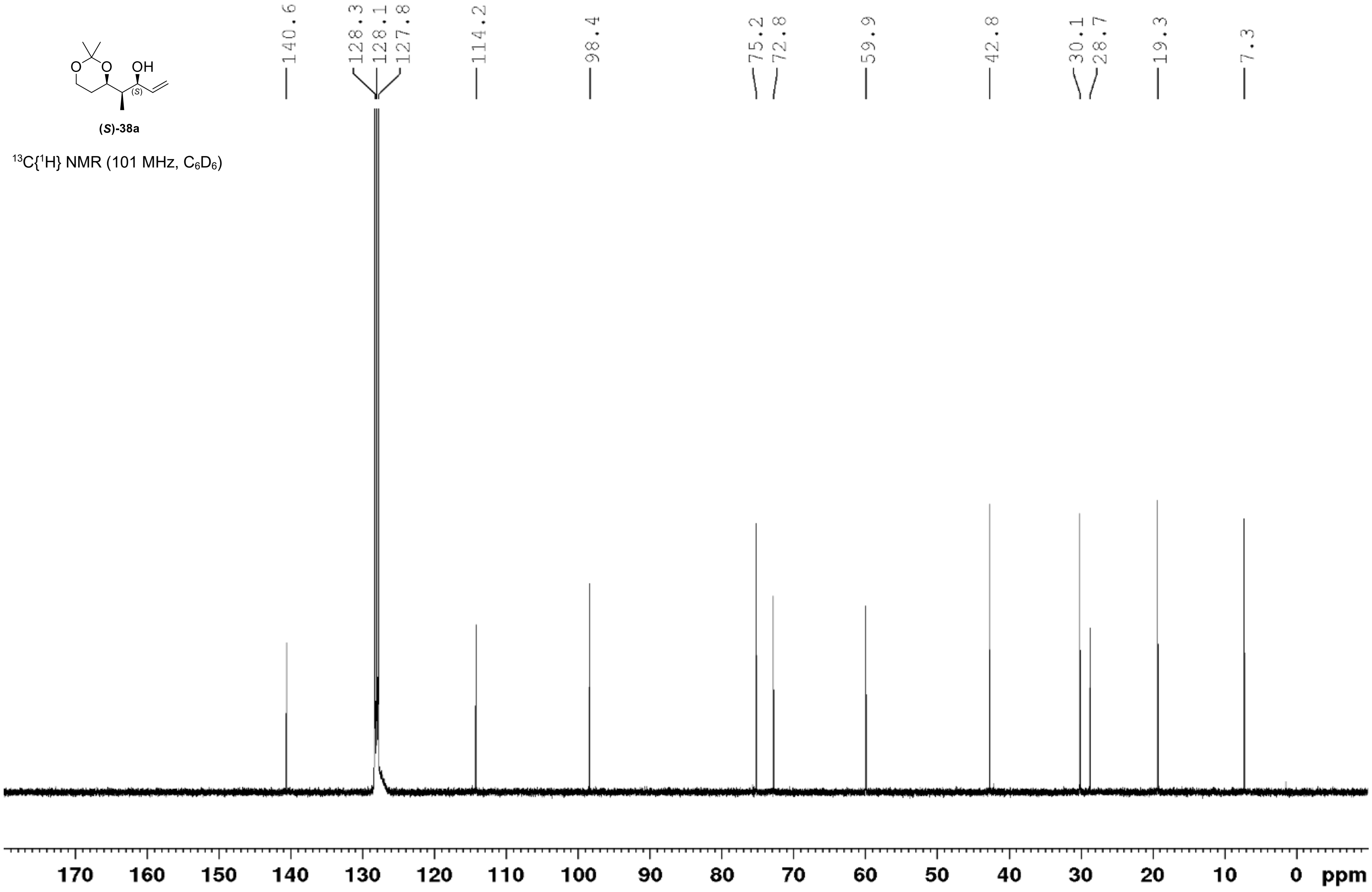

OH
(S)
(S)-38a
$^{13}C\{^{1}H\}$ NMR (101 MHz, C_6D_6)
140.6
128.3
128.1
127.8
114.2
98.4
75.2
72.8
59.9
42.8
30.1
28.7
19.3
7.3
170 160 150 140 130 120 110 100 90 80 70 60 50 40 30 20 10 0 ppm

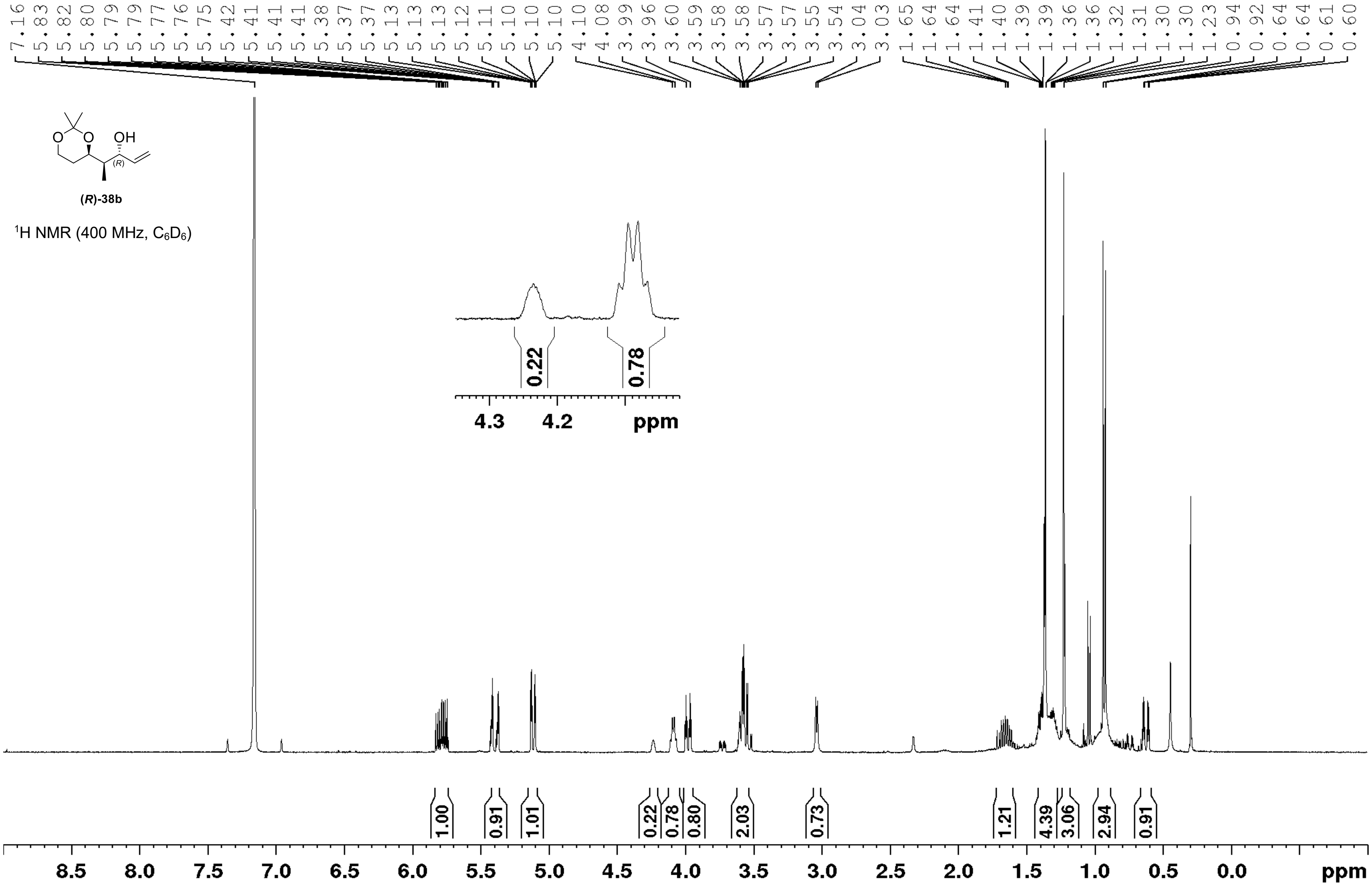
(R)-38b
^{1}H NMR (400 MHz, C_6D_6)
7.16 5.83 5.82 5.80 5.79 5.79 5.77 5.76 5.75 5.42 5.41 5.41 5.41 5.38 5.37 5.37 5.13 5.13 5.13 5.12 5.11 5.10 5.10 5.10 4.10 4.08 3.99 3.96 3.60 3.59 3.58 3.58 3.57 3.57 3.55 3.54 3.04 3.03 1.65 1.64 1.64 1.41 1.40 1.39 1.39 1.36 1.36 1.32 1.31 1.30 1.30 1.23 0.94 0.92 0.64 0.64 0.61 0.60
1.00 0.91 1.01 0.22 0.78 0.80 2.03 0.73 1.21 4.39 3.06 2.94 0.91
0.22 0.78
4.3 4.2 ppm
8.5 8.0 7.5 7.0 6.5 6.0 5.5 5.0 4.5 4.0 3.5 3.0 2.5 2.0 1.5 1.0 0.5 0.0 ppm

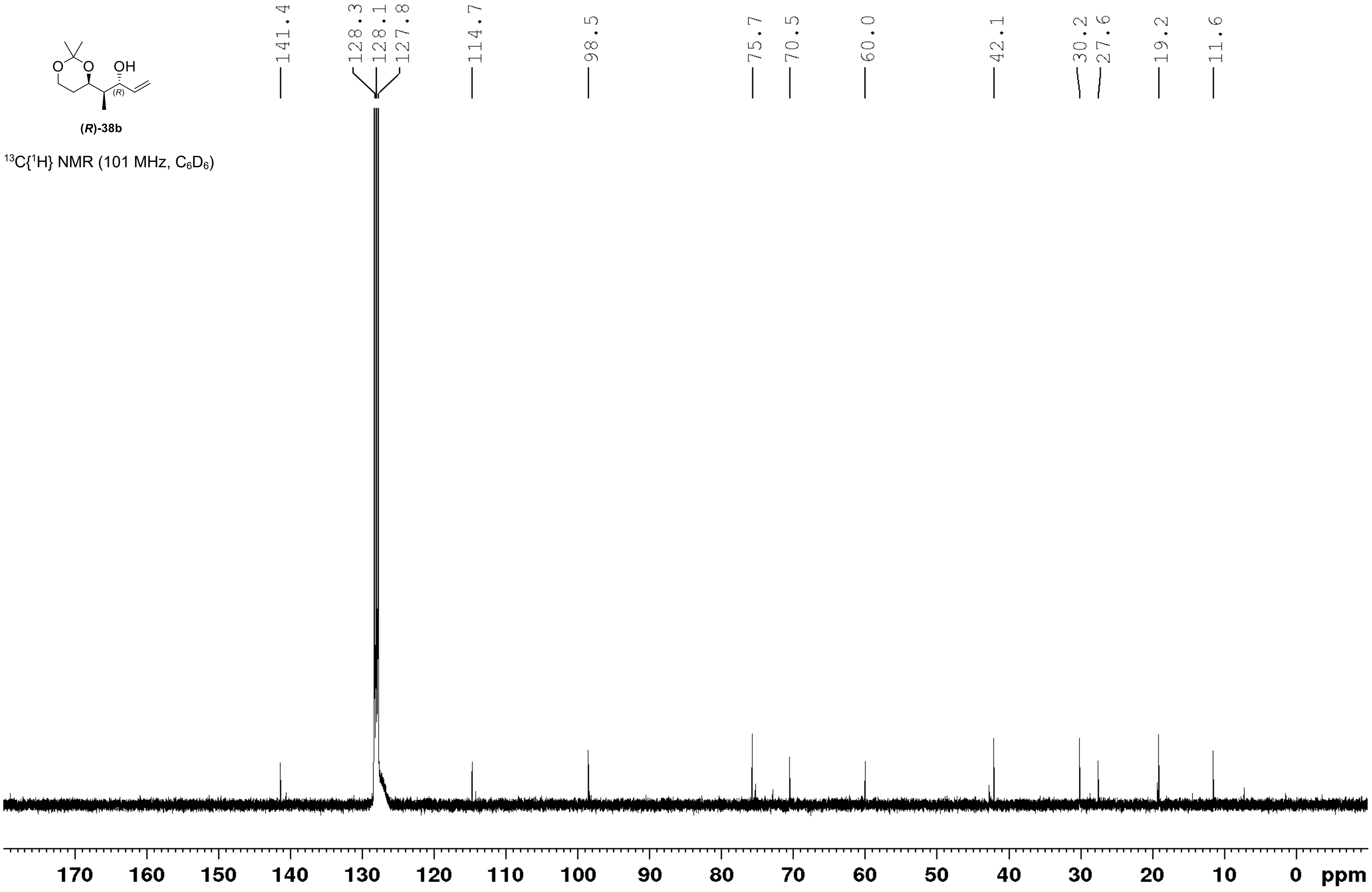
OH
(R)
(R)-38b
13C{1H} NMR (101 MHz, C6D6)
141.4
128.3
128.1
127.8
114.7
98.5
75.7
70.5
60.0
42.1
30.2
27.6
19.2
11.6
170
160
150
140
130
120
110
100
90
80
70
60
50
40
30
20
10
0
ppm

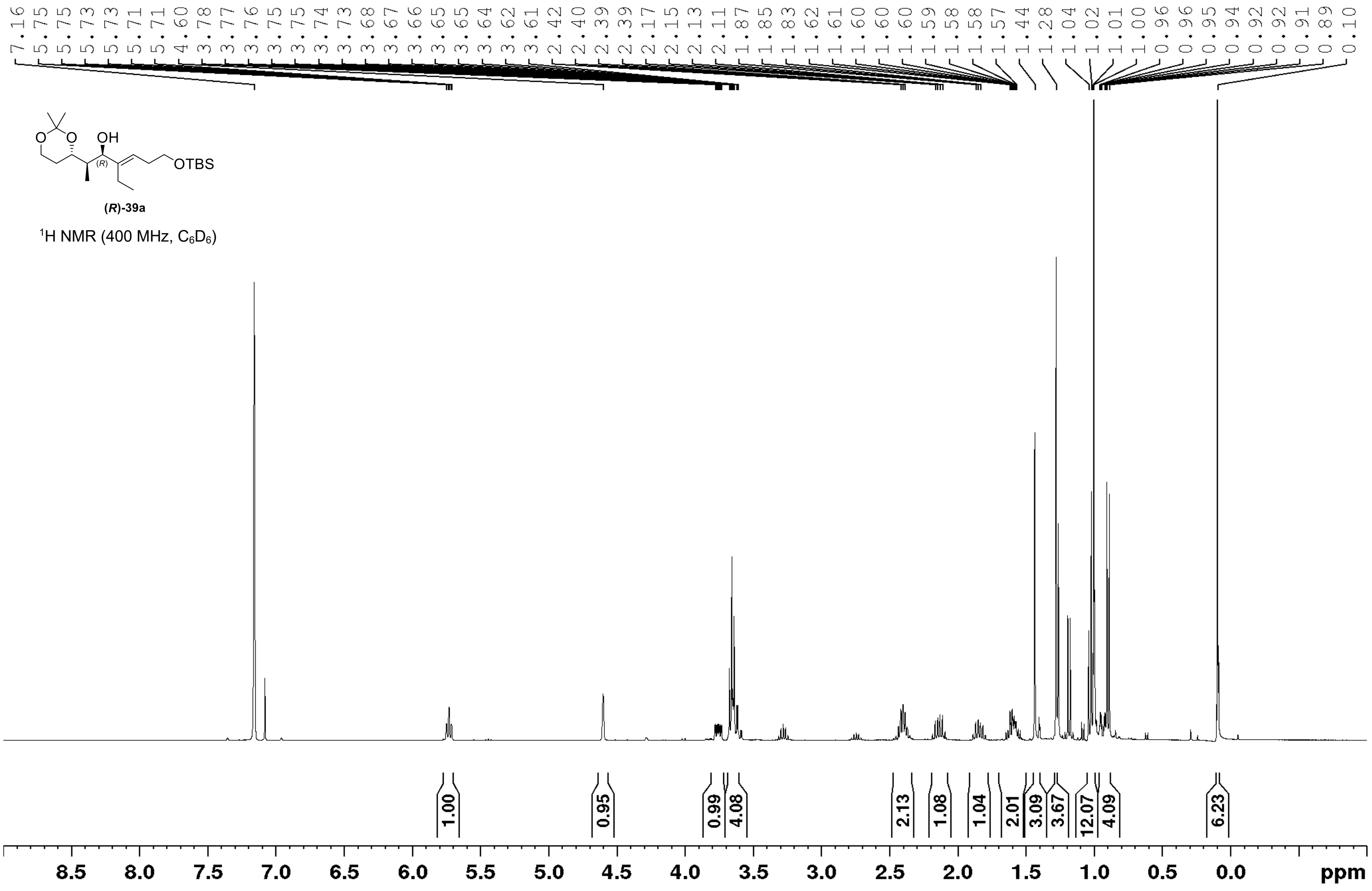

OH
OTBS
(R)
(R)-39a
^{1}H NMR (400 MHz, C_6D_6)
7.16 5.75 5.75 5.73 5.73 5.71 5.71 4.60 3.78 3.77 3.76 3.75 3.75 3.74 3.73 3.68 3.67 3.66 3.65 3.65 3.64 3.62 3.61 2.42 2.40 2.39 2.39 2.17 2.15 2.13 2.11 1.87 1.85 1.83 1.62 1.61 1.60 1.60 1.60 1.59 1.58 1.58 1.57 1.44 1.28 1.04 1.02 1.01 1.00 0.96 0.96 0.95 0.94 0.92 0.92 0.91 0.89 0.10
1.00 0.95 0.99 4.08 2.13 1.08 1.04 2.01 3.09 3.67 12.07 4.09 6.23
8.5 8.0 7.5 7.0 6.5 6.0 5.5 5.0 4.5 4.0 3.5 3.0 2.5 2.0 1.5 1.0 0.5 0.0 ppm

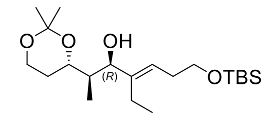

(*R*)-39a

$^{13}C\{^{1}H\}$ NMR (101 MHz, C_6D_6)

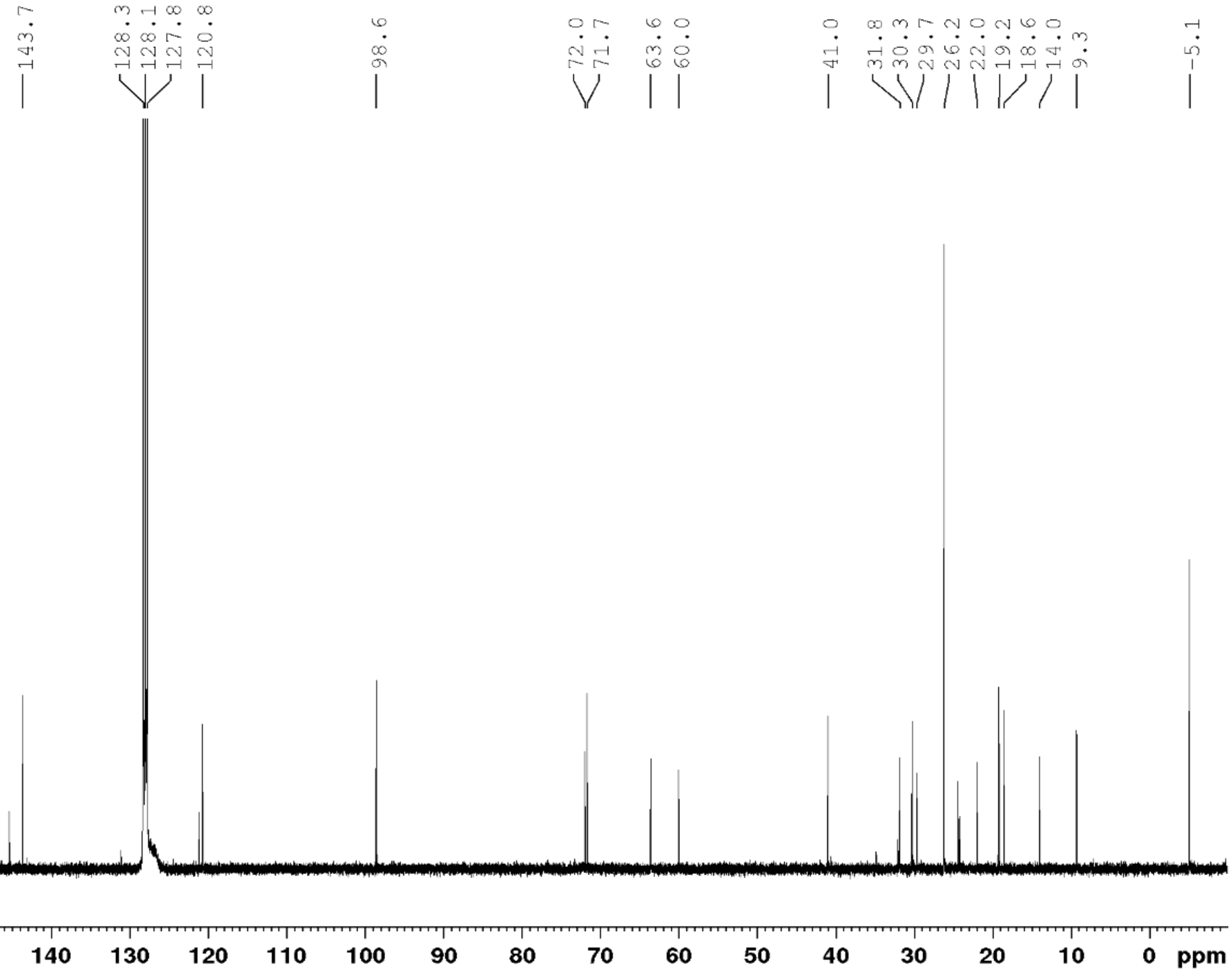

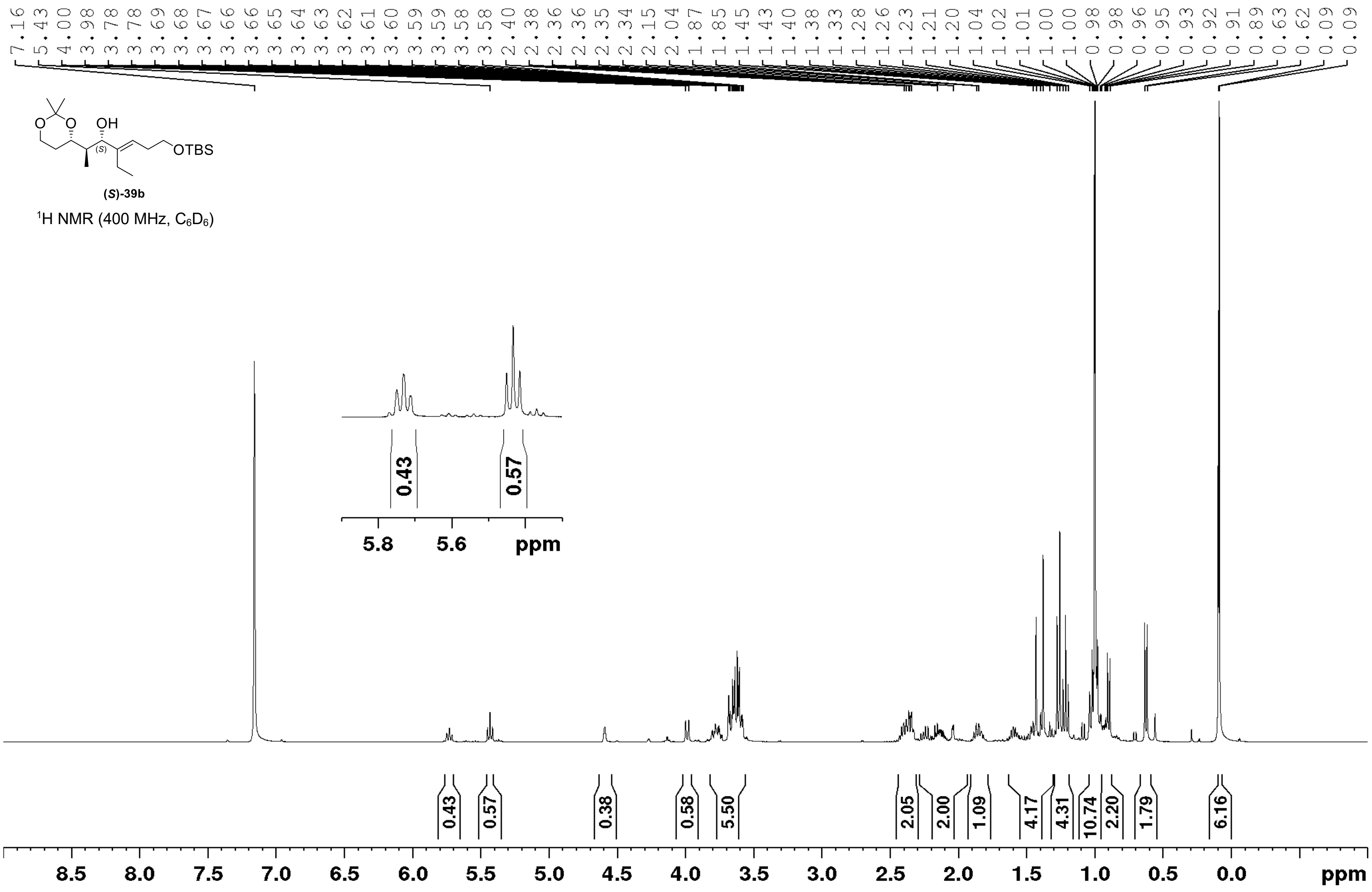
OH
OTBS
(S)
(S)-39b
¹H NMR (400 MHz, C₆D₆)
0.43
0.57
5.8
5.6
ppm
0.43
0.57
0.38
0.58
5.50
2.05
2.00
1.09
4.17
4.31
10.74
2.20
1.79
6.16
8.5
8.0
7.5
7.0
6.5
6.0
5.5
5.0
4.5
4.0
3.5
3.0
2.5
2.0
1.5
1.0
0.5
0.0
ppm

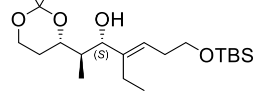

(*S*)-39b

$^{13}C\{^1H\}$ NMR (101 MHz, C_6D_6)

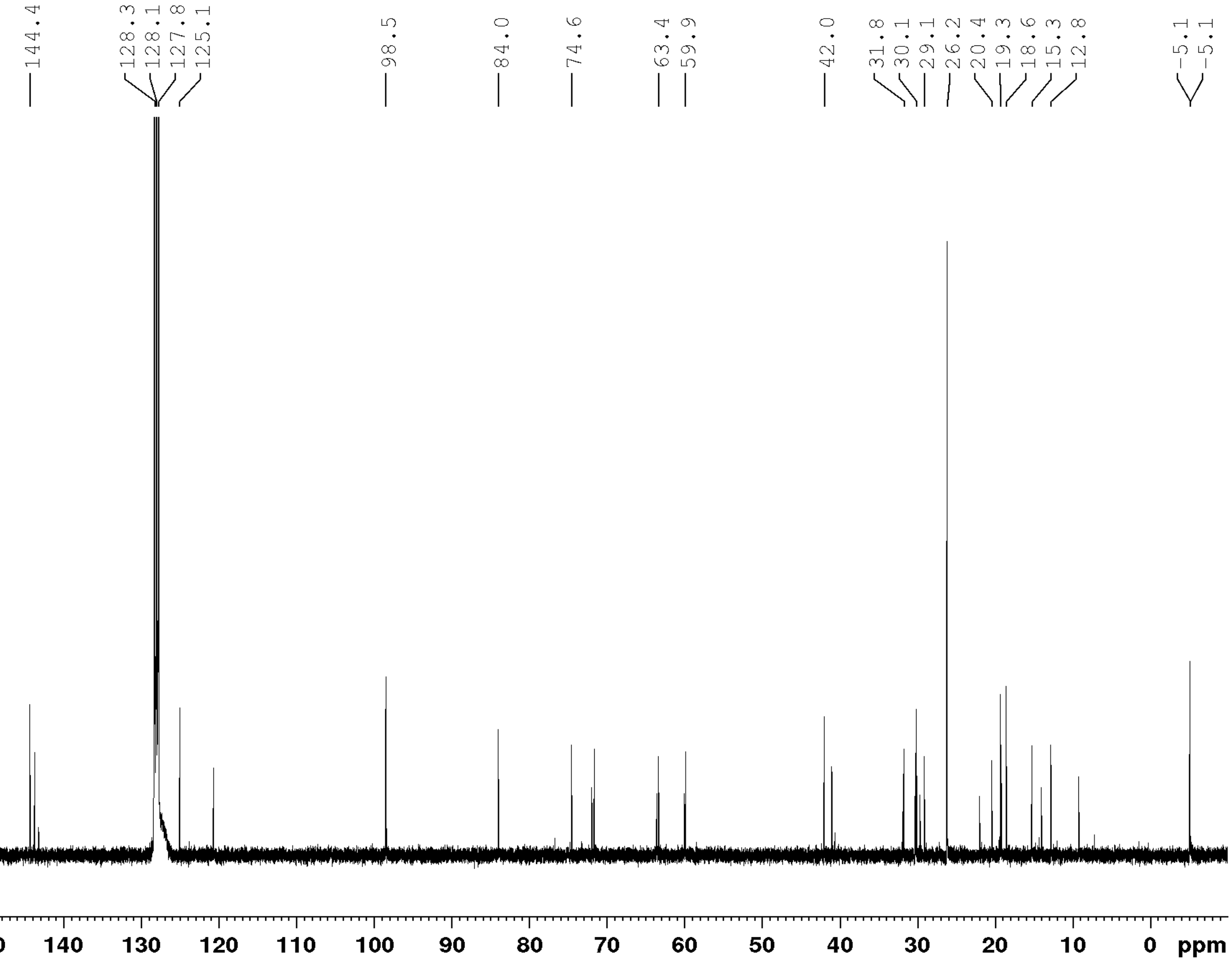

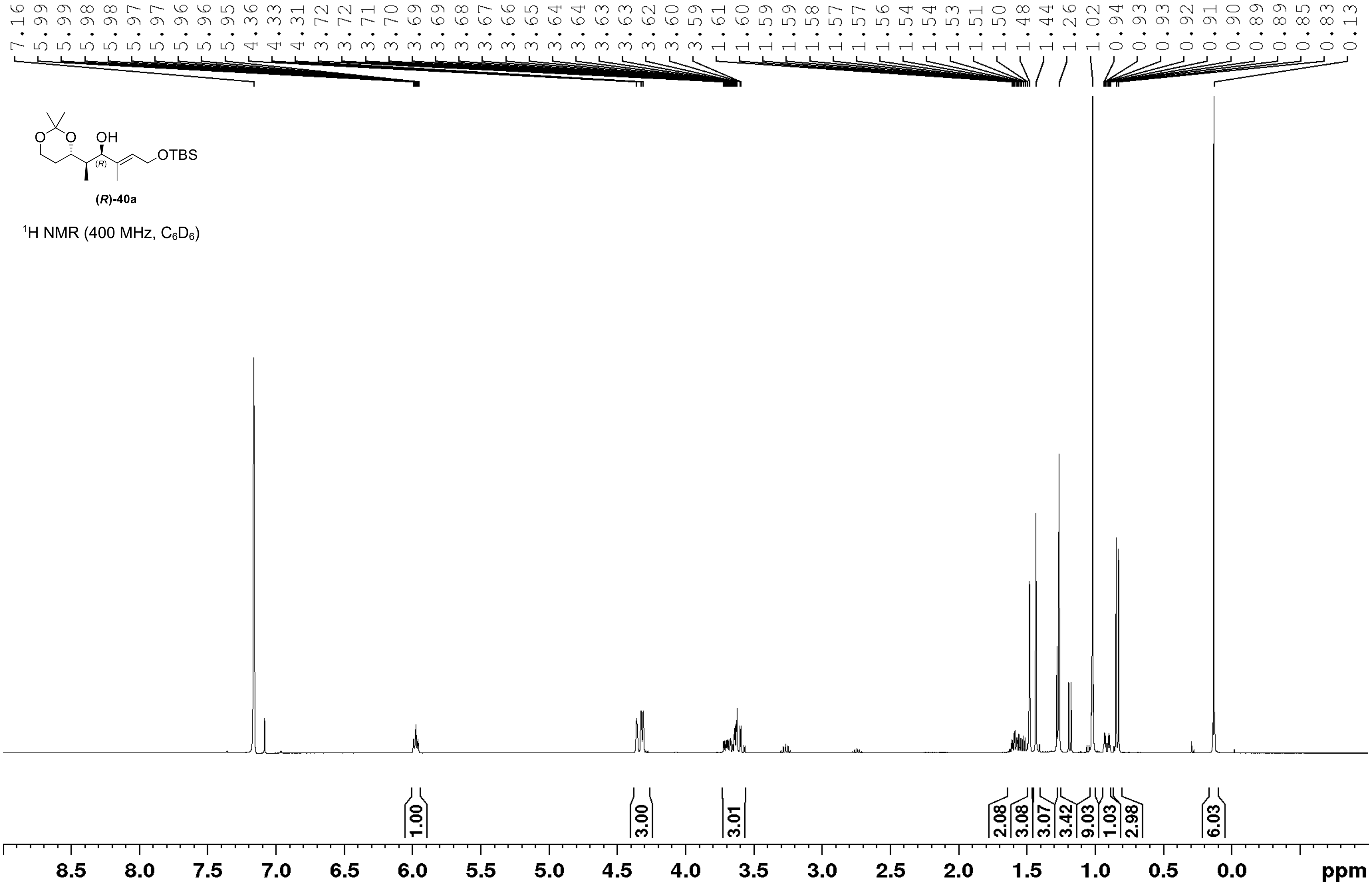
7.16 5.99 5.99 5.98 5.98 5.97 5.97 5.96 5.96 5.95 4.36 4.33 4.31 3.72 3.72 3.71 3.70 3.69 3.69 3.68 3.67 3.66 3.65 3.64 3.64 3.63 3.63 3.62 3.60 3.59 1.61 1.60 1.59 1.59 1.58 1.57 1.57 1.56 1.54 1.54 1.53 1.51 1.50 1.48 1.44 1.26 1.02 0.94 0.93 0.93 0.92 0.91 0.90 0.89 0.89 0.85 0.83 0.13
O
O
OH
(R)
OTBS
(R)-40a
1H NMR (400 MHz, C6D6)
1.00
3.00
3.01
2.08
3.08
3.07
3.42
9.03
1.03
2.98
6.03
8.5 8.0 7.5 7.0 6.5 6.0 5.5 5.0 4.5 4.0 3.5 3.0 2.5 2.0 1.5 1.0 0.5 0.0
ppm

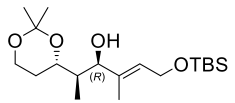

(*R*)-40a

$^{13}C\{^1H\}$ NMR (101 MHz, C_6D_6)

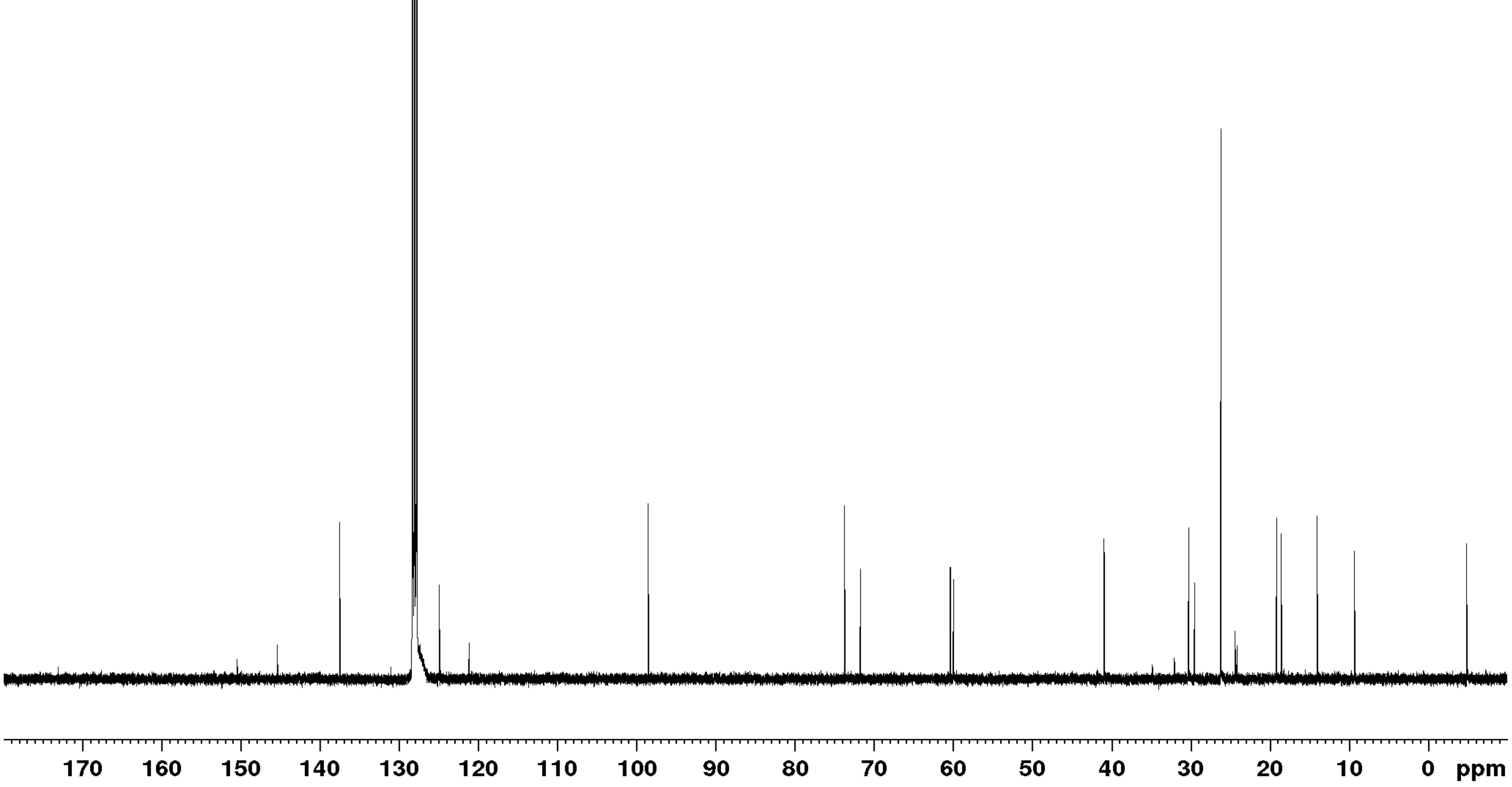

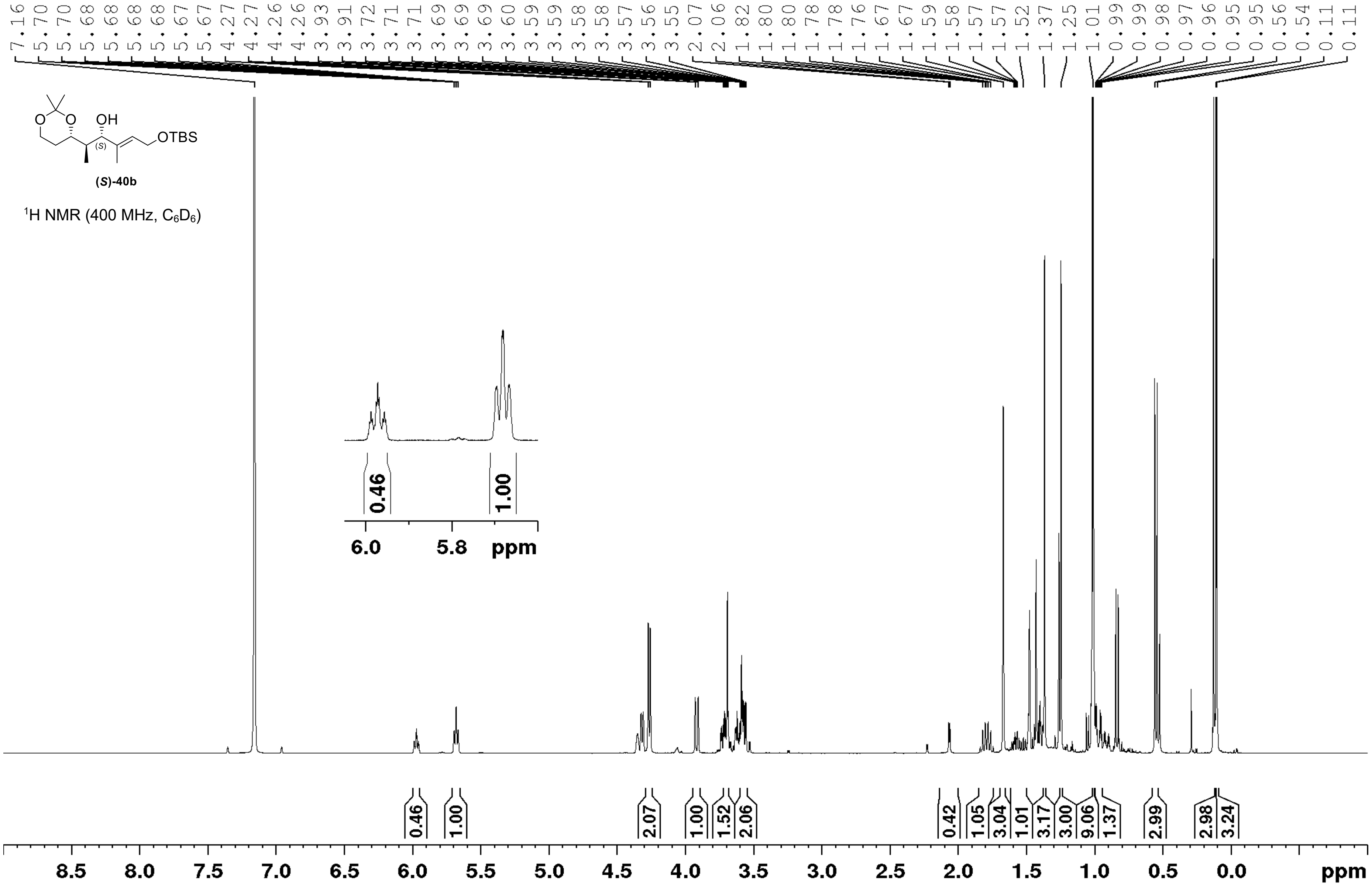

OH
OTBS
(S)-40b
^{1}H NMR (400 MHz, C_6D_6)
ppm

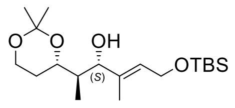

(S)-40b

$^{13}C\{^{1}H\}$ NMR (101 MHz, C_6D_6)

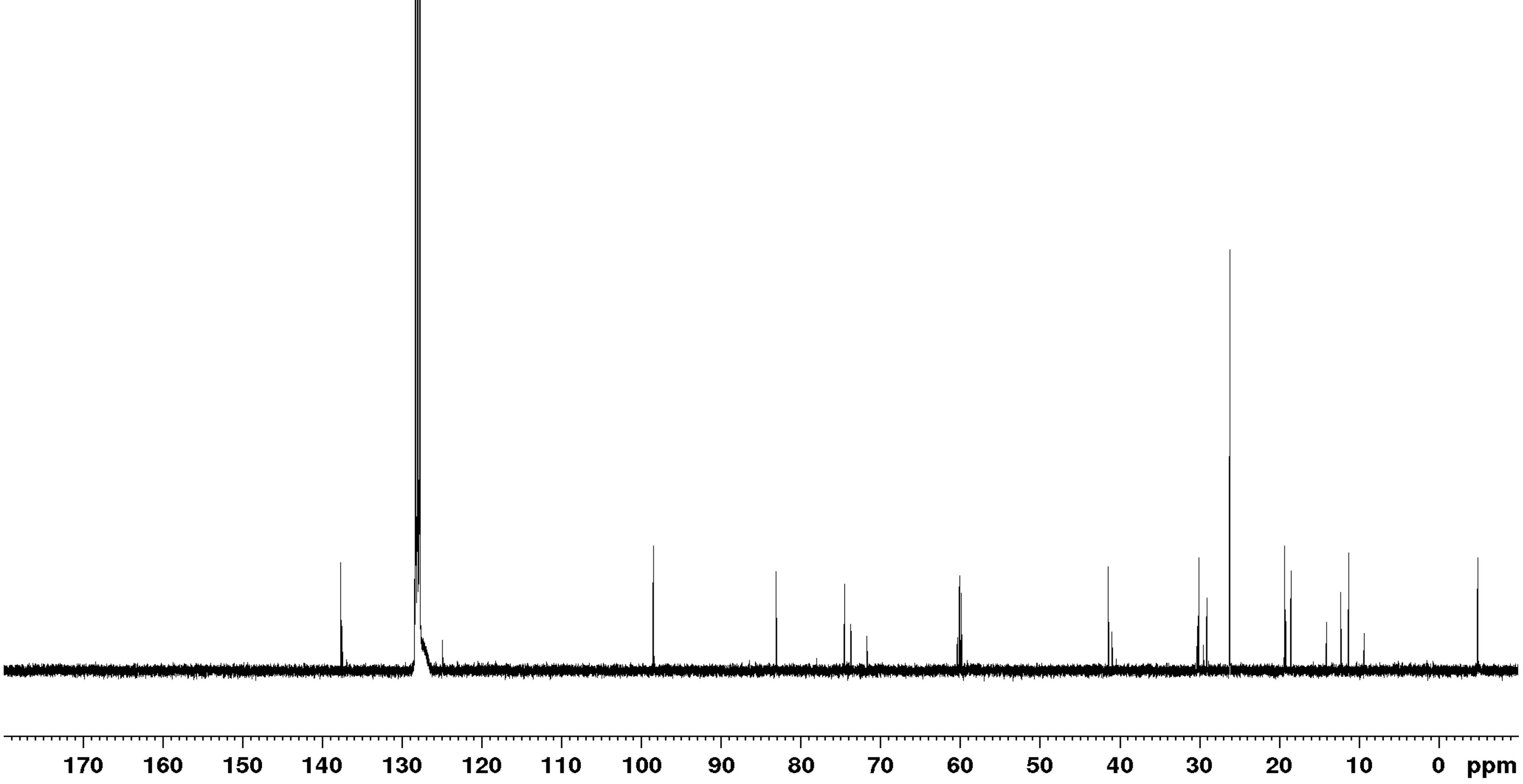

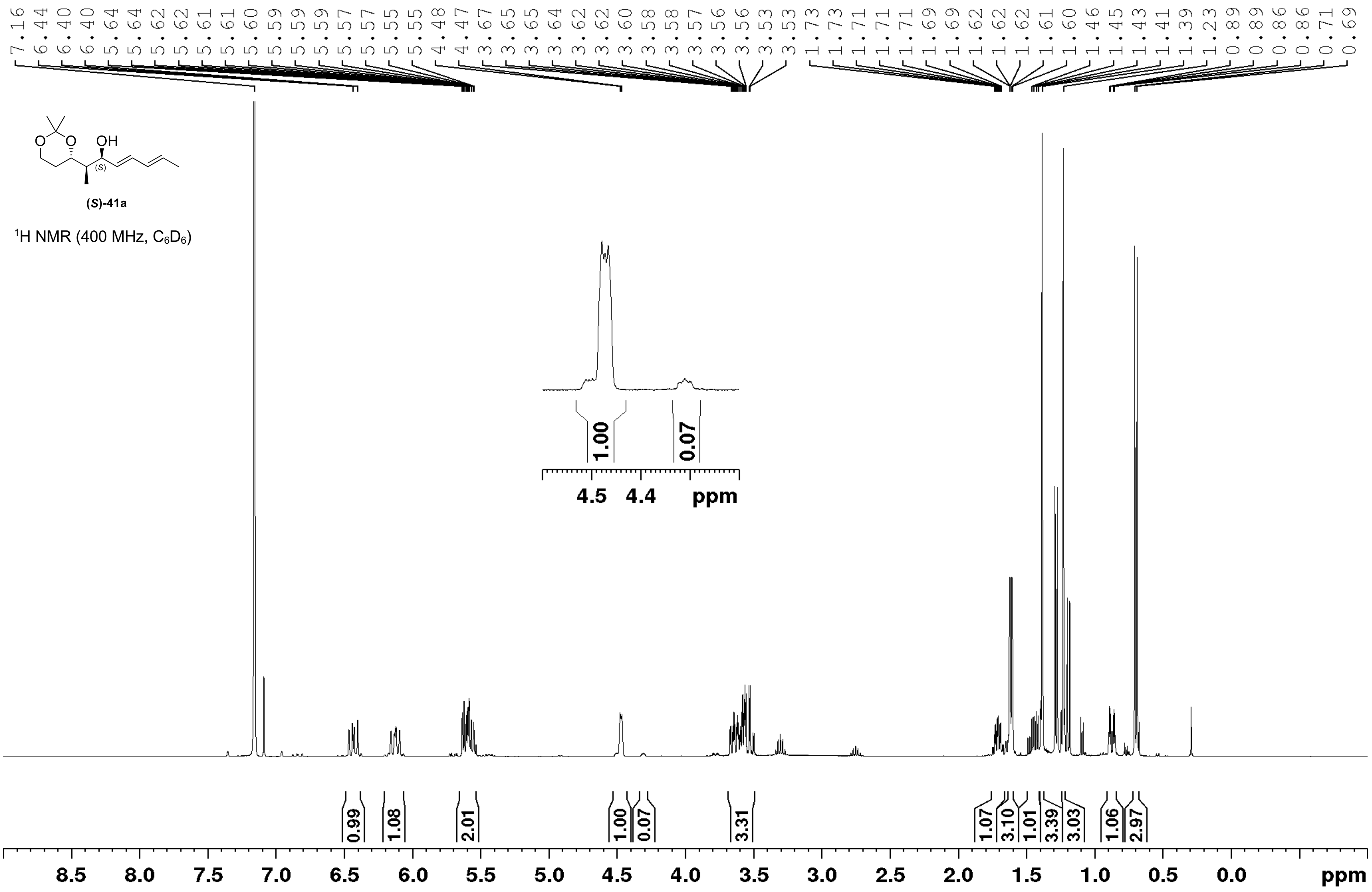
(S)-41a
¹H NMR (400 MHz, C6D6)
7.16 6.44 6.40 6.40 5.64 5.64 5.62 5.62 5.61 5.61 5.60 5.59 5.59 5.59 5.57 5.57 5.55 5.55 4.48 4.47 3.67 3.65 3.65 3.64 3.62 3.62 3.60 3.58 3.58 3.57 3.56 3.56 3.53 3.53 1.73 1.73 1.71 1.71 1.71 1.69 1.69 1.62 1.62 1.62 1.61 1.60 1.46 1.45 1.43 1.41 1.39 1.23 0.89 0.89 0.86 0.86 0.71 0.69
1.00 0.07
4.5 4.4 ppm
0.99 1.08 2.01 1.00 0.07 3.31 1.07 3.10 1.01 3.39 3.03 1.06 2.97
8.5 8.0 7.5 7.0 6.5 6.0 5.5 5.0 4.5 4.0 3.5 3.0 2.5 2.0 1.5 1.0 0.5 0.0 ppm

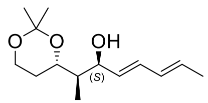

(*S*)-41a

$^{13}C\{^{1}H\}$ NMR (101 MHz, C_6D_6)

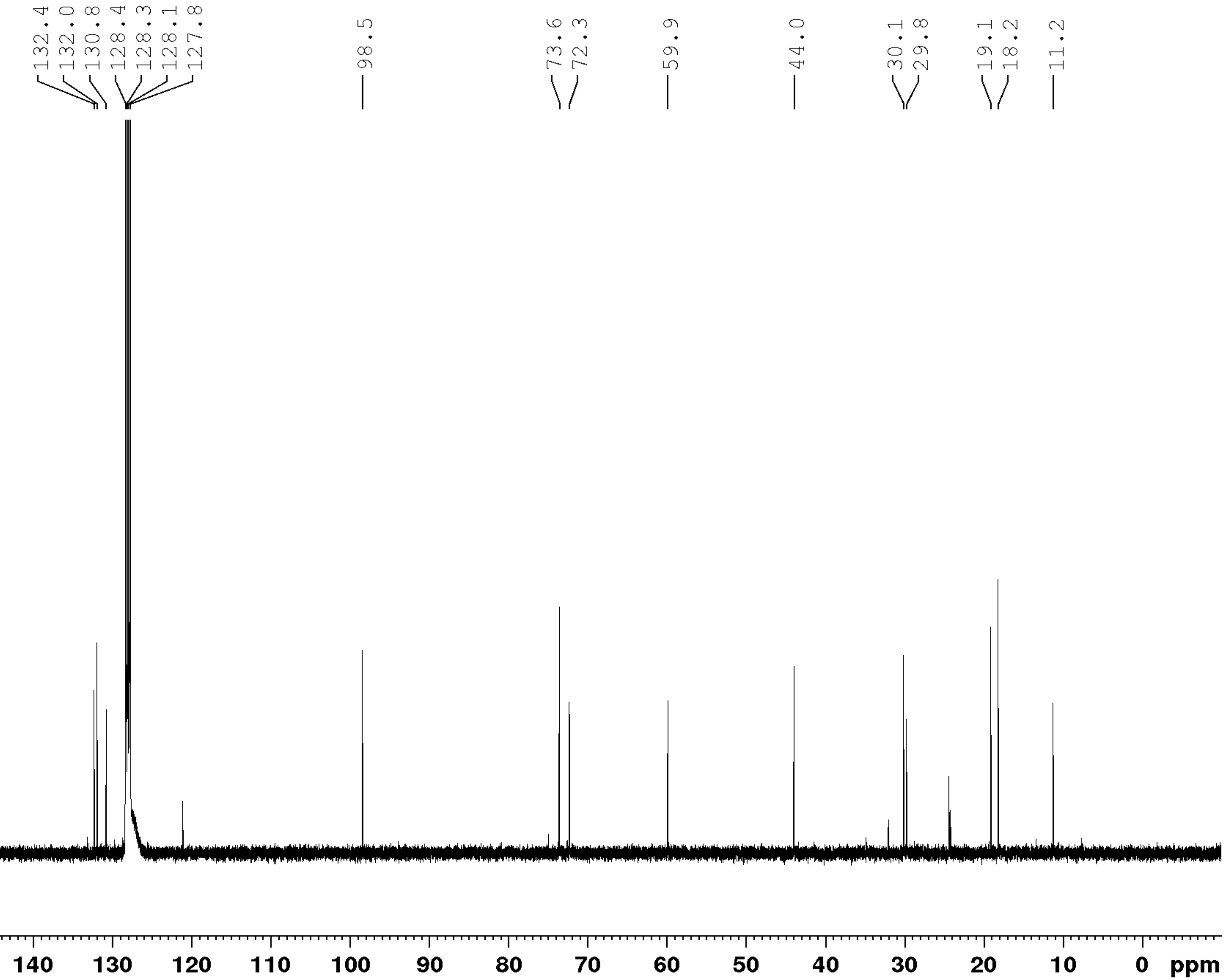

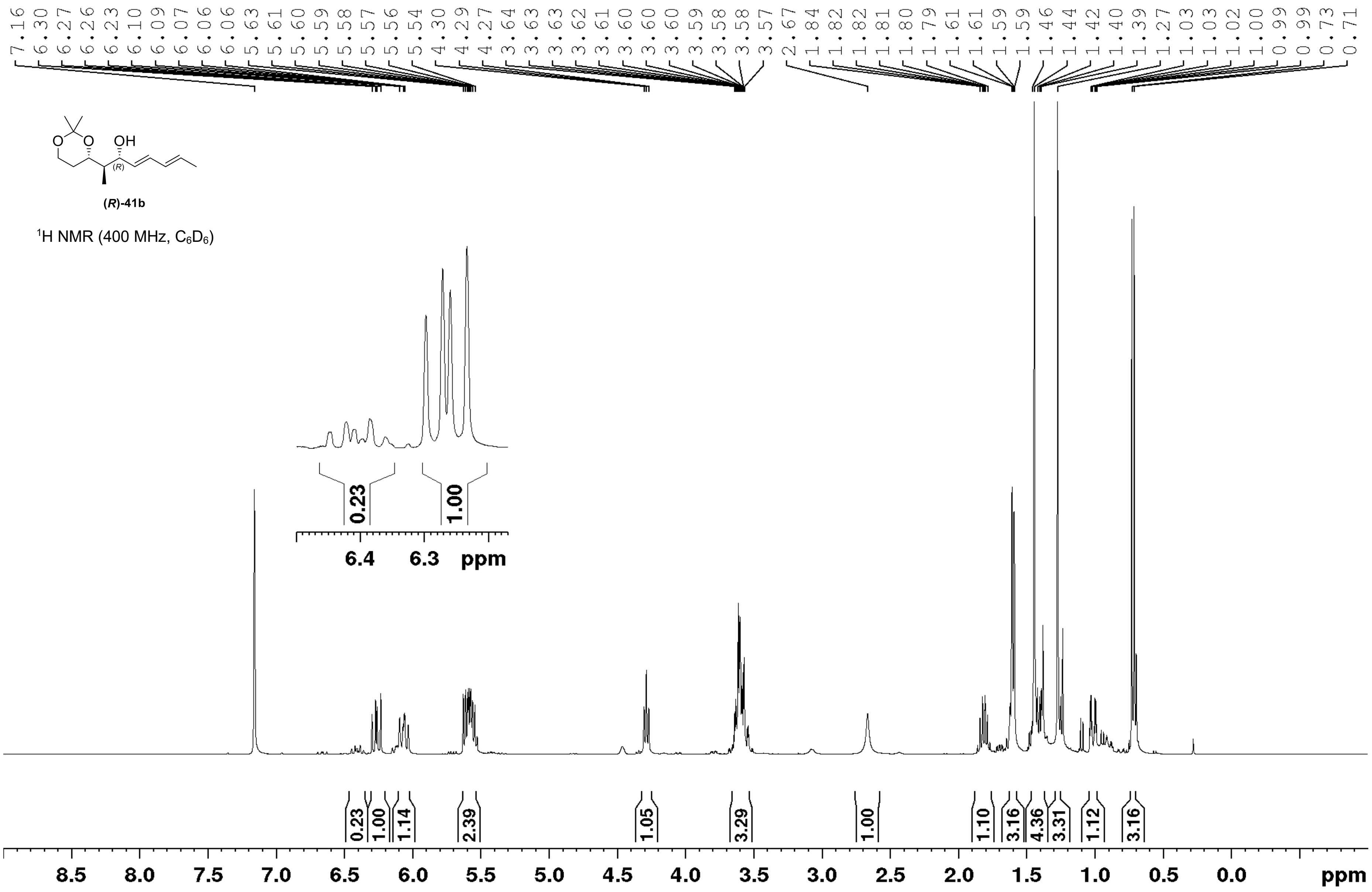

OH
(R)
(R)-41b
^{1}H NMR (400 MHz, C_6D_6)
ppm

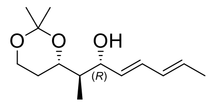

(*R*)-41b

$^{13}C\{^{1}H\}$ NMR (101 MHz, C_6D_6)

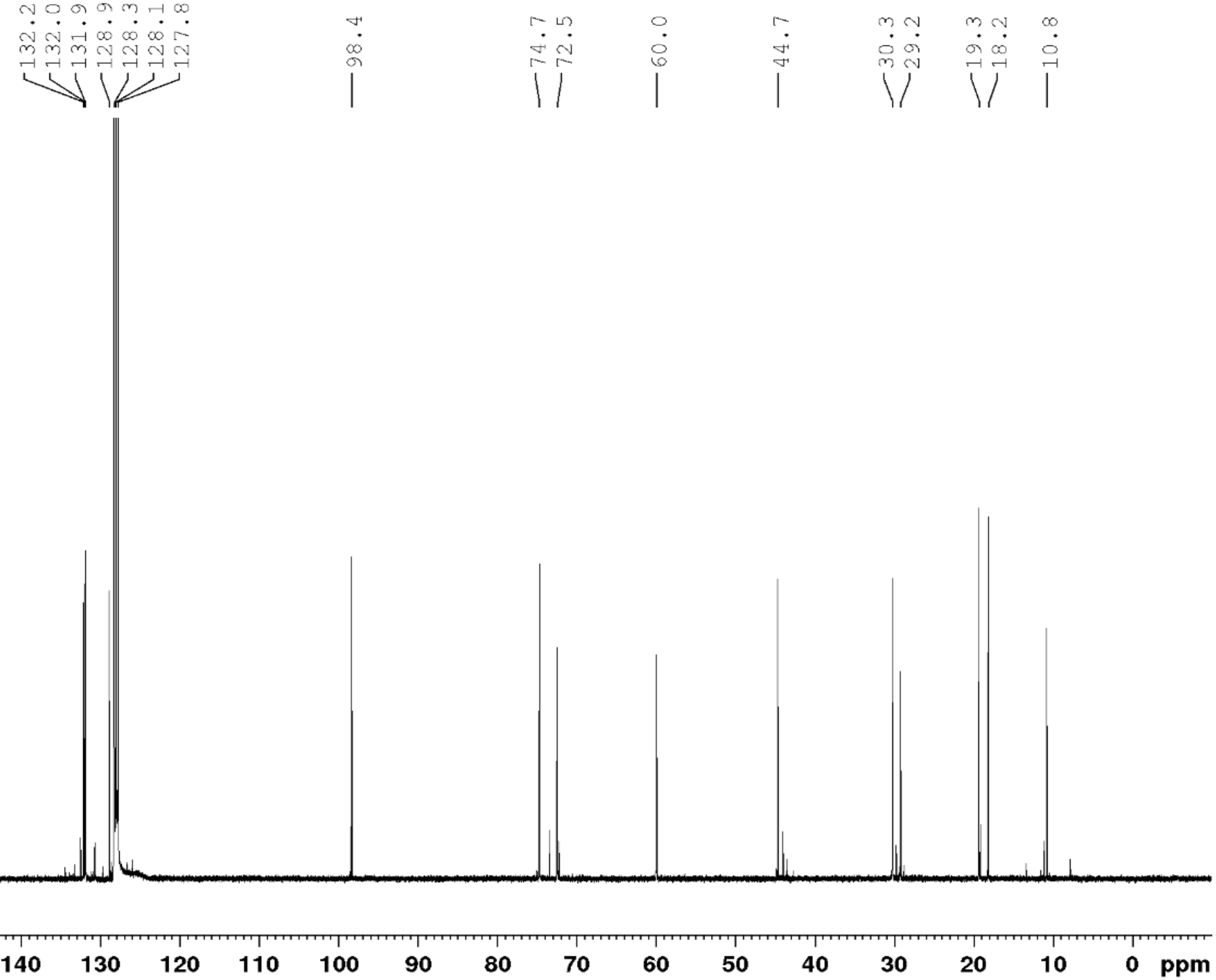

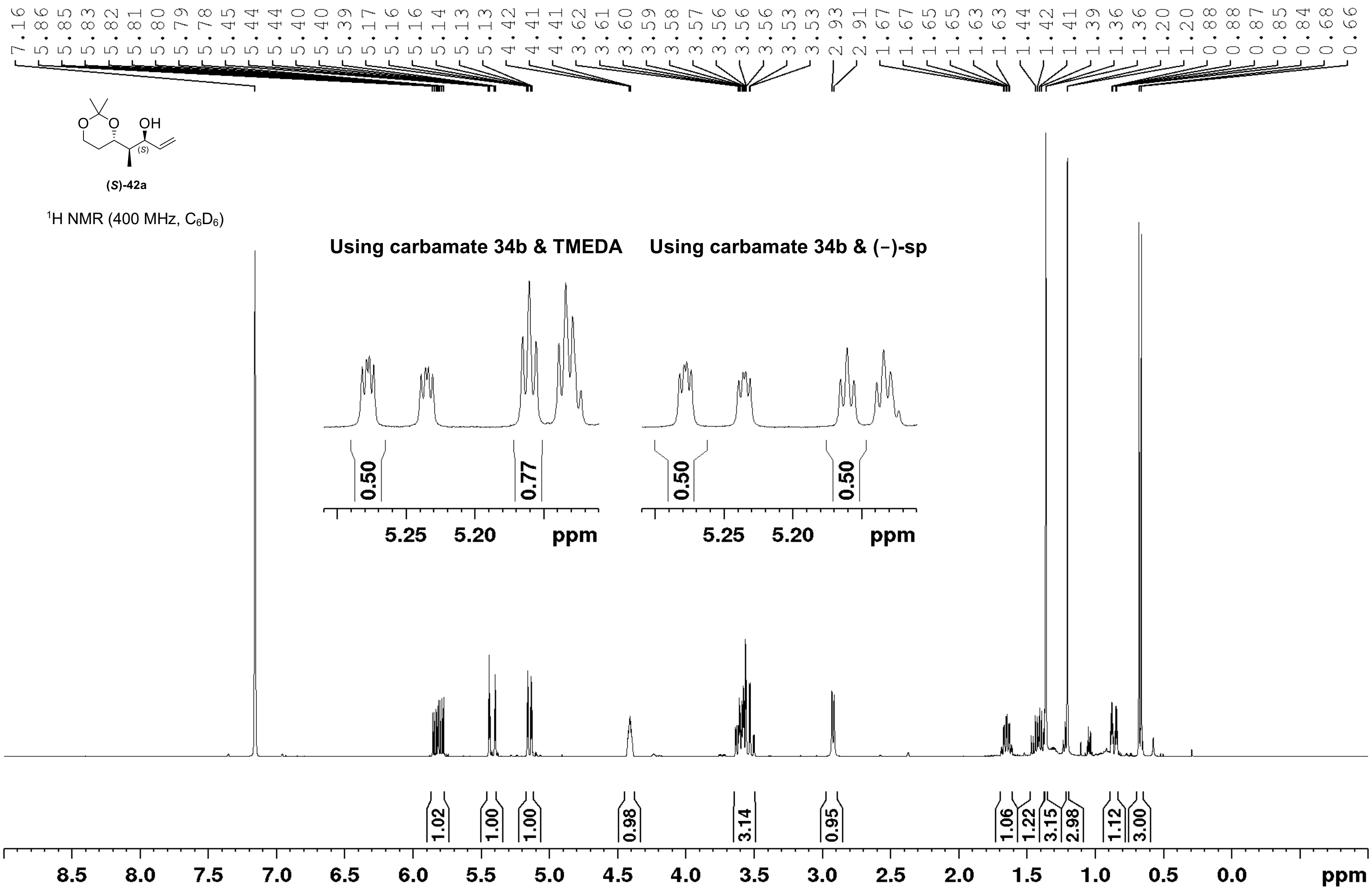

(S)-42a
^{1}H NMR (400 MHz, C_6D_6)
Using carbamate 34b & TMEDA
Using carbamate 34b & (−)-sp
ppm

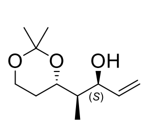

(*S*)-42a

$^{13}C\{^1H\}$ NMR (101 MHz, C_6D_6)

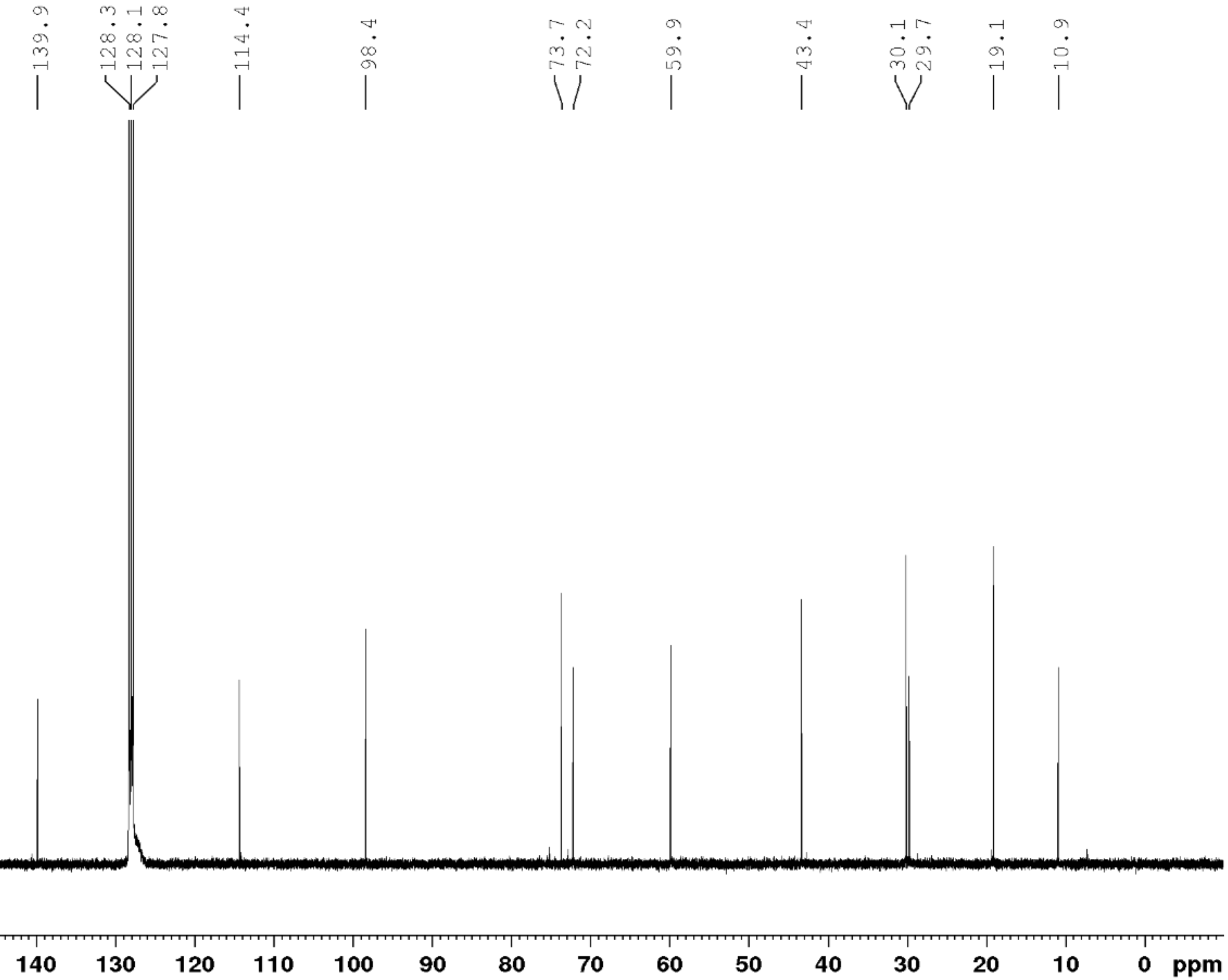

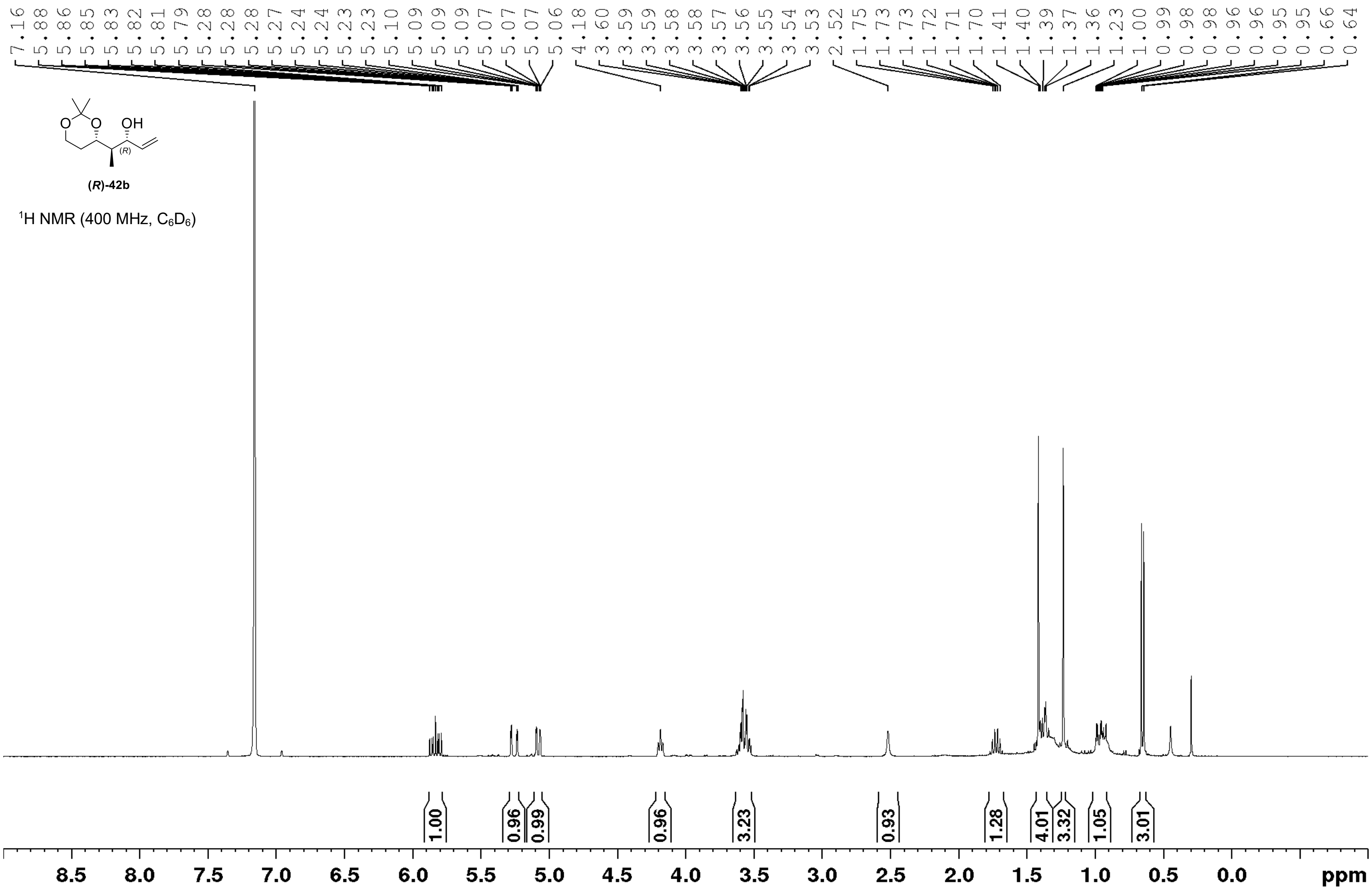

(*R*)-42b

^{1}H NMR (400 MHz, C_6D_6)

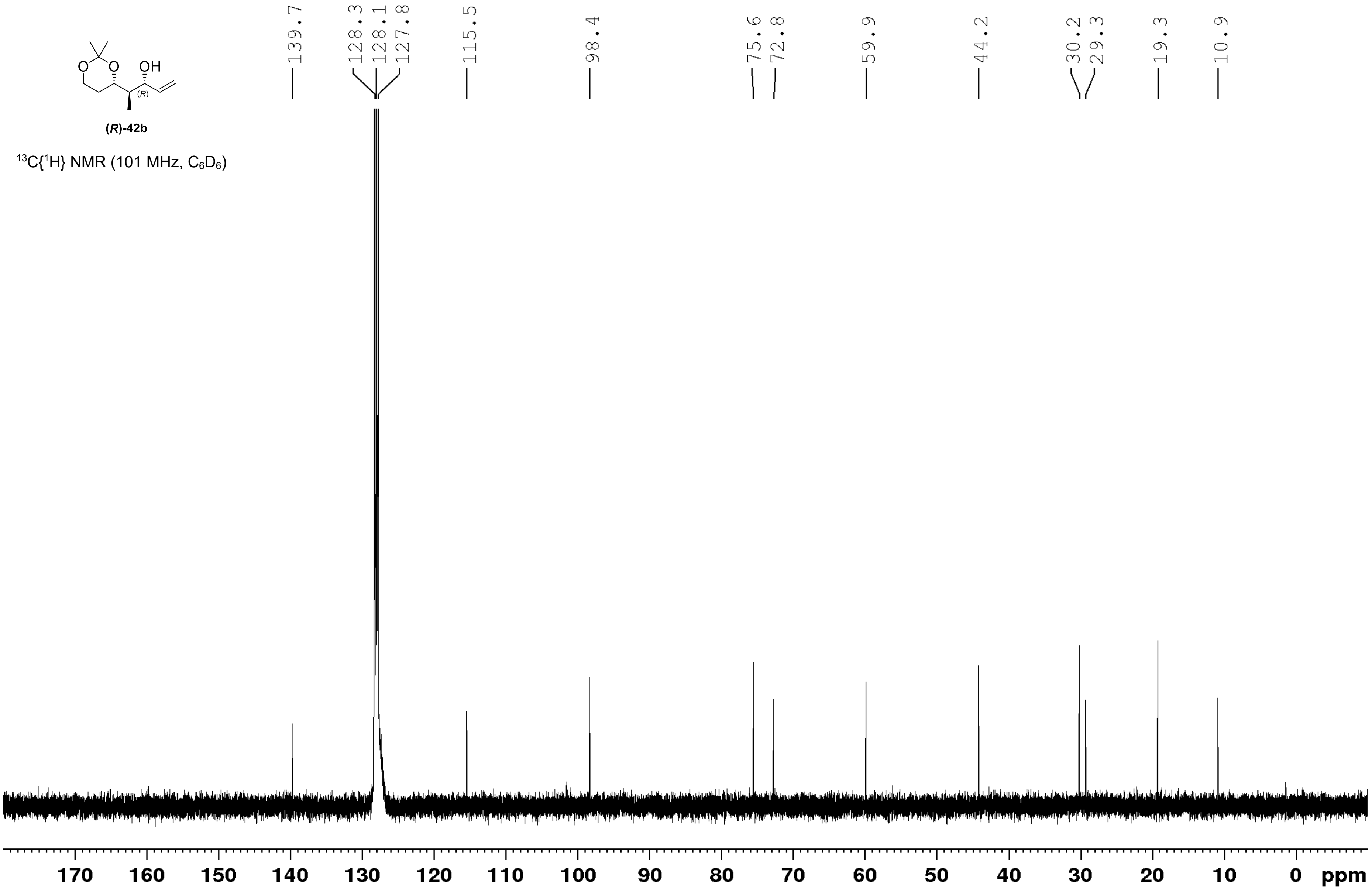

(R)-42b
13C{1H} NMR (101 MHz, C6D6)
139.7
128.3
128.1
127.8
115.5
98.4
75.6
72.8
59.9
44.2
30.2
29.3
19.3
10.9
170 160 150 140 130 120 110 100 90 80 70 60 50 40 30 20 10 0 ppm

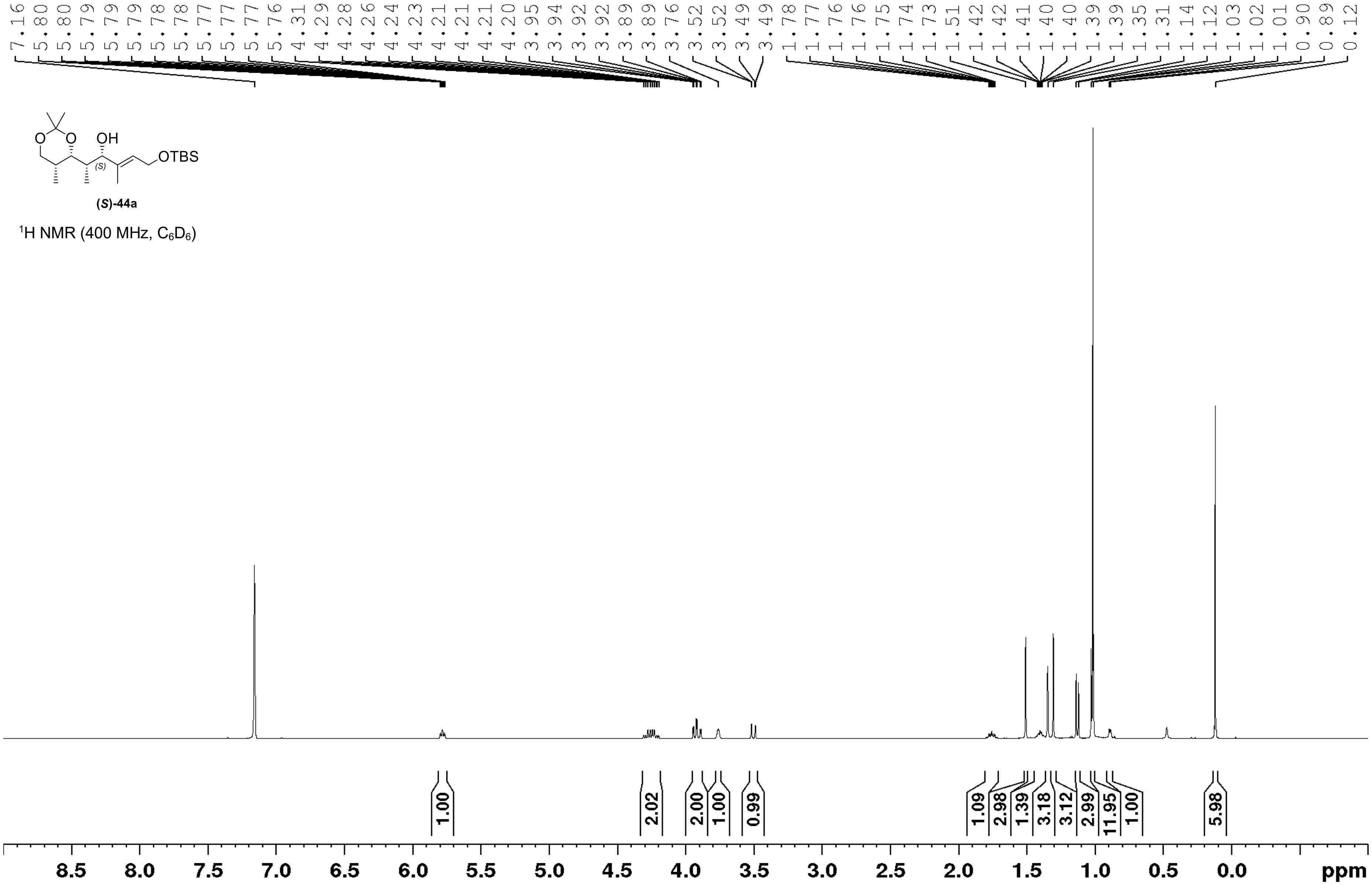
7.16 5.80 5.80 5.79 5.79 5.79 5.78 5.78 5.77 5.77 5.77 5.76 4.31 4.29 4.28 4.26 4.24 4.23 4.21 4.21 4.21 4.20 3.95 3.94 3.92 3.92 3.89 3.89 3.76 3.52 3.52 3.49 3.49 1.78 1.77 1.76 1.76 1.75 1.74 1.73 1.51 1.42 1.42 1.41 1.40 1.40 1.39 1.39 1.35 1.31 1.14 1.12 1.03 1.02 1.01 0.90 0.89 0.12
OH
OTBS
(S)
(S)-44a
1H NMR (400 MHz, C6D6)
1.00 2.02 2.00 1.00 0.99 1.09 2.98 1.39 3.18 3.12 2.99 11.95 1.00 5.98
8.5 8.0 7.5 7.0 6.5 6.0 5.5 5.0 4.5 4.0 3.5 3.0 2.5 2.0 1.5 1.0 0.5 0.0 ppm

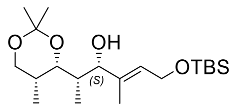

(*S*)-44a

$^{13}C\{^1H\}$ NMR (101 MHz, C_6D_6)

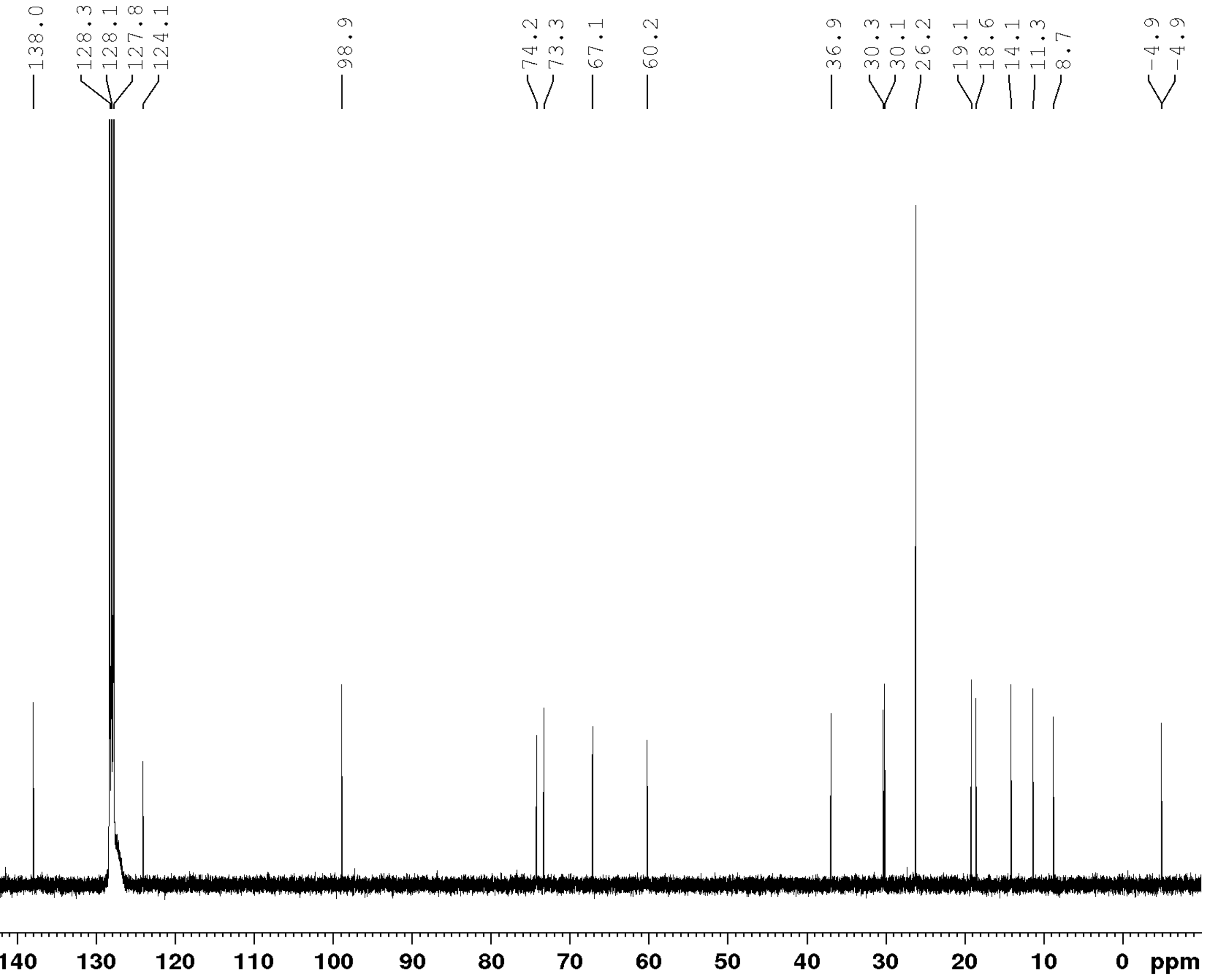

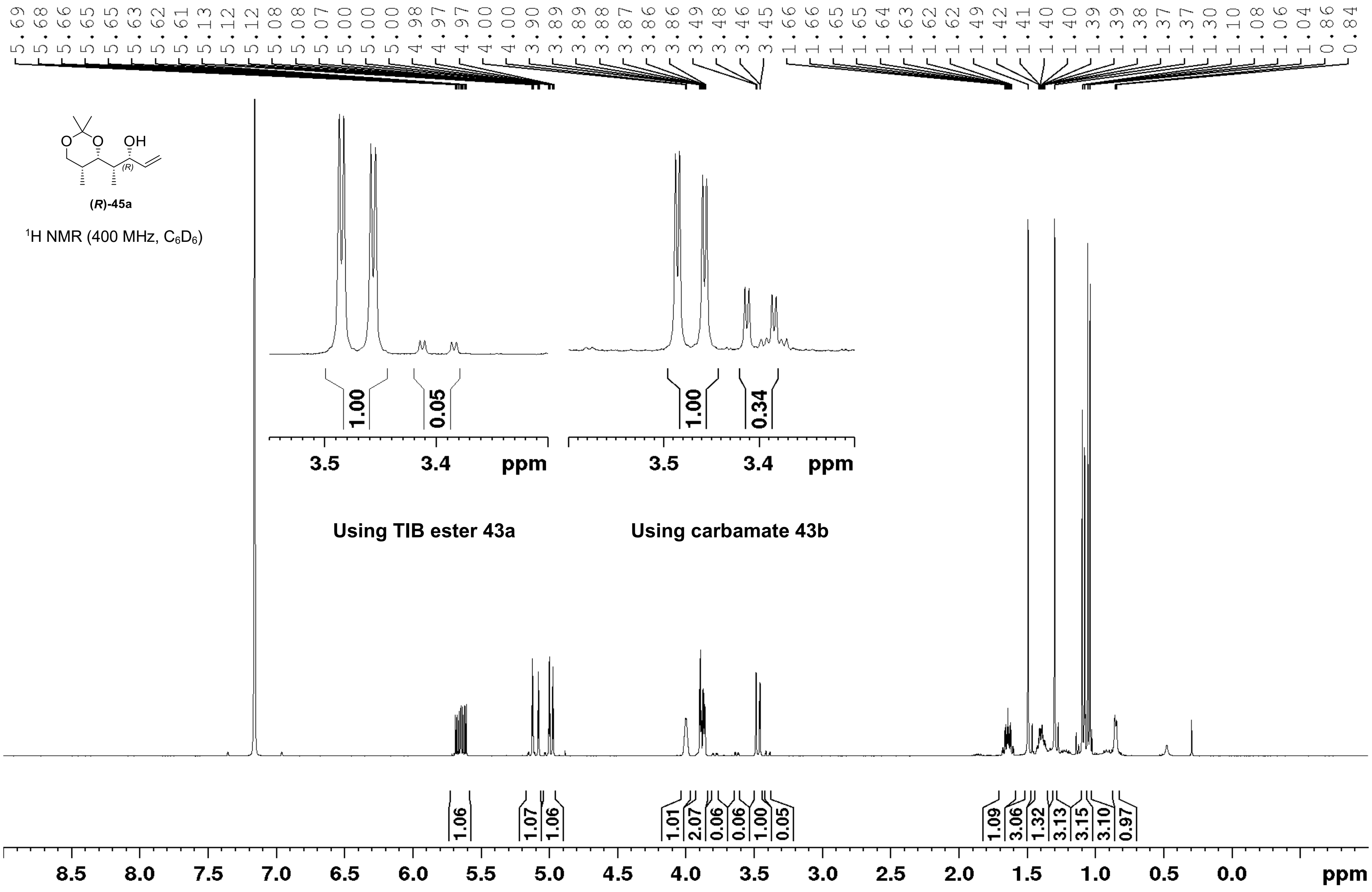
OH
(R)
(R)-45a
1H NMR (400 MHz, C6D6)
Using TIB ester 43a
Using carbamate 43b
ppm

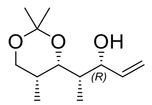

(R)-45a

$^{13}C\{^1H\}$ NMR (101 MHz, C_6D_6)

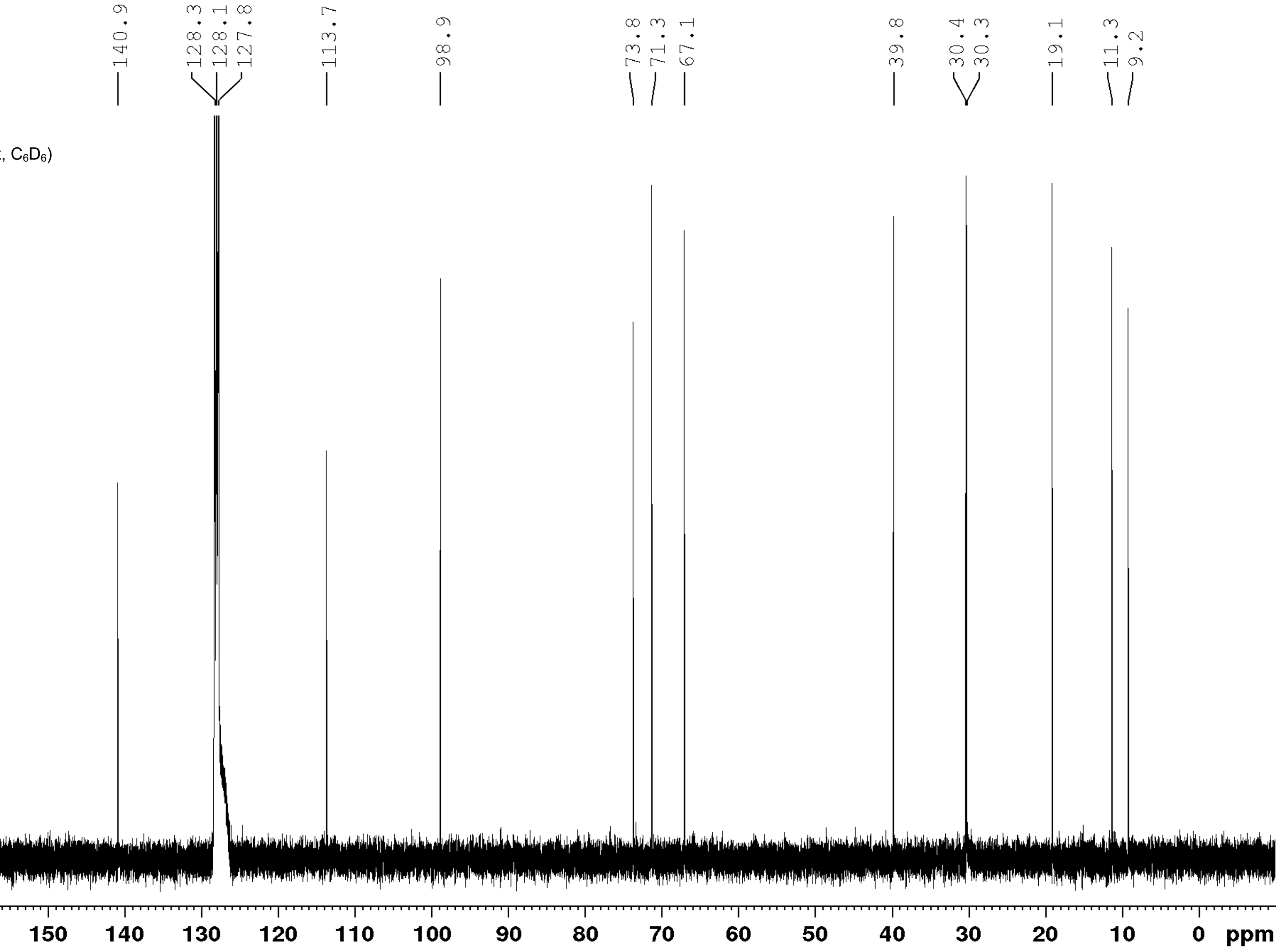

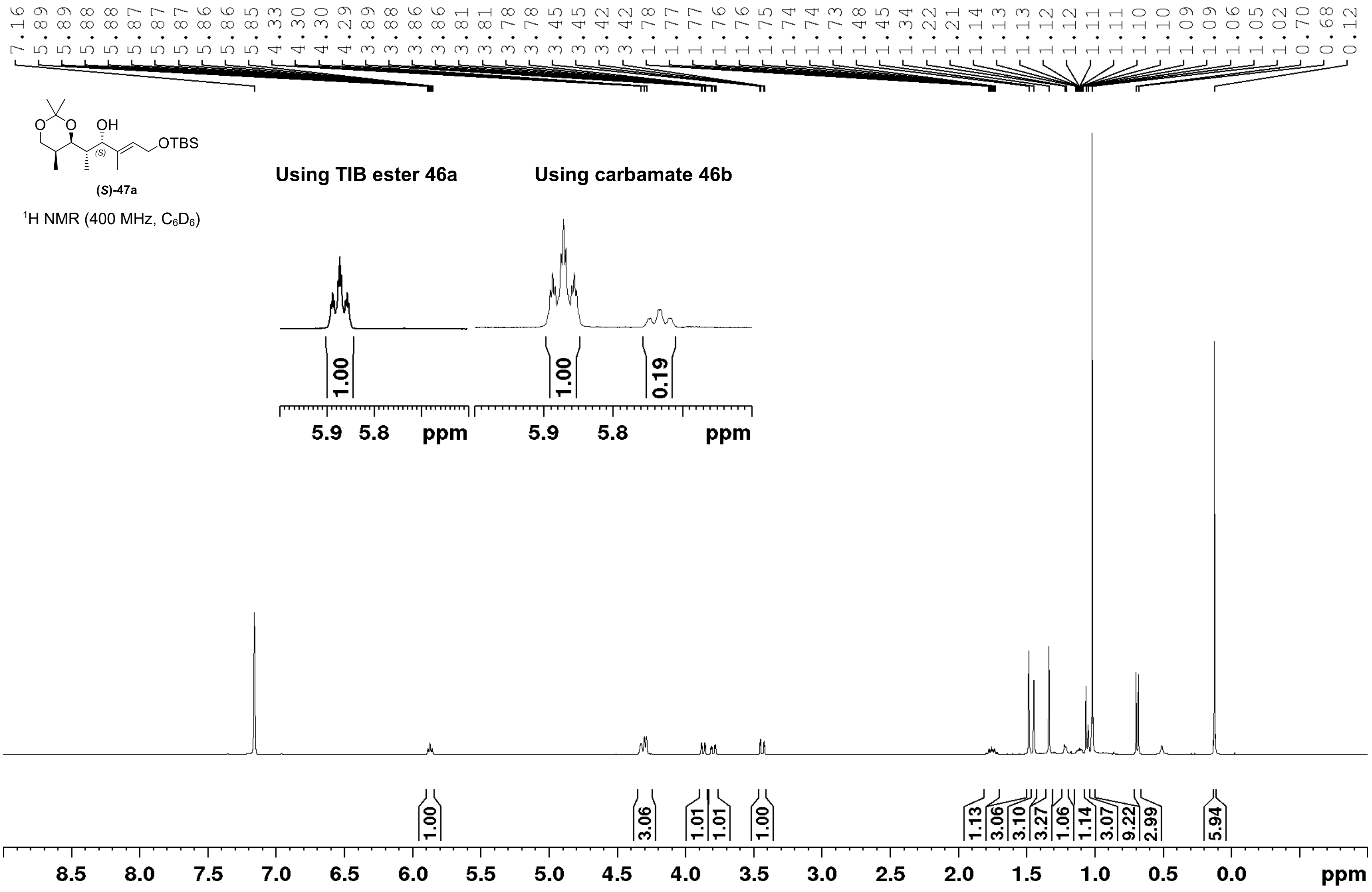

^{1}H NMR (400 MHz, C_6D_6)

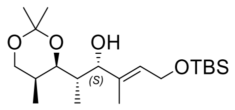

(*S*)-47a

$^{13}C\{^{1}H\}$ NMR (101 MHz, C_6D_6)

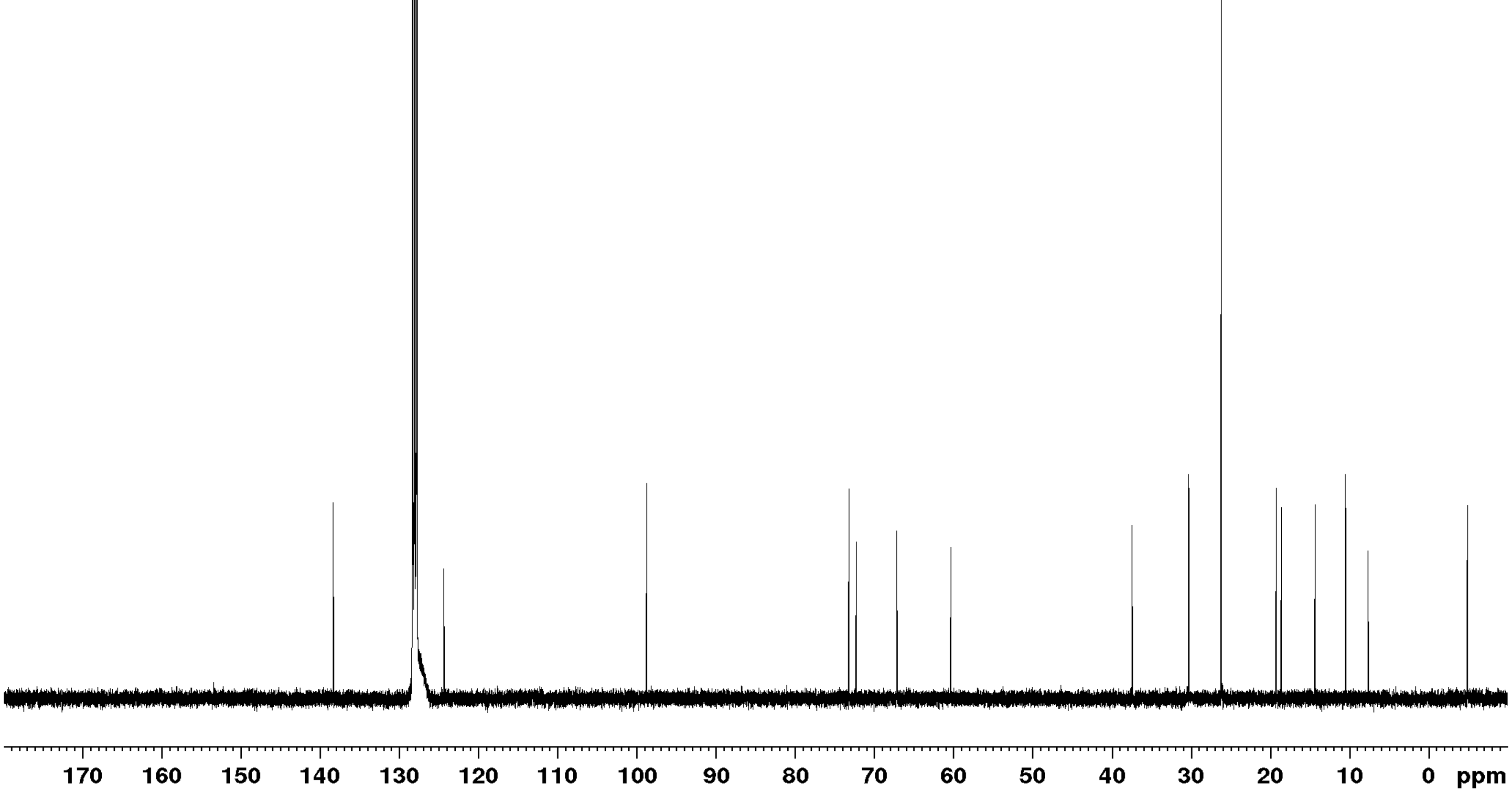

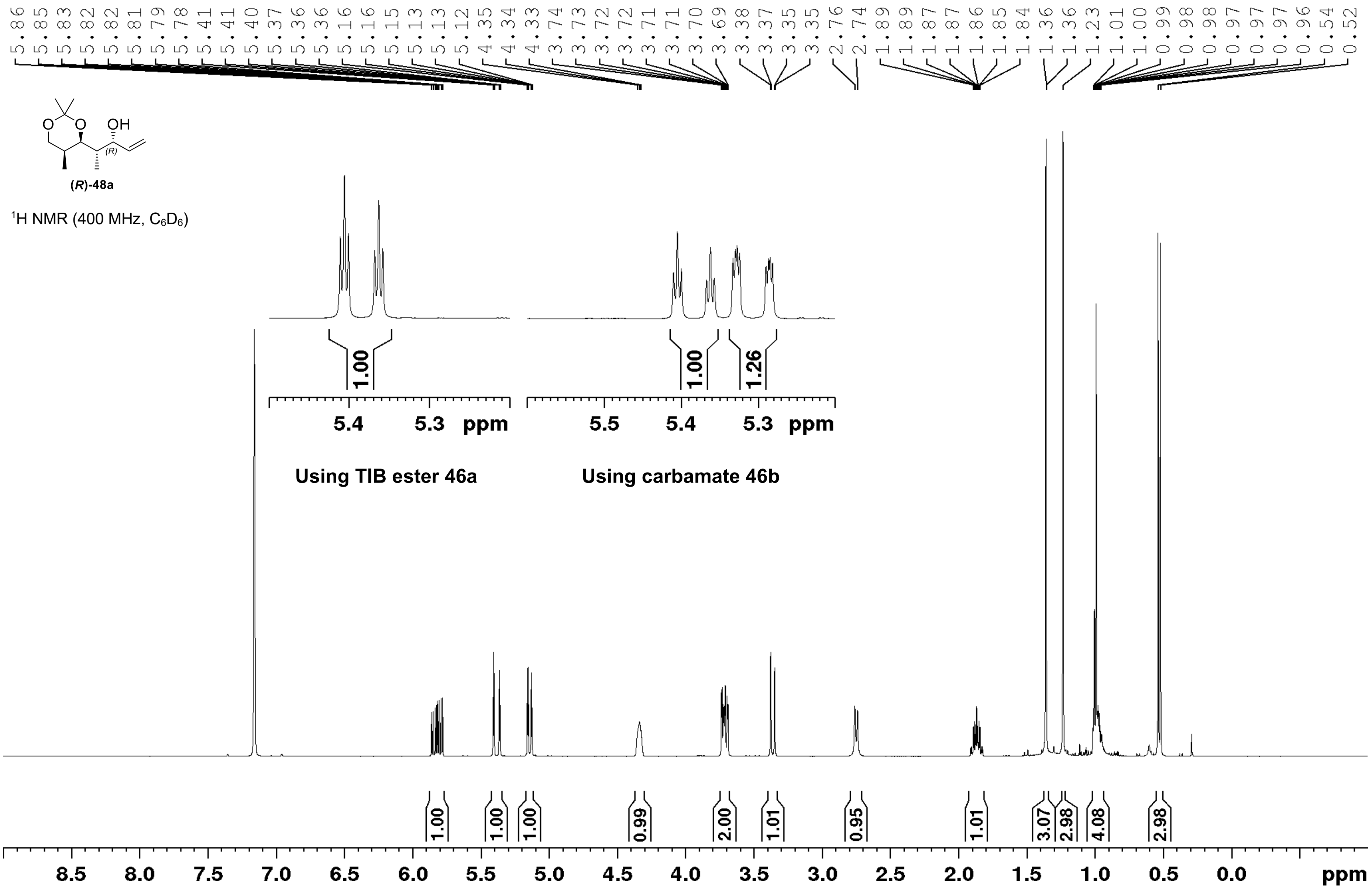

5.86 5.85 5.83 5.82 5.82 5.81 5.79 5.78 5.41 5.41 5.40 5.37 5.36 5.36 5.16 5.16 5.15 5.13 5.13 5.12 4.35 4.34 4.33 3.74 3.73 3.72 3.72 3.71 3.71 3.70 3.69 3.38 3.37 3.35 3.35 2.76 2.74 1.89 1.89 1.87 1.87 1.86 1.85 1.84 1.36 1.36 1.23 1.01 1.00 0.99 0.98 0.98 0.97 0.97 0.97 0.96 0.54 0.52
OH
(R)
(R)-48a
1H NMR (400 MHz, C6D6)
1.00
5.4 5.3 ppm
Using TIB ester 46a
1.00 1.26
5.5 5.4 5.3 ppm
Using carbamate 46b
1.00 1.00 1.00 0.99 2.00 1.01 0.95 1.01 3.07 2.98 4.08 2.98
8.5 8.0 7.5 7.0 6.5 6.0 5.5 5.0 4.5 4.0 3.5 3.0 2.5 2.0 1.5 1.0 0.5 0.0 ppm

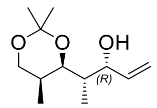

(*R*)-48a

$^{13}C\{^{1}H\}$ NMR (101 MHz, C_6D_6)

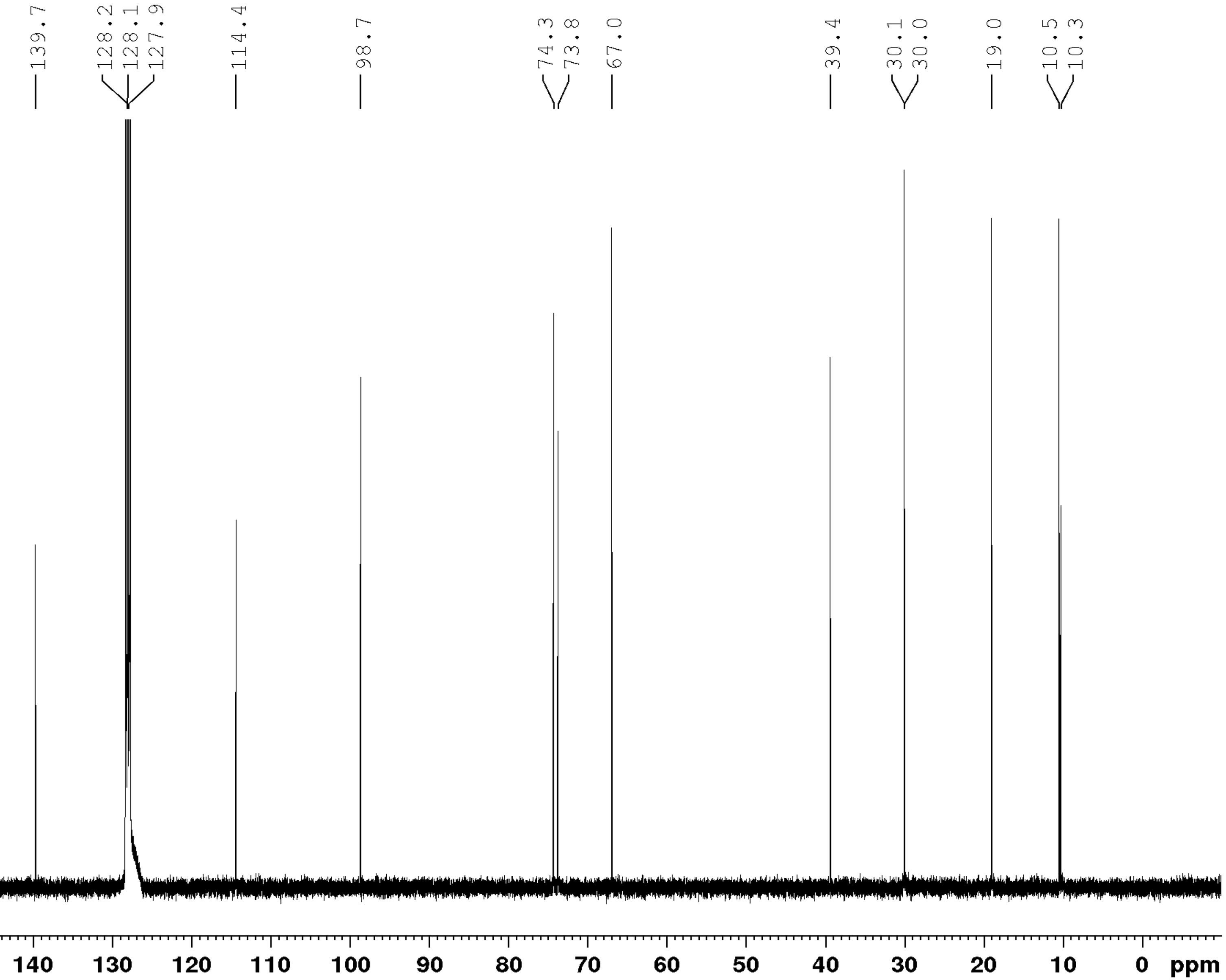

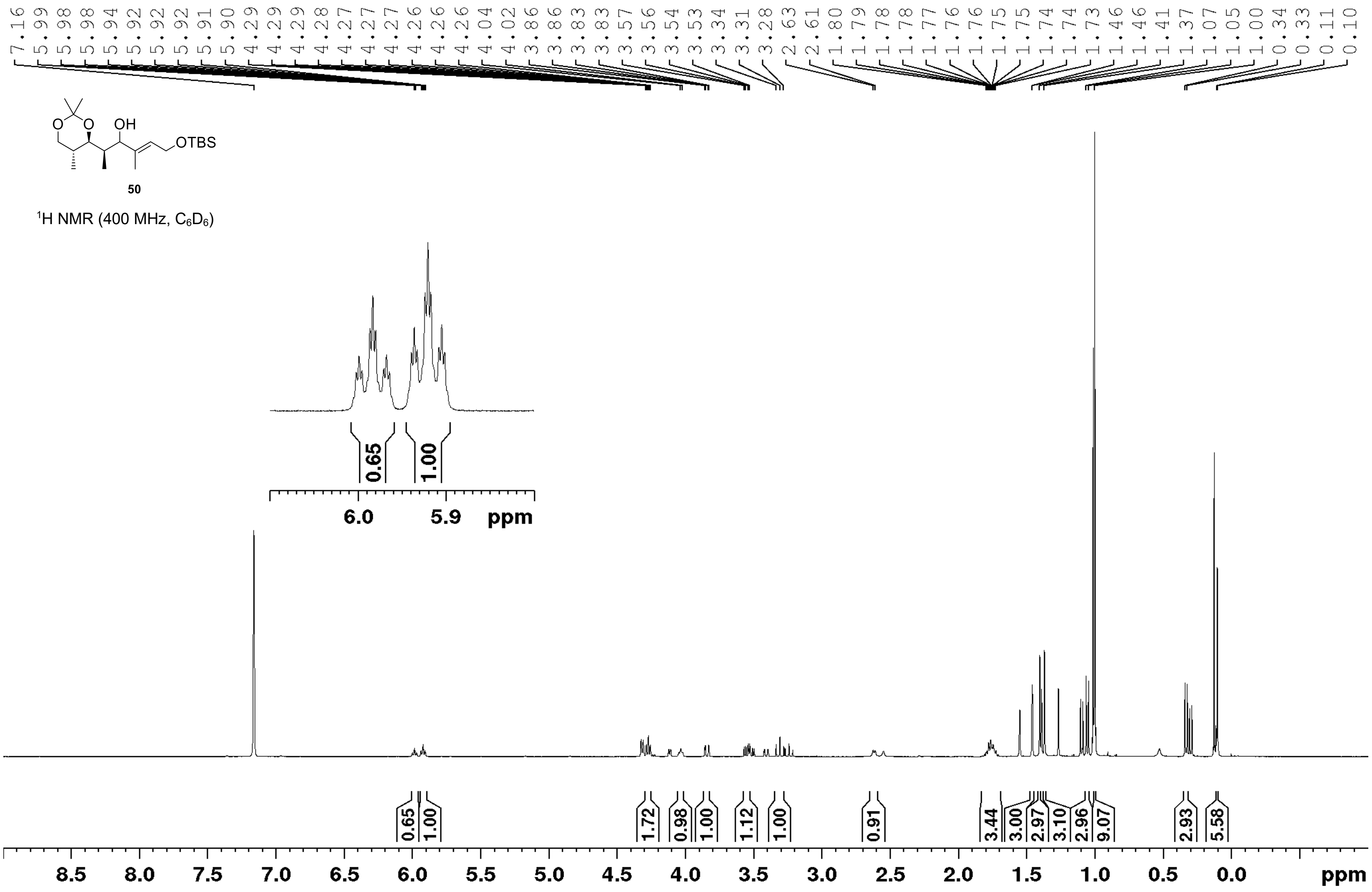

50

^{1}H NMR (400 MHz, C_6D_6)

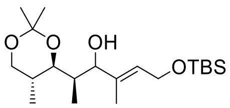

50

$^{13}C\{^1H\}$ NMR (101 MHz, C_6D_6)

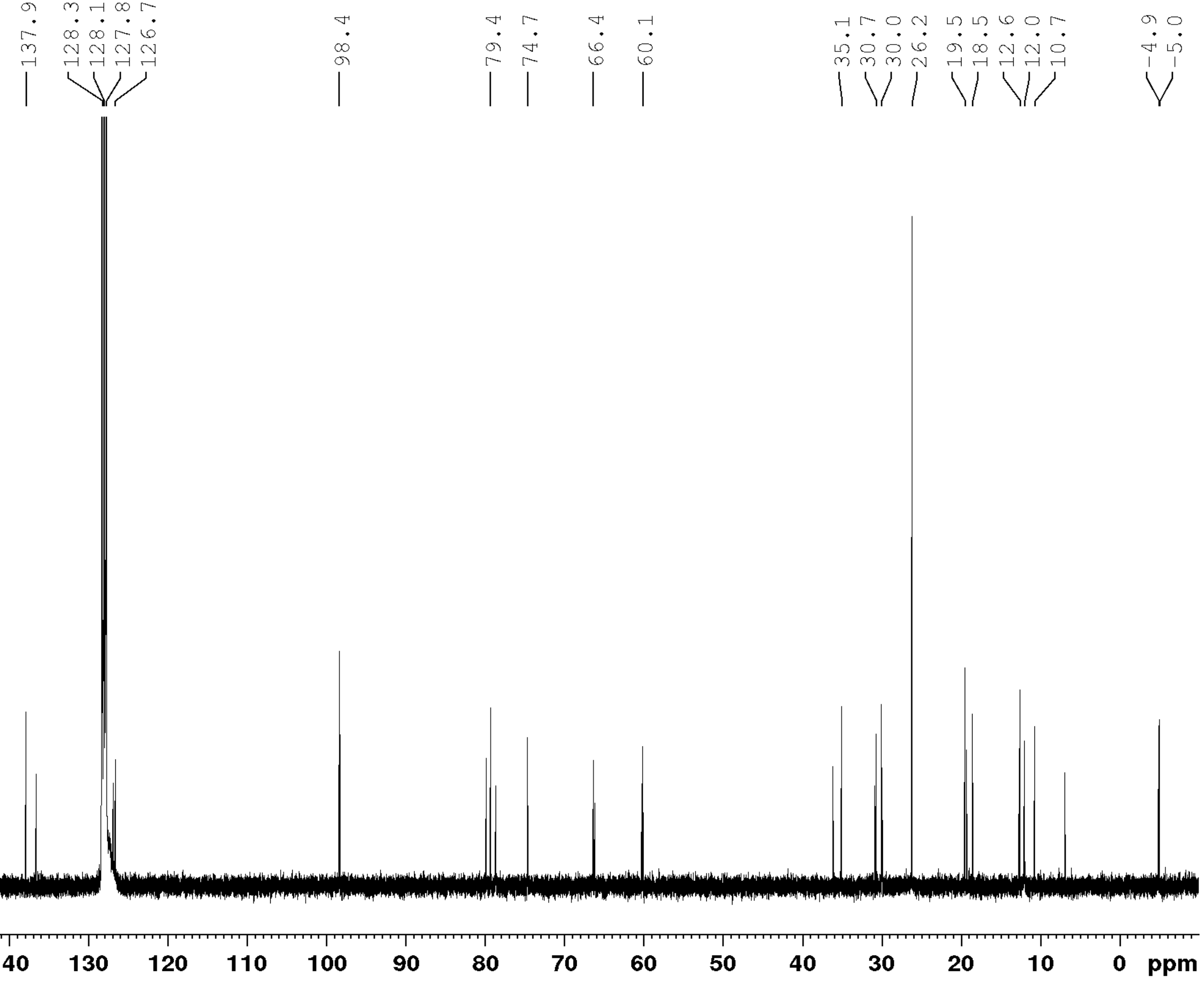

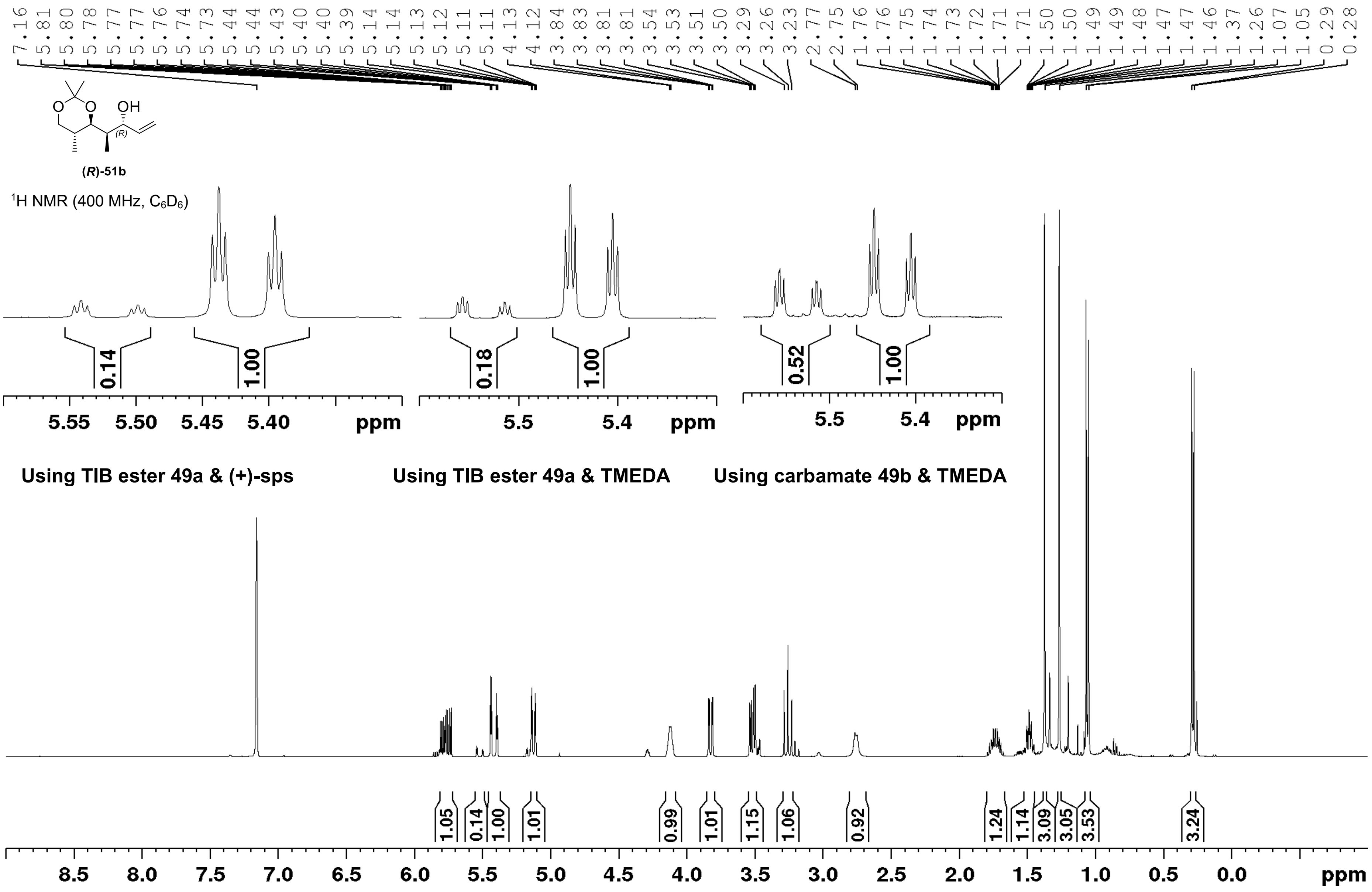

(R)-51b
1H NMR (400 MHz, C6D6)
Using TIB ester 49a & (+)-sps
Using TIB ester 49a & TMEDA
Using carbamate 49b & TMEDA
ppm

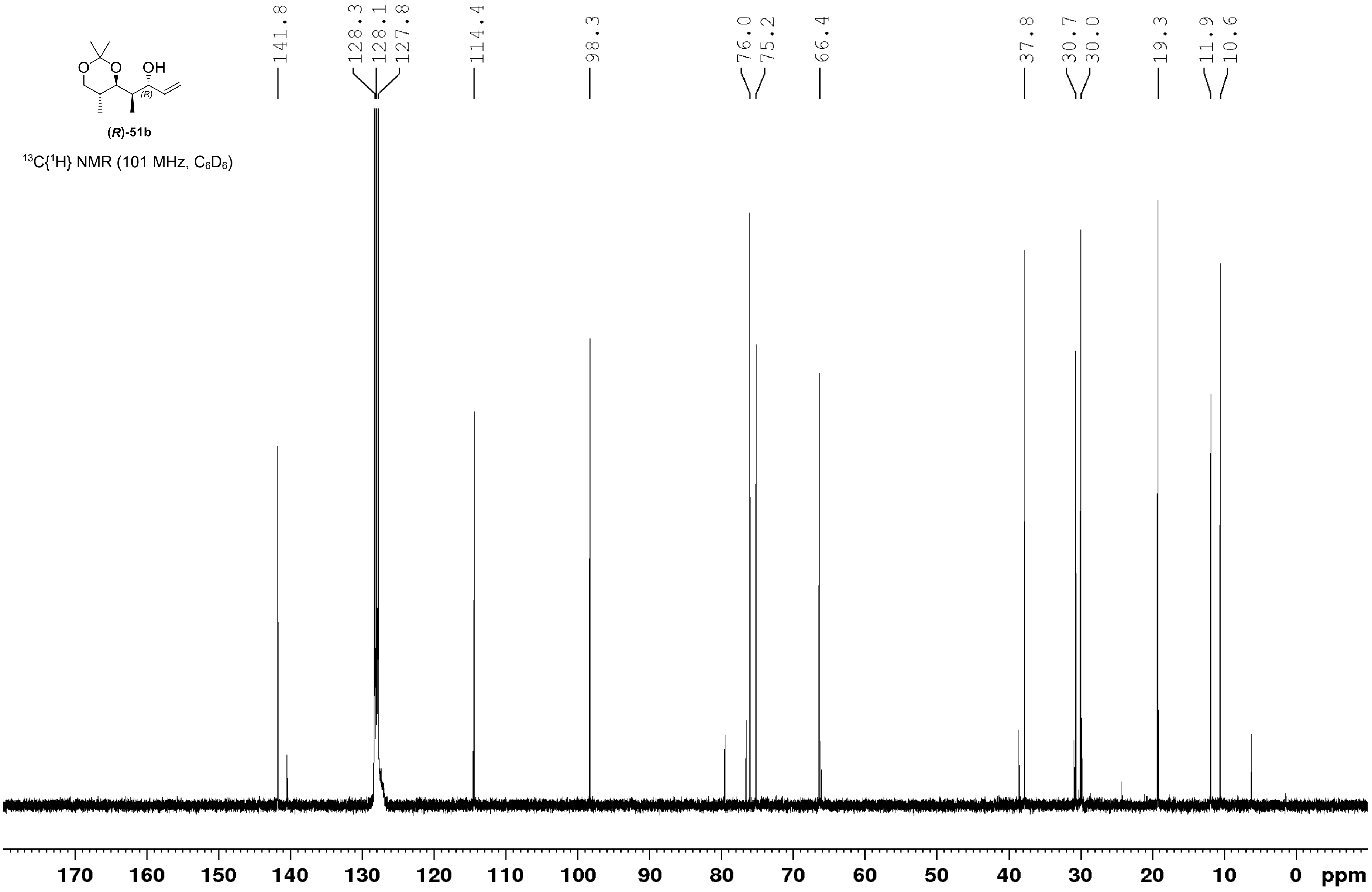
141.8
128.3
128.1
127.8
114.4
98.3
76.0
75.2
66.4
37.8
30.7
30.0
19.3
11.9
10.6
OH
(R)
O
O
(R)-51b
$^{13}C\{^{1}H\}$ NMR (101 MHz, C_6D_6)
170
160
150
140
130
120
110
100
90
80
70
60
50
40
30
20
10
0
ppm

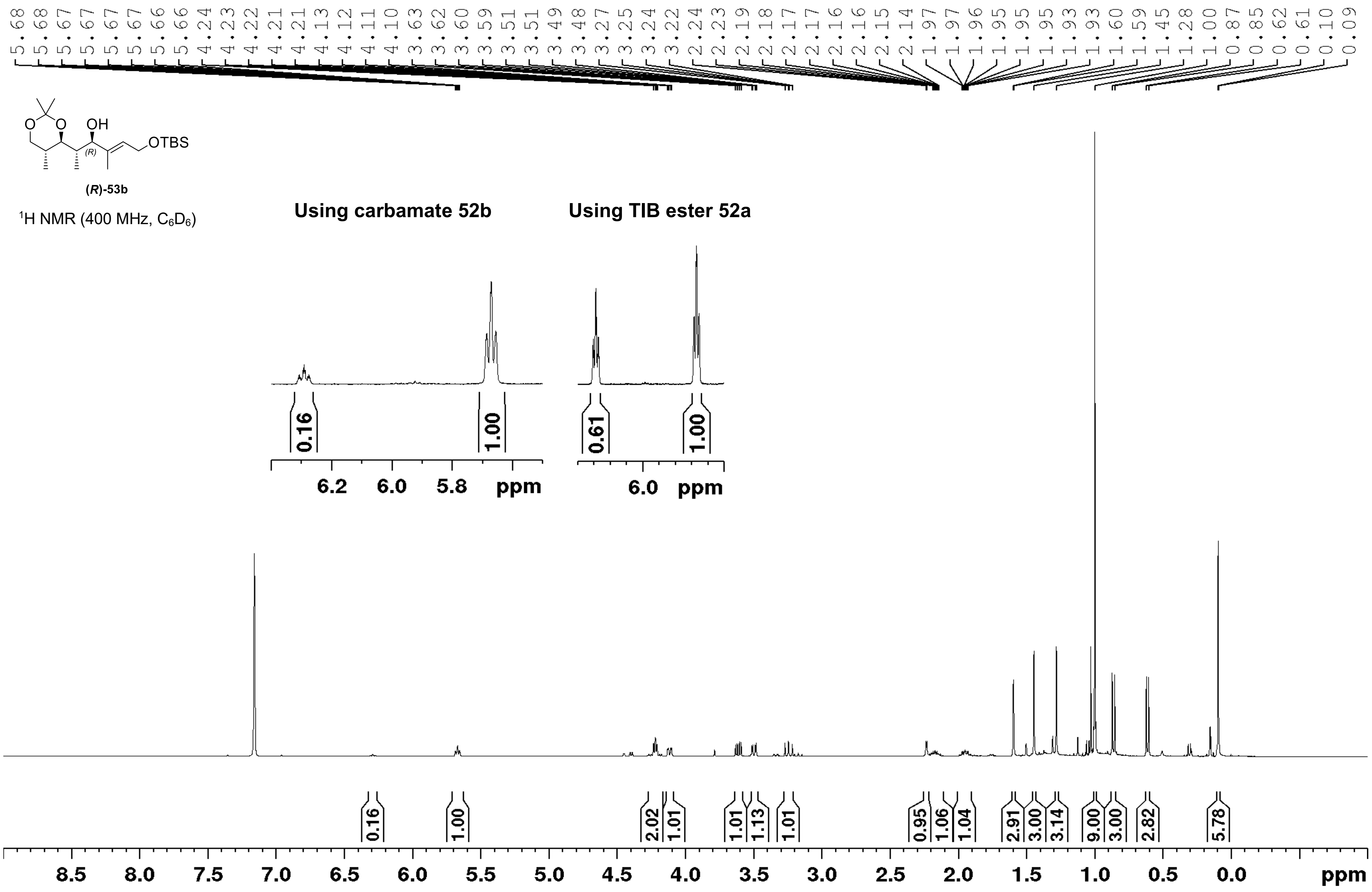

OH
OTBS
O
O
(R)
(R)-53b
1H NMR (400 MHz, C6D6)
Using carbamate 52b
Using TIB ester 52a
5.68 5.68 5.67 5.67 5.67 5.67 5.66 5.66 4.24 4.23 4.22 4.21 4.21 4.13 4.12 4.11 4.10 3.63 3.62 3.60 3.59 3.51 3.51 3.49 3.48 3.27 3.25 3.24 3.22 2.24 2.23 2.19 2.18 2.17 2.17 2.16 2.16 2.15 2.14 1.97 1.97 1.96 1.95 1.95 1.95 1.93 1.93 1.60 1.59 1.45 1.28 1.00 0.87 0.85 0.62 0.61 0.10 0.09
0.16
1.00
0.61
1.00
6.2 6.0 5.8 ppm
6.0 ppm
0.16 1.00 2.02 1.01 1.01 1.13 1.01 0.95 1.06 1.04 2.91 3.00 3.14 9.00 3.00 2.82 5.78
8.5 8.0 7.5 7.0 6.5 6.0 5.5 5.0 4.5 4.0 3.5 3.0 2.5 2.0 1.5 1.0 0.5 0.0 ppm

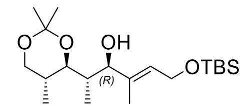

(*R*)-53b

$^{13}C\{^{1}H\}$ NMR (101 MHz, C_6D_6)

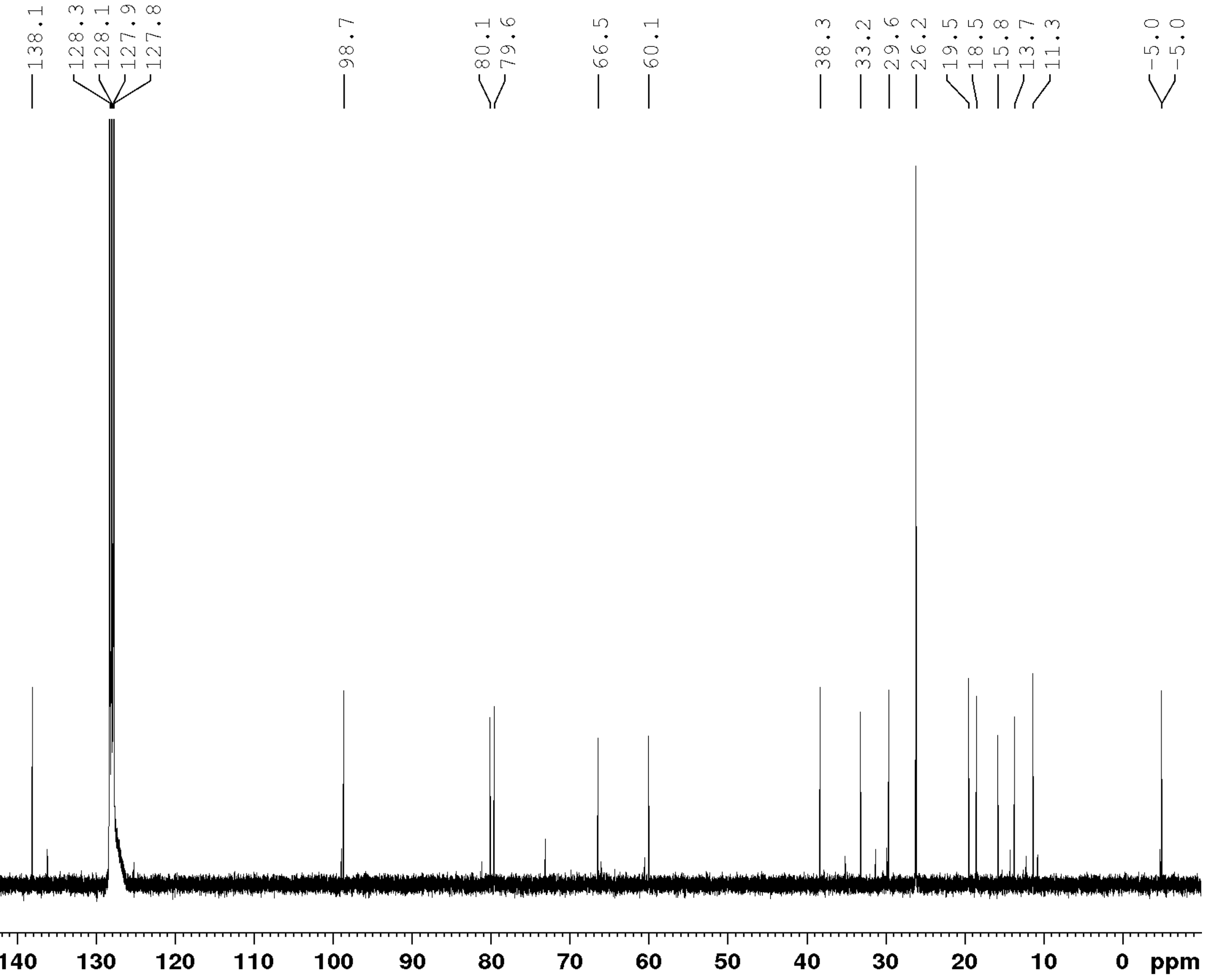

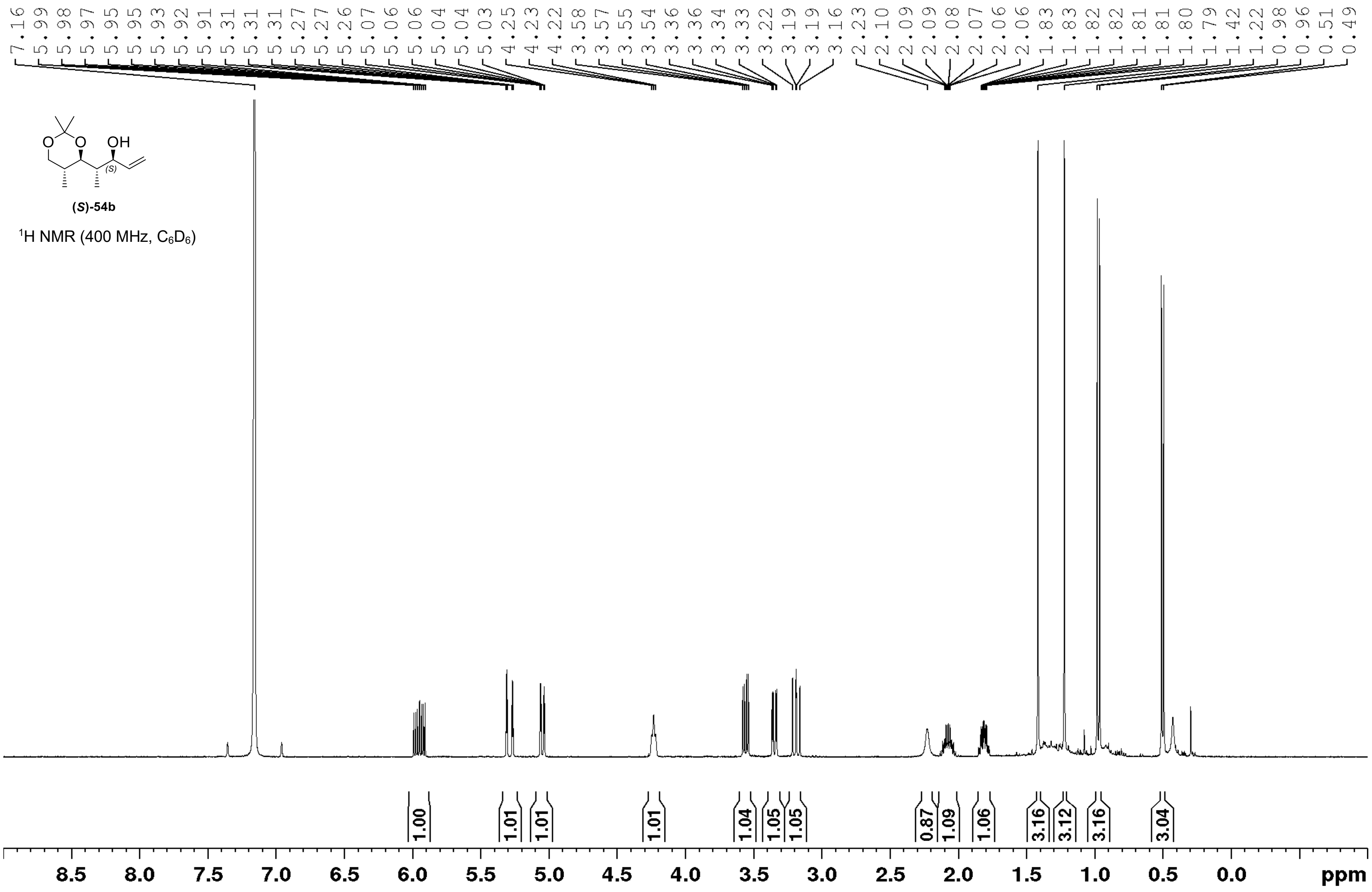

(S)-54b
^{1}H NMR (400 MHz, C_6D_6)
ppm

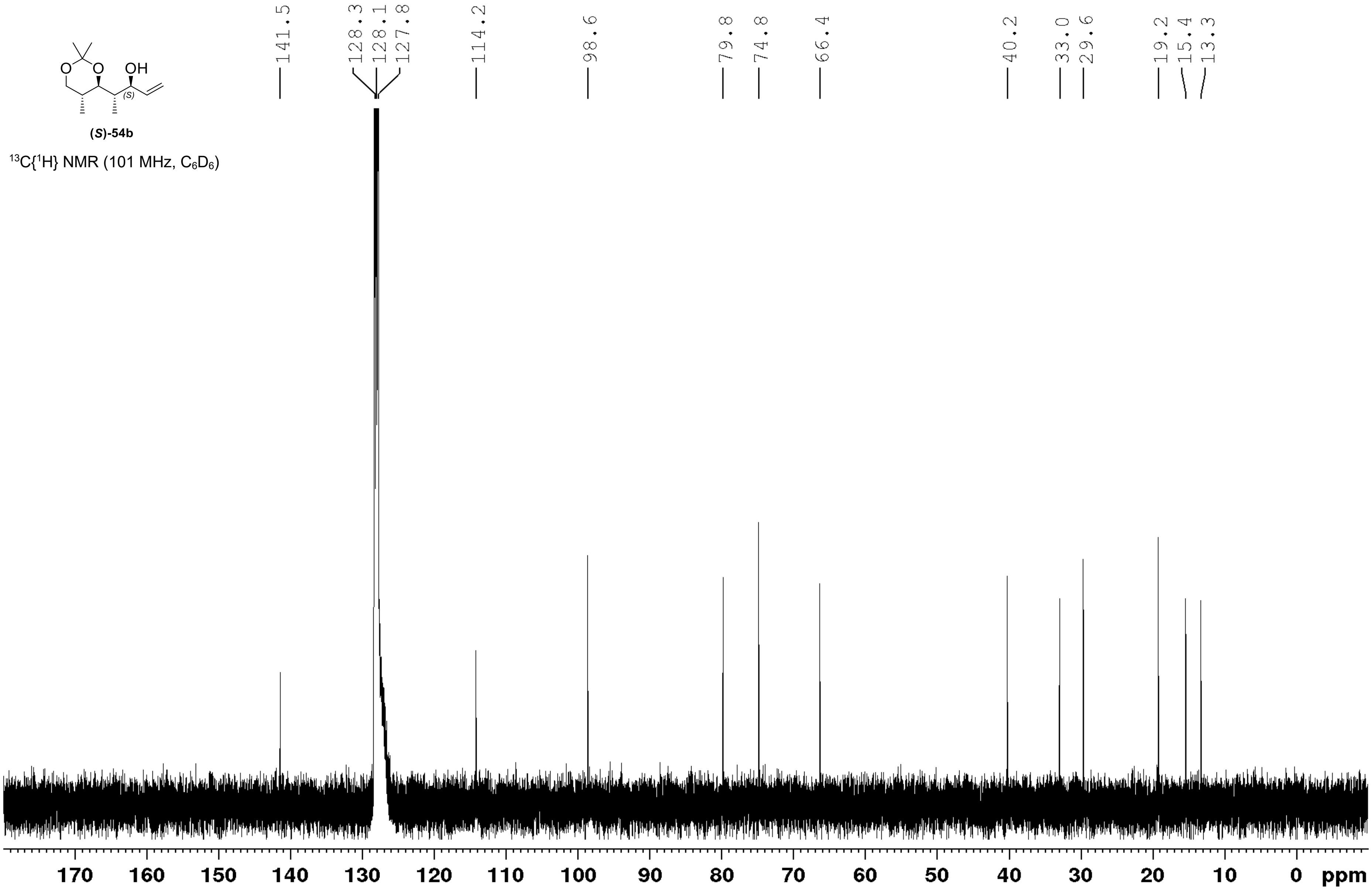
(S)-54b
$^{13}C\{^1H\}$ NMR (101 MHz, C_6D_6)
141.5
128.3
128.1
127.8
114.2
98.6
79.8
74.8
66.4
40.2
33.0
29.6
19.2
15.4
13.3
170 160 150 140 130 120 110 100 90 80 70 60 50 40 30 20 10 0 ppm

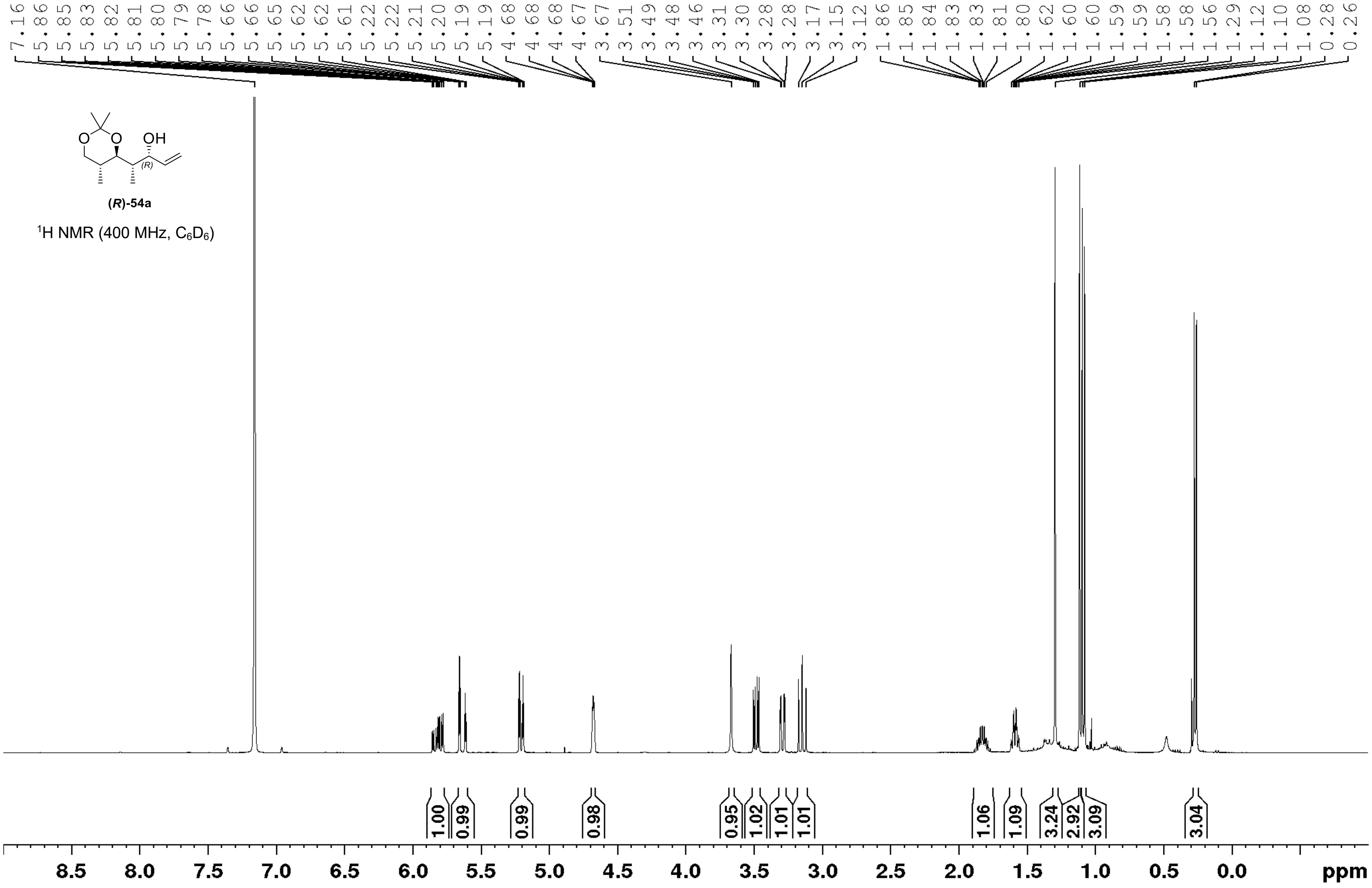

(*R*)-54a

^{1}H NMR (400 MHz, C_6D_6)

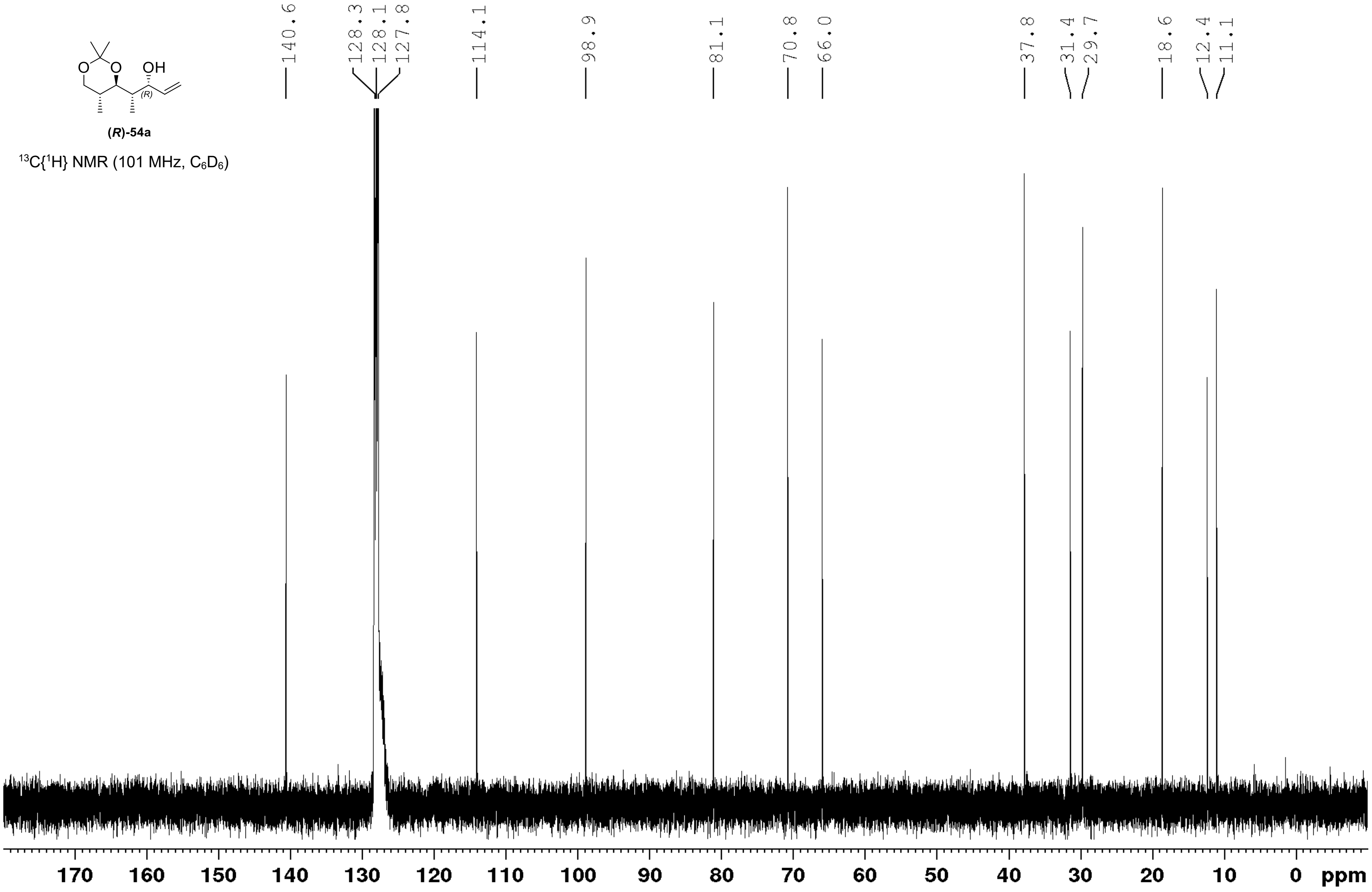
OH
O
O
(R)
(R)-54a
$^{13}C\{^{1}H\}$ NMR (101 MHz, C_6D_6)
140.6
128.3
128.1
127.8
114.1
98.9
81.1
70.8
66.0
37.8
31.4
29.7
18.6
12.4
11.1
170
160
150
140
130
120
110
100
90
80
70
60
50
40
30
20
10
0
ppm

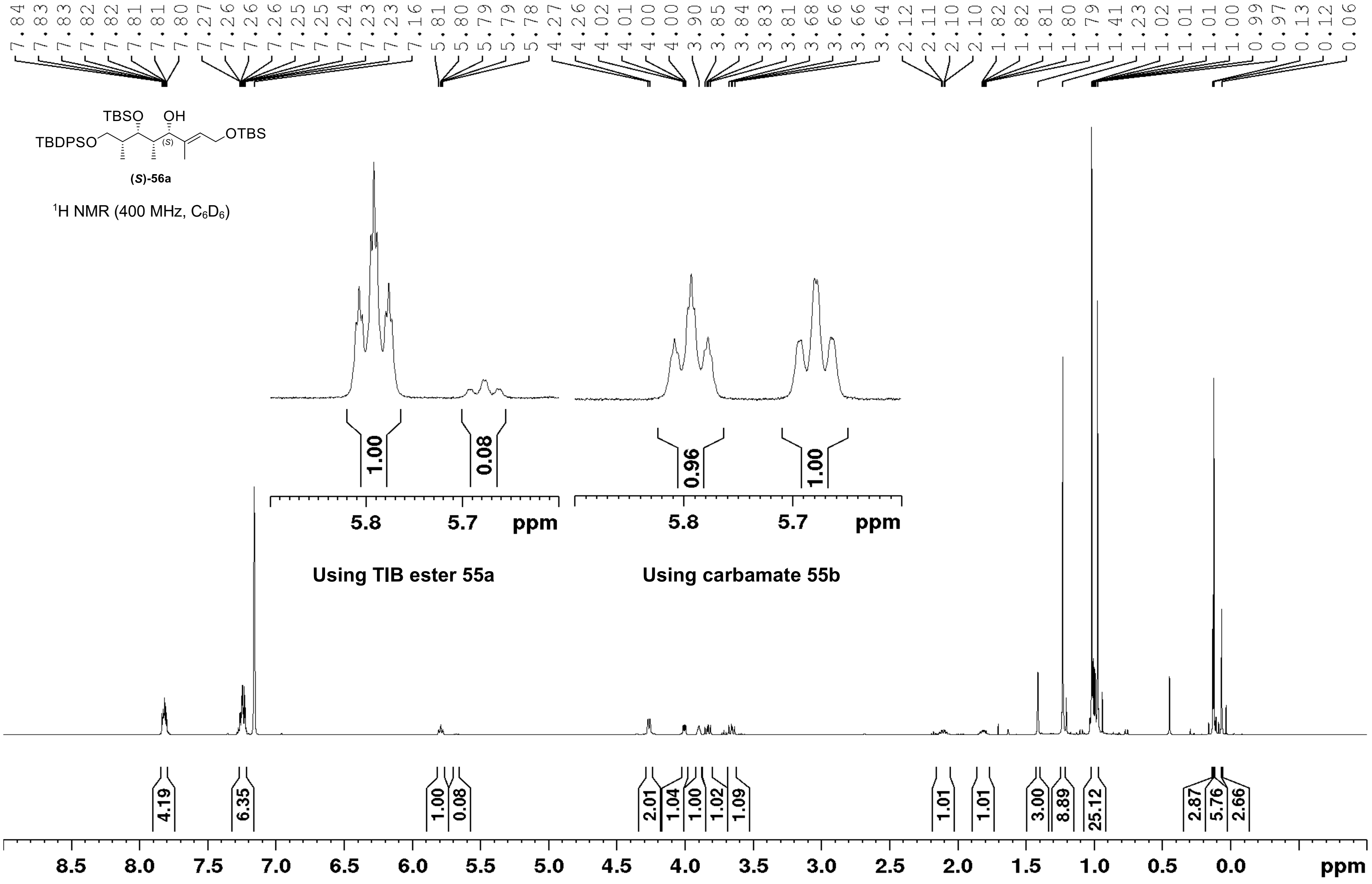
TBSO OH
TBDPSO OTBS
(S)-56a
¹H NMR (400 MHz, C6D6)
Using TIB ester 55a
Using carbamate 55b
ppm

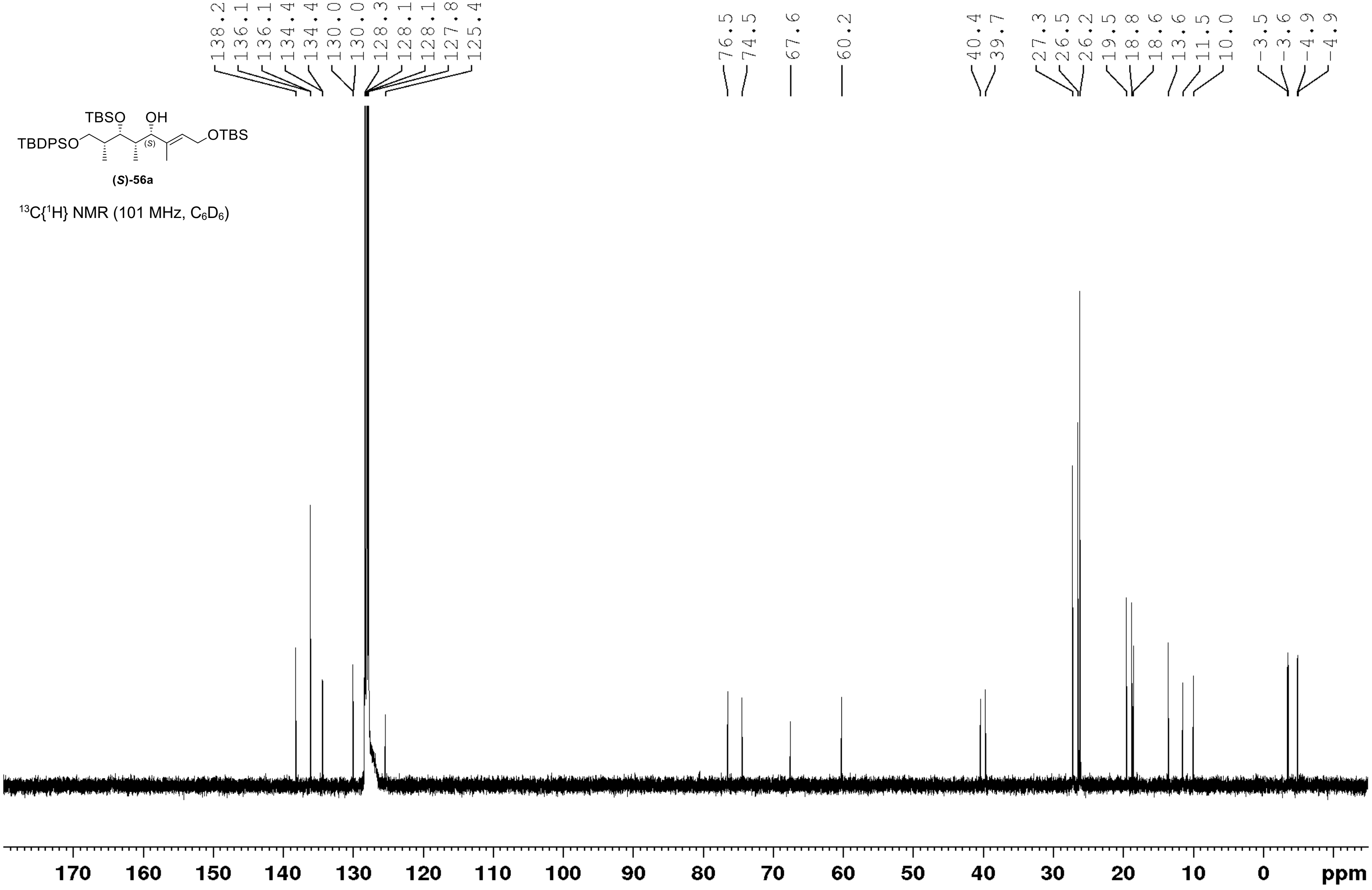

(*S*)-56a

$^{13}C\{^{1}H\}$ NMR (101 MHz, C_6D_6)

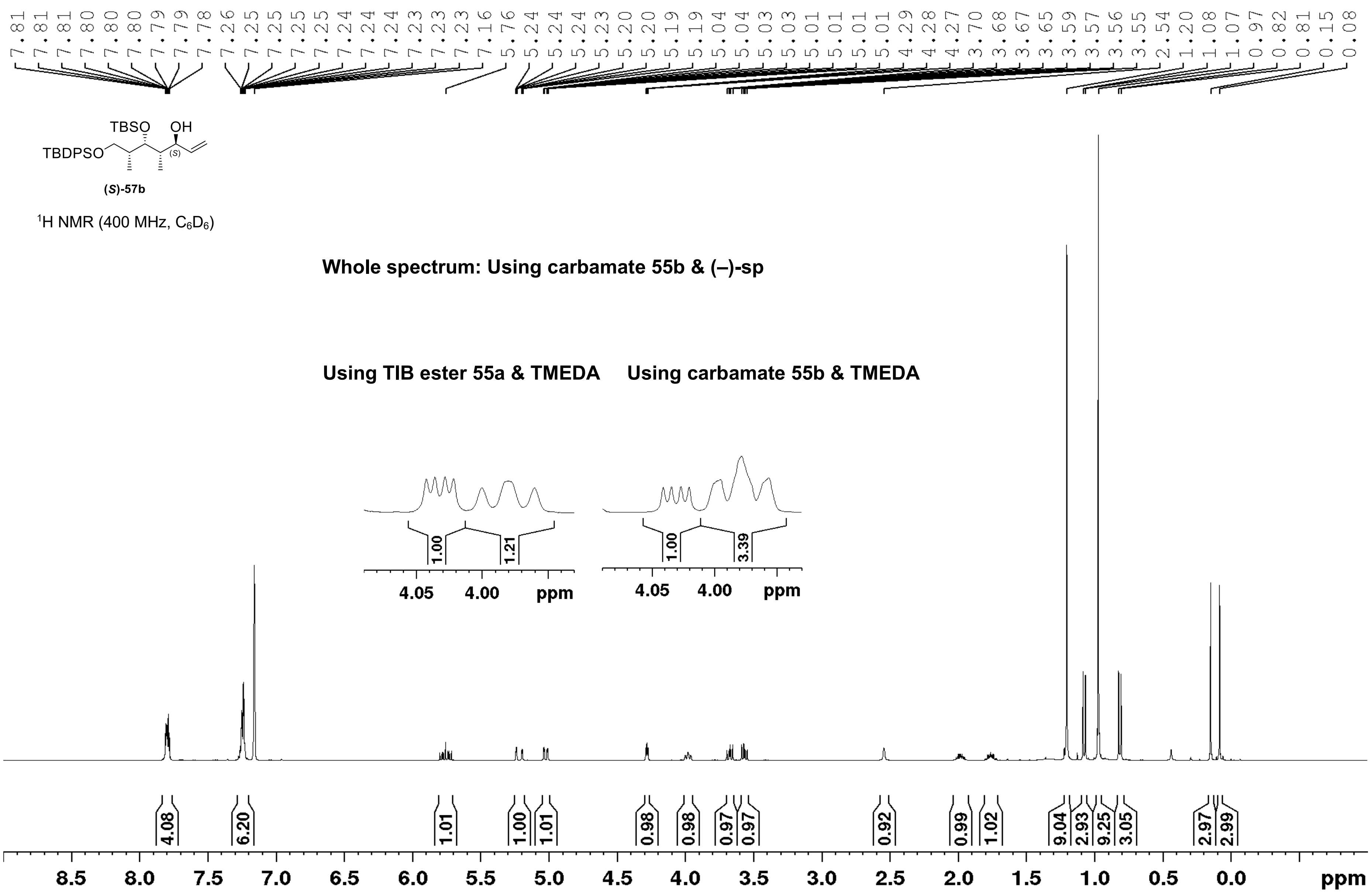

TBSO OH
TBDPSO
(S)
(S)-57b
^{1}H NMR (400 MHz, C_6D_6)
Whole spectrum: Using carbamate 55b & (–)-sp
Using TIB ester 55a & TMEDA
Using carbamate 55b & TMEDA
7.81 7.81 7.81 7.80 7.80 7.80 7.79 7.79 7.78 7.26 7.25 7.25 7.25 7.25 7.24 7.24 7.24 7.23 7.23 7.23 7.16 5.76 5.24 5.24 5.24 5.23 5.20 5.20 5.19 5.19 5.04 5.04 5.03 5.03 5.01 5.01 5.01 4.29 4.28 4.27 3.70 3.68 3.67 3.65 3.59 3.57 3.56 3.55 2.54 1.20 1.08 1.07 0.97 0.82 0.81 0.15 0.08
4.08 6.20 1.01 1.00 1.01 0.98 0.98 0.97 0.97 0.92 0.99 1.02 9.04 2.93 9.25 3.05 2.97 2.99
1.00 1.21
1.00 3.39
4.05 4.00 ppm
8.5 8.0 7.5 7.0 6.5 6.0 5.5 5.0 4.5 4.0 3.5 3.0 2.5 2.0 1.5 1.0 0.5 0.0 ppm

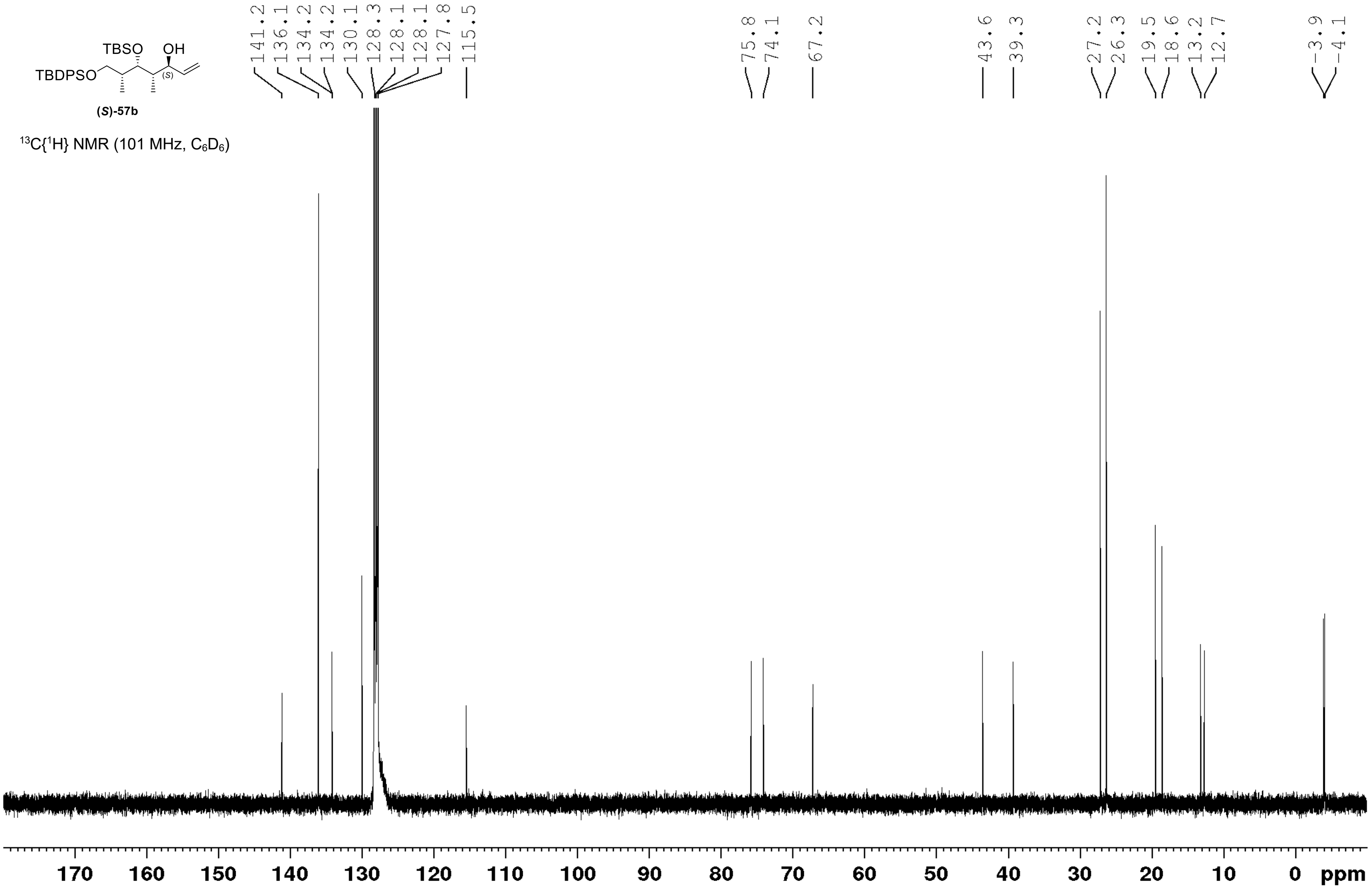
TBSO
OH
TBDPSO
(S)
(S)-57b
$^{13}C\{^1H\}$ NMR (101 MHz, C_6D_6)
141.2
136.1
134.2
134.2
130.1
128.3
128.1
128.1
127.8
115.5
75.8
74.1
67.2
43.6
39.3
27.2
26.3
19.5
18.6
13.2
12.7
-3.9
-4.1
170
160
150
140
130
120
110
100
90
80
70
60
50
40
30
20
10
0
ppm

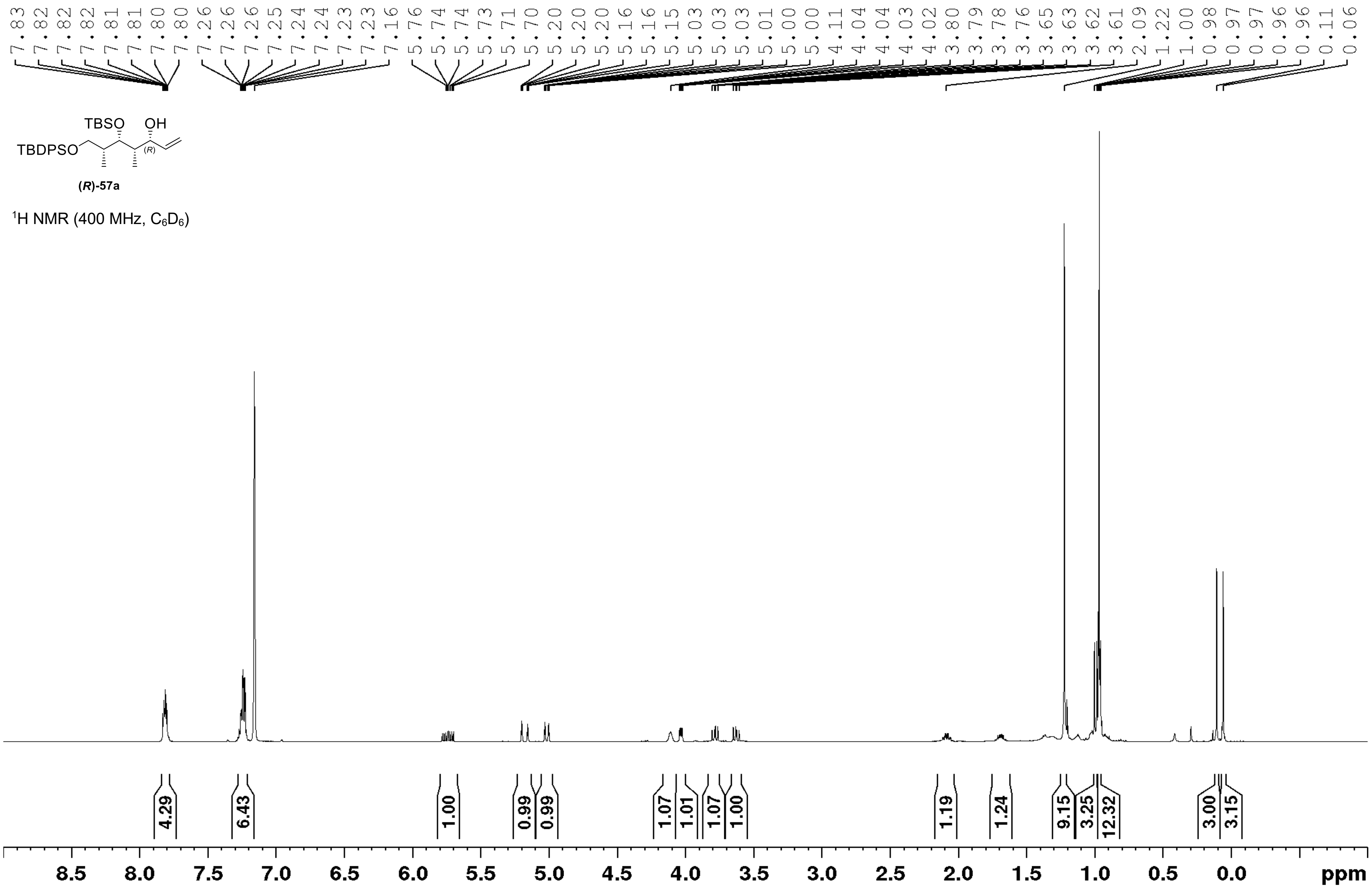
7.83 7.82 7.82 7.82 7.81 7.81 7.80 7.80 7.26 7.26 7.26 7.25 7.24 7.24 7.23 7.23 7.16 5.76 5.74 5.74 5.73 5.71 5.70 5.20 5.20 5.20 5.16 5.16 5.15 5.03 5.03 5.03 5.01 5.00 5.00 4.11 4.04 4.04 4.03 4.02 3.80 3.79 3.78 3.76 3.65 3.63 3.62 3.61 2.09 1.22 1.00 0.98 0.97 0.97 0.96 0.96 0.11 0.06
TBSO
OH
TBDPSO
(R)
(R)-57a
¹H NMR (400 MHz, C6D6)
4.29
6.43
1.00
0.99
0.99
1.07
1.01
1.07
1.00
1.19
1.24
9.15
3.25
12.32
3.00
3.15
8.5
8.0
7.5
7.0
6.5
6.0
5.5
5.0
4.5
4.0
3.5
3.0
2.5
2.0
1.5
1.0
0.5
0.0
ppm

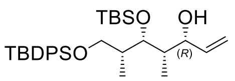

(*R*)-57a

$^{13}C\{^{1}H\}$ NMR (101 MHz, C_6D_6)

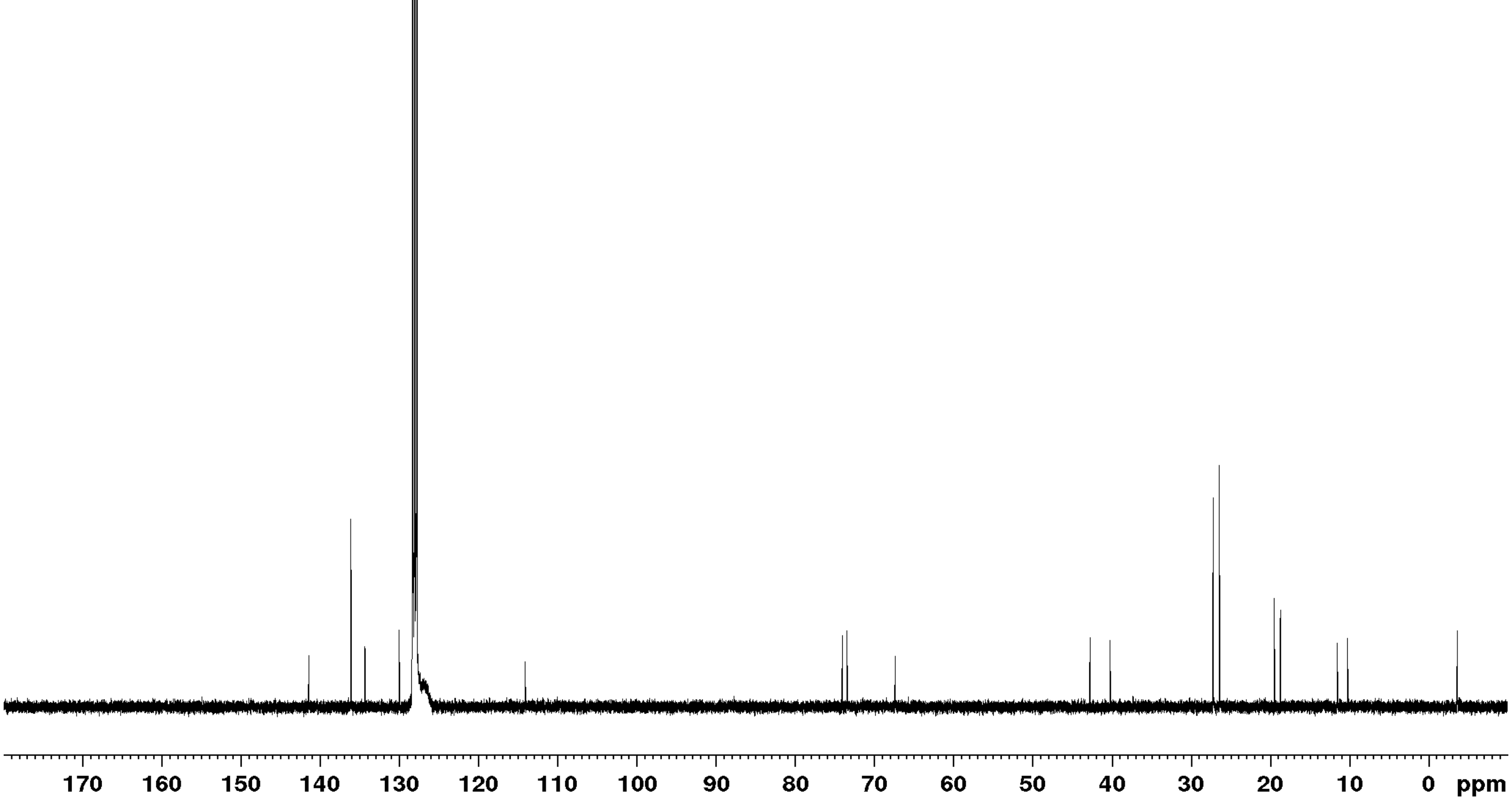

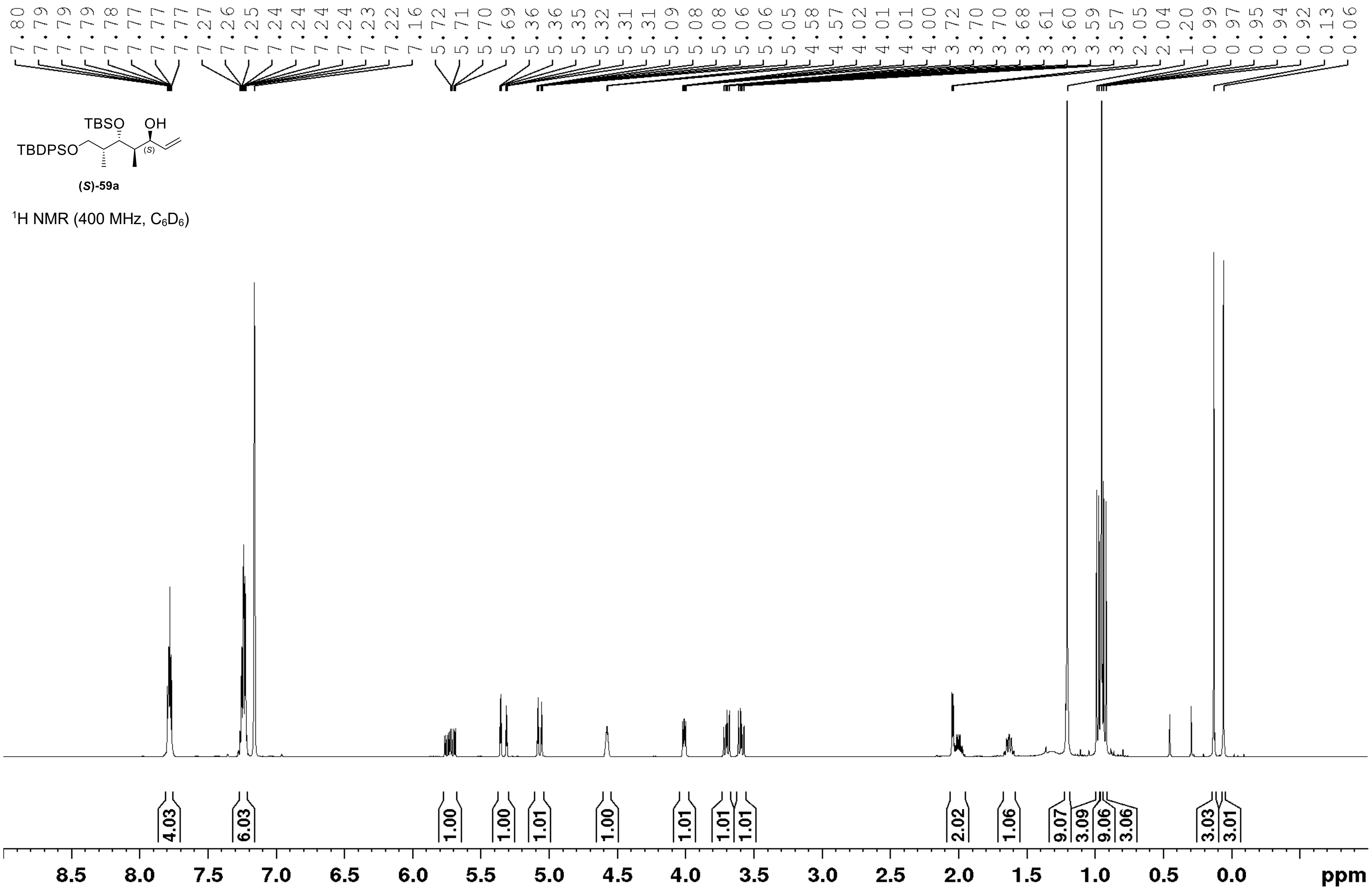

7.80 7.79 7.79 7.79 7.78 7.77 7.77 7.77 7.27 7.26 7.25 7.24 7.24 7.24 7.24 7.23 7.22 7.16 5.72 5.71 5.70 5.69 5.36 5.36 5.35 5.32 5.31 5.31 5.09 5.08 5.08 5.06 5.06 5.05 4.58 4.57 4.02 4.01 4.01 4.00 3.72 3.70 3.70 3.68 3.61 3.60 3.59 3.57 2.05 2.04 1.20 0.99 0.97 0.95 0.94 0.92 0.13 0.06
TBSO OH
TBDPSO
(S)
(S)-59a
1H NMR (400 MHz, C6D6)
4.03 6.03 1.00 1.00 1.01 1.00 1.01 1.01 1.01 2.02 1.06 9.07 3.09 9.06 3.06 3.03 3.01
8.5 8.0 7.5 7.0 6.5 6.0 5.5 5.0 4.5 4.0 3.5 3.0 2.5 2.0 1.5 1.0 0.5 0.0 ppm

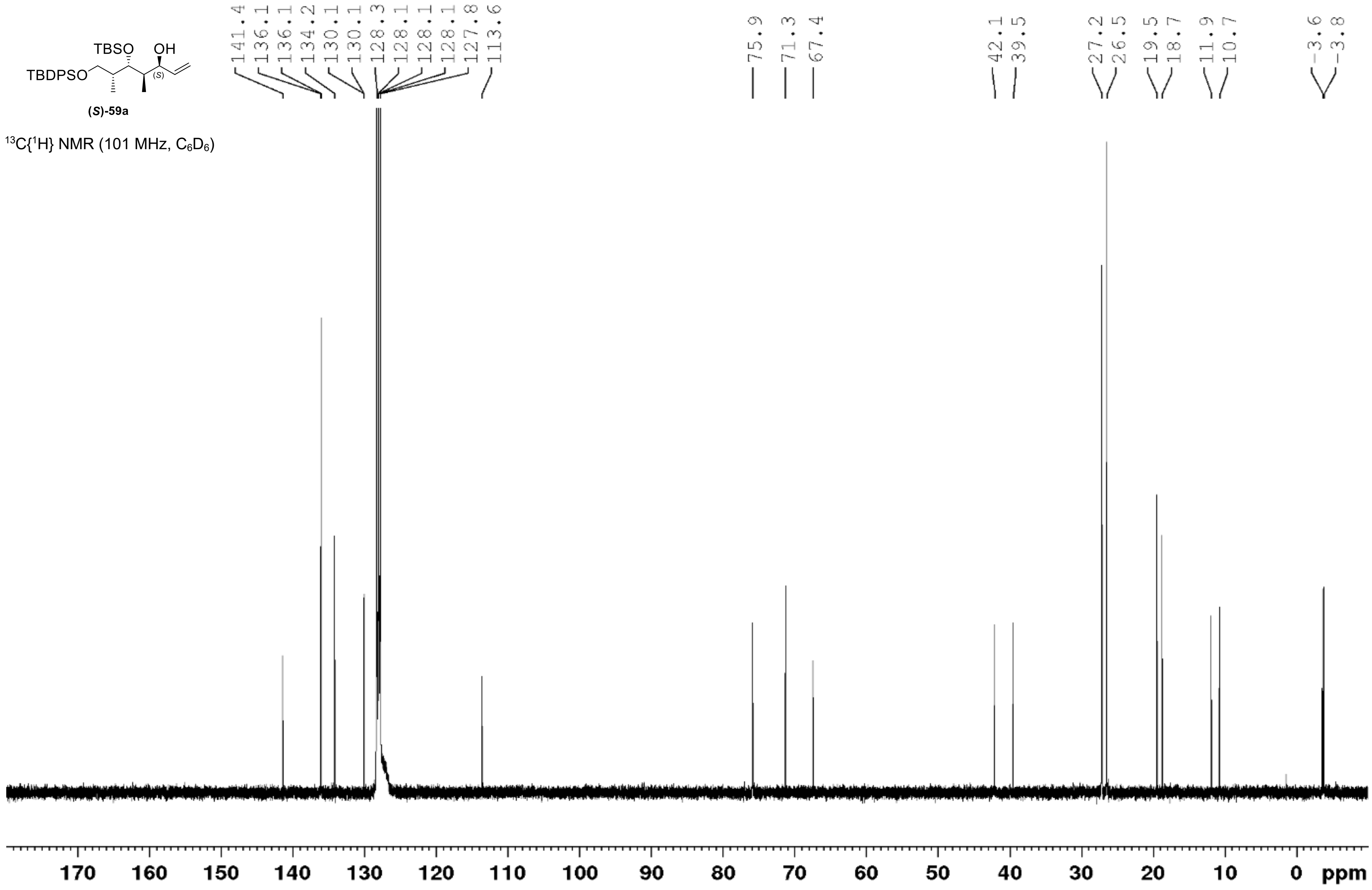
TBSO
OH
TBDPSO
(S)
(S)-59a
$^{13}C\{^1H\}$ NMR (101 MHz, C_6D_6)
141.4
136.1
136.1
134.2
130.1
130.1
128.3
128.1
128.1
128.1
127.8
113.6
75.9
71.3
67.4
42.1
39.5
27.2
26.5
19.5
18.7
11.9
10.7
-3.6
-3.8
170
160
150
140
130
120
110
100
90
80
70
60
50
40
30
20
10
0
ppm

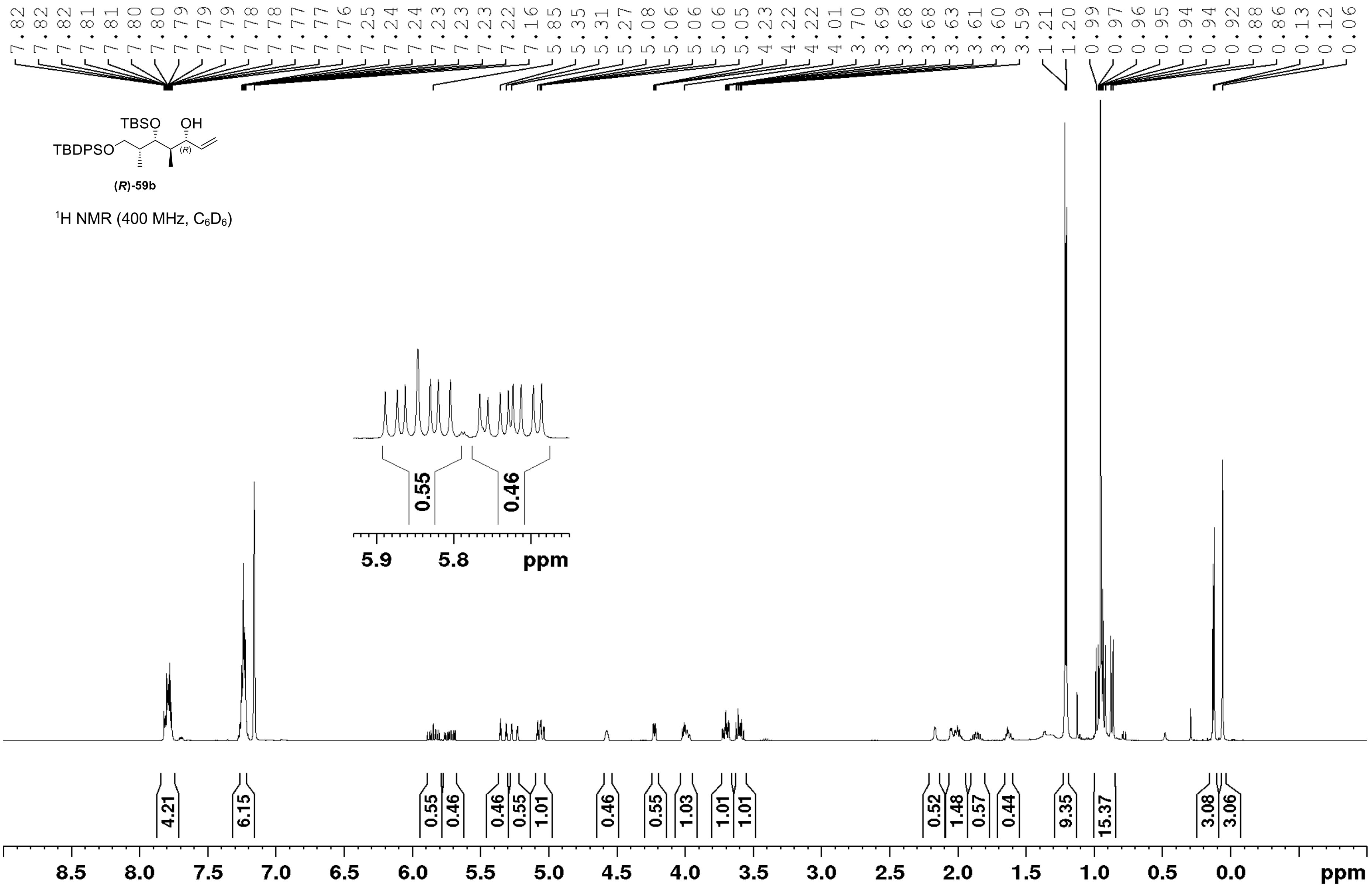
TBSO OH
TBDPSO
(R)
(R)-59b
1H NMR (400 MHz, C6D6)
0.55
0.46
5.9
5.8
ppm
4.21
6.15
0.55
0.46
0.46
0.55
1.01
0.46
0.55
1.03
1.01
1.01
0.52
1.48
0.57
0.44
9.35
15.37
3.08
3.06
8.5 8.0 7.5 7.0 6.5 6.0 5.5 5.0 4.5 4.0 3.5 3.0 2.5 2.0 1.5 1.0 0.5 0.0 ppm

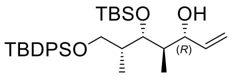

(*R*)-59b

$^{13}C\{^{1}H\}$ NMR (101 MHz, C_6D_6)

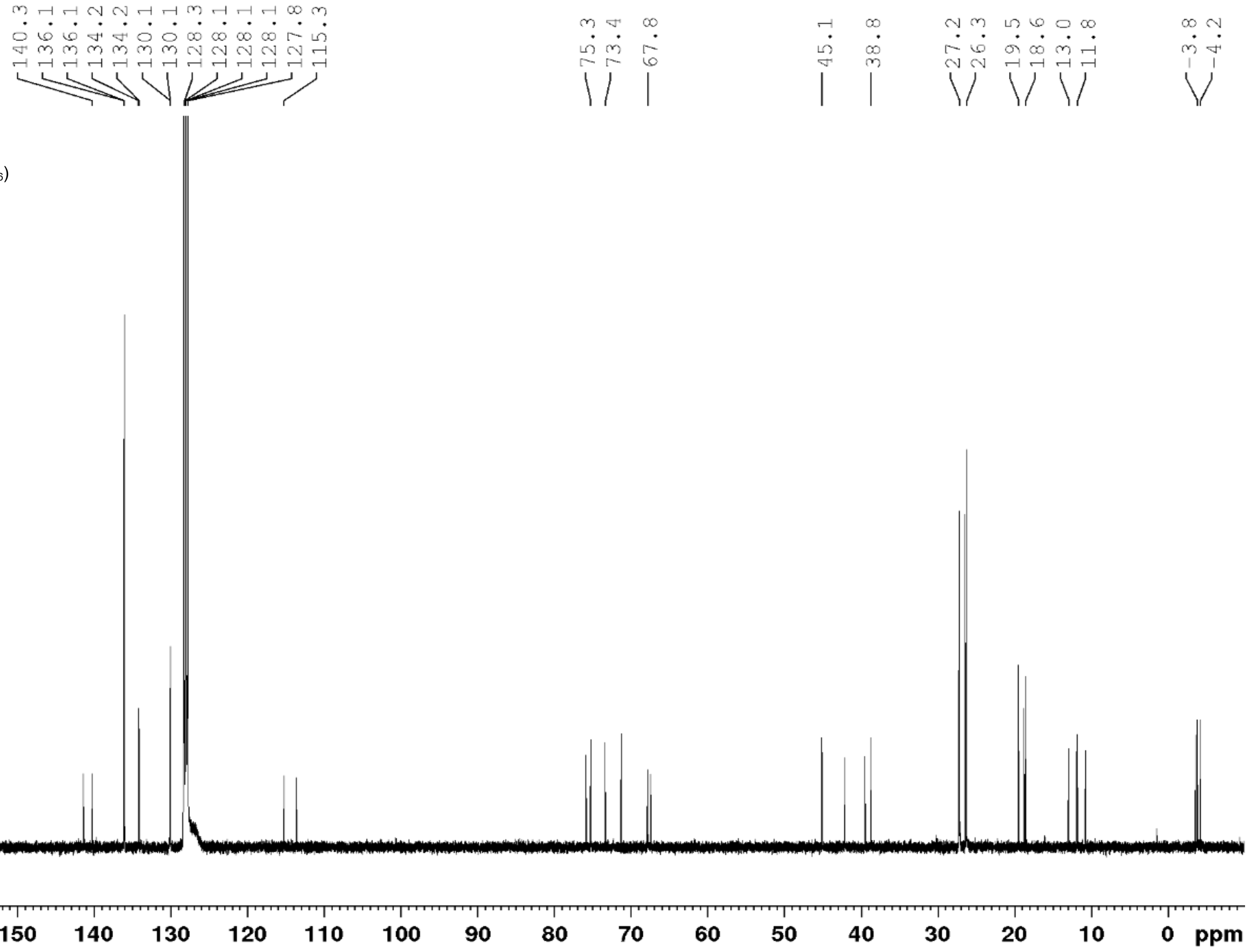

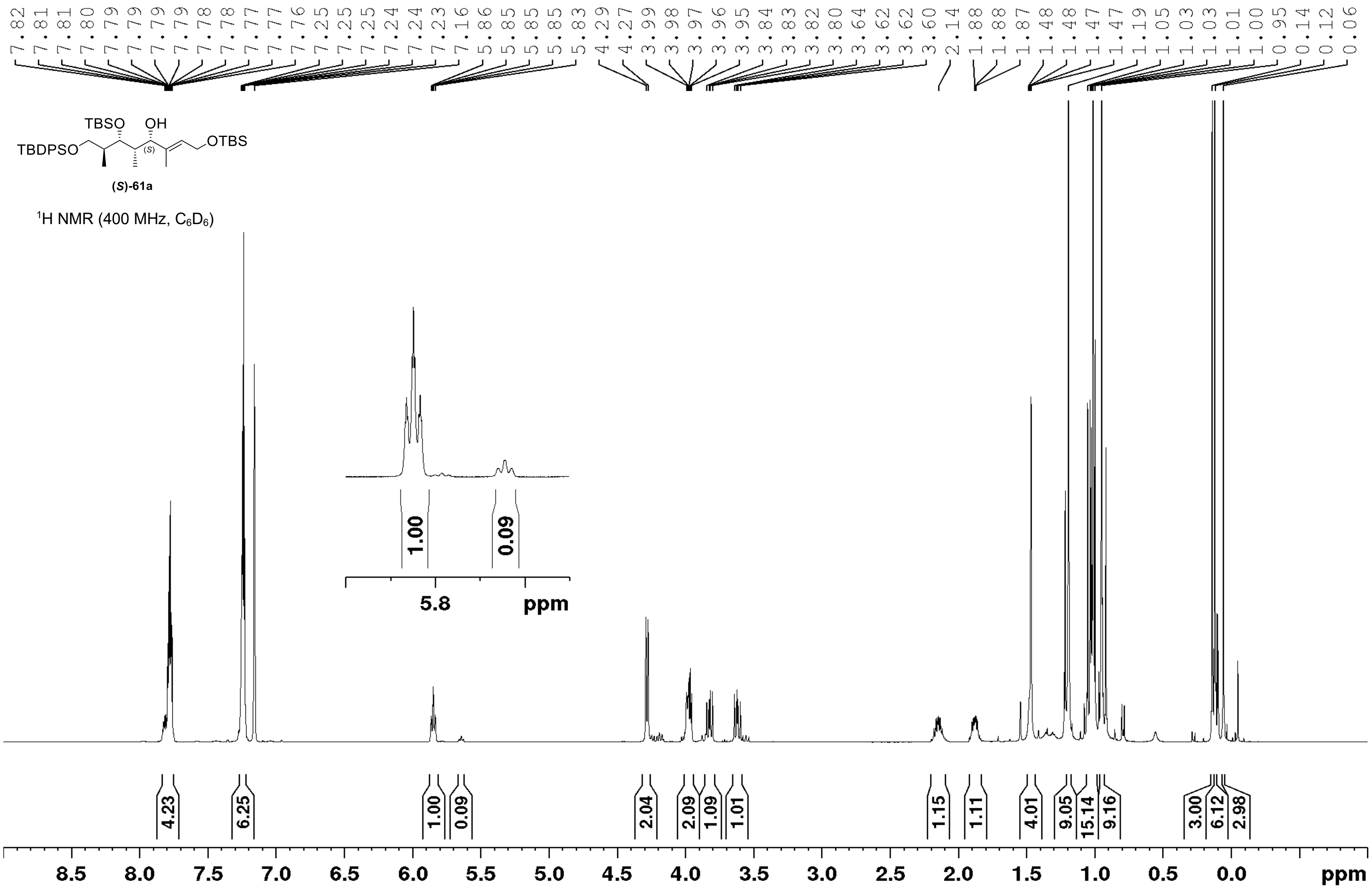
TBSO
OH
TBDPSO
OTBS
(S)
(S)-61a
1H NMR (400 MHz, C6D6)
ppm

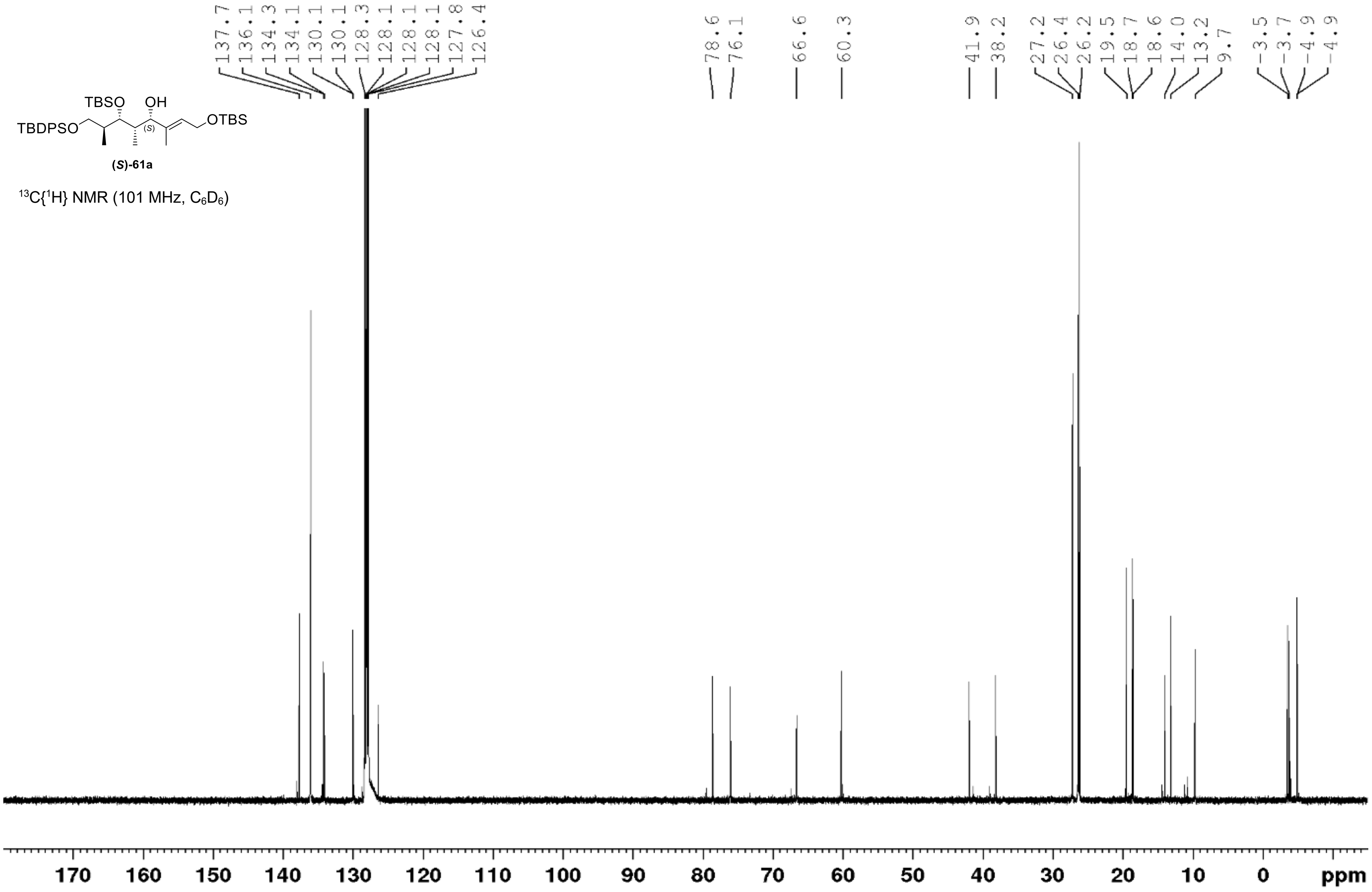
TBSO
OH
TBDPSO
(S)
OTBS
(S)-61a
$^{13}C\{^1H\}$ NMR (101 MHz, C_6D_6)
137.7
136.1
134.3
134.1
130.1
130.1
128.3
128.1
128.1
128.1
127.8
126.4
78.6
76.1
66.6
60.3
41.9
38.2
27.2
26.4
26.2
19.5
18.7
18.6
14.0
13.2
9.7
-3.5
-3.7
-4.9
-4.9
170
160
150
140
130
120
110
100
90
80
70
60
50
40
30
20
10
0
ppm

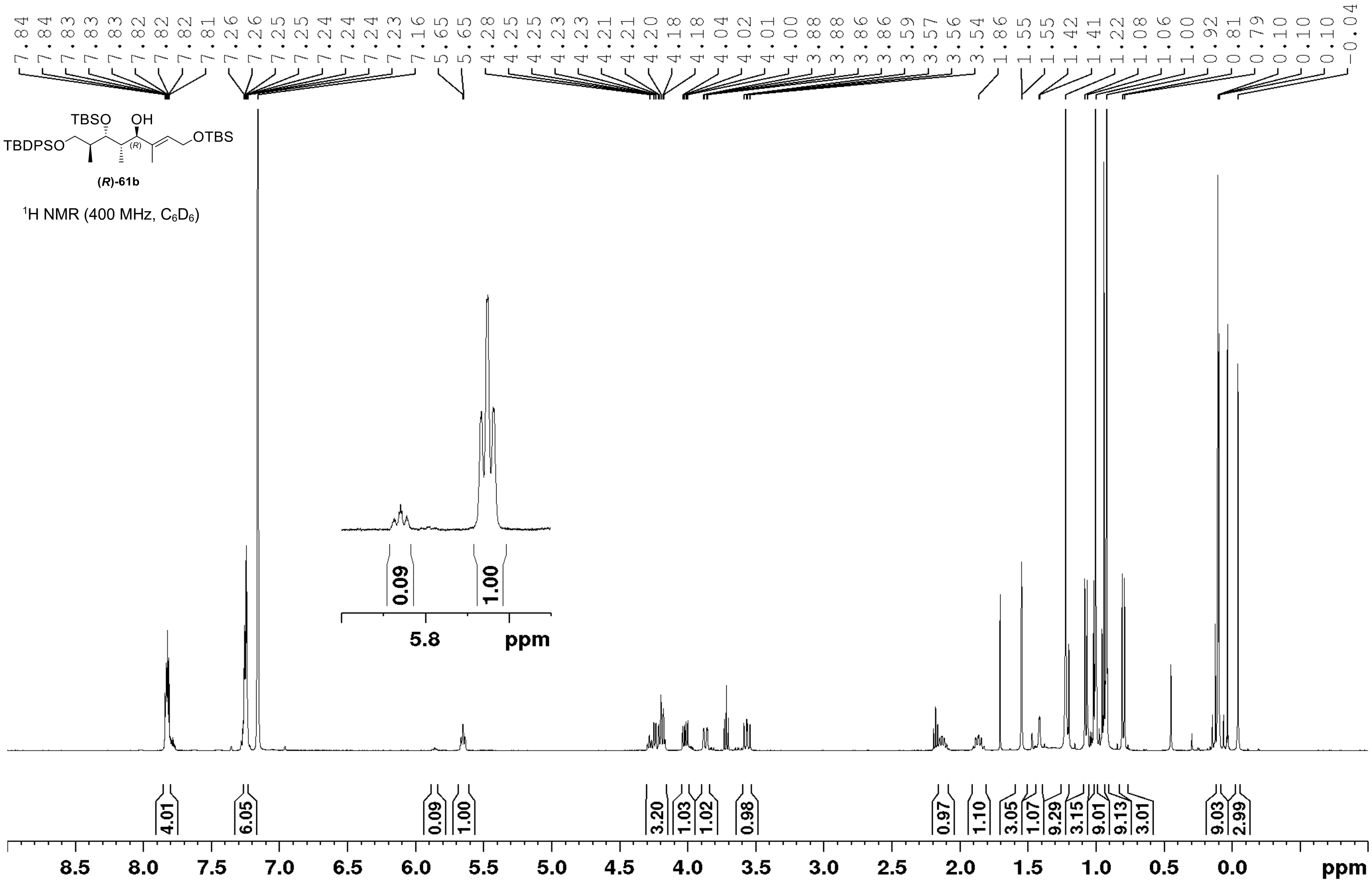
TBSO OH
TBDPSO
OTBS
(R)
(R)-61b
^{1}H NMR (400 MHz, C_6D_6)
ppm

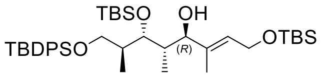

(*R*)-61b

$^{13}C\{^{1}H\}$ NMR (101 MHz, C_6D_6)

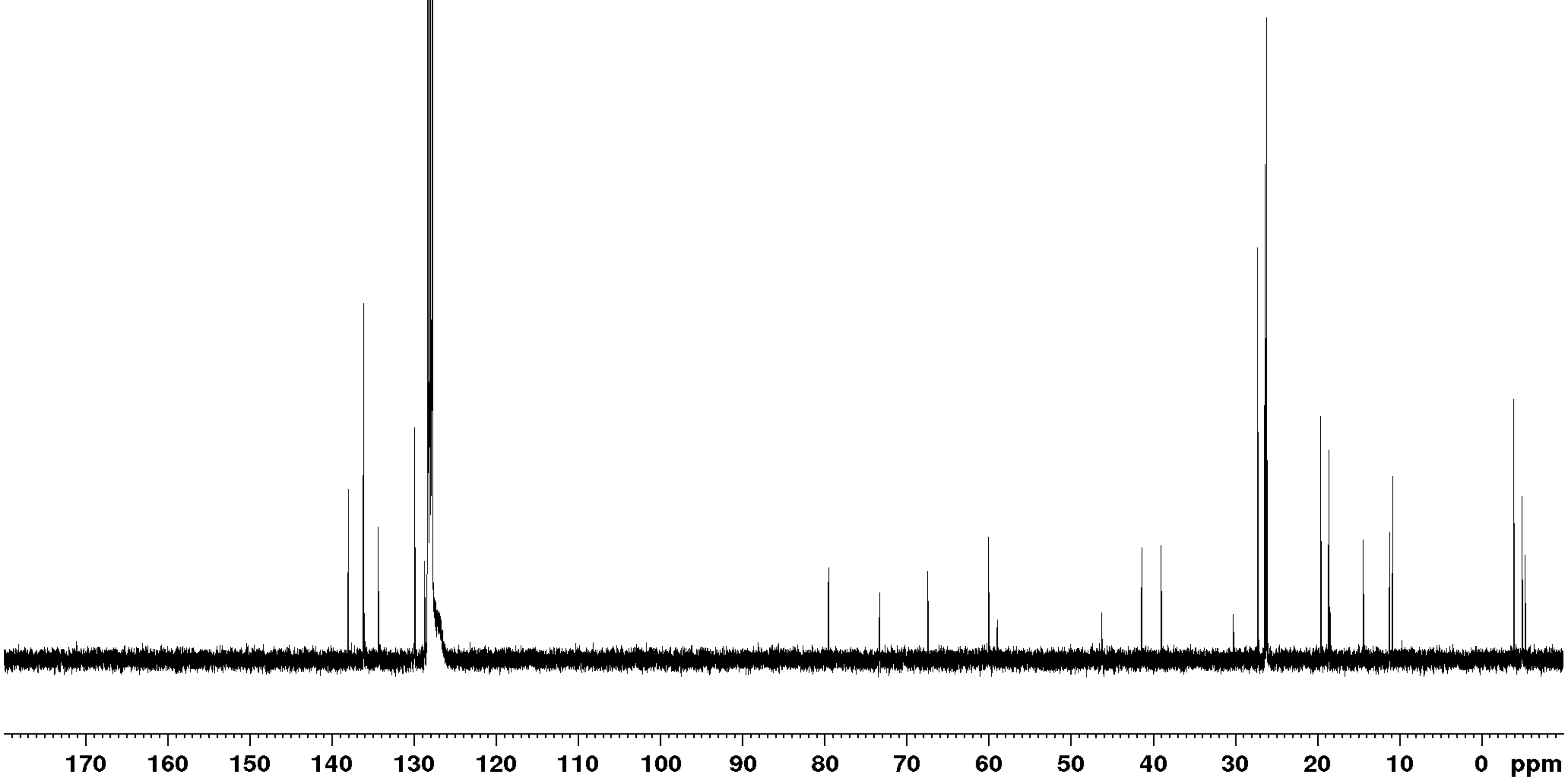

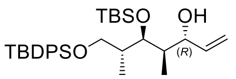

(*R*)-62b

^{1}H NMR (400 MHz, C_6D_6)

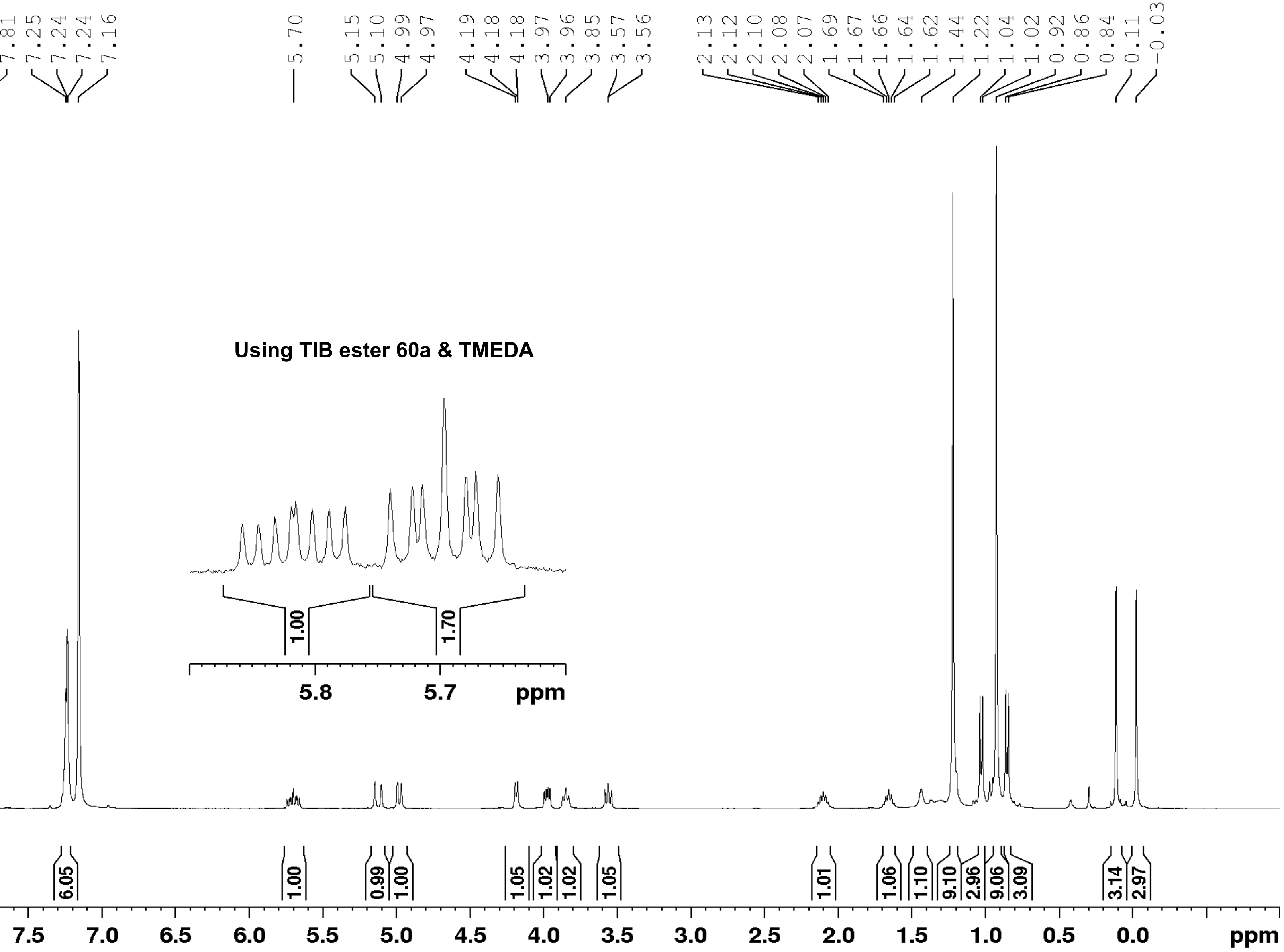

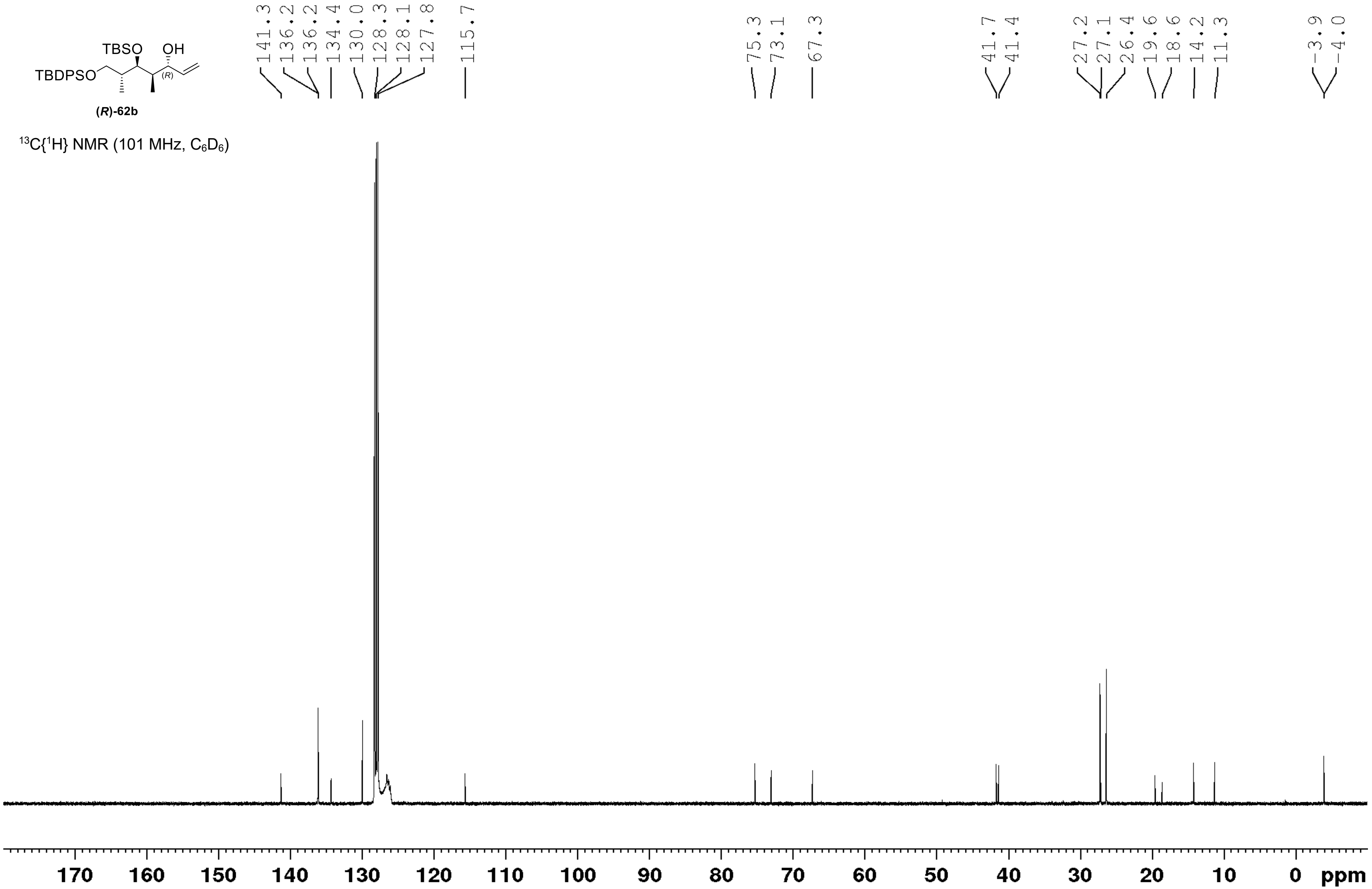
TBSO
OH
TBDPSO
(R)
(R)-62b
$^{13}C\{^1H\}$ NMR (101 MHz, C_6D_6)
141.3
136.2
136.2
134.4
130.0
128.3
128.1
127.8
115.7
75.3
73.1
67.3
41.7
41.4
27.2
27.1
26.4
19.6
18.6
14.2
11.3
-3.9
-4.0
170
160
150
140
130
120
110
100
90
80
70
60
50
40
30
20
10
0
ppm

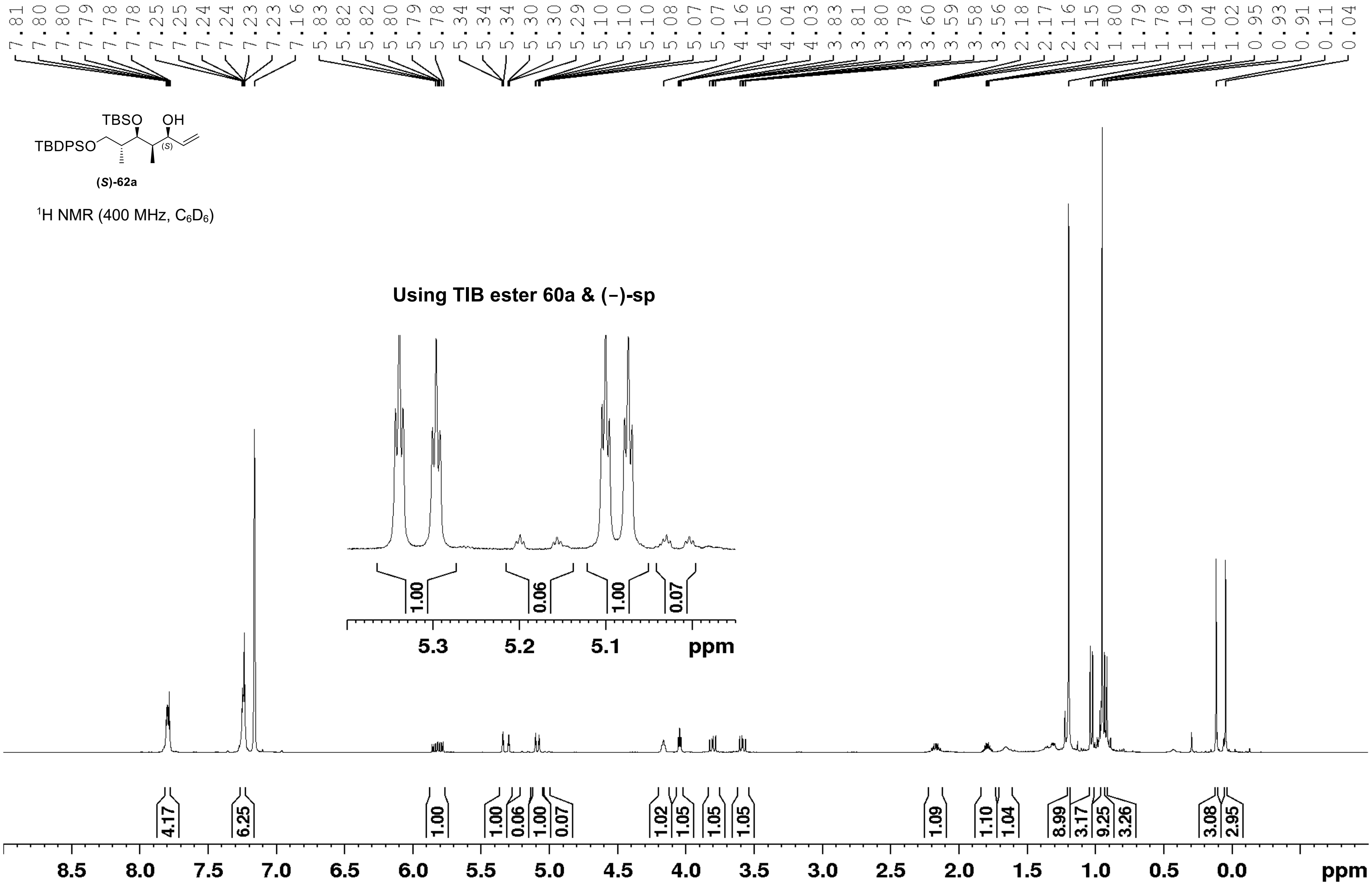

7.81 7.80 7.80 7.79 7.78 7.78 7.25 7.25 7.24 7.24 7.23 7.23 7.16 5.83 5.82 5.82 5.80 5.79 5.78 5.34 5.34 5.34 5.30 5.30 5.29 5.10 5.10 5.10 5.08 5.07 5.07 4.16 4.05 4.04 4.03 3.83 3.81 3.80 3.78 3.60 3.59 3.58 3.56 2.18 2.17 2.16 2.15 1.80 1.79 1.78 1.19 1.04 1.02 0.95 0.93 0.91 0.11 0.04
TBSO OH
TBDPSO
(S)
(S)-62a
^{1}H NMR (400 MHz, C_6D_6)
Using TIB ester 60a & (−)-sp
1.00 0.06 1.00 0.07
5.3 5.2 5.1 ppm
4.17 6.25 1.00 1.00 0.06 1.00 0.07 1.02 1.05 1.05 1.05 1.09 1.10 1.04 8.99 3.17 9.25 3.26 3.08 2.95
8.5 8.0 7.5 7.0 6.5 6.0 5.5 5.0 4.5 4.0 3.5 3.0 2.5 2.0 1.5 1.0 0.5 0.0 ppm

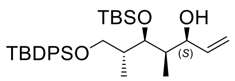

(*S*)-62a

$^{13}C\{^{1}H\}$ NMR (101 MHz, C_6D_6)

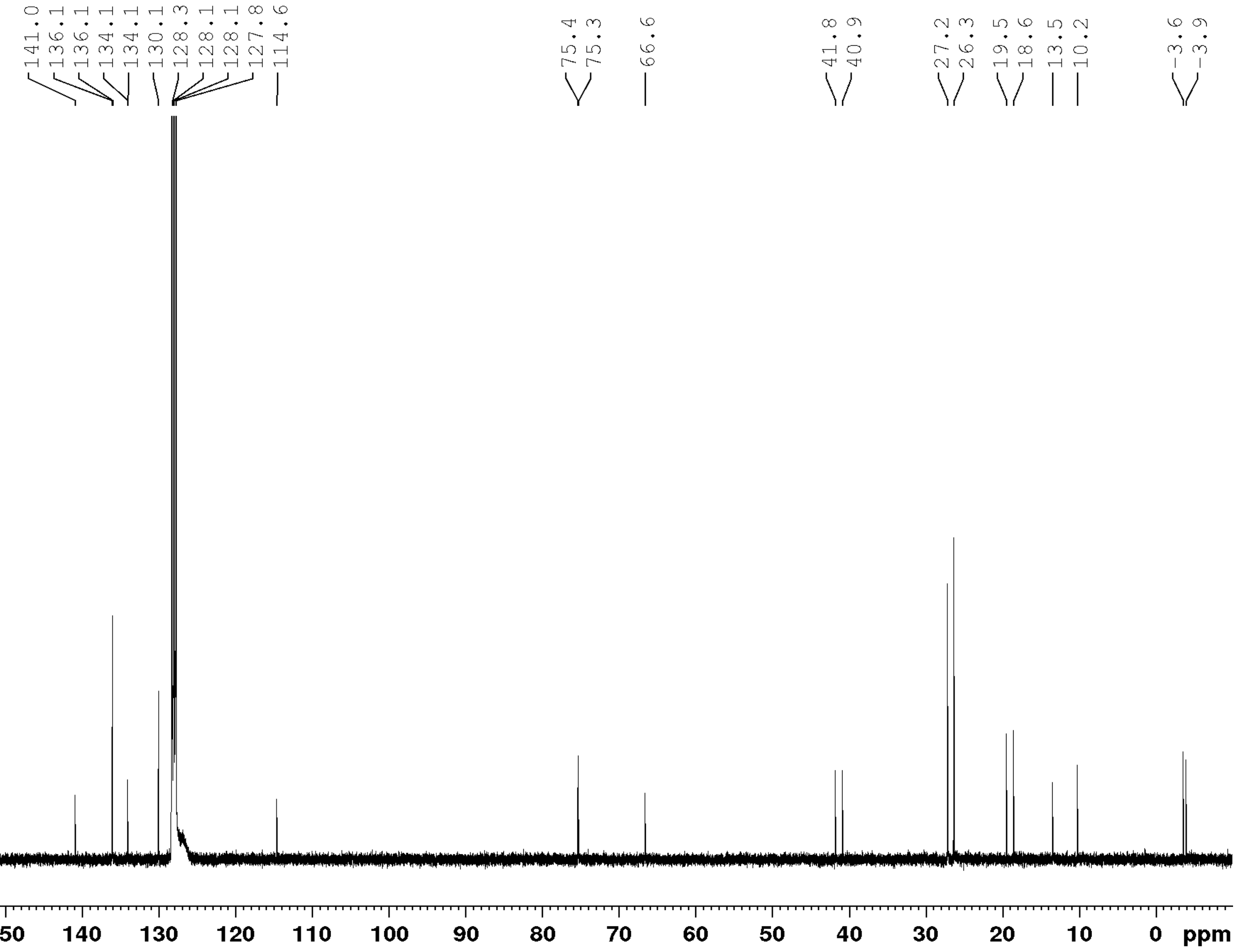

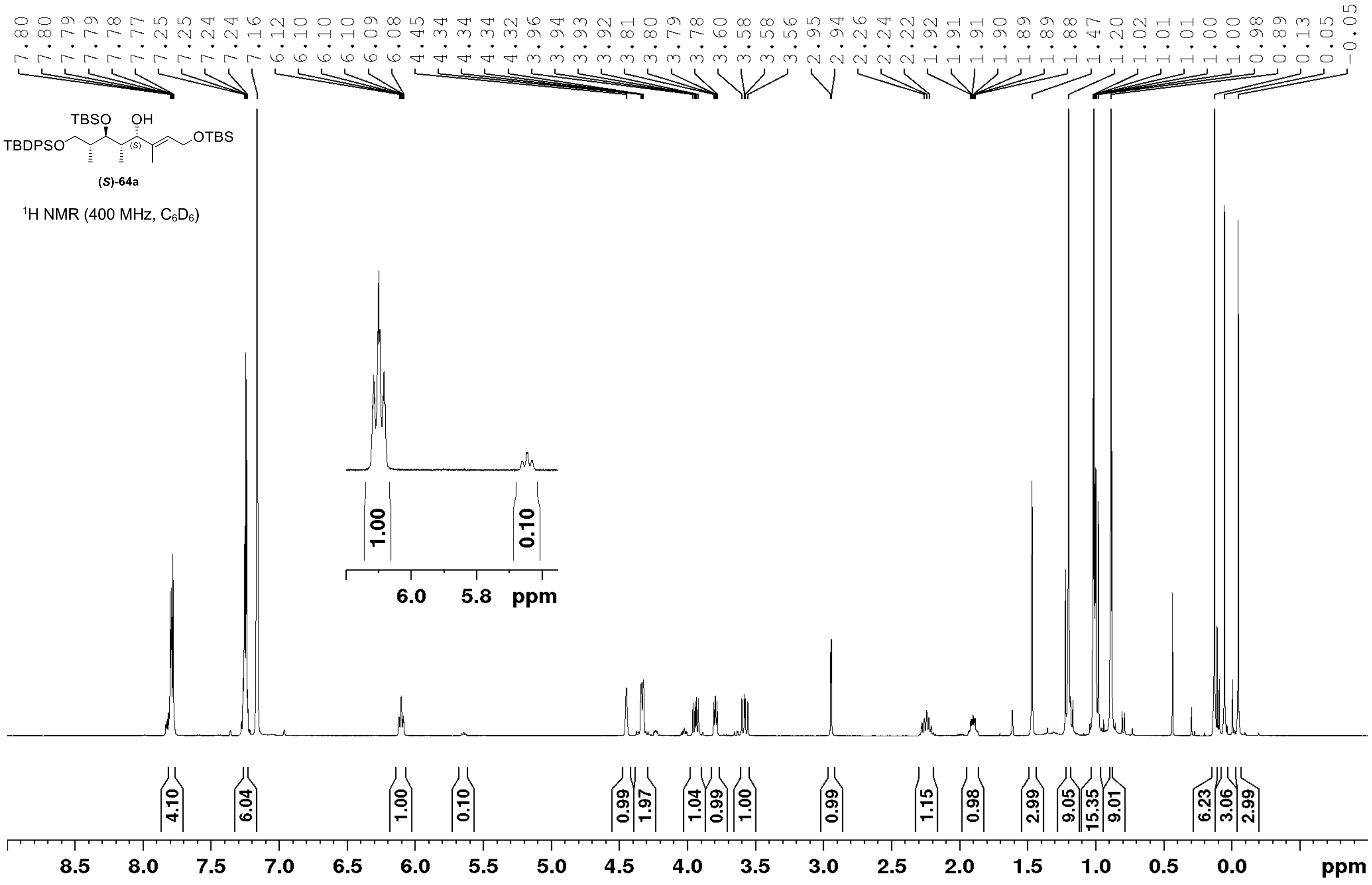
TBSO
OH
TBDPSO
OTBS
(S)
(S)-64a
^{1}H NMR (400 MHz, C_6D_6)
7.80 7.80 7.79 7.79 7.78 7.77 7.25 7.25 7.24 7.24 7.16 6.12 6.10 6.10 6.10 6.09 6.08 4.45 4.34 4.34 4.34 4.32 3.96 3.94 3.93 3.92 3.81 3.80 3.79 3.78 3.60 3.58 3.58 3.56 2.95 2.94 2.26 2.24 2.22 1.92 1.91 1.91 1.90 1.89 1.89 1.88 1.47 1.20 1.02 1.01 1.01 1.00 1.00 0.98 0.89 0.13 0.05 −0.05
1.00
0.10
6.0
5.8
ppm
4.10
6.04
1.00
0.10
0.99
1.97
1.04
0.99
1.00
0.99
1.15
0.98
2.99
9.05
15.35
9.01
6.23
3.06
2.99
8.5 8.0 7.5 7.0 6.5 6.0 5.5 5.0 4.5 4.0 3.5 3.0 2.5 2.0 1.5 1.0 0.5 0.0 ppm

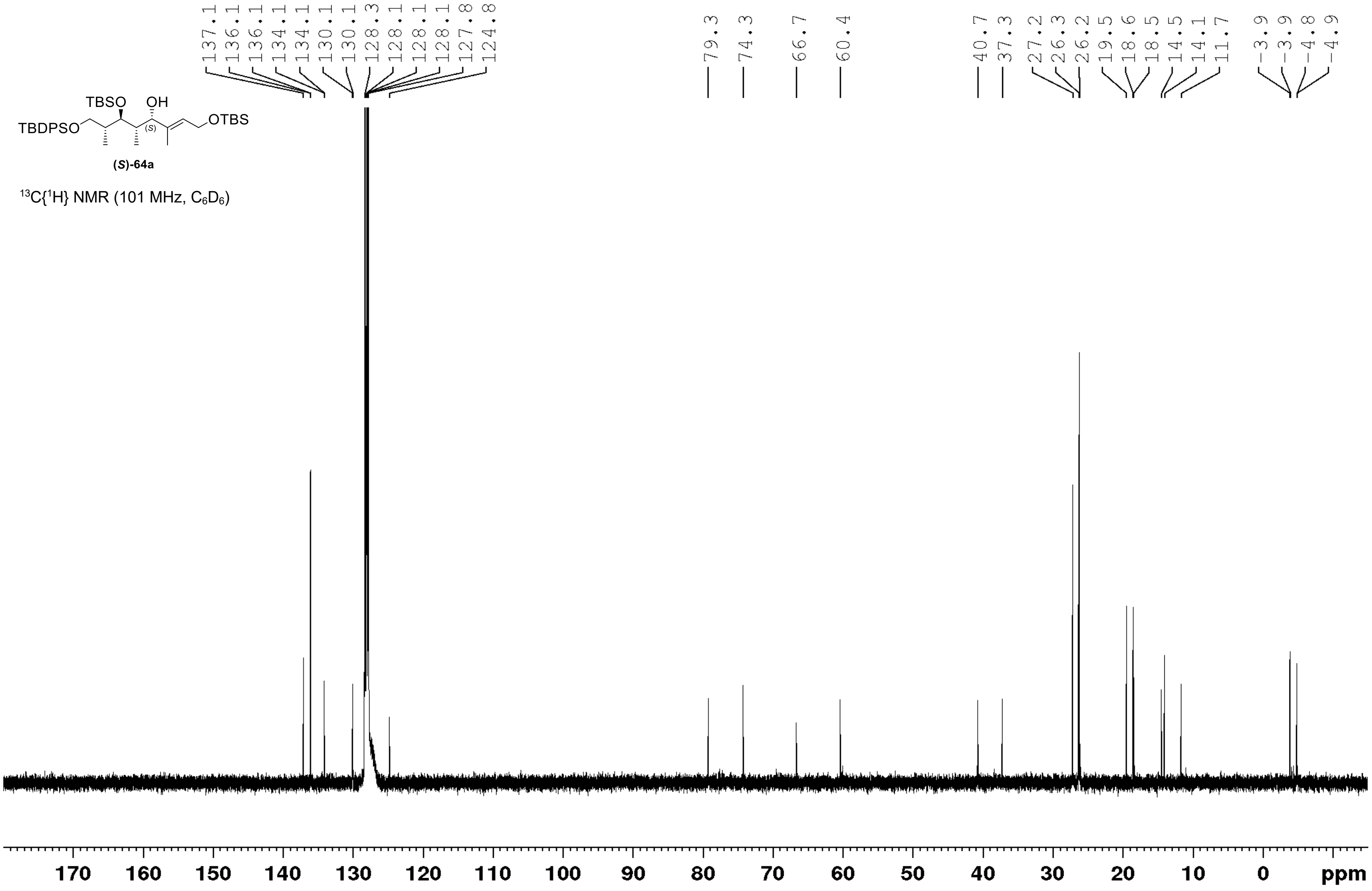
137.1
136.1
136.1
134.1
134.1
130.1
130.1
128.3
128.1
128.1
128.1
127.8
124.8
79.3
74.3
66.7
60.4
40.7
37.3
27.2
26.3
26.2
19.5
18.6
18.5
14.5
14.1
11.7
-3.9
-3.9
-4.8
-4.9
TBSO
OH
TBDPSO
(S)
OTBS
(S)-64a
$^{13}C\{^{1}H\}$ NMR (101 MHz, C_6D_6)
170
160
150
140
130
120
110
100
90
80
70
60
50
40
30
20
10
0
ppm

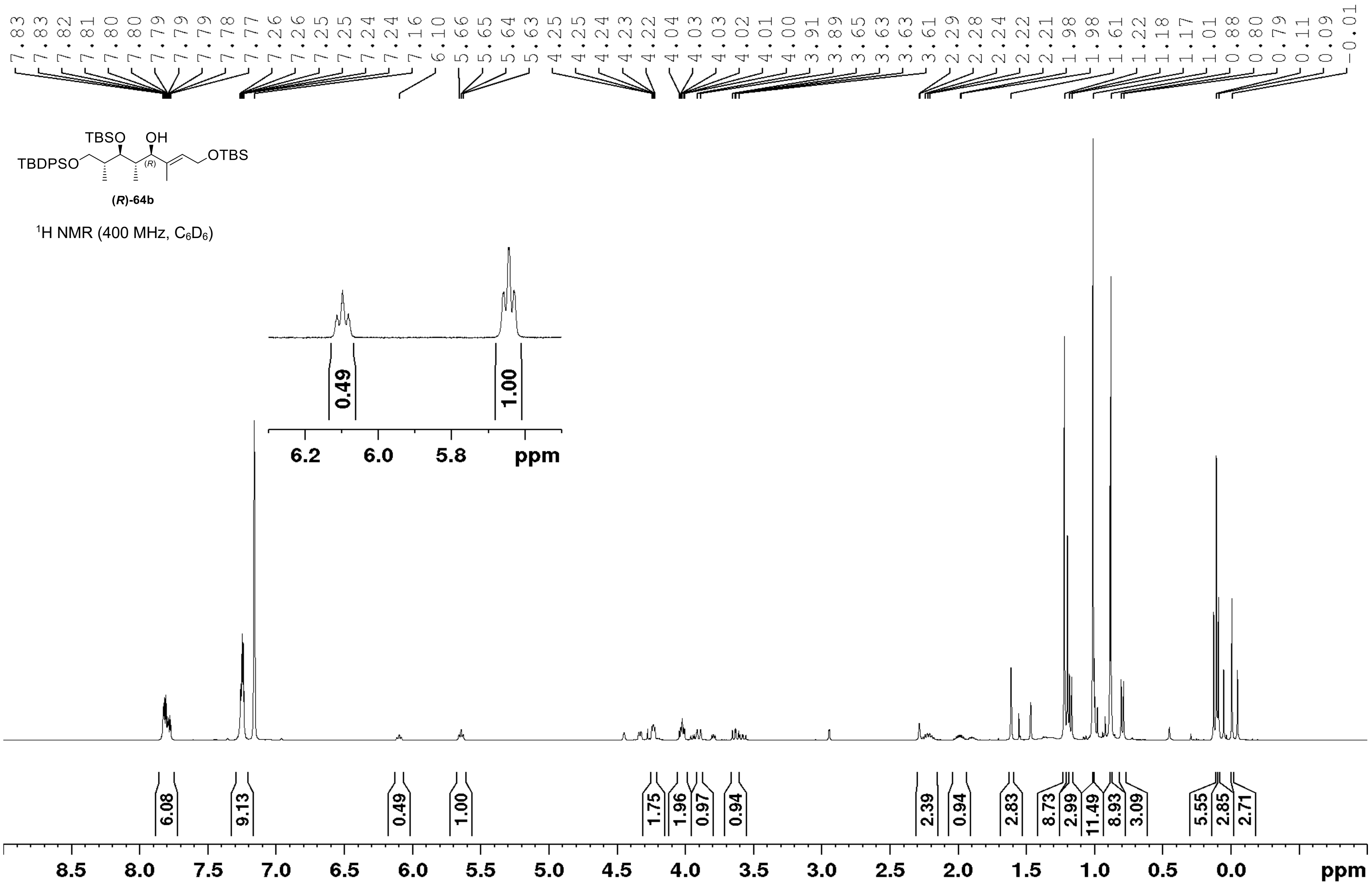
TBSO OH
TBDPSO OTBS
(R)
(R)-64b
¹H NMR (400 MHz, C6D6)
ppm

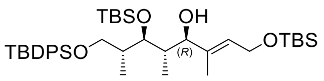

(*R*)-64b

$^{13}C\{^1H\}$ NMR (101 MHz, C_6D_6)

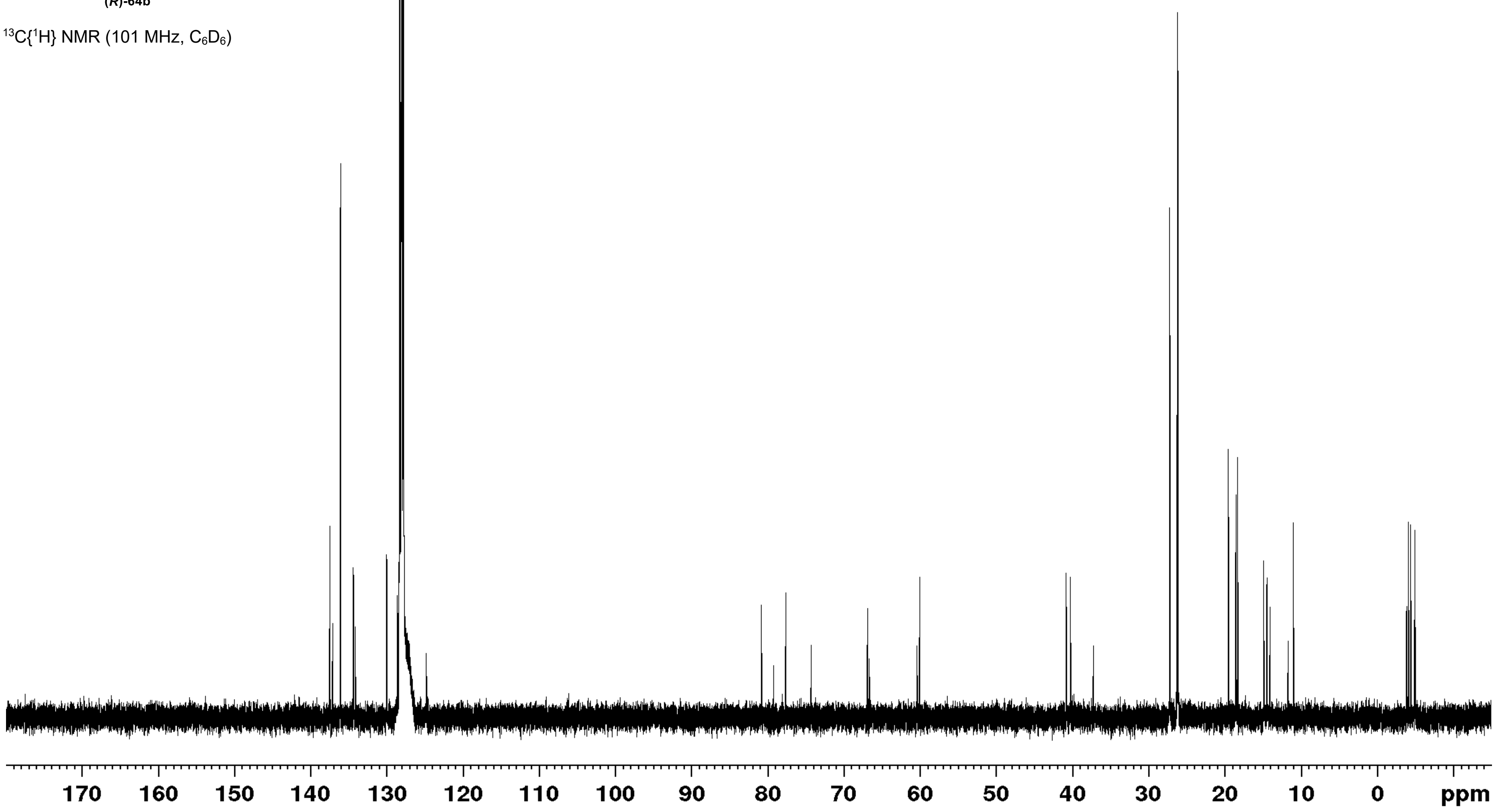

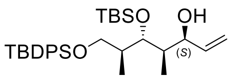

(*S*)-65a

^{1}H NMR (400 MHz, C_6D_6)

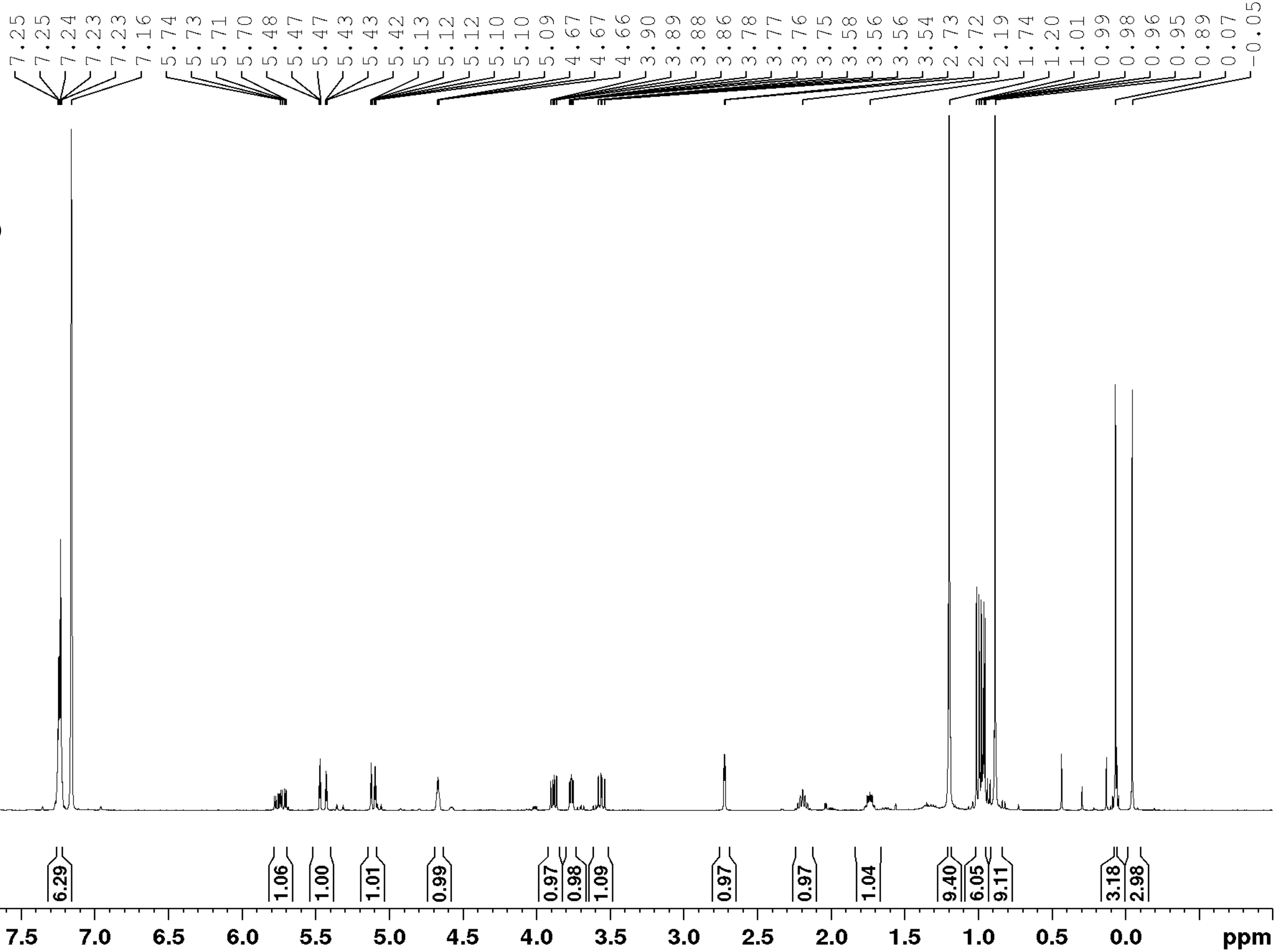

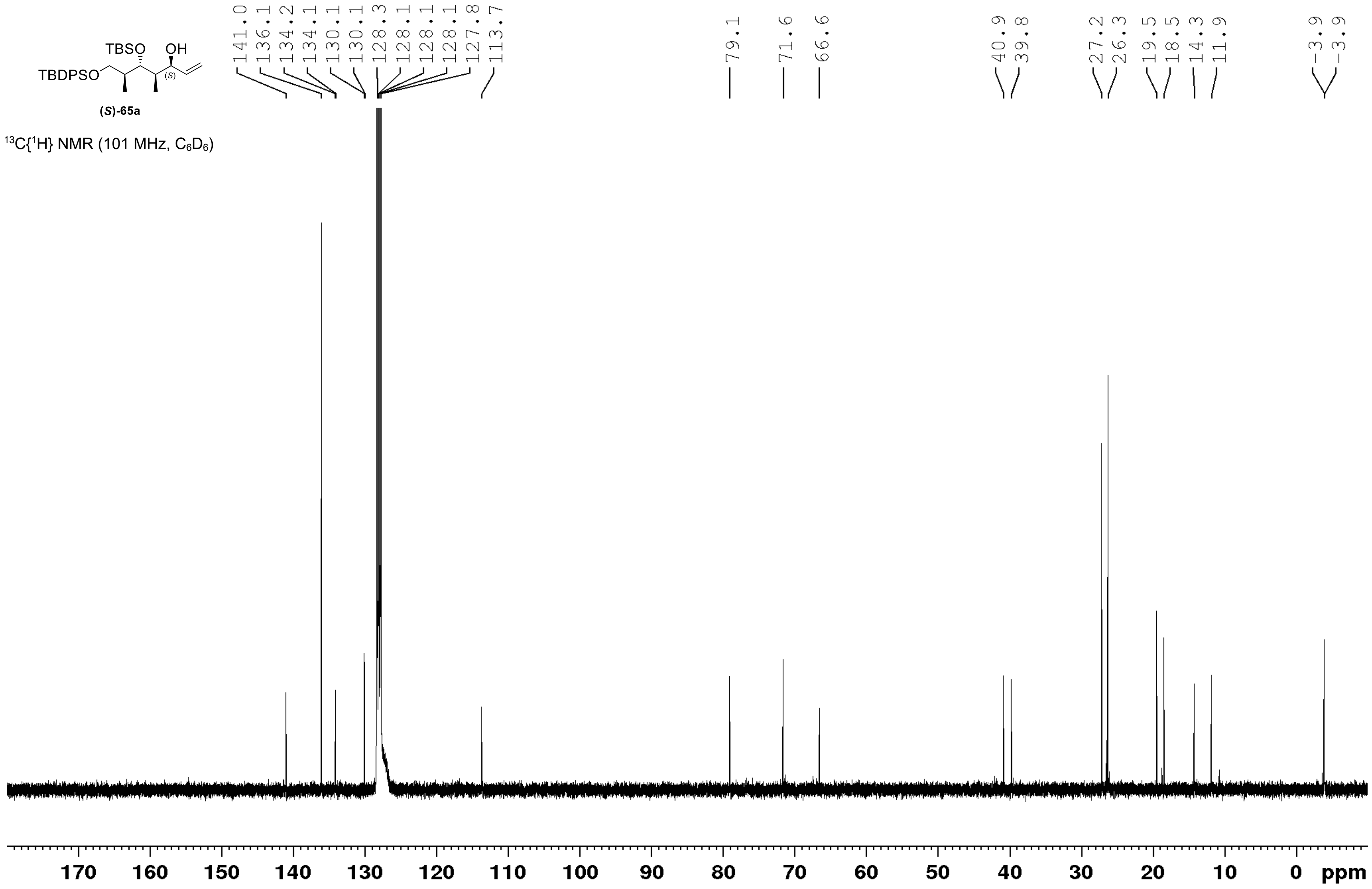
TBSO OH
TBDPSO
(S)
(S)-65a
$^{13}C\{^{1}H\}$ NMR (101 MHz, C_6D_6)
141.0
136.1
134.2
134.1
130.1
130.1
128.3
128.1
128.1
128.1
127.8
113.7
79.1
71.6
66.6
40.9
39.8
27.2
26.3
19.5
18.5
14.3
11.9
-3.9
-3.9
170
160
150
140
130
120
110
100
90
80
70
60
50
40
30
20
10
0
ppm

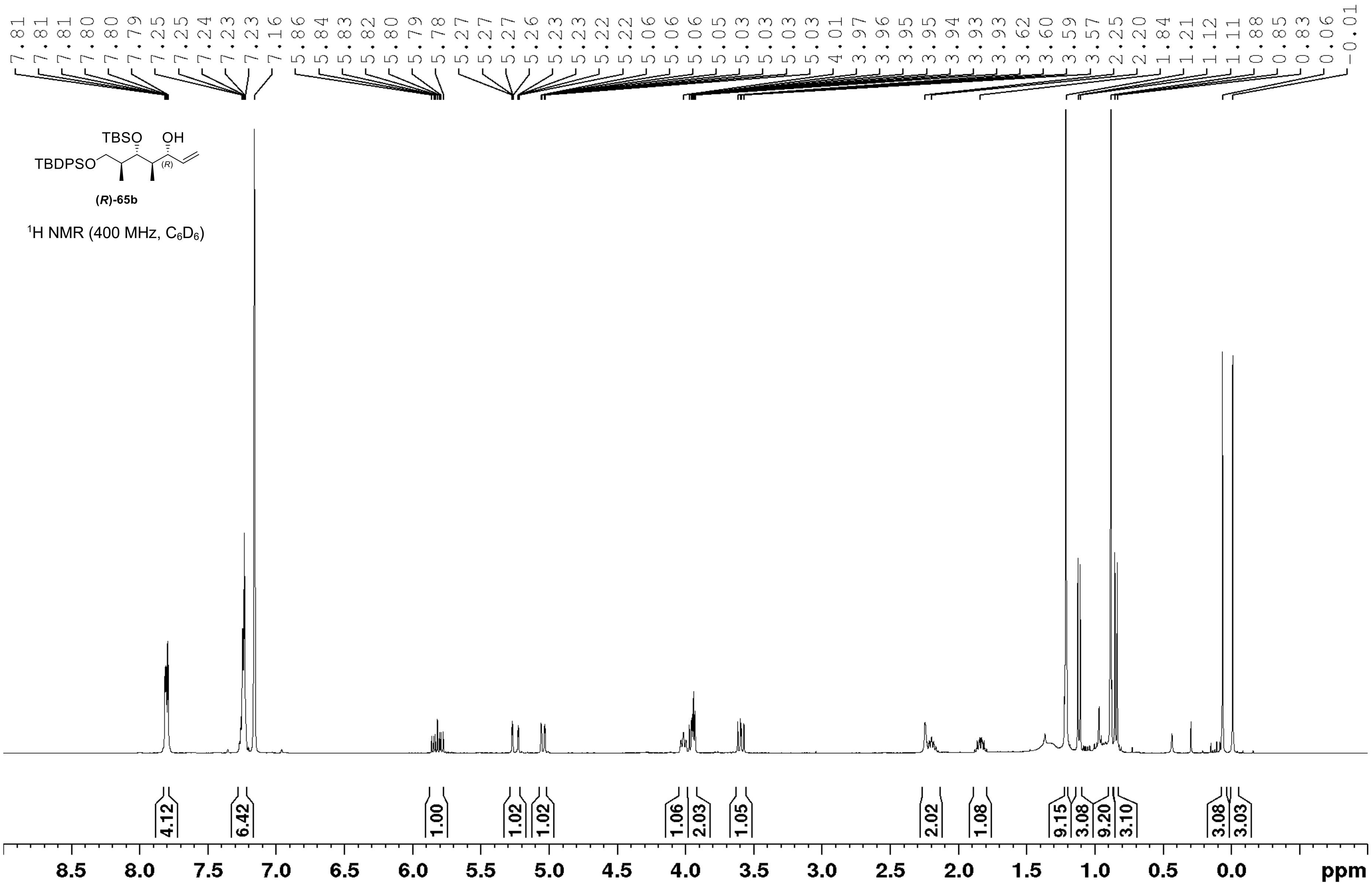
7.81 7.81 7.81 7.80 7.80 7.79 7.25 7.25 7.24 7.23 7.23 7.16 5.86 5.84 5.83 5.82 5.80 5.79 5.78 5.27 5.27 5.27 5.26 5.23 5.23 5.22 5.22 5.06 5.06 5.06 5.05 5.03 5.03 5.03 5.03 5.01 4.01 3.97 3.96 3.95 3.95 3.94 3.93 3.93 3.62 3.60 3.59 3.57 2.25 2.20 1.84 1.21 1.12 1.11 0.88 0.85 0.83 0.06 −0.01
TBSO
OH
TBDPSO
(R)
(R)-65b
1H NMR (400 MHz, C6D6)
4.12
6.42
1.00
1.02
1.02
1.06
2.03
1.05
2.02
1.08
9.15
3.08
9.20
3.10
3.08
3.03
8.5 8.0 7.5 7.0 6.5 6.0 5.5 5.0 4.5 4.0 3.5 3.0 2.5 2.0 1.5 1.0 0.5 0.0 ppm

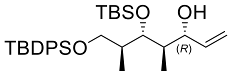

(*R*)-65b

$^{13}C\{^{1}H\}$ NMR (101 MHz, C_6D_6)

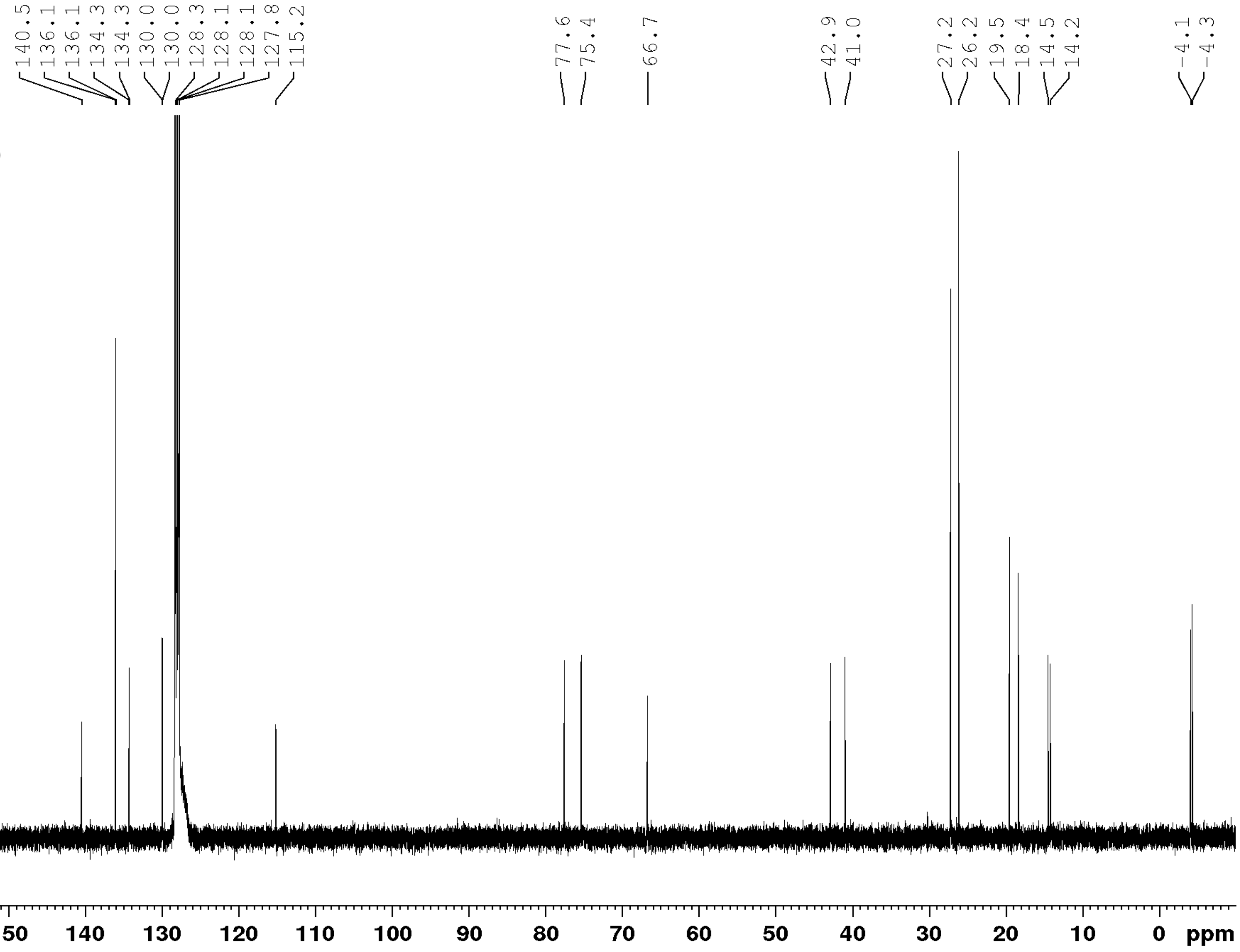

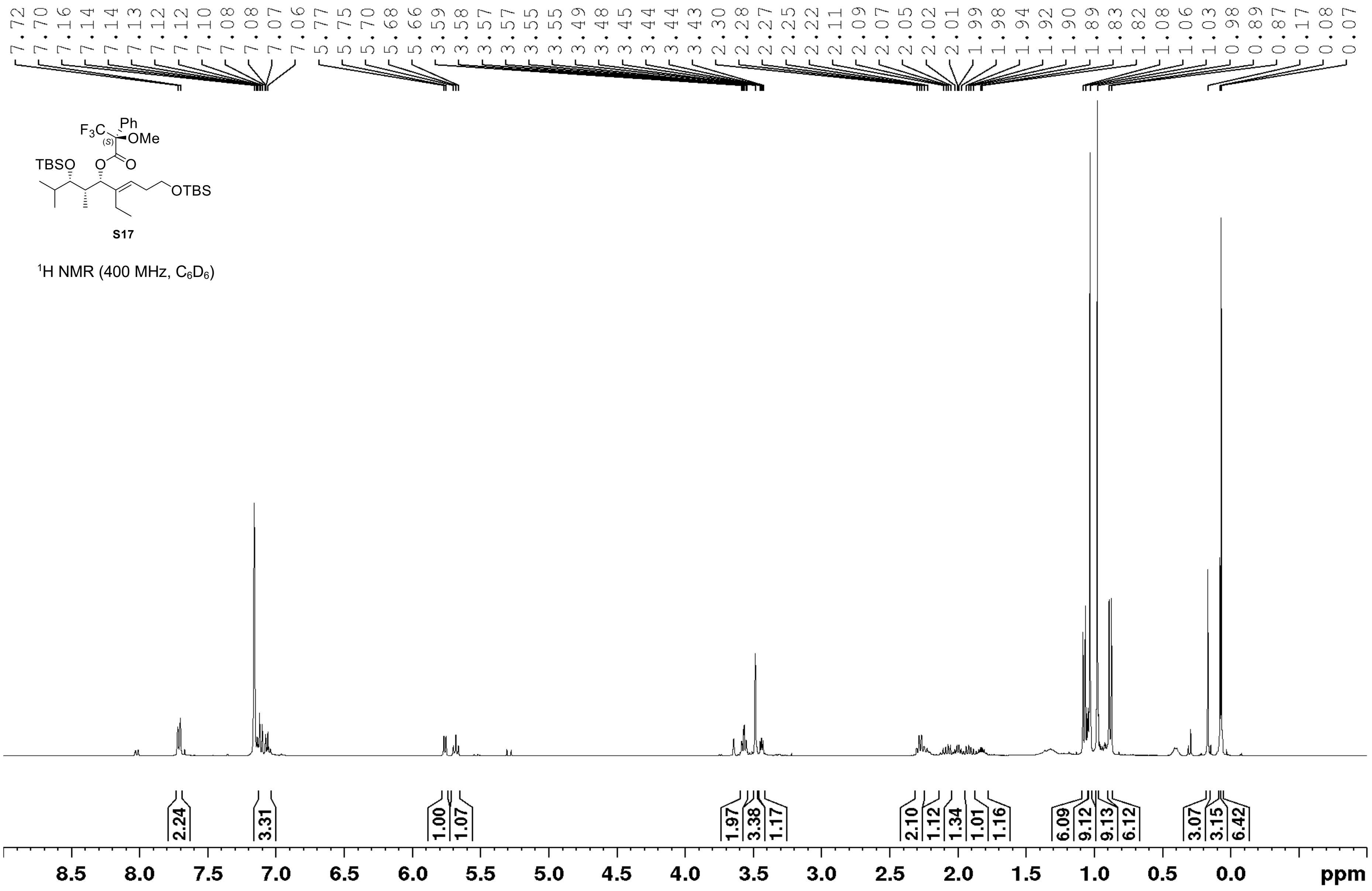

S17

^{1}H NMR (400 MHz, C_6D_6)

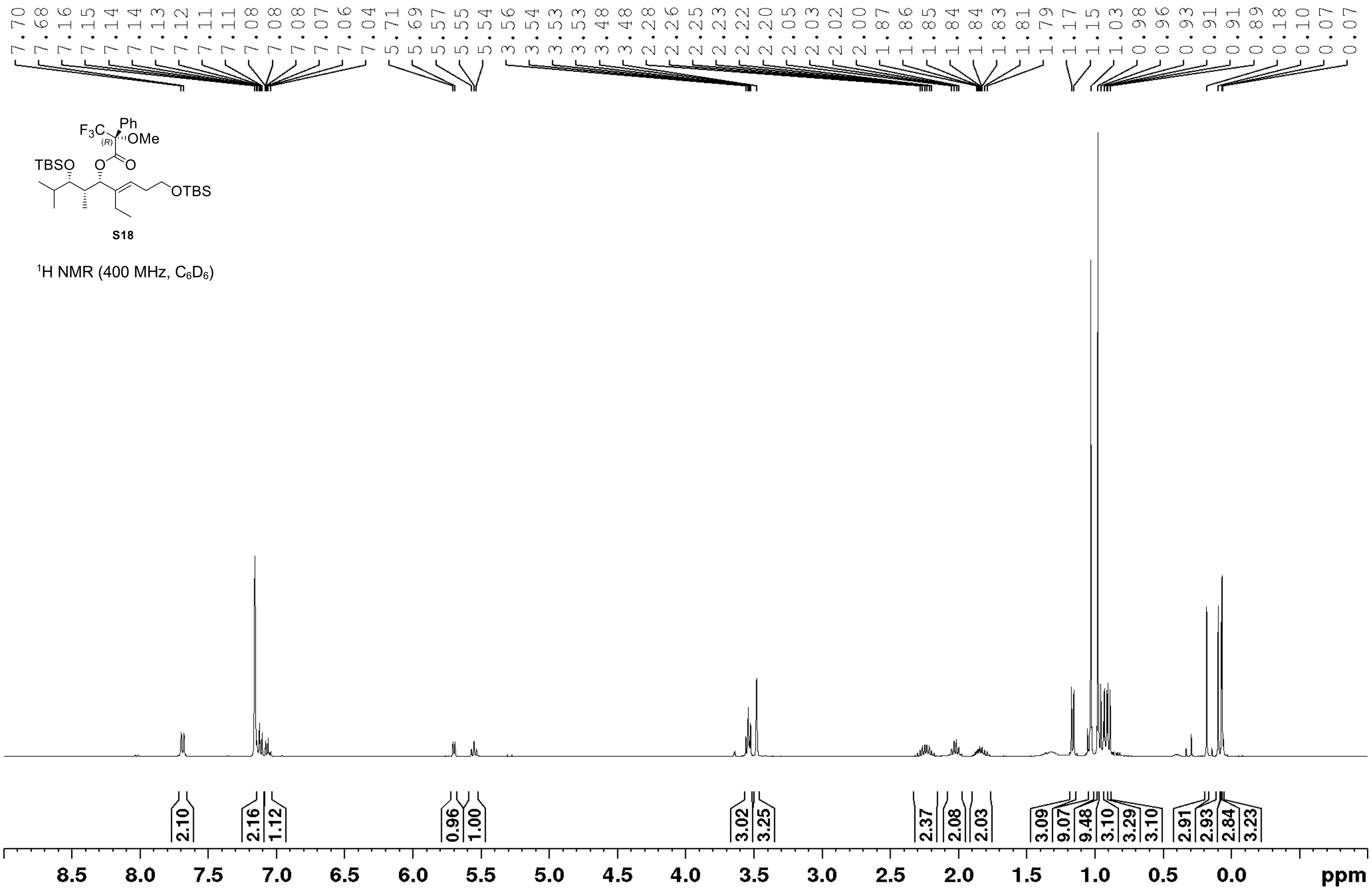
7.70 7.68 7.16 7.15 7.14 7.14 7.13 7.12 7.11 7.11 7.08 7.08 7.08 7.07 7.06 7.04 5.71 5.69 5.57 5.55 5.54 3.56 3.54 3.53 3.53 3.48 2.28 2.26 2.25 2.23 2.22 2.20 2.05 2.03 2.02 2.00 1.87 1.86 1.85 1.84 1.84 1.83 1.81 1.79 1.17 1.15 1.03 0.98 0.96 0.93 0.91 0.91 0.89 0.18 0.10 0.07 0.07
F3C
Ph
(R)
OMe
TBSO
O
O
OTBS
S18
1H NMR (400 MHz, C6D6)
2.10
2.16
1.12
0.96
1.00
3.02
3.25
2.37
2.08
2.03
3.09
9.07
9.48
3.10
3.29
3.10
2.91
2.93
2.84
3.23
8.5 8.0 7.5 7.0 6.5 6.0 5.5 5.0 4.5 4.0 3.5 3.0 2.5 2.0 1.5 1.0 0.5 0.0 ppm

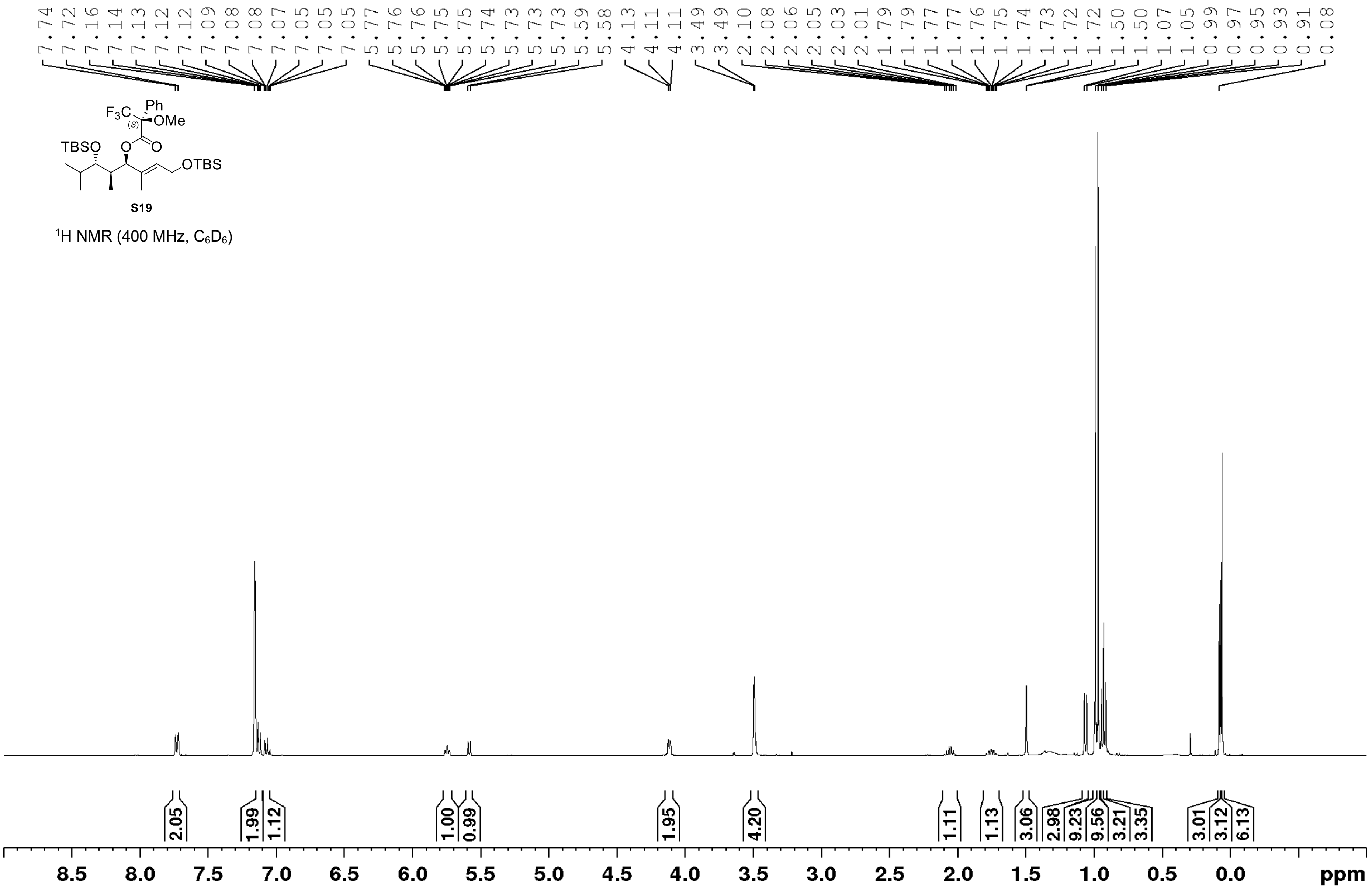

7.74 7.72 7.16 7.14 7.13 7.12 7.12 7.09 7.08 7.08 7.07 7.05 7.05 7.05 5.77 5.76 5.76 5.75 5.75 5.74 5.73 5.73 5.73 5.73 5.59 5.58 4.13 4.11 4.11 3.49 3.49 2.10 2.08 2.06 2.05 2.03 2.01 1.79 1.79 1.77 1.77 1.76 1.75 1.74 1.73 1.72 1.72 1.50 1.50 1.07 1.05 0.99 0.97 0.95 0.93 0.91 0.08
F3C
Ph
(S)
OMe
TBSO
O
O
OTBS
S19
1H NMR (400 MHz, C6D6)
2.05 1.99 1.12 1.00 0.99 1.95 4.20 1.11 1.13 3.06 2.98 9.23 9.56 3.21 3.35 3.01 3.12 6.13
8.5 8.0 7.5 7.0 6.5 6.0 5.5 5.0 4.5 4.0 3.5 3.0 2.5 2.0 1.5 1.0 0.5 0.0 ppm

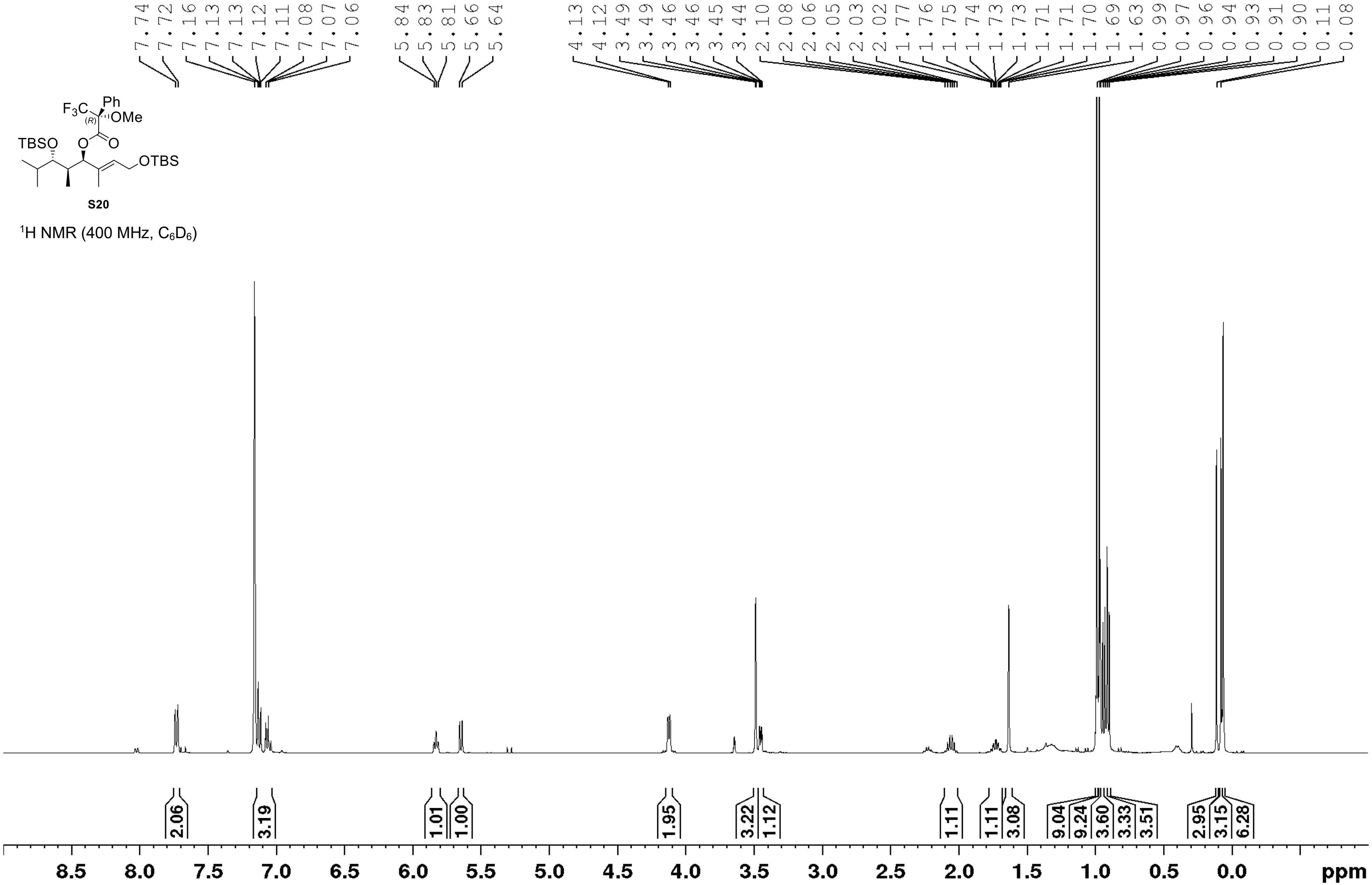
7.74 7.72 7.16 7.13 7.13 7.12 7.11 7.08 7.07 7.06
5.84 5.83 5.81 5.66 5.64
4.13 4.12 3.49 3.49 3.46 3.46 3.45 3.44 2.10 2.08 2.06 2.05 2.03 2.02 1.77 1.76 1.75 1.74 1.73 1.73 1.71 1.71 1.70 1.69 1.63 0.99 0.97 0.96 0.94 0.93 0.91 0.90 0.11 0.08
F3C
Ph
(R)
OMe
TBSO
O
O
OTBS
S20
1H NMR (400 MHz, C6D6)
2.06
3.19
1.01
1.00
1.95
3.22
1.12
1.11
1.11
3.08
9.04
9.24
3.60
3.33
3.51
2.95
3.15
6.28
8.5 8.0 7.5 7.0 6.5 6.0 5.5 5.0 4.5 4.0 3.5 3.0 2.5 2.0 1.5 1.0 0.5 0.0
ppm

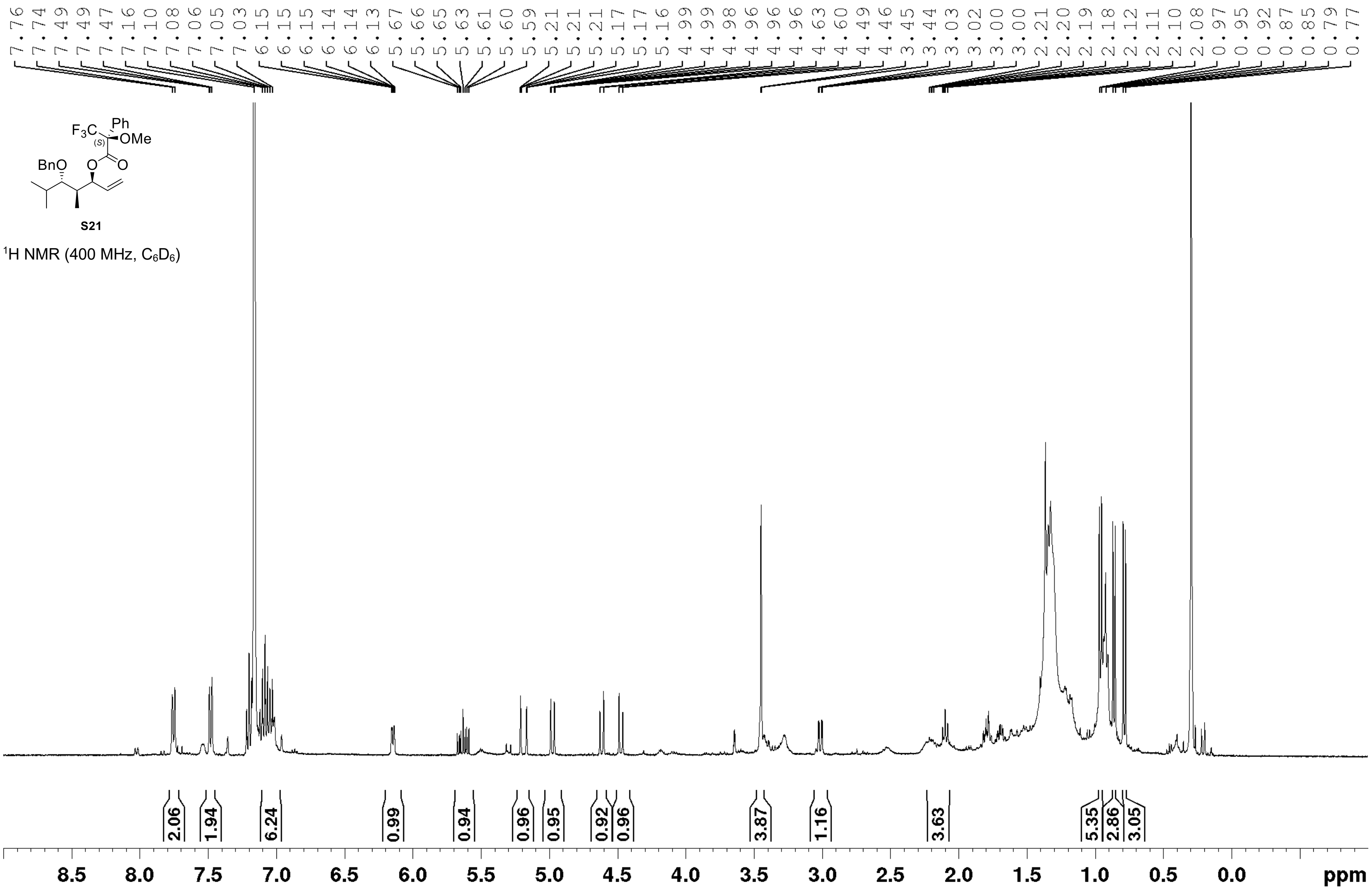

^{1}H NMR (400 MHz, C_6D_6)

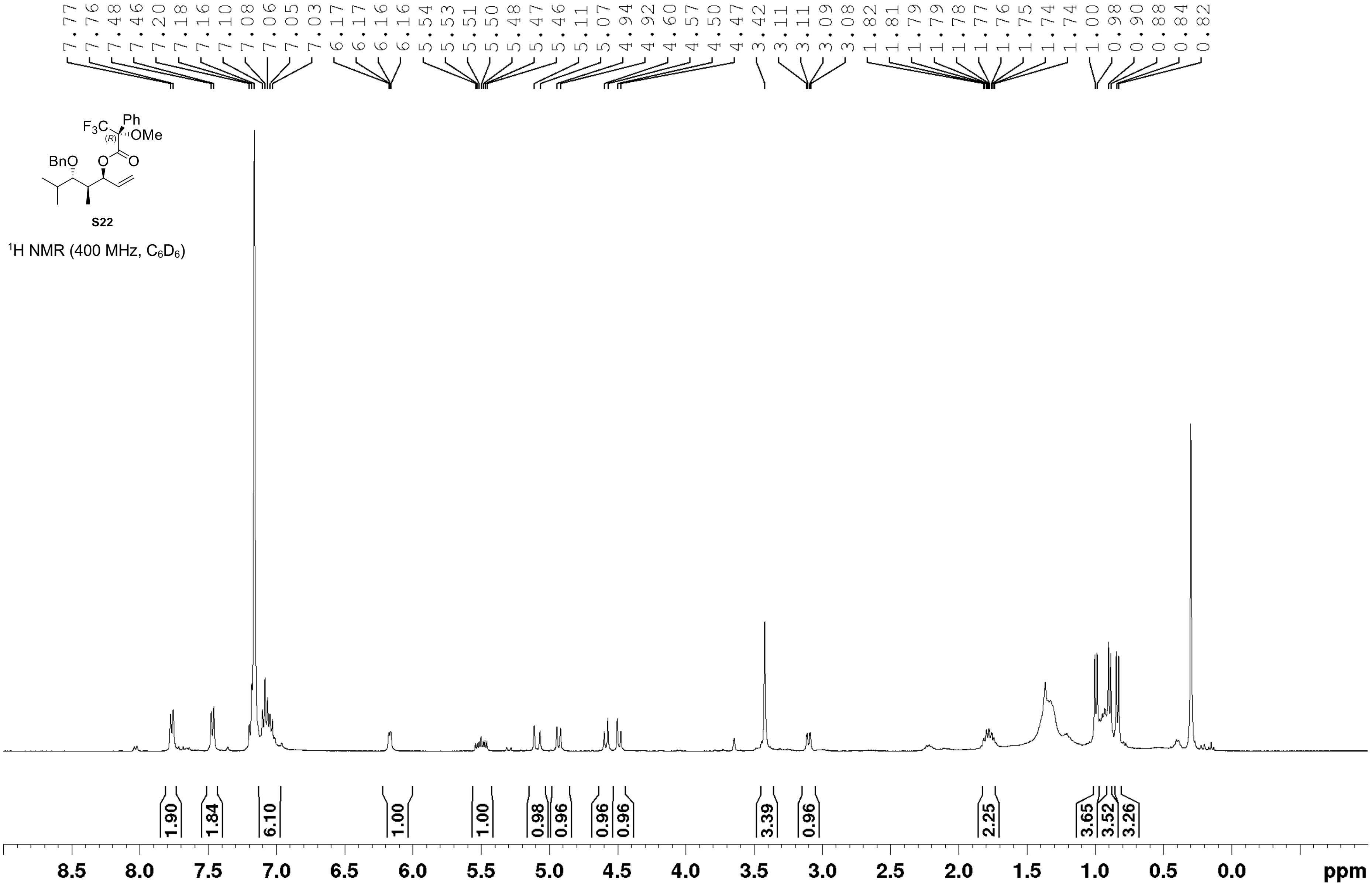

S22

^1H NMR (400 MHz, C_6D_6)

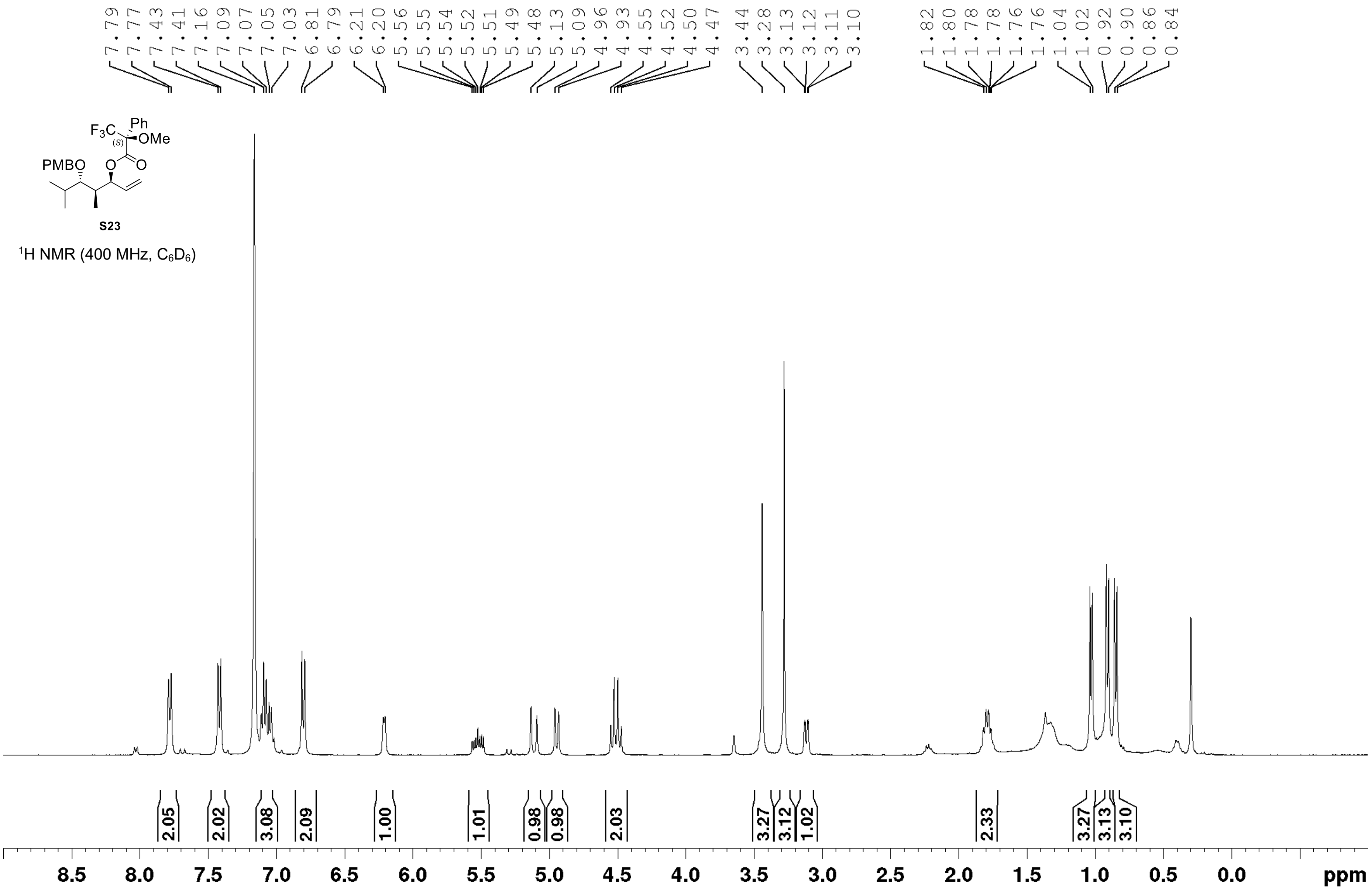
PMBO
F3C
Ph
OMe
(S)
O
O
S23
1H NMR (400 MHz, C6D6)
7.79 7.77 7.43 7.41 7.16 7.09 7.07 7.05 7.03 6.81 6.79 6.21 6.20 5.56 5.55 5.54 5.52 5.51 5.49 5.48 5.13 5.09 4.96 4.93 4.55 4.52 4.50 4.47 3.44 3.28 3.13 3.12 3.11 3.10 1.82 1.80 1.78 1.78 1.76 1.76 1.04 1.02 0.92 0.90 0.86 0.84
2.05 2.02 3.08 2.09 1.00 1.01 0.98 0.98 2.03 3.27 3.12 1.02 2.33 3.27 3.13 3.10
8.5 8.0 7.5 7.0 6.5 6.0 5.5 5.0 4.5 4.0 3.5 3.0 2.5 2.0 1.5 1.0 0.5 0.0 ppm

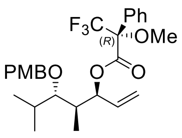

S24

^1H NMR (400 MHz, C_6D_6)

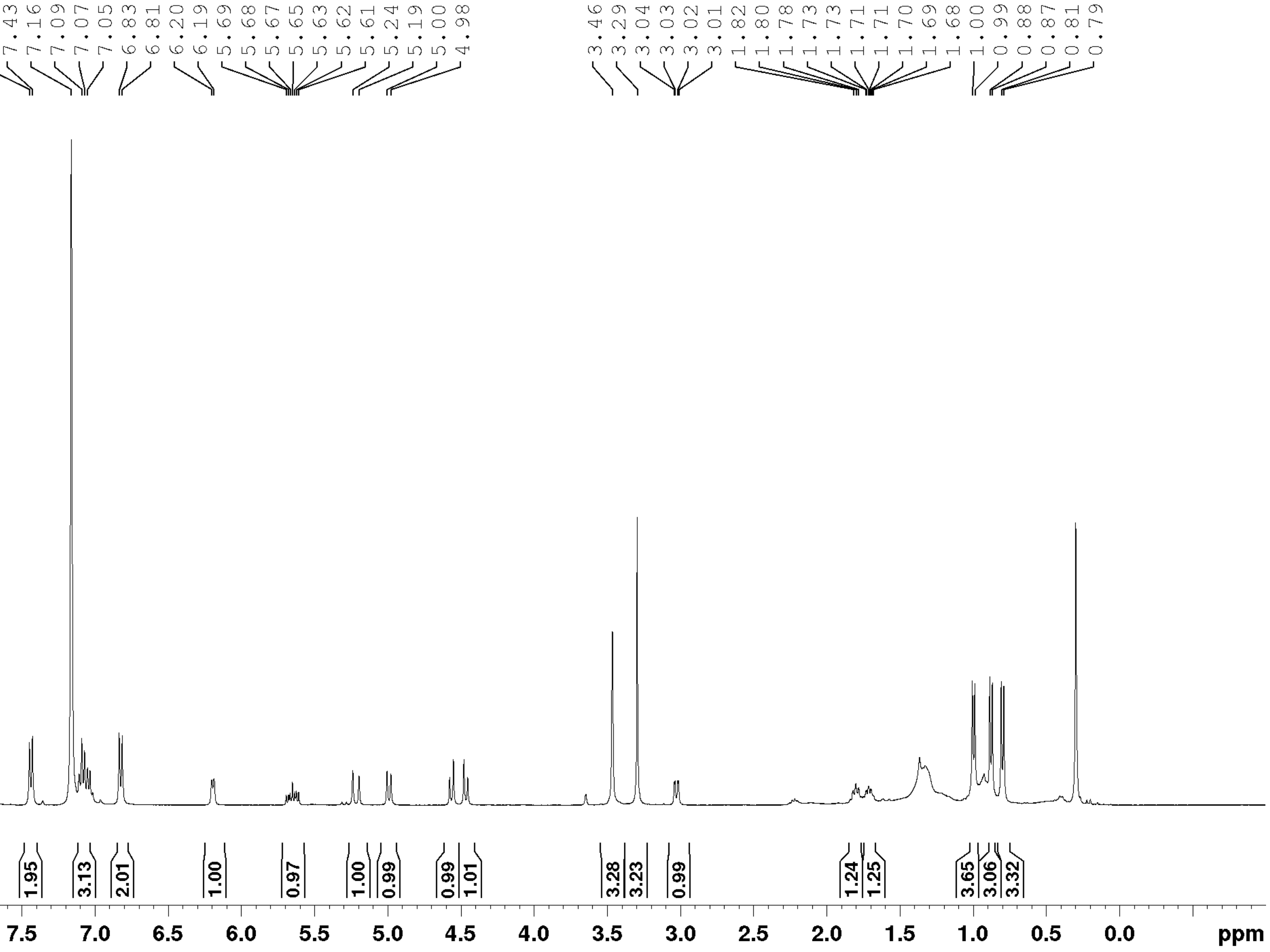

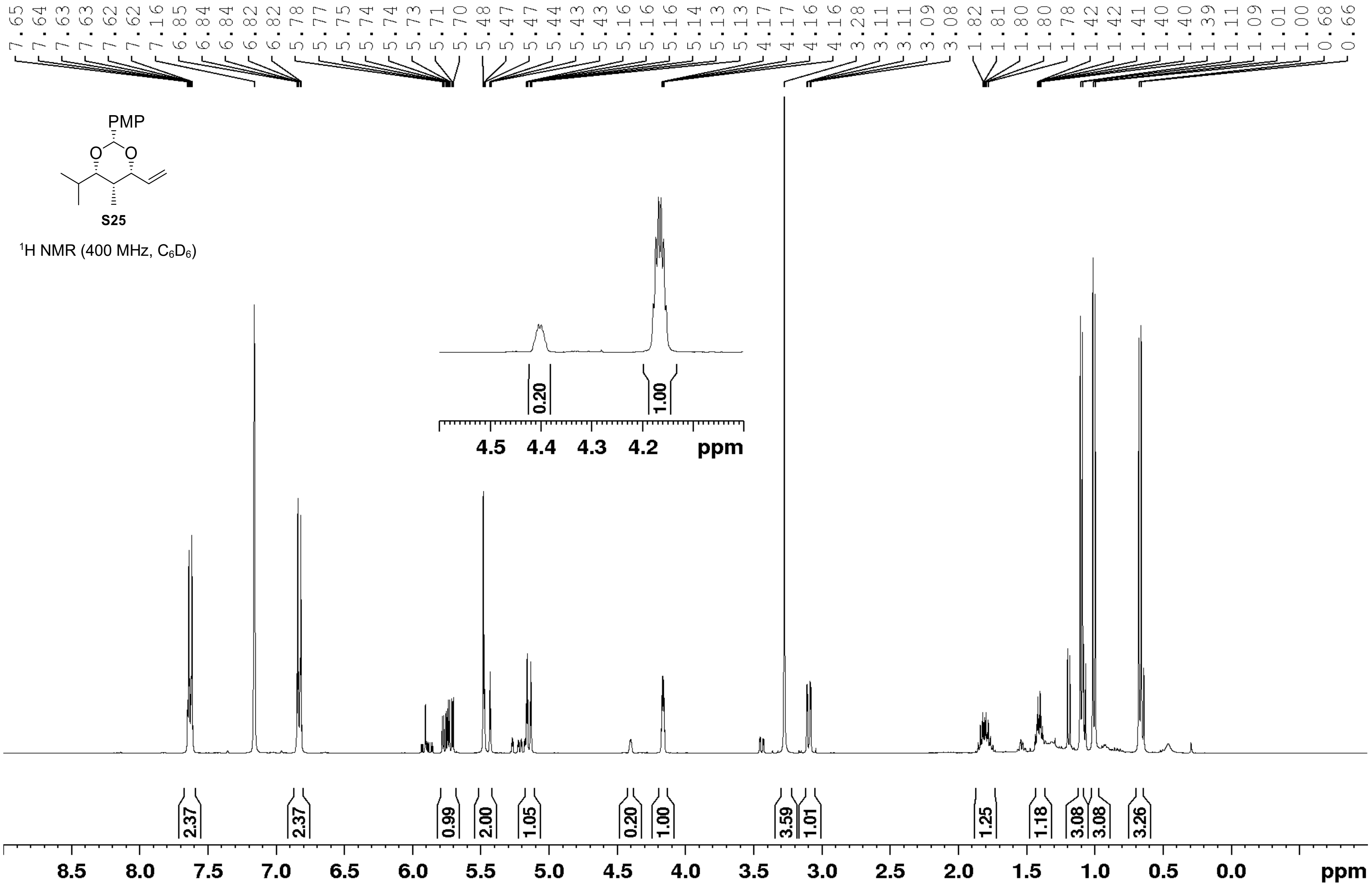
PMP
O
O
S25
^{1}H NMR (400 MHz, C_6D_6)
7.65 7.64 7.63 7.63 7.62 7.62 7.16 6.85 6.84 6.84 6.82 6.82 5.78 5.77 5.75 5.74 5.74 5.73 5.71 5.70 5.48 5.47 5.47 5.44 5.43 5.43 5.16 5.16 5.16 5.14 5.13 5.13 4.17 4.17 4.16 4.16 3.28 3.11 3.11 3.09 3.08 1.82 1.81 1.80 1.80 1.78 1.42 1.42 1.41 1.40 1.40 1.39 1.11 1.09 1.01 1.00 0.68 0.66
0.20
1.00
4.5 4.4 4.3 4.2 ppm
2.37 2.37 0.99 2.00 1.05 0.20 1.00 3.59 1.01 1.25 1.18 3.08 3.08 3.26
8.5 8.0 7.5 7.0 6.5 6.0 5.5 5.0 4.5 4.0 3.5 3.0 2.5 2.0 1.5 1.0 0.5 0.0 ppm

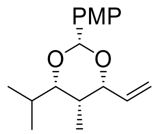

S25

$^{13}C\{^1H\}$ NMR (101 MHz, C_6D_6)

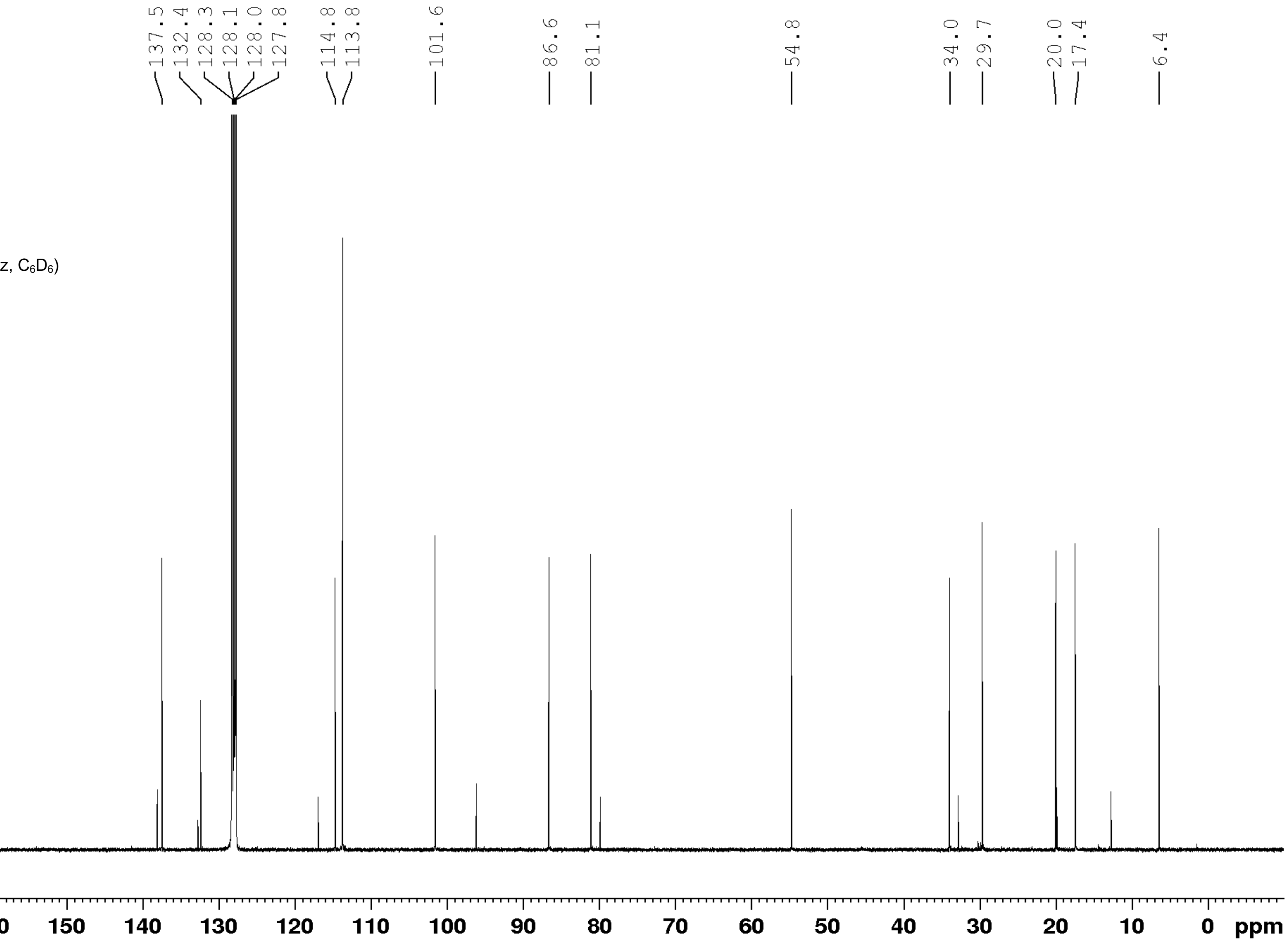

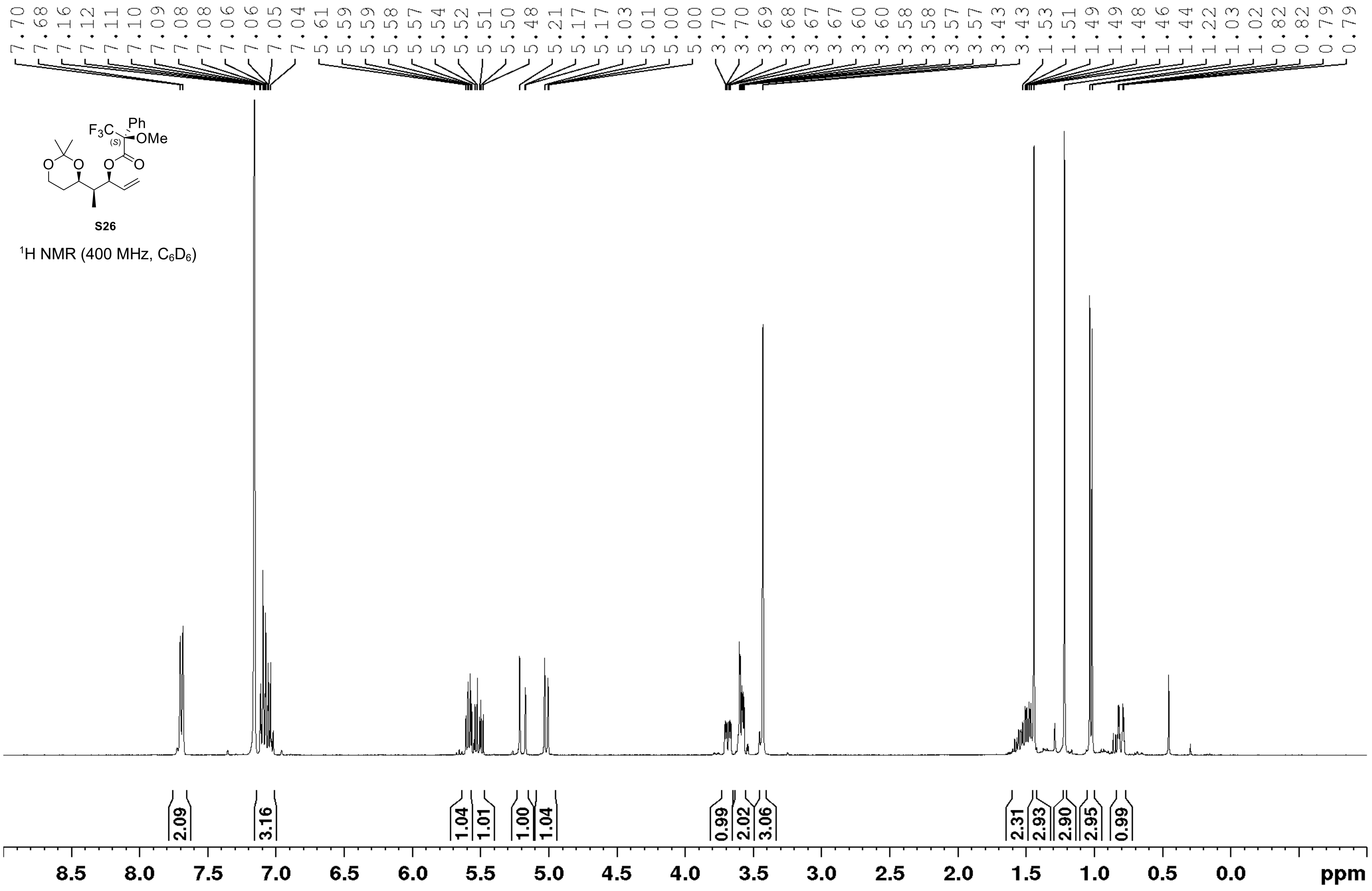
S26
¹H NMR (400 MHz, C₆D₆)
7.70 7.68 7.16 7.12 7.11 7.10 7.09 7.08 7.08 7.06 7.06 7.05 7.04 5.61 5.59 5.59 5.58 5.57 5.54 5.52 5.51 5.50 5.48 5.21 5.17 5.17 5.03 5.01 5.00 5.00 3.70 3.70 3.69 3.68 3.67 3.67 3.60 3.60 3.58 3.58 3.57 3.57 3.43 3.43 1.53 1.51 1.49 1.49 1.48 1.46 1.44 1.22 1.03 1.02 0.82 0.82 0.79 0.79
2.09 3.16 1.04 1.01 1.00 1.04 0.99 2.02 3.06 2.31 2.93 2.90 2.95 0.99
8.5 8.0 7.5 7.0 6.5 6.0 5.5 5.0 4.5 4.0 3.5 3.0 2.5 2.0 1.5 1.0 0.5 0.0 ppm

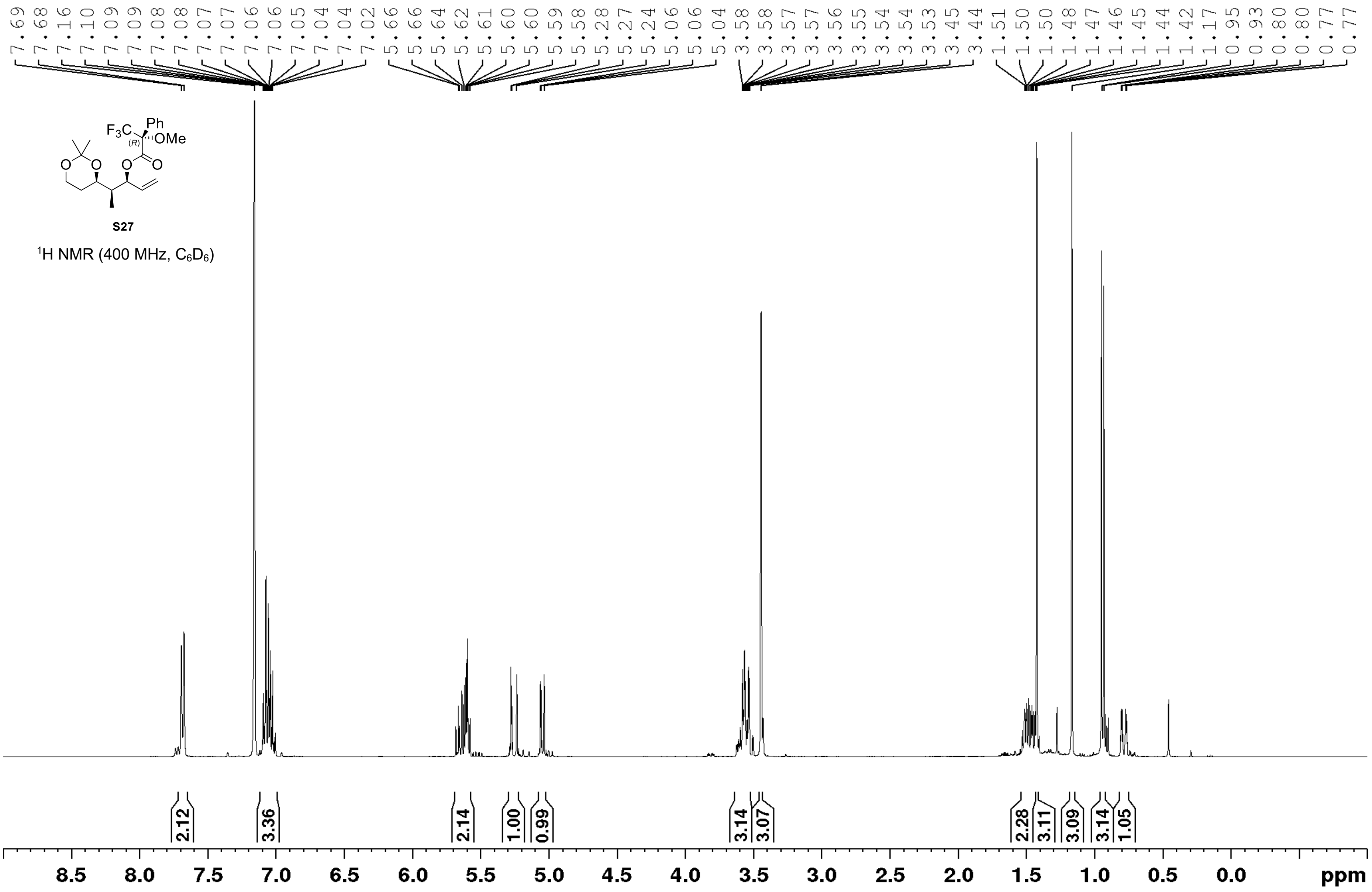
7.69 7.68 7.16 7.10 7.09 7.09 7.08 7.08 7.07 7.07 7.06 7.06 7.05 7.04 7.04 7.02 5.66 5.66 5.64 5.62 5.61 5.60 5.60 5.59 5.58 5.28 5.27 5.24 5.06 5.06 5.04 3.58 3.58 3.57 3.57 3.56 3.55 3.54 3.54 3.53 3.45 3.44 1.51 1.50 1.50 1.48 1.47 1.46 1.45 1.44 1.42 1.17 0.95 0.93 0.80 0.80 0.77 0.77
F3C
Ph
(R)
OMe
O
O
O
O
O
S27
1H NMR (400 MHz, C6D6)
2.12
3.36
2.14
1.00
0.99
3.14
3.07
2.28
3.11
3.09
3.14
1.05
8.5 8.0 7.5 7.0 6.5 6.0 5.5 5.0 4.5 4.0 3.5 3.0 2.5 2.0 1.5 1.0 0.5 0.0 ppm

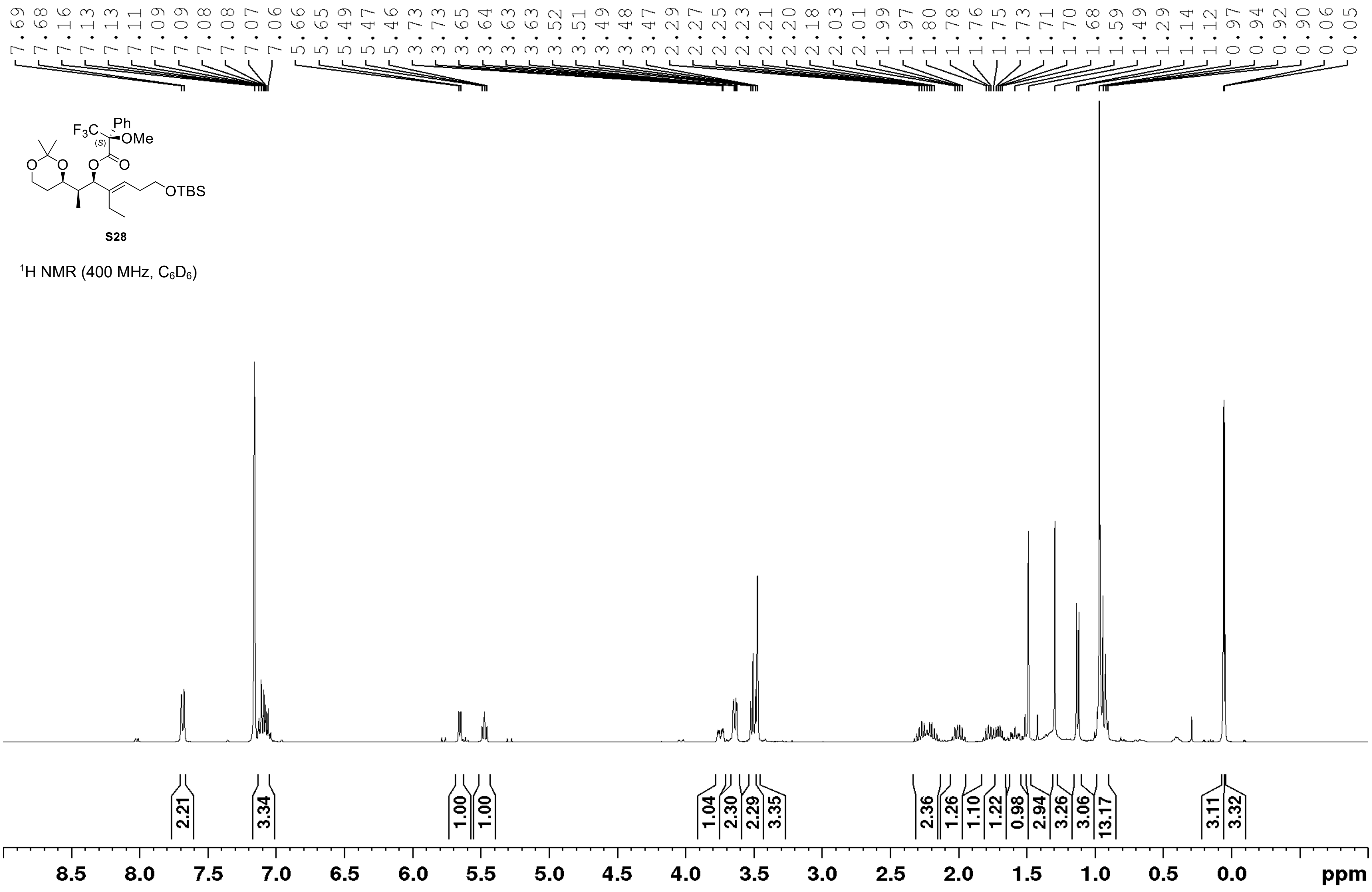

7.69 7.68 7.16 7.13 7.13 7.11 7.09 7.08 7.08 7.07 7.06 5.66 5.65 5.49 5.47 5.46 3.73 3.73 3.65 3.64 3.63 3.63 3.52 3.51 3.49 3.48 3.47 2.29 2.27 2.25 2.23 2.21 2.20 2.18 2.03 2.01 1.99 1.97 1.80 1.78 1.76 1.75 1.73 1.71 1.70 1.68 1.59 1.49 1.29 1.14 1.12 0.97 0.94 0.92 0.90 0.06 0.05
F3C
Ph
(S)
OMe
O
O
O
O
OTBS
S28
1H NMR (400 MHz, C6D6)
2.21 3.34 1.00 1.00 1.04 2.30 2.29 3.35 2.36 1.26 1.10 1.22 0.98 2.94 3.26 3.06 13.17 3.11 3.32
8.5 8.0 7.5 7.0 6.5 6.0 5.5 5.0 4.5 4.0 3.5 3.0 2.5 2.0 1.5 1.0 0.5 0.0 ppm

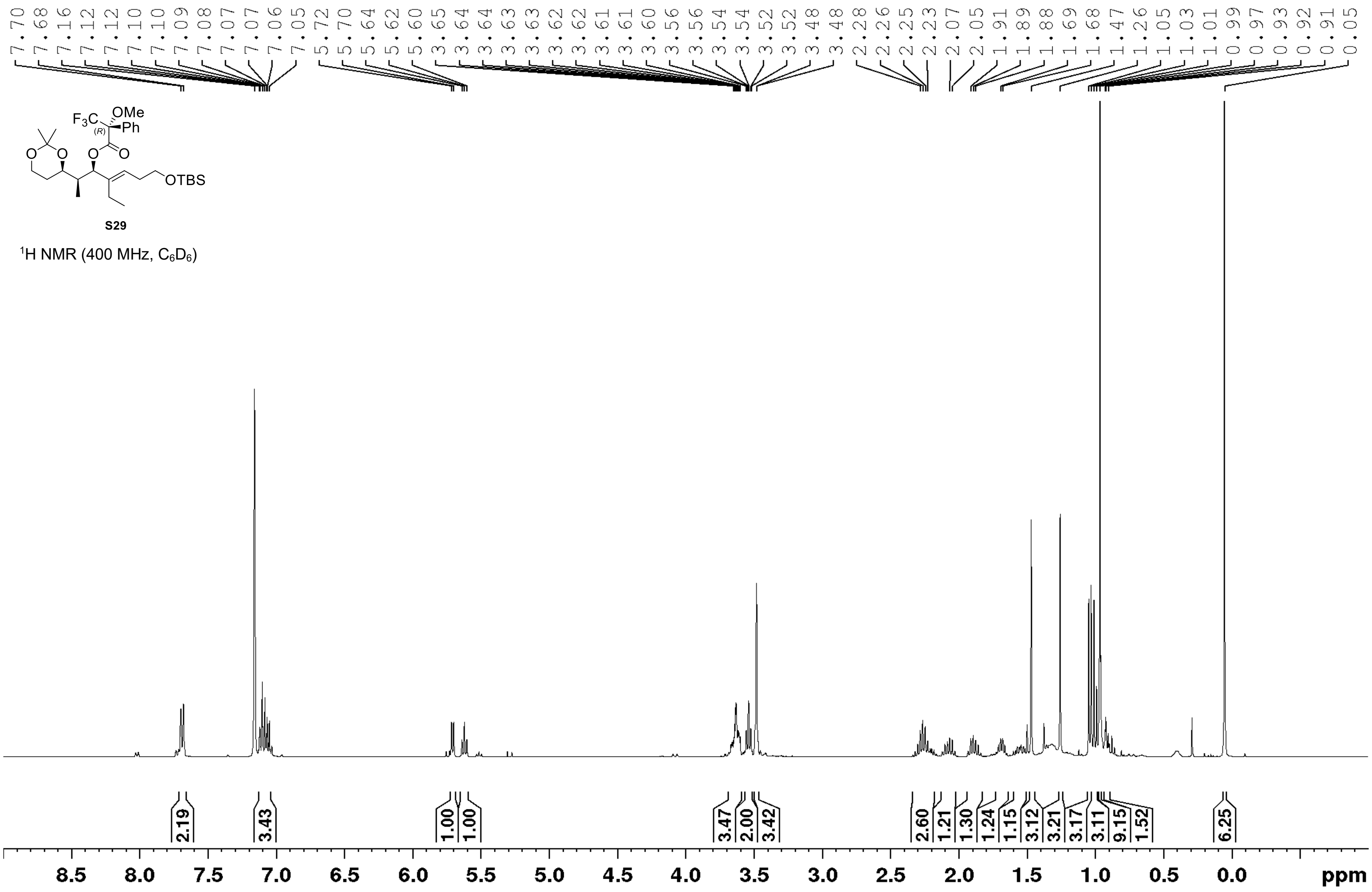

F3C
OMe
(R)
Ph
O
O
O
O
OTBS
S29
1H NMR (400 MHz, C6D6)
7.70 7.68 7.16 7.12 7.12 7.10 7.10 7.09 7.08 7.07 7.07 7.06 7.05 5.72 5.70 5.64 5.62 5.60 3.65 3.64 3.64 3.63 3.63 3.62 3.62 3.61 3.61 3.60 3.56 3.56 3.54 3.54 3.52 3.52 3.48 3.48 2.28 2.26 2.25 2.23 2.07 2.05 1.91 1.89 1.88 1.69 1.68 1.47 1.26 1.05 1.03 1.01 0.99 0.97 0.93 0.92 0.91 0.05
2.19 3.43 1.00 1.00 3.47 2.00 3.42 2.60 1.21 1.30 1.24 1.15 3.12 3.21 3.17 3.11 9.15 1.52 6.25
8.5 8.0 7.5 7.0 6.5 6.0 5.5 5.0 4.5 4.0 3.5 3.0 2.5 2.0 1.5 1.0 0.5 0.0 ppm

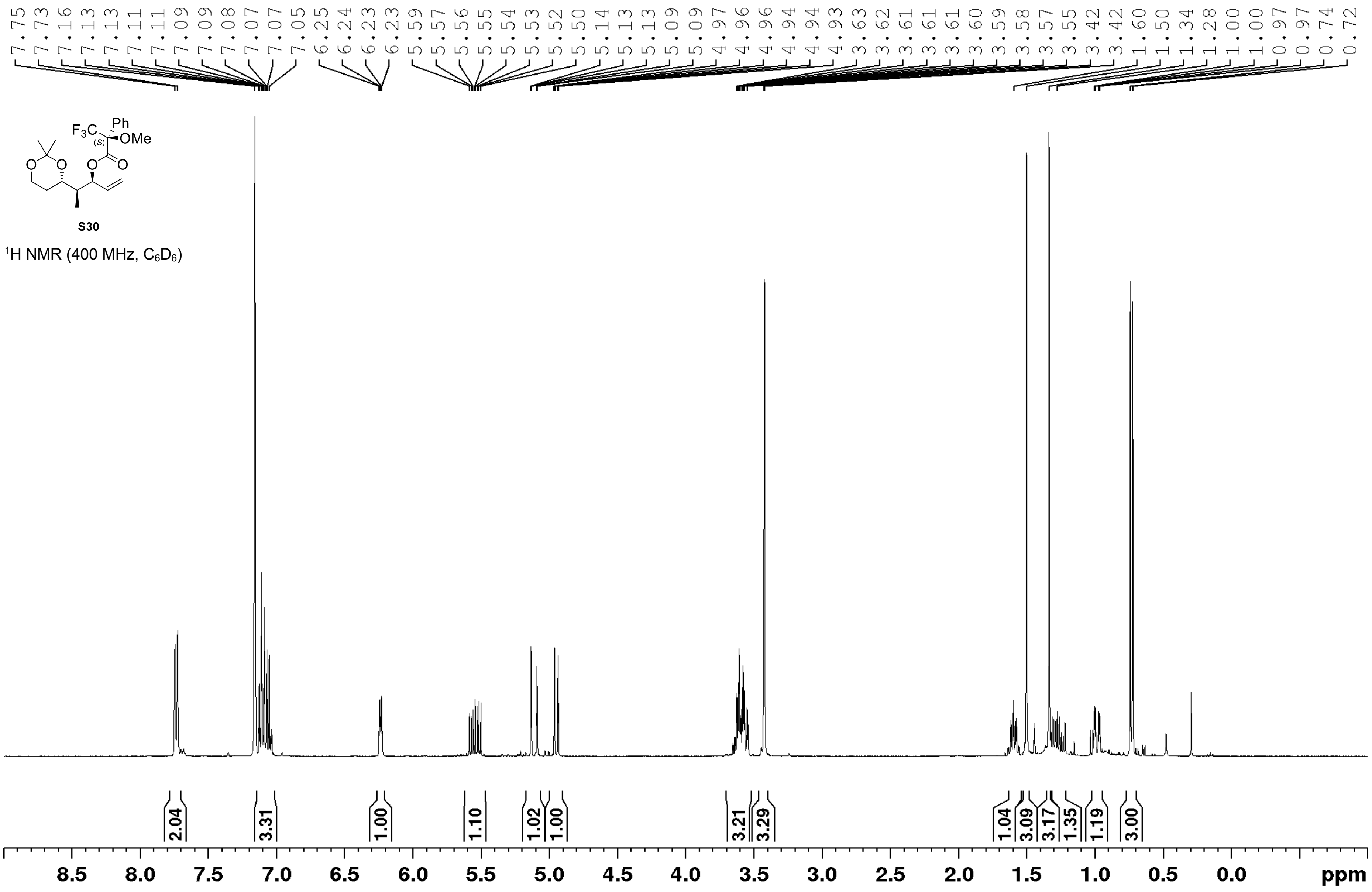
S30
^{1}H NMR (400 MHz, C_6D_6)
ppm

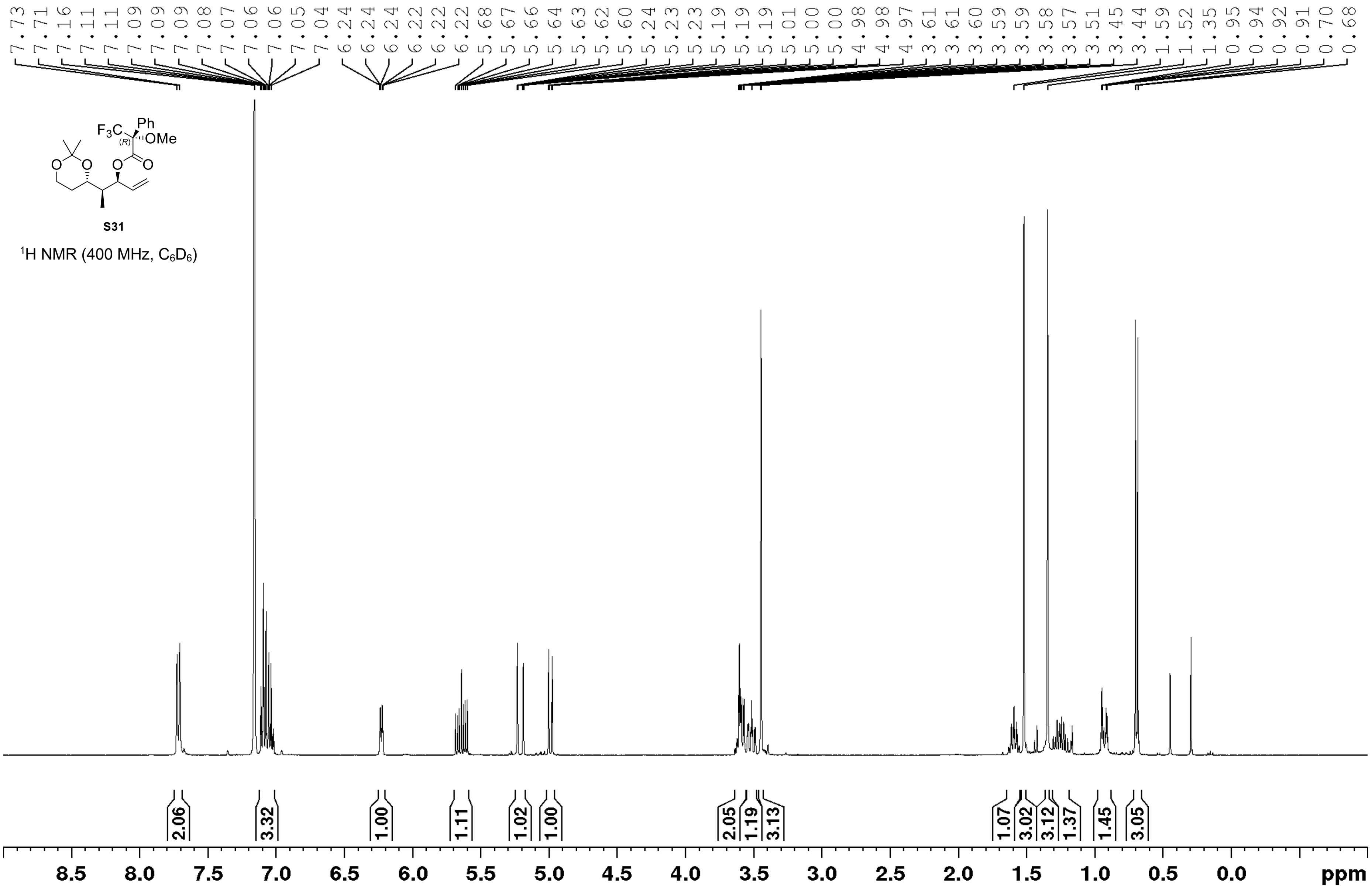
7.73 7.71 7.16 7.11 7.11 7.09 7.09 7.09 7.08 7.07 7.06 7.06 7.05 7.04 6.24 6.24 6.24 6.22 6.22 6.22 5.68 5.67 5.66 5.64 5.63 5.62 5.60 5.24 5.23 5.23 5.19 5.19 5.19 5.01 5.00 5.00 4.98 4.98 4.97 3.61 3.61 3.60 3.59 3.59 3.58 3.57 3.51 3.45 3.44 1.59 1.52 1.35 0.95 0.94 0.92 0.91 0.70 0.68
F_3C
Ph
(R)
OMe
O
O
O
O
S31
^{1}H NMR (400 MHz, C_6D_6)
2.06
3.32
1.00
1.11
1.02
1.00
2.05
1.19
3.13
1.07
3.02
3.12
1.37
1.45
3.05
8.5 8.0 7.5 7.0 6.5 6.0 5.5 5.0 4.5 4.0 3.5 3.0 2.5 2.0 1.5 1.0 0.5 0.0
ppm

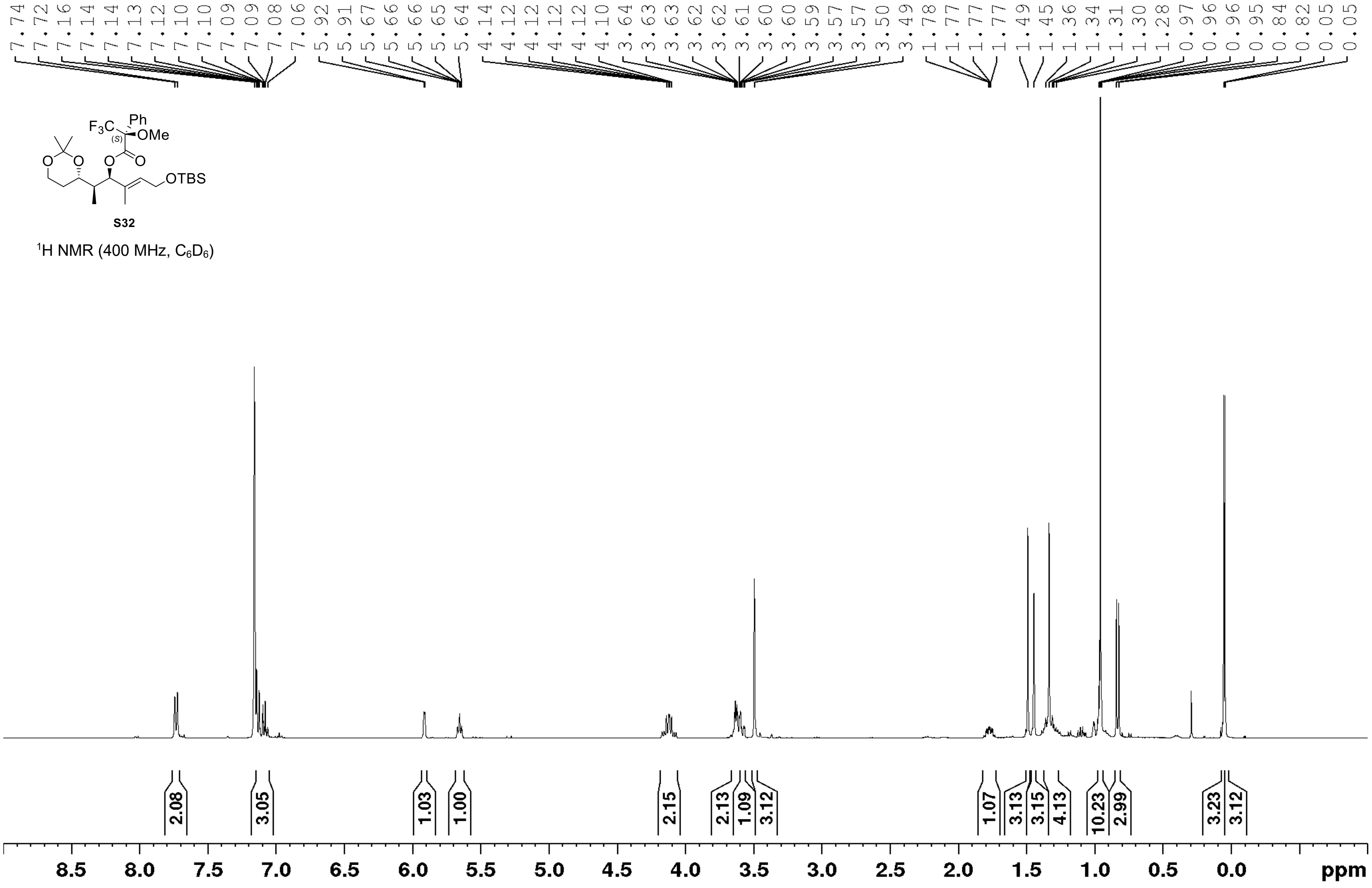
7.74 7.72 7.16 7.14 7.14 7.13 7.12 7.10 7.10 7.09 7.08 7.06 5.92 5.91 5.67 5.66 5.66 5.65 5.64 4.14 4.12 4.12 4.12 4.10 3.64 3.63 3.63 3.62 3.62 3.61 3.60 3.59 3.57 3.57 3.50 3.49 1.78 1.77 1.77 1.77 1.49 1.45 1.36 1.34 1.31 1.30 1.28 0.97 0.96 0.96 0.95 0.84 0.82 0.05 0.05
F3C
(S)
Ph
OMe
O
O
O
O
OTBS
S32
1H NMR (400 MHz, C6D6)
2.08
3.05
1.03
1.00
2.15
2.13
1.09
3.12
1.07
3.13
3.15
4.13
10.23
2.99
3.23
3.12
8.5 8.0 7.5 7.0 6.5 6.0 5.5 5.0 4.5 4.0 3.5 3.0 2.5 2.0 1.5 1.0 0.5 0.0
ppm

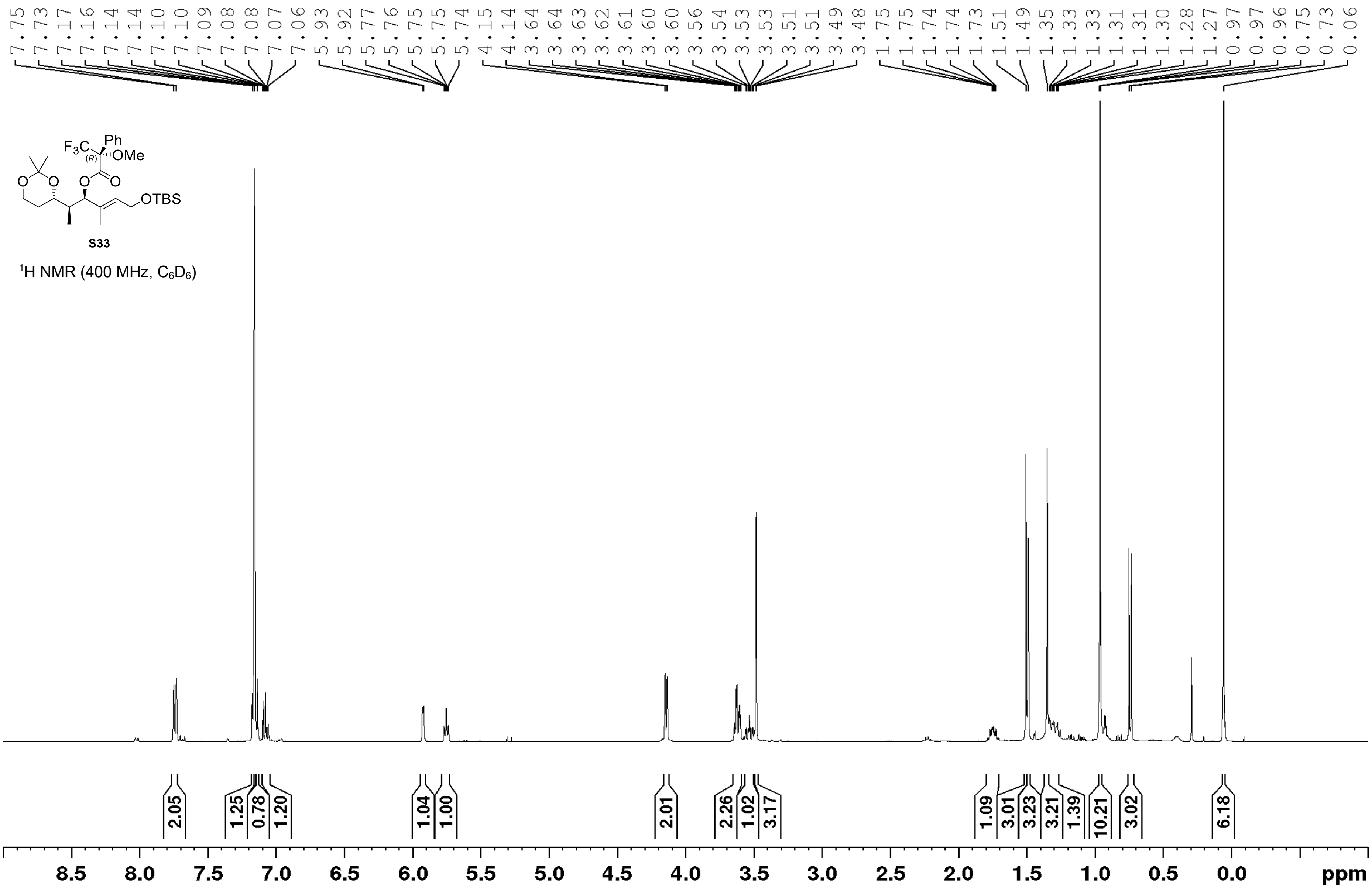
7.75 7.73 7.17 7.16 7.14 7.14 7.10 7.09 7.08 7.08 7.07 7.06 5.93 5.92 5.77 5.76 5.75 5.75 5.74 4.15 4.14 3.64 3.64 3.63 3.62 3.61 3.60 3.60 3.56 3.54 3.53 3.53 3.51 3.51 3.49 3.48 1.75 1.75 1.74 1.74 1.73 1.51 1.49 1.35 1.33 1.33 1.31 1.31 1.30 1.28 1.27 0.97 0.97 0.96 0.75 0.73 0.06
F3C
Ph
(R)
OMe
O
O
O
O
O
OTBS
S33
1H NMR (400 MHz, C6D6)
2.05 1.25 0.78 1.20 1.04 1.00 2.01 2.26 1.02 3.17 1.09 3.01 3.23 3.21 1.39 10.21 3.02 6.18
8.5 8.0 7.5 7.0 6.5 6.0 5.5 5.0 4.5 4.0 3.5 3.0 2.5 2.0 1.5 1.0 0.5 0.0 ppm

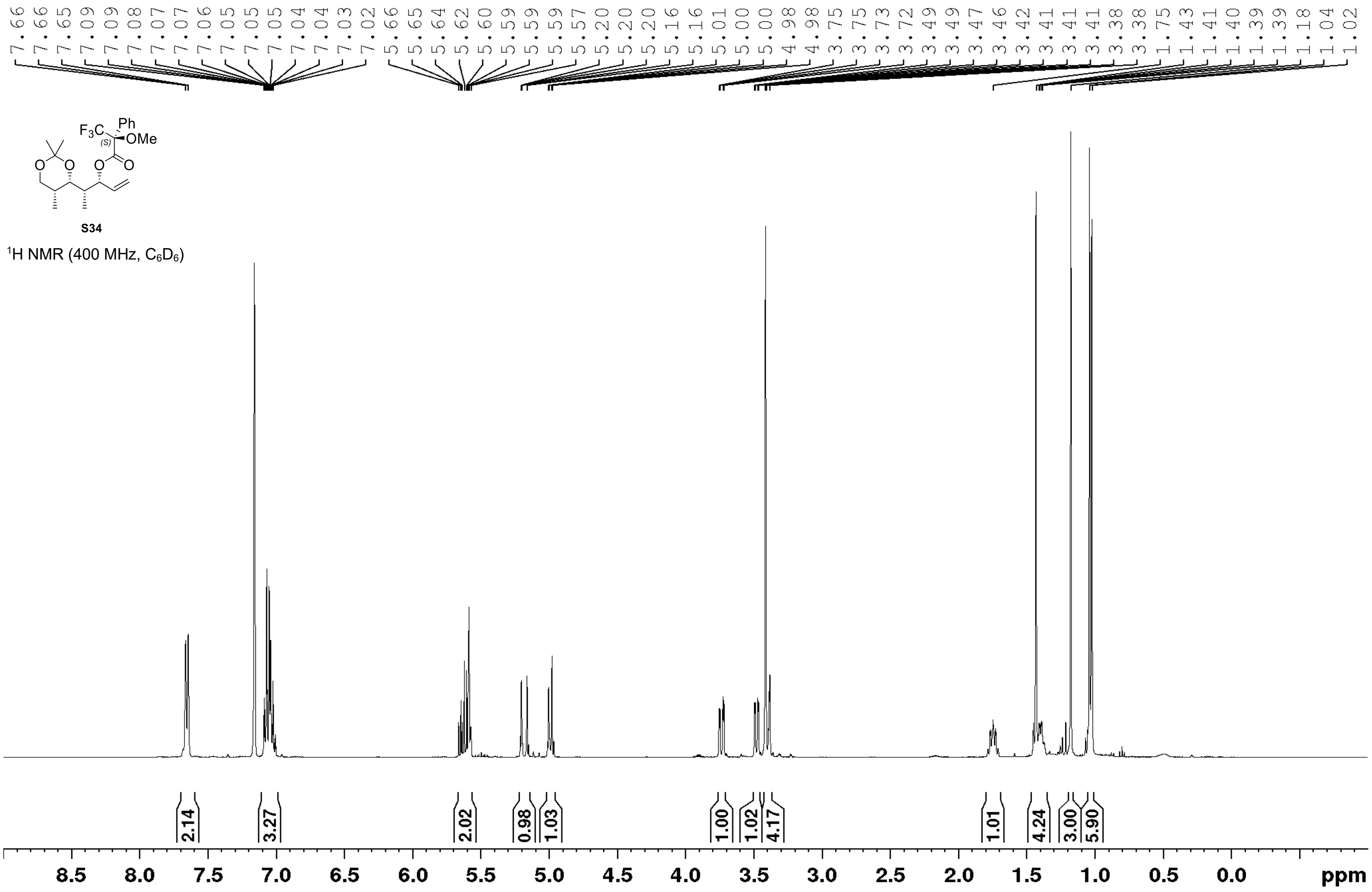
F3C
Ph
(S)
OMe
O
O
O
O
S34
1H NMR (400 MHz, C6D6)
7.66
7.66
7.65
7.09
7.09
7.08
7.07
7.07
7.06
7.05
7.05
7.04
7.04
7.03
7.02
5.66
5.65
5.64
5.62
5.60
5.59
5.59
5.59
5.57
5.20
5.20
5.20
5.16
5.16
5.01
5.00
5.00
4.98
4.98
3.75
3.75
3.73
3.72
3.49
3.49
3.47
3.46
3.42
3.41
3.41
3.41
3.38
3.38
1.75
1.43
1.41
1.40
1.39
1.39
1.18
1.04
1.02
2.14
3.27
2.02
0.98
1.03
1.00
1.02
4.17
1.01
4.24
3.00
5.90
8.5
8.0
7.5
7.0
6.5
6.0
5.5
5.0
4.5
4.0
3.5
3.0
2.5
2.0
1.5
1.0
0.5
0.0
ppm

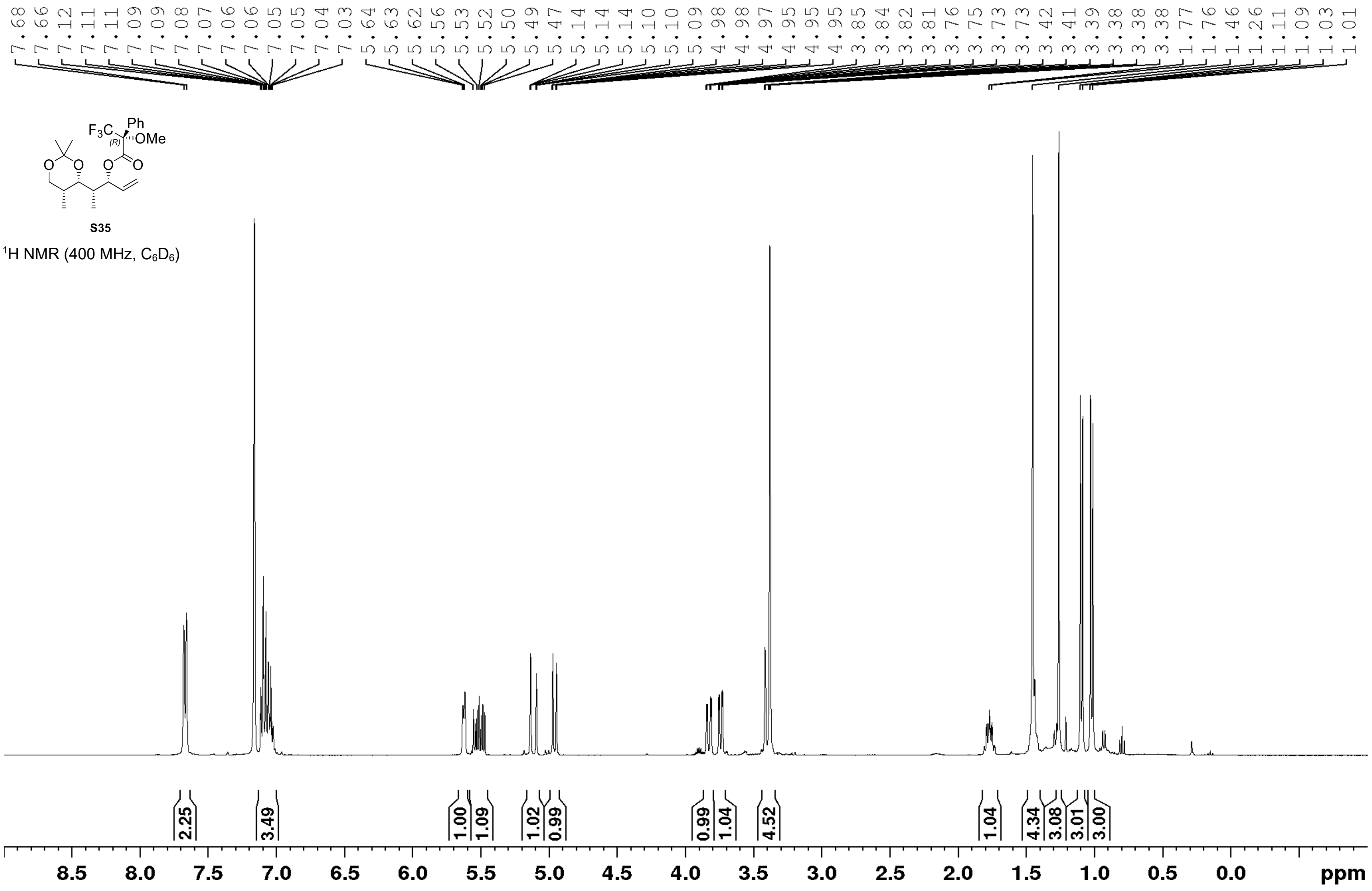

S35
^{1}H NMR (400 MHz, C_6D_6)

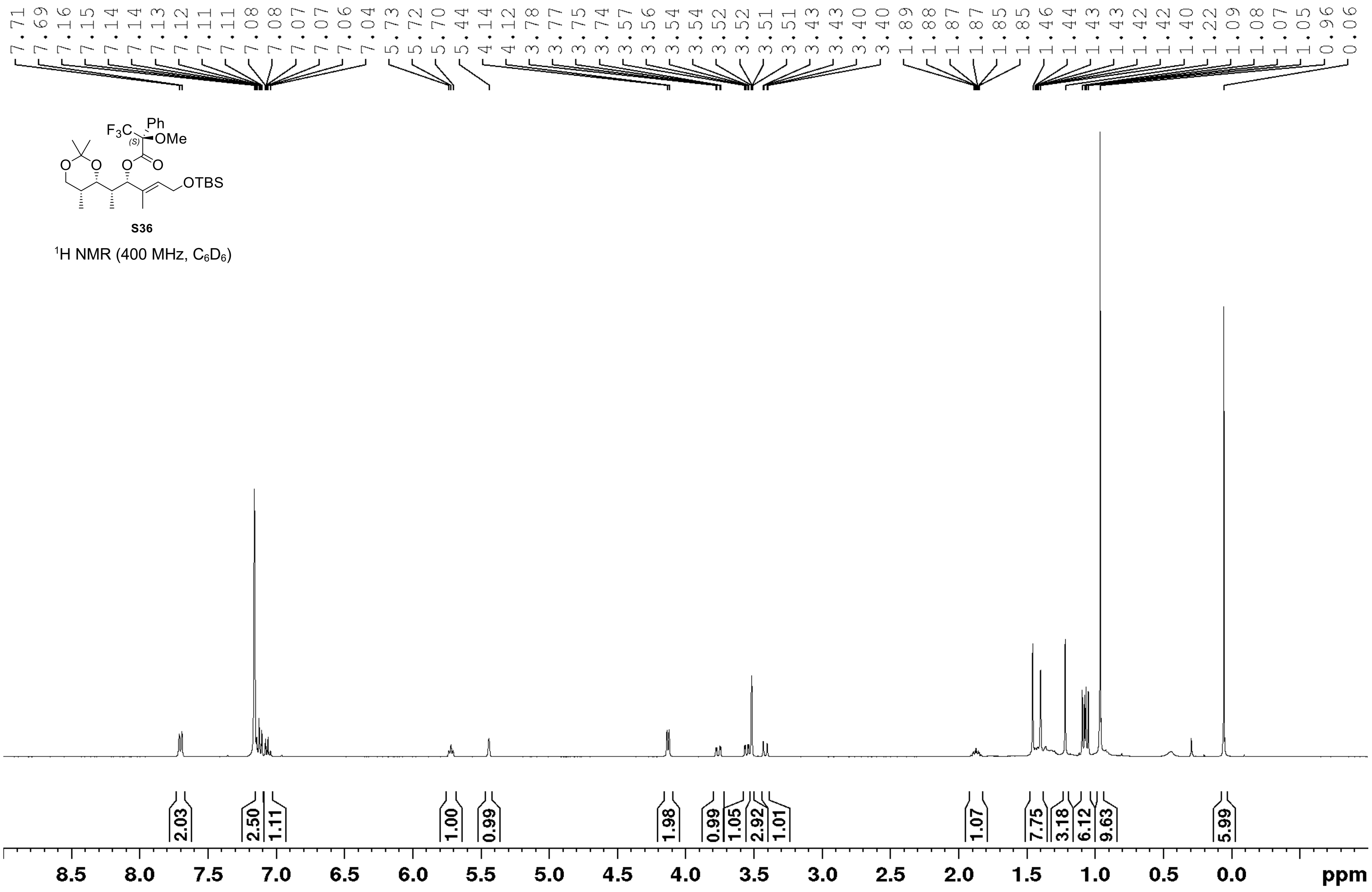
7.71 7.69 7.16 7.15 7.14 7.14 7.13 7.12 7.11 7.11 7.08 7.08 7.07 7.07 7.06 7.04 5.73 5.72 5.70 5.44 4.14 4.12 3.78 3.77 3.75 3.74 3.57 3.56 3.54 3.54 3.52 3.52 3.51 3.51 3.43 3.43 3.40 3.40 1.89 1.88 1.87 1.87 1.85 1.85 1.46 1.44 1.43 1.43 1.42 1.42 1.40 1.22 1.09 1.08 1.07 1.05 0.96 0.06
Ph
F_3C
(S)
OMe
O
O
O
O
OTBS
S36
^{1}H NMR (400 MHz, C_6D_6)
2.03
2.50
1.11
1.00
0.99
1.98
0.99
1.05
2.92
1.01
1.07
7.75
3.18
6.12
9.63
5.99
8.5 8.0 7.5 7.0 6.5 6.0 5.5 5.0 4.5 4.0 3.5 3.0 2.5 2.0 1.5 1.0 0.5 0.0
ppm

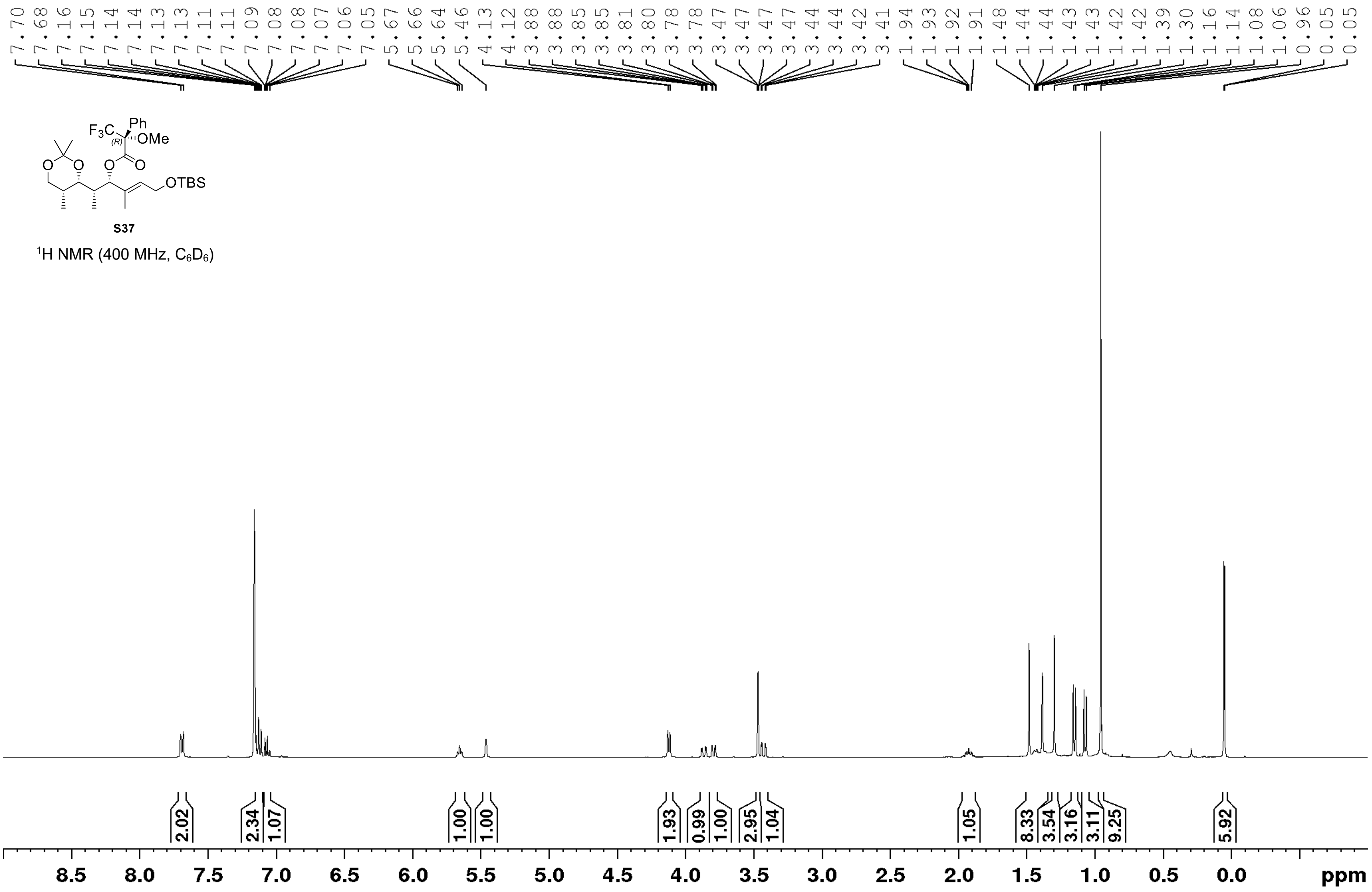
S37
^{1}H NMR (400 MHz, C_6D_6)

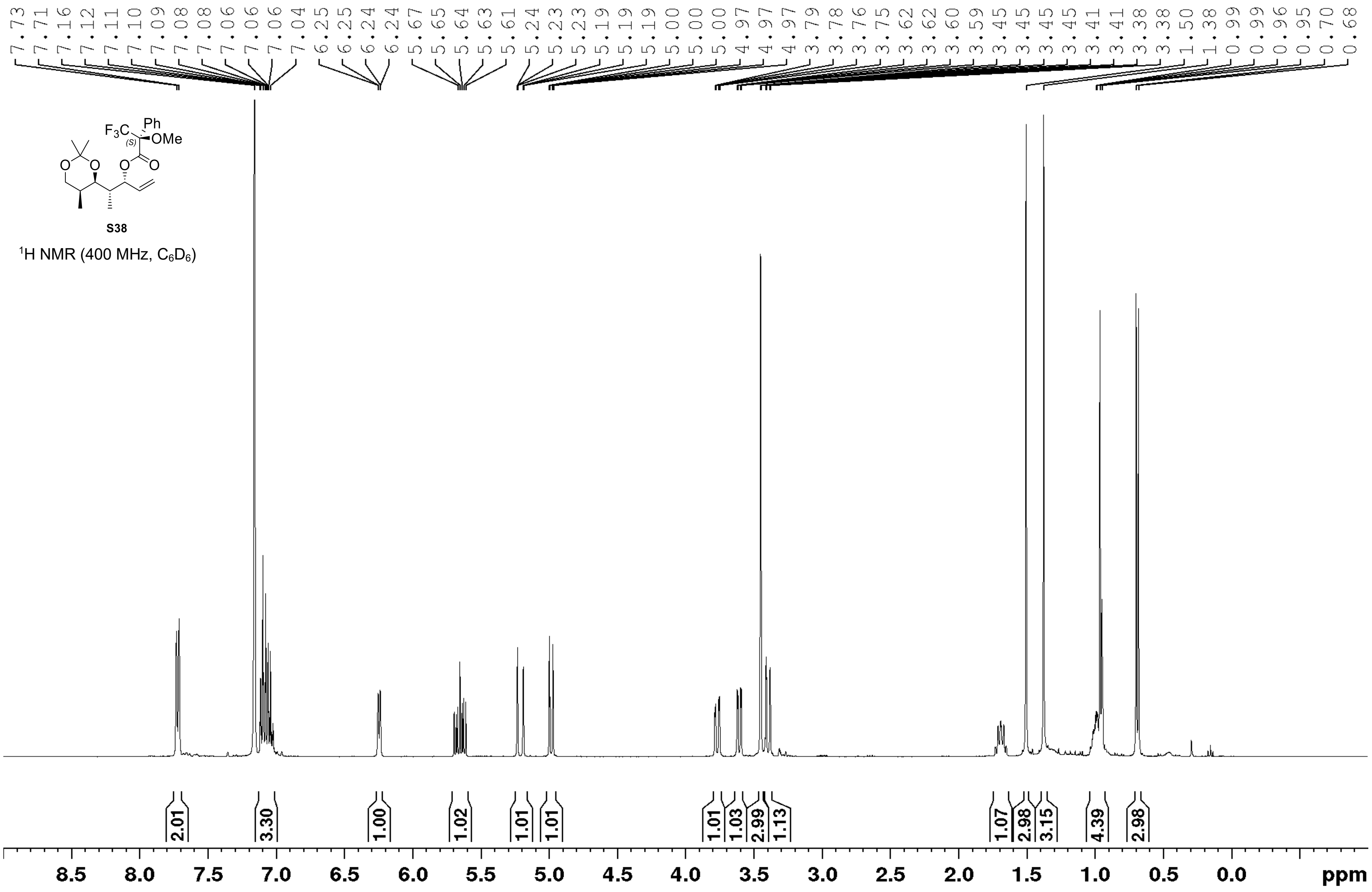
F3C Ph OMe
(S)
O O O O
S38
1H NMR (400 MHz, C6D6)
7.73 7.71 7.16 7.12 7.11 7.10 7.09 7.08 7.08 7.06 7.06 7.04 6.25 6.25 6.24 6.24 5.67 5.65 5.64 5.63 5.61 5.24 5.23 5.23 5.19 5.19 5.19 5.00 5.00 5.00 4.97 4.97 4.97 3.79 3.78 3.76 3.75 3.62 3.62 3.60 3.59 3.45 3.45 3.45 3.45 3.41 3.41 3.38 3.38 1.50 1.38 0.99 0.99 0.96 0.95 0.70 0.68
2.01 3.30 1.00 1.02 1.01 1.01 1.01 1.03 2.99 1.13 1.07 2.98 3.15 4.39 2.98
8.5 8.0 7.5 7.0 6.5 6.0 5.5 5.0 4.5 4.0 3.5 3.0 2.5 2.0 1.5 1.0 0.5 0.0 ppm

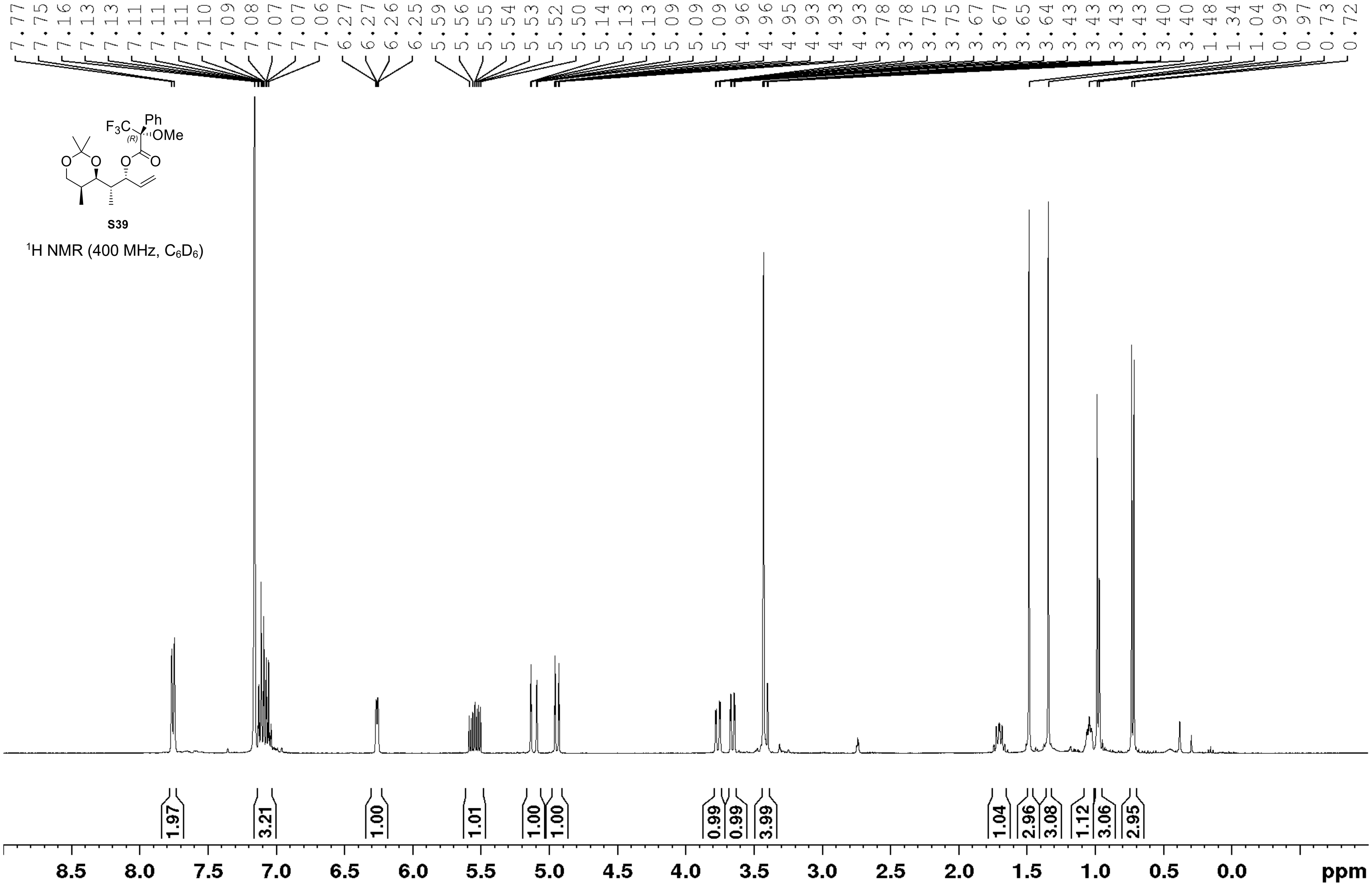
F3C Ph OMe
(R)
O O
O O
S39
1H NMR (400 MHz, C6D6)
1.97 3.21 1.00 1.01 1.00 1.00 0.99 0.99 3.99 1.04 2.96 3.08 1.12 3.06 2.95
8.5 8.0 7.5 7.0 6.5 6.0 5.5 5.0 4.5 4.0 3.5 3.0 2.5 2.0 1.5 1.0 0.5 0.0 ppm

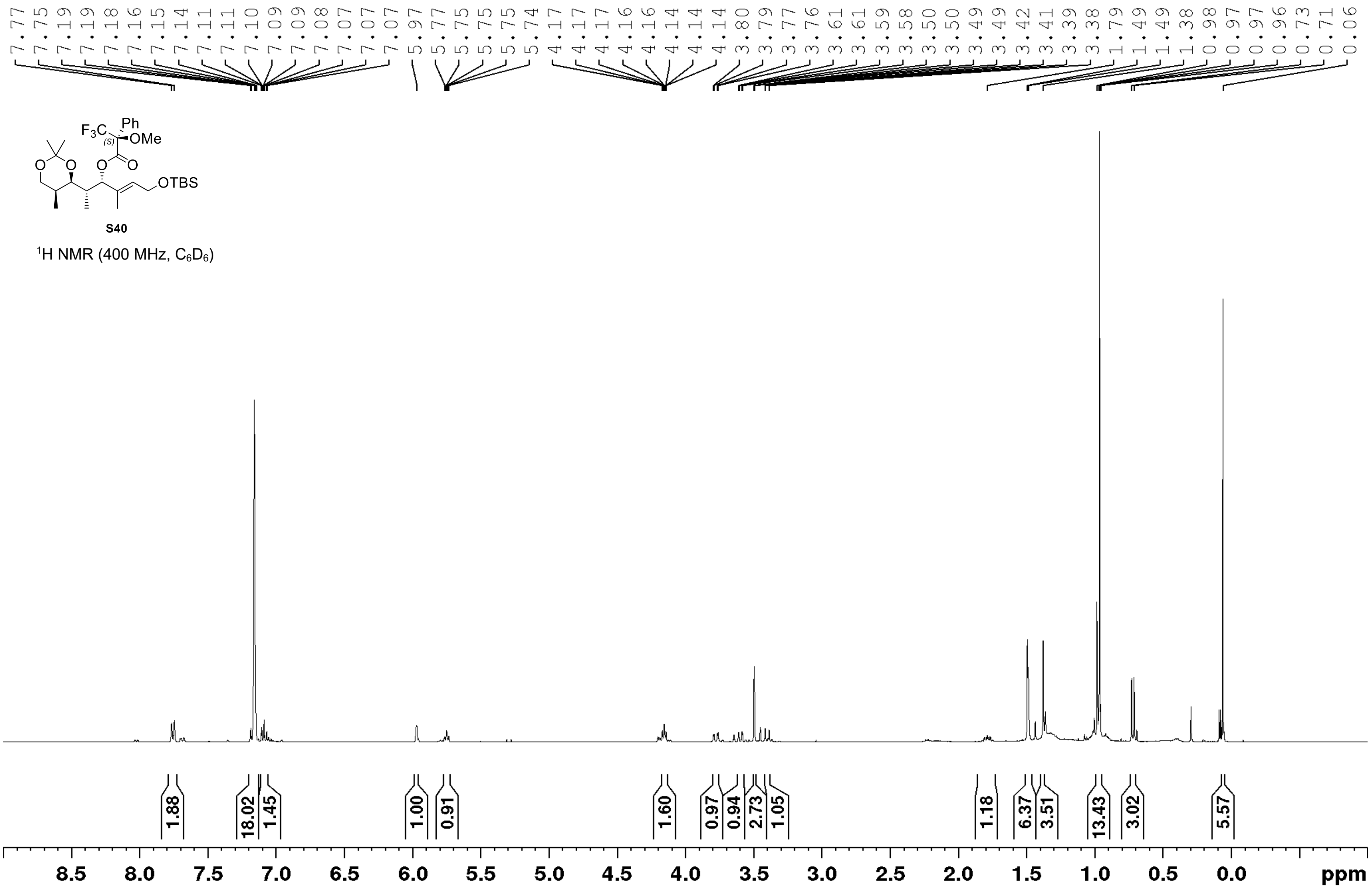
S40
^{1}H NMR (400 MHz, C_6D_6)
ppm

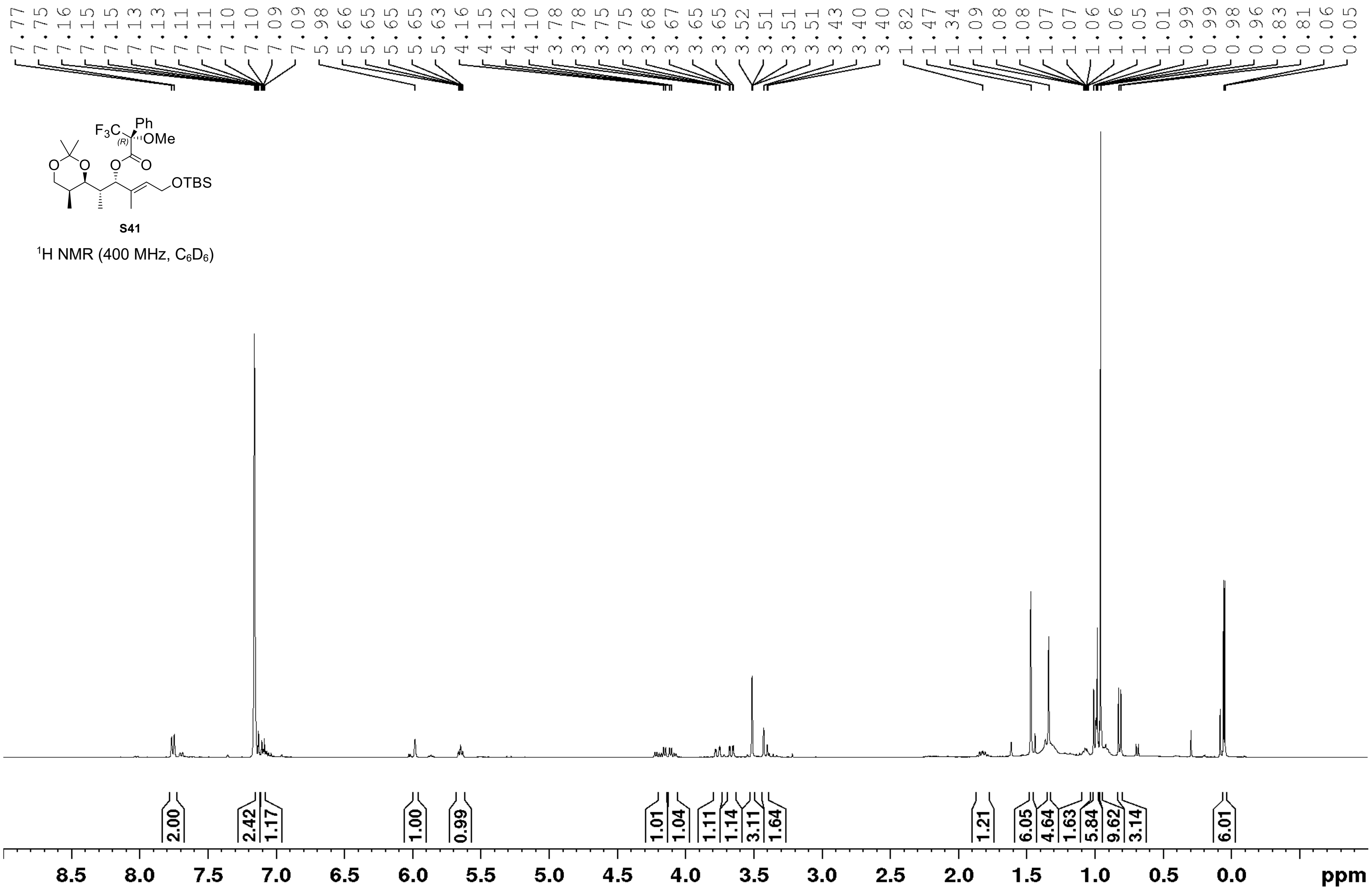

S41

^{1}H NMR (400 MHz, C_6D_6)

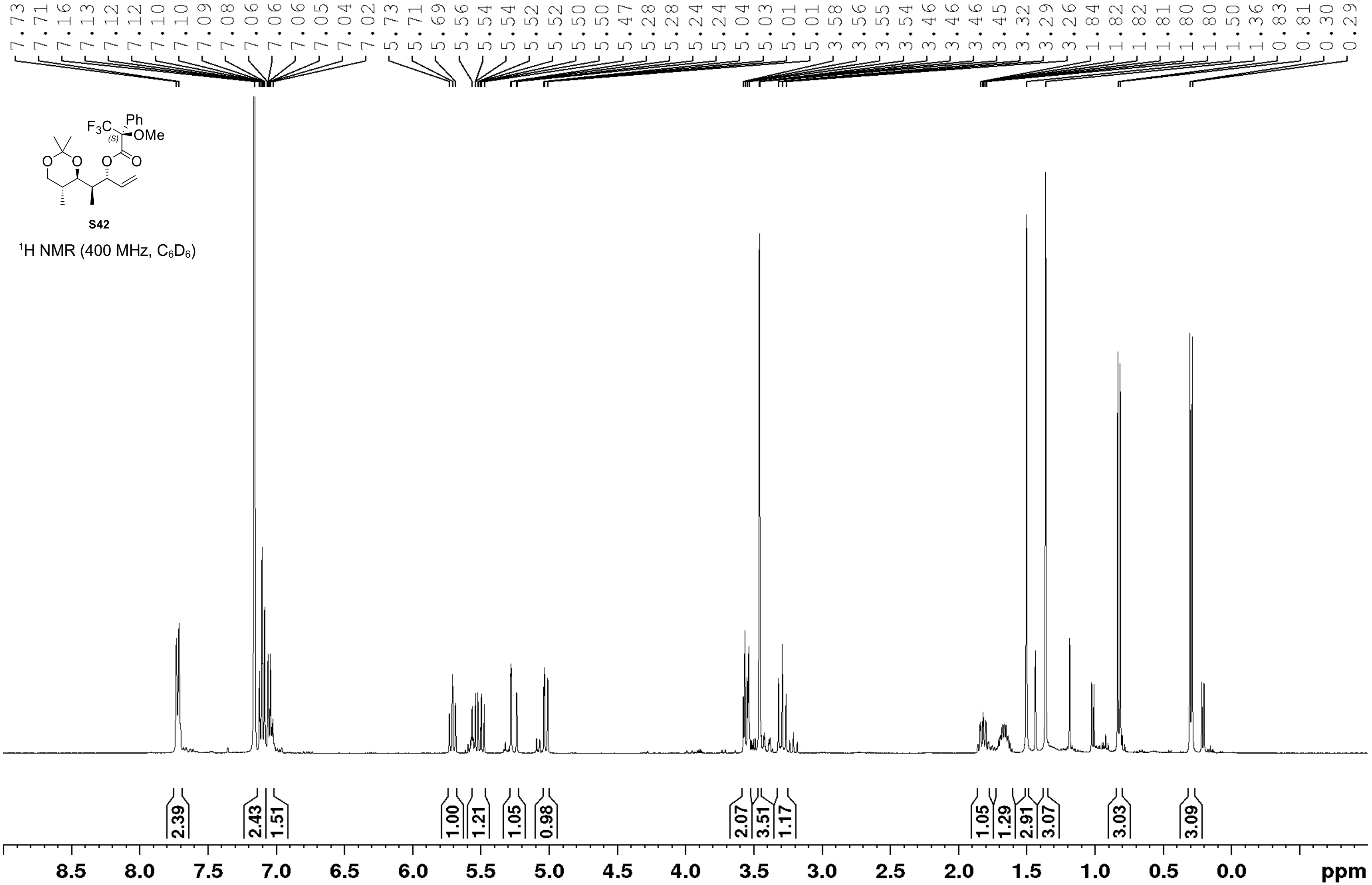

7.73 7.71 7.16 7.13 7.12 7.12 7.10 7.09 7.08 7.06 7.06 7.06 7.05 7.04 7.02 5.73 5.71 5.69 5.56 5.54 5.54 5.52 5.52 5.50 5.50 5.47 5.28 5.28 5.24 5.24 5.04 5.03 5.01 5.01 3.58 3.56 3.55 3.54 3.46 3.46 3.46 3.45 3.32 3.29 3.26 1.84 1.82 1.82 1.81 1.80 1.80 1.50 1.36 0.83 0.81 0.30 0.29
F3C
(S)
Ph
OMe
O
S42
1H NMR (400 MHz, C6D6)
2.39 2.43 1.51 1.00 1.21 1.05 0.98 2.07 3.51 1.17 1.05 1.29 2.91 3.07 3.03 3.09
8.5 8.0 7.5 7.0 6.5 6.0 5.5 5.0 4.5 4.0 3.5 3.0 2.5 2.0 1.5 1.0 0.5 0.0 ppm

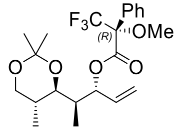

S43

^{1}H NMR (400 MHz, C_6D_6)

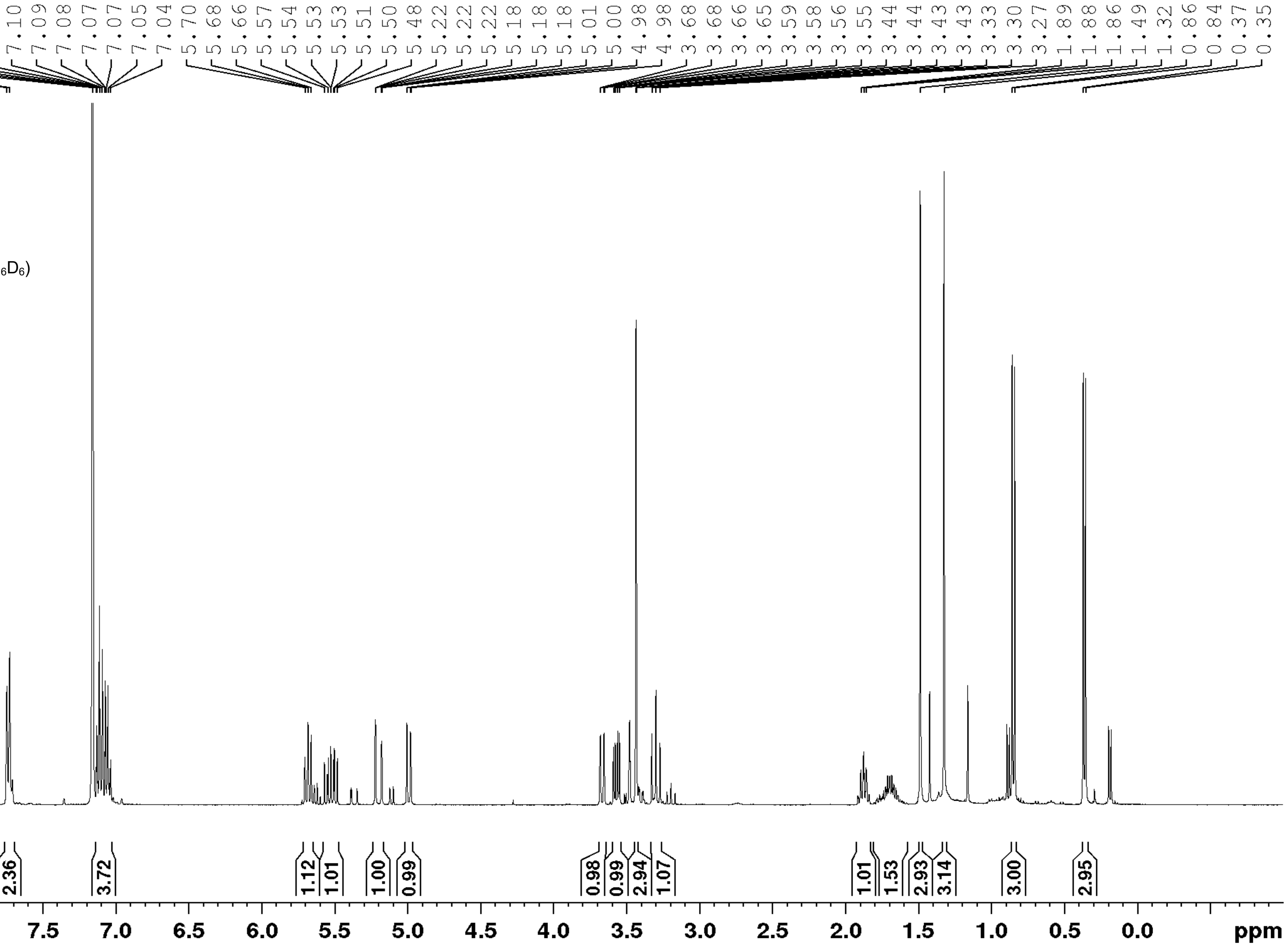

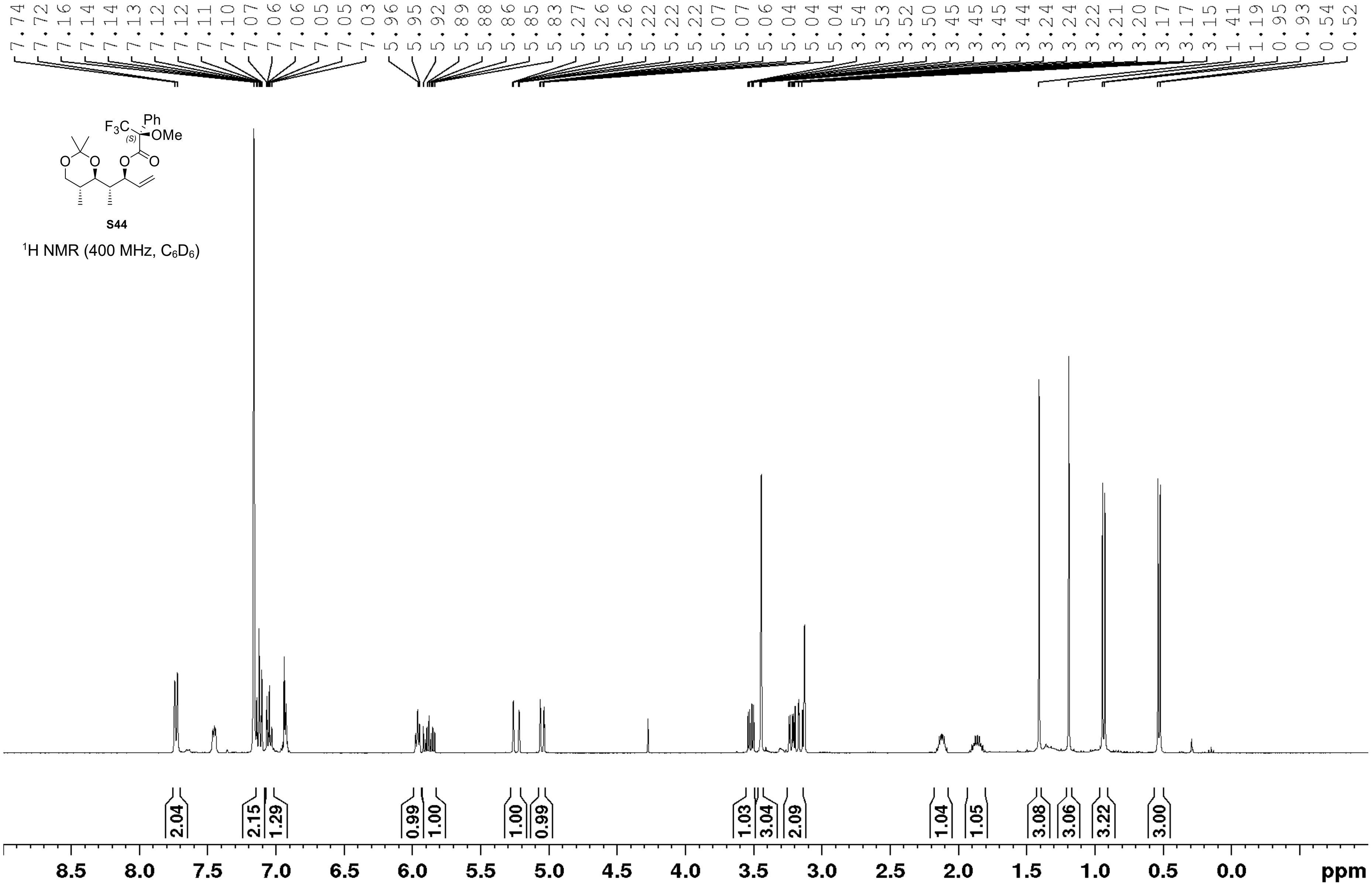
S44
^{1}H NMR (400 MHz, C_6D_6)
ppm

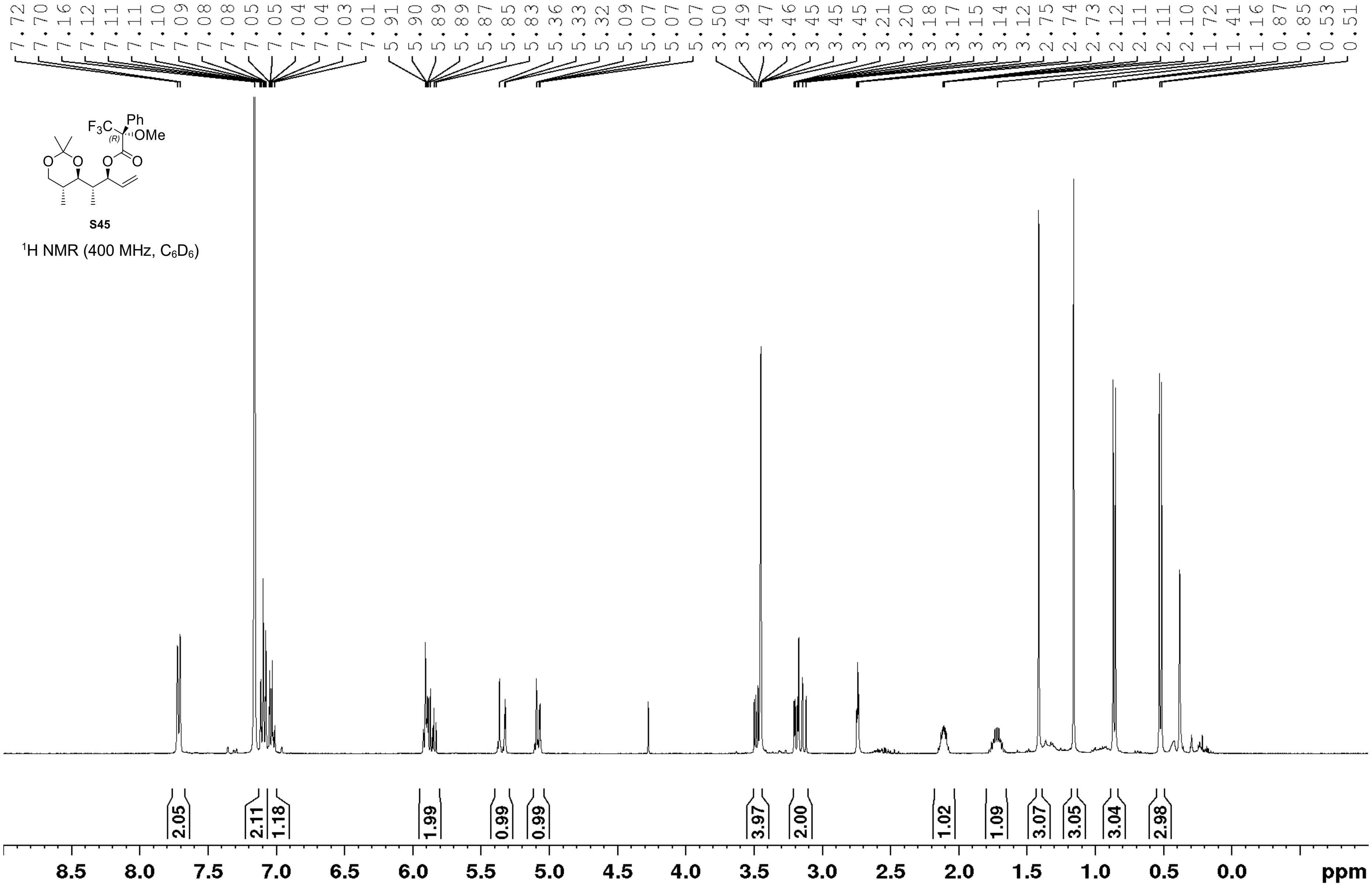
F3C
Ph
(R)
OMe
O
O
O
O
S45
1H NMR (400 MHz, C6D6)
7.72 7.70 7.16 7.12 7.11 7.11 7.10 7.09 7.08 7.08 7.05 7.05 7.04 7.04 7.03 7.01 5.91 5.90 5.89 5.89 5.87 5.85 5.83 5.36 5.33 5.32 5.09 5.07 5.07 5.07 3.50 3.49 3.47 3.46 3.45 3.45 3.45 3.21 3.20 3.18 3.17 3.15 3.14 3.12 2.75 2.74 2.73 2.12 2.11 2.11 2.10 1.72 1.41 1.16 0.87 0.85 0.53 0.51
2.05
2.11
1.18
1.99
0.99
0.99
3.97
2.00
1.02
1.09
3.07
3.05
3.04
2.98
8.5 8.0 7.5 7.0 6.5 6.0 5.5 5.0 4.5 4.0 3.5 3.0 2.5 2.0 1.5 1.0 0.5 0.0 ppm

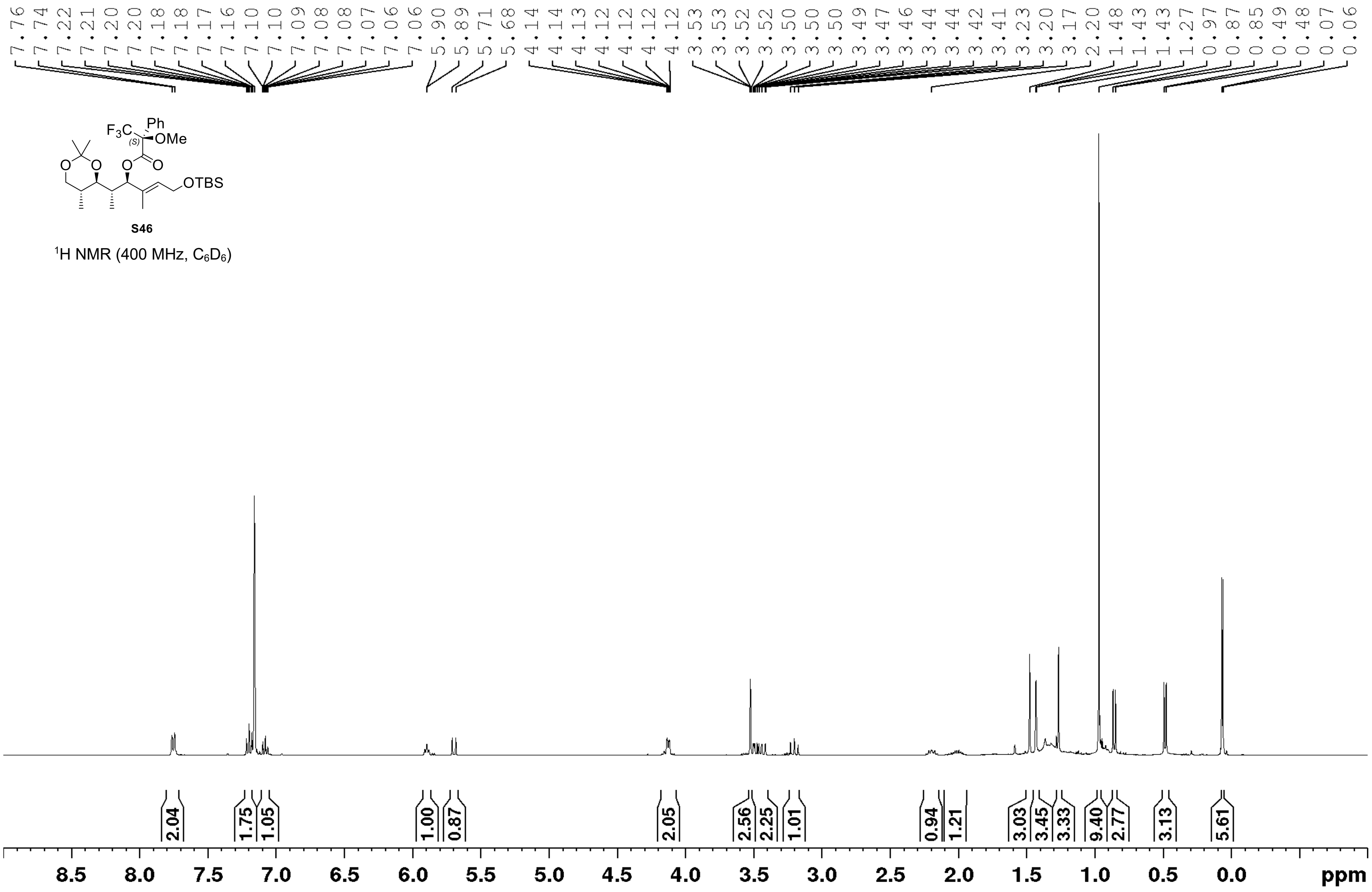

7.76 7.74 7.22 7.21 7.20 7.20 7.18 7.18 7.17 7.16 7.10 7.10 7.09 7.08 7.08 7.07 7.06 7.06 5.90 5.89 5.71 5.68 4.14 4.14 4.13 4.12 4.12 4.12 4.12 3.53 3.53 3.52 3.52 3.50 3.50 3.49 3.47 3.46 3.44 3.44 3.42 3.41 3.23 3.20 3.17 2.20 1.48 1.43 1.43 1.27 0.97 0.87 0.85 0.49 0.48 0.07 0.06
F3C
Ph
OMe
(S)
O
O
O
O
OTBS
S46
1H NMR (400 MHz, C6D6)
2.04 1.75 1.05 1.00 0.87 2.05 2.56 2.25 1.01 0.94 1.21 3.03 3.45 3.33 9.40 2.77 3.13 5.61
8.5 8.0 7.5 7.0 6.5 6.0 5.5 5.0 4.5 4.0 3.5 3.0 2.5 2.0 1.5 1.0 0.5 0.0 ppm

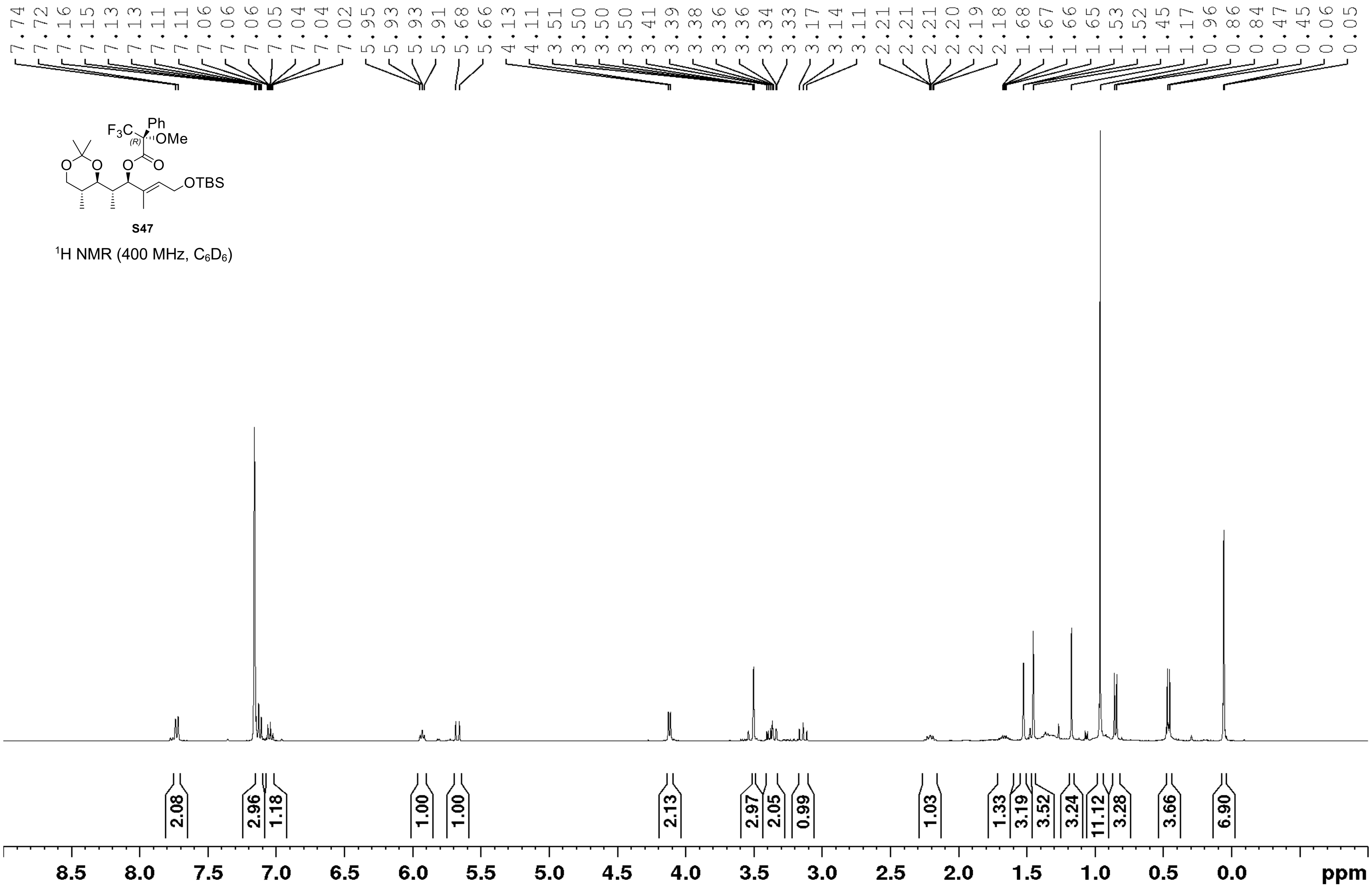
Ph
F3C
(R)
OMe
O
O
O
O
OTBS
S47
1H NMR (400 MHz, C6D6)
7.74 7.72 7.16 7.15 7.13 7.13 7.11 7.11 7.06 7.06 7.06 7.05 7.04 7.04 7.02 5.95 5.93 5.93 5.91 5.68 5.66 4.13 4.11 3.51 3.50 3.50 3.50 3.41 3.39 3.38 3.36 3.36 3.34 3.33 3.17 3.14 3.11 2.21 2.21 2.21 2.20 2.19 2.18 1.68 1.67 1.66 1.65 1.53 1.52 1.45 1.17 0.96 0.86 0.84 0.47 0.45 0.06 0.05
2.08 2.96 1.18 1.00 1.00 2.13 2.97 2.05 0.99 1.03 1.33 3.19 3.52 3.24 11.12 3.28 3.66 6.90
8.5 8.0 7.5 7.0 6.5 6.0 5.5 5.0 4.5 4.0 3.5 3.0 2.5 2.0 1.5 1.0 0.5 0.0 ppm

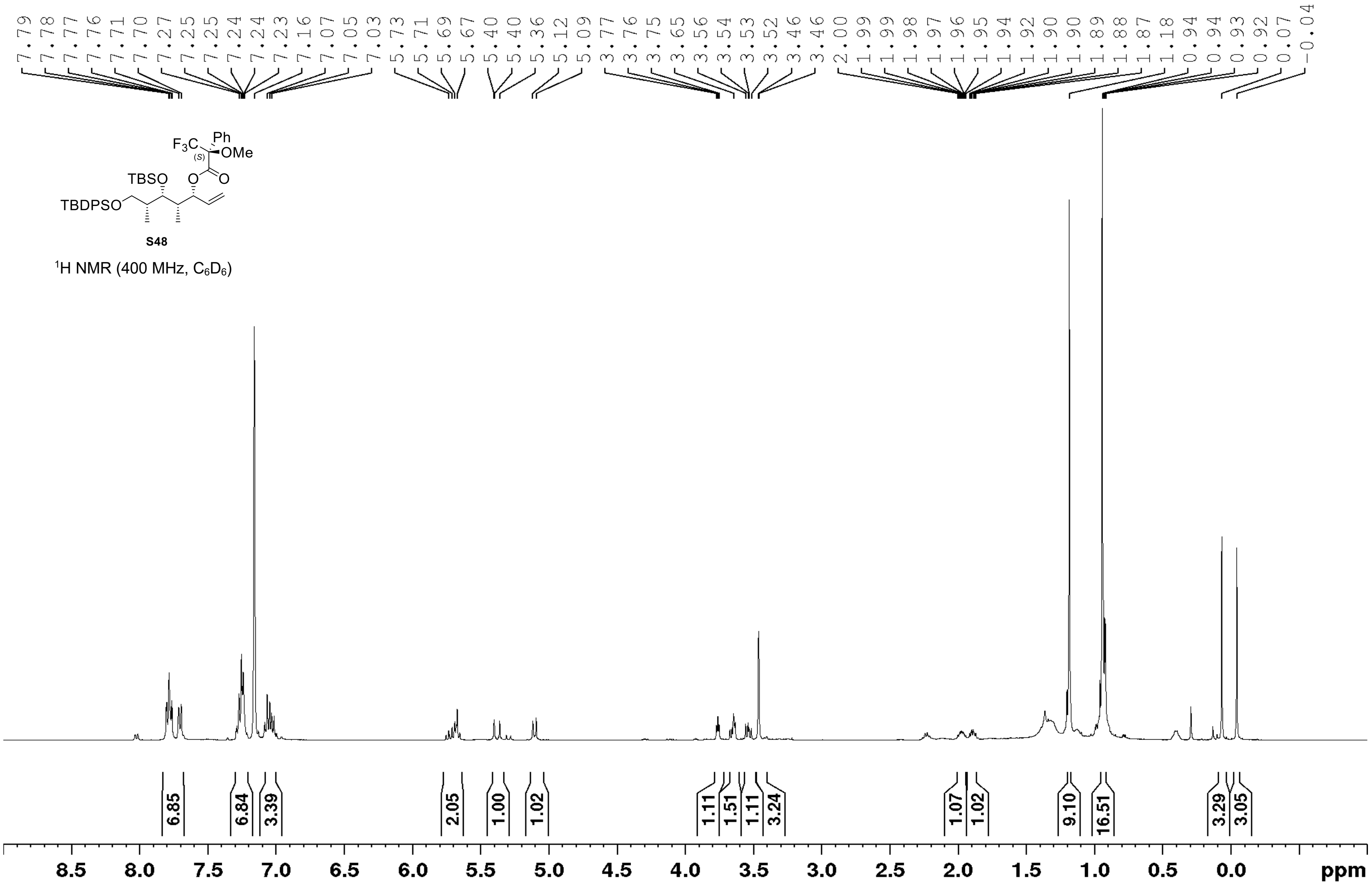
S48
¹H NMR (400 MHz, C6D6)
ppm

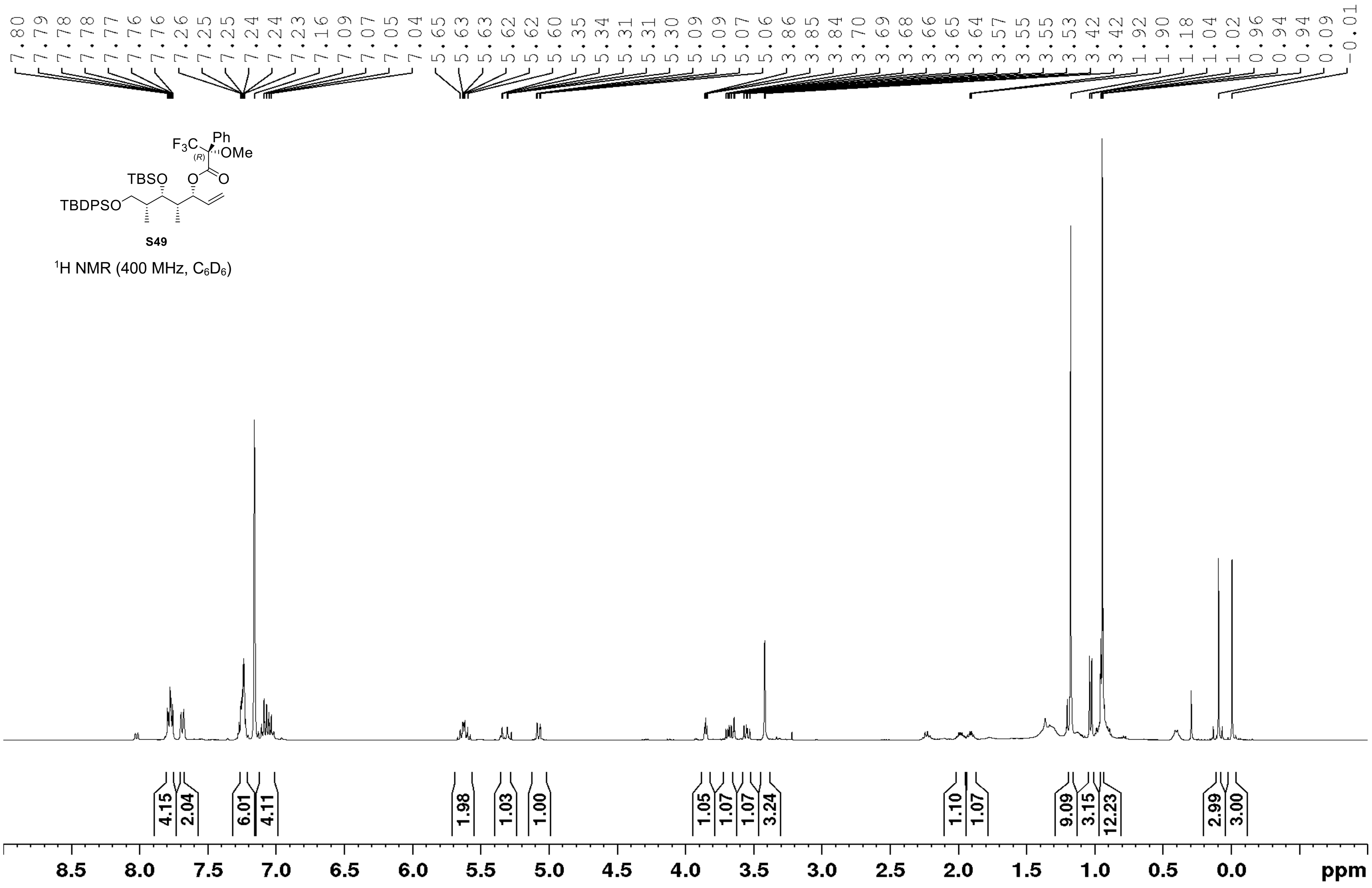

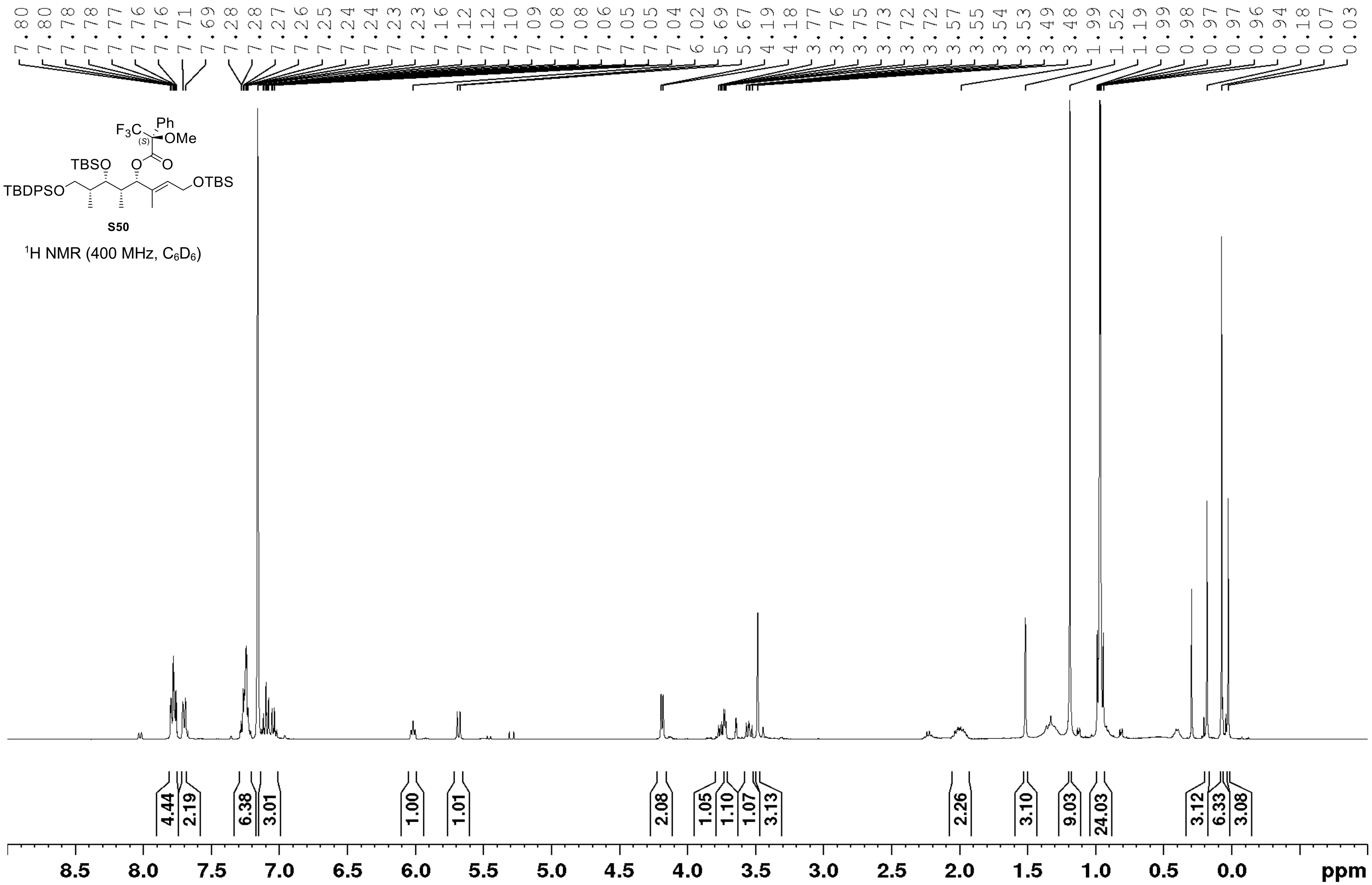

7.80 7.80 7.78 7.78 7.77 7.76 7.76 7.71 7.69 7.28 7.28 7.27 7.26 7.25 7.24 7.24 7.23 7.23 7.16 7.12 7.12 7.10 7.09 7.08 7.08 7.06 7.05 7.05 7.04 6.02 5.69 5.67 4.19 4.18 3.77 3.76 3.75 3.73 3.72 3.72 3.57 3.55 3.54 3.53 3.49 3.48 1.99 1.52 1.19 0.99 0.98 0.97 0.97 0.96 0.94 0.18 0.07 0.03
Ph
F_3C
(S)
OMe
TBSO
O
O
TBDPSO
OTBS
S50
^{1}H NMR (400 MHz, C_6D_6)
4.44 2.19 6.38 3.01 1.00 1.01 2.08 1.05 1.10 1.07 3.13 2.26 3.10 9.03 24.03 3.12 6.33 3.08
8.5 8.0 7.5 7.0 6.5 6.0 5.5 5.0 4.5 4.0 3.5 3.0 2.5 2.0 1.5 1.0 0.5 0.0 ppm

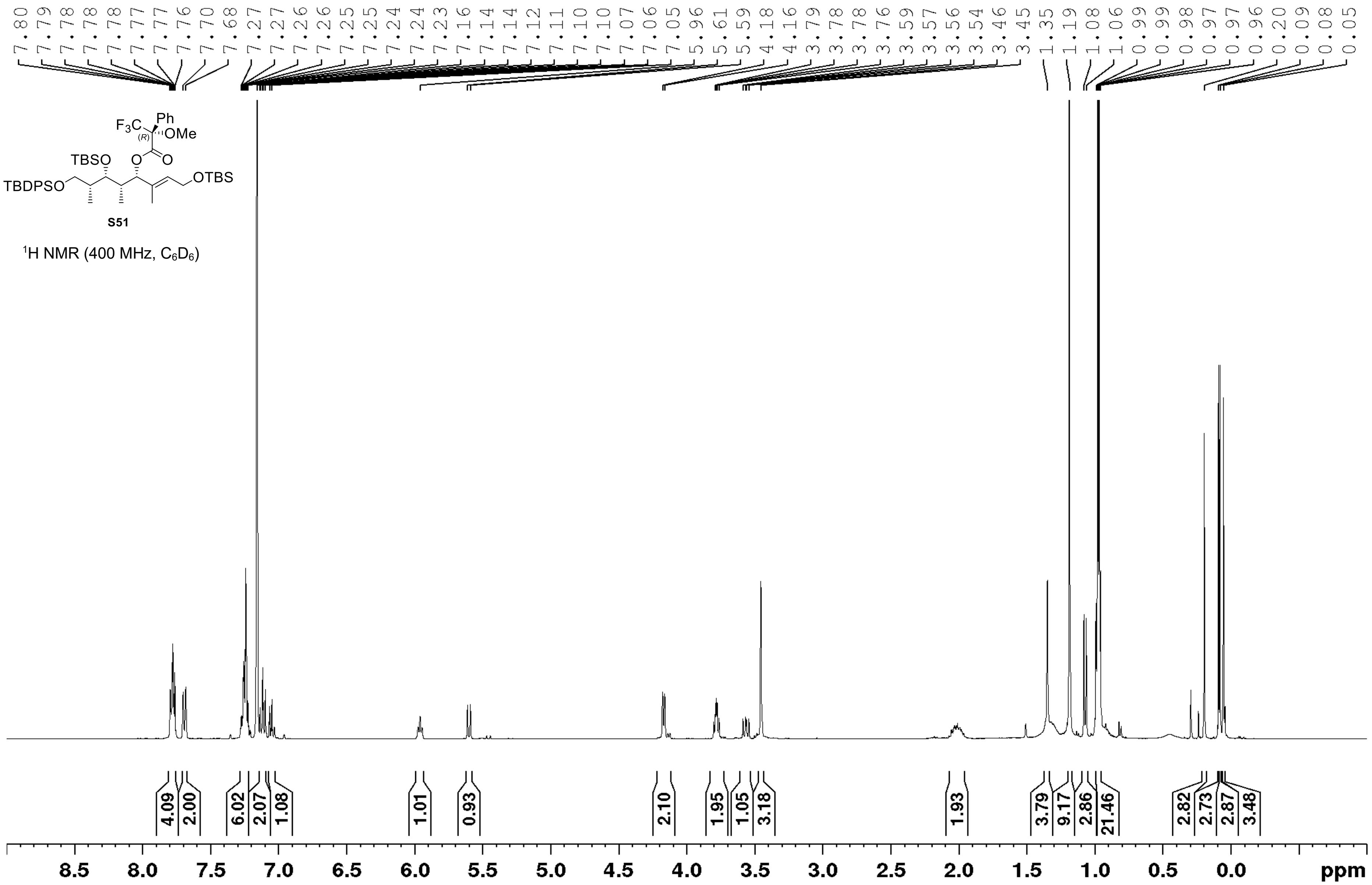

7.80 7.79 7.78 7.78 7.78 7.77 7.77 7.76 7.70 7.68 7.27 7.27 7.26 7.26 7.25 7.25 7.24 7.24 7.23 7.16 7.14 7.14 7.12 7.11 7.10 7.10 7.07 7.06 7.05 5.96 5.61 5.59 4.18 4.16 3.79 3.78 3.78 3.76 3.59 3.57 3.56 3.54 3.46 3.45 1.35 1.19 1.08 1.06 0.99 0.99 0.98 0.97 0.97 0.96 0.20 0.09 0.08 0.05
F_3C
Ph
(R)
OMe
TBSO
O
O
TBDPSO
OTBS
S51
^{1}H NMR (400 MHz, C_6D_6)
4.09 2.00 6.02 2.07 1.08 1.01 0.93 2.10 1.95 1.05 3.18 1.93 3.79 9.17 2.86 21.46 2.82 2.73 2.87 3.48
8.5 8.0 7.5 7.0 6.5 6.0 5.5 5.0 4.5 4.0 3.5 3.0 2.5 2.0 1.5 1.0 0.5 0.0 ppm

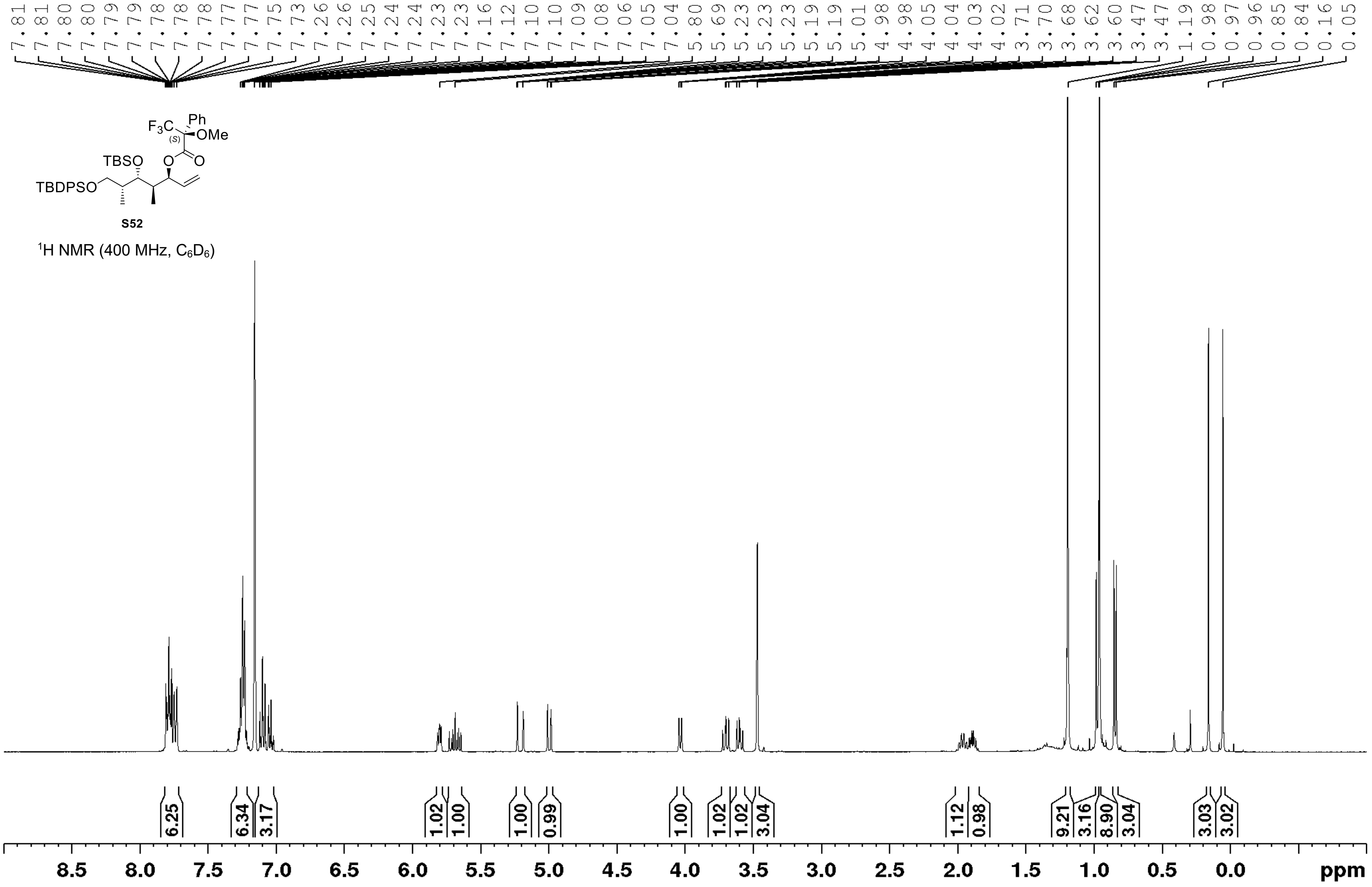
7.81 7.81 7.80 7.80 7.79 7.79 7.78 7.78 7.78 7.77 7.77 7.75 7.73 7.26 7.26 7.25 7.24 7.24 7.23 7.23 7.16 7.12 7.10 7.10 7.09 7.08 7.06 7.05 7.04 5.80 5.69 5.23 5.23 5.23 5.19 5.19 5.01 4.98 4.98 4.05 4.04 4.03 4.02 3.71 3.70 3.68 3.62 3.60 3.47 3.47 1.19 0.98 0.97 0.96 0.85 0.84 0.16 0.05
F3C
Ph
(S)
OMe
TBSO
O
O
TBDPSO
S52
1H NMR (400 MHz, C6D6)
6.25 6.34 3.17 1.02 1.00 1.00 0.99 1.00 1.02 1.02 3.04 1.12 0.98 9.21 3.16 8.90 3.04 3.03 3.02
8.5 8.0 7.5 7.0 6.5 6.0 5.5 5.0 4.5 4.0 3.5 3.0 2.5 2.0 1.5 1.0 0.5 0.0 ppm

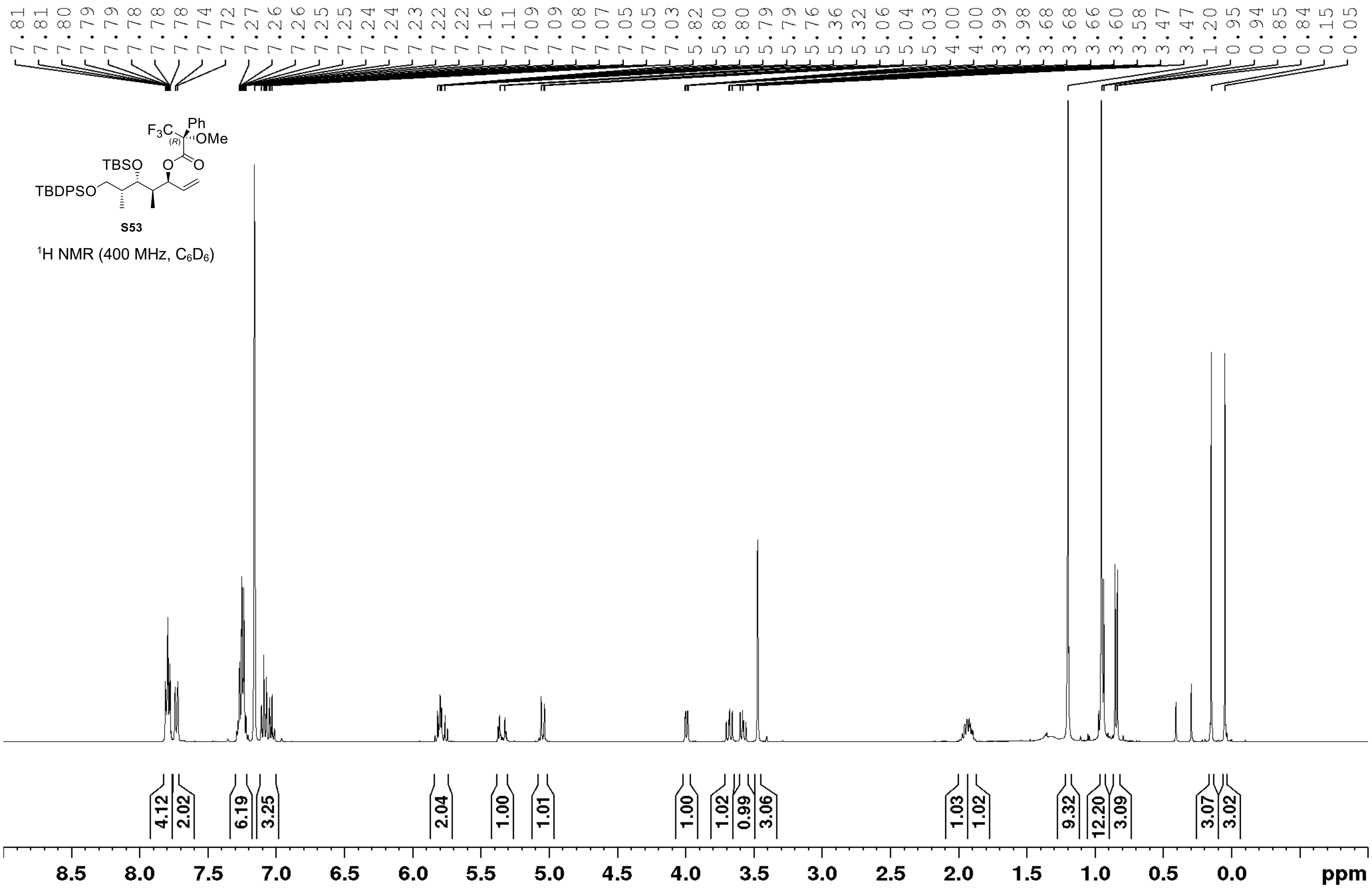
7.81 7.81 7.80 7.79 7.79 7.78 7.78 7.78 7.74 7.72 7.27 7.26 7.26 7.25 7.25 7.24 7.24 7.23 7.22 7.22 7.16 7.11 7.09 7.09 7.08 7.07 7.05 7.05 7.03 5.82 5.80 5.80 5.79 5.79 5.76 5.36 5.32 5.06 5.04 5.03 4.00 4.00 3.99 3.98 3.68 3.68 3.66 3.60 3.58 3.47 3.47 1.20 0.95 0.94 0.85 0.84 0.15 0.05
F3C
Ph
(R)
OMe
TBSO
O
O
TBDPSO
S53
1H NMR (400 MHz, C6D6)
4.12 2.02 6.19 3.25 2.04 1.00 1.01 1.00 1.02 0.99 3.06 1.03 1.02 9.32 12.20 3.09 3.07 3.02
8.5 8.0 7.5 7.0 6.5 6.0 5.5 5.0 4.5 4.0 3.5 3.0 2.5 2.0 1.5 1.0 0.5 0.0 ppm

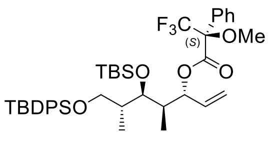

S54

^1H NMR (400 MHz, C_6D_6)

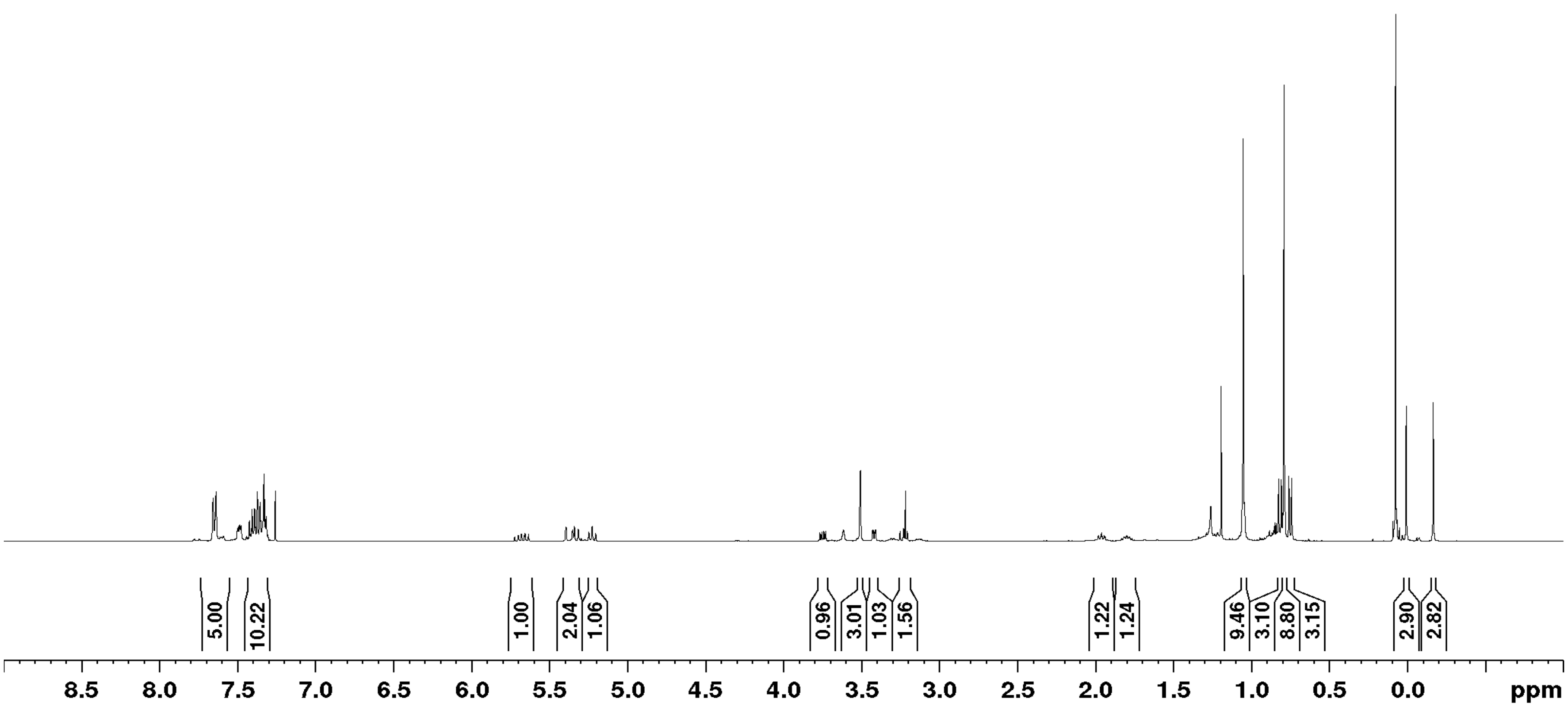

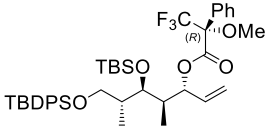

S55

^{1}H NMR (400 MHz, C_6D_6)

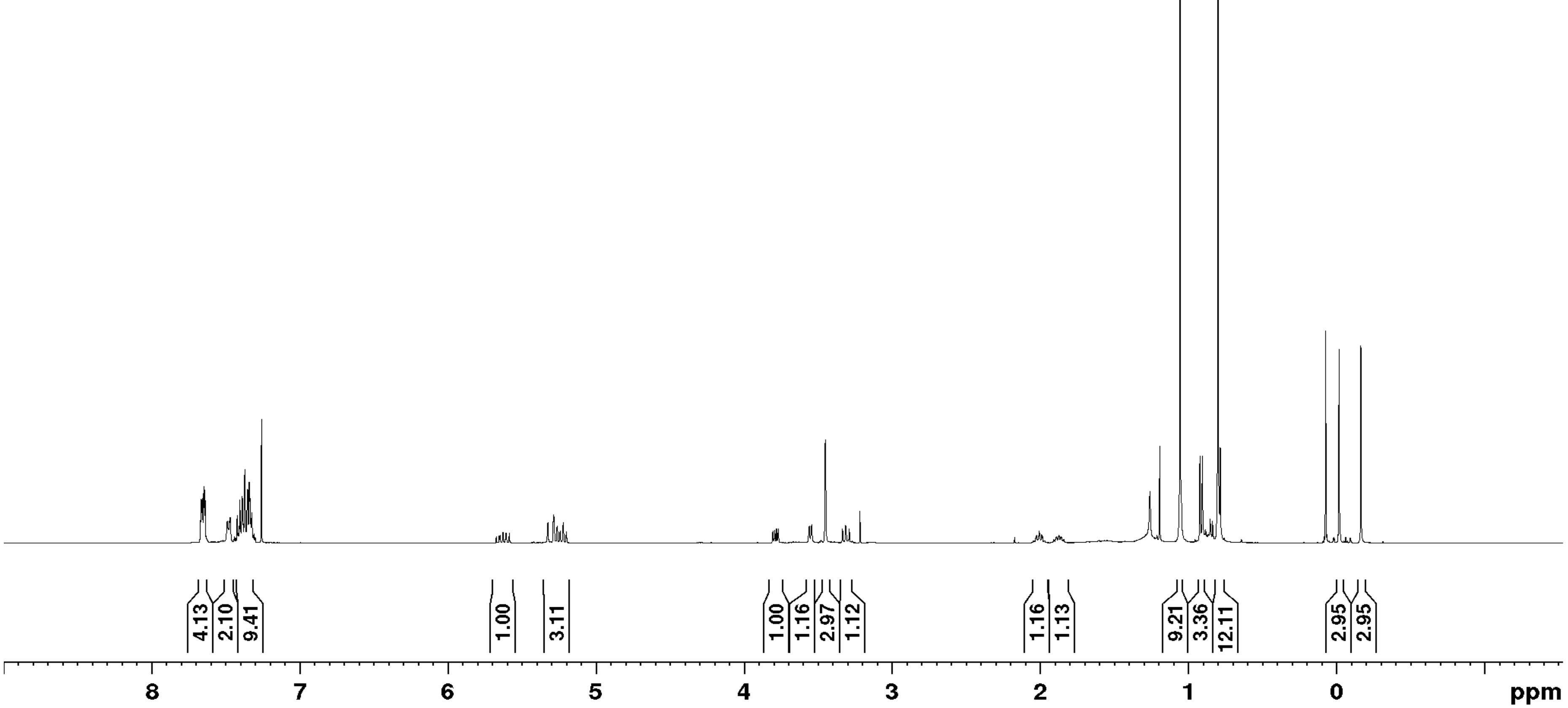

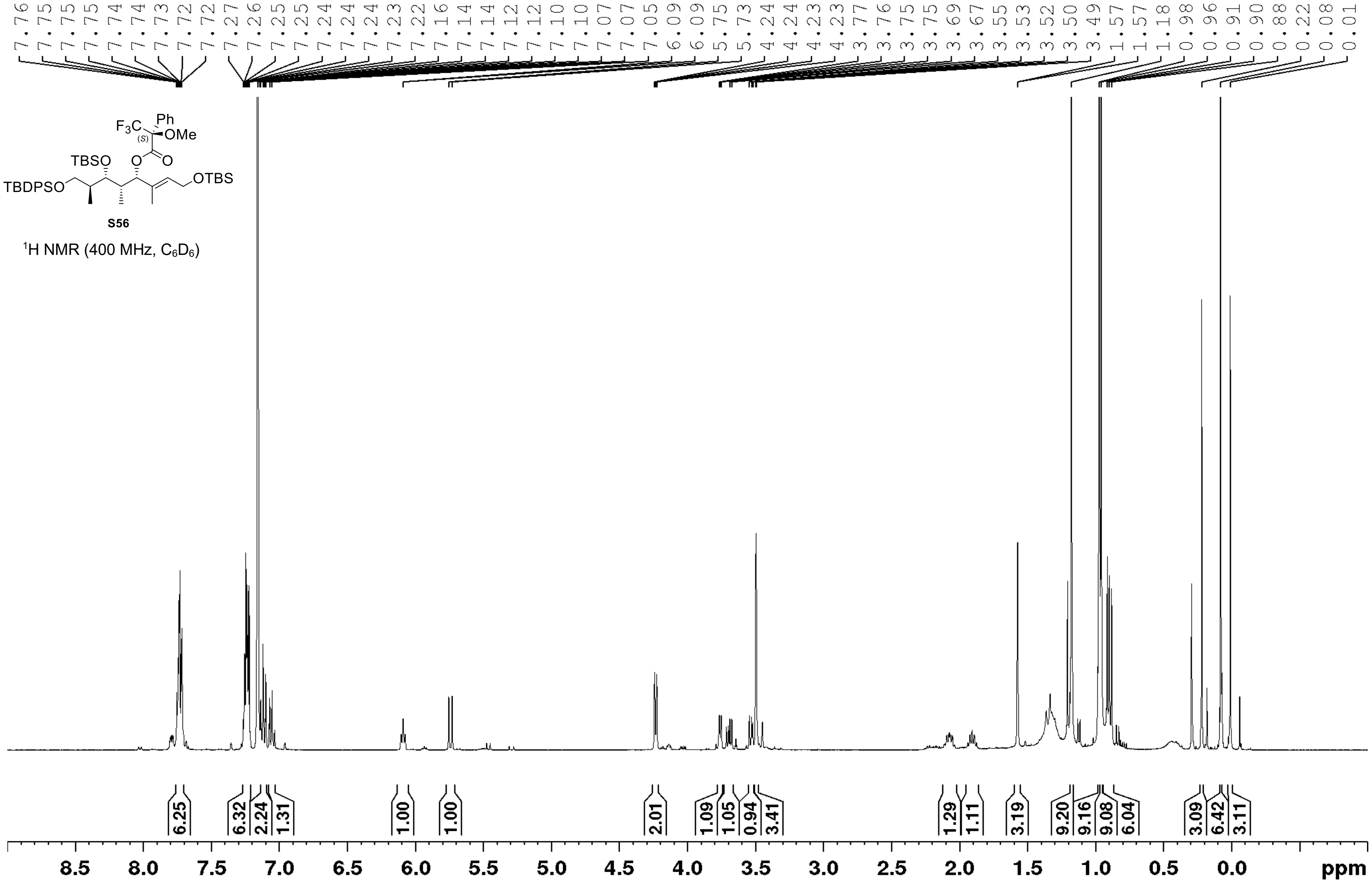

S56

^{1}H NMR (400 MHz, C_6D_6)

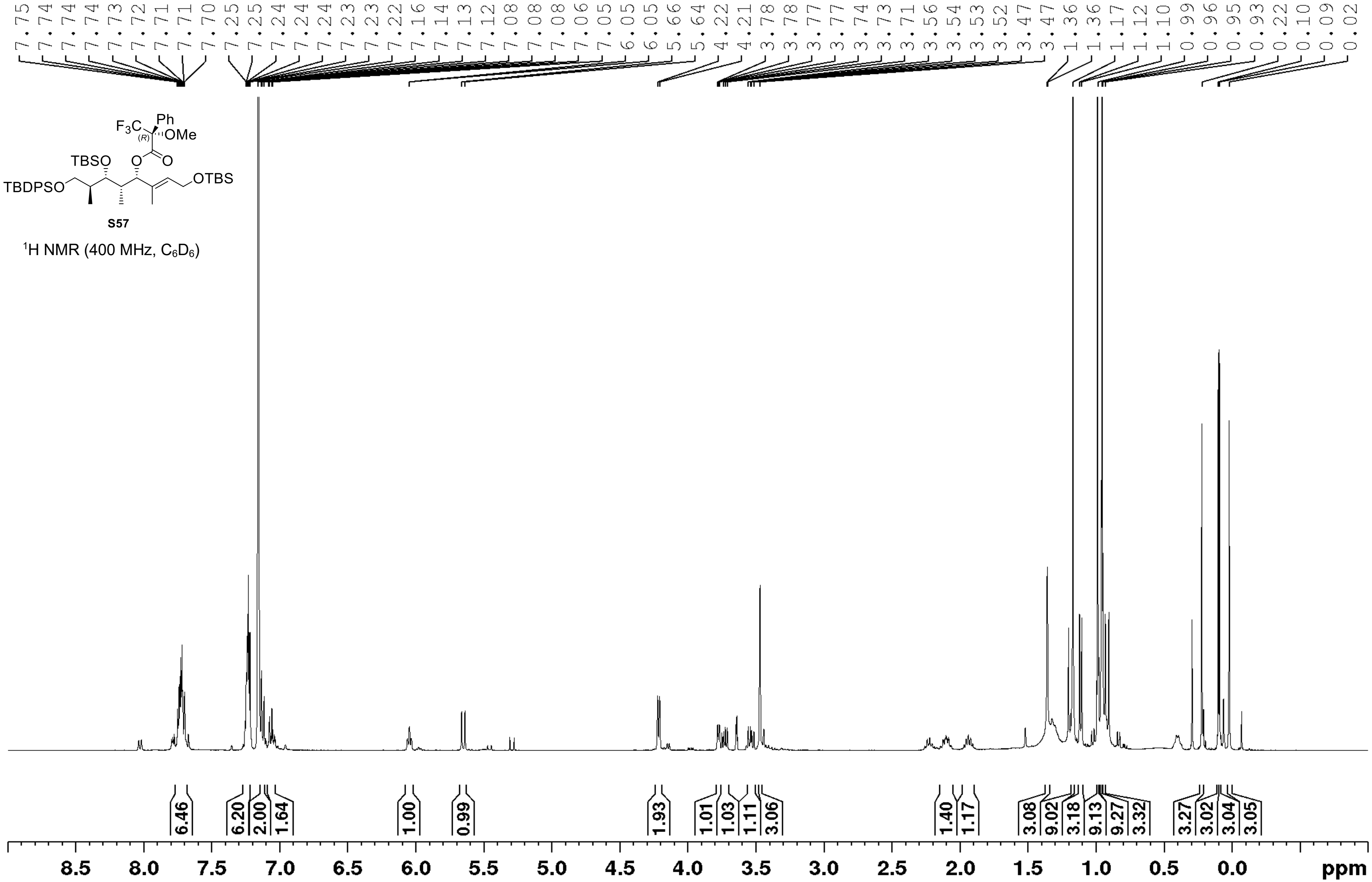

S57

^1H NMR (400 MHz, C_6D_6)

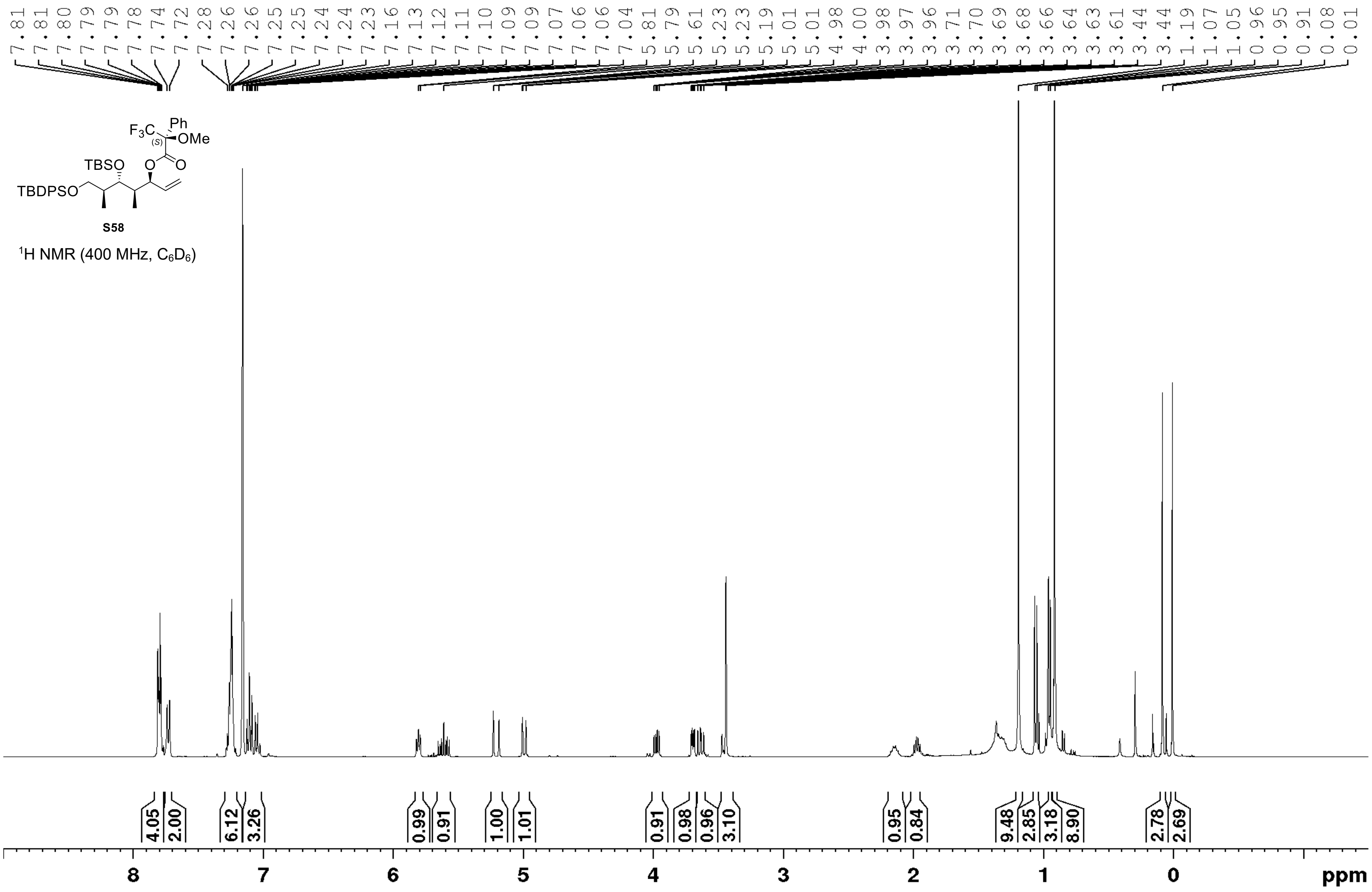
S58
¹H NMR (400 MHz, C₆D₆)
7.81 7.81 7.80 7.79 7.79 7.78 7.74 7.72 7.28 7.26 7.26 7.25 7.25 7.24 7.24 7.23 7.16 7.13 7.12 7.11 7.10 7.09 7.09 7.07 7.06 7.06 7.04 5.81 5.79 5.61 5.23 5.23 5.19 5.01 5.01 4.98 4.00 3.98 3.97 3.96 3.71 3.70 3.69 3.68 3.66 3.64 3.63 3.61 3.44 3.44 1.19 1.07 1.05 0.96 0.95 0.91 0.08 0.01
4.05 2.00 6.12 3.26 0.99 0.91 1.00 1.01 0.91 0.98 0.96 3.10 0.95 0.84 9.48 2.85 3.18 8.90 2.78 2.69
8 7 6 5 4 3 2 1 0 ppm

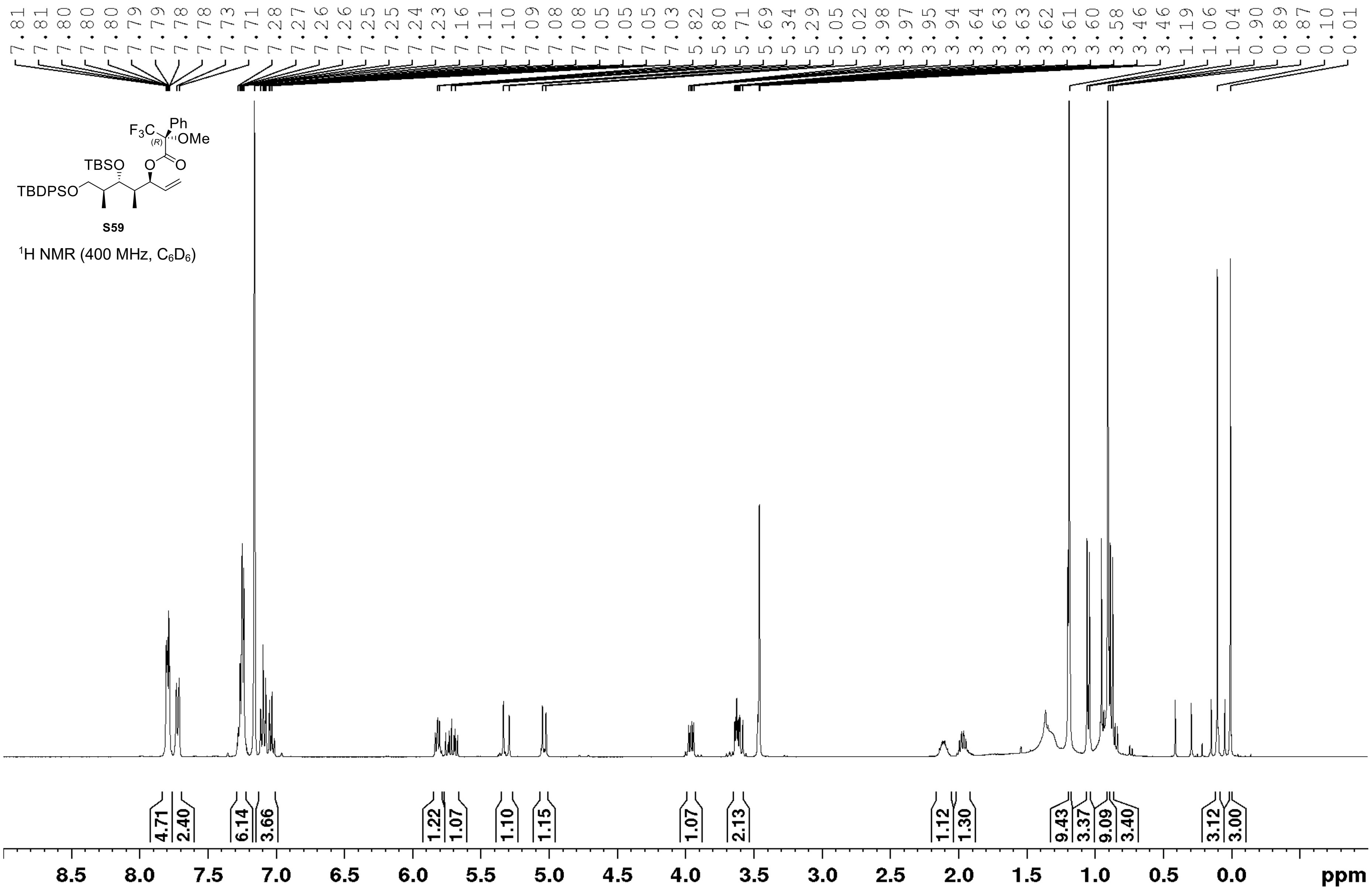
7.81 7.81 7.80 7.80 7.80 7.79 7.79 7.78 7.78 7.73 7.71 7.28 7.27 7.26 7.26 7.25 7.25 7.24 7.23 7.16 7.11 7.10 7.09 7.08 7.08 7.05 7.05 7.05 7.03 5.82 5.80 5.71 5.69 5.34 5.29 5.05 5.02 3.98 3.97 3.95 3.94 3.64 3.63 3.63 3.62 3.61 3.60 3.58 3.46 3.46 1.19 1.06 1.04 0.90 0.89 0.87 0.10 0.01
F_3C
(R)
Ph
OMe
TBSO
O
O
TBDPSO
S59
^1H NMR (400 MHz, C_6D_6)
4.71 2.40 6.14 3.66 1.22 1.07 1.10 1.15 1.07 2.13 1.12 1.30 9.43 3.37 9.09 3.40 3.12 3.00
8.5 8.0 7.5 7.0 6.5 6.0 5.5 5.0 4.5 4.0 3.5 3.0 2.5 2.0 1.5 1.0 0.5 0.0
ppm

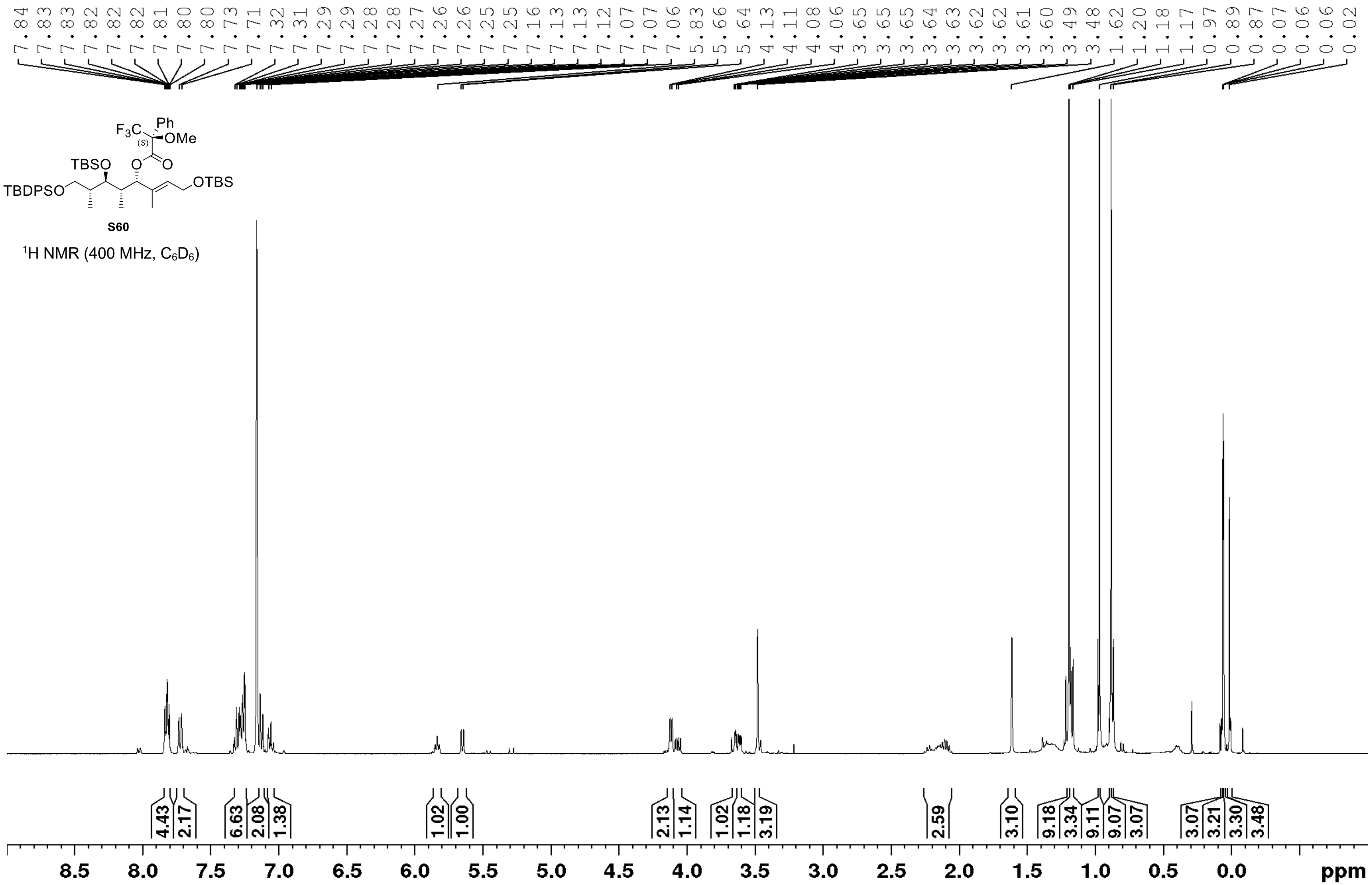

7.84 7.83 7.83 7.82 7.82 7.82 7.81 7.80 7.80 7.73 7.71 7.32 7.31 7.29 7.29 7.28 7.28 7.27 7.26 7.26 7.25 7.25 7.16 7.13 7.13 7.12 7.07 7.07 7.06 5.83 5.66 5.64 4.13 4.11 4.08 4.06 3.65 3.65 3.65 3.64 3.63 3.62 3.62 3.61 3.60 3.49 3.48 1.62 1.20 1.18 1.17 0.97 0.89 0.87 0.07 0.06 0.06 0.02
F3C
Ph
OMe
(S)
O
O
TBSO
TBDPSO
OTBS
S60
1H NMR (400 MHz, C6D6)
4.43 2.17 6.63 2.08 1.38 1.02 1.00 2.13 1.14 1.02 1.18 3.19 2.59 3.10 9.18 3.34 9.11 9.07 3.07 3.07 3.21 3.30 3.48
8.5 8.0 7.5 7.0 6.5 6.0 5.5 5.0 4.5 4.0 3.5 3.0 2.5 2.0 1.5 1.0 0.5 0.0 ppm

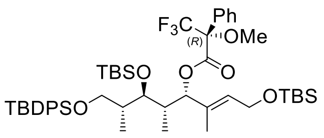

S61

^{1}H NMR (400 MHz, C_6D_6)

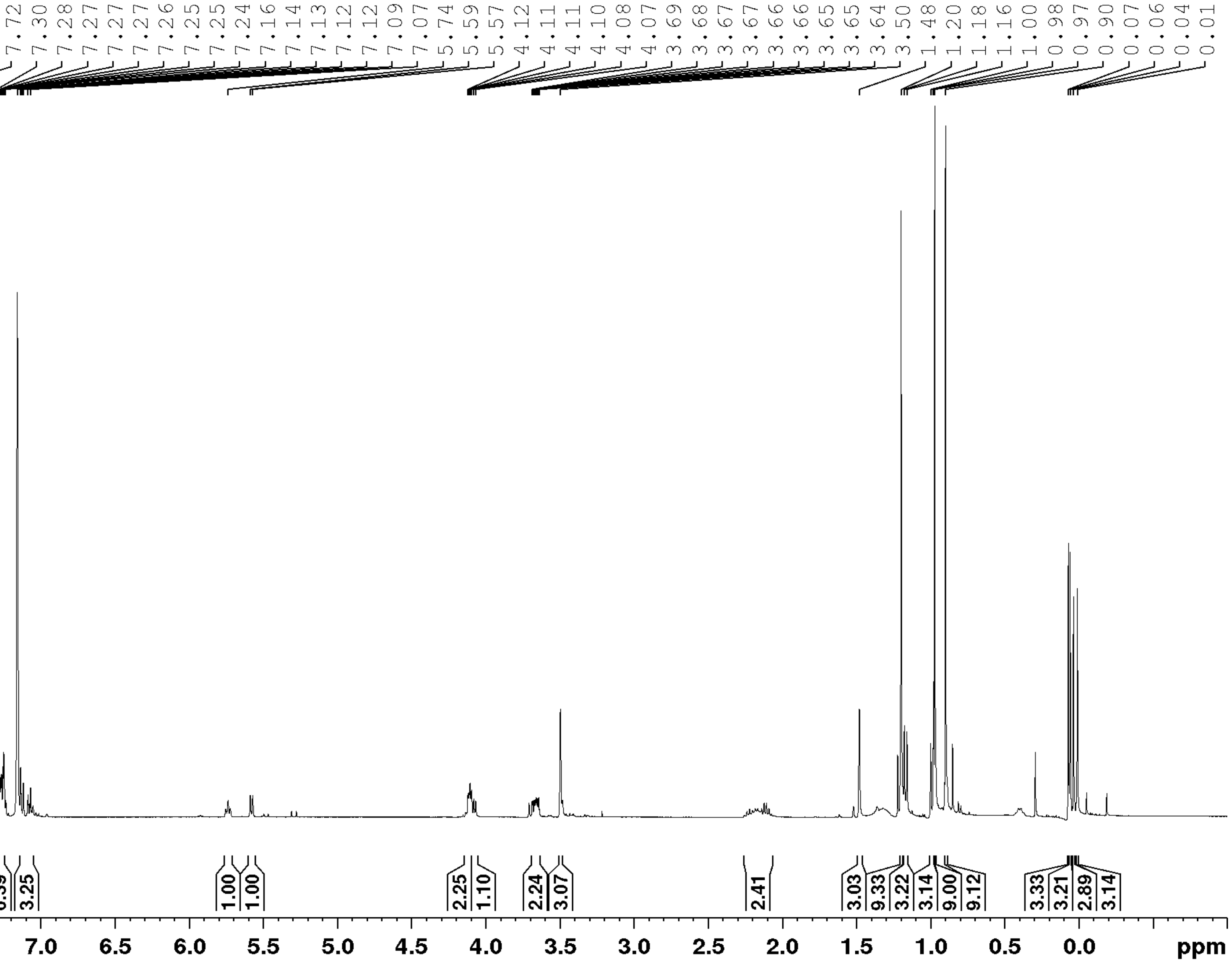

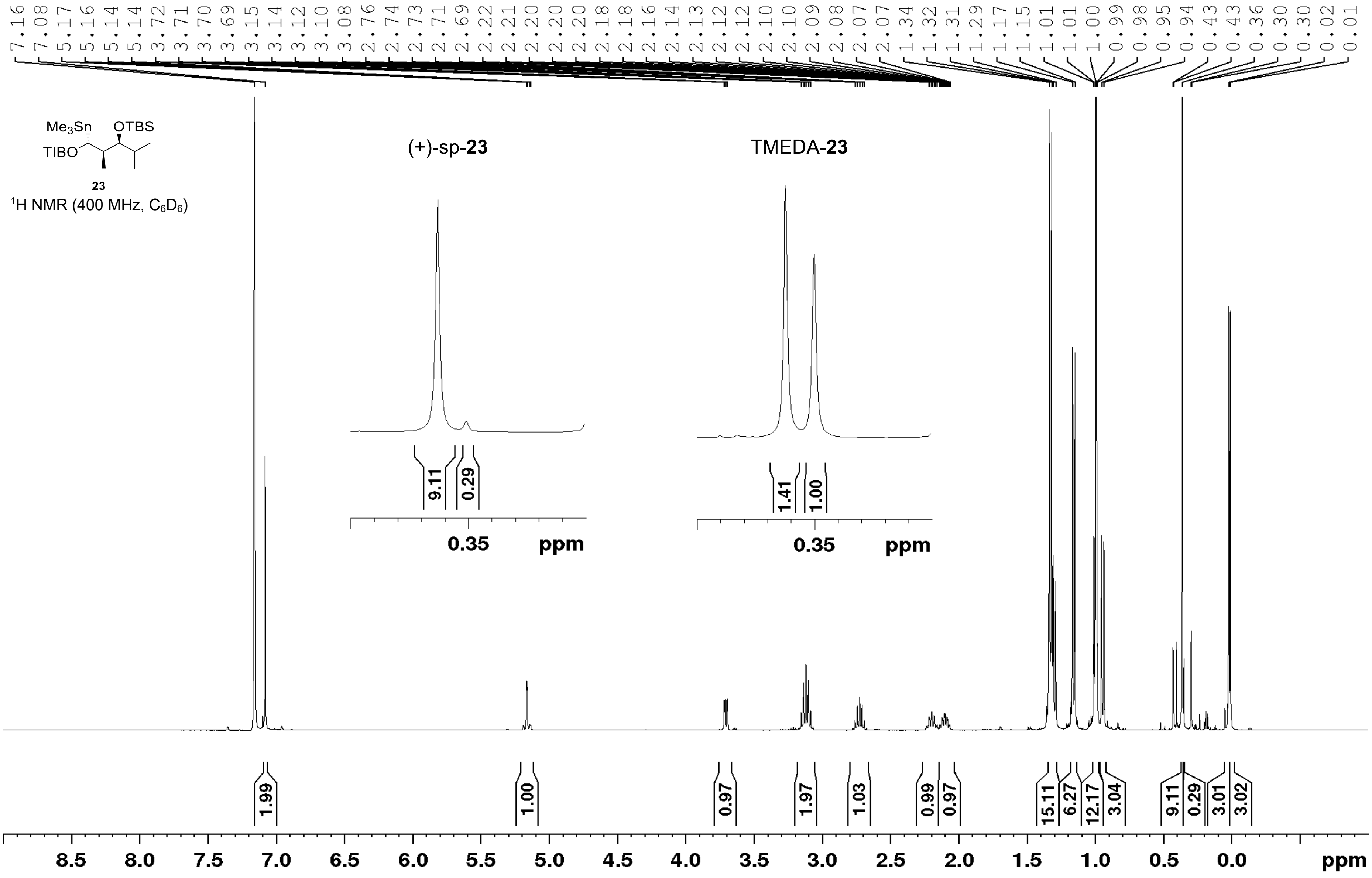
Me3Sn OTBS
TIBO
23
1H NMR (400 MHz, C6D6)
(+)-sp-23
TMEDA-23
ppm

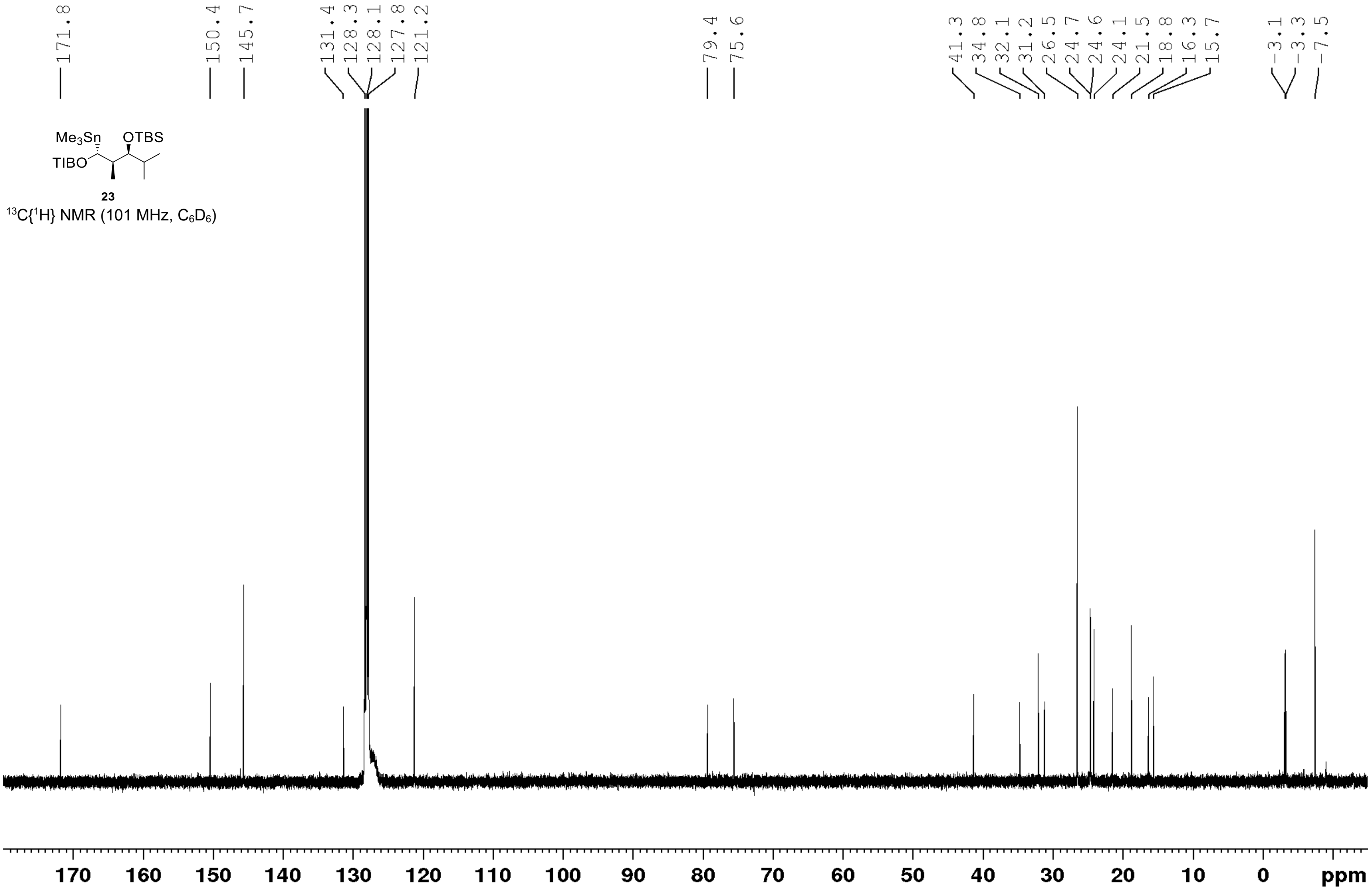
171.8
150.4
145.7
131.4
128.3
128.1
127.8
121.2
79.4
75.6
41.3
34.8
32.1
31.2
26.5
24.7
24.6
24.1
21.5
18.8
16.3
15.7
-3.1
-3.3
-7.5
Me3Sn
OTBS
TIBO
23
13C{1H} NMR (101 MHz, C6D6)
170
160
150
140
130
120
110
100
90
80
70
60
50
40
30
20
10
0
ppm

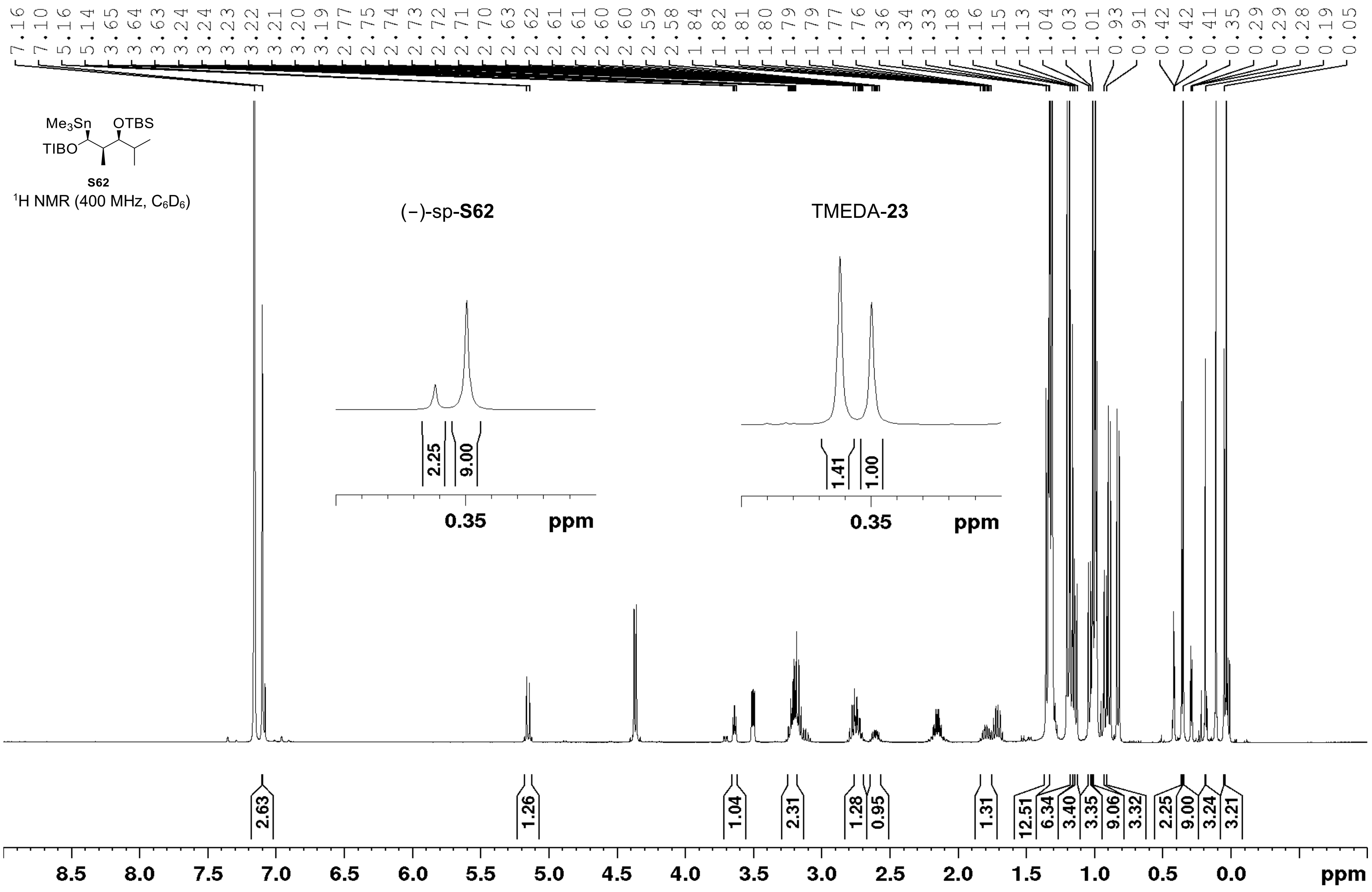

Me3Sn OTBS
TIBO
S62
1H NMR (400 MHz, C6D6)
(−)-sp-S62
TMEDA-23
2.25
9.00
0.35
ppm
1.41
1.00
7.16 7.10 5.16 5.14 3.65 3.64 3.63 3.24 3.24 3.23 3.22 3.21 3.20 3.19 2.77 2.75 2.74 2.73 2.72 2.71 2.70 2.63 2.62 2.61 2.61 2.60 2.60 2.59 2.58 1.84 1.82 1.81 1.80 1.79 1.79 1.77 1.76 1.36 1.34 1.33 1.18 1.16 1.15 1.13 1.04 1.03 1.01 0.93 0.91 0.42 0.42 0.41 0.35 0.29 0.29 0.28 0.19 0.05
2.63 1.26 1.04 2.31 1.28 0.95 1.31 12.51 6.34 3.40 3.35 9.06 3.32 2.25 9.00 3.24 3.21
8.5 8.0 7.5 7.0 6.5 6.0 5.5 5.0 4.5 4.0 3.5 3.0 2.5 2.0 1.5 1.0 0.5 0.0 ppm

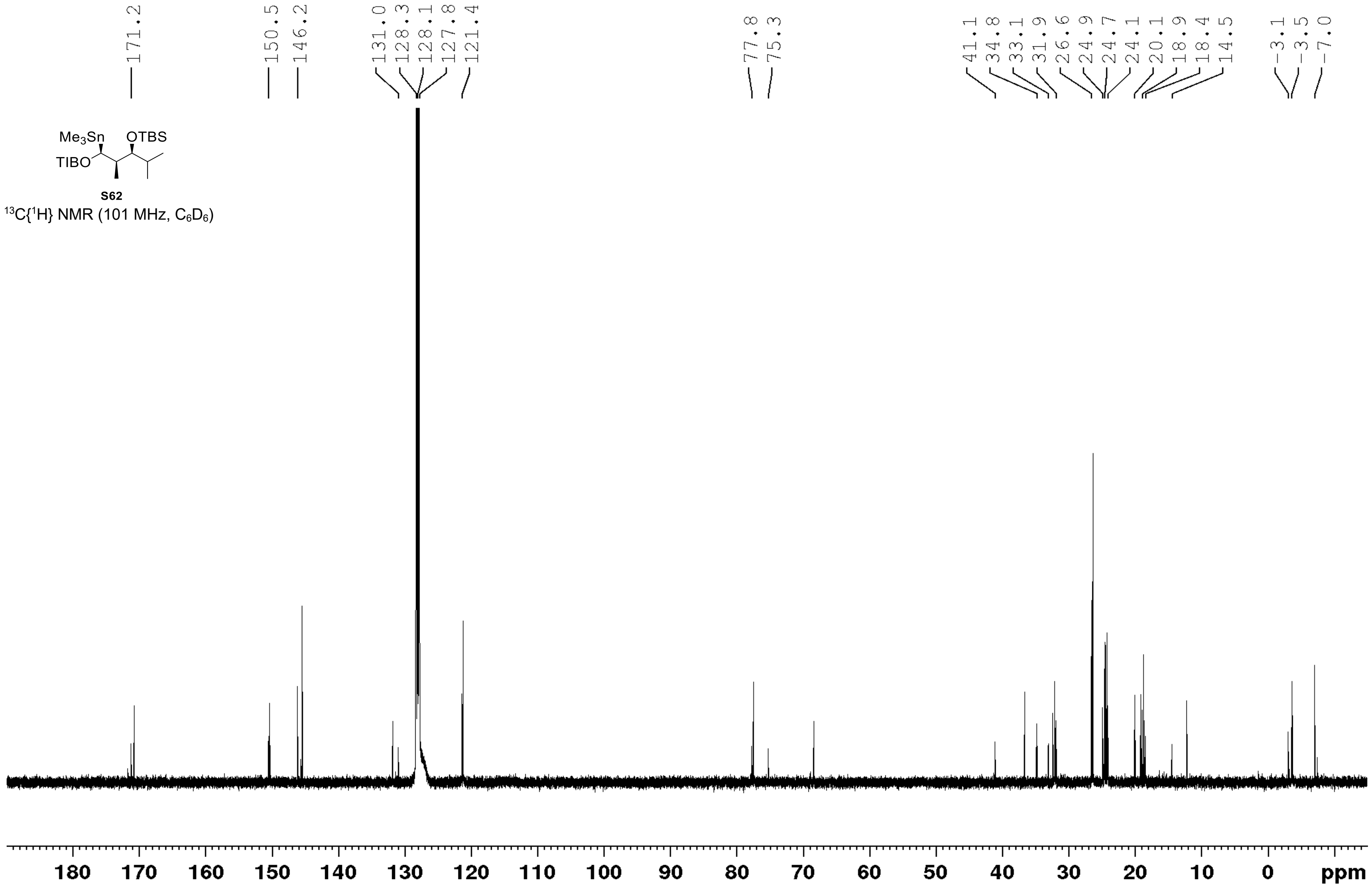
171.2
150.5
146.2
131.0
128.3
128.1
127.8
121.4
77.8
75.3
41.1
34.8
33.1
31.9
26.6
24.9
24.7
24.1
20.1
18.9
18.4
14.5
-3.1
-3.5
-7.0
Me3Sn
OTBS
TIBO
S62
13C{1H} NMR (101 MHz, C6D6)
180
170
160
150
140
130
120
110
100
90
80
70
60
50
40
30
20
10
0
ppm

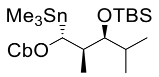

S63

^1H NMR (400 MHz, C_6D_6)

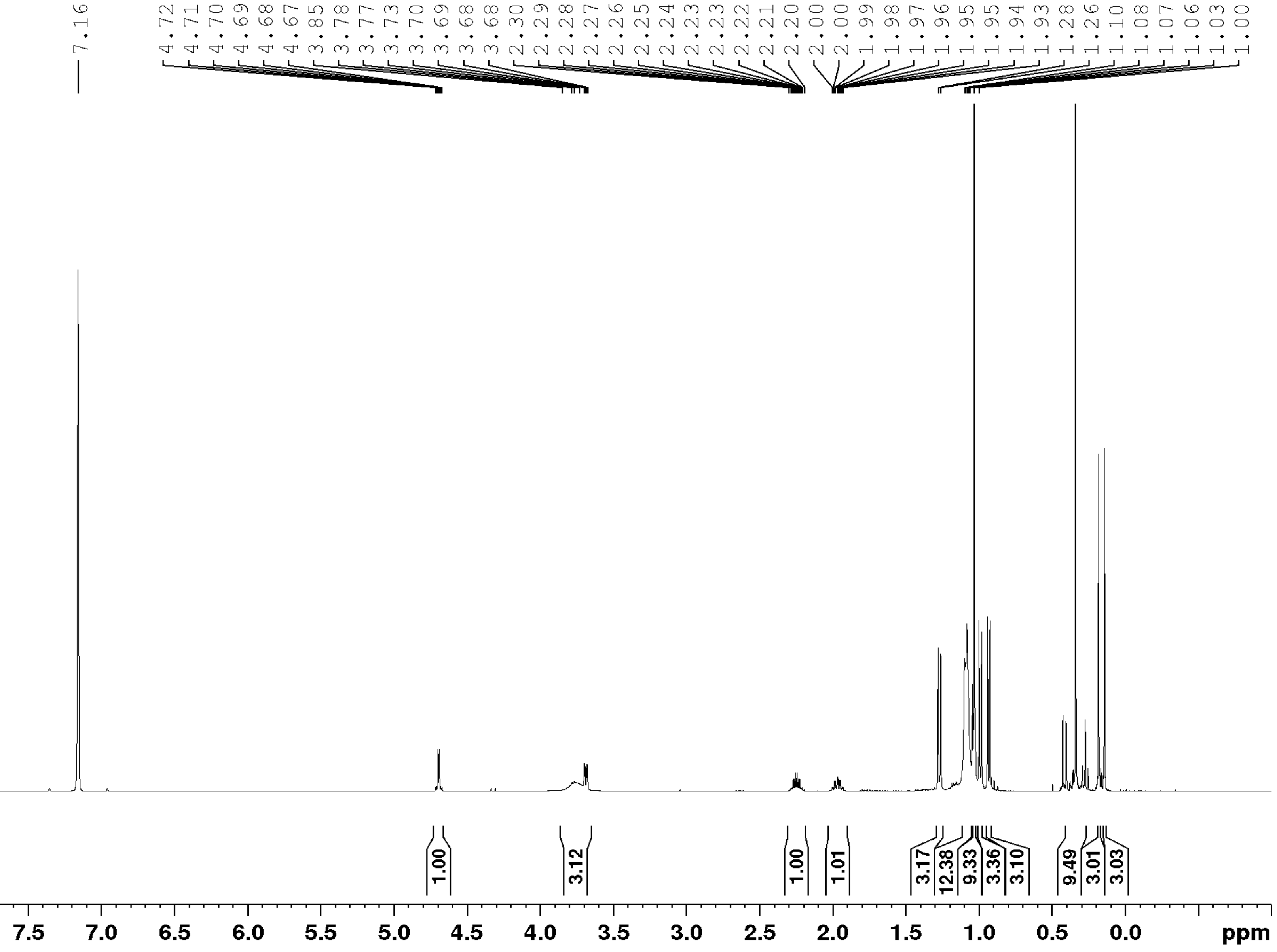

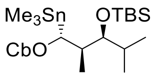

S63

$^{13}C\{^1H\}$ NMR (101 MHz, C_6D_6)

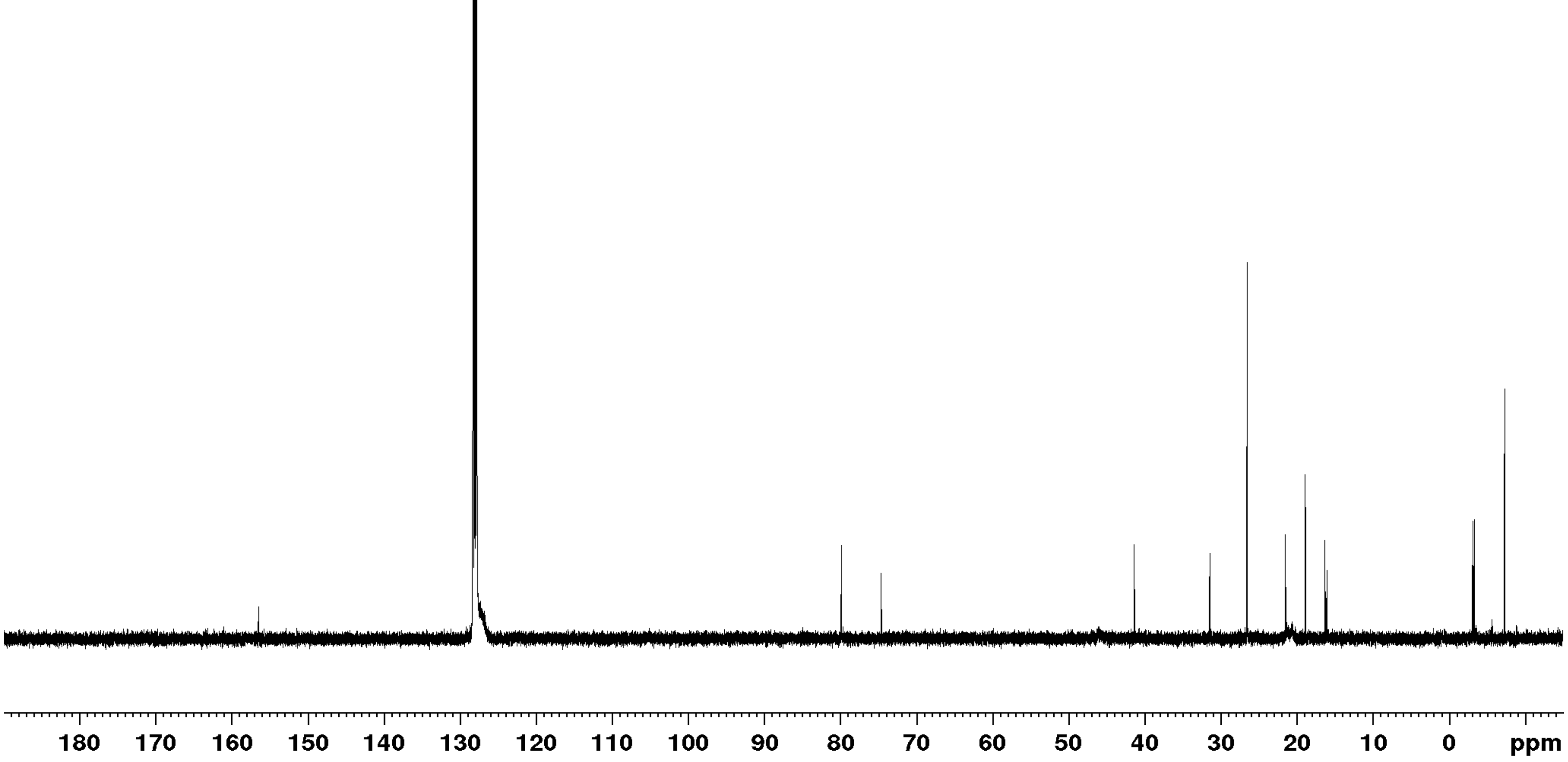

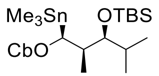

24

^{1}H NMR (400 MHz, C_6D_6)

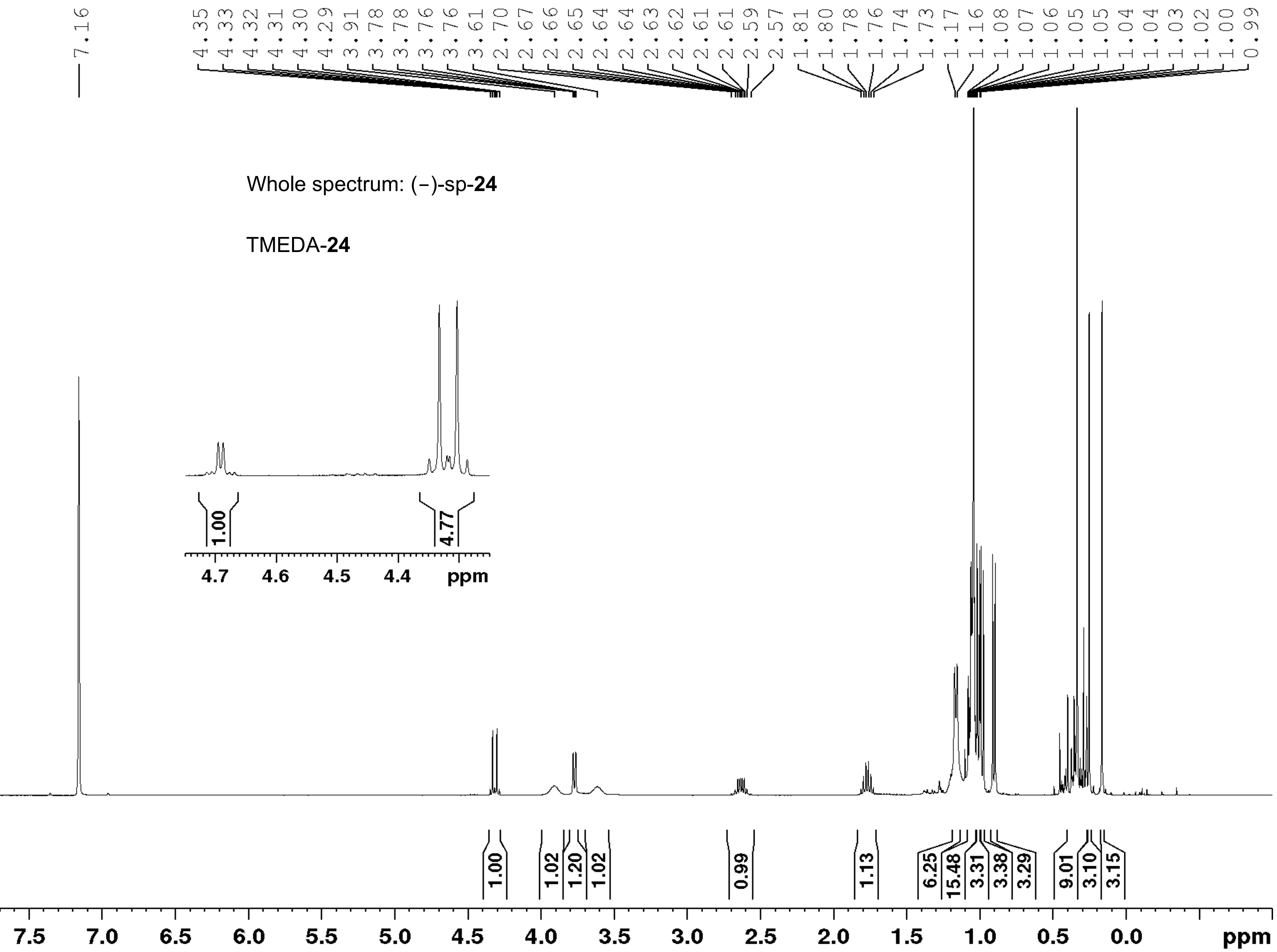

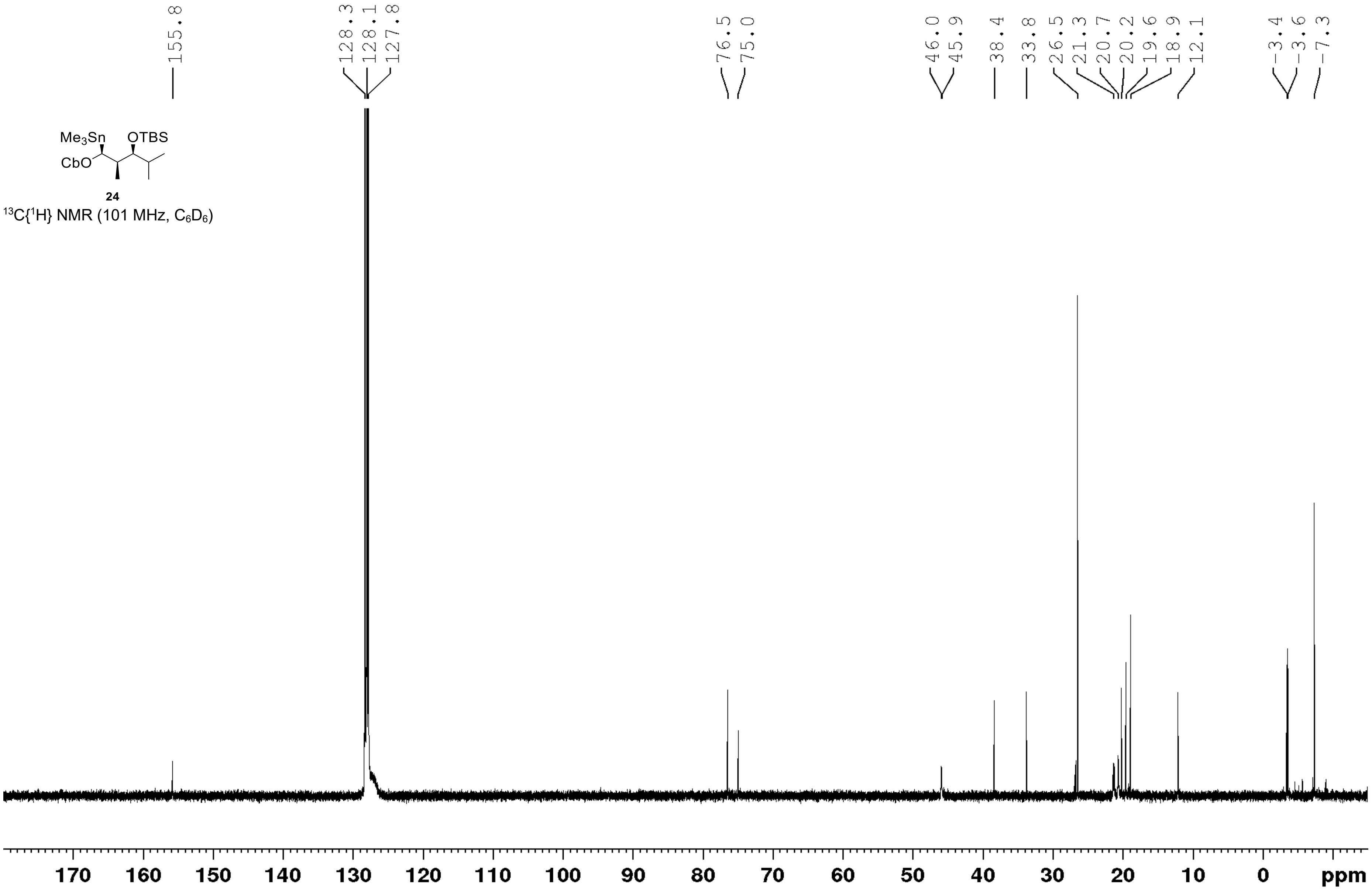
155.8
128.3
128.1
127.8
76.5
75.0
46.0
45.9
38.4
33.8
26.5
21.3
20.7
20.2
19.6
18.9
12.1
-3.4
-3.6
-7.3
Me3Sn
OTBS
CbO
24
13C{1H} NMR (101 MHz, C6D6)
170
160
150
140
130
120
110
100
90
80
70
60
50
40
30
20
10
0
ppm

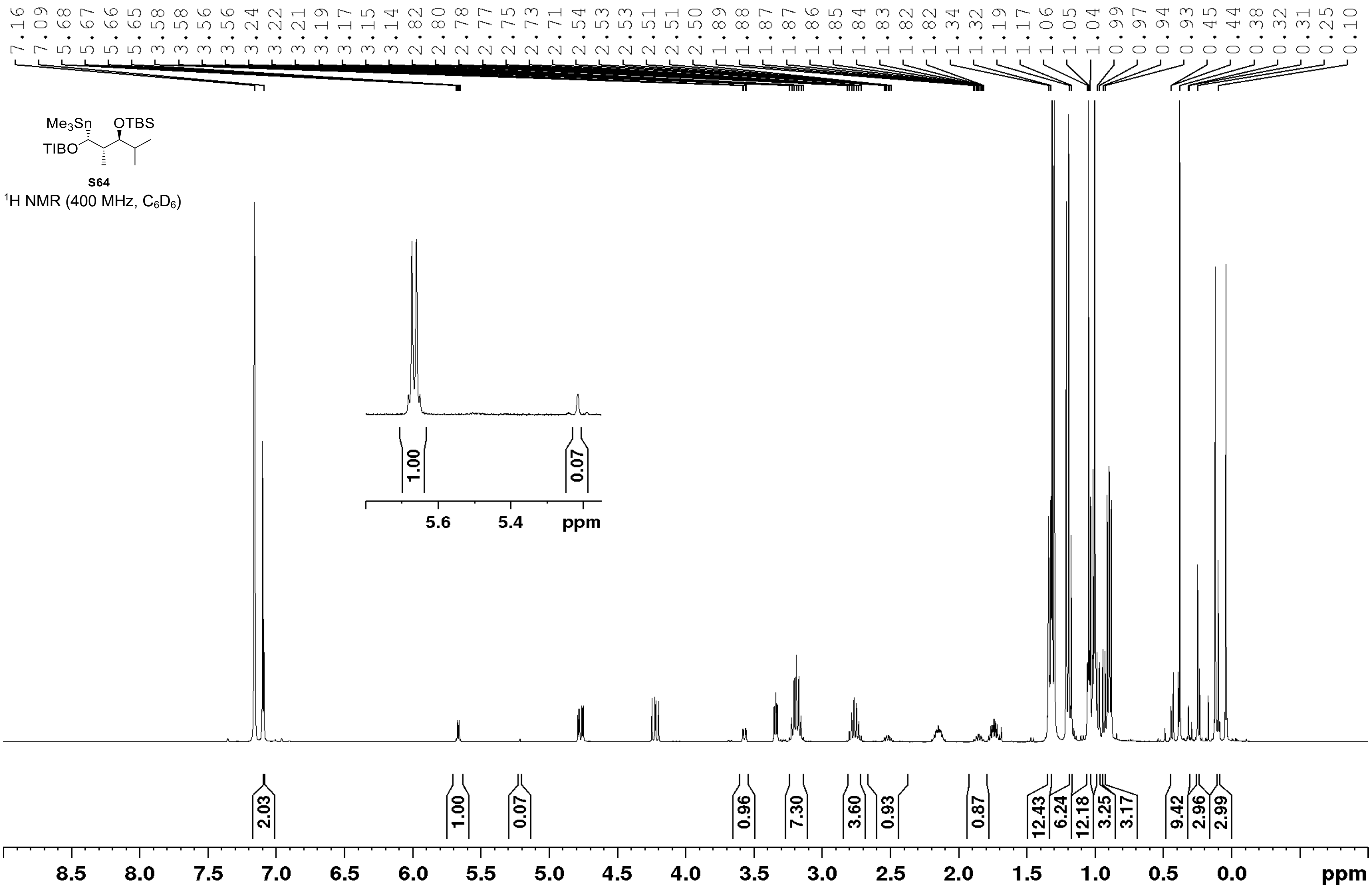
7.16 7.09 5.68 5.67 5.66 5.65 3.58 3.58 3.56 3.56 3.24 3.22 3.21 3.19 3.17 3.15 3.14 2.82 2.80 2.78 2.77 2.75 2.73 2.71 2.54 2.53 2.53 2.51 2.51 2.50 1.89 1.88 1.87 1.87 1.86 1.85 1.84 1.83 1.82 1.82 1.34 1.32 1.19 1.17 1.06 1.05 1.04 0.99 0.97 0.94 0.93 0.45 0.44 0.38 0.32 0.31 0.25 0.10
Me3Sn
OTBS
TIBO
S64
1H NMR (400 MHz, C6D6)
1.00
0.07
5.6
5.4
ppm
2.03
1.00
0.07
0.96
7.30
3.60
0.93
0.87
12.43
6.24
12.18
3.25
3.17
9.42
2.96
2.99
8.5 8.0 7.5 7.0 6.5 6.0 5.5 5.0 4.5 4.0 3.5 3.0 2.5 2.0 1.5 1.0 0.5 0.0
ppm

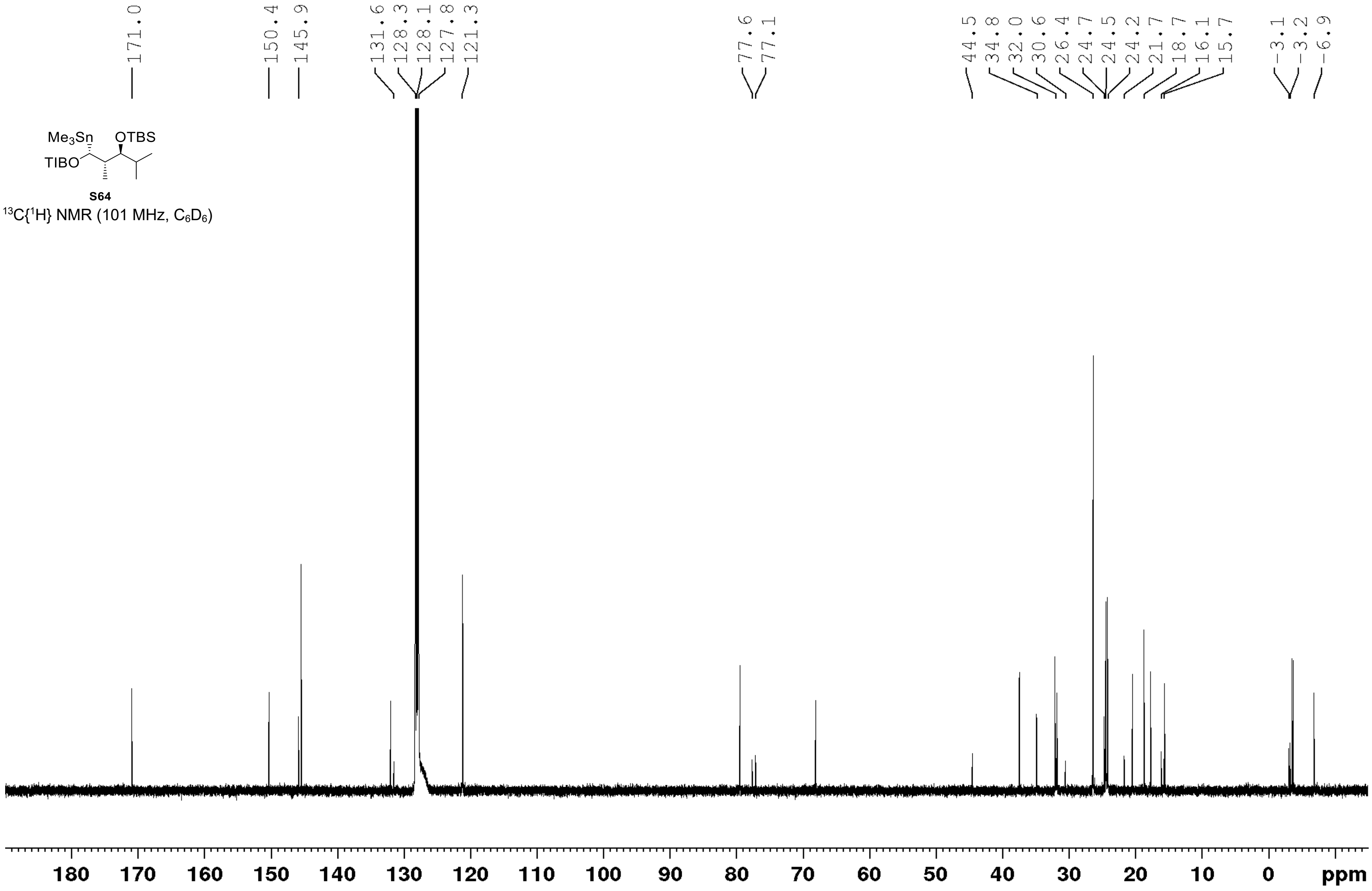
171.0
150.4
145.9
131.6
128.3
128.1
127.8
121.3
77.6
77.1
44.5
34.8
32.0
30.6
26.4
24.7
24.5
24.2
21.7
18.7
16.1
15.7
-3.1
-3.2
-6.9
Me3Sn
OTBS
TIBO
S64
13C{1H} NMR (101 MHz, C6D6)
180 170 160 150 140 130 120 110 100 90 80 70 60 50 40 30 20 10 0 ppm

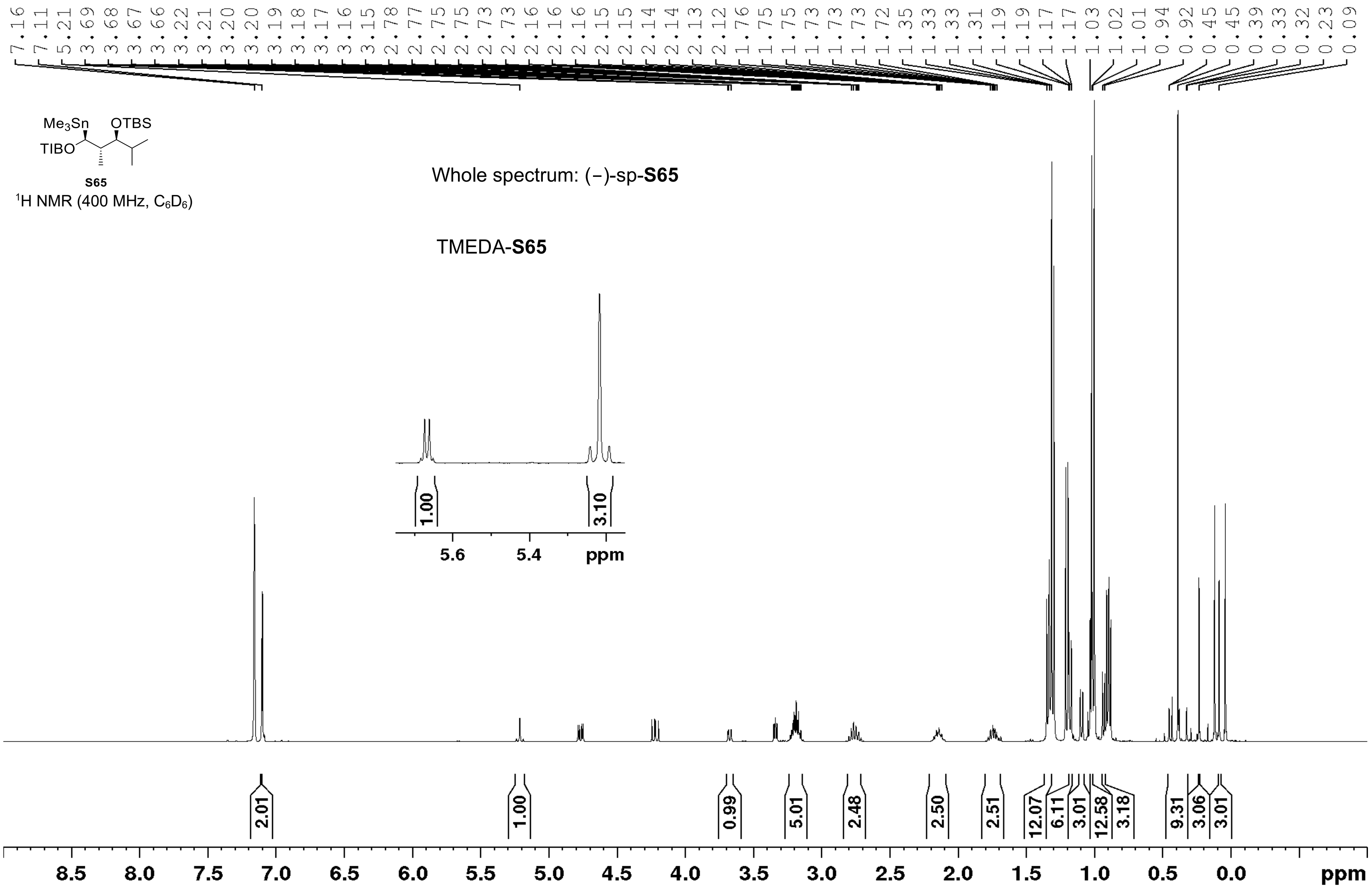

Me3Sn OTBS
TIBO
S65
1H NMR (400 MHz, C6D6)
Whole spectrum: (−)-sp-S65
TMEDA-S65
7.16 7.11 5.21 3.69 3.68 3.67 3.66 3.22 3.21 3.20 3.20 3.19 3.18 3.17 3.16 3.15 2.78 2.77 2.75 2.75 2.73 2.73 2.16 2.16 2.16 2.15 2.15 2.14 2.14 2.13 2.12 1.76 1.75 1.75 1.73 1.73 1.73 1.72 1.35 1.33 1.33 1.31 1.19 1.19 1.17 1.17 1.03 1.02 1.01 0.94 0.92 0.45 0.45 0.39 0.33 0.32 0.23 0.09
1.00
3.10
5.6
5.4
ppm
2.01
1.00
0.99
5.01
2.48
2.50
2.51
12.07
6.11
3.01
12.58
3.18
9.31
3.06
3.01
8.5 8.0 7.5 7.0 6.5 6.0 5.5 5.0 4.5 4.0 3.5 3.0 2.5 2.0 1.5 1.0 0.5 0.0 ppm

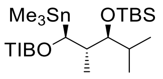

$^{13}C\{^{1}H\}$ NMR (101 MHz, C_6D_6)

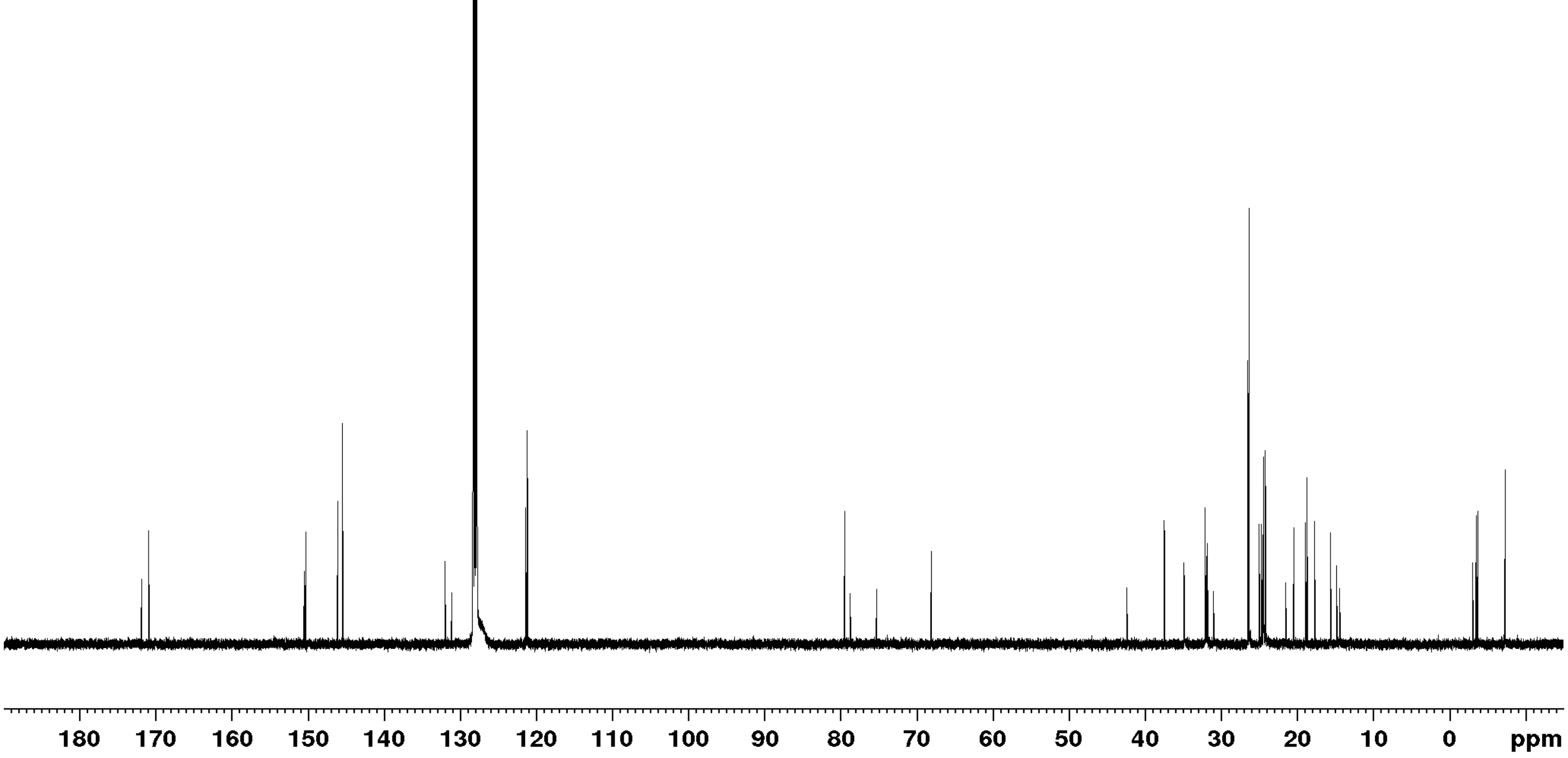

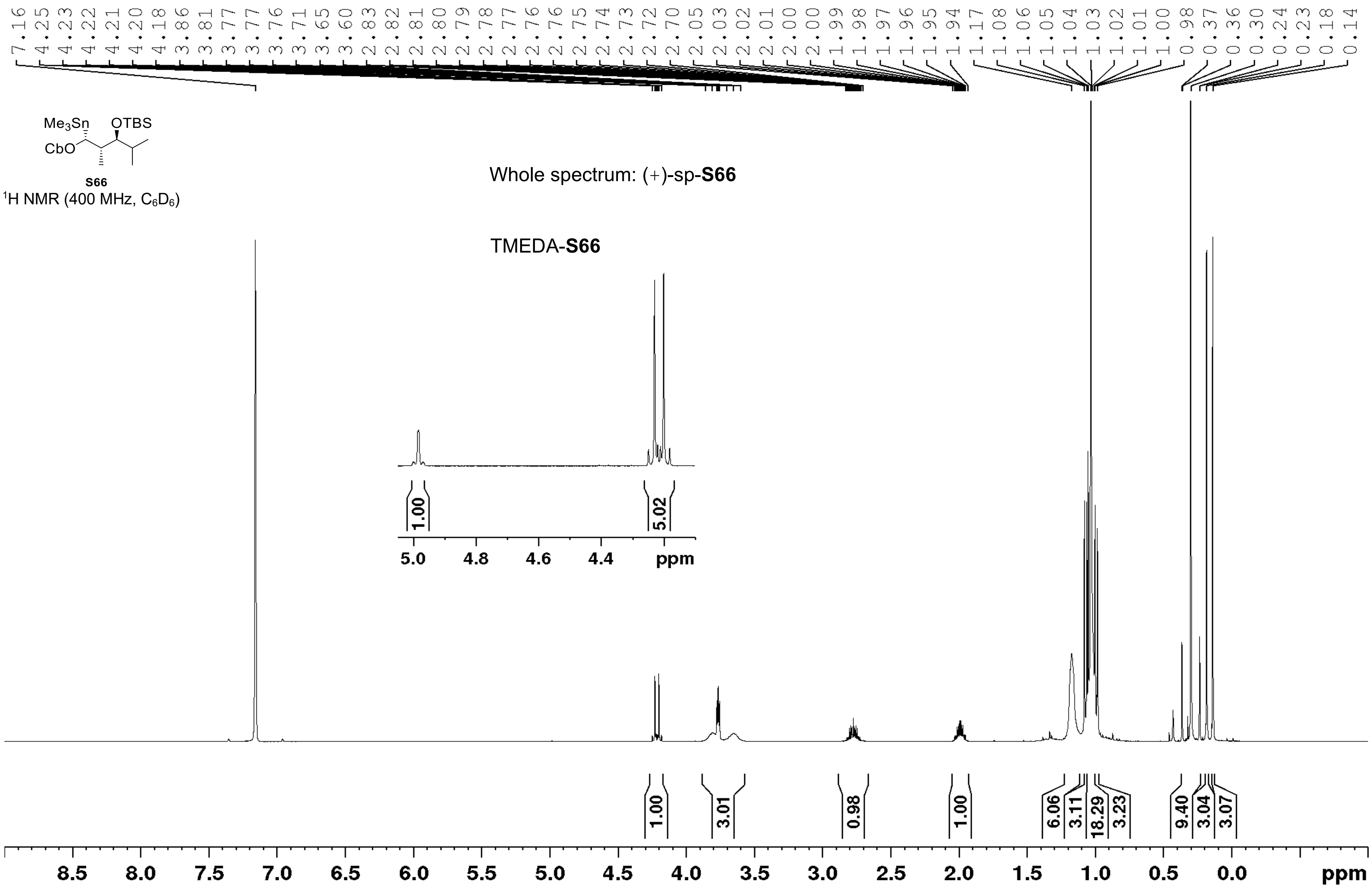
7.16 4.25 4.23 4.22 4.21 4.20 4.18 3.86 3.81 3.77 3.77 3.76 3.71 3.65 3.60 2.83 2.82 2.81 2.80 2.79 2.78 2.77 2.76 2.76 2.75 2.74 2.73 2.72 2.70 2.05 2.03 2.02 2.01 2.00 2.00 1.99 1.98 1.97 1.96 1.95 1.94 1.17 1.08 1.06 1.05 1.04 1.03 1.02 1.01 1.00 0.98 0.37 0.36 0.30 0.24 0.23 0.18 0.14
Me3Sn
OTBS
CbO
S66
1H NMR (400 MHz, C6D6)
Whole spectrum: (+)-sp-S66
TMEDA-S66
1.00
5.02
5.0 4.8 4.6 4.4 ppm
1.00
3.01
0.98
1.00
6.06
3.11
18.29
3.23
9.40
3.04
3.07
8.5 8.0 7.5 7.0 6.5 6.0 5.5 5.0 4.5 4.0 3.5 3.0 2.5 2.0 1.5 1.0 0.5 0.0 ppm

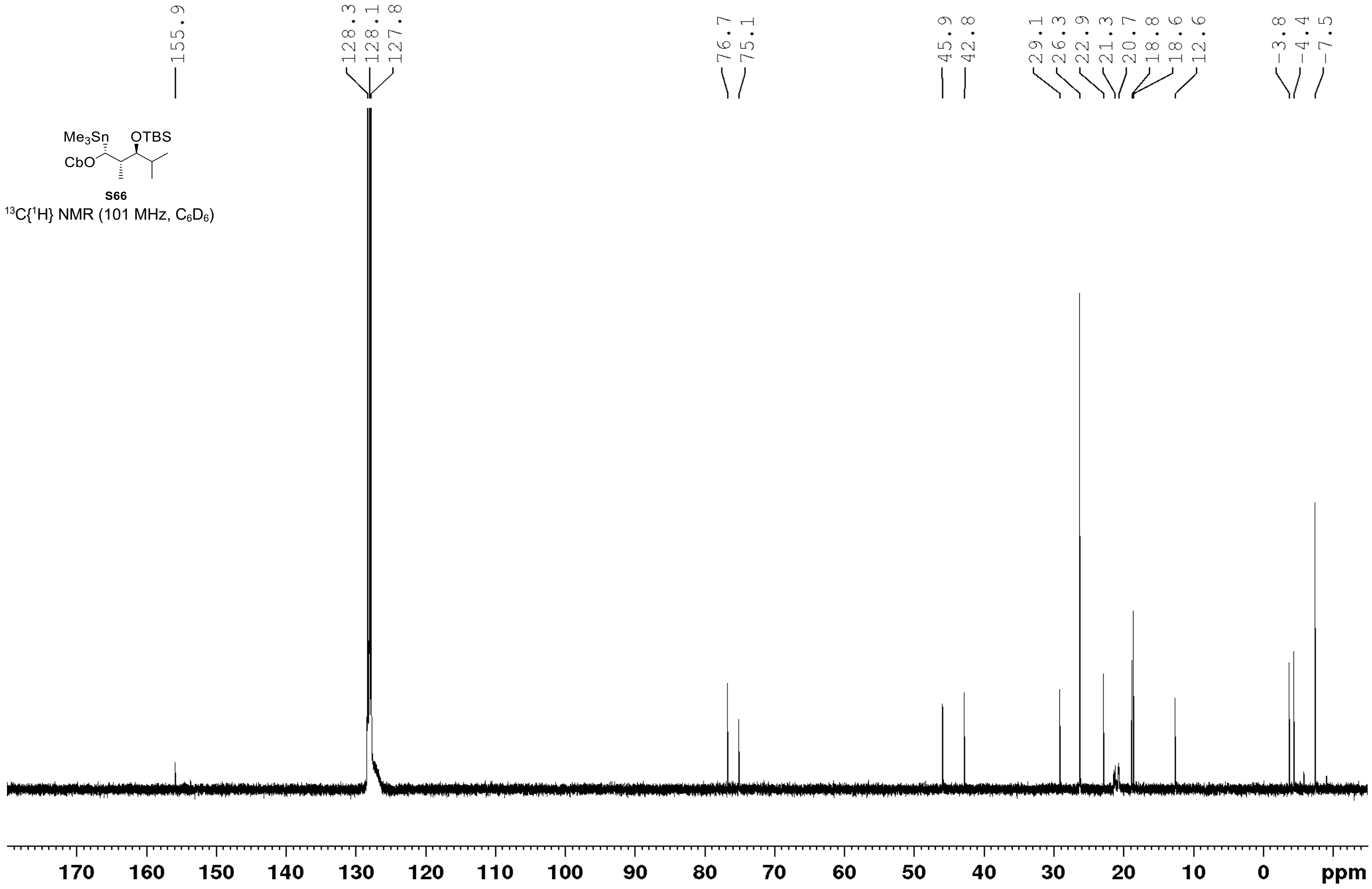
155.9
128.3
128.1
127.8
76.7
75.1
45.9
42.8
29.1
26.3
22.9
21.3
20.7
18.8
18.6
12.6
-3.8
-4.4
-7.5
Me3Sn
OTBS
CbO
S66
13C{1H} NMR (101 MHz, C6D6)
170 160 150 140 130 120 110 100 90 80 70 60 50 40 30 20 10 0 ppm

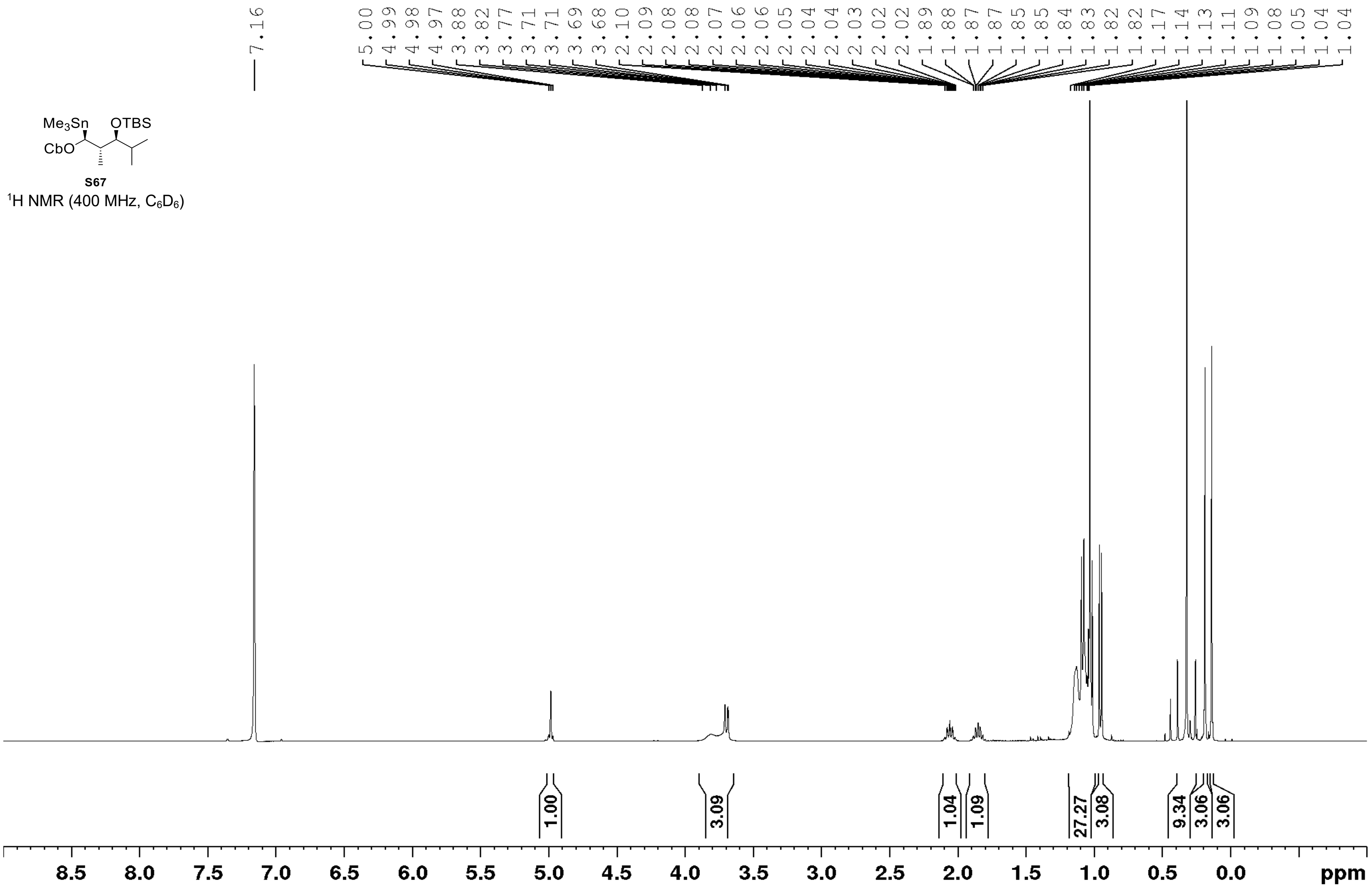

Me3Sn
OTBS
CbO
S67
1H NMR (400 MHz, C6D6)
7.16
5.00 4.99 4.98 4.97 3.88 3.82 3.77 3.71 3.71 3.69 3.68 2.10 2.09 2.08 2.08 2.07 2.06 2.06 2.05 2.04 2.04 2.03 2.02 2.02 1.89 1.88 1.87 1.87 1.85 1.85 1.84 1.83 1.82 1.82 1.17 1.14 1.13 1.11 1.09 1.08 1.05 1.04 1.04
1.00
3.09
1.04
1.09
27.27
3.08
9.34
3.06
3.06
8.5 8.0 7.5 7.0 6.5 6.0 5.5 5.0 4.5 4.0 3.5 3.0 2.5 2.0 1.5 1.0 0.5 0.0 ppm

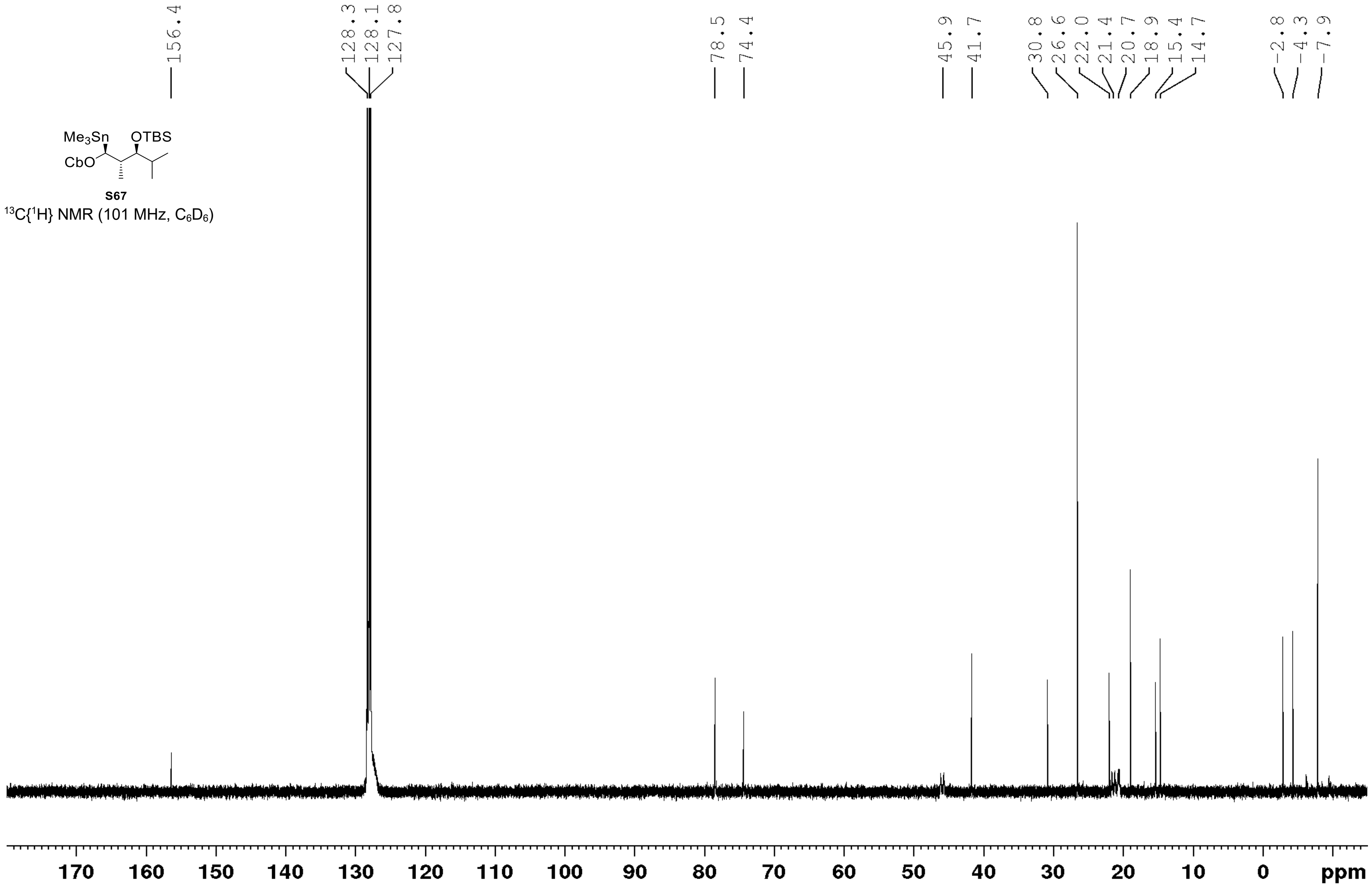

156.4
128.3
128.1
127.8
78.5
74.4
45.9
41.7
30.8
26.6
22.0
21.4
20.7
18.9
15.4
14.7
-2.8
-4.3
-7.9
Me3Sn
OTBS
CbO
S67
13C{1H} NMR (101 MHz, C6D6)
170
160
150
140
130
120
110
100
90
80
70
60
50
40
30
20
10
0
ppm

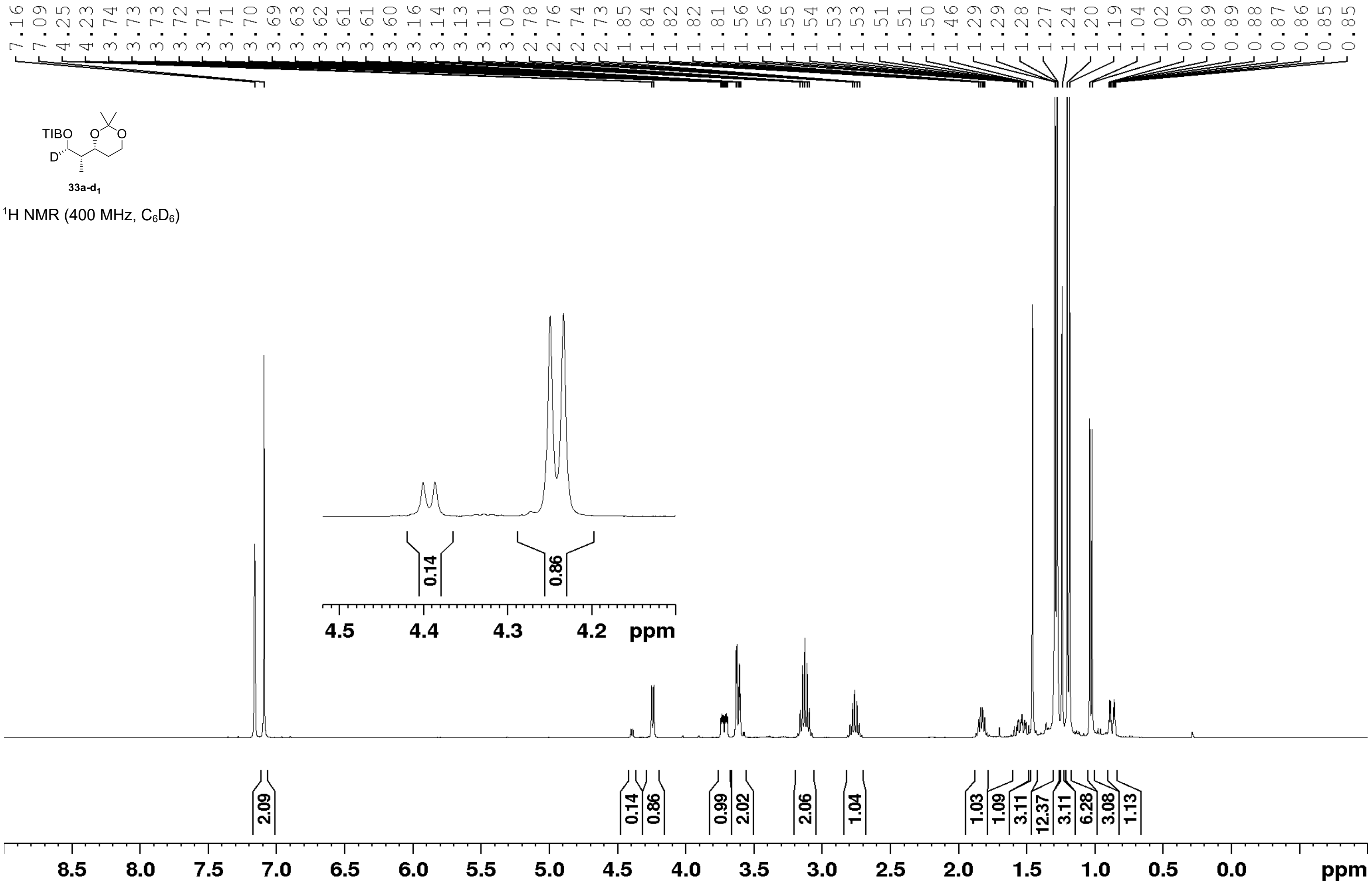

33a-d1

^{1}H NMR (400 MHz, C_6D_6)

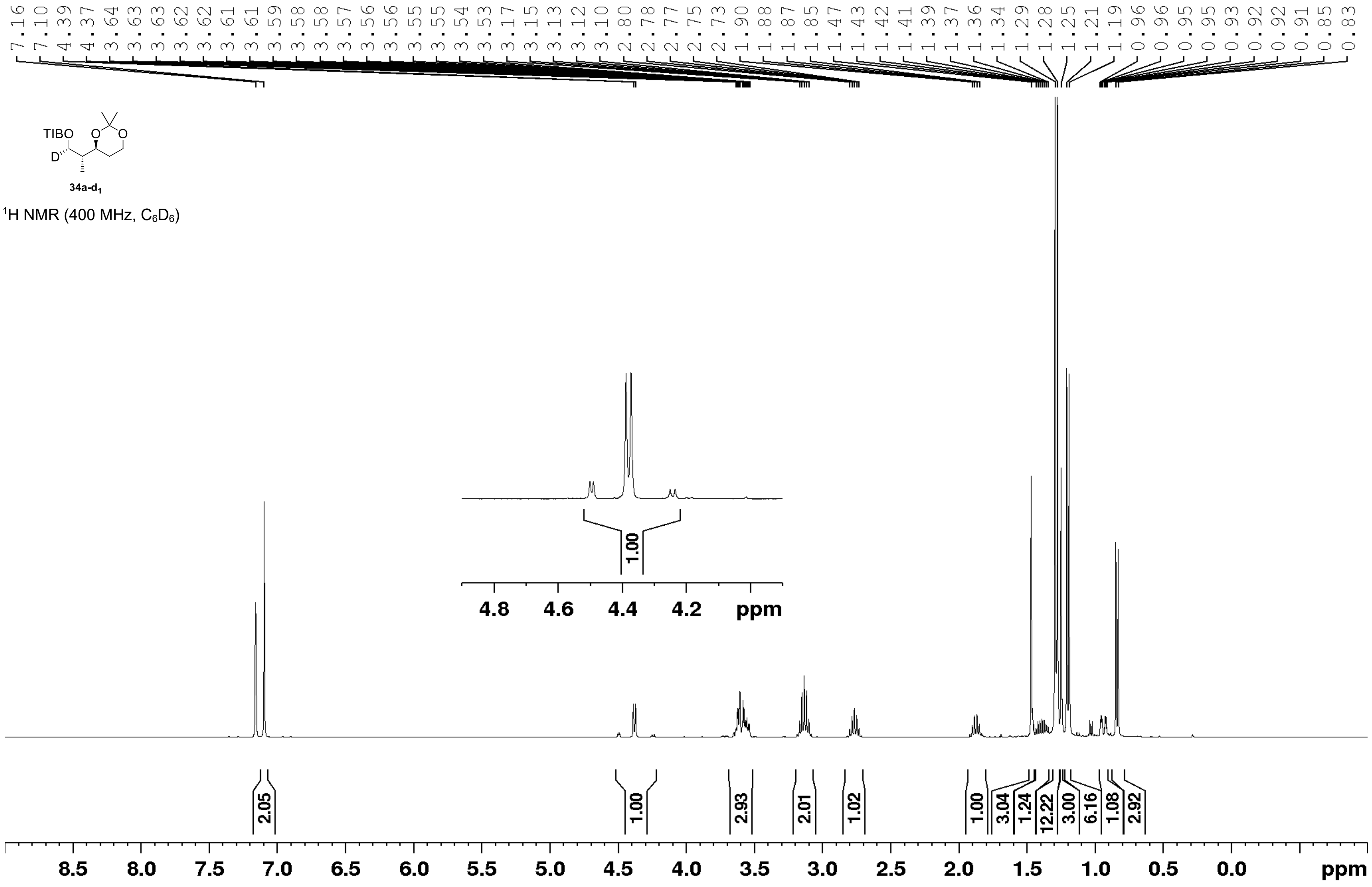

34a-d$_1$

^{1}H NMR (400 MHz, C_6D_6)

33b-d1

^{1}H NMR (400 MHz, C_6D_6)

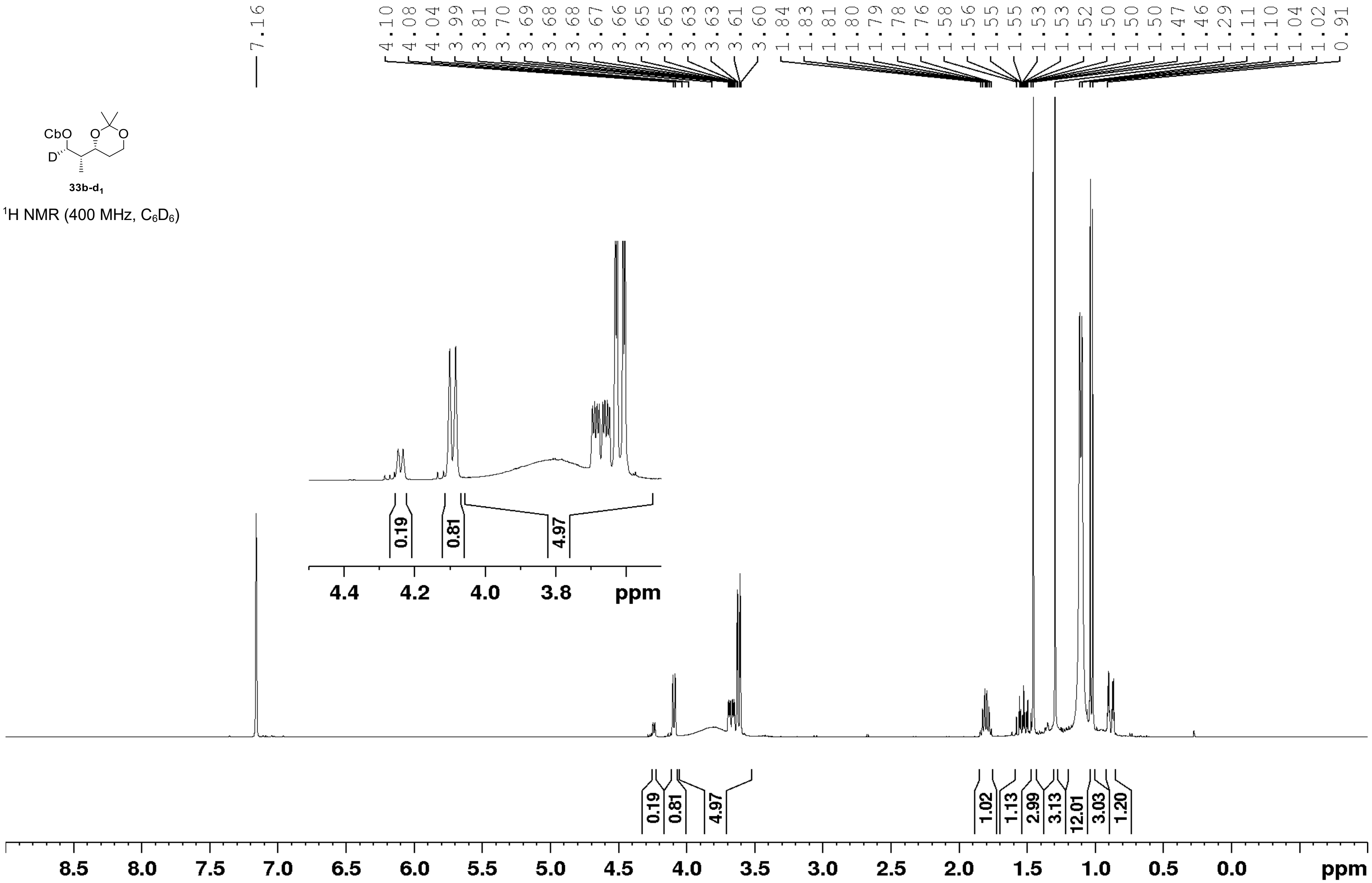

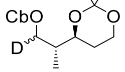

34b-d₁

^{1}H NMR (400 MHz, C_6D_6)

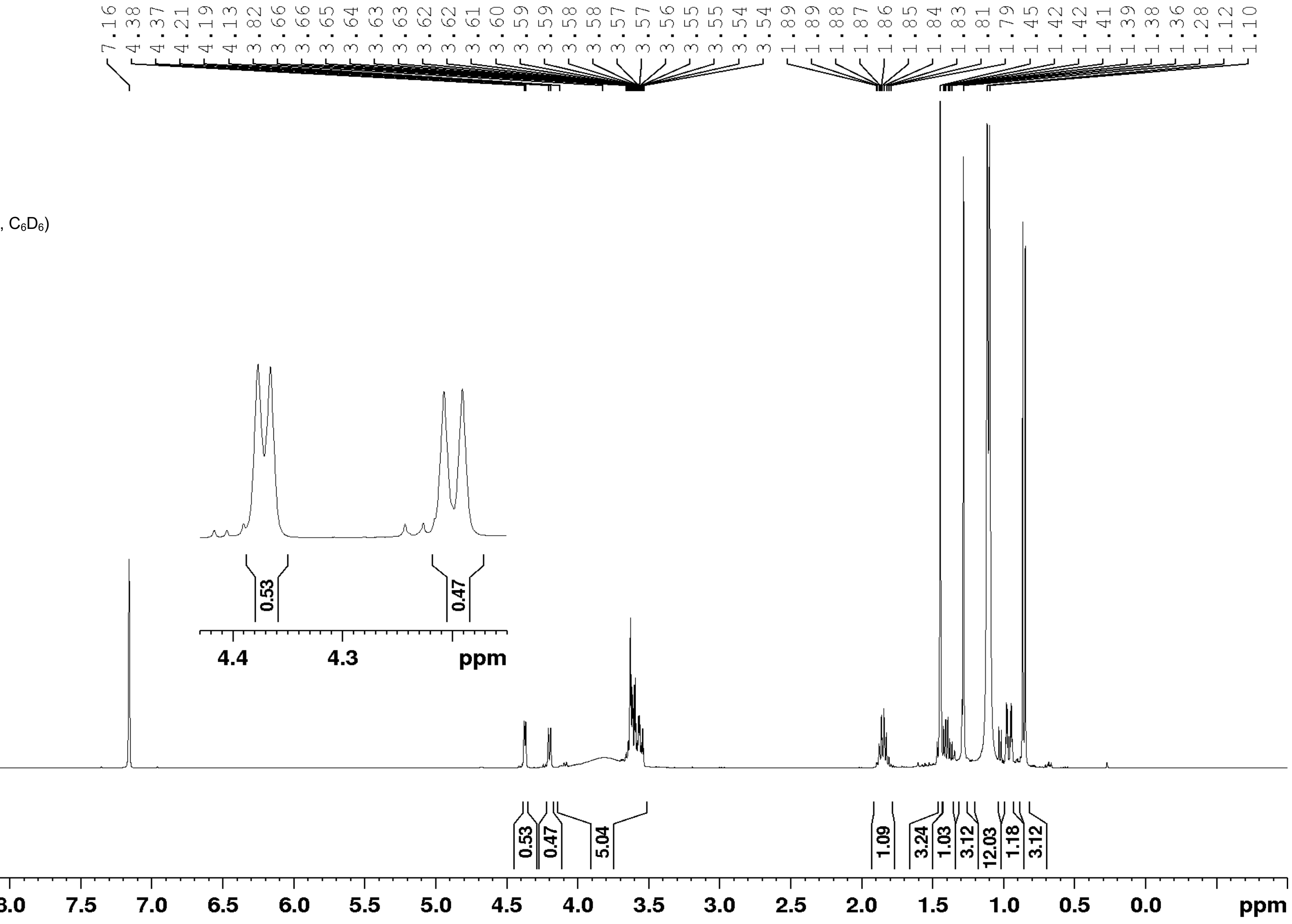

Supplement: Supplementary file 1 [file jo5c01854_si_001.pdf]
